# Recollection of participating in a trial: A qualitative study of patients with severe and very severe chronic obstructive pulmonary disease

**DOI:** 10.1371/journal.pone.0204701

**Published:** 2018-09-27

**Authors:** Claudia Véron, Sophie Pautex, Catherine Weber, Jean-Paul Janssens, Christine Cedraschi

**Affiliations:** 1 Department of Community Medicine, Primary Care and Emergency Medicine, Geneva University Hospitals, Geneva, Switzerland; 2 Division of Pulmonary Diseases, Geneva University Hospitals, Geneva, Switzerland; 3 Division of General Medical Rehabilitation, Geneva University Hospitals, Geneva, Switzerland; 4 Division of Clinical Pharmacology and Toxicology, Geneva University Hospitals, Geneva, Switzerland; National and Kapodistrian University of Athens, SWITZERLAND

## Abstract

**Background:**

Despite having similar palliative needs to patients with lung cancer, advanced chronic obstructive pulmonary disease (COPD) patients are less likely to receive palliative care. To evaluate the effect of introducing specialized palliative care with severe to very severe COPD patients, a randomized controlled trial (RCT) was conducted in Switzerland.

**Aim:**

To explore COPD patients’ recollection of the trial, their needs and the usefulness of the palliative care interventions.

**Design and setting:**

Qualitative study with advanced COPD patients who participated in a specialized palliative care intervention, conducted in a general hospital.

**Method:**

Eighteen patients with severe to very severe COPD were interviewed about their experiences. Interviews were transcribed and thematic content analysis was performed.

**Results:**

Patients had poor recollection of the trial and difficulties understanding the palliative care intervention. No major differences were observed between patients who received the specialized intervention and those who did not. Content analysis emphasized that although they experienced disabling symptoms, participants tended to attribute their limitations to problems other than COPD and some declared that they were not sick. Patients reported restrictions due to oxygen therapy, and the burden of becoming dependent on it. This dependence resulted in intense anxiety, leading participants to focus on the present only. A strong feeling of perceived helplessness emerged from the patients’ interviews.

**Conclusions:**

Our findings suggest that poor recollection and understanding of the palliative care intervention act as barriers to the conduct of clinical trials with severe and very severe COPD patients. Their cognitive difficulties, perception of COPD, functional limitations, overwhelming anxiety, focus on the present and perceived helplessness also seem to hinder the implementation of such care.

## 1. Introduction

Chronic obstructive pulmonary disease (COPD) is a progressive lung disorder that causes important mortality and morbidity worldwide [1]. Severe COPD is associated with disabling physical symptoms, emotional distress, social isolation and poor quality of life [2, 3]. The illness trajectory of COPD has been described as one of long-term limitations with recurrent exacerbations that can result in death [4]. Within 2 years after admission for an acute exacerbation, mortality rates are between 36–50% [5].

The unpredictable illness trajectory of COPD makes it difficult to determine prognosis and can be a barrier to the provision of palliative care for these patients [6]. Despite having similar palliative needs to patients with lung cancer, studies have shown that COPD patients are less likely to receive palliative care than patients with lung cancer [2, 7]. Many COPD patients have limited access to palliative care services [2, 8–10]. Furthermore, patients with moderate to severe COPD often report infrequent and poor-quality communication about end-of-life care with their physicians [11, 12].

A randomized controlled trial (RCT) was conducted in Switzerland to evaluate the effect of introducing specialized palliative care for patients with severe and very severe COPD [13]. The primary objective of this study was to assess the impact of early specialized palliative care on hospital, intensive care unit and emergency admissions of these patients. Preliminary results show no significant differences between the intervention and control group in terms of exacerbation, hospital and intensive care unit admissions, or on scores for anxiety and depression. The results of the trial will be presented elsewhere.

Little is known about the views of advanced COPD patients on palliative care. To better understand the experiences of these patients with a specialized palliative care consultation, we undertook a qualitative study as a supplement to the above-mentioned RCT. More specifically, we investigated their recollection of participating in the trial and their particular needs at this stage of the disease. The results of this study could provide insights as to how patients suffering from this life-threatening lung disease remember and benefit from palliative care interventions and, conversely, on the possible barriers to the conduct of clinical trials and the introduction of such care with these patients.

## 2. Material and methods

### 2.1 Design/Sampling

The randomized controlled trial [13] was a 3-year single center study with a 2 arms parallel groups design. Inclusion criteria were patients with COPD defined according to the Global Initiative for Chronic Obstructive Lung Disease (GOLD) criteria with a stage III or IV disease and/or long-term treatment with domiciliary oxygen and/or home mechanical ventilation and/or one or more hospital admissions in the previous year for an acute exacerbation. Exclusion criteria were patients with cognitive impairment (<23 on the Mini Mental State Examination (MMSE)) and/or with active cancer and/or in their last days of life.

The 49 patients included were randomly allocated either to the intervention group that benefited from an early specialized palliative care intervention in addition to standard care, or to the control group that benefited from standard care. The early palliative care intervention consisted of the visit of experienced nurses specialized in palliative care once a month during one year, and focused on symptom management, nutrition, understanding of illness and coping with the disease, anticipation and decision-making, support of relatives, socio-spiritual needs, coordination between different health providers and proposing alternative approaches such as relaxation, reflexology and massages.

**Fig 1:**
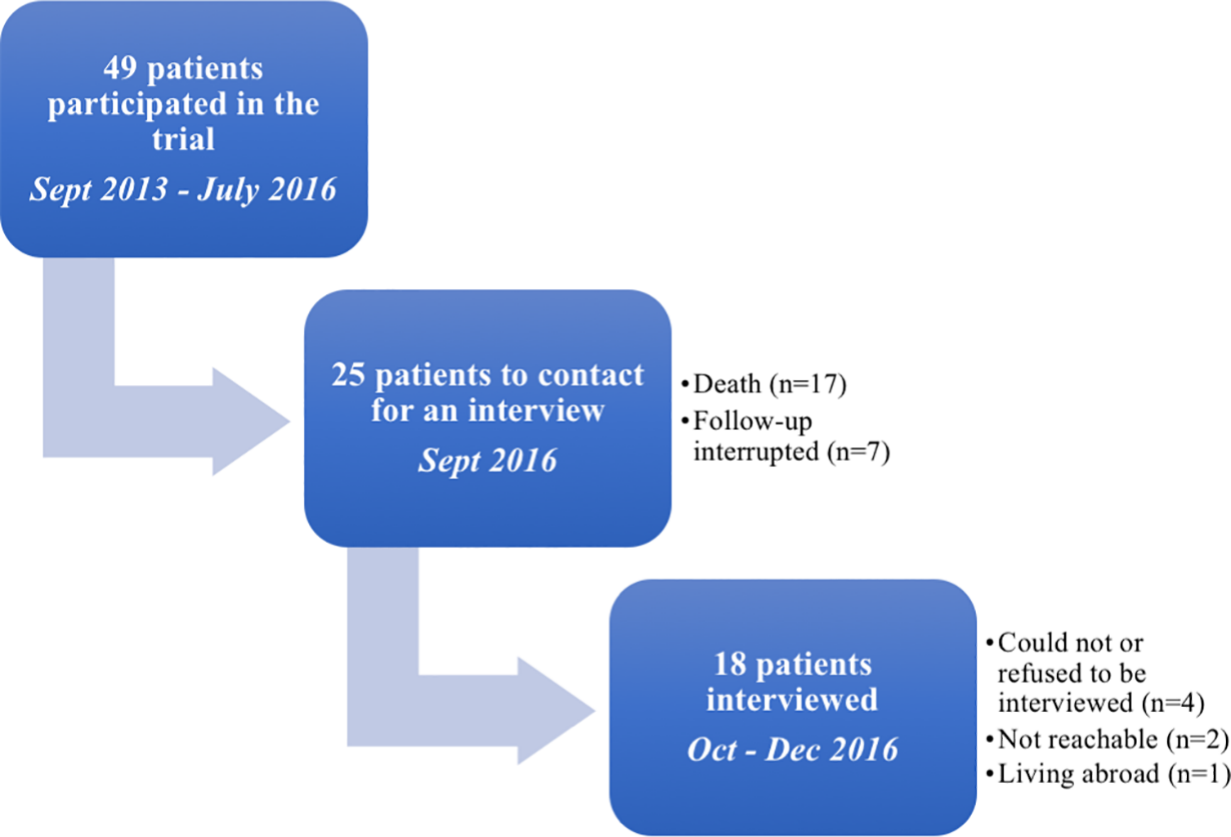
Flow chart of sampling.

At the end of the trial, the research team informed surviving participants that a psychologist (CV) would contact them for an interview about their participation in the study and their experience with COPD. Among the 49 patients included in the RCT, 17 patients died since the beginning of the trial. The follow-up of 7 patients was interrupted during the trial, leaving only 25 patients to contact for an interview. Among those, 4 patients could not or did not want to be interviewed notably because of their poor health condition, 2 individuals could not be joined by telephone and one person was living abroad. Eighteen semi-structured interviews were finally conducted with the surviving patients between October and December 2016 (Fig 1).

The study was approved by the Cantonal Research Ethics Committee in Geneva CCER (n°PB_2016–02208 (13–102) and the trial has been registered at clinicaltrials.gov under NCT02223780. Informed written consent was obtained from all patients before the interviews. In terms of procedures, the consolidated criteria for reporting qualitative research (COREQ) were followed [14].

### 2.2 Data collection

A psychologist (CV) carried out the interviews with patients alone at their home. In order to obtain in-depth qualitative information, two topic guides (one for the intervention group, one for the control group) were developed from reference to the literature and brainstorming with the multidisciplinary research team (one pulmonologist JPJ, two palliative care doctors CW and SP, two psychologists CC and CV) (S1 Text). The interview guides focused on patient’s participation to the study (expectations), their opinions on the specialized palliative care received/usual care received (overall appreciation, information received, symptom management, psychological support, spiritual support, advanced directives), support from family and friends (support received, needs, involvement in the care provided), the needs and usefulness of a specialized palliative care and their unmet needs. After a first question inviting the patients to share their experience of their participation in the trial, open-ended questions were used to generate further narratives in relation to the topics of the interview guide. All interviews were tape recorded, anonymized and transcribed (S2 Text). Field notes were taken after each interview.

### 2.3 Data analysis

Using thematic content analysis methods [15], two research psychologists (CC and CV) simultaneously and independently read and analyzed the interviews. The analysis led to the identification of recurring categories and themes. Iterative analysis was performed throughout data collection until no new categories and themes emerged from transcript analysis [16, 17]. CC and CV met frequently to discuss their findings and to reach consensus on the qualitative analysis grid. The grid was then submitted to the research team (JPJ, CW, SP) for verification [18], with the transcripts of two interviews, and modified in light of their review. Interview coding was performed in Microsoft Excel spread sheets, using the final grid. Finally, the multidisciplinary research team (JPJ, CW, SP, CC, CV) discussed the findings according to their areas of expertise and previous research findings until the emergence of a consensus.

## 3. Results

Among the 18 interviews conducted (10 men, 8 women), 11 patients had been randomized to the palliative care group of the RCT (7 men, 4 women) and 7 patients to the usual care group (3 men, 4 women). Patients were aged between 56 and 82 years old (Table 1). There were no significant differences between the subgroup of patients interviewed and the total of patients included in the RCT for socio-demographic, anthropometric and subjective variables. Interviews lasted 52 minutes on average.

**Table 1:** Characteristics of the 18 patients interviewed.

Sex (male/female)	10/8
Age (years; mean (SD; range))	72 (7; 56–82)
Living with family carer (number of patients)	4
Domiciliary oxygen (number of patients)	10
FEV1 (%; mean (SD))^a^	35.4 (18.6)
Hospitalisations during the year (number of patients)	11
Hospital anxiety and depression scale, anxiety subscore (mean (SD))^b^	8.6 (4.3)
Hospital anxiety and depression scale, depression subscore (mean (SD))^c^	5.7 (3.3)

Data collected at RCT inclusion, except for participants’ age registered at interview.

^a^ FEV_1_ = forced expiratory volume in one second.

^b^ Scores between 8 and 10 indicate a suspected anxiety disorder.

^c^ Scores up to 7 indicate an absence of depression disorder.

### 3.1 Overview of findings

The first finding of this study is that no major differences could be observed between the group of patients who received a specialized palliative care intervention and those who benefited from standard care. Because of this lack of differences, we decided to carry out a single thematic content analysis for the whole of the 18 interviews that focuses on patients’ experiences of the trial and of living with COPD. The key findings of this analysis can be grouped into the following topics: (1) “nothing was done for me”, (2) “I’m not sick”, (3) functional limitations, (4) overwhelming anxiety, (5) focus on the present and (6) perceived helplessness.

### 3.2 “Nothing was done for me”

A majority of participants related a lack of understanding of the purpose of the study and questioned its usefulness. The patients in the intervention group mainly remembered completing study questionnaires but did not seem to remember and/or acknowledge the palliative care consultation as such.

*“Well*, *we didn’t talk much*, *we did this questionnaire*, *and then they [the research nurses] would leave*, *so*. *No*, *there wasn’t any discussion like I have with you [interviewer] today (…)*. *There wasn’t any discussion besides the questionnaire*.*”* (Participant 11, palliative care, male, 56 years old)

Moreover, two patients couldn’t remember at all the palliative care intervention.

*“But I can’t remember*, *I can’t remember*, *you can’t remove it from me*. *I don’t want to talk nonsense to say*, *because I don’t know*, *so*, *honestly*. *I can’t even tell you how those ladies [the nurses] were*. *Probably nice*, *but…”* (Participant 10, palliative care, male, 73 years old)

Whether they belonged to the intervention or control group, most patients described that nothing was done for them as the nurses didn’t change their medication, improve their lung function nor helped with their other functional limitations.

*“But*, *but what did she come to do to me*? *She didn’t do anything to me*! *I continued as I have always done*! *So she was there to say hello to me*, *and that’s it*! *I don’t know*! *Because I can’t recall having changed treatments*, *or whatever*, *I can’t remember*! *I have the same treatment since the beginning*, *so I don’t know why*, *I don’t know*, *no*.*”* (Participant 10, palliative care, male, 73 years old)

For 3 participants, this lack of effect was in particular explained by the fact that a nurse and not a doctor performed the visits.

*“Well*, *listen*, *if it’s done by a doctor*, *OK*. *But if it’s done by a nurse*, *she can at the most report to the doctor what she has seen*, *but*, *but she can’t do better*! *It’s not her who’s going to change your treatment*.*”* (Participant 6, usual care, female, 74 years old)

Only 3 patients in the intervention group reported that the monthly visits were useful. The information provided, the skills of the nurses and the support received for the management of their anxiety figured among the elements that were appreciated.

*“She [nurse] especially brought me practical elements that nobody else could give me*, *in fact*. *Doctors don’t treat us like that*. *So she did the transition between the simply human side*, *banal questions and the doctor who never answers those questions*: *‘how should I eat’ or ‘what I could do to breathe better’*.*”* (Participant 14, palliative care, female, 70 years old)

The components of the palliative care interventions such as symptom management, nutrition, disease information, or alternative approaches were rarely mentioned by the patients who benefited from this care. Indeed, patients rather reported that what they appreciated in the nurses’ home visits was having companionship, talking about other subjects than their illness and the moral support provided.

*“It was pleasant to have the visit of this lady [nurse]*, *to indulge in small talk*. *But I mean*, *it didn’t bring me anything*, *by telling me*, *by optimizing*, *by telling me ‘I’m going to live for another 10 years’*.*”* (Participant 9, palliative care, male, 70 years old)

### 3.3 “I’m not sick”

Although they suffered from advanced COPD with disabling symptoms, participants tended to talk more about other health problems they suffered from than about their COPD. Moreover, some attributed their functional limitations to aging rather than their illness and 5 patients even declared that they were not sick.

*“But I’m OK*, *I cope with it*, *you tell me*: *‘you have a radiant glow’*. *I say*: *‘yes*, *but I’m not sick*, *that’s it’ (laughs)*.*”* (Participant 2, usual care, female, 74 years old)

*“Yeah*, *I never believed in COPD*. *(…)*. *I didn’t feel sick actually*.*”* (Participant 18, usual care, male, 78 years old)

Participants thus appeared to privilege avoidant coping strategies such as denial or distraction over active coping strategies such as anticipation or information seeking.

*“And all of this I managed to evacuate*, *not thinking about the illness anymore but rather about silly things*, *jokes*, *puns*. *And it’s a way for me to evacuate basically*. *And that’s what saves me a bit*. *(…)*. *If we think about it*, *we keep talking about it*, *it becomes burdensome*. *Yes*. *Too burdensome*.*”* (Participant 7, palliative care, male, 71 years old)

### 3.4 Functional limitations

Most patients expressed suffering from restrictions in daily activities. In particular, all but 2 participants reported difficulties in moving from one place to another because of dyspnea, oxygen therapy and/or aging. With the worsening of COPD, patients then mentioned the burden of becoming dependent on others and on oxygen to help them function.

*“And I felt dependent all the same*. *I felt tied to here in the house*, *the wire is 15 meters long*. *I felt as if I was kept on a leash*! *And I couldn’t do anything without it*.*”* (Participant 3, usual care, female, 78 years old)

*“We already have the constraint at the end of the line*, *so you can keep the rest of constraints*.*”* (Participant 6, usual care, female, 74 years old)

Furthermore, the difficulties encountered by patients in organizing oxygen therapy were seen as barriers to engaging in activities such as shopping for food, taking public transports or participating in social encounters. Some participants also shared that oxygen therapy exposed them to stigmatization, which could lead to social isolation.

*“With my illness*, *I can walk*, *I can breathe*, *it’s ok*. *I could continue like that*, *if I remain stable*, *why not*. *But the oxygen is destroying me… It’s the oxygen*. *I only feel sick now*. *Before I was sick*, *but we couldn’t see it*. *It means that I knew it*, *but people didn’t see it*. *(…) Maybe I’m less breathless when I walk*, *it’s true*. *But in my head*, *no*, *morally no it’s not helping me*.*”* (Participant 15, palliative care, female, 70 years old)

This sense of stigma, increased limitations of daily activities, feeling of dependence and unpredictable aggravation of COPD were identified as major sources of suffering.

### 3.5 Overwhelming anxiety

Throughout the patients’ interviews, a strong feeling of living in a constant state of anxiety emerged. Anxiety was related to the fear of running out of oxygen (with oxygen therapy), having a respiratory distress, the aggravation of their COPD and death.

*“So it stresses me out and it’s frightening to always be dependent on oxygen*, *on tubes (…)*. *And it’s a very*, *very frightening disease*, *I have to say*. *(…) if there’s a power cut*, *I’m out of oxygen*. *(…) so you’re always at the mercy of a technical problem*. *And a person who is*, *who can move around*, *she opens the window*, *she can breathe*, *she’s thirsty*, *she will be able to go and get water*. *We are very handicapped by this problem*.*”* (Participant 6, usual care, female, 74 years old)

Moreover, they felt physically vulnerable to climate changes, microbes or technical problems with their oxygen supplies and at the mercy of their unpredictable disease. This anxiety was strongly associated to the dependence on others and on oxygen therapy generated by the worsening of COPD and the increase of functional limitations.

### 3.6 Focus on the present

It appeared through the interviews that the patients tended to focus on the present and avoid talking about the future.

*“So*, *I knew more or less what to expect*. *Even if we refuse to believe or think about what happens next*. *No*, *no*, *we want to live the moment but we don’t want to know what happens next*. *And when you’re in the moment after*, *it’s difficult to accept*.*”* (Participant 6, usual care, female, 74 years old)

Participants shared indeed living one day at a time and adapting their daily activities according to how they felt and to the weather. Throughout the interviews, narratives on the history of their COPD were poor and discussions about end-of-life issues were often avoided. As a matter of fact, some patients clearly eluded advance directives regarding end-of-life care.

*“And I still have the brochure [on advance directives] here*, *I know exactly what it is*, *what*. *But when I’ll be*, *when I’ll be up to it*, *when I’ll feel better*, *when maybe I’ll be set differently in my mind*, *yes I’ll do it (laughs)*.*”* (Participant 4, palliative care, female, 68 years old)

When asked about their specific needs, they mostly reported practical needs to help with their daily functioning and didn’t wish for any spiritual support. Moreover, most participants expressed wanting to be cured and to be able to breathe. Some wished to be supported and to have their anxiety reduced.

### 3.7 Perceived helplessness

A strong feeling of perceived helplessness emerged from the patients’ interviews. COPD was described as an incurable affection that caused a progressive and inevitable deterioration of their lungs.

*“And then that’s it*, *we go down a step*, *we try to go back up*. *But you go down two steps*, *you go back up only one*!*”* (Participant 6, usual care, female, 74 years old)

*“Anyway*, *it’s not going away*. *It’s not curable*, *if it was something that we could cure like a cancer or like that*, *then I would get informed*. *But there I don’t inform myself*, *it’s like that*!*”* (Participant 3, usual care, female, 78 years old)

In the same way, most participants mentioned that they didn’t expect anything from their participation in the trial and that they participated for science advances rather than for themselves.

*“Nothing*. *I didn’t expect anything*. *I knew it wouldn’t improve my health at all*.*”* (Participant 9, palliative care, male, 70 years old)

However, most patients expressed the wish to be healed and half of them were hoping for medical advances for COPD.

*“Yes if you find another*, *a good medication*, *let me know*! *I will take it (laughs)*. *Something so I don’t need to take this [oxygen therapy] anymore*. *They will find something one day*! *They will find something that you put under your skin that will make oxygen and that we won’t have to fill and everything*. *For sure*, *they will find something*.*”* (Participant 10, palliative care, male, 73 years old)

*“I would like a medication that makes me go back to how I was last year*, *so I can get better*.*”* (Participant 6, usual care, female, 74 years old)

## 4. Discussion

### 4.1 Barriers to conducting clinical trials with advanced COPD patients

The findings of this qualitative study provide insights as to possible barriers to conducting clinical trials and implementing palliative care interventions for people suffering from advanced COPD. The particular way in which COPD affects individuals at various levels may explain the surprising lack of recall, understanding and benefit of the palliative care intervention. As we already knew and as shown through the interviews, advanced COPD not only impairs their body, but also disturbs them at the affective, cognitive, psychological and social levels. For instance, several studies have demonstrated that cognitive dysfunctions are frequent in COPD patients, notably due to persistent hypoxemia [19–21]. Although cognitive function was not or slightly impaired at the beginning of the study, as indicated by the MMSE scores of these patients, we thus may assume that cognitive difficulties could have affected patients’ ability to remember the intervention at the time of the interview.

The fact that some patients with advanced COPD did not see themselves as sick also seems to be a major obstacle to the identification of a need for palliative care. This non-recognition of COPD as a disease was also found in other studies. Patients with severe COPD have previously described COPD as a “way of life” rather than an illness [22] or as a “vague feeling of being ill” [23]. Habraken et al [24] suggested that COPD patients did not ask for help because they did not see themselves as ill. Patients did not regard their limitations as abnormal but as part of ageing, which concurs with our findings and those of others [25]. The specific illness trajectory of COPD brings patients to adapt to their limitations and feel sick only during exacerbations [22, 24]. Our findings thus suggest a lack of comprehension of COPD and its prognosis in some patients with advanced COPD. Several studies have revealed this inadequate provision of information and the need for improved communication about end-of-life care for these patients [2, 11, 12, 23, 26–30].

Furthermore, the anxiety expressed through the interviews seems to lead patients to live one day at a time and focus on the present as thinking about the future appears to be too frightening. It feels as though COPD imposes a restriction of their mental horizon by narrowing their activities, mobility, social contacts, pulmonary and cognitive capacities and life expectancy. These increasing limitations in COPD have been well documented [23, 24, 31, 32] and seem to trap patients into a state of dependency and anxiety. If they cannot have “new lungs” or a miracle cure, patients then wish for practical solutions to help them function on a daily basis and therefore regain a sense of control over their lives. These primary needs could contribute to explain their reluctance to discuss advance directives. Our findings are in line with those of Marx et al who describe the attempts of severe COPD patients to maintain life as usual and to deny the threat to life for as long as possible [23].

The fact that most participants did not recognize palliative care as something useful could also be linked to their strong feeling of perceived helplessness. Other studies have described this feeling of “being at the mercy of the disease” [23], that they cannot be helped [24] and this form of “passive acceptance” [22]. As they feel that nothing can be done for them, patients tend to avoid actively seeking for information and help for their illness and thus seem to not know about available support besides their actual treatment. This lack of knowledge can be illustrated by the idea shared by several participants that “real” care can only be provided by a doctor who can change their treatment and not by a nurse. As reported by Spathis and Booth [33], there appears to be some misconceptions about palliative care and COPD that act as barriers to the provision of end-of-life care for these patients.

### 4.2 Strengths and limitations of the study

The 18 patients interviewed were a sample of a population selected for the RCT and thus may differ from an unselected population of patients with advanced COPD. In particular, participants in the trial had a higher level of education and a rather high proportion of home care by various care providers. The numerous healthcare visits could thus have hindered the identification of the palliative care received as part of the study. Moreover, the delay between the end of the RCT and the interview (4 to 17 months) could have contributed to the difficulty for participants to recall the intervention. The fact that the study questionnaires (every 3 months) and the palliative care intervention (every month) occurred in the same lapse of time could have participated to the sense of confusion among patients in the “palliative care” group. Finally, we cannot exclude that the cognitive status of certain patients may have deteriorated since initial screening for participation in the trial (MMSE). As this was designed as a qualitative study, we did not seek to generalize our results but to obtain a better understanding of patients’ experiences with a specialized palliative care consultation. In this context, the most notable limitation is the common challenge of transferability of the findings. As in all qualitative studies, our study sample was small and the exploration of experiences calling upon recall memory in this specific population indicates that the transferability may thus be limited to people and settings with characteristics similar to those investigated in this study.

Regardless of these limitations, our findings on patients’ experiences of living with advanced COPD are in line with other qualitative studies [22–24]. Besides contributing to the comprehension of these specific experiences, the use of qualitative research methods helped to better understand the lack of effects of the palliative intervention of the RCT. The usefulness of qualitative methods alongside RCTs to better understand complex healthcare interventions has repeatedly been underlined [34, 35]. Discussing with the multidisciplinary research team the results of the RCT in light of the findings of the qualitative study enriched the understanding of the effect of the intervention. To our knowledge, this is the first qualitative study on severe to very severe COPD patients who received early palliative care in the form of monthly visits of an experienced nurse.

### 4.3 Implications for practice and research

Specific needs in advanced COPD should be assessed before implementing palliative care. According to our results, it may be assumed that care should first focus on addressing anxiety and functional limitations issues before discussing advance directives. Bove et al recently showed that home-based psychoeducation for patients with advanced COPD helped them self-manage anxiety and dyspnea and thus increased their sense of control [36]. Hence, education and better information on available support could help to reduce patients’ feelings of perceived helplessness. Other types of interventions, such as peer support [37, 38], mentioned as beneficial by some participants in our study, may also provide moral support, tips on how to deal with COPD, and reduce social isolation and sense of stigma. As for daily life limitations, a multidisciplinary team could help patients deal with these constraints and should focus on the difficulties encountered with oxygen therapy.

When conducting research on severe and very severe COPD, the possible difficulties encountered by patients in recalling and understanding clinical interventions should be considered. In order to avoid recall bias, the lengths of time between each intervention, and between the trial and interviews when using mixed methods research, should be as short as possible.

## 5. Conclusions

Besides contributing to the comprehension of the experience of living with advanced COPD, the use of qualitative research methods helped to better understand patients’ recall of the trial and the lack of effects of the palliative care intervention. Although studies advocate the implementation of early palliative care for advanced COPD [2, 8, 39–41], different barriers still hinder the introduction of such care. Further research is needed to deeper comprehend patients’ needs [8], understanding and appreciation of palliative care. The use of qualitative research before trial [34] could provide insights on the most adequate configuration of palliative care interventions for these patients.

## Supporting information

S1 Text Interview guides. (PDF)

S2 Text Full transcript of the interviews. (PDF)
