# Supplementary material for: Recollection of participating in a trial: A qualitative study of patients with severe and very severe chronic obstructive pulmonary disease
Source: PLoS One. 2018 Sep 27;13(9):e0204701. doi: 10.1371/journal.pone.0204701 (PMC6160180; doi:10.1371/journal.pone.0204701)
Supplement: S1 Text — (PDF) [file pone.0204701.s001.pdf]

S1_Text : Interview guides

Guide d'entretien / *Groupe intervention*

Consigne
Vous avez participé à une étude qui avait pour objectif de comparer le traitement habituel de votre maladie pulmonaire (BPCO) avec une prise en charge précoce, soutenue et intégrée. Vous étiez dans le groupe bénéficiant de cette prise en charge globale spécialisée et vous avez reçu la visite d'une infirmière une fois par mois pendant un an. Pouvez-vous me raconter comment cela s'est passé ?

Thèmes	Sous-thèmes	Relances
1. Participation à l'étude	Attentes en termes de participation à l'étude	– *Qu'est-ce que vous attendiez en participant à cette étude ?*
2. Appréciation de la prise en charge globale spécialisée et besoins	Appréciation globale	– *Qu'est-ce qui vous a plu ou déplu dans les visites de l'infirmière ?*<br>– *Qu'auriez-vous souhaité recevoir en plus ou en moins ?*
	Informations reçues lors des visites (sur la BPCO, son traitement, son évolution, etc.)	– *Quelle est votre appréciation des informations reçues (sur la BPCO, son traitement, son évolution, etc.) lors des visites de l'infirmière ?*<br>– *Quelles informations supplémentaires auriez-vous aimé avoir ?*
	Gestion des symptômes physiques (difficulté respiratoire, douleur, insomnie, fatigue, etc.)	– *Comment les visites de l'infirmière et les informations reçues vous ont-elles aidées, ou non, à gérer les symptômes de la BPCO?*<br>– *De quoi d'autre auriez-vous eu besoin?*
	Soutien psychologique (gestion des émotions, souffrance, etc.)	– *Que pouvez-vous me dire sur le soutien psychologique apporté lors des visites de l'infirmière ?*<br>– *De quoi d'autre auriez-vous eu besoin ?*
	Soutien spirituel (discussion autour de la fin de vie, etc.)	– *Que pouvez-vous me dire sur le soutien au niveau spirituel apporté lors des visites de l'infirmière ?*<br>– *De quoi d'autre auriez-vous eu besoin ?*
	Directives anticipées (décision autour des soins en fin de vie)	– *Lors de ces visites, avez-vous abordé les types de soins souhaités en fin de vie ?*<br>– *Si oui, comment se sont passés ces échanges ?*
3. Soutien des proches	Soutien reçu des proches	– *Quel soutien avez-vous reçu de la part de vos proches ?*<br>– *De quoi d'autre auriez-vous eu besoin ?*
	Proches et visites de l'infirmière	– *Comment se sont déroulées les visites de l'infirmière pour vos proches ?*
4. Utilité d'une prise en charge globale spécialisée	Appréciation de l'utilité d'une prise en charge globale spécialisée	– *Quelle a été pour vous l'utilité des visites de l'infirmière ?*
	Besoins non satisfaits	– *De quoi auriez-vous eu besoin ?*
	Besoins actuels	– *De quoi auriez-vous besoin aujourd'hui ?*

Interview guide[1] / *Intervention group*

First question
You participated in a study designed to compare usual care for your pulmonary disease (COPD) with early integrated care. You were in the group that benefited from this comprehensive and specialized care and you received the visit of a nurse once a month for a year. Could you tell me how this was for you?

**Themes**	**Sub-themes**	**Probes**
5. Participation in the study	Expectations	– *What did you expect from participating in this study?*
6. Appreciation of comprehensive and specialized care received and needs	Overall appreciation	– *What did you like or dislike in the nurse's visits?*<br>– *What else would you liked to have had?*
	Information received during the visits (on COPD, COPD treatment, disease evolution)	– *What is your appreciation of information received during the nurse's visits (on COPD, COPD treatment, disease evolution, etc.)?*<br>– *What additional information would you have liked to have had?*
	Physical symptoms management (breathing difficulties, pain, insomnia, fatigue, etc.)	– *How did the nurse's visits and the information received help you, or not, manage the physical symptoms of COPD?*<br>– *What else would you have needed?*
	Psychological support	– *What can you tell me on the psychological support received during the nurse's visits?*<br>*What else would you have needed?*
	Spiritual support	– *What can you tell me on the spiritual support received during the nurse's visits?*<br>*What else would you have needed?*
	Advanced directives (decision regarding end-of-life care)	– *During those visits, did you discuss the type of end-of-life care you wish for? If yes, how did those discussions go?*
7. Support from family and friends	Support from family and friends	– *What kind of support did you receive from family and friends?*<br>*What else would you have needed?*
	Family and friends and nurse's visits	– *How did your family and friends react to the nurse's visits?*
8. Usefulness of comprehensive and specialized care	Appreciation of the usefulness of comprehensive and specialized care	– *How useful were the nurse's visits for you?*
	Unmet needs	– *What would you have needed?*
	Current needs	– *What would you need now?*

[1] Translated from French to English.

Guide d'entretien / *Groupe contrôle*

Consigne
Vous avez participé à une étude qui avait pour objectif de comparer le traitement habituel de votre maladie pulmonaire (BPCO) avec une prise en charge précoce, soutenue et intégrée. Vous avez été dans le groupe bénéficiant des soins habituels. Pouvez-vous me dire comment cela s'est passé ?

**Thèmes**	**Sous-thèmes**	**Relances**
1. Participation à l'étude	Attentes participation	– *Qu'est-ce que vous attendiez en participant à cette étude ?*<br>– *Pourquoi avez-vous accepté de participer à cette étude ?*
	Réaction participation au groupe bénéficiant des soins habituels	– *Quelle a été votre réaction en apprenant que vous faisiez partie du groupe bénéficiant des soins habituels ?*
2. Appréciation des soins reçus et besoins	Appréciation générale	– *Quelle est votre appréciation générale des soins reçus dans le cadre de votre maladie pulmonaire ?*<br>– *De quoi d'autre auriez-vous eu besoin?*
	Informations reçues (sur la BPCO, son traitement, son évolution, etc.)	– *Quelle est votre appréciation des informations reçues (sur la BPCO, son traitement, son évolution, etc.)?*<br>– *Quelles informations supplémentaires auriez-vous aimé avoir?*
	Gestion des symptômes physiques (difficulté respiratoire, douleur, insomnie, fatigue, etc.)	– *Comment s'est déroulée la gestion des symptômes physiques de la BPCO ?*<br>– *De quoi d'autre auriez-vous eu besoin?*
	Soutien psychologique	– *Quel type de soutien avez-vous reçu au niveau psychologique ?*<br>– *De quoi d'autre auriez-vous eu besoin?*
	Soutien spirituel	– *Quel type de soutien avez-vous reçu au niveau spirituel ? De quoi d'autre auriez-vous eu besoin?*
	Directives anticipées (décision autour des soins en fin de vie)	– *Avez-vous discuté avec un professionnel de la santé du type de soins souhaités en fin de vie ?*<br>– *Si oui, comment se sont passés ces échanges ?*
3. Soutien des proches	Soutien des proches	– *Quel soutien avez-vous reçu de la part de vos proches ?*<br>– *De quoi d'autre auriez-vous eu besoin?*
	Proches et soins	– *Comment se sont déroulés les soins reçus dans le cadre de votre maladie pulmonaire pour vos proches ?*
4. Besoins et utilité d'une prise en charge globale spécialisée	Besoins d'une prise en charge globale spécialisée	– *Si vous aviez été dans le groupe bénéficiant d'une prise en charge globale spécialisée, quel type d'aide et informations par rapport à votre maladie pulmonaire auriez-vous souhaité recevoir ?*
	Utilité perçue d'une prise en charge globale spécialisée	– *Quelle aurait été pour vous l'utilité d'une prise en charge globale spécialisée ?*
	Besoins actuels	– *De quoi auriez-vous besoin aujourd'hui ?*

Interview guide[2] / *Control group*

First question
You participated in a study designed to compare usual care for your pulmonary disease (COPD) with early integrated care. You were in the group that benefited from usual care. Could you tell me how this was for you?

Themes	Sub-themes	Probes
5. Participation in the study	Expectations	– *What did you expect from participating in this study?*<br>– *Why did you accept to participate in this study?*
	Reaction to participating in the group with usual care	– *What was your reaction when you learnt that you were part of the group receiving usual care?*
6. Appreciation of care received and needs	Overall appreciation	– *What is your appreciation of the care you received for your pulmonary disease?*<br>– *What else would have you needed?*
	Information received (on COPD, COPD treatment, disease evolution)	– *What is your appreciation of information received (on COPD, COPD treatment, disease evolution, etc.)?*<br>– *What additional information would you have liked to have had?*
	Physical symptoms management (breathing difficulties, pain, insomnia, fatigue, etc.)	– *How did the management of the physical symptoms of COPD go?*<br>– *What else would you have needed?*
	Psychological support	– *What type of care did you receive on the psychological level?*<br>– *What else would you have needed?*
	Spiritual support	– *What type of care did you receive on the spiritual level?*<br>– *What else would you have needed?*
	Advanced directives (decision regarding end-of-life care)	– *Did you discuss with a healthcare professional the type of end-of-life care you wish for?*<br>– *If yes, how did those discussions go?*
7. Support from family and friends	Support from family and friends	– *What kind of support did you receive from family and friends?*<br>– *What else would you have needed?*
	Support and care	– *How did your family and friends react to the care you received for your pulmonary disease?*
8. Needs and usefulness of comprehensive and specialized care	Need for comprehensive and specialized care	– *If you had been in the group that received comprehensive and specialized care, what kind of care and information on your pulmonary disease would you have wished to have?*
	Perceived usefulness of comprehensive and specialized care	– *What would have been, for you, the usefulness of comprehensive and specialized care?*
	Current needs	– *What would you need now?*

[2] Translated from French to English.
