# Supplementary material for: Recollection of participating in a trial: A qualitative study of patients with severe and very severe chronic obstructive pulmonary disease
Source: PLoS One. 2018 Sep 27;13(9):e0204701. doi: 10.1371/journal.pone.0204701 (PMC6160180; doi:10.1371/journal.pone.0204701)
Supplement: S2 Text — (PDF) [file pone.0204701.s002.pdf]

# S2_Text : Full transcript of the interviews

## Participant n°1

Nom d'emprunt : Chantal
Âge : 76 ans
Sexe : F
Groupe : Intervention

**CV : Il y a environ une année, vous avez participé à cette étude qui avait pour objectif de comparer le traitement habituel de votre maladie, donc la BPCO, avec une prise en charge qui était dite précoce, soutenue et intégrée. Et vous aviez été dans le groupe qui bénéficiait de cette prise en charge globale spécialisée et là vous avez reçu la visite d'une infirmière une fois par mois pendant un an. Est-ce que vous pouvez me raconter comment cela s'est passé ?**

C : J'en ai eu trois.

**CV : Vous avez [eu trois**

C : différentes]. Je sais pas.

**CV : Trois infirmières différentes.**

C : Un groupe de trois qui venait une après l'autre. Je sais pas si c'est tous les trimestres ou tous les mois… Je me souviens même pas… quand elles venaient…

**CV : Comment ça s'est passé ?**

C : Bon elles posaient des questions, elles avaient des, des formulaires qu'elles nous posaient les questions qu'il y avait dessus. C'était toujours les mêmes questions quoi… Pis on répondait ce qu'elles demandaient quoi. Je me rappelle plus ce que c'est maintenant, c'est ça le problème. *(raclement de gorge)* J'avais jamais gardé ça… Peut-être que vous les avez ces feuilles ?

**CV : Non, non justement *(rires)*.**

C : Ah alors c'est malin *(rires)*.

**CV : J'ai même pas les [*(rires)***

C : *(rires)* ] ça c'est b', j'ai même pas ces… ces formules... Oh il aurait fallu les garder, non ?

**CV : Non non… Non.**

C : C'était, je sais pas les types de questions euh… « Comment vous sentez-vous ? » ou « Qu'est-ce que vous faites ? » xxx quoi. *(rires).* Qu'est-ce que vous voulez que je réponde quand, moi j'aimerais mieux qu'on me soigne pour quelque chose d'efficace, qu'on trouve quelque chose une fois, qu'on remet quelque chose, que de poser toujours ces questions inutiles mais… Je sais pas… Je pense qu'ils arrivent pas euh… Parce qu' moi je me fixe toujours avec l'EPFL, ils peuvent faire tellement de choses, ces grandes organisations… s'ils peuvent soulager un peu cette maladie… Parce que c'est pas marrant hein… Mais…

**CV : Vous aviez l'impression que ces questions étaient assez inutiles donc.**

C : Je me demandais à quoi ça servait dans le fond. Bon ils faisaient le nécessaire pour savoir comment… comment on prenait la chose, mais… Pis y a des fois on a pas envie de trop parler. Je sais pas. Mais elles étaient toutes très gentilles je dois dire. Mais… Mais oui, bon je me souviens plus

des questions. Je pourrais même pas, il y avait beaucoup de questions de… je sais pas « de 1 à 10, combien vous sentez-vous ? » des trucs comme ça, comme à l'hôpital vous savez.

**CV : Mais vous aviez l'impression que c'était pas vraiment efficace.**

C : Pour moi non m' *(rires)*. Bon pour certaines personnes peut-être parce que ça peut les aider, quand on est vraiment seul ça peut aider… Bon là comme j'ai de la famille un peu qui vienne assez, pas mal souvent, ça va encore, mais… Ceux qui sont réellement seuls, c'est pas marrant… Non…

**CV : Donc là vous avez de la, de la famille**

C : Oui j'ai quand même de la famille qui s'occupe de moi, mais… Mais c'est pas tout. On a toujours l'air de les ennuyer, je sais pas. 'fin je pense ça mais… Hein quand il faut toujours demander les choses c'est pénible… Alors voilà, je sais pas… J'en sais pas beaucoup plus que vous *(rires).*

**CV : *(rires).* Vous avez voilà quand même ce… ce soutien des proches, des proches qui [sont là.**

C : Oui] ben heureusement qu'il y a ça parce que… Vous savez les gens d'à côté on peut pas tellement compter dessus hein je trouve… C'est juste des « bonjour, bonjour » comme ça pis voilà maintenant y disent « Berthe j'ai vu ». On dirait qu'ils ont peur de se frotter à des gens malades, je sais pas… Euh moi je ressens ça... Ils ont de la peine euh… Ou bien ils ont pitié de nous ou bien ils… pfff… ça les ennuie de, d'aider quelqu'un vraiment hein xxx. Moi je prends souvent le bus avec ces bon' parce que j'ai la petite bonbonne là.

**CV : Oui.**

C : Alors faut voir les regards que j'ai des gamins et tout hein… Je vous dis pas, c'est pas facile *(rires)*. Alors ça je comprends pas qu'ils fassent pas autre chose, qu'on soit moins observé disons….

**CV : Par rapport justement à.**

C : Maintenant ça va parce que je me suis habituée, mais au début c'était dur hein… Bon ben des jeunes maintenant y en a qui sont bien, qui me laissent la place et tout mais… Je demande même pas parce que, je veux dire un arrêt je reste debout, c'est pas dur, là j'arrive bien…

**CV : Mais…**

C : Mais bon c'est des fois il y a des jeunes qui… des des filles souvent elles sont…

**CV : C'est dur [le regard.**

C : Ouais] les jeunes hommes il y en a des bien qui sont tout de suite, qui comprennent… Mais bon qu'est-ce que faut faire avec hein. C'est partout comme ça…

**CV : Mais… Mais pour vos proches, ils étaient là aussi quand il y avait eu les visites des infirmières ou non ?**

C : Non, non je voulais personne… Je vais pas encore leur donner du souci avec mes affaires... Il y en a eu assez comme ça cette année. Oh…

**CV : Vous avez eu pa' pas mal [de soucis cette année.**

C : Ouais y en a] eu pas mal ouais… On a eu pas mal de décès, de trucs, de maladies, alors… Des fois on en a ras le *(rires)…*

**CV : Oui ça fait… ça fait beaucoup…**

C : On peut pas comprendre, mais… C'est comme ça, c'est la vie paraît-il *(rires)*. Drôle de vie hein ?... Bon je sais pas, je peux pas tellement vous aider, parce que j'ai plus ces papiers.

**CV : Non mais… Ou qu'est-ce qui vous a plu ou déplu dans les visites de l'infirmière ? … Peut-être quelque chose qui vous revient ? ...**

C : Y a rien qui m'a déplu parce qu'elles sont toutes sympathiques et tout… Elles faisaient ça sérieusement tout en riant aussi, on faisait ça... Je peux pas vous dire… Maintenant je peux plus vous dire quelles questions elles avaient posées franchement… Mais je pense il faudrait relire et pis voir ce qu'il faudrait supprimer à la longue…

**CV : Vous sauriez pas trop me dire voilà qu'est-ce que vous auriez voulu avoir peut-être en plus ? Ou…**
C : On peut peut-être changer de… je sais pas…

**CV : Ou en moins ?**

C : Quand on se relit après, peut-être qu'on… qu'on voit des choses qu'on aurait pas dû dire. J'en sais rien… C'est pas facile comme ça… Moi je pensais que vous aviez un brouillon mais…

**CV : Oui ça fait un petit moment, donc c'est vrai que c'est pas facile toujours de se rappeler mais.**

C : Non moi je me rappelle plus… Je sais pas si j'avais encore des… Mais ça m'étonne que j'aie quelque chose…

**CV : Mmh… Et qu'est-ce que vous attendiez en participant à cette étude, quand vous avez [accepté de.**

C : Je sais] pas, j'ai accepté pour accepter. Mais je sais pas pourquoi, qu'est-ce qu'ils m'avaient demandé avec ça… Je pensais qu'ils allaient faire quelque chose de… conséquent pour moi, mais *(rires)*.

**CV : *(rires).***

C : *(Rires)* mais y'a rien *(rires)*. Y a rien eu de bien… Mais bon… C'est qu'ils ont tellement de, de choses à penser à l'hôpital, peuvent pas penser à notre cas… Je pense mais… Tout le monde attend le maximum de l'hôpital, mais ils peuvent pas tout faire… Alors je sais pas… Qu'est-ce que vous pensez-vous ?

**CV : Vous attendiez quelque chose de plus conséquent, c'est ça ?**

C : Je pensais que ça aurait servi pour d'autres choses, je sais pas. Mais je pense il y a peut-être des trucs où on peut aller là-bas, peut-être voir d'autres personnes… Je sais pas si c'est ça ? … Rencontrer d'autres gens, je pense c'est ça ou bien?

**CV : Alors euh… Là c'était vraiment juste ces visites voilà des infirmières.**

C : Ah non alors c'était pas du tout ça.

**CV : Donc là euh… Peut-être par rapport aux informations que vous avez reçues par les infirmières sur, sur la maladie, sur son évolution, euh…**

C : Bon ça j'ai lu souvent, mais je sais pas on lit pas tellement parce que ça nous fiche le bourdon d'entendre les *(rires)*… Je sais pas quoi… [Si.

**CV : Vous] vous auriez pas aimé avoir des informations supplémentaires alors ?**

C : Non ça ne sert à rien parce que ça me fiche le noir... J'aimerais mieux qu'on me dise « Oui faudrait faire ça pour améliorer, pour si, pour ça ». Là je reste à un stade pff… Je sais pas. Je suis pas médecin, j'en sais rien… Bon là ils ont, ils m'ont mis ce masque toutes les nuits hein mais… Le, vous connaissez ce truc ?

**CV : Oui.**

C : C'est pas facile déjà s'adapter avec ça. Bon je le mets, mais c'est avec… Je le mets quatre heures de temps et pis c'est tout, là je peux pas plus. Ca s'arrête automatiquement, je sais pas pourquoi. J'en sais rien…

**CV : D'accord…**

C : Ouais.

**CV : Ça c'est difficile le masque.**

C : A supporter c'est pas rien hein. J'ai mis tellement de temps à … à le supporter. Ouais c'est, et même à l'hôpital, ils étaient énervants, ils arrivaient avec ce machin. Ah je les aurais tué. *(rires).*

**CV : *(rires).***

C : J'ai de la peine avec les soins intensifs, parce que y en a une qui me saute dessus avec ce t', je dis « Vous partez, je reviens des soins intensifs, arrêtez moi ce truc, je vais pas supporter ça »… Ils avaient une tronche…. Oh mais ça c'était même pas une petite jeune, c'étAt une ancienne qui m'énervait tout le temps… Non les jeunes elles étaient sympas…

**CV : Ca c'était difficile à supporter justement.**

C : Oui c'est très difficile…

**CV : Le masque…**

C : C'est à dire qu' ça vous « mmh », ça vous serre là, pis j'avais toujours des marques partout. Oh c'était horrible… Bon maintenant je supporte un peu m', je me suis habituée parce que je suis seule... Et pis euh… bon mais quand je le pose et pis que je l'enclenche, je dors bien pendant tant de temps, c'est marqué l'heure. Peut-être quatre heures ou... J'ai fait maximum c'est cinq heures, c'est pas mal je crois. Je sais pas jusqu'à quand ça peut aller, bah pourquoi ça s'arrête tout d'un coup j'en sais rien. C'est ça que j'ai pas d'explications… Bon ils nous donnent des trucs, mais y a pas trop d'explications…

**CV : Ca vous avez… il vous manque un peu des explications ?**

C : Oui pour tout ! Parce que maintenant pour X *(entreprise qui distribue des gaz médicaux)*, j'ai… ils auraient du', j'étais embêtée le vendre', ils viennent tous les quinze jours mettre l'eau dans la la bonbonne. Et pis la veille, j'ai dit, là c'était presque à zéro, et pis le matin, je voulais remplir la petite bonbonne qu'on doit remplir avec cette grosse, je sais pas si vous comprenez ?

**CV : Oui, oui, oui.**

C : Et pis ça a giclé, ça a giclé jusqu'au plafond, comme le jet d'eau de Genève vous savez. Alors j'ai pris peur pis j'ai téléphoné. « Ah mais non, faut faire comme si, comme ça. Mais c'est rien », « Ah oui bon d'accord c'est rien » *(ton ironique)*. Mais ça continuait de plus belle, ça s'arrêtait plus. Alors j'ai retéléphoné, j'ai dit « il faut absolument que quelqu'un passe ». Y'en a un qui est passé le matin à 8 heures, il doit bien être par X *(ville)*. *(Raclement de gorge)* Pis il est quand même venu, ben il a changé carrément la bonbonne hein. C'était dangereux ce truc… Vous savez comment c'est cette bonbonne ?

**CV : Oui j'ai, j'ai vu.**

C : Ah.

**CV : Oui, oui. C'est le masque et ça fait travailler c'est ça... ça pousse.**

C : Non mais la bonbonne.

**CV : Ah oui la bonbonne qui est reliée au masque.**

C : Oui, oui… Alors ils ont toujours l'air de croire qu'on sait tout, mais… Ceux qui livrent ils devraient, je s', euh ben ils ont pas assez de personnel, j'en sais rien… [Faut touj'

**CV : Là] vous aimeriez plus de…**

C : C'est à dire que quand on a besoin de quelqu'un, il faudrait quand même que quelqu'un nous réponde. On dirait qu'on ait l'air l'air de les embêter, mais c'est pas… On fait pas ça exprès hein. Parce que voyez le matin, j'ai, ils viennent demain je crois, ils viennent porter le euh l'oxygène à huit heures. Bon il faut attendre d'ici qu'ils amènent, qui la prenne, que je remets dans la petite truc. Ca prend presque… une heure, ouais un peu plus peut-être. Pis après ben j'aimerais quand même aller faire quelques petites commissions avant qu'il fasse trop chaud. Bon maintenant il fait froid. Alors euh… D'ici que je sois prête, c'est long hein, ben c'est… Tout ça. Et pis voilà, pis une fois que je suis prête, je pars. Mais la dernière fois, j'ai pas pu sortir. J'ai pas pu, j'avais pas la petite bonbonne, j'avais l'autre qui marchait pas. Ils sont revenus le lendemain je crois, je m'en rappelle plus… Je sais plus si c'est le lendemain qu'ils ont…

**CV : [Un manque de**

C : De changer la grande bonbonne]. Je sais pas ça manque de personnel ou…. Ou un manque d'argent pour changer les trucs, j'en sais rien. Y a peut-être de tout je pense…

**CV : Alors mmh lors de ces visites des infirmières, vous avez reçu des informations justement par rapport à l'oxygène, par rapport à… vous aviez l'impression d'avoir assez de… d'informations ?**

C : Bon les infirmières, je trouve qu'à l'hôpital elles sont… elles sont pas tellement euh… formées pour ç' pour l'oxygène, pour ce que j'ai moi par exemple.

**CV : Et pis celles qui sont venues à domicile justement une fois par mois et qui ont fait ces évaluations ?**

C : Ah non ça elles ont pas regardés ça… Y en a une qui était cheffe, qui était bien, qui m'avait expliqué. Une dame un petit peu plus âgée, je sais plus comment elle s'appelle. Celle là elle savait très bien son truc. Mais les autres elles sont… C'est à dire que j'avais pas demandé tout ça avant. Mais cette dame qui, qui avait l'air de bien connaître quand même…

**CV : Elle a pu vous…**

C : Ben elle était plus là maintenant, elle est partie maintenant.

**CV : Mais elle a pu vous aider…**

C : Elle a, bon quand elle me demandait quelque chose, elle savait de quoi elle parlait disons. Pis ça c'est bien d'avoir des gens comme ça. Vous savez il y a les petites jeunes « Ouais ouais, on sait tout, on sait très bien faire et hop et hop et on fait ça » pis après là *(rires)*.

**CV : Le fait d'avoir des gens expérimentés c'était, c'était bien.**

C : Oui moi je trouve c'est très important. Alors voilà pour ce… Non je trouve que, je pense qu'à l'hôpital ça va venir de plus en plus parce que, comme à X *(aide et soins à domicile)*, vous connaissez X *(aide et soins à domicile)* ?

**CV : Oui.**

C : Je leur avais dit qu'il fallait absolument des saturomètres. Elles viennent là, elles ont pas des saturomètres, c'est important pour nous. Et maintenant ils en ont depuis que j'ai dit ça. J'ai râlé et pis… Mais oui, écoute c'est la moindre des choses. Elles vont voir des patients qui ont ça, c'est important. A l'hôpital aussi j'avais râlé… Bon à l'hôpital ils sont quand même plus… Prévu pour ce

genre de choses, mais… Mais là pas. Et pis maintenant les infirmières par là elles ont les saturomètres.

**CV : Pis celles qui étaient venues aussi une fois par mois, elles avaient les… elles avaient ce qu'il fallait, les saturomètres ?**

C : Oui je crois qu'elles avaient… Il me semble ouais… Ouais, ouais elles avaient leurs trucs…

**CV : Et comment ces visites justement des infirmières une fois par mois et les informations que vous avez reçues ont pu vous aider ou pas à gérer les symptômes de la BPCO ? …**

C : Ca m'a rien servi je trouve… Je sais pas elles me posaient des questions, fallait remplir des questionnaires, c'est tout ce que j', remplir des trucs ça me servait à rien... Enfin pour elles peut-être. Je devais répondre quelque chose et puis, puis des fois dans ces trucs c'est un peu… Vous s'av'… Je sais pas, c'est pas ça quoi… Ah je sais pas ! C'est dommage j'ai jamais gardé ces trucs, mais…

**CV : C'est pas grave. [C'était plus**

C : Vous devriez trouver].

**CV : C'était plus par rapport à… voilà [qu'est-ce que**

C : Non « Que] pensez-vous de mettre un chiffre entre 1 et 10», mais qu'est-ce que vous pensez qu'à 10 ou 9... Hein c'est comme à l'hôpital, faut mettre ces chiffres tout le temps… Des fois je mettais n'importe quoi *(rires).*

**CV :** ***(rires).*** **Mais par rapport aux symptômes du coup c'était pas, ça vous a pas…**

C : Qu'est-ce qu'ils posaient  [comme questions ?

**CV : Vraiment aidé**]. **Voilà. Ou quand elles sont venues justement, ça vous a, vous avez pas l'impression que ça vous a aidé à g' à mieux gérer les symptômes de votre maladie ?**

C : Non. Peut-être si j'avais insisté, je sais pas pas… Des fois c'est elles qui devraient poser les questions adéquates parce que… On sait pas trop pourquoi elles viennent, est-ce que… Je sais pas…  Ou c'est peut-être moi qui… Qui savait pas bien gérer, j'en sais rien.

**CV : C'était… C'était pas très clair pourquoi elles étaient là.**

C : Oui je savais pas. Elles venaient une fois tous les… tous trimestres qui viennent?

**CV : Tous les mois.**

C : Tous les mois ou tous les… ? Il y en avait trois pour.

**CV : Pendant un an, une fois par mois. [A peu près.**

C : Ouais]… Mais je sais pas à quoi ça servait. J'ai jamais compris à quoi ça servait ce truc…. Vous savez ? Vous avez pas le résultat ? Rien du tout ? Ca servait à rien. Ils dépensent du, de l'argent à l'hôpital pour rien là… *(rires)*. Si ça sert à rien. *(rires).*

**CV : Alors là c'est.**

C : Vous dites rien. Non mais ça peut servir pour des personnes, peut-être sûrement. Mais pour moi… pfff… Moi ce qu'il faudrait c'est qu'ils me disent ce qu'on pourrait faire ! *(brandit les mains)* Faire pour améliorer ce qu'on, notre état et tout. C'est tout. Pas besoin de 36'000 combines…

**CV : Plus quelque chose de.**

C : Oui de précis. Clair et net… Mais…

**CV : Quelque chose de concret.**

C : Ouais. Enfin je suis compliquée mais… *(rires).*

**CV : *(rires).***

C : *(rires)* Je suis compliquée. Ahhh.

**CV : Plus de solutions.**

C : Oui… Je sais pas comment vous dire ça. Mais c'est bête que vous avez pas ce truc…

**CV : Et plus de…**

C : Mais elles avaient toutes des questions préparées, comme ça *(regarde le guide d'entretien de l'interviewer)*, des feuilles… Vous avez pas vu ça ces feuilles, ces questionnaires. [Non.

**CV : Non], je n'ai [pas accès à ça justement.**

C : Ah vous êtes plus haut vous].

**CV : Mais c'est plus pour voir, c'est plus pour voir qu'est-ce que, comment, quel, comment vous vous souvenez que ça s'est passé et pis qu'est-ce qu' comment vous avez apprécié ça justement.**

C : Au point de vue gentillesse, j'appréciais, elles étaient toujours bienvenues. Des fois même on riait, elles me posaient des questions, je les charriaient aussi. Je les aimais bien. Ca faisait de la visite quoi *(rires).* C'était peut-être pas ça qu'elles voulaient, je sais pas. Sûrement pas.

**CV : Elles vous ont… Elles ont pu vous amener, vous apporter du soutien ?**

C : Oh si j'avais demandé sûrement. Mais j'ai eu tellement de trucs, même pendant ces années. Je suis retombée deux fois à l'hôpital hein… Alors euh… C'est pour ça que je suis un peu *(rires).* En bas de…. Je suis un peu… Mais comment c'qu'on dit ?... Un peu déçue, disons pas déçue, de… qui y ait pas plus de… d'apport à ça.

**CV : Vous aimeriez plus d'apports. De quel type ? Vous sauriez dire ?**

C : Je sais pas, qu'on nous dise comment procéder pour faire un peu mieux les choses et tout. Bon ils nous diront « Ben allez dans une société, allez vous inscrire je sais pas où » … faire de la gym ou je sais pas quoi. Mais ils se rendent pas compte que quand vous portez une bonbonne pis qu'il faut marcher, aller jusque je sais pas où en tram, c'est pas de la tarte *(rires).* C'est ça qu'ils comprennent pas… Quand on conduit encore ça va hein c'est rien, mais… Mais ils disent d'aller faire de la gymnastique à l'hôpital ou près de l'hôpital. Vous faites comment ? … « Non c'est rien d'aller là-bas ». Ah ouais c'est rien *(ton ironique)*. Il faut aller dans les bus pleins euh… Pis quand vous demandez les services de, ceux qui peuvent un peu aider, les taxis, c'était pas vu, pas bien, j'avais pas le droit, pourquoi ? Il y avait toujours des t' des trucs négatifs… Que j'avais droit qu'aux visites médicales mais pas à la physio. Des trucs comme ça, aberrant. Le docteur m'avait alors répondu « Alors vous aimez mieux qu'elle tombe dans le tram, qu'elle se casse la figure, ça rapportera xxx plus »…

**CV : Vous auriez aimé de la physio ou... [autre chose ?**

C : Non j'en ai de la physio], mais faut toujours demander la charité. Non les physios ils sont, ils sont bien, c'est pas… J'y vais dans un petit truc au X *(quartier)* qui est, il est très bien le jeune homme... Mais avant j'all' c'était le docteur qui connaissait toute une bande qui, c'était bien du reste… à … X *(rue)*, non c'était où ? X *(quartier)*, par là. C'est une dame qui s'en occupait pis y avait… on était plusieurs là. C'est vrai qu'elle faisait très bien la… la physio, les… les marches, les machins euh. Alors euh voilà mais…

**CV : Vous aimeriez plus donc de.**

C : J'aurais continué chez elle si j'avais, si j'avais pu me débrouiller disons. Mais… là c'est un peu angoissant d'y aller… Voyez quand vous devez y aller pour deux heures ou je sais pas quoi, d'ici que vous prenez le bus, arrivez à … disons que le X *(numéro de bus)* il est déjà bourré depuis là… le bus… Alors voilà je veux pas vous expliquer, ce serait trop long. *(rires).*

**CV : C'est angoissant de… voilà de, de devoir prendre les transports [et les offres.**

C : Oui exactement].

**CV : Qu'on vous propose sont peut-être pas adaptées.**

C : C'est à dire oui ils prennent… pas en compte que on a pas toujours les moyens de payer chaque fois des taxis. Pour finir je prenais des taxis, je crois que ça me revenait moins cher que de prendre… euh… un qui conduisait pour les, pour les aides là, comment… Je crois que je payais pas plus cher pour finir. Pis comme ils nous remboursaient pas à l'assurance, alors…

**CV : C'était com' compliqué.**

C : Alors j'aimais bien payer moi-même, terminé. Mais on peut pas faire sans arrêt ça hein ? Ca va vite *(rires)*.

**CV : *(rires).***

C : Enfin, je vous radote des choses qui vous intéressent pas du tout.

**CV : Non, non.**

C : *(rires).*

**CV : Et mmh vous m'avez parlé un petit peu que vous aviez du soutien de la part de vos proches, quel soutien vous avez reçu justement [de la part de vos proches ?**

C : Et ben ma sœur] m'a beaucoup aidé pour tout hein. Quand je suis arr' quand je suis tombée là et pis heureusement qu'il y avait celui de X *(entreprise qui distribue des gaz médicaux)*. Il est arrivé, j'étais raide parterre. Heureusement qu'il avait les clés pour ouvrir, ils savaient pas que j'… Vous savez ils m'ont embarqué en ambulance… Euh oui ben ma sœur bon elle passe souvent vers moi quand même… C'est elle qui, qui m'aide à faire les commissions et tout… Bon maintenant j'y vais un peu toute seule, je prends le bus jusqu'à la X *(magasin d'alimentation)* et pis… ils me livrent, je demande de me livrer… Mais bon ça fatigue vite hein d'aller, de sortir… Mais je fais livrer alors ça va…

**CV : C'es'.**

C : Non c'est bien. Ils sont gentils pour ça la X *(magasin d'alimentation)*. Je paie sur place pis ils me livrent à telle heure et puis voilà ils viennent… Non pour ça c'est bien, comme ça je peux faire mon repas et tout. Je pouvais plus supporter ces repas de X *(aide et soins à domicile)*,,. Oh quelle horreur.

**CV : Donc vous avez votre sœur qui est là pour.**

C : Non elle elle l'a jamais fait elle, c'est moi qui' après c'est moi qui m'oc' au début j'ai fait X *(aide et soins à domicile)*, pour pas l'encombrer avec ça. Et moins porter pendant un certain temps. Et après j'ai fait moi-même… Non mais je peux pas toujours lui demander à faire ci à faire ça hein. Elle en aura marre aussi. *(rires).*

**CV : Vous disiez que vous aviez un peu peur d'être euh de trop demander.**

C : C'est ça parce que… on a toujours l'air de demander quelque chose. Oh j'essaie de pas trop demander… Pis ici les gens bon la dame en face m' dit « Si vous avez besoin de quelque chose,

vous me dites » pour les commissions. Je lui ai presque demandé aujourd'hui, voilà qu'elle partait jusqu'à dimanche.

**CV : Votre voisine ?**

C : La maîtresse d'école, celle qui est en face. Mais c'est toujours comme ça. Vous demandez et c'est juste quand il faut pas *(rires).*

**CV : *(rires).***

C : Non mais elle était gentille. Une fois elle m'a fait, pris des courses, des trucs lourds…

**CV : Donc il y a votre voisine qui est là aussi.**

C : Ouais mais bon elle est pas toujours là vous voyez. S'il faut que je redonne une clé à quelqu'un, faut vraiment quelqu'un de confiance. Mais elle j'aurais confiance. Parce que y avait une dame qui s'occupait de moi xxx mais elle m'a rendu, elle voulait plus s'en occuper parce que je crois que c'est trop de soucis. Elle était bien pourtant, elle m'a bien aidé. Je sais pas je lui avais jamais rien demandé à cette femme. Mais elle s'est bien occupé au début pis je pense, maintenant ça lui, vous comprenez quand on s'occupe de quelqu'un faut, faut être tout le temps là dans le fond. S'il arrive quelqu' quelque chose, qu'il faut ouvrir la porte, il faut être là. Voyez…

**CV : Ca demande une présence.**

C : Oh ouais. Faudrait, pis on ose pas y donner tout au concierge les clés. Je sais pas.

**CV : Oui ça demande d'avoir quelqu'un de proche et quelqu'un de de confiance oui.**

C : Oui c'est ça. Et pis là y a pas grand chose. Y a une dame qui est âgée et qui vient de là, mais elle est tellement âgée. Pis en face une jeune femme avec deux petits gamins, mais elle est pas souvent là. Bon y a la maîtresse d'école, mais… Suffise qu'elle est à l'école et tout que ça se passe quand elle est pas là. Elle peut pas quand même sortir de l'école.

**CV : Et à côté de votre sœur, il y a avait aussi d'autres personnes de votre famille [ou des proches.**

C : Ouais bien sûr] mais… Ils vont quand même pas venir de tous les coins du, de la ville… Ce qui m'énerve c'est déranger tout le temps quelqu'un. C'est ça…

**CV : Avoir l'impression de [déranger.**

C : D'être] une encouble... Les soirs, on se fait des… des scrupules… Il y en a qui s'en font pas, mais quand même. C'est pas rien…

**CV : Le sentiment que c'est… c'est difficile de demander aussi de l'aide.**

C : Oui de demander oui…

**CV : C'est pas… pas toujours évident.**

C : Non c'est pas évident de demander *(rires).*

**CV : *(rires).***

C : Moi j'ai beaucoup de plaisir avec votre docteur que vous connaissez pas là…

**CV : Avec.**

C : Le docteur D. *(pneumologie)*. C'est quelqu'un de vraiment bien.

**CV : Là vous aviez eu du soutien.**

C : Oh il était gentil… Je lui avais demandé qu'il m'écrive une lettre pour… pour ici. Ils faisaient tellement de poussière et tout. Ils en ont fait de la poussière. J'en ai pas mal avalé.
**CV : Avec les travaux ?**

C : Oui. Oh oui… Mais les m' mais il m'avait fait une lettre comme quoi je devais changer d'appartement entre temps. Et bien sûr j'ai changé, mais c'est de nouveau en pleins travaux *(rires).*

**CV : *(rires)* oui il y a des événements *(rires).***

C : Non y a…

**CV : Est-ce que vous arrivez à penser à quelque chose, de quoi vous auriez eu besoin en plus, par exemple quand ces infirmières sont venues à domicile, qu'est-ce qu'elles auraient pu vous offrir de plus ?**

C : Ah les vôtres ?

**CV : Oui. Celles qui sont venues une fois par mois.**

C : Ah. Je sais pas, vous êtes pas de la même sorte alors *(rires).*

**CV : *(rires).***

C : J'… Peut-être de demander si on avait besoin de quelque chose de… comment on dit ? *(brandit les mains)*...

**CV : Concret ?**

C : De concret peut-être. « Vous avez besoin de quelque chose, je sais pas, qu'on vous apporte, pas à manger, mais… ». Quelque chose qui vous distrait ou bien des, je sais pas, des petites choses. J'aurais dit non, notez bien, mais… 'fin je sais pas, c'est des petites choses. Des fois je pense comme ça, mais… Ca m'occupe pas plus que ça…

**CV : Plus quelque chose qui vous distrait.**

C : Ouais… Quoi mais j'aimerais m'acheter un X *(smartphone)*, mais j'aimerais qu'on me montre comme il faut parce que j'ai de la peine, bon… j'ai celui-là *(montre son téléphone portable)* mais il est trop vieux. Il faudrait que quelqu'un m'apprenne comme il faut sans m'én' sans s'énerver. Parce que ma sœur me dit « T'as pas besoin d'apprendre ça, t'y comprendras rien » *(rires).* Alors rien que ça, ça, ça me coupe les moyens et pis je peux pas. Mais il me faut vraiment quelqu'un de très clame, qui, prenne un certain temps quoi. Je sais bien que je suis dure à comprendre, mais bon j'ai bien appris la première tous ces trucs avant.

**CV : Donc plus quelque chose, des solutions pratiques.**

C : Ouais pratiques, ouais.

**CV : [Comme.**

C : Pis] c'est vrai que c'est important ces X *(smartphone)*, c'est X *(smartphone)*, je sais pas comment on dit. Pis ça peut rendre service pour un tas de choses quand même.

**CV : Donc plus des coups de mains un peu pratique.**

C : Ouais exactement. Qu'on puisse découvrir un tas de choses quand même. Parce que ça occupe l'esprit, ça je trouve c'est bien. Avant j'aimais pas, mais je trouve que bon. Je regarde la télévision beaucoup, mais… Mais avec ça je trouve que… on veut apprendre quelque chose, voilà tout de suite

la solution quoi, c'est comme le, l'internet quoi. Parce que j'ai internet mais ça fonctionne plus je crois *(rires).*

**CV : *(rires*). Comme vous disiez, peut-être quelque chose qui pourrait vous distraire.**

C : Ouais... Ouais y a bien des bénévoles à X *(ville)* là. J'ai demandé un ou deux pour réparer une fois le téléphone. Mais ils savent juste le… Disons qu'ils font le bien leur travail, mais c'est pas des experts... Non mais c'est c'est gentil de venir bénévolement déjà. Mais si y en avait des… Ils peuvent pas parce qu'il faudrait les payer trop cher… Qu'est-ce que vous voulez, on peut pas tout avoir. Et il y en a qui se débrouillent très bien tout seul hein c'est. Mais moi je suis pas une débrouille alors *(rires).*

**CV : Donc quelqu'un qui est là pour donner des petits coups de mains.**

C : Oui c'est ça… Ben il me faut pas une bonne femme vieille, il faut une jeune normalement constituée pis qui soit calme surtout ouh, qui s'énerve pas *(rires)*.

**CV : *(rires)*… Et peut-être mmmh lors des visites des infirmières, qu'est-ce que vous pouvez me dire sur le soutien au niveau spirituel qui a été apporté ?**

C : Bon ben pas du tout.

**CV : Pas du tout.**

C : Non... Non ça chacun demande ce qu'il veut hein. Je suis sûre si j'avais demandé quelque chose, elles auraient fait le nécessaire…

**CV : Mais…**

C : Non là bon je suis encore xxx ça va… Mais elles qu'est-ce que vous voulez… Je sais pas si elles f', si elles font ça, dans ce sens là. Elles doivent bien essayer comme y a des gens quand même… qui pensent qu'il faudrait partir une fois quoi… Mais je sais pas.

**CV : Mais y a pas eu en tout cas de voilà.**

C : Non je pense qu'elles m'ont laissé faire… Si j'avais voulu, j'aurais demandé, parce que j'ai été à l'hôpital quand même plusieurs fois… Et pis à l'hôpital ils me demandaient si j'avais besoin de quelqu'un. J'avais eu un prêtre. Très gentil du reste…

**CV : Mais pas… Pas dans c' dans ce cadre là en tout cas, des infirmières, là il y a pas eu.**

C : Je crois pas qu'elles m'aient demandés quelque chose. Ou alors j'ai oublié. Je sais pas… Mais pour elles c'est délicat aussi de demander, je comprends très bien… *(rires).*

**CV : Est-ce qu'il y aurait d'autres choses justement que [vous imaginiez.**

C : Vous avez une belle montre]. C'est une X *(marque de montre)* ?

**CV : Non c'est une X *(autre marque de montre)*. C'est une petite marque.**

C : C'est français ?

**CV : Oui je crois que c'est français.**

C : Ils nous copient tous maintenant.

**CV : J'aime bien quand on voit bien l'heure.**

C : Oui on voit bien. Pis elle est plate, elle est jolie.

**CV : On voit bien.**

C : Les français ils commencent à faire de belles montrent maintenant. Ils ont tout copié sur la Suisse.

**CV : Elle est simple. C'est pas une Suisse mais *(rires).***

C : Non. Elle est pas française ?

**CV : Je crois qu'elle est française, oui.**

C : Ah ouais c'est ce que je dis. Ils ont commencé à, à copier la Suisse.

**CV : Comme ça au moins je vois bien, je vois bien l'heure.**

C : C'est vrai qu'elle elle est très jolie comme ça…

**CV : Est-ce qu'il y aurait d'autres choses que vous aimeriez rajouter ou quelque chose un peu dans vos attentes, vos besoins ? De quoi vous auriez eu besoin ? Ou [vous auriez besoin.**

C : Du chocolat] *(rires).*

**CV : Du chocolat *(rires).***

C : Vous voulez boire de l'eau ?

**CV : Non ça va, c'est gentil, merci.**

C : Bon… Je sais pas, faudrait… C'est des choses faudrait que… Je me, je m'habitue au cerveau, pas laisser tomber ce cerveau. Non mais avec la physio tout ça, ça me fait déjà pas mal. La physio, les docteurs et tout… Rien que les déplacements ça me tue. *(rires).*

**CV : Ca fait déjà pas mal.**

C : Ca fait oui… ça fatigue vite. Pis les commissions, encore le pire… Pis je vais au petit marché là, au X *(ville)*…. Ils ont fait un petit marché de viande, c'est bien.

**CV : Donc rien d'autre de particulier, voilà que vous aimeriez encore ajouter.**

C : Non j'en sais rien.

**CV : Ou dire sur comment ça s'est passé justement avec.**

C : Avec les anciennes filles ?

**CV : Avec ces infirmières voilà [qui.**

C : Non] elles étaient sympa comme tout… Mais… Elles ont fait leur boulot, mais je sais plus, je pourrais plus vous dire les questions qu'elles posent.

**CV : Non c'est pas grave.**

C : Si vous retrouviez leurs feuilles mais… Chaque fois je charriais et je disais « Encore ces questions ? Vous me les avez déjà posées » *(rires).*

**CV : *(rires).* C'était.**

C : Il y en avait une qui riait tout le temps, je sais plus comment elle s'appelait.

**CV : C'était beaucoup aussi les questions.**

C : Oui y avait beaucoup de questions. Mais je suis sûre que vous allez retrouver ça.

**CV : Mais oui.**

C : Si je les retrouve, je vous les envoie *(rires)*.

**CV : Non non, ils les ont. Mais l'idée c'était plus voilà sans rentrer dans le détail des questions, un peu qu'est-ce que vous en aviez un peu pensé et…**

C : Non ça m'a pas frappé vraiment.

**CV : Non.**

C : Je sais pas c'était utile pour les autres. J'en sais rien.

**CV : Mais en tout cas pour vous l'utilité de ces visites.**

C : Pour ce genre de questions, je trouvais… Je sais pas au début, ceux qui sont seuls c'est possible…. Qu'ils aient… besoin un peu de ça, j'en sais rien. Voir l'esprit des gens. Y en a qui sont peut-être plus intelligents que moi *(rires)*, ils veulent savoir ce qu'ils font. C'est ça le problème…

**CV : Mais pour vous, vous sentiez moins seule et vous aviez moins besoin.**

C : Ah pour savoir savoir ce qu'on veut?

**CV : Non de justement vous disiez que c'était plus utile pour des personnes qui sont [seules.**

C : Ouais qui] sont vraiment seules, seules, seules et pis qu'ont besoin d'une présence, mais… Je crois pas que ça serve à une présence ces dames parce qu'elles viennent une fois pis c'est terminé. Une fois par euh je sais pas trimestre ou mois, je m'en rappelle plus…

**CV : Cela aurait été utile d'avoir quelqu'un plus présent ou.**

C : Ouais alors peut-être venir plus souvent, j'en sais rien. Parce que vraiment elles étaient sympathiques comme tout. Ben oui elles venaient quand même presque une heure de temps, pas plus hein. Est-ce que ça c'est, j'en sais rien…

**CV : Peut-être si ça avait été plus long.**

C : Je sais pas si faut payer des gens à l'hôpital pour faire ça… Faut pas que je dise ça parce que *(elle regarde l'enregistreur) (rires).*

***CV : (rires).* Non non, vous avez le droit de tout dire *(rires).***

C : Ca fait des frais.

**CV : Vous avez le droit de tout dire.**

C : Mais si ça valait la peine, je sais pas. Ca c'est à demander aux autres… Je sais pas [quand.

**CV : Vous] vous ne voyez pas trop l'utilité [de.**

C : Non] non *(rires).*

**CV : Est-ce qu'il y a autre chose que vous aimeriez encore dire ou quelque chose par rapport à ces visites qui vous vient à l'esprit ? Ou… Ou vous m'avez dit un peu tout ce que**.

C : *(le téléphone sonne, la patiente répond et revient s'asseoir).* Alors qu'est-ce que vous vouliez d' ?

**CV : Est-ce qu'il y a autre chose encore que vous aimeriez ajouter.**

C : Je m'en rappelle plus.

**CV : Par rapport à cette prise en charge avec ces infirmières pendant un an. Quelque chose d'autre [que.**

C : Parce] que là elles ont fini maintenant? Elles vont revenir ?

**CV : Là c'est juste, non c'était, elles sont venues pendant, celles de la recherche en tout cas, elles sont venues pendant.**

C : C'est fini.

**CV : Oui voilà c'est fini. Elles sont venues il y a un an, quelque chose comme ça.**

C : Oui parce que j'en ai vu une quand j'étais à X *(nom d'hôpital)* là. Une ancienne qui était venue ici. Je sais plus comment elle s'appelle, très sympa, une infirmière.

**CV : J'ai les noms.**

C : Je sais plus le nom. Vous avez pas le nom ?

**CV : C'est *Madame Laure Chappuis (infirmière de recherche), Julie Mottier (infirmière de recherche)* ou *Claire Favre (infirmière de recherche)*.**

C : *Claire (infirmière de recherche)* je l'ai vu souvent, on riait tout le temps.

**CV : C'est les trois infirmières qui sont venues.**

C : Pis une qui est à X *(nom d'hôpital)* après, je l'ai vu il y a pas si longtemps. Je sais plus comment elle s'appelait, le nom.

**CV : En tout cas j'ai ces trois noms là.**

C : Non elles étaient les trois sympathiques.

**CV : Donc voilà si je comprends bien, vous avez trouvé ça... 'fin que c'était des moments plutôt agréables, sympathiques.**

C : Ah oui elles étaient très agréables ces filles.

**CV : Mais après en termes d'utilité, c'était un petit peu plus difficile à [à voir.**

C : Je pensais] qu'il y aurait eu d'autres trucs qu'elles auraient pu faire peut-être, je sais pas… Ou alors prendre plus souvent la… la saturation. Il y en a qu'une qui faisait ça, qui était cheffe et qui était pas dans votre liste. Plus âgée… Celle-là elle savait bien faire le… le truc, le… l'oxygène. Elle connaissait bien. Et pis la saturation, elle savait, tout de suite elle prenait… Ca c'est important…

**CV : Vous arrivez à imaginer les autres choses qu'elles auraient pu faire qui, que vous auriez apprécié ?**

C : Ca, mais je sais pas si les autres elles faisaient pas, à part la la cheffe là.

**CV : La saturation oui.**

C : La saturation. Je crois pas qu'elles faisaient les autres… Je pense ils peuvent pas donner à tout le monde. Ca coute cher ces trucs… Pis en acheter un je sais pas si ça vaut la peine parce qu'on est toujours en train de regarder. Hein…

**CV : Mais en tout cas là vous auriez peut-être apprécié qu'il y ait un petit peu plus ça, plus souvent la saturation qui soit [vérifiée.**

C : Peut-être c'est] b' ouais. A des moments peut-être. Bon maintenant à X *(aide et soins à domicile)*, ils ont en ont maintenant. Depuis que j'ai râlé. *(rires).*

**CV : *(rires).***

C : *(rires).* C'est grâce à moi qu'ils en ont.

**CV : Donc voilà, à part la saturation un peu plus, vous ne voyez pas trop.**

C : Non euh la tension ils prennent à chaque fois qu'ils viennent les infirmières. Avant elles venaient… deux fois par semaine. Maintenant elles viennent plus qu'une fois par semaine. J'ai déjà tous les médicaments que j'ai déjà préparés du reste… J'en avale de la saleté. *(rires).*

**CV : Ca fait pas mal… Donc à part un p' vérifier plus la saturation, il y avait d'autres choses que vous auriez voulu qu'elles fassent ?**

C : Non même pas, mais… Des fois on aurait besoin d'elles en tant que soutien pour demander peut-être quelque chose, mais maintenant je vois plus ce que… je pourrais demander… Par exemple, quand il m'est arrivé mon pépin pour la…

**CV : Avec l'oxygène.**

C : Pour l'oxygène, j'aurais voulu que quelqu'un soit là, que je pose la question, pour que je puisse poser la question « pourquoi ? ». Comme maintenant, j'ai l'oxygène, on appuie sur des trucs pis y a tant de points qui s'allument encore, si c'est plein, et maintenant j'ai vu qu'il y a plus qu'un qui est allumé, c'est… Demain ils viennent, heureusement mais ! Est-ce que je peux aller jusqu'à demain ? J'en sais rien. Je vais essayer quand même de téléphoner… C'est tout des petits trucs qui énervent, voyez. Qu'on aimerait s'en passer tout ça…

**CV : Plus des choses pratiques.**

C : Ouais, ouais. Faudrait qu'on sache tout, mais comment veux-tu. Pis on se fait encore engueuler si on demande *(rires).*

**CV : *(rires).***

C : *(rires)* Oh lala.

**CV : Donc voilà, de manière générale c'était quelque chose d'assez agréable ces visites. Mais après en termes d'utilité, vous attendiez peut-être [autre chose.**

C : Qu'elles] viennent une fois d'accord, mais pas tout le temps… Je sais pas, il faudrait demander aux autres ce qu'elles en pensent, c'est ça. C'est possible qu'il y en a qui aiment bien hein. J'aimais bien les recevoir, ça d'accord. Mais je vois pas l'utilité, à quoi ça sert… *(rires).*

**CV : *(rires).***

C : *(rires).* Ouais, ouais.

**CV : Donc pas trop d'utilité, mais en tout cas c'était quelque chose de voilà agréable.**

C : Oui oui très... Je sais pas. Vous les saluerez bien si vous les voyez.

**CV : Oui *(rires)* ça marche.**

C : Elles viennent plus alors elles ?

**CV : Euh non, non non. Je crois pas.**

C : Elles ont fini leur stage.

**CV : Merci en tout cas d'avoir, est-ce qu'il y a d'autres choses que vous aimeriez rajouter ?**

C : Non, je crois pas.

**CV : Vous avez dit un peu tout... [tout ce que vous pensez en termes de.**

C : Je crois pas], je vais téléphoner pour ce… cet oxygène là.

**CV : Oui.**

C : Vous voulez que je vous montre comment c'est ?

**CV : Oui vous pouvez me montrer.**

C : *(la patiente se lève et montre à l'interviewer comment marche son appareil à oxygène qui est situé dans une autre pièce).*

## Participant n°2

Nom d'emprunt : Vivianne
Âge : 74 ans
Sexe : F
Groupe : Contrôle

**C : Donc si je reprends, c'était y a environ deux ans maintenant, je pense que ça s'est terminé l'étude. Vous avez participé à une étude qui avait pour objectif de comparer le traitement habituel de votre maladie pulmonaire, la BPCO, avec une prise en charge précoce, soutenue et intégrée. Et vous avez été dans le groupe bénéficiant des soins habituels. Pouvez-vous me dire comment ça s'est passé ?**

**V :** Oh, très bien !

**C : Très bien.**

**V :** Très bien, oui *(rires)*.

**C :** ***(rires)*****.**

**V :** C'était pas du tout contraignant. Et puis, y avait pas, j'avais pas beaucoup de rendez-vous, en fait. Donc c'était assez fluide, ça s'est, c'était, non, bien.

**C : Ça s'est, ça s'est bien passé ?**

**V :** Ouais, ouais, bien passé, oui *(rires)*.

**C :** ***(rires)*****. Donc pas trop contraignant ?**

**V :** Non, mais bon, c'est toujours les, toujours ces questionnaires. Les questionnaires, des fois, je trouve qu'ils sont, même pour la physio, les trucs comme ça, y a toujours un, on est entre-deux, souvent. C'est pas, c'est pas très, très clair. 'Fin, c'est pour moi, mais, j'entends, j'en n'ai pas, on s'est expliqué, pis c'était, c'était ok. Mais je me souviens plus, j'ai plus de, de trucs précis à, la question précise, à mentionner, parce c'est, ça date quand même, pis j'ai une mémoire qui, qui est des fois un peu fatiguée *(rires).*

**C : Ouais *(rires)*. Et parfois c'est un peu difficile, entre-deux, pour la réponse ?**

**V :** Oui, voilà. Mais bon, c'est, c'est, mais c'était pas, c'était pas dramatique, ça va.

**C : Oui. Donc ça va, c'était pas trop, pas trop contraignant ?**

**V :** Non, non, non, pis c'était pas trop long, non c'était assez bien mené, je dois dire quand même, ouais.

**C : D'accord, d'accord. Et peut-être, qu'est-ce que vous attendiez en participant à, à cette étude ?**

**V :** Ben, rien de spécial, c'était le fait de, de faire avancer tout, quoi, de participer à quelque chose qui avance. C'est toujours intéressant les, les études, mais bon, comme je vous ai dit tout à l'heure, le, tant qu'on n'a pas les résultats, c'est difficile de savoir à quel point ça a été bénéfique, ou pas. Voilà *(rires)*.

**C : Ça, ça on, on verra *(rires)*.**

**V :** Ouais *(rires)*. On verra. Mais on aura les résultats ? On va nous, non ben.

**C : Alors vous pourrez les, les demander, là ils sont en train d'être analysés, et puis vous pourrez les demander. Donc là, je peux pas vous en dire en plus à ce stade-là, *(rires)* ça prend toujours un petit peu de temps, mais, mais voilà. Donc, donc pas trop d'attentes, spécifiques [par rapport à la participation ?**

**V :** Oh non, j'étais] juste le, le, le, de, de faire, comment dire, avancer le schmilblick, un peu, voilà. Parce que c'est toujours bien, c'est bien que des gens se penchent sur, sur ces problèmes et fassent des études. Donc pour faire des études, il faut qu'y ait des gens qui répondent, voilà. Alors c'est, c'est, c'est dans ce but-là en fait, que j'ai participé.

**C : Faire, faire avancer les choses.**

**V :** Voilà.

**C : Parce que vous aviez été, voilà, dans le, dans le groupe où y avait pas eu de soins spécifiques en plus. Donc vous auriez pu aussi, à ce moment-là, dire que vous ne vouliez plus participer. Mais voilà, pourquoi vous avez quand même accepté de participer ?**

**V :** Ben, je viens de vous dire, c'était pour que [ça fasse avancer.

**C : Pour faire avancer].**

V : Ouais. Je trouve que c'est, c'est bien, le, le, l'idée de faire des études. 'Fin, parce que c'est comme ça qu'on avance. Et puis, bon y a, c'est comme on voit maintenant, moi j'ai une petite-fille, je vois, ma fille va avoir 40 ans, ma petite-fille en a eu 5, et je me dis, mais, je vois comme on les élève, et les, les médicaments qu'on leur donne et les trucs comme ça, c'est complètement, c'est complètement l'inverse de ce que j'avais fait quand elle était petite. Mais bon, c'est, c'est dû aussi à des études qui ont été faites, c'est, c'est tout un processus comme ça.

**C : Ouais. Ça, ça évolue *(rires)* ?**

**V :** Ça évolue, voilà, ça évolue. Des fois, c'est pas, c'est pas toujours en bien, mais. Pis bon, aussi le, le, j'ai jamais vu, j'ai jamais vu autant d'enfants qui ont recours à du X *(médicament bronchodilatateur)* maintenant. C'est quelque chose que je connaissais pas à l'époque. Peut-être que moi j'en avais besoin, sans le savoir aussi. Parce que ma petite-fille, elle a les bronches, très délicates, alors elle a, des fois le, la, l'oxygène elle est, l'été, l'hiver dernier, elle est allée trois ou quatre fois aux urgences, elle avait la lunette avec l'oxygène. Elle disait : « je suis comme ma grand-mère, mais elle, elle a un cancer des poumons ». ; xxx « ben heureusement que t'as pas ça ». *(rires)*.

**C : Ouais *(rires)*.**

**V :** Mais bon, c'est tout abstrait, à cet âge-là, évidemment.

**C : Ouais ça, ça change *(rires)*.**

**V :** Oui.

**C : Et, peut-être quelle a été votre réaction en apprenant que vous faisiez partie du groupe qui bénéficiait des soins habituels, quand on vous a dit ?**

**V :** Ben c'était, comme c'était un, c'est, c'était un tirage au sort. Alors c'est, c'est, ça m'a, ça m'a pas… Non, ça m'a pas gênée du tout. Parce que faut jouer le jeu, ou bien pas, ou pas s'emmêler, quoi, c'est tout. Alors ça m'a pas du tout, ennuyée ou perturbée. C'était juste, c'est comme ça, bon je fais partie de l'autre, voilà. Pis comme j'avais aussi fait, une fois partie d'un truc contre l'ostéoporose, et je, j'avais, moi j'avais le placebo. Mais euh, après on m'avait dit à la fin : « toute façon c'est un, un, c'est pour l'étude d'un nouveau médicament, qui ne sera pas remboursé par les assurances maladies ». Pis je m'étais dit : « mais j'arrête alors, ça sert à quoi ! » Pis après j'ai dit : « non, c'est quand même, c'est quand même utile ». *(rires)*. Mais voilà.

**C : Ouais. Donc quand même une certaine utilité à participer à, à cette étude ?**

**V :** Voilà. Ouais.

**C : Ouais. Et, si l'on regarde, ben les soins que, que vous avez reçus, quelle est votre appréciation générale des soins reçus dans le cadre de votre maladie pulmonaire ?**

**V :** Ben je, j'entends que c'est, c'est, ils sont, ils sont utiles, parce que si j'avais pas l'oxygène, je serais paumée, perdue. Pis le, bon le masque pour les apnées, c'est bien, parce que je dors mieux. Bon, c'est, le masque j'en ai, j'en suis à mon six ou septième. Je trouve ça devient un peu cher, parce qu'on nous en rembourse que deux par année. On a tendance à vous les changer un peu trop, maintenant je râle quand c'est comme ça, *(rires)* mais. Bon, j'ai les derniers, on a mis de nouveau deux nouveaux à l'hôpital, et pis y en a qu'un qui m'est remboursé. Alors, à coup de 250 balles, je trouve que ça devient un peu cher. Parce que c'est insensé ce que la, ce que la médecine est chère en Suisse. Bon je, bon je suis Suisse aussi, mais bon. Je, je trouve que c'est, c'est aberrant, quoi. Pis bon, les assurances, moi j'ai aussi reçu l'augmentation de primes. On nous danse sur le ventre allègrement, je trouve.

**C : Donc, vous êtes plutôt satisfaite des soins, mais c'est quand même assez cher ?**

**V :** Ouais, ouais, c'est, bon le, ils, c'est cher d'être malade hein. Ça coûte cher d'être malade *(rires)*. Mais je sais pas moi, je vois quand, avec les, l'immuno', avec, bon, déjà le, le, le, l'oncologue voulait me traiter en immunothérapie. Mais l'immunothérapie, la, la, l'assurance maladie ne rembourse pas. Il faut d'abord faire des chimios, en tout cas trois. Pis comme ça marchait bien, il en a fait six, 'fin elle marchait bien, la première non, y a des ennuis après. Mais, et pis la même chose avec l'immunothérapie, la première, ça a bien marché, pis j'ai fini à l'hôpital le soir, avec 39.4 de fièvre. Même chose avec le, le, le, le, les premiers, la, la, les, ah, les, les, les traitements qu'on nous donne, le premier traitement, ah je sais plus comment ça s'appelle, j'ai un blanc. Mais le, le, le, le médecin m'a dit, l'oncologue, il m'a dit : « mais toute façon là, pour l'immunothérapie, faut pas vous en faire, je serai votre avocat. Parce que, je, je peux pas vous soigner autrement, je peux pas opérer, je peux pas faire des, des rayons. Donc y a pas de miracles, et vous allez être remboursée, mais ça implique ». Je sais qu'ils ont, ils, ça a pris en tout cas un mois et demi, pour qu'ils aient la possibilité de le faire. Alors j'entends, si, 'fin je sais pas, *(sonnerie de téléphone)* je m'excuse.

**C : Oui, oui, répondez.**

**V :** *(conversation téléphonique).*

**C : Donc, voilà, un peu, un peu cher donc ces, voilà ces, ces traitements.**

**V :** Ouais, ouais, voilà. Et pis, avec la chimio, la chimio, quand, à chaque chimio, le lendemain, je devais faire une injection de X *(médicament)*, c'est des, c'est des globules blancs. Quand j'ai vu les factures, une, une injection c'est 1'300 francs. « Pfiou ». Bon j'ai eu, comme j'ai eu pas mal d'hôpital, j'ai eu pas mal de frais, aussi, parce que y a quand même le 10%. Et l'hôpital, c'est cher, quoi ! Mais voilà.

**C : C'est coûteux.**

**V :** C'est coûteux. Mais bon, là on m'a dit dans ma famille, toute façon on te file un coup de main si jamais, parce que c'est quand même ta santé qui compte avant tout.

**C : Oui, oui, oui, oui. Et, de quoi d'autre auriez-vous eu besoin peut-être comme soins, pour la BPCO ?**

**V :** Je vois pas, je vois pas grand-chose d'autre. Je, parce que c'est, c'est, c'est, cette, cette peine à, à respirer, à, à, bon là, y a aussi la, quand y a des particules, sortir, ou quand il fait trop chaud, je sors pas. Je, j'ai l'impression d'entrer dans une, dans une, un rôtissoire, et pis je peux plus souffler. Alors, mais sans ça, je, je continue à aller faire mes courses. Bon, je m'arrête si, si je suis essoufflée, pis, pis ça redémarre. J'entends, je, je, je, je persiste quand même. Parce que, là quand j'ai eu cette, cette nouvelle, tout le monde m'a dit : « mais on viendra te faire la cuisine ». J'ai dit : « mais laissez-moi le peu qui me reste quand même. Il faut que je puisse faire quelque chose *(rires)* ». Alors le, j'aime pas faire faire mes courses pour la cuisine par quelqu'un d'autre, parce que j'aime choisir en fonction des, de ce que ça, ce qui me fait envie. J'adore les légumes, donc j'ai pas de problèmes aussi pour ça. Mais j'aime choisir ce dont j'ai envie ! Un petit peu sale caractère, mais voilà.

**C : *(rires)*. Voilà, envie de, envie de rester indépendante.**

**V :** Oui, voilà. J'ai, je trouve que la dépendance, c'est horrible. Comme j'ai, de mettre des bas de contention au début, j'ai, y a les soins à domicile qui sont venus les mettre le matin. Pis j'ai trouvé toujours

moyen de m'aider par quelqu'un, pour les mettre, les enlever le soir, parce que c'est très difficile. Pis bon, quand j'avais, j'avais 25 kilos de plus, c'était encore plus difficile. Et puis, là, j'ai dit : « non, mais il faut que j'y arrive toute seule, c'est pas possible ! » Et puis je me suis débrouillée, et pis j'ai, j'ai, j'ai dit aux gens qui venaient, j'ai dit : « écoutez, je vais, on va arrêter, mais pendant encore trois fois, vous faites mon coach, pis vous me dites si ça va ». Pis ça a été, et pis après j'ai dit, j'ai réussi à y faire toute seule.

**C : Ouais. Vous arrivez encore à faire des choses.**

**V :** Ouais, ouais, parce que j'entends, c'est, c'est, c'est horrible d'être, moi je trouve que c'est, j'aurai tout le temps quand je serai encore beaucoup plus vieille, mais, 'fin notez que ça, c'est demain, quoi, *(rires).*

**C : *(rires)*.**

**V :** Mais quand même *(rires)*. 74 ans, j'ai eu cette année, putain. C'est, je suis la dernière d'une famille de cinq, la toute dern'. Et le, le, mon plus jeune frère, il avait dix ans de plus que moi. Donc, quand on était plus que les deux, je l'ai perdu l'année dernière. Là, ça m'a fait un choc, alors je me dis : « presque plus personne ». Voilà, voilà.

**C : Ça va vite *(rires)*.**

**V :** Ça va vite, oui *(rires)*. Mais, c'est marrant parce que quand je, mon père il était 1899, alors c'est Mathusalem hein, en gros *(rires)*.

**C : *(rires)*.**

**V :** Pis ma mère de 1900. Et ma mère, elle m'a eu à l'époque, à 42 ans. Donc c'était, c'était, j'étais pas du tout désirée, c'était, j'étais la, elle m'a toujours dit : « t'étais, tu étais ma ménopause ». Je lui ai dit : « mais c'est sympa ! »

**C : *(rires)*.**

**V :** Alors voilà.

**C : Donc, donc vous avez, voilà, du, du soutien de la part de vos proches ?**

**V :** Ah oui, oui, alors j'ai une fille qui est, qui est juste un bonheur pur et simple, qui, qui m'est venue à l'hôpital, et qui vient avec moi souvent aux, aux consultations avec l'oncologue. C'est, c'est ma, c'est mon oreille, parce que comme je disais à l'oncologue, au bout d'un moment, j'ai dit : « moi je zappe, j'écoute plus parce que ». Pis il me dit : « mais c'est tout à fait normal » *(rires).* Alors elle est, elle est attentive. Pis bon elle est infirmière, ça aide aussi.

**C : Oui c'est vrai *(rires)*.**

**V :** Oui *(rires)*.

**C : Donc voilà, vous avez ce soutien de la part de.**

**V :** Ouais.

**C : De, de votre, de votre fille. Et, ouais, comment ça s'est passé les, les soins, reçus dans le cadre de votre maladie pour vos proches, justement ?**

**V :** Oh j'étais bien, j'étais alors, j'étais bien entourée. Tout, tout le monde me téléphone, venait me voir. Presque, c'était presque un peu trop. Des fois, je disais : « fichez, j'ai, j'ai envie d'être, j'ai pas envie de voir quelqu'un ». *(rires)*. Mais c'était super génial alors, très, très, très gentil. Pis ils sont aux quatre, ils sont aux quatre coins de la planète, 'fin pas aux quatre, mais j'ai une nièce qui est au X *(pays d'Afrique)*, j'ai, j'en ai, j'ai des, des, de la famille au X *(région de Suisse)*, j'ai, à, dans le canton de X *(Suisse)*, 'fin voilà. Mais, bon je sors moins, je vais moins, on fait moins de fêtes de famille, parce que c'est un peu difficile à, à gérer avec l'oxygène, il faut, il faut emprunter un, un… un appareil qui produit de l'oxygène, et pis il faut, c'est, c'est pas toujours, c'est pas toujours disponible. Pis j'ai eu deux ou trois ennuis, où on

m'avait dit que les batteries étaient pleines, pis elles étaient pas pleines, enfin. J'ai un peu râlé, aussi. Mais je, la ligue pulmonaire X *(canton)* c'est, je, je maintiens que c'est une équipe de bras cassés, mais voilà. C'est embêtant, ça. Mais, bon, ça m'a rendu service. J'y suis allée une fois, en X (*pays*), et pis on a, on a pu faire livrer de l'oxygène, pour ça, c'était génial, ils se sont bien débrouillés. Mais comme c'était, c'était dans un, dans un petit village, et pis c'était, un, enfin une maison perdue dans la campagne, c'est assez difficile, parce qu'il faut être sûr qu'il y ait quelqu'un pour réceptionner le truc. Faut faire tout un binz, pour *(rires)*. Mais bon, ça avait fonctionné quand même, ça avait fonctionné. Mais bon, là je, j'aurais pu y aller cet été à nouveau, mais il faisait trop chaud, j'ai dit : « non je vais pas aller là-bas, pour rester enfermée dans la maison, autant rester chez moi ».

**C : Ouais, ouais. Donc voilà, c'est un peu difficile parfois pour certains événements, [en termes d'organisation ?**

**V :** Oui, voilà, c'est un peu,] c'est un peu, mais bon on fait avec, quoi, c'est, c'est, c'est, c'est. Bon là j'étais invitée aux 30 ans d'une de mes petites nièces, pis j'avais, j'ai dit : « mais y a trop de monde ». Pis c'était à la montagne. J'ai dit : « non, mais, je, je, je veux pas venir ». Parce que, y a aussi le fait de voir qu'y a quand même un nombre de choses que vous pouvez plus faire, ça vous sape votre moral, et pis ça j'ai pas envie *(rires)*. Ça je veux garder mon moral quand même. Parce que ça me rappelle mon père, je me souviens que je les avais amené mes parents, j'avais des amis, en X *(région d'un pays voisin)*, et qui habitent près de X *(région d'un pays voisin)*, et puis on est, je les avais amenés avec moi, on était allé après voir le X *(monument)*. Mon père avait deux cannes, il pouvait plus marcher, il a, je me souviendrais toujours de ce regard triste, de pas pouvoir aller plus près. Alors j'ai dit : « non, je veux pas ça, je veux pas ! » *(rires)*. Alors j'ai pas été à ce, à cette fête.

**C : Ouais, donc certaines, certaines activités où c'est un peu plus [difficile ?**

**V :** Ouais, ouais, voilà.] Mais sans ça, ça, ça va. Parce que j'ai, je vais quand même au théâtre, je vais quand même. Mais j'ai moins besoin de voir les gens comme avant, on se retrouvait dans un, dans un bistrot pour un apéro, tous les copains. Bon de temps en temps, je passe dire bonjour, mais, c'est pas, j'ai l'impression de pas les avoir quitté, qu'y a toujours les mêmes discussions *(rires)*. C'est un peu rasant *(rires)*. Pis si vous buvez pas trop, parce que, bon, j'aime bien boire un coup de temps en temps, mais, pas forcément tout le temps. Il faut partir assez vite, parce qu'on n'est plus du, pas dans l'histoire, quoi c'est, c'est. Bon, 'fin on a tous été, j'avais été comme eux, aussi, hein. Mais j'entends c'est des fois rasant, c'est vrai.

**C : Oui *(rires)*. Et, donc voilà, vous disiez beaucoup de, beaucoup de soutien, et parfois presque un peu trop, vous avez envie de, de moments un [peu seule ?**

**V :** Ouais, ouais.] Mais, bon c'est parce que, bon j'ai quand même un nombre de belles-soeurs, comme on était, on était cinq enfants, y a, y a quand même du monde, hein. Pis y a des, y a des, j'ai des neveux et des nièces. Pis on a, on a une bonne entente, on a une bonne entente. A part les enfants de mon frère ainé. Mais bon, mon frère ainé, je m'entendais pas beaucoup avec lui, alors c'est pas, mais j'aime bien ses enfants, mais ils sont, ils sont, ils se parlent pas pour des questions d'héritage sordides, le fric, moi ça me, 'fin voilà *(rires)*.

**C : *(rires)*. Donc voilà, vous avez une grande famille, [et, et, et.**

**V :** On a une grande famille ouais,] c'est, c'est, c'est sympa. Pis bon, on est, ma fille elle aime aussi bien les, la, la famille, quoi. Et c'est… voilà. Mais ça, ça, ça f', les, les gens partent les uns après les autres, quoi, mais c'est, c'est, c'est la vie, c'est normal. Ça va bien m'arriver un jour aussi, faut pas que je me leurre *(rires)*.

**C : Oui. Et, si on regarde un peu, peut-être au niveau des symptômes physiques de, de, la BPCO, comment se déroule la gestion de ces symptômes physiques ? Que ce soit la difficulté à respirer, vous avez parlé de ça un peu, la fatigue ? Avec les soins que vous avez reçus ?**

**V :** Non, ben c'est, c'est, bon j'ai des médicaments, j'ai… j'ai de l'oxygène. Il faut, il faut bien gérer, bien gérer, bien gérer tout ça. Mais, disons que c'est quand même assez précieux parce que, ça, ça aide bien ! Alors, je vois pas comment est-ce qu'on peut améliorer, je vois pas comment est-ce qu'on peut améliorer, mais c'est déjà bien, ça soutient pas mal quand même, je trouve… Pis bon, là

maintenant j'ai une physio r', une physio respiratoire qui vient à la maison. Et je m'aperçois que je respire comme, comme, je respirais tout faux, quoi ! Mais dans, pour, pour mieux ventiler les poumons et tout ça, j'avais tout le truc à l'envers. Mais, pis je commence, à 'fin, on est bientôt à la 9ème séance, pis je vais en faire, en tout cas deux séries de 9, après ça va aller mieux. Parce qu'elle m'a donné deux ou trois trucs qui font que j'ai des petites astuces quand j'attends le bus, ou des trucs comme ça, pis je, je mets en pratique. Et puis c'est bien, parce que ça, ça fonctionne !

**C : Donc la, la physio respiratoire, ça a pu vous donner un peu des astuces pour [mieux.**

**V :** Pour mieux gérer ma,]

**C : Gérer.**

**V :** Ouais, voilà, ouais.

**C : C'est, la respiration.**

**V :** Pis c'est une dame extraordinaire, parce que y a des, non, je trouve. Pis elle explique bien, donc c'est… c'est, pis, ouais, non, j'ai, j'ai, j'ai, j'ai bien apprécié ça. Et pis le, l'oncologue, il m'avait dit : « oui, oui, ça il faut, alors, il faut absolument, pis on va vous, faut choisir ». Parce que c'est vrai que d'aller en salle ou des trucs comme ça, c'est pas évident. Et pis, je, je vois que, d'après ce qu'elle explique et pis le, l'échelle de, de ce qu'on fait, si on le fait à l'hôpital, où y a <u>tout</u>, y a <u>tout</u> à portée de mains si y a quoi que ce soit, les données, les, les, les, les maximums, à la maison, ils sont plus bas pour, et pis je pense qu'on le fait mieux.

**C : Donc c'est mieux à la maison que.**

**V :** Ouais, ouais.

**C : Que à l'hôpital ?**

**V :** Ouais. Parce que bon, y a aussi le temps, ils peuvent pas tous prendre du temps pour vous, et puis, y a quand même du, c'est très, c'est très couru ces séances. Pis bon y a des gens qui comprennent rien, y a des. Mais moi j'étais pas, j'étais pas fière en arrivant, j'arrivais pas à marcher sur un tapis, je reculais *(rires)*. Mais quelle horreur ce truc, c'était la hantise ! Pis après ça a marché super bien, mais. Je me disais : « mais qu'est-ce que je dois avoir l'air gourde ! »

**C :** ***(rires)*****. Donc voilà, la physio a pu vous donner des explications, des informations sur la, sur la BPCO ?**

**V :** Non, mais m'aider à respirer convenablement déjà, je pense que c'est, que c'est précieux.

**C : Oui, oui... Et en termes d'informations que vous avez reçues sur la BPCO, son traitement, son évolution, quelle est votre appréciation de ces informations ?**

**V :** Alors ça, je suis toujours, parce qu'ils font à l'hôpital un, une séance mensuelle de tous les gens atteints de BPCO, 'fin on est plusieurs à aller. Ça fait, ça fait, je sais pas, bientôt quatre ans que j'y vais, régulièrement. Et puis on se donne des astuces pour nos masques, d'autres trucs comme ça. Bon, y en a déjà trois ou quatre qui sont décédés. Ça fait toujours un peu un, un creux autour de la table, mais, ils viennent, ils viennent des nouveaux, et pis. Y a des gens qui, qui ont très peur de tous ces, ces accessoires qu'y a, par exemple, j'ai un sac à dos avec un, un appareil qui, qui me distribue du, de l'oxygène, que je remplis chez moi parce que j'ai le cube aussi. Et, là y a des tas de gens qui veulent pas, qui veulent, qui : « non, ça je veux jamais, je, je veux pas, je veux pas ». Moi je voulais pas non plus, pis quand je l'ai reçu, j'ai dit : « mais avec ça je sors <u>tout de suite</u>, parce que sinon je vais <u>jamais</u> mettre les pieds dehors ! » Et pis alors y a, y a tous les, y a tous les commentaires, les enfants, c'est génial : « qu'est-ce que t'as ? » ; « je peux pas souffler ». *(rires)*. Pis y a les autres : « y a de la fumée qui sort, qu'est-ce que c'est ? » Alors suivant la tête des gens, je dis : « c'est pas grave, ça va sauter dans deux minutes ». Alors tout le monde *(signe de peur)*. J'ai fait ça dans un train, après j'ai dit : « non, je rigole, je rigole ». Mais parce qu'y avait quelqu'un qui m'avait agacé, j'avais entendu qu'il disait à sa copine : « t'as vu dans quel état elle est, celle-ci ». Et pis j'avais envie de lui dire : « mais ta gueule ! » Et puis, après y a

une dame qui dit : « mais ça fume, ça fume ! C'est pour'» Pis j'ai dit : « mais ça va sauter dans 30 secondes, faut pas vous en faire ». Et je la vois comme ça, j'ai dit : « non, non, je plaisante ». *(rires)*. Et l'autre, et le mec, il a aussi, j'étais contente, parce que j'avais fait mon effet *(rires)*. Il m'avait embêté, tiens, retour *(rires)*.

**C : Ça avait marché *(rires)*.**

**V :** Mais voilà.

**C : Mais, voilà, vous avez pas mal de soutien dans ce groupe ?**

**V :** Y a beaucoup de soutien, si, si, pis y a, y a mon, y a mon pneumologue de l'époque. Et puis y a, y a l'équipe du, des quatre infirmiers à X *(commune)*, ils viennent aussi, tous les six mois contrôler mon, mon CPAP. Et, c'est une entente, c'est des gens extraordinaires, on s'entend super bien ! Et un jour, j'avais, j'avais plus d'oxygène, j'ai téléphoné, pis ils ont dit : « mais venez, on vous prête un truc, vous le rendez quand vous pouvez ». Très sympa, quoi ! Je sais que là j'ai toujours un, un appui.

**C : De la part des professionnels ?**

**V :** Ouais, ouais, voilà.

**C : De la famille, pis aussi de ce groupe [de, de patients ?**

**V :** De ce groupe, ouais, ouais.] Pis l'oncologue, tout ça. Je suis, je suis… c'est, c'est, c'est, le, l'oncologie chez qui je suis soignée, c'est la Rolls, des médecins, c'est les Rolls là. Et pis, et pis, humains, très humains, pas du tout grosse tête, parce que j'ai eu des Professeurs que je trouvais qui avaient quand même la grosse tête. Et le *Docteur E*. qui, qui est Professeur, il a, il a pas la grosse tête, et pourtant Dieu sait s'il mériterait de l'avoir *(rires)*. Mais c'est un, c'est un type génial, génial, génial ! Très humain, et, et tout, et pis toute son équipe, il s'entoure de gens, enfin il sait, il sait qui il prend avec lui, quoi.

**C : Donc une bonne équipe, [une bonne prise en charge oncologique ?**

**V :** Une bonne équipe, ouais.] Ouais, ouais. Et ça, ça fait du bien à la tête hein, je peux vous dire ! Ouais…

**C : Donc, pas vraiment besoin d'informations supplémentaires ? Vous auriez pas voulu avoir d'autres informations sur la BPCO ?**

**V :** Non, mais je, parce que, bon on entend aussi tous les gens qui ont été opérés, qui expliquent ce qu'ils ont eu, et tout. Pis, moi je m'estime que j'ai de la chance parce que j'ai pas tout ce tralala. J'ai pas d'opérations, donc, c'est déjà, c'est déjà, je suis très contente. Mais bon j'ai, j'ai, je suis une embêteuse, parce que j'ai des bactéries, et pis, j'ai jamais autant mangé d'antibiotiques que depuis le mois de janvier. Et ça, j'aime pas trop, mais bon je les supporte. Mais bon, je fais attention de manger beaucoup de Yaourts. Et je fais un peu attention de l'autre coté, et pis. Mais ça va, je supporte, tu me dis : « vous avez une mine radieuse ». Je dis : « oui, mais je suis pas malade, quoi, c'est tout ». *(rires)*. Mais quand même, de temps en temps ça revient, mais. Là, je me réjouis, je me réjouis, je me réjouis de voir le résultat, je dois aller faire un scanner jeudi, alors on va voir si ma, ma tumeur a diminué. Parce que, avec l'immunothérapie, au départ, avec tout ce qu'ils envoient, elle peut facilement grossir. Pis, envoyer des zones à d'autres trucs, pis ficher en l'air pas mal de trucs à côté, mais. On avale quand même pas mal de, de trucs, pas racontables *(rires)*. Mais voilà.

**C : Donc ce rendez-vous important cette semaine.**

**V :** Ouais, et pis le, l'autre, et pis mardi, lundi le rendez-vous, lundi d'après, rendez-vous avec le, l'oncologue, et pis le, le mardi la cinquième immunothérapie, ou sixième, je sais plus, faut que je regarde. Je vais pouvoir regarder, parce que j'ai noté *(elle cherche son agenda)*.

**C : Dans votre agenda *(rires)* ?**

**V :** Ouais, ouais moi je dois noter tout là…. *(elle regarde dans son agenda).* La cinquième…

**C : Ouais pis comme, comme vous disiez ça fait du bien aussi à, à la tête d'avoir un, un bon soutien, [voilà de.**

**V :** Ouais, tout à fait,] tout à fait ! Alors ça c'est très, très important, je trouve, très important !... Pis bon, on rigole en plus, on plaisante pas mal… Moi je, toujours, j'ai, j'ai une grande gueule, on dit. Mais bon le, j'ai toujours, j'envoie toujours des vannes. Mais ça marche, parce qu'ils éclatent de rire, les toubibs, alors on, on rigole, et puis après, on parle sérieusement. Mais ça fait du bien, ça fait, ça crée diversion quoi, un peu *(rires)*.

**C : Oui *(rires)*. Des bons moments, donc, voilà… Et si on imagine si vous aviez été dans le groupe qui bénéficiait de cette prise en charge, globale, spécialisée, quelles types d'aides et informations, par rapport à votre maladie pulmonaire, auriez-vous souhaité recevoir en plus ?**

**V :** Je vois pas, je vois pas, je vois pas. Puisque, non, je vois vraiment pas. Pis bon, je sais pas comment, je sais pas exactement ce qu'ils faisaient, donc c'est, j'arrive pas à répondre à ça. C'est pas, c'est pas pour vous embêter *(rires)*.

**C : Non, non *(rires)*. Oui. Donc voilà, pas de, vous auriez pas besoin de quelque chose en plus, ou ?**

**V :** Non, parce qu'il me semble que je suis bien soignée. J'ai, tout ce qu'on peut faire c'est en train d'être fait, donc, c'est moi qu'il faut que, c'est à moi d'en prendre bon parti, quoi. Et d'essayer de faire bien tout comme il faut, voilà *(rires)*.

**C : Donc voilà pas de, pas de besoins particuliers [aujourd'hui, ou plus ?**

**V :** Non, non.] Non, ben je vois, parce que vraiment je vois pas, je vois pas.

**C : Oui… Bon ben vous avez, vous êtes plutôt satisfaite avec.**

**V :** Voilà !

**C : Le traitement que.**

**V :** Oui bien sur !

**C : [Que vous recevez ?**

**V :** Oui, oui, oui !] Oui, parce que, comme je vous dis je vois pas ce qu'on pourrait faire d'autre, pour améliorer. Pis bon, c'est… c'est comme ça *(rires)*.

**C : Parfois on peut recevoir aussi du, du soutien au niveau psychologique. Est-ce que vous, quel type de soutien avez-vous reçu vous, au niveau psychologique, vous avez ?**

**V :** Oh j'ai, j'ai, j'ai, j'ai, euh j'ai vu des, des psychothérapeutes, dans le cadre d'un, d'un truc, quand j'étais avec le *Professeur D.* pour les, pour essayer de perdre du poids. Mais j'étais, j'ai un a priori très défavorable, parce que je trouvais qu'ils posaient tous les mêmes questions, ils avaient tous, on aurait dit une partition de musique, tous le même, le même, la même tonalité, les mêmes. Et pis, des, des questions, ils me disaient : « mais, qu'est-ce que vous pensez, qu'est-ce que vous pensez ? » Je sais plus quoi. Et pis j'ai dit. « pourquoi vous êtes là ? » Ou quelque chose dans ce goût-là. Pis j'ai dit : « mais si je savais, j'aurais pas besoin de vous ! » C'est, bon je me suis laissée dire : « c'est moi qui pose les questions, c'est vous qui répondez ». Alors j'ai vite arrêté, assez rapidement *(rires)*, ça m'a agacée. Et c'était, c'était toujours, pis ce côté un peu sirupeux. Ça, moi ça me scotche au plafond *(rires)*, des trucs comme ça. Et pis, et pis voilà, alors c'est pour ça que j'avais pas envie avec l'histoire des trois, des trois fois par semaine, à la physio à l'hôpital, d'avoir encore une, une, un truc comme ça, plus une nutritionniste, plus un, un, un pneumologue, en disant : « mais j'en ai déjà un, pis j'ai un l'oncologue, donc ». Mon pneumologue, je le vois plus, je parle plus qu'avec l'oncologue. Mais il est venu me voir à la maison, l'autre jour. Parce qu'il fait partie de ce, de ce, de ce team qui reçoit les, tous, chaque moi, ça s'appelle « *(nom)* ». Et, il a dit, pis j'ai dit : « mais venez une fois, j'aimerais quand même une fois venir boire un café ». Il m'a dit : « je sais plus si je dois venir chez vous de temps en temps. J'ai l'impression

d'être une affreuse ingrate ». *(rires)* Pis il dit : « non, non, mais comme je sais où vous habitez, je viendrais vous voir ». Et quand il est rentré, quand je suis rentrée à la maison, y avait son assistante qui prenait rendez-vous, quand est-ce qu'il peut venir. Et j'avais trouvé très sympa. Ouais.

**C : Donc vous avez suffisamment et vous avez pas envie d'avoir, d'avoir [du soutien psychologique ?**

**V :** Non ça va, non.] Pis j'ai, j'ai assez de rendez-vous. J'ai l'impression d'avoir plus de temps pour moi. 'Fin, pour moi ent', mais pour faire autre chose que soigner et tout ça. Alors ça devient un peu, un peu lourd ! Mais là ça va quoi, ça va comme ça, mais je veux pas en rajouter. Pour l'instant, j'en ai, parce que j'en ressens pas le besoin, mais si, le jour où j'en ai besoin, peut-être que je changerai d'idée.

**C : Ouais. Mais là… .**

**V :** Là, ça va.

**C : Pas, pas envie de plus [ou besoin de plus ?**

**V :** Pas envie de plus,] voilà, c'est ça *(rires)*. Ras-le-bol ! *(rires)*.

**C : Ouais *(rires)*. Et, parfois certaines personnes ont aussi du soutien plus au niveau spirituel, [ça ?**

**V :** Non, alors je suis pas croyante.] J'ai, j'ai la, la belle-maman de ma fille, la mère de son copain, qui m'a dit : « je vais prier pour toi ». J'ai dit : « mais écoute, te donne pas cette peine, ça sert à rien ! » Et pis elle me dit : « mais tu dois croire ! » J'ai dit : « non, je dois rien du tout ». Mais, mais j'avais un frère, mon frère, mon plus jeune frère qui est mort l'année dernière, qui avait fait des études de, 'fin il, il avait fait, en cours du soir, un Doctorat en théologie. Pis il avait, bon, protestant, alors il avait, il, il faisait les, des, des mariages, des baptêmes, des enterrements, des trucs comme ça. Et puis, j'avais tapé ses thèses, pour la, mais bon, lui il allait à l'église et des trucs comme ça, pis il me dit : « tu viens ? » J'ai dit : « non ! Mais ça m'intéresse pas ! » Je suis pas croyante du tout. Bon, on croit toujours un peu en quelque chose, mais je dis, je suis pas pratiquante, en tout cas. Alors voilà j'ai, alors, c'est marrant, parce qu'à l'hôpital : « vous voulez venir à la messe ? » ; « non ». ; « au culte ? » ; « non ». *(rires)*. Non, mais, bon ça, j'en ressens pas le besoin, donc, je sais pas. Mais je, je trouve que c'est un peu bizarre de, de, de, d'éprouver ce besoin si vous avez jamais, tout d'un coup, prier pour je sais pas quoi, parce que, parce que vous sentez, vous sentez le besoin, pis être complètement à côté de la plaque, je trouve que ça va pas non plus, hein. Alors c'est soit être intègre, ou bien voilà *(rires)*. Alors disons que je suis intègre, oui *(rires)*.

**C : *(rires)*. Voilà, c'est pas, pas quelque chose dont vous auriez [besoin.**

**V :** Non,] non, je pense pas.

**C : Ouais, ouais… On a déjà bien, bien fait le tour *(rires)*.**

**V :** *(rires).*

**C : De pleins de, de sujets différents… Voilà… Est-ce qu'y a quelque chose de, de spécifique ou d'autres besoins qui vous vient à l'esprit, ou ?**
**V :** Non, non, non, je vois, je vois vraiment pas, à part un verre d'eau *(rires) (elle se sert un verre d'eau)*. Vous voulez un verre d'eau ?

**C : Ça va, c'est gentil, j'ai déjà assez, assez bu. Je sais aussi que certains professionnels discutent aussi des, des décisions des soins autour de la fin de vie, ou si on arrive une fois à plus pouvoir décider ce qu'on aimerait comme soins, [ça arrive xxx.**

**V :** Ouais, mais il faudrait que] je fasse un, y a, y a un truc à faire, ses, ses, ses dernières volontés. Mais j'ai pas encore fait, mais faudrait que je fasse.

**C : Ouais des, des, ça s'appelle des directives anticipées, voilà.**

**V :** Voilà, directives anticipées, ouais. Mais j'ai pas fait encore. Mais bon, je me suis arrangée quand même avec ma fille, elle a, elle a, elle a, elle a la signature sur mon, mon, mon, ma banque, et ça, mais faut que, faudrait que je fasse quand même deux ou trois trucs. Mais.

**C : Parce que, voilà, au niveau médical ils ont aussi ces directives anticipées où on peut dire quels soins, on aimerait avoir ou pas, si une fois, dans l'éventualité de plus pouvoir [dire ce qu'on aimerait.**

**V :** Ouais, alors ça je sais pas,] il faudrait que je me fasse aider, parce que je sais pas exactement le. Pis je sais pas comment je me sentirais, pis si je serais pas complètement gâteuse, aussi, parce que y a ça. Moi j'ai une soeur qui est, j'ai une soeur et un frère qui ont, qui ont eu Alzheimer. Donc ma soeur elle était, je suis plus allée la voir parce que j'étais, je pouvais plus dormir pendant deux semaines quand j'allais la voir. Mais elle avait, elle avait… elle avait ce côté, elle avait l'air d'une folle, méchant, presque méchant. Et pis, un autre frère, alors gentil comme tout, et puis aussi Alzheimer. Alors moi quand j'oublie quelque chose, je suis, pis on m'a dit : « non mais c'est pas comme ça ». *(rires)*. Mais c'est vrai qu'avec l'âge, vous oubliez. Je sais pas, je vais, je vais, jusqu'au frigidaire, je me dis : « mais je cherchais quoi ? » *(rires)*. Et, et puis bon, je me souviens pas toujours de la, du prénom des gens. Mais alors, ça je leur demande, je dis : « écoute, je me souviens plus ». Et… ou bien alors je cherche un nom d'acteur, de, de comédien : « attends, attends ». Pis mais ça me revient quand même au bout d'un moment, mais ça peut, ça peut <u>agacer</u> !

**C : De, d'avoir des trous de mémoire ?**

**V :** Oui, ouais.

**C : Ouais. Mais donc là vous, vous avez pas discuté avec un professionnel de la santé du type de soins souhaités, ou ?**

**V :** Non, j'ai pas fait ça encore.

**C : Ouais. C'est quelque chose qui peut se faire ou qu'on, qu'on propose parfois, mais.**

**V :** Ouais, ouais, je sais, on me l'a proposé, mais je sais plus qui sait qui me l'a proposé. Il faut que je, mais je peux, ah ouais mais c'est X *(aide et soins à domicile)*, ils m'ont dit, ouais, on peut faire, je pourrais. Mais bon, faut que je sois bien avec la personne avec qui je parle, parce que c'est, faut qu'y ait pas de, de, il faut que le, le, le feeling passe, parce que sinon c'est un peu difficile, je pense. Déjà que c'est pas drôle de parler de tout ça *(rires)*.

**C : Donc voilà, vous, vous seriez prête à le faire mais avec quelqu'un avec qui, [voilà, le feeling passe et ?**

**V :** Ouais, moi je serais pas anti, anti ça,] mais je pense qu'il faut même le faire, c'est important. Mais, disons que j'attends d'aller un peu mieux peut-être *(rires)*, 'fin d'être. Non, mais là je suis, je me réjouis quand même des résultats de jeudi, donc, ça me travaille un peu quand même *(rires)*.

**C : *(rires)* Ouais, ça vous travaille un peu… Voilà, je crois qu'on a un petit peu.**

**V :** On a un peu fini.

**C : Je crois qu'on a bien, bien fait le tour, à part si vous voyez quelque chose, ou vous aimeriez rajouter encore quelque chose, par rapport à la, à la prise en charge de, de la BPCO ? [Par rapport à des besoins, des ?**

**V :** Non, là ça va, je crois] que c'est assez clair, hein c'est, ça, ça va.

**C : Ouais. Donc voilà j'ai entendu que vous étiez bien soutenue [que ce soit au niveau de la famille, au niveau des professionnels.**

**V :** Oui, tout à fait, ah ouais tout à fait,] alors je suis, je suis dans un, du, de la ouate *(rires)*.

**C : *(rires).* [Voilà aussi avec groupe de.**

**V :** Cotton wool voilà] *(rires)*.

**C : *(rires)*. Avec ce groupe de patients aussi que [vous voyez aussi une fois par mois.**

**V :** Oui, oui, c'est,] ben j'ai rendez-vous vendredi, justement. Mais j'y vais avec plaisir, pis c'est une heure, mais on discute. Et y a un psychiatre là. Mais bon là on l'a, il nous a fait un cancer là, y a quelques temps, pis on l'a pas revu ces derniers temps, là. Mais c'était un monsieur qui avait, c'est marrant, parce qu'il avait de la peine à s'exprimer mais, il écoutait tout ! Il, il avait, des fois je me disais : « mais comment est-ce qu'on peut faire ce métier, pis avoir de la peine ». Ça paraissait ardu, quand il faisait des phrases. Et pis après quand il a eu, après, après il parlait mieux. Et puis après bon, il avait horriblement maigri, il avait une moumoute, et tout, enfin, c'est, c'était assez clair et limpide sa maladie, mais. On ve', il est pas m', il est pas mentionné sur la liste, on verra, on deman' moi je demande chaque fois de ses nouvelles quand même.

**C : Ouais, ouais. En tout cas une bonne entente dans ce groupe, [des bons partages.**

**V :** Ouais, voilà, ouais.]

**C : Vous disiez d'astuces, de.**

**V :** Oui, oui.

**C : Conseils, et.**

**V :** Ouais, ouais, tout à fait. Pis bon y a, y a aussi, y a des gens bizarres, y a un type qui allait, militaire, et pis il faut faire comme ci, faut faire comme ça. Pis il dit, il va chez le médecin pour lui dire qu'il faut qu'il l'opère. Pis le pneumologue qui est là il a dit : « mais il va pas vous opérer s'il, s'il sait qu'il peut rien faire. Faut pas, faut pas croire que c'est vous qui décidez de tout, quand même ». Et des fois il est presque, odieux. Et y a un jour, il dit à une, y avait une infirmière qui venait aussi, pis qui a eu, qui a eu des, des, des opérations pas possibles, très, très gentille, et tout ça. Pis elle dit comme ça : « ouais, pis entre-temps j'ai développé un cancer de ». Je sais pas quoi. Pis, ce type ose lui dire : « vous avez pas le monopole du cancer ». Moi je me suis cramponnée à la table, parce que je me suis dit que je me levais, et j'allais le gifler. Parce qu'on ra', on dit pas des choses comme ça, mais ! Il est, c'est un, alors lui je peux pas le voir, ça, *(rires)* ça c'est, je crois qu'il a compris d'ailleurs *(rires)*. Ça doit se voir tellement, ça doit faire *(bruit)* comme ça *(elle passe la main devant son visage) (rires)*.

**C : *(rires)*. Mais mis à part cette personne, plutôt, voilà, des, des bons échanges, et.**

**V :** Ouais, ouais, non sans ça y a des bon échanges, ça c'est vrai.

**C : Ouais, ouais. Ouais, donc pas de, voilà, vous disiez pas, pas de besoins supplémentaires.**

**V :** Non.

**C : Niveau [soutien psychologique, spirituel, et.**

**V :** Non, ça, ça va, là, ça va.]

**C : Et voilà. Donc tout, tout, tout va plutôt [bien.**

**V :** Tout va plutôt bien,] ouais c'est vrai, c'est vrai, c'est vrai !

**C : *(rires)*. Ben merci beaucoup d'avoir pris le temps de, voilà *(rires)*.**

**V :** Mais je vous en prie. [Parce que je me suis dit que.

**C : Partager là-dessus.]**

**V :** Oui, oui, j'ai dit faut, j'ai dit : « mais je vais me souvenir de rien ». Pis je me suis dit : « faut aller jusqu'au bout, parce que sinon ça vaut pas la peine ».

**C : Oui, oui.**

**V :** Voilà.

**C : Mais c'est, merci beaucoup d'avoir pris le temps de partager tout ça, je pense ça sera bien utile pour, pour la suite. Et on va essayer de.**

**V :** Ouais.

**C : Faire quelque chose avec.**

**V :** Avec tout ça.

**C : Avec tout ça. Essayer de, d'améliorer encore un peu ce qu'on peut offrir comme, comme soins.**

**V :** Mais c'est bien, c'est, c'est bien. *Docteur A. (pneumologie)* je trouve qu'il est quand même bien comme médecin, je… j'aime bien.

**C : Quelqu'un de, de très humain.**

**V :** Ouais, ouais.

**C : Je crois qu'on peut, on peut arrêter ça *(elle arrête l'enregistreur)*.**

# Participant n°3

| |
|---|
| Nom d'emprunt : Louise |
| Âge : 78 ans |
| Sexe : F |
| Groupe : Contrôle |

**C : Donc si je reprends, vous avez participé à une étude, qui avait pour objectif de comparer le traitement habituel de votre maladie pulmonaire, la BPCO, avec une prise en charge précoce, soutenue et intégrée. Vous avez été dans le groupe qui bénéficiait des soins habituels. Donc, pouvez-vous me dire comment ça s'est passé ?**

**L :** Ben, très bien, xxx, elle m'a envoyé les feuilles, je les remplissais, pis elle venait les reprendre. Je veux dire, qu'y a pas eu tellement de, de suivi médical, en fait. Y a pas eu de discussion tellement médicale.

**C : Voilà, vous aviez juste.**

**L :** Alors évidemment les feuilles, c'était des hauts et des bas, c'était pas toujours, chaque deux mois ou je sais plus, le même résultat, suivant comment j'étais, c'est tout. Parce que ça fluctue, hein.

**C : Mmh. Donc vous aviez, voilà, juste là, quand vous étiez dans ce, le groupe ou y avait le traitement habituel, vous aviez juste la, la phase de questionnaire, c'est ça ?**

**L :** Voilà, c'est ça, [rien de plus.

**C : Voilà, oui, oui.] Oui.**

**L :** Oui.

**C : Et, qu'est-ce que vous attendiez en participant à cette étude ?**

**L :** Rien du tout, en fait. J'ai dit ça peut peut-être servir à quelqu'un, aux médecins et tout ça, c'est tout !

**C : Mmh, mmh. Donc pas de.**

**L :** Rien.

**C : [Pas d'attentes.**

**L :** Pas], rien d'autre pour moi, non, non, non, non, j'ai pas pensé à ça, non.

**C : Oui. Donc c'était plus pour.**

**L :** Pour la prise générale. Pour la connaissance générale de ces problèmes. Mais pas d'attentes pour moi.

**C : D'accord, oui. Donc, quand vous avez appris que vous étiez dans le groupe qui bénéficiait des soins habituels, comment vous avez réagi ?**

L : Bon je me suis même pas posé cette question, non *(rires)*

**C : C'était, voilà, vous participiez juste pour.**

**L :** Voilà, c'est ça, ouais, ouais, ouais.

**C : Ouais. D'accord. Et si on pense, comme vous avez eu les soins habituels pour la BPCO, quelle est votre appréciation générale des soins, que vous avez reçus, pour [votre maladie pulmonaire ?**

**L :** Que j'ai reçu à l'hôpital], ou que je reçois maintenant ?

**C : De manière générale.**

**L :** Ben *(soupir)* comme j'ai dit, c'est des, c'est, y a des hauts et des bas, hein. Là depuis trois jours, je souffle un peu, j'ai de la peine à. Mais je vais marcher quand même tous les jours. Mais je veux dire que, y a des moments, où les examens, parce que j'y vais tous les trois mois chez le pneumologue, où c'est en hausse, et pis tout d'un coup ça redescend, enfin ça fluctue. Suivant le temps, je pense, ou bien psychologiquement, je sais pas.

**C : Mmh. Donc y a des fluctua', [des fluctuations**

**L :** Ouais, ouais, ouais], c'est pas stable. Ça c'est sûr.

**C : Donc voilà, aussi avec des, des phases.**

**L :** Et pis après l'hôpital ce printemps, j'ai pris l'oxygène pas trop longtemps, *(rires)* j'ai tout laissé tombé. Et c'est remonté très bien ! Il avait pas eu de si bons résultats depuis deux ans. Et la dernière fois, c'est redescendu un peu. Et là je pense que c'est de nouveau assez bas.

**C : Ouais, là c'est un peu, [c'est un peu plus en bas de.**

**L :** Ouais, ouais, ouais, ouais], je, j'ai de la peine.

**C : A respirer ?**

**L :** A respirer, oui.

**C : Oui, oui, oui. Et, et peut-être de quoi d'autre auriez-vous besoin pour justement, [en termes de traitement ?**

**L :** Ben rien].

**C : Rien ?**

**L :** Rien du tout, je pense pas… Non, non.

**C : Vous avez, vous avez ce qu'il faut ?**

**L :** Oui, oui, j'ai pas l'impression que je peux avoir autre chose, hein *(rires)*.

**C : Oui, oui, oui.**

**L :** Ouais.

**C : D'accord. Et, peut-être par rapport aux informations, que vous avez dû recevoir sur la BPCO, son évolution, son traitement euh [quelle .**

**L :** J'ai] eu ces informations y a cinq ans, à Rolle. Mais j'ai tout oublié *(rires)*. Parce que c'était, aussi, oral. Elle nous informait avec un grand tableau et tout. Mais j'ai tout oublié. Je crois que j'écoute plus au bout d'un moment. Je mets ça de côté. Donc je sais pas du tout, [ce que c'est.

**C : D'accord. Ouais. Donc [vous sav'**

**L :** On peut] dire que zéro.

**C : D'accord *(rires)*.**

**L :** *(rires)*.

**C : Zéro information *(rires)*.**

**L :** Non, mais c'est pas la faute des médecins *(rires)*, c'est de ma faute à moi. Je me suis même pas renseignée.

**C : Oui. Donc vous désiriez pas.**
**L :** Je me suis plus renseignée sur la macula, ben y a dix ans, quand j'ai eu un oeil, j'ai perdu un oeil, que là-dessus, parce que, pour moi, la vue c'est essentiel, pour lire. Et pis, le reste je me suis même pas renseignée… Depuis que je suis née, j'ai des problèmes pulmonaires. J'ai eu une tuberculose, xxx, pis après ça n'a pas arrêté. Donc, *(soupir)* je vis avec, hein...

**C : Vous aviez pas, pas besoin d'aller chercher plus d'informations ?**

**L :** Non, en fait. Toute façon, ça va pas partir, hein. C'est pas guérissable, ça, si c'était quelque chose, qu'on puisse guérir comme un cancer ou comme ça, là je m'informerais. Mais là, je m'informe pas, c'est, c'est comme ça !

**C : Oui. Donc pas, pas de besoins [en particulier de ?**

**L :** Non, non].

**C : D'informations [supplémentaires ?**

**L :** Sans ça je], j'allais sur internet, hein ! Mais, xxx c'est une curiosité que j'ai pas eue à propos de ça… Pour d'autres choses oui, mais pas à propos de ça. Peut-être que j'éloigne hein, je vis dans le déni, en fait.

**C : Peut-être que comme vous disiez, comme y avait pas de cure ou de guérison par rapport à, à d'autres maladies, vous alliez pas chercher plus loin.**

**L :** Voilà, c'est ça, ouais…

**C : Ouais. [D'autres.**

**L :** Ouais, ouais.]

**C : Informations...**

**L :** Non, pis, je trouve sur internet, c'est toujours, angoissant, anxiogène hein, moi j'ai pas envie de ça... en fait.

**C : Ouais, c'est les [les informations.**

**L :** Non, ça m'embête,] je sais pas… je sais pas ce que j'en retirerais, d'aller chercher plus d'informations. Je prends les médicaments qu'on me dit de prendre, je vais marcher, parce que le pneumologue dit faut que je marche. C'est vrai que ça me fait du bien, mais c'est vrai qu'aussi cet après-midi j'irai me coucher, mais, et puis je me dis : « bon ben j'ai, je me sens quand même assez en forme hein ! » Je vais pas courir un marathon, ça je sais.

**C : Donc voilà, vous avez pas besoin, vous prenez les médicaments, et vous avez pas besoin de.**

**L :** Ben je prends toujours des choses dans mon sac, si jamais j'ai une, une détresse respiratoire. Mais j'en avais beaucoup y a trois, quatre ans, mais j'ai plus maintenant, plus tellement, hein ! Parce que moi j'anticipe, la détresse. C'est quand je rentre le soir, j'aime pas avoir l'hiver et tout, la pluie, tout ça, et pis là tout d'un coup je me provoque, une détresse. Donc, je sais, qu'il faut pas que j'anticipe. Mais ça, c'est difficile *(rires)*.

**C : Oui *(rires)*. Vous avez des, des astuces.**

**L :** Oui, je connais un peu les choses, depuis le temps. Alors j'utilise ces connaissances-là, mais les connaissances qui sont, qui sont spécifiques à moi, en fait. Qui sont sûrement pas les mêmes pour une autre patiente.

**C : Donc c'est vos propres informations.**

**L :** Ouais, ouais.

**C : Et votre expérience.**

**L :** Oui, c'est mes informations corporelles, c'est tout, hein ! Pas extérieures.

**C : Ouais, ouais. Vous ajustez par rapport à ça ?**

**L :** Ouais, tout à fait.

**C : Oui.**

**L :** *(rires)*.

**C : *(rires)*.**

**L :** Au lieu de marcher 35 minutes, je marche 20 minutes. Hier, c'est ce que j'ai fait, parce que je pouvais pas aller plus loin. Et ça, depuis trois mois je marchais bien, 35 minutes, 45 minutes par jour. Mais là, depuis deux jours, non.

**C : [Vous disiez que ça fluctue.**

**L :** Ça, ça m'embête un peu], oui. Ma foi, c'est comme ça, hein. *(Elle se tape le coude sur la chaise)* Aie. C'est pas vrai !

**C : *(rires)*. Donc, justement par rapport à la gestion des symptômes, vous avez ces médicaments, comment ça se déroule, pour gérer les symptômes physiques de la BPCO, que ce soit ?**

**L :** Ça, ben je sais pas, j'ai des médicaments qui sont réguliers depuis quatre, cinq ans, et pis voilà… C'est des prises régulières, et puis voilà, c'est tout.

**C : Ouais. Donc vous avez pas, d'autres besoins ?**

**L :** Non, non, non. Cet été, j'ai arrêté l'oxygène assez rapidement, je me suis fait engueulée par l'entreprise qui donne les, les bonbonnes, et tout. On doit pas le faire soi-même. Mais ça faisait deux mois qu'ils venaient pas remplir. Alors j'ai dit : « toute façon, c'est pas la peine de m'engueuler ! J'aurais pu mourir pendant ces deux mois ! ». Non, non, j'ai arrêté très vite. En sortant de l'hôpital, ils m'avaient dit que c'était pour longtemps, mais j'ai arrêté très vite.

**C : Donc c'est, voilà, vous qui avez décidé [d'arrêter le, l'oxygène ?**

**L :** Ouais, ouais, oui, oui, tout à fait], ouais. Je mettais des fois le soir, la nuit, parce que j'avais l'angoisse, mais c'était pas pour respirer, c'était pour calmer mes angoisses. Je me disais : « là au moins, j'ai quelque chose pour respirer ». C'est tout. Pis après, j'ai abandonné, tout ça.

**C : Oui. Donc voilà, juste, [vous l'utilisez de temps en temps.**

**L :** Non, pis c'est] épouvantable pour quelqu'un qui a de la peine à respirer, de lui foutre une petite bonbonne qui pèse 3 kilos, sur le dos. D'autant que c'est pas une maladie qu'on a très jeune, donc on a plus de peine à porter. Et je trouve que c'est incohérent ! xxx, faut prendre un chariot et tout, non !

**C : Mmh. Donc là vous avez, vous avez arrêté ?**

**L :** *(rires)*. J'ai acheté un sac à dos. Et puis après, j'ai arrêté. Oui, j'ai gardé quand même presque trois mois, hein…

**C : Oui. Mais là ça faisait trop en termes de poids ?**

**L :** Tout. Pis je me sentais tributaire, quand même. Je me sentais liée à ici dans la maison, le fil il fait 15 mètres, hein. Je me sentais tenue en laisse ! Pis je pouvais rien faire sans. Mais c'est vrai que je perds le souffle hein, suivant ce que je fais. Avant-hier, j'ai un peu astiqué. Parce que j'avais pas un parquet vitrifié. Et après pour faire, voyez là j'étais à bout de souffle… Bon je gère, je m'assieds, je respire, pis ça passe.

**C : Donc là oui, vous vouliez plus être dépendante de, [du fil ?**

**L :** Du fil], non, non, non, non. Ça, je voulais pas. On peut rien faire avec ce truc. On se prend les pieds dedans, c'est même <u>dangereux</u>, je trouve !

**C : Oui. Donc là, vous avez un petit peu moins de souffle, lié à ça ? Vous me disiez, [de temps en temps**

**L :** Non, non], j'ai, là, ça va pas très bien ces trois jours, ces trois quatre jours. Je sais que l'hiver, c'est assez difficile… Mais ça, c'est aussi dans la tête hein, attention ! J'anticipe un peu, là, de nouveau.

**C : Mmh, vous pensez que c'est un peu plus dans votre tête ?**

**L :** Je peux dire que c'est, c'est pas moitié-moitié, mais y a quand même beaucoup dans la tête, hein. Pour tout, d'ailleurs. [Je vais boire une goutte d'eau.

**C : Oui, prenez !]**

**L :** Vous voulez un verre d'eau ?

**C : Ça va, c'est gentil, merci. Prenez, prenez.**

**L :** Je suis tout de suite à vous. Je sèche, parce que j'suis, j'ai.

**C : Et pis je vous fait parler, en plus *(rires)*.**

**L :** Non, mais c'est. *(Elle se sert un verre d'eau.)* Je suis comme ça, c'est dans la tête, toujours. Qu'est-ce qu'il s'en passe dans la tête, c'est terrible !

**C : *(rires)*.**

**L :** Voilà.

**C : Donc vous aviez l'oxygène, vous avez décidé de, d'arrêter.**

**L :** D'arrêter .

**C : Vous auriez eu besoin d'autre chose, ou ?**

**L :** Non, non, pas du tout. Même à X *(clinique)*, je pensais pas toujours à prendre la bonbonne. Et là, les infirmières, elles piquaient des crises… parce que…

**C : Oui *(rires)*, oui.**

**L :** Non, non, ça va, ça va. J'ai une bonne saturation, et tout. J'ai… ce problème des jambes m'ennuie, parce que ça me fait mal et puis je, j'espère que j'ai pas d'infection qui s'est déclarée en plus. Mais j'y vais tous les deux jours, alors. Bon, moi ce qui me fait peur maintenant l'hiver, c'est de prendre le tram. Parce que je prends beaucoup le tram, j'ai des cours à la rue de X, c'est que y a des

gens qui toussent, y a des gens. Et là je me dis, c'est, mon fils me dit : « t'as qu'à mettre un masque ». J'ai dit : « écoute, arrête ! ».

**C : Ouais. Donc, des situations [comme prendre le tram.**

**L :** Voilà. C'est pour ça l'hiver], c'est pour ça que j'angoisse un peu. Parce que, là je descends à la clinique de X tous les deux jours, ben je suis dans le tram, hein. Je descends pas à pied quand même, c'est un peu loin *(rires)*.

**C : Ouais *(rires)*. Donc c'est un peu angoissant l'hiver avec les personnes qui toussent [dans le tram.**

**L :** Ouais, ouais c'est ça,] ouais. Moi je chope tout, quoi ! Parce que l'année dernière, j'ai chopé cette grippe horrible, mais j'avais la, j'avais le vaccin. Bon il a décidé de m'en faire deux, cette année. J'ai déjà eu un, pis y en aura un deuxième. Parce qu'évidemment, on est beaucoup plus vulnérable, avec cette maladie. Y a un terrain qui est, qui est plus faible, pis les bactéries, elles foncent dessus *(rires)* les garces *(rires).*

**C : Oui *(rires)*. Donc là vous avez des médicaments régulièrement, plus ben là, [les vaccins.**

**L :** Pour le souffle], ouais j'ai les médicaments pour respirer, en fait ! Les aides respiratoires, ouais…

**C : Oui, oui… Et, vous disiez aussi que y a pas mal de choses qui sont dans la tête, est-ce que vous reçu du soutien au niveau psychologique, ça peut arriver parfois ?**

**L :** Alors, à l'hôpital, ils m'avaient envoyé chez un psychiatre. Non, d'abord ils m'en ont envoyé un qui est, qui est venu deux mois, et tout. Et on a papoté, pis il me parlait de lui, finalement. C'était un médecin qui avait fermé son cabinet de médecine générale, et pis qui avait repris des études de psychiatrie, qui avait fini, très, très sympa, et tout. Et pis après, ils ont dit : « non, maintenant, vous devez prendre quelqu'un, en dehors ». Ils m'ont donné l'adresse au X. Pis je crois, trois fois, pis. Y avait pas de contact entre nous, et tout, pis je voyais pas ce que je faisais là. Je voulais pas qu'il me ramène dans ma jeunesse, qui était un peu pourrie, et je voulais pas. Donc je voyais pas ce qu'il allait pouvoir faire pour moi. La perte de mon mari, je peux gérer moi. J'ai pas besoin de quelqu'un pour m'aider, à faire ça. Et puis, pis au bout de trois fois, j'ai dit : « non, j'arrête. Ça collera jamais, entre nous d'ailleurs ».

**C : Ouais. Donc là, vous aviez quelqu'un, mais pas vraiment de contact, donc vous avez.**

**L :** Non, non, mais j'ai arrêté là. J'ai été trois fois.

**C : Oui, oui. Et de, vous auriez eu besoin de, d'autre chose, ou ?**

**L :** Ben non. Mais je sais pas, c'est très bizarre, je crois, je vois pas ce qu'on peut m'apporter, en fait.

**C : Oui, oui…**

**L :** C'est surtout ça… Non, j'suis assez rétive, pour *(rires)*.

**C : *(rires)*.**

**L :** Essayer de me faire de la sophro' euh l'hypnose pour me faire arrêter de fumer. Impossible !... Il faudrait que je fasse tous les hypnotiseurs de la ville pour en trouver un qui arrive *(rires)*.

**C : *(rires)*. L'hypnose, ça a pas marché.**

**L :** Non, non. J'avais une amie qui se faisait soigner les dents, comme ça. Mais y a déjà 30 ans, 40 ans. Alors là, il faudrait s'y mettre longtemps, hein. Non, non y a rien à faire *(rires)*. [Ça ne marche pas !

**C : Pour l'arrêt du tabac], ça marche pas.**

**L :** Non, non. Alors là, j'ai arrêté quand j'étais à l'hôpit'… quand j'étais à l'hôpital au début de l'année, j'ai pas fumé du tout. Après, j'ai arrêté, ben j'ai pas repris. Et puis, pis j'ai repris. Mais je fume deux, trois paq', trois cigarettes par jour. Mais des très basses, c'est-à-dire y a 0.00 truc, et pis voilà. Mais, c'est vrai que tous les jours je me dis : « faut que j'arrête ». D'autant que j'écoute la radio toute la journée et, c'est le mois de l'arrêt. Ah il est en bon état votre carton là. *(elle regarde le carton de l'enregistreur)*

**C : Oui *(rires)*.**

**L :** *(rires)*. Vous avez des rats chez vous *(rires)* ?

**C : Non *(rires)*. C'est un vieil enregistreur et un vieil *(rires)*, un vieux paquet, ouais *(rires)*.**

**L :** Non, j'ai pensé que c'était un hamster [ou un petit rat *(rires)*.

**C : Non, non, non, je crois pas].**

**L :** Non, parce que y a le petit trou là, j'ai dit c'est peut-être une petite souris qui est passée.

**C : Ah, j'espère pas *(rires)*.**

**L :** Pourquoi ? C'est chou les souris.

**C : Ouais. J'espère [vous en aurai pas ramené.**

**L :** Moi j'ai vécu toute ma jeunesse] avec des souris dans la maison. Enfin dans l'appartement.

**C : Ouais *(rires)*. Et vous m'avez parlé de votre fils donc vous avez du soutien aussi du côté de ?**

**L :** Non, je l'énerve mon fils. On a toujours eu les mêmes rapports depuis qu'il est tout petit. Et ça a pas tellement changé, je l'énerve *(rires)*. Non, pis quand il me voit remonter, comme c'est chaque fois assez rapide, du coup, il se dit : « bon ben elle y arrive, elle y arrive ». Alors je lui demande des aides. Parce que y a des choses évidemment, je dois aller laver ma voiture, mais vraiment la laver, mais il a pas le temps ! Il pense que je vais le faire. Là j'ai refait cet été toute une armoire, l'armoire à balais, je l'ai transformée en armoire, pour mettre les, la nourriture, le riz, les lentilles, et tout ces machins. Mais, il a tout de suite pensé que je pouvais le faire moi-même. Il m'a pas dit : « je viens t'aider ». Hein.

**C : Donc, voilà [dès que.**

**L :** Non, non, non], là il me pose même pas la question, hein !

**C : Donc, dès que vous allez un peu [mieux.**

**L :** Il voit que] ça va, que je repars, et tout. Alors donc, il s'intéresse plus… Il me demande pas. Mais je pense aussi que il a peur, c'est peut-être ça. C'est peut-être la peur, je sais pas, j'analyse, mais j'analyse mal, alors je, je dis bon ben, je l'énerve, en plus. *(sourire)*

**C : Ouais *(rires)*. 'Fin vous avez l'impression que vous l'énervez ?**

**L :** Ah non, je sais, hein *(rires)*. Il me dit : « arrête ! ». Je l'énerve, je peux pas lui poser des questions sur la j' quand il était jeune et tout, j'ai complètement oublié quand il était petit. Mais non, jamais il me répond ! Je l'énerve *(rires)*.

**C : Mais il aurait aussi peut-être peur, un peu, vous disiez ?**

**L :** Je sais pas, peut-être qu'il a peur aussi, ou que, il veut pas. Non, il est content, parce qu'il voit que je suis capable de faire pleins de trucs… Mais c'est aussi comme ça, une poussée, j'ai commencé les armoires et tout, pis après pouf, j'arrête *(rires)*.

**C : Ouais. Mais vous auriez peut-être besoin un peu plus d'aide, par exemple, pour la voiture, ou pour les armoires, ou ?**

**L :** De l'aide. Ou bien qu'il me téléphone une fois par semaine. Non, il vient boire le café. Rapide. Et pis, *(rires)*, samedi il est venu boire le café, moi je m'étais acheté, moi j'adore les casse-têtes *(elle prend un casse-tête sur la table).* Et pis il était là, assis, pis pendant une demi-heure il a essayé *(rires)*. C'est comme si j'étais pas là. Ou bien ce journal *(elle prend un magazine sur la table)*, alors maintenant je les mets là, comme ça il peut pas les prendre. Et il fait son devoir, c'est ça. C'est peut-être dégueulasse, ce que je dis, c'est peut-être pas son devoir. Mais moi j'ai l'impression qu'il fait son devoir.

**C : Qu'il vient rapidement et voilà.**

**L :** Parce qu'il faut venir…

**C : Et vous aviez [d'autres ?**

**L :** Mais] là il me pose jamais de questions sur la maladie et tout ça. Je sais même pas s'il sait ce que j'ai. Je sais pas.

**C : Ça vous, vous en parlez pas ?**

**L :** Ben non. Il voit que des fois j'ai de la peine à respirer et tout, et pis c'est tout ! … Alors il me dit : « t'as pas assez marché cette semaine ». Non, non, il m'accuse, en fait… Il m'aiderait pas à marcher, il me dit : « tu dois marcher plus, comme ça, ça ira beaucoup mieux ». En fait c'est ça, le problème *(rires)*. Bon, j'aime autant ça finalement. Parce que s'il était aux petits soins : « comment vas-tu ma mamie chérie ? » Et tout ça, jamais il a fait ça *(rires)*. On a des rapports assez particuliers. Pis lui c'est un grand sportif, et tout.

**C: Donc.**

L : Donc, il me dit : « j'aurais dû ». C'est vrai j'aurais dû, mais j'ai tellement bossé dans ma vie, que j'ai pas eu le temps. En plus j'avais un mari, fallait vraiment s'en occuper, il était pas capable de faire un oeuf au plat. Et pis voilà. Et pis mon fils, bon mon fils, on peut dire y a longtemps qu'il est parti de la maison, il a 56 ans. C'est… il était adulte très vite. Quand je vois ses enfants, ses garçons, c'est hallucinant. Alors je me suis posée la question s'il fait avec eux ce que j'ai pas fait pour lui… Ils seront encore là à 30 ans, hein *(rires)*. Ça c'est évident. J'en vois pas un quitter la maison.

**C : *(rires)*. Et, en dehors de votre fils, vous avez aussi du, du soutien ?**

**L :** Non. J'ai des amis au cours de calligraphie, que je vois tous les mardis. Ça, pour moi c'est très important. C'est pour ça que le soir, j'ai, mon prof il me dit : « mais tu viens dans la semaine, parce que y a d'autres cours l'après-midi ». Ah j'ai dit : « non ! Moi ce qu'il me faut, c'est aussi voir mes amis ». Et puis, elles sont beaucoup plus jeunes ! Elles ont 50 ans. Y en a une qui me téléphone assez souvent.

**C : Vous avez ces amis du, du [cours calligraphie qui sont là.**

**L :** Oui, oui, oui, oui, c'est tout.] Autrement pas du tout… Oui. Je suis très seule, en fait. Mais ça, ça vient aussi de soi-même, hein, quand on est seule. Y a des gens qu'ont beaucoup d'amis, moi non. Mais ça c'est, je pense que ça vient de moi ça. Je me suis jamais investie dans une amitié. Ça prend du temps une amitié, je pense *(rires)*.

**C : *(rires)* Ça prend du temps.**

**L :** Il faut téléphoner *(rires)*, faut. Et puis c'est pas, non, non, c'est de ma faute, hein.

**C : Mais, de, de quoi d'autre auriez-vous eu besoin, en terme de soutien ?**

**L :** Rien… Non, rien. J'ai X *(aide et soins à domicile)* qui vient me faire le ménage, deux fois par sem', deux fois par mois. Mais, c'est pas un grand soutien ça, parce qu'elles font rien *(rires)*. Je m'énerve, c'est tout. Mais, de soutien, non, j'ai pas l'impression que j'ai besoin de quelque chose. Oui, quelqu'un qui vienne, qui me prenne la maladie, pis qui l'enlè', qui l'emporte avec *(rires)*, c'est ça. Pis des fois je me dis, quand ça va bien, quand je peux bien marcher, je me dis : « finalement, je suis pas mal du tout, ça va ».

**C : C'est juste de, de temps en temps [que vous aimer'.**

**L :** Ce que j'aimerais] c'est, enlever les rides, et voilà *(rires)*. C'est ça et tout d'un coup me sentir comme je suis dans la tête. Parce qu'on se reconnaît pas dans le miroir, quand on regarde des choses. Non, ce que j'aurais besoin c'est d'avoir mes yeux.

**C : De… pour la vue ?**

**L :** Ah ouais, ouais. Je peux presque plus lire… Ouais. C'est ça mon problème, mon gros problème, plus gros que le… que le poumon.

**C : Plutôt les yeux que le poumon ?**

**L :** Oh oui, ça c'est sûr, ouais, ouais… Bon, j'ai des livres là-dessus *(elle cherche sa tablette)*.

**C : Sur ?**

**L :** Mais j'aime bien le papier. Sur la tablette, que je peux mettre les lettres plus grandes. Mais après ce sera fini, je pourrai même plus. Mais… non c'est pas la même chose que le livre.

**C : Ouais. C'est différent *(rires)*.**

**L :** Et puis bon, l'ophtalmo il me dit : « mais vous avez qu'à acheter des, des CDs ». Je déteste ça. Pis je fais quoi avec les mains, pendant que j'écoute un livre. C'est pas possible *(rires)*. Je vais devenir folle. Je prends plus le tricot, parce que j'ai trop tricoté. Je peux rien faire ! La calligraphie, je peux pas, parce que je peux pas faire deux choses à la fois. Ça demande énormément d'attention la calligraphie… Mais ça je peux encore, parce que… je peux faire du gros.

**C : Vous pouvez encore faire, voilà la, [la calligraphie ?**

**L :** Ouais.] J'ai un problème, mais j'arrive quand même à utiliser, c'est que je vois pas le pinceau quand il touche le papier. Alors de temps en temps, je fais un petit peu un sursaut. Je fais, pas assise pour pas me foutre les bras en l'air. Mais, non, je peux encore !

**C : Alors ça [vous pouvez encore.**

**L :** Oui. Hier j'ai fait] un gros travail, mais je peux encore.

**C : Ouais. Ça vous pouvez encore faire, ouais *(rires)*.**

**L :** Oui.

**C : Et si on imaginait que vous aviez été dans le groupe qui bénéficiait de cette prise en charge, globale spécialisée avec l'infirmière qui venait une fois par mois, évaluer des besoins. Quelles types d'aides et informations par rapport à votre maladie pulmonaire, auriez-vous souhaité recevoir ?**

**L :** Rien. J'ai reçu tout ce que j'voulais quand j'étais à X *(hôpital, pneumologie)*, et j'ai rien écouté *(rires)*.

**C : Ouais *(rires)*.**

**L :** *(rires)*. Et ça va pas changer…

**C : Donc [rien.**

**L :** De toute façon] je pense c'est quelque chose qui, qu'on peut empêcher d'évoluer… en étant attentive, en marchant, en faisant des choses, en prenant bien ses, empêcher d'évoluer ça, pas complètement. C'est, comme je vous ai dit, c'est, y a des hauts et des bas. Mais on peut pas guérir !... C'est ça ! Si j'avais un gros problème à la jambe, on peut couper la jambe, mettre une prothèse, c'est fini ! Mais ça, c'est pas possible, on peut rien faire. Ça j'ai bien compris *(rires)*.

**C : *(rires).***

**L :** *(rires)*. Vraiment j'ai bien compris.

**C : Oui ?**

**L :** Ouais.

**C : Donc, comme on peut rien faire, vous avez pas besoin, justement [d'informations, de ?**

**L :** Non, non, non, non,] bien sûr que non ! Si j'avais un cancer, ma belle-fille a eu un cancer du sein, si j'avais un cancer du sein, là j'aurais pris des informations, vraiment, pour voir ce qu'on peut faire, et tout. Mais… là, c'est une évolution qui va… Ma mère en est morte. Parce que j'ai jamais revu ma mère, mais on m'a dit qu'elle était morte de ça…

**C : De la BPCO ?**

**L :** Ouais. Elle avait la bonbonne à la maison depuis des années, paraît-il. Mais elle est morte à l'âge que j'ai, hein. Moi je suis là *(rires)*.

**C : *(rires)* Oui.**

**L :** Voilà, c'est ça…

**C : Donc pas de.**

**L :** Non, non, mais j'ai pas de… la seule chose que je sais, c'est que c'est, on peut stopper un peu, mais c'est évolutif, je veux dire, c'est pas quelque chose qu'on peut. C'est ce qu'il y a d'emmerdant, en fait.

**C : Guérir.**

**L :** On peut pas guérir. Ouais, je peux arrêter mes deux cigarettes. Je crois pas que ça ferait de l'effet ça. Marcher plus… Et puis, *(soupir)*… moi je marche quand j'ai envie de marcher. Là, cet après-midi, je sais pas si j'ai envie. Je me suis levée trop tôt.

**C : Donc, y a certaines choses que vous pouvez faire ?**

**L :** Oui, mais je fais, par exemple, dimanche je suis descendue à la permanence pour me faire refaire mon pansement sur ma jambe. Et puis après, j'ai été au, au marché d'X *(quartier)*. J'ai acheté des légumes et tout, et puis ça m'a fait du bien, j'ai été manger des crêpes chez des amis, et tout. Et, je suis rentrée à la maison, j'étais contente… Non, là je, tout d'un coup quand je me mets en marche, je fais. Mais je suis capable aussi de rester sur ma chaise à faire un jeu sur l'ordinateur, et puis pas bouger, en fait.

**C : Donc ça, [ça fluctue, comme vous disiez.**

**L :** Mais j'en fais trop], quand j'en fais…

**C : Quand vous faites quelque chose, c'est peut-être un [petit peu trop *(rires)*.**

**L :** Oui, oui, trop.] Oui, oui *(rires)*. C'est la culpabilité qui me fait agir, moi. C'est ça, en fait.

**C : La culpabilité de, [de pas faire ?**

**L :** De rien faire,] voilà, c'est ça.

**C : Ça vous pousse à, [à faire, oui *(rires)*.**

**L :** Ouais, ouais.] Bon attendez, pas tout de suite hein *(rires)*. Non, mais je me fais des programmes comme ça, le marché d'X *(quartier)* et tout ça, tout l'été j'ai fait. Ça me fait marcher, les gens sont sympas. Les commerçants aussi.

**C : Comme ça vous avez ces activités de [marche, de sortie.**

**L :** Ouais, ouais,] ouais.

**C : Ouais, ouais, ouais. Et, je sais que parfois certaines personnes ont aussi du soutien spirituel.**

**L :** Non.

**C : Avez-vous reçu ?**

**L :** Non, non.

**C : Non ?**

**L :** Ça m'intéresse pas.

**C : Non.**

**L :** Ma belle-fille, est très zen, elle fait du yoga à haut niveau, et tout. Oui mais alors, elle est… détachée du monde, hein... Y avait des bagarres à table avec les gamins, bon maintenant ils sont plus grands, mais ça arrive aussi. Elle, elle bouge pas, elle bronche pas. Non, non. Le yoga, quand on fait à haute, on est, on est détaché. Y a personne à côté.

**C : Une forme de, de [détachement ?**

**L :** Ouais, ouais.] Parce que mon fils, cette année, il a quand même pris quelques jours, pour faire, il fait du parapente. Pis moi ça m'angoisse quand il part en voiture, et tout ça. Alors je dis qu'il faut qu'il me rappelle, et pis, il m'appelle pas, il m'engueule. Et puis, mais elle, non, jamais. Elle peut rester cinq jours sans qu'il lui téléphone, ça l'inquiète pas…

**C : Donc y a**

**L :** Non, on se ressemble pas du tout *(rires)*. On a le même prénom, mais on se ressemble pas *(rires)*.

**C : *(rires)*. Voilà vous, elle par exemple, ou d'autres personnes pourraient avoir besoin, mais pour vous, ça, pas besoin de, de ce soutien spirituel ou [quelque chose ?**

**L :** Non, pas du tout,] pas du tout, pas du tout… Non. Dans tout ce que j'ai lu, non, non, non, non, non, c'est je sais pas... Non. Pour l'instant, ça va, je suis bien avec moi *(rires)*.

**C : *(rires)*.**

**L :** Ah ben j'ai vu la chatte passer.

**C : Ah !**

**L :** J'espère que vous aimez les chats quand même ?

**C : Oui, oui. [J'aime beaucoup les chats.**

**L :** Elle est passée ?]

**C : Elle est là *(rires)*.**

**L :** Ah ouais.

**C : Ouais, elle est là.**

**L :** Ah, mais j'ai balayé, alors j'ai mis le truc, alors. xxx c'était terrible cette matinée, pauvre chatte.

**C : *(rires)*.**

**L :** Ouais.

**C : Et, je sais qu'aussi certains professionnels parlent des types de soins qu'on souhaiterait en, en [fin de vie.**

**L :** Ah la voilà].

**C : Ah le chat *(rires)*.**

**L :** Non, moi je souhaiterais pas de soins en fin de vie. Bon, ben j'espère que ça va durer encore quelques temps hein, et j'aurai le temps d'y penser. Moi je veux pas que, qu'on me garde en vie. Si ça va plus, ça va plus, un point c'est tout. J'ai donné hein, toute ma vie…

**C : Pas de ?**

**L :** Non…

**C : Voilà donc je sais que, voilà, on peut écrire aussi, y a des, ce qu'on appelle des directives anticipées où [on dit, voilà.**

**L :** Oui, j'avais,] mais je crois que je l'ai viré *(rires)*. J'avais le truc, on m'avait envoyé, mais j'ai fait de l'ordre. Alors quand je fais de l'ordre, je fais que des conneries, hein. Parce que j'ai dû le mettre à la poubelle. Bon, je peux toujours demander à mon médecin, tout ça. Ouais, ouais.

**C : Donc ça, ça on, on vous l'avait proposé, mais.**

**L :** J'avais reçu un petit truc. On m'a pas proposé. Je sais pas j'ai reçu, je pense qu'ils envoient aux personnes qui ont été à l'hôpital ou comme ça. Et puis, mais ils donnent des numéros de téléphone, en fait. Ils disent rien, hein. Pour les renseignements, faut téléphoner.

**C : Oui. Donc, donc voilà c'est pas quelque chose maintenant, mais peut-être [plus tard, pour ?**

**L :** Ben non,] j'espère pas, en fait *(rires)*.]

**C : Plus par rapport à, qu'est-ce qu'on déciderait, en fait. Si une fois, ça arrive qu'on, qu'on puisse plus [donner notre avis.**

**L :** Oui.] Ben là j'ai pas eu le temps, hein, la dernière fois que ça m'est arrivé. J'aurais pas eu le temps de le faire. Peut-être que je devrais faire ça. *(soupir).*

**C : Voilà c'est, si dans l'éventualité où on, où on peut peut-être pl' [pas communiquer**

**L :** Plus rien faire].

**C : Ou voilà, on n'arrive pas à dire ce qu'on aimerait, y a cette possibilité [d'écrire.**

**L :** *(En s'adressant au chat qui mange de l'herbe à chat sur la table) : «* c'est bon hein,] tu vas me dégueuler quelque part, hein ». C'est pour ça que j'ai passé xxx 'stique partout *(rires)*.

**C : *(rires)*. Donc voilà, y a cette possibilité de dire à l'avance en fait, [si un jour ça nous arrive.**

**L :** Ouais. Faudrait que je fasse, ouais.] Faudrait que j'écrive ça. Je devrais écrire, pendant que je peux encore écrire, faudrait en profiter. Je peux pas écrire sur l'ordinateur parce que je vois pas, pas assez. Avec les, le crayon, ça va mieux. Bon.

**C : Donc ça, vous pourriez ?**

**L :** Je pourrais faire un truc, un papier, ouais. Pis le donner à mon fils.

**C : Ouais. Mais ça, ça c'est pas quelque chose que vous avez déjà fait ou qu'on vous a déjà proposé, voilà. Oui, oui.**

**L :** Proposé, oui. J'ai reçu cette direct', cette, mais c'était que des numéros de téléphone, en fait… Ça m'énerve. Ça veut dire que je suis vieille quand je reçois des trucs comme ça. Ah elles tiennent plus ces boucles d'oreilles. Tant pis, elle est tombée. J'enlève l'autre…

**C : Donc ouais de, de quoi vous auriez besoin aujourd'hui ?**

**L :** La santé, pis la jeunesse *(rires)*, et puis. De rien, en fait. Non, je crois que si je réfléchis j'ai, j'ai ce qu'il faut… Les yeux.

**C : Les yeux ?**

**L :** Voilà, oui. Une greffe, avec des nouveaux yeux *(rires)*. Ça oui, ça me plairait bien. J'ai été très, très, très, très bien toute ma vie, mais vraiment à un point. Et puis quand j'ai eu la cataracte, j'ai mon ophtalmo qui m'a dit : « écoutez, je vais vous opérer de la cataracte, mais est-ce que vous accepteriez que je vous opère de la myopie en même temps ? Mais j'enlève pas complètement ». J'ai dit : « oui, mais moi j'ai pas les sous, pis mon assurance, elle va pas payer ». Il me dit : « oui, mais, ça passera dans le ». Alors il m'a opéré la myopie. J'ai mis une année avant de m'habituer de pas avoir des gros « r » comme ça. Et puis après, je me suis habituée. Deux ans après, j'ai eu la dégénérescence maculaire. C'est pas de chance, hein *(rires)*.

**C : Ouais *(rires)*. Donc plutôt.**

**L :** Non, les yeux.

**C : Plutôt les yeux autrement pour.**

**L :** Y a que les yeux que j'aimerais. Une greffe. *(En s'adressant au chat) :* « tu arrêtes de manger de l'herbe, tu vas dégueuler. Eh ! ». Tu vois hein, ils ont besoin d'herbe les chats, quand ils sortent pas. *(En s'adressant au chat) :* « t'es coincée, hein ». Voilà.

**C : Donc voilà. Si je résume un peu, par rapport à la maladie pulmonaire, à la BPCO, pas de besoins particuliers, à part si quelqu'un pouvait venir, comme vous disiez, vous enlever, voilà la, la maladie, mais.**

**L :** Oui, c'est ça. Ou me rendre mes yeux. Ouais, ouais. Non, non.

**C : Mais, voilà. Comme c'est quelque chose dont on peut pas guérir, [comme vous disiez, qui est évolutif.**

**L :** Ben oui, qu'est-ce que vous voulez que je fasse,] c'est évolutif. En fait, depuis deux ans, trois ans, c'est stabilisé hein. Je vais même, je vais même mieux qu'y a trois, quatre ans. Parce qu'y a trois, quatre ans je, je souffrais vraiment. J'avais des crises de, de, même y a cinq ans j'en ai fait aussi au, des crises de, détresse respiratoire, vraiment ! Maintenant j'ai plus ça !…

**C : Donc là c'est assez [stabilisé votre maladie.**

**L :** Mais j'ai quand même] eu des difficultés, parce que je soufflais un peu. Mais en fait, je peux aller chercher mon tram, je peux faire des choses quand même, j'ai pas. Je suis descendue à mon cours, et tout... Mais, j'ai l'impression que c'est plutôt meilleur qu'y a quatre, cinq ans… Cet été c'était formidable ! Et puis tout d'un coup ça redescend. Alors là c'est, c'est dans la tête. C'est angoissant.

**C : C'est angoissant ? Que ça fluctue ?**

**L :** Ouais, ouais, c'est ça. xxx, l'hiver qui vient, et tout, et tout, et là j'ai peur de nouveau, parce que c'est toujours au mois de novembre ou au mois de janvier que je suis à l'hôpital.

**C : L'hiver c'est angoissant [comme période ?**

**L :** Ouais, ouais, ouais, ouais.] Ouais. Pis là, depuis 15 jours, j'ai la bronchite de nouveau, mais ça, je suis tellement habituée à avoir la bronchite. Mais j'ai toujours toussé toute ma vie, alors ça change pas grand-chose, hein... Pis ça se situe là hein *(elle se touche au niveau de la gorge)*, ça descend pas sur les poumons, pour l'instant.

**C : [Tout reste au niveau.**

**L :** Ouais, ouais, ouais.] Parce que ça descend du nez. Ça descend là. J'ai pas ces grosses toux de, pour l'instant hein, je tousse un peu, mais pas, ça va.

**C : Ouais, donc c'est aussi des périodes où l'hiver c'est [un peu plus difficile.**

**L :** Ouais, je déteste.] Là, faudrait que je m'endorme maintenant. Pis je me réveille au mois de mars. Comme les marmottes *(rires)*.

**C : *(rires)*.**

L : Je sais pas y en a encore d'autres qui font ça… Je sais pas si les écureuils ils doivent faire ça, avec leur approvisionnement *(rires)*.

**C : *(rires)*. Et, quel aurait peut-être été pour vous l'utilité d'une prise en charge, ils disaient globale spécialisée, où justement on a quelqu'un qui vient une fois par mois, évaluer les besoins.**

**L :** Mais quels besoins ?

**C : Les, en terme de gestion des symptômes physiques. Peut-être des besoins au niveau des symptômes psychologiques, du soutien spirituel, si y a besoin, voilà. Tous les différents endroits où on pourrait amener du soutien.**

L : Je sais pas.

**C : En termes [d'informations.**

**L :** Rien,] ça va bien. Pis moi j'ai pas tellement de volonté. Ça veut dire que je, y a une petite salle de gymnastique à côté, pis j'ai mon programme de X *(hôpital, pneumologie)*. Et puis c'est à cinq minutes, sept minutes. Il faudrait que j'aille, pis j'y aille trois fois par semaine, le matin. Comme ça je suis pas obligée d'aller marcher une heure l'après-midi. Mais faut y aller *(rires)*.

**C : Mmh. [Une question de.**

**L :** Et je passe souvent devant,] parce que quand je vais faire mes grands tours, je dis : « mince, faudrait que j'y aille ! » Et puis, c'est la volonté qui me manque, en fait. Mais ça j'ai tout le temps, je me suis inscrit à pleins de trucs. J'en ai fait hein des salles, *(rires)* pendant ma vie. Mais j'ai jamais été au-delà de deux mois, trois mois. Mais là c'était déjà bien. Tandis que là, j'aimerais bien pouvoir y arriver deux, trois fois par semaine.

**C : C'est un peu la, [la volonté.**

**L :** Mais il me faut la volonté,] voilà c'est ça ! Je vais y aller une fois, je vais être contente, et pis après une deuxième fois, pis après : « oh il pleut ! Oh il fait froid ! Et tout, je reste au lit ». *(rires)*.

**C : *(rires)*. Donc pour aussi ce type de, [de prise en charge ?**

**L :** Ouais, non, c'est ça.] La volonté, personne va me la donner, hein. C'est moi qui doit gérer ça. C'est tout !

**C : Ça aurait été peut-être un peu trop d'avoir une fois par mois quelqu'un ?**

**L :** *(soupir).* Je sais pas à quoi ça peut, qu'est-ce que ça peut aider, pour moi. C'est-à-dire, non, moi je vois juste, je vois me parler toujours de cette maladie, je l'ai, je connais, et tout. Ça m'intéresse pas que quelqu'un vienne m'en parler tout le temps : « comment vous allez ? Qu'est-ce que vous faites ? » Et tout. Si vraiment, je vais pas bien, là je vais voir avec mon pneumologue, mais. Lui, il trouve ça va *(rires)*. Il m'a vu très bas, là il trouve que c'est pas mal, et puis, et puis, je sais pas, il veut pas me voir tous les mois ! Là, tous les trois mois, il trouve ça va assez, pour moi.

**C : Donc, pas besoin de plus de suivi, [ou de ?**

**L :** Ben non,] on peut pas faire grand-chose. C'est à moi de faire hein, surtout, c'est vrai. Faudrait que je balaye dans ma tête les angoisses, mais ça, je vis avec des angoisses depuis toujours. Donc, je voudrais balayer ça, et puis, et puis, et puis me forcer, à sortir !

**C : Mais là, pour, pour par exemple, ces angoisses, ce serait plutôt vous qui aimeriez faire quelque chose ?**

**L :** Oui, mais qu'est-ce que vous voulez faire contre les angoisses ? Moi j'ai eu des angoisses toute, j'ai toujours eu des angoisses, j'ai des angoisses tout le temps. Même mon fils qui a 56 ans, quand il part faire du parapente, j'ai le noeud là *(rires)*. Mais je suis toujours comme ça. Je lui ai bouffé la vie, je crois. C'est pour ça qu'il est comme ça avec moi *(rires)*. Les angoisses.

**C : Vous avez encore un peu, ouais, ces, ces angoisses, mais, ouais.**

**L :** Ouais, j'ai toujours. Hier soir, je vous ai dit, mais alors j'ai, après j'ai bien dormi. J'ai été faire mon sac... Pis je me suis plantée devant l'armoire, en disant : « qu'est-ce que j'vais prendre ? » Parce que j'aime pas ces chemises : « je prends ça, je prends ça ». Pis j'ai dit : « mais je suis débile, moi ! A deux heures du matin, *(rires)* à me poser la question qu'est-ce que je vais prendre pour être le mieux possible, et tout ça ». J'ai dit : « je suis complètement débile ! » Mais ça, ça, l'angoisse est tombée là. J'ai été me coucher, pis c'était de nouveau bien, j'ai dormi.

**C : Vous avez donc préparé votre sac, comme pour aller à l'hôpital, c'est ça ?**

**L :** Oui, c'est ça !

**C : Et, pis après ça, ça vous a calmé ?**

**L :** Ouais, ouais.

**C : Vous étiez moins angoissée.**

**L :** Je me suis dit : « mais ça va pas dans la tête ! » *(rires)*. Non, non, et j'avais déjà préparé mon sac, comme j'y vais assez souvent. Et c'est les slips, les trucs comme ça. Alors j'ai *(rires)*.

**C : *(rires)* Vous avez préparé.**

**L :** Je me suis dit mais que je suis complètement folle ! *(rires)*.

**C : *(rires)*. Donc voilà, vous avez pas de, pas de besoins particuliers. Vous aimeriez, peut-être avoir un peu moins [ces angoisses, ou mieux.**

**L :** Ben évidemment !] Mais je crois que, je vis avec hein, depuis toujours hein. Pis tout d'un coup j'ai plus. Pis le soir, ben, je prends un petit truc, un demi mach', comprimé pour dormir, parce que c'est le soir.

**C : Ouais. Donc c'est plus, c'est plus ça, les angoisses.**

**L :** Ouais, ouais c'est tout !

**C : Et pis la vue [qui vous pèse, un peu, voilà ?**

**L :** Ouais, ouais, ouais, ouais, c'est ça qui me pèse le plus.]

**C : Ouais. [Et autrement, par rapport.**

**L :** Bon hier,] quand j'ai marché, pis que je m'essoufflais, ça m'a ennuyé, en fait. J'ai dit : « merde, ça recommence ! » Parce que j'étais tellement bien ces temps, j'allais chez le dentiste à X, j'allais à pied, et tout. Et là, non *(rires)*, je fais pas la moitié du trajet. Alors ça, ça m'ennuie.

**C : Oui. [Ça vous embête de plus pouvoir.**

**L :** Voilà.] Pis après quand je suis là, j'écoute la radio, j'y pense plus...

**C : Ouais, c'est, de pas pouvoir faire [certaines activités.**

**L :** Ouais.] Non pis, j'ai des petites, des bonheurs quand même, je crois que c'est l'addition des petits bonheurs, qui fait une bonne journée, en fait. Là c'est, tout à l'heure, je me suis cuit des patates douces, j'adore les patates douces ! J'ai fait en purée, j'ai mangé ça avec des oeufs, parce que je mange pas de viande. Et, ce sera un petit bonheur super ! Je pense déjà aux biscuits que je vais manger après *(rires)*. J'adore ça *(rires)* ! Alors ça c'est un bonheur, le matin le petit-déjeuner, et tout.

**C : Ouais. [Donc y a l'addition de ces petits bonheurs.**

**L :** Mais je crois que c'est ça qu'il faut, ça.] Ouais, ouais, c'est ça. Pis entre-deux, tout d'un coup, je plonge, alors j'ouvre ma truc, et pis je fais un jeu débile, qui me plaît bien *(rires)*. Pis voilà.

**C : Ouais. Donc vous avez, même si ça fluctue, vous avez ces petits bonheurs.**

**L :** Ouais, ouais, ouais, ouais.

**C : Ouais, ouais. Donc voilà, je sais pas si, si y avait autre chose que vous aimeriez rajouter ?**

**L :** Non, non.

**C : Par rapport à, voilà, à cette étude-là, par rapport à [vos besoins, par rapport à ?**

**L :** Non, non, non,] non, non.

**C : A la, à la BPCO. Je crois que.**

**L :** Non, mais ça va, hein !

**C : Ouais. Y avait pas, voilà, comme vous avez dit, vous aviez pas d'attentes particulières en participant à cette étude.**

**L :** Non.

**C : C'était plus [pour la recherche.**

**L :** Plus, ouais,] la recherche, voilà. Ben oui, y a pas de raisons de pas participer à ça. C'est comme quand y a des, des émissions sur les maladies orphelines et tout ça, il faut participer, merde ! C'est pas pour nous, c'est pour les enfants d'après ! C'est important !

**C : C'est ça, ouais. Donc c'est ça, vous aviez pas d'attentes par rapport [à vous-même, mais c'était.**

**L :** A moi, non, non, non, non,] c'est.

**C : Plus pour la recherche, et.**

**L :** Oui, voilà.

**C : Et voilà. Donc vous.**

**L :** J'étais à l'hôpital, y a quatre, cinq ans, avec une dame qui habite pas loin, qui habite à X. Et, qui était plus jeune que moi, mais c'était pire ! En plus elle avait une sonde gastrique, et tout ça. Et puis, mais elle fumait comme un pompier. Et, elle était bien plus jeune. Et je me dis : « bon ben ça va encore ! J'ai pas de sonde gastrique ». Bon, il m'en ont mis une quand j'étais aux soins intensifs, mais je m'en suis même pas aperçue. Je m'en suis aperçue quand ils l'ont retirée. Mais, ça va. *(En s'adressant au chat) :* « non on arrête avec l'herbe ! ».

**C : *(rires)*. Donc voilà, pis par rapport à, au traitement, vous êtes plutôt, ben satisfaite [avec votre traitement.**

**L :** Ben oui, ben oui !]

**C : Vous prenez [vos médicaments régulièrement.**

**L :** Voilà, c'est ça, ouais.] Alors je me demandais justement, mais je vais voir avec l'ophtalmologue, si c'est pas un médicament spécifique, c'est celui que je prends le plus, qui me file ces allergies. Parce que, à l'hôpital, ils me le donnaient pas. Ils ont pas à l'hôpital ! Il fallait que je remonte le mien de la maison, parce qu'ils ont pas ce médicament à l'hôpital, mais là j'avais pas de boutons, j'avais pas d'allergie. Alors je me demande si c'est pas ça. J'ai changé de couette, j'ai changé, changé les draps, j'ai tout fait, passé l'aspirateur, en disant c'est des puces de lit et tout ça. Mais c'est pas vrai, parce que hier après-midi j'étais pas là, et pis ça me grattait partout. Et je me demande, c'est peut-être un médicament.

**C : C'est juste peut-être un effet.**

L : Et pis un antiallergique qui m'a donné, ça provoque des urticaires, c'est con ! *(rires)*. Il donne un antiallergique pendant cinq jours, et ça provoque des urticaires *(rires)*.

**C : Ouais *(rires)*. Donc voilà, quelques effets peut-être [secondaires de.**

**L :** Secondaires,] c'est con hein *(rires)*.

**C : [De certains médicaments, qui sont embêtants.**

**L :** Alors xxx, je regarde] jamais la posologie, qu'est-ce que ça peut provoquer, je vais regarder ça. Mais à l'hôpital, j'avais pas, hein ! Mais je prenais pas ce médicament. Donc peut-être, faudrait que j'aille voir un allergologue. Mais ça aussi, il faut se pousser pour téléphoner, tout ça. Moi je fais rien de tout ça. Mon fils m'a dit qu'il avait fait une coloscopie, et tout. Moi j'ai dit : « j'ai jamais fait moi ». *(rires)*.

**C : Ouais *(rires)*. Donc pas de.**

**L :** Moi je refuse de m'occuper de moi comme ça *(rires)*.

**C : Oui *(rires)*. Donc oui, et pis, vous m'aviez dit aussi, pas de, pas besoin d'informations supplémentaires, voilà.**

**L :** Ben non, parce que ça m'intéresse pas, en fait. Je vais pas revenir en arrière. Si j'avais 30 ans, alors là vraiment, je m'informerais. Mais, non, 78 ans, non… Je sais même pas si j'ai envie de vivre jusqu'à 90 ans. A moins d'être en forme, de bien marcher, et tout, et tout. Moi, avec la respiration, j'imagine. Et pis là, je vais avoir la canne blanche, alors... J'ai toujours dit : « le rollateur, pour mettre la bonbonne, plus la canne blanche, non ». Mais je suis sûre que j'arriverais à vivre avec ça, hein.

**C : Mais pas, pour l'instant pas.**

**L :** Pour l'instant, xxx. Justement, plus d'informations.

**C : Ouais. Donc assez, voilà. Et pas de… pas de, d'autres besoins particuliers ?**

**L :** Non, non.

**C : Ouais. Merci beaucoup en tout cas d'avoir pris le temps. Je sais pas si vous avez, vous voulez encore [rajouter quelque chose, par rapport à ça ?**

**L :** *(soupir)* Non, rien, non, non, non, non, non,] non, non

**C : Par rapport à, à vos besoins ?**

**L :** *(En s'adressant au chat)* : « mais t'arrêtes un peu ! »

**C : *(rires)*. Voilà, je pense qu'on peut.**

**L :** Vous avez un chat ? Non ?

**C : Non, non. J'en ai eu beaucoup. Mais, mais là, non, comme je suis dans un appartement. Le jour, peut-être, où, où je pourrais avoir une maison, quelque chose *(rires)*.**

**L :** Ah oui, bien sûr ! Bon elle, j'ai un grillage sur la fenêtre, parce que je peux pas vivre les fenêtres fermées.

# Participant n°4

| |
|---|
| Nom d'emprunt : Sylvianne |
| Âge : 68 ans |
| Sexe : F |
| Groupe : Intervention |

**C : Vous avez donc participé à une étude qui avait pour objectif de comparer le traitement habituel de votre maladie, la BPCO, avec une prise en charge précoce, soutenue et intégrée. Vous étiez dans le groupe bénéficiant de cette prise en charge globale spécialisée et vous avez reçu la visite d'une infirmière une fois par mois pendant un an. Pouvez-vous me raconter comment ça s'est passé ?**

S : Faut dire que c'est de l'histoire ancienne presque. Très bien, très très bien. Non ça s'est très bien passé. On a eu, j'ai eu un très bon contact avec les deux personnes qui sont venues. Et non tout a, tout était bien. On parlait de beaucoup de choses, on parlait de la vie en générale, de de pfff de beau' beaucoup de choses. Et c'était des moments un peu privilégié je dois dire. Parce que je vis <u>seule</u> alors comme ça y a eu euh, on peut <u>communiquer</u> quoi, c'était très bien. Oh non bien.

**C : [Pour vous ça s'est bien passé.**

S : Non, non, bien. Ouais]. Ouais pis moi bon de toute façon, j'aime bien savoir exactement ce que j'ai, où j'en suis et tout, donc ça me pr' ça me permettait de poser pas mal de questions aussi. Alors voilà.

**C : Donc voilà vous avez au moins pu [avoir ces moments.**

S : Ah ouais non] j'ai eu un très bon contact avec les deux personnes et pis non, pis je pense que le ressenti était bien. Très bien.

**C : Vous avez pu avoir, voilà ces moments un peu [privilégiés.**

S : Oui, oui absolument], tout à fait. Ouais, ouais. Non c'était bien.

**C : Et poser des questions sur.**

S : Poser, bon des questions, oui. Oui on pouvait poser des questions, et pis des d'… Oui avoir des renseignements sur beaucoup de choses, pis pis, non c'était bien. Très bien.

**C : Ca, vous avez apprécié d'avoir ces informations.**

S : <u>Vraiment</u>. Ouais, bien apprécié. Pis toutes sortes de questions, toutes sortes de, pis pour beaucoup de choses. Bon sur la maladie, je pense, maintenant je pense que je suis quand même bien au point parce que j'ai quand même pas mal de, pis j'aime bien savoir, donc je me renseigne toujours. Quand quand, à l'hôpital ou comme ça, quand on me fait quelque chose, pourquoi on me le fait, ce que j'ai exactement et tout. Et tout le monde m'a toujours bien bien répondu clairement et tout et je sais vraiment où j'en suis dans ma maladie et puis bon ben voilà quoi. Et pis si j'ai des quest', si j'avais des questions, je profitais de ces moments justement avec euh avec ces personnes qui venaient pour euh pour euh pour les poser hein. Alors voilà *(sourire)*. Non c'était bien. Très bien *(sourire).*

**C : Donc là vous avez reçu suffisamment d'informations, pu poser vos questions.**

S : Ouais. Tout bien. Tout bien, tout bien.

**C : Tout bien. Et vous pouviez aussi parler d'autre chose vous disiez, de.**

S : Ah ouais absolument, on parlait de tout. De la pluie et du beau temps, de de de, d'un tas de choses. De l'environnement, de la famille, des, des non, tout bien. Non c'était bien, vraiment très très bien.

**C : Un bon contact vous disiez.**

S : Un très bon contact ouais. Pis ces personnes sont super bien formées je trouve, parce que… Très ouvertes, très, non très bien. Vraiment…

**C : Peut-être, qu'est-ce que vous attendiez en participant à cette étude ?**

S : Oh moi j'attendais rien du tout. J'étais d'accord de participer, je voulais voir ce que c'était, pour moi c'était un peu, c'était une inconnue quoi. Je savais pas de quoi il, bon on m'avait plus ou moins dit que ça concernait la BPCO, ça oui tout à fait. Mais euh mais autrement euh, bon ben je savais pas ce qu'on allait me poser comme questions, à quoi ça servait. Mais… ben voilà quoi, c'était bien. C'était bien. Moi j'attendais rien de spécial, mais je dois dire que j'ai beaucoup apprécié *(rires)*.

**C : *(rires)*. Donc voilà pas, vous attendiez rien de spécial, mais vous avez apprécié.**

S : Absolument. Tout à fait.

**C : D'accord. Et mmh peut-être si on regarde plus particulièrement, qu'est-ce qui vous a plus ou déplu dans les visites [de l'infirmière une fois par mois ?**

S : Y a rien qui m'a déplu]. Y a rien qui m'a déplu. Y a qu'une ch', peut-être qu'une chose, c'est, on m'avait donné, donc quand je suis sortie de l'hôpital, j'avais déjà eu, j'avais vu *Claire (infirmière de recherche)* justement à l'hôpital, avant que je sorte de l'hôpital, qui m'avait, c'est elle qui m'avait contacté justement pour, pour cette fameuse étude. Et puis elle m'avait donné tout de suite un un un un petit feuillet, un prospectus pour les directives anticipées… Et pis j'ai dit : « bon ok, d'accord, ça je vais regarder ». Et plusieurs fois quand, chaque fois qu'elle revenait euh là c'était *Sophie (infirmière de recherche)* ouais, chaque dois qu'elle venait, elle me disait : « et pis les directives anticipées ? », je lui disait : « on en parlera plus tard ! » et pis maintenait c'est toujours : « on en parlera plus tard » ! Donc voilà *(rires).*

**C : *(rires)* donc ça elle vous a, voilà, [parlé de.**

S : Oui, oui] elle m'a parlé. Pis bon j'ai toujours la brochure ici, je sais exactement de quoi il en est, de quoi. Mais quand je serais, quand je serais d'attaque, ben quand je serais d'aplomb, quand je serais, dans ma tête peut-être posée différemment, et ben oui je le ferais *(rires)*.

**C : Mais c'était pas pour tout de suite.**

S : Non, non c'était pas pour tout de suite, non *(raclement de gorge)*. Pas pour tout de suite. J'avais pas envie de parler de ça, j'avais pas, non, non.

**C : Donc ça ça vous a un peu plus déplu, si y avait [quelque chose.**

S : Non] pas le fait que ça m'a déplu, non. Je sais, non non, absolument pas, mais, mais elle revenait chaque fois là dessus et moi chaque fois je disais : « non, pas maintenant » et elle disait : « ok » et voilà *(rires)*.

**C : *(rires).***

S : Non, non, mais c'est pas quelque chose qui m'a, qui m'a déplu. Absolument pas…

**C : Mais c'était quelque chose pas pour maintenant, voilà.**

S : Ah toute façon ! C'est toujours pas pour maintenant *(rires)*.

**C : *(rires).***

S : Et pis si jamais, j'en parlerais avec ma, avec mon, avec mon médecin traitant, avec ma pneumologue. Donc le, donc le moment venu. Alors voilà…

**C : Et voilà vous m'avez parlé un peu des des informations que vous avez reçues, quelles informations supplémentaires auriez-vous aimé avoir ?**

S : Aucune. J'ai tout eu… Je m'y connais pas suffisamment pour demander, pour avoir des informations supplémentaires. Je pense que toutes les informations que j', que j'ai eu, non c'est tout bon quoi…

**C : Donc tout bon au niveau des informations, oui…**

S : Non là franchement, je vois pas, je vois pas. Euh non... A part qu'elles me parlent de quelque chose que je ne connais pas, à ce moment là je dirais : « bon ben d'accord expliquez-moi, j'ai envie de savoir ! », mais comme ça, non quoi. Je peux pas, informations complémentaires, je p' je pense qu'elles m'ont donné toutes les informations que moi je voulais, elles mes les, elles ont pu me, elles ont pu répondre à mes informations et pis elles m'ont donné des informations aussi que voilà quoi. Non, non.

**C : Donc pas de besoins [d'informations supplémentaires.**

S : Non, non. Absolument pas].

**C : Et… aussi au niveau des, de la gestion des symptômes physiques, comment les visites de l'infirmière et les informations que vous avez reçues vous ont-elles aidé ou non à gérer les symptômes [de la BPCO.**

S : Ben] ça je dirais que non, ben c'est que pour les informations, ouais xxx de toute façon on répète toujours la même chose hein, c'est toujours les mêmes choses. Et pis moi ça fait quand même pas mal d'années que je suis, que j'ai ce problème donc euh le ressenti de la personne fait tout quoi, au moment. Parce que tous les jours changent. Un jour on se lève on est bien, le lendemain on se lève, on est moins bien. Donc on, on se, c'est le ressenti qui fait que voilà, qu'on… Parce que ce jour là, je suis moins bien et ben je fais moins de choses que quand ben voilà. On fait un peu au jour le jour. Par contre, y a toujours une chose moi qui me dérange énormément, c'est que depuis que je suis sous oxygène, je suis une personne qui faisait les choses : « ah ben tiens, j'ai 5-10 minutes, ben tiens je pourrais vite faire ça » pis de partir pour aller acheter ça, pour aller faire ci. Et maintenant, je peux plus réagir comme ça parce que chaque fois il faut que je, il faut que je remplisse ma petite bonbonne et tout ça ben j'ai, du coup ça me, j'ai plus envie de faire ça quoi… Voyez ce que je veux dire.

**C : Oui, oui.**

S : Du coup j'ai plus envie de partir ! Donc voilà, c'est un peu, c'est un peu ça qui est, qui est, le fait qu'on soit sous oxygène ça c'est, ça c'est un peu plus embêtant… Pour moi du moins parce que, c'est les genres de choses on doit tout prévoir. Et moi prévoir les choses, je sais pas. J'aime bien le faire : « Et hop ! » sur le moment *(rires)*.

**C : *(rires)*. Donc ouais là ça.**

S : Ca c'est vrai que c'est un truc, ça « pfiou », y a des fois je me dit : « non, mais c'est pas vrai » bon… Alors voilà, souvent le matin je remplis, parce que j'ai deux bonbonnes, j'en ai une sur un petit chariot, qui est un peu plus grande que l'autre, que je mets sur le *(toux),* que je porte sur le dos. Donc des fois le matin je remplis mes deux petites bon', mes deux bonbonnes, pis je me dit : « voilà, comme ça voilà c'est prêt ». Malheureusement les bonbonnes elles se vident même si on les utilise pas ! Donc *(rires)*.

**C : *(rires)*. Donc ouais ça c'est un peu plus [embêtant pour.**

S : Ouais, ouais c'est embêtant !] C'est emb', ça je dois dire que vraiment c'est embêtant. Mais bon, heureusement qu'on a ça, on peut faire des choses qu'on pourrait pas faire si on avait pas l'oxygène hein. Ca c'est vrai que… on a encore de la chance dans notre malheur… Alors voilà.

**C : De faire d'autres choses maintenant que vous avez aussi l'oxygène.**

S : Ah oui, oui. Bon c'est clair que je peux plus faire tout ce que je voudrais faire quoi, comme les marches à pied, tout ça, *(soupir)*. C'est… ça va un petit bout quoi, ça va un petit moment, mais… Y a beaucoup de choses que je fais <u>plus</u>, que je faisais avant. Style simplement d'aller me balader, parce que moi d'aller me balader pour m'arrêter tous les dix mètres *(soupir)* pour respirer, non. Pour moi c'est pas de la ballade, c'est… C'est plus contraignant que, j'ai pas de plaisir, j'ai plus de plaisir à aller me balader comme ça, donc heureusement que je conduis. Ca ça va bien. Ca ça me, ça me permet de pouvoir faire mes courses déjà. Et pis voilà. Pis autrement je fais de la pétanque ! Je peux plus faire de la montagne, je peux plus faire de ski, je peux plus faire beaucoup de choses, alors je me suis dit : « faut que je trouve quelque chose », alors je fais de la pétanque !

**C : [Donc là vous pouvez faire de la pétanque.**

S : Là toutes les semaines] je vais jouer de la pétanque, parfois la semaine, parfois le week-end également et pis voilà quoi, c'est chouette, c'est sympa. Pis on se trouve entre amis, entre, je fais partie d'une amicale, donc c'est agréable. Pis ça permet de voir du monde aussi. *(Toux)*. Non c'est bien.

**C : C'est chouette de pouvoir faire de [la pétanque, de voir du monde.**

S : De pouvoir faire quelque chose ! *(Rires)*] Simplement. C'est tombé sur la pétanque parce que ça me c', ça me convient tout à fait quoi. Avec l'oxygène, j'ai mon petit, ma petite bonbonne sur mon chariot, je vais d'un côté, de l'autre du terrain avec ma petite bonbonne *(rires).* Pis tout se passe bien ! Tout se passe bien. Alors voilà *(sourire).*

**C : Vous disiez que vous étiez seule et que justement ces moments de pétanque ou.**

S : Ah c'est privilégié, ça c'est sûr ! C'est vrai, ça j'apprécie beaucoup ! J'apprécie beaucoup. On va une fois par semaine, tous les jeudis soir. Et puis, bon jusqu'au mois d'octobre, là c'est fini jusqu'au mois d'avril. Mais on peut toujours aller jouer euh au comment, au X *(quartier)* là. Alors voilà… Mais c'est bien.

**C : Donc voilà vous avez ces moments là.**

S : Mais ouais, c'est des bons, c'est des bons moments !... Alors voilà. Mais des fois on a des sorties du' d'une journée et là, avec la pétanque, là il faut que je prenne <u>mes deux bonbonnes</u>, donc la petite sur le dos plus mon petit chariot avec l'autre bonbonne, parce que autrement je tiens pas toute la journée quoi. Parce que quatre litres euh, quatre litres euh, la journée c'est quatre litres. Quand je suis au repos, je suis sur un litre, la nuit un litre. La journée quand je vais et viens comme ça c'est quatre litres et quand je marche c'est six litres, donc avec six litres je tiens une heure et demi de temps quoi avec la petite bonbonne. Donc voilà quoi…

**C : Donc là vous devez prendre.**

S : Alors je prends les deux et pis une fois que une et est vide, je laisse de côté pis je fais un trafic *(rires)*. Pis dès que je suis assise, ben je l'arrête. Parfois je l'arrête même complétement, parce que pour essayer de la tenir le plus longtemps possible *(rires)*. C'est un micmac *(rires).*

**C : *(Rires).* [C'est une organisation.**

S : Enfin voilà, ouais !] Mais bon voilà *(rires).* Non, non, mais c'est *(rires).* Enfin c'est vrai, mieux vaut en rire quoi.

**C : Vous disiez aussi que vous appréciez ces visites à domicile parce que cela faisait de [la visite.**

S : Oui voilà], ça faisait de la visite. Une personne qui sort de, de l'extérieur et pis et pis qui me parle de tout à fait autre chose et ça aussi c'est agréable quoi. C'est vrai que c'est bien. C'est bien…

**C : Si on regarde un peu avec les proches, quel soutien avez-vous reçu de la part de vos proches ?**

S : De mes proches, de ma famille ? *(Raclement de gorge)*. Oh non j'étais bien entourée. J'ai donc, j'ai deux fils, j'en ai un qui habite dans le X *(région de Suisse)*, malheureusement il est, enfin malheureusement, non, c'est formidable d'habiter dans le X *(région de Suisse)*, mais bon c'est vrai qu'il est plus loin quoi. Qui a un petit garçon de huit ans. Et puis, qui est autiste malheureusement, oui malheureusement, mais enfin il est chouquinet. Et puis euh puis j'ai un autre fils ici qui habite X *(ville de Suisse)*, qui vient d'avoir un petit bout là de d'une semaine alors oui c'était, lui il est, il ferait tout pour moi quoi. Du reste il me dit : « si t'es pas bien, tu me téléphones. Si je réponds pas, tu me téléphones plusieurs fois de suite comme ça je sais qu'il y a quelque chose, qu'il y a urgence » et puis voilà quoi. Mais, non non, bien. Je les vois, il vient, il vient me trouver régulièrement, enfin s'il vient pas me trouver, il m'envoie des longs messages et puis on a X *(messagerie instantanée)* avec les enfants et tout donc. C'est bien, on peut, c'est bien, c'est chouette. Non non c'est très bien.

**C : [Votre fils.**

S : J'ai mon frère] aussi. Mon frère et ma belle-sœur aussi qui m'entourent bien également. Alors voilà.

**C : Donc vous avez voilà [votre fils.**

S : Non, non, non, c'est bien]. Ca va bien. Tout se passe bien avec la famille. Parfait !

**C : Du soutien de la part de de vos proches.**

S : Ouais !... Pis les amis aussi, également.

**C : Les amis aussi.**

S : Ouais, ouais. J'ai une amie qui m'appelle pour ainsi tous les deux jours, tous les jours. Elle me dit : « comment ça va ? » pis moi aussi je l'appelle parce qu'elle aussi elle est pas en super forme. Mais *(rires)* alors voilà quoi.

**C : *(Rires).* Donc voilà du soutien des.**

S : « Alors voilà tu réponds, c'est que t'es bien ! » *(rires).*

**C : *(Rires).* Donc voilà [des amis aussi.**

S : Oui oui tout à fait].

**C : [Votre frère, votre belle-sœur.**

S : Ouais, non, non, c'est bien]. Non ça c'est vrai que, c'est vrai que c'est bien appréciable quoi.

**C : Donc vous appréciez [ce soutien.**

S : Oui absolument]. Et puis ils me disent : « si t'as besoin de quoi que ce soit, tu m'appelles ou ». Non y a, pis y a plusieurs personnes également. Et mon voisin, mes voisins également, à côté. Ils m'ont dit : « si y a quoi que ce soit, si un jour t'es pas bien à la salle de bain, tu tapes fort contre le, contre le *(rires)* contre le mur » ; « oui oui d'accord » *(rires)*. Et puis il a les clés également de chez moi donc si y avait quoi que ce soit. Ben voilà quoi.

**C : Au cas où, aussi les voisins qui sont là.**

S : Et pis j'ai le voisin à côté *(rires)* qui est là. Non c'est vrai que c'est appréciable. On est pas, on est, on est chacun chez soi, mais dès le moment où il y a un problème, ben voilà on fait ce qu'il faut quoi… Alors voilà. On est pas toujours les uns chez les autres, mais euh, mais si y a un problème, je sais que je peux compter, que je peux compter sur lui aussi.

**C : Les voisins.**

S : Absolument. Des fois je vais faire des courses et puis c'est un peu lourd à porter. Il me dit : « mais dis-moi ! Je vais vider ta voiture ! ». Pis moi j'aime bien faire les choses par moi-même aussi. Donc je descends la voiture, c'est un peu ma cave la voiture, le coffre de la voiture. Et pis je monte de temps en temps chercher ce qu'il faut *(rires)*. On s'arrange, mais ouais *(rires)*, mais bon c'est sûr. Enfin voilà.

**C : Donc vous avez du soutien de la [part des voisins.**

S : Oui absolument] du soutien de la part des voisins, de la part des amis, de la part de la famille. Non c'est bien.

**C : Et comment ça s'est déroulé ces visites de l'infirmière pour vos proches ? Quand elle est venue une fois par mois ?**

S : Pour mes proches, alors je leur en ai même pas parlé !... Savez on parle pas, non je leur en ai même pas parlé… Ben peut-être à *Luc*, mon fils qui habite ici. Je lui ai dit : « J'ai une infirmière », oui peut-être que je lui ai dit. Mais euh, mais je lui ai pas, je lui ai pas, je lui ai pas, je lui faisais pas de compte-rendu, rien du tout quoi *(rires)*. Alors non *(rires).* Non, non, ça c'est, non, non c'est…

**C : Pas.**

S : Ben non…

**C : Pas au courant…**

S : Oui au courant de ma maladie, ils savent exactement ce que j'ai, tous les deux. Ils savent très bien que c'est une chose qui va pas en s'améliorant, mais qui peut être stabilisé. Le mieux qu'on peut faire c'est de stabiliser. Et puis et puis voilà. Mais euh mais non mais non ils savaient, non… Non pis je veux pas, mais oui ils sont au courant, pis je veux pas chaque fois remettre ça, non. Ils savent ce que j'ai et puis voilà, on va pas chaque fois en parler et… Marteler en tête et… Non… Pis moi j'aime pas ça.…

**C : Vous aimez pas trop en parler.**

S : Non. Non *(rires)*.

**C : *(rires).***

S : Quand je vais bien, tout vas bien ! Voilà ! Non, non *(soupir)*.

**C : Pas trop parler, voilà de la maladie, [de.**

S : Mais non] ! Mais non, écoutez, c'est, c'est, il fait beau, le jour. Non, non, c'est bien. Pis non c'est vrai j'aime pas en parler… Même avec mes amis ou comme ça, non. On se voit, c'est des moments de plaisir ! Avec mes enfants aussi quand on se voit c'est pour le plaisir, c'est pas… Non, non non. Pis non pis si ils voient bien des fois que je suis essoufflée et tout et c'est le pr', ces les premiers à dire : « écoute reposes-toi, prends ton temps, on a le temps, on est pas pressé ou » et pis voilà mais. Mais autrement, à part ça, on en parle pas quoi. Non pis moi je veux pas. Je veux pas que ça vienne, non non non. Pis vous savez tout le monde a ses problèmes donc on va pas encore leur mettre, marteler en tête là dessus quoi…

**C : Pas envie d'en reparler.**

S : Non j'aime pas ressasser quoi… Voilà. J'ai déjà passé des mauvais moments quand j'étais à l'hôpital parce qu'ils se sont fait beaucoup de soucis, donc on va pas encore leur créer du souci parce que j'ai ça quoi ! Chaque fois revenir la dessus, ils savent et pis voilà. Si je vais pas bien, ils sont là, ils sont là pour pour pour me soutenir, pour faire ce qu'il faut, mais mais on va pas toujours en parler quoi, non ! Non moi je suis pas d'accord ! Ca fonctionne pas comme ça. Non.

**C : Vous avez pas envie, comme vous disiez, de créer du souci.**

S : Voilà ! Je veux pas créer de l'ang', du souci quoi. Et pis du souci ils s'en font certainement hein, de toute façon. Mais euh je veux pas, non ! Non, non.

**C : Pas trop en parler.**

S : Non. Non. Si ça va pas bien, et ben ils sav', je sais que je peux compter sur eux. Mais euh mais quand moi je me sens bien, euh y a pas de raisons qu'on qu'on reparle de ça et pis qu'on retombe dans cette maladie, machin, « gna gna gna ». Non ! Pourquoi ? Ca servirait à quoi ? Ca servirait à rien du tout ! Alors voilà…

**C : Mmh si on repense à ces visites, quelle a été pour vous l'utilité des visites de l'infirmière une fois par mois ?…**

S : L'utilité ?

**C : Oui, pour vous ?... Justement une fois par mois.**

S : Pour ma maladie ?

**C : Pour vous, de manière générale.**

S : Ben c'est comme je viens de vous dire, c'était le fait d'avoir quelqu'un qui venait de l'extérieur et pis que je puisse communiquer. Pis si jamais j'avais une question à leur poser pour un médicament ou pour ou pour euh ben voilà elles étaient là et je pouvais leur poser la question, elles me répondaient. Mais, mais moi c'était surtout le fait de de pouvoir converser un moment avec une personne qui qui voilà converser de ma maladie avec une personne qui savait exactement ce que j'ai. Et ça, ça c'est voilà. Ca c'était bien.

**C : D'avoir.**

S : Pis on en revenait toujours aux mêmes choses hein. C'était… les questions elles étaient là, elles me répondaient et pis voilà quoi ! On… Non non c'était bien. Et puis *Sophie (infirmière de recherche)* elle me faisait à chaque fois les massages du dos. Ca c'était très agréable ! *(rires)*.

**C : *(rires).* Ca.**

S : On prenait des bons moments ouais. Oh c'est vrai que c'était bien. *(rires)*. C'était bien.

**C : D'avoir quelqu'un qui, pouvoir parler avec quelqu'un qui connaît bien et.**

S : Oui quelqu'un qui me connaît, oui ! Quelqu'un qui connaît bien. Et puis non non c'était bien. Vraiment bien. Des bons moments. Des bons moments de rigolade aussi en même temps. Ouais. Non c'est vrai *(rires)*.

**C : *(rires).* Et les massages en plus.**

S : Et les massages. Et les massages, je finissais toujours par un massage. « Oh lala » ça c'était génial. Ouais. En fin de compte, maintenant j'ai beaucoup moins mal au dos. Ca fait un moment qu'elle est pas venue pourtant. Ca fait bientôt une année non ?

**C : Oui ça doit faire, ça doit faire à peu près une année que.**

S : Ca va faire une année qu'elle a arrêté hein ? Ouais voilà. Ouais c'est sûr. Ouais, ouais. Alors voilà.

**C : Ouais, ouais. Et mmh de quoi auriez besoin aujourd'hui ?**

S : Rien.

**C : Rien.**

S : Oui ! J'aurais besoin d'avoir plus d'air *(rires).* Ca c'est un grand besoin *(sourire).* Mais autrement euh… non par rapport à ce qu', à ce que je vis maintenant avec avec mon, avec mon ma ma avec ma BPCO et puis… Ce que j'aurais besoin ?... Non, tout va bien.

**C : Rien de voilà, de l'air si vous pouviez voilà en avoir en plus *(sourire).***

S : Ouais, ça ça serait bien. Mais malheureusement… ben j'ai mes béquilles là maintenant, ça va très bien ça.

**C : Vous avez des béquilles ?**

S : Non, je dis mes béquilles, c'est mes deux bonbonnes *(rires).*

**C : Ah ! *(rires).***

S : Pour moi c'est des béquilles. Mais non autrement je v', je sais pas. Oh ben c'est sûr, ce que j'aurais besoin c'est, euh *(soupir)*… Je vois pas, non… Non, je vois pas à part euh, non…

**C : Rien de spécial qui vous vient voilà à.**

S : Qu'est-ce que je pourrais avoir besoin ? J'ai besoin de rien moi ! Moi j'ai tout ! *(rires).*

**C : *(rires).***

S : Comme ça, assise sur une chaise, on a tout ! Dès le moment où je me lève et pis je vais monter les escaliers, je vais vous dire : « oui j'aurais peut-être besoin d'avoir des jambes un peu plus musclées, d'avoir des » *(rires).* Mais du reste il faut que je recommence le, le réentraînement à l'effort parce que j'ai arrêté là ça fait bientôt, ça fait un bon moment déjà. Pis j'ai pas repris parce que, parce que j'étais ess' plus sous oxygène et il fallait que je me parque au parking de X et du parking X pour aller jusqu'à l'hôpital, ça fait quand même un bout à pied. Pour aller ça va, parce que c'est en descente. Mais pour remonter après après une heure d'effort à l'hôpital euh vélo, tapis et tout, <u>refaire</u> le bout en sens inverse *(soupir)*… Ca c'est un peu euh… C'est un peu embêtant. Et puis aller en bus, ben mon problème c'est d'aller d'ici au tram. Pis après d'aller prendre la le tram jusqu'aux X et pis après de prendre un autre petit bus qu'il y en a tous les trois-quarts d'heure je crois. Non, non, c'est ça qui est un peu embêtant c'est que, c'est que pour des personnes qui sont, qui sont, qu'ont besoin d'oxygène, et bien euh c'est un peu embêtant. Et pis bon j'avais peut-être pas forcément non plus envie. Parce que avant j'y allais régulièrement, mais j'avais moins besoin d'oxygène aussi. C'est depuis qu'ils ont passé à quatre litres la journée que, que là c'est…

**C : C'est un peu plus [embêtant.**

S : C'est un peu plus] embêtant ouais. Ou je me suis mise ça dans la tête que c'était plus embêtant et que j'y arriverais peut-être très bien. Je sais pas. Le fait que, faut que j'aille, faut que je téléphone à xxx, pis que je reprenne rendez-vous et pis voilà. Pis on verra comment on fait. Parce que, c'est vrai comme là j'étais à la maternité dernièrement, deux-trois fois, ben j'ai à chaque fois pris le taxi hein. Parce que pour aller jusqu'à là-bas aussi. Vous parquez où ? Nouveau parking X ? Pis pour aller jusqu'à la maternité c'est encore plus loin que l'hôpital *(rires)*… *(Soupir)* donc j'ai à chaque fois pris le taxi, mais bon si faut à chaque fois, deux fois par semaine il faut prendre le taxi aller-retour, ça fait 30-60, ça fait quand même 120 francs par semaine hein. Donc euh.

**C : C'est coûteux.**

S : Ca devient coûteux ! Ouais. Ca devient coûteux les entraînements *(rires).* Les réentraînements à l'effort *(rires).*

**C : Donc voilà c'est des réentraînements à l'effort auxquels vous alliez avant.**

S : Oh oui ça fait des années que je vais là-bas, ouais. Ca fait des années que j'avais commencé ça et puis euh, et puis j'ai toujours fait et puis là ça fait plusieurs mois que j'ai arrêté. Et puis là il faut que je reprenne… Enfin on verra. On va faire ça peut-être pour euh, 2017 ? On verra.

**C : C'est peut-être quelque chose que vous ferez.**

S : Certainement. Même peut-être avant. Je sais pas. On verra.

**C : Vous verrez…**

S : Mais c'est dans ma tête, de toute façon il faut que je le fasse. Parce que c'est vrai que ça me faisait beaucoup beaucoup de bien ça aussi…

**C : L'entraînement, ces entraînements.**

S : Oui ces entraînements, ouais. Réentraînement à l'effort. Donc c'est un groupe, le Dr A. *(pneumologie)*, qui avait, qui avait mis ça sur pied je crois. Ca c'est bien.

**C : Ca ça vous fait, ça vous faisait du bien ?**

S : Eh ben oui parce que ça me remettait, ça me remettait un peu de masse musculaire. C'est vrai quand on fait plus rien comme ça, les muscles y fondent hein. Pis c'est pas bon quoi. C'est pas bon…

**C : Donc ça vous verrez pour 2017.**

S : Ouais peut-être avant ! Je sais pas. Peut-être que tout d'un coup, un jour je vais prendre le téléphone, je vais appeler pis là : « ouah, youpi ! » *(rires), «* j'ai envie de recommencer, est-ce que y a une petite place pour moi ? ». Non mais je devrais déjà les appeler maintenant parce que souvent ces, ces cours c'est quand même des cours qui sont assez prisés, je crois que les gens demandent facilement. Et puis euh. Donc il faudrait quand même que je les appelle. Ouais ça faut que je mette à l'ordre du jour, tiens.

**C : Dans la liste *(rires)*.**

S : Non parce qu'une fois que j'ai recommencé, après c'est bon quoi. Une fois qu'on est dans le circuit, après c'est tout bon. On sait que, c'est soit une fois par semaine, soit deux fois par semaine quand on peut le faire, pendant 3 mois et puis après 27 semaines exactement je crois… Et puis euh après c'est une fois par semaine pour entretenir… Voilà. Enfin on verra bien ça !

**C : Vous verrez bien. Mmh je sais aussi que parfois on peut recevoir du, du soutien au niveau spirituel. C'est peut être quelque chose qu'elles ont fait pendant les visites.**

S : Alors non. Non parce que, non. Peut-être qu'on avait parlé et puis non. On en a peut-être parlé, oui c'est possible. Mais bon c'est peut-être certainement moi qui avait pas forcément envie, je crois. Ouais, je crois que c'est ça.

**C : Ca vous aviez pas forcément [envie de.**

S : Non j'ai] pas forcément envie, non… Non…

**C : Donc ça vous en aviez peut-être parlé, mais pas voilà.**

S : Oui, on avait effleuré et je crois que c'est moi qui a un peu, qui a pas voulu. Je pense… Qu'on qu'on qu'on parle de ce, qu'on parle de… De cette approche spirituelle, je pense, je sais pas. Je m'en souviens plus. Je m'en souviens plus.

**C : Pas forcément envie…**

S : Non. Pis bon. Non…

**C : Et parfois aussi, elles apportent du soutien psychologique, qu'est-ce que vous pouvez m'en dire là dessus ?... Lors des visites…. Ça arrive…**

S : Oui bon ben y a des fois, on a le moral un peu parterre et pis c'est vrai que, elles, non elles écoutaient plus qu'autre chose quand j', quand on avait, quand j'avais des problèmes. J'ai eu des problèmes, bon d'… Non mais je peux pas dire que… Non. Du point de vue psychologique, oh y a des hauts et des bas quoi. Mais, mais y a rien de spécial. Pis avec elles, je me souviens pas, non je peux pas dire qu'on a beaucoup parlé de ça… Parce que moi dans ma tête, j'étais plus ou moins bien quoi…

**C : Elles vous écoutaient.**

S : Ouais elles écoutaient, c'est tout. Peut-être qu'il y a des jours où j'ai, c'est arrivé mais je pense que c'était avec *Julie (infirmière de recherche)* une fois... Je m'étais mis à pleurer, je sais plus pourquoi *(elle chuchote)*… Oh y avait eu quelque chose, mais ça devait pas être tellement important en fin de compte parce que je m'en, parce que je m'en souviendrai… Je m'en souviendrai. Mais là je m'en souviens plus du tout. Je m'étais mis à pleurer… Pis plus elle parlait et plus je pleurais. Pis elle trouvait ça très drôle *(rires)*.

**C : *(rires).***

S : *(rires)*. C'était un jour comme ça. Un jour peut-être, un jour sans peut-être. Un jour de brouillard ou je sais pas quoi.

**C : Parfois on a moins le moral.**

S : Oui pis bon elles venaient le matin, à 10h donc, donc y a des fois des matins où on a le ressenti qui est différent. Le matin et l'après-midi ça peut être différent quoi. Mauvaise, mauvaise journée, pas bien, enfin je sais pas. Je sais plus. Ou y avait peut-être eu quelque chose avec, je sais pas… Je sais plus… Oui je sais *(elle tape sur la table)*. Parce que j'avais ma belle-fille qui a eu deux cancers. Un cancer du sein et puis, c'était quand on avait parlé de *Nicolas*, justement mon fils qui habite dans le X *(région de Suisse)*, qui est marié et qui a ce petit garçon autiste. Et ils, ma belle-fille a eu un cancer du sein en 2008, en 2012… et la même année ils ont appris que le petit était autiste… 2011 ou en 2012, je sais plus… En 2012 je crois. Et pis après, eh ben je crois que c'est l'année dernière qu'elle avait fait cette récidive. Cancer et ça s'était, ça c'est propagé dans les os et dans le foie. Ca a été très bien et tout d'un coup « paf » de nouveau. Et je crois que c'est là, un jour qui, je venais d'apprendre ça et puis elle est arrivée et puis la j'ai « pfff » j'ai vidé mon sac quoi. J'avais besoin, j'avais besoin de pleurer. J' toute seule comme ça, je pleure pas souvent, mais dès le moment où on en parle, « hop » ça y'est. Ca, ça m'a fait beaucoup de bien du reste. Et puis maintenant ma belle-fille elle va super bien de nouveau. Elle a pu s'en sortir et tout et puis. Non mais là je me disais, mais c'est pas vrai, avec tout ce qu'ils ont, c'est pas possible quoi. Et pis maintenant, maintenant c'est mon fils qui est, qui est à l'assurance, il a des problèmes de dos, il a deux hernies discales, dont une hernie discale qui lui coince le, le nerf sciatique et il est carreleur donc il peut plus faire son métier de carreleur. Il adorait son métier. Maintenant y faut qu'il fasse, qu'il se recycle, qu'il refasse un apprentissage à 38 ans, qu'il refasse un apprentissage pour un autre métier. Ah j'ai dit : « mais c'est pas vrai ! Ca va s'arrêter quand chez eux là-bas dans le X *(région de Suisse)*? ». Ils ont vraiment eu que des problèmes quoi. Et ça je, « pfiou » y a des jours bon comme aujourd'hui ça va, mais y a des jours je me dis : « non c'est pas possible. C'est pas juste quoi ! ».

**C : [Y a des jours avec et des jours sans.**

S : Y a vraiment des jours, avec ouais !] Pis je me dis que le malheur s'acharne comme ça sur, sur des pers', toujours sur les mêmes personnes, non c'est pas normal quoi ! Enfin voilà. Je pense que ça devait être, ah oui pis c'était à cette période là. Donc voilà donc avec ça on peut en parler, c'est vrai, de nos problèmes personnels, on pouvait en parler. Et pis elles étaient, elle était à l'écoute quoi, c'est ça.

**C : Ouais. Ca vous avait fait du bien [de vider votre sac comme vous disiez.**

S : Ah oui, absolument ! Tout à fait]. Ouais vider son sac. Et c'est vrai que toute seule comme ça, on… Moi je me mets pas, non je me mets pas facilement à pleurer toute seule dans mon coin quoi. Y a des moments on en parle, on gratouille un tout petit peu et pis « hop » y a tout qui part quoi *(rires).*

**C : *(rires).* Oui.**

S : Aïe, aïe, aïe. Non, non, mais c'est voilà quoi. Non mais autrement, voilà ! C'est vrai que, ben voyez avec elles je pouvais parler de ça. Ca c'était très bien. C'était un, c'était un, un problème que j'avais et que… et pis voilà quoi. Je peux en parler, c'était parfait…

**C : Pouvoir parler de ses problèmes personnels ou d'autres choses.**

S : Oui parce que si on en a, on a gros sur le cœur pour quelque chose et ben voilà, et ben j'ai dit : « ben voilà ce qu'il se passe et tout » pis, pis voilà.

**C : Donc ça vous aviez apprécié.**

S : Ouais, c'est vrai ! Bon c'était, ce jour là c'était ça. Peut-être qu'un autre jour j'avais eu autre chose, j'aurais peut-être parlé d'autre chose, mais voilà. Mais bon elle était à l'écoute…

**C : Voilà. Il y avait pas mal de choses que vous aviez apprécié.**

S : Ah non mais j'ai tout apprécié ! Franchement j'ai tout apprécié. Ca c'est vrai. Ouais.

**C : Vous me disiez autant l'écoute, pouvoir poser des [questions.**

S : Y a l'écoute], y a pouvoir poser des questions, et puis puis elles étaient vraiment, elles étaient vraiment, elles étaient juste dans leurs façons de répondre, enfin c'est ce que j'attendais quoi ! La façon dont elles répondaient, elles répondaient vraiment comme comme je l'attendais. Pas vaguement, pas euh si on voulait poser des questions un peu plus pointues, ben elles répondaient quoi, c'est ça qui était bien. Ouais, non vraiment bien. J'ai trouvé vraiment très très bien. Vraiment.

**C : Pouvoir avoir des personnes, aussi vous disiez, qui connaissaient bien [la maladie, pouvoir voilà.**

S : Oui… Ouais absolument].

**C : Et si ça tombait sur un jour sans aussi, pouvoir parler d'autre chose**

S : De pouvoir parler d'autre chose. Absolument. Tout à fait. Ouais, ouais. Non non, c'était bien…

**C : L'écoute donc euh… Voilà vous m'avez dit déjà pas mal de choses, je sais pas s'il y a autre chose que vous aimeriez rajouter.**

S : Non.

**C : Par rapport à ça, par rapport à ce que vous auriez souhaité recevoir en plus ou en moins lors de ces visites.**

S : Non. Peut-être avoir oui quand vous aurez le, euh le résultat de cette euh le résultat de euh

**C : De l'étude ?**

S : De l'étude là, ouais, une fois que ça sera. Ça ça serait pas mal d'avoir…

**C : Savoir un peu.**

S : Ouais d'avoir connaissance de ce que ça a pu apporté quoi. Ce que ça peut, ouais ce que ça peut apporter surtout c'est ça. C'est bien joli de faire des études, mais faut voir aussi le, le hein ce que ça apporter au patient quoi.

**C : Oui… Qu'est-ce qu'on peut en faire ?**

S : Ce qu'on peut en faire. Ouais, ce qu'on peut en faire, oui tout à fait. Ce qu'on peut en faire, c'est ça… Ouais tout à fait…

**C : En tout cas quelque chose que vous avez bien apprécié et vous voyez pas vraiment des choses qui vous ont déplu [ou d'autres choses que vous auriez besoin aujourd'hui.**

S : Non. Vraiment pas].

**C : Ou que vous auriez voulu différemment ou… Voilà.**
S : Non…

**C : Voilà donc c'était quelque chose d'utile pour vous et… et que vous avez apprécié.**

S : Ouais ut', oui utile je pense. C'est vrai que si ça avait pas été fait, si y avait pas eu ça, ben voilà y aurait pas eu ça. Mais je pense que c'était utile, ouais… Pour moi c'était un plus ouais. C'était un plus… Parce que, non c'était un plus je pense…

**C : C'était un.**

S : Un plus…

**C : D'avoir ces visites… une fois par mois… Mmh je crois qu'on a fait un peu le tour. Il y avait juste voilà, vous m'aviez dit, cette histoire un peu des directives anticipées où là voilà vous auriez peut-être.**

S : Peut-être plus tard, mais je regarderais ça avec ma, le jour où j'ai envie de le faire, j'en parlerais avec ma pneumologue… Pis voilà. Pis peut-être mon fils aussi, qui habite ici sur, à X *(ville de Suisse)*. Peut-être qu'à ce moment là, voilà. Mais autrement… Non.

**C : Autrement, voilà à part ça vous avez.**

S : Pour l'instant, j'ai pas envie… *(rires).*

**C : *(rires).* Mais autrement voilà de manière générale en tout cas c'est quelque chose que vous avez apprécié**

S : Ouais.

**C : Et que voilà vous ne voyez pas vraiment d'autres chose à changer [et.**

S : Non]. Ah non je vois pas d'autres choses à changer ! Vous savez tout ce qui vient de l'extérieur euh ben moi je trouve bien quoi… Enfin peut-être pas tout, mais là la façon dont ça a été, ça a été amené, entrepris et tout, j'ai bien, j'ai bien apprécié. Mais par contre ce qui v', quelque chose d'autre je vois pas quoi… Je sais pas… Qu'est-ce qu'on pourrait bien avoir d'autre ? Non je sais pas…

**C : En tout cas… Oui…**

S : Non non, c'est tout bien ça. C'est tout bien. Est-ce que ceci ça va dans le, c'est ça, non est-ce que ça sera, ça, non ça sera pas disponible dans mon dossier médical hein ?

**C : L'entretien ?**

S : Ouais, non pas l'entretien, le résultat et tout ça.

**C : Alors il faudrait que je me renseigne pour, après je sais pas exactement comment vont être transmis les résultats ou informés mais je sais que en tout cas tout ce qui était par rapport à votre participation, voilà ça a dû resté dans votre dossier médical, par rapport à qu'est-ce qui**

**a été fait, une fois par mois. Ca je sais que c'est inscrit quelque part. Après par rapport au résultat global de l'étude, je pense qu'il y aura une communication, après à voir.**

S : Ah ouais à voir si jamais ça apparaît dans mon do', parce que justement je suis connectée avec mon dossier médical et ça je trouve pas mal du tout. Parce que pour ma pneumologue déjà ou pour quand je vais, comme j'ai fait pas mal de, j'ai fait pas ma d'aller-retour à l'hôpital là en 2015. Ouais 2014-2015. On est quoi ? En 2016 ?

**C : 2016.**

S : Ouais 2014, fin 2014 et début 2015, ouais. Et bien euh…

**C : Là vous avez ce dossier médical.**

S : Ouais, j'ai, c'est tout dans mon dossier médical quoi. Ca fait que je peux, moi je peux aller voir sur internet dans mon dossier médical « machin point gnan». Je me branche dessus, ils me donnent mon, ils me donnent mon code et « hop » je peux aller voir ce qui, je peux aller voir exactement ce qu'il y a. Et je me demandais justement si ces résultats ils seraient disponibles là dedans, mais peut-être pas ça.

**C : Il me semble que ça sera pas dans le dossier médical les résultats de l'étude, mais après.**

S : Ouais parce que c'est les résultats de l'étude en général.

**C : C'est ça.**

S : C'est pas personnalisé hein.

**C : Non, non.**

S :Tout à fait, parce que y a pas de nom, c'est impersonnel.

**C : Oui exactement.**

S : Tout à fait.

**C : Donc là il ne devrait pas y a voir.**

S : D'accord.

**C : Mais ça en tout cas, si vous avez aussi des autres questions vous pouvez tout le temps contacter, voilà vous avez le contact du *Docteur C. (soins palliatifs)*.**

S : *(En prenant le courrier qui lui avait été adressé sur l'étude).* Voilà *Docteur C. (soins palliatifs)* ouais.

**C : Si vous aimeriez avoir plus d'informations. Pour l'instant c'est en train d'être analysé. Mais voilà.**

S : Non non, mais là y a pas de soucis. Franchement je ne vois pas ce que j'aimerais… ce que j'aimerais en plus à part de l'air… Autrement non…

**C : Autre chose que vous aimeriez encore rajouter ou ?**

S : Non !

**C : Non.**

S : Vraiment je vois pas…

**C : Merci en tout cas d'avoir pris le temps [de répondre et partager votre expérience.**

S : Mais écoutez c'est nor', mais écoutez c'est normal ! ] Le ressenti.

**C : C'est important.**

# Participant n°5

| |
|---|
| Nom d'emprunt : Marc |
| Âge : 79 ans |
| Sexe : M |
| Groupe : Intervention |

**C : Vous avez participé à une étude qui avait pour objectif de comparer le traitement habituel de votre maladie pulmonaire, la BPCO, avec une prise en charge précoce, soutenue et intégrée. Et vous étiez dans le groupe bénéficiant de cette prise en charge globale, spécialisée. Et vous avez reçu la visite d'une infirmière une fois par mois pendant un an. Pouvez-vous me raconter comment cela s'est passé ?**

**M :** Comment s'est passé ?! Il est venu, il m'a posé les questions, pis j'ai répondu à les questions qu'il m'a posées et pis voilà !... C'est tout comment s'est passé *(rires)*, comment s'est passé. Ils ont, ils m'ont fait beaucoup de demandes et pis j'ai répondu à les demandes qu'il m'a fait ! Mais ça allait, y a des jours ça va mieux, des jours y a pire, voilà…

**C : Donc y avait pas mal de demandes, lors de ces visites, et vous répondiez à ces demandes ?**

**M :** Ouais. Il me posait les questions sur le, j'ai répondu à les questions qu'il m'a posé ! Voilà.

**C : Ouais, oui. Et, qu'est-ce que vous attendiez en participant à cette étude ?**

**M :** Qu'est-ce que tu veux que j'attends *(rires)*, j'attends rien ! Il paraît que la maladie ça, ça va pas guérir. Alors, je la garde comme ça, qu'est-ce que tu veux. xxx je peux, quand ça arrive le moment, ça arrive le moment ! Qu'est-ce que tu veux faire, c'est comme ça...

**C : Vous attendiez pas quelque chose de spécial ?**

**M :** Qu'est-ce que, qu'est-ce que vous voulez que j'attende de spécial *(rires)* ? Maintenant, j'ai presque 80 ans, alors… Je, je xxx mes jours, quoi… C'est dommage, parce que, je peux pas aller dans une place, parce qu'il faut ça *(il tire sur le tuyau de la bonbonne d'oxygène)*, il faut... Obligé rester dans le quartier, quoi, pas aller trop loin, juste ça… Maintenant, j'allais à la physio à l'hôpital, maintenant il veut plus, parce que, paraît il faut aller, ouais, trois fois par semaine, moi trois fois par semaine, c'est un petit peu chargé, quoi, faire le tapis, le vélo, musculature. Là, c'était bien parce que c'était tout près. Qu'est-ce que tu veux faire.

**C : Avant c'était tout près ?**

**M :** Quand j'allais à l'hôpital, c'était un étage, j'allais faire la physio, et tout ça. Maintenant, ils ont mis trois fois par semaine, alors je sais pas, trois fois par semaine, c'est trop pour moi. Des fois, j'arrive, mais des fois j'arrive pas parce que tu comprends il manque le souffle. Il me vient les crises, parce que si je vais à, maintenant je, je suis à, je vais à, à X *(quartier)*, X *(rue)*, deux fois par semaine, mais ces jours j'ai pas été, parce que comme je suis grippé. Alors j'avais un petit peu la peine, ça fait un petit peu loin. S'il m'arrive quelque chose, xxx à l'hôpital, c'était tout près. Là, s'il m'arrive une crise, c'est différent. Là-bas.

**C : C'est plus loin.**

**M :** Plus loin, pis aller, il faut prendre le bus, après il faut reprendre le bus, il faut aller X *(quartier)*, revenir ici. xxx m'arranger deux fois par semaine là-bas, ça va. Je vais retourner là-bas, je vais voir l'année prochaine quoi, si je suis encore en vie...

**C : Et, et quand vous avez eu ces infirmières qui venaient une fois ?**

**M :** Ben j'allais là, j'allais là, juste à, le mois passé j'allais toujours à l'hôpital. Maintenant, ils ont changé, il paraît que il faut trois fois par semaine. Alors, le, le médecin m'a fait une ordonnance,

d'aller à la physio, pis ils ont dit y avait pas de place, et pis maintenant il faut trois fois par semaine, il faut pas deux fois. Alors, alors le machin pulmonaire où j'avais l'habitude, là-bas. Elle m'a dit d'aller là-bas. Elle a téléphoné elle-même, elle a fait tout le nécessaire, et pis j'ai été là-bas... Maintenant que j'ai la grippe, ça a pas été, ça fait trois, trois fois que je vais pas, deux semaines que je vais pas.

**C : Et quand vous aviez ces infirmières qui étaient venues une fois par mois pendant un an, [à la maison.**

**M :** Ben j'allais toujours à l'hôpital.].

**Femme de M :** *(La femme de M. rentre dans la pièce)* Mais non, tu te souviens pas quand elle venait ici une fois par mois te poser des question, avoir des, pris des dispositions aussi au cas où, si tu voulais quelqu'un à l'hôpital, tu te souviens pas de ces choses là ?

**M :** Mais quelqu'un à l'hôpital, comment quelqu'un à l'hôpital ?

**Femme de M :** Mais pour les questions de le prêtre, ou bien.

**M :** Oh le prêtre *(rires)*.

**Femme de M :** Tu te souviens ? Ben ça, c'est ça ! Que t'as signé aussi, c'est, c'est.

**M :** Ah oui, signé comment tu voulais mourir, et tout ça.

**Femme de M :** Voilà. Eh ben, c'était, c'était ça ! C'était *Valérie* qui venait, c'était qui, comment, elle s'appelait comment ? Ou *Valérie* ou *Marie, Marie*.

**C : Y avait *Laure Chappuis*, *Julie Mottier* et *Claire Favre (infirmières de recherche)*.**

**Femme de M :** Une fois il est, alors c'était qui, c'était le médecin *Marie* ?

**C : C'est possible.**

**Femme de M :** C'est peut-être, c'était le médecin, oui c'est ça, oui. Tu te souviens pas quand y avait *Laure (infirmière de recherche)* qui venait tout le mois ?

**M :** Ouais, elle est venue, oui, je me rappelle. Bon : « comment tu veux mourir ?». Moi je *(grommellement)*.

**Femme de M :** Tu te rappelles de ça maintenant ? *(rires)*. xxx Elle t'a posé beaucoup, beaucoup de questions, chaque fois y avait des questions xxx.

**M :** Elle a la liste.

**Femme de M :** Voilà, voilà. Alors.

**M :** Elle a la liste.

**Femme de M :** Si t'as besoin de comprendre quelque chose, si je me rappelle, je.

**M :** Elle me posait des questions.

**Femme de M :** Eh ben réponds.

**M :** Les questions qu'elle me pose, je réponds

**Femme de M :** Si t'as été sincère, tu réponds les mêmes questions ! Si t'as dit des mensonges, tu dis des choses différentes ! Et comme je te connais depuis 50 ans, et tu sais bien comment ça se passe, alors je me mêle plus *(elle repart dans l'autre pièce)*.

**C : *(rires)*. Donc voilà, si on revient.**

**M :** Mais je me rappelle plus, qu'est-ce que tu veux !

**Femme de M :** *(Elle répond depuis la pièce à côté)* C'est pas que tu te rappelles pas, c'est question, c'est, c'est question de, de, de, de sincérité !

**M :** Elle m'a dit comment je voulais mourir, et tout ça. Bon, mourir, je meurs à l'hôpital ou à la maison, je sais pas. S'il me vient une attaque là, je meurs là. Si j'arrive à aller à l'hôpital, ben, mais je veux pas aller à, je voulais pas aller à X (*hôpital*) pour mourir là-bas.

**C : Donc vous avez pu discuter un peu de ça, de qu'est-ce que vous vouliez ? *(la femme de Marc revient dans la pièce).***

**M :** De ce que je voulais, comme j'habite en ville, il paraît c'est la ville qui s'en occupe, alors, il fait le nécessaire.

**Femme de M :** Mais c'était pas sa question, c'était l'assistance à l'hôpital.

**M :** Ben, l'assistance de l'hôpital.

**Femme de M :** Quand vient le moment, si tu voulais un curé, ou bien quelqu'un d'autre qui s'occupe de ta spiritualité, c'est ça, c'est ça que xxx. Mais c'était pas ça l'essentiel, y a eu beaucoup, beaucoup, beaucoup de questions, je sais. Mais à chaque fois, t'en avais marre *(rires)*.

**M :** Moi je sais pas, questions, [xxx questions, je me souviens plus, moi.

**Femme de M :** Parce que là y avait, y avait,] ça s'appelle comment, *Nicole (infirmière)*, celle qui vient tous les trois mois, qui t'avait proposé ça, si tu voulais participer à cette étude. Tu l'as fait, maintenant, c'est la suite *(elle repart dans l'autre pièce)*.

**C : Donc voilà, si on, si on revient justement à cette, à ces visites une fois par mois, voilà, y avait des questions qui avaient été posées, vous avez pu discuter aussi.**

**M :** Ouais. Ben, j'ai répondu à des questions, c'est tout marqué les questions qu'elle a ! C'est elle qui a marqué tous les questions, comment, elle m'a demandé tout. Je me rappelle toutes ces questions. Bon, je me rappelle : « comment est-ce que vous voulez mourir ? » Tout ça. Elle avait tout marqué, là, je sais pas, si je voulais le curé, je voulais ça. J'ai dit : « bon ». Le curé, je sais pas.

**C : Donc vous avez pu parler un peu de ce que vous désiriez ?**

**M :** Oui, je sais pas qu'est-ce qu'il faut faire, si je, si je vais brûler, ou bien mettre… xxx, je sais pas…

**C : Oui. Je sais que, parfois aussi elles parlent de ce qu'on aimerait comme soins aussi en, en fin de vie.**

**M :** Ben, les soins en fin de vie, si ça va pas, elle m'a dit : « entubé », si ça va pas, qu'est-ce que tu veux entuber ?! Tu veux rester comme ça là, mieux vaut partir et pis terminé hein. Faut pas rester, xxx si ça va pas, faire une piqûre, pis terminé. xxx souffrir…

**C : Donc vous, vous avez pu parler de ça avec les infirmières et ?**

**M :** On a parlé de ça, oui, mais il dit, les piqûres, non, ça va pas. *(rires)* xxx quand il arrive la mort, il arrive la mort, qu'est-ce que tu veux faire. C'est sûr quand t'es très mal, c'est sûr le souffle ça va pas, maintenant c'est le souffle ça va pas, c'est l'asthme, c'est les poumons quoi, ils sont foutus ! Qu'est-ce que tu veux faire, on peut rien faire ! On peut pas changer les poumons *(rires)*. Penser avant ça… C'est la peinture, tout ça, mets le masque, mais personne lui donnait le masque, alors tu veux faire quoi. C'était comme ça, c'est pas que la, un petit peu la fumée, les cigarettes, un petit peu tous les produits qu'ils ont servi, et voilà c'est, petit à petit, c'est usé.

**C : Usé les poumons.**

**M :** Usé les poumons et tout. C'est comme tous, tous celles qui ont fait ponçage de parquet, là ils en ont tous, tous comme ça ! Celles qui ont fait, je crois la peinture à la voiture, la même chose ! Carrossiers, les peintres, tous comme ça ! Je connaissais beaucoup de peintres par-là, ils sont tous passés à la casserole, hein, 65 ans... Avant c'était comme ça. Mais maintenant, c'est la peinture à la flotte, c'est différent, mais à cette époque là...

**C : Et, vous avez parlé aussi de, d'un prêtre, est-ce que y a eu au niveau d'un soutien spirituel ?**

**M :** Moi je suis catholique, mais qu'est-ce que tu veux. Ma femme, elle est Témoin de Jéhovaz… Mon fils, il est protestant, il va jamais à l'église comme moi, quoi *(rires)*…

**C : Qu'est-ce que vous pouvez me dire sur le, le soutien spirituel qui a été apporté lors de ces visites avec les infirmières ? Vous avez discuté ?**

**M :** Ben on a discuté un petit peu, mais maintenant, je me rappelle pas les demandes. Mais elle avait dit comment je veux mourir, soit que je vais sous terre quoi, je vais pas me brûler, je suis catholique...

**C : Vous avez pu exprimer ce que vous désiriez ?**

**M :** Ouais, comme ça, ma femme, si elle veut pas brûler, alors, si on met à côté, xxx, je sais pas...

**C : Et mmh qu'est-ce qui vous a plu ou déplu dans les visites de l'infirmière qui venait une fois par mois, pendant un an ?**

**M :** Ben, elle venait, elle posait les questions, moi je répondais à ses, à la question qu'elle me posait : « oui ; non » ! Comment, la santé comment ça allait, elle posait beaucoup de questions ! Mais maintenant, je me rappelle pas les questions qu'elle me posait !...

**C : Donc, qu'est-ce que vous auriez souhaité peut-être recevoir en plus ou en moins pendant ces visites ?**

**M :** Moi *(rires),* plus ? Qu'est-ce qu'il peut faire ? Il demande, il pouvait rien faire *(rires)*. Qu'est-ce que vous voulez me faire *(rires)* ! Elle voulait rien faire ! Je demande, bon. Quand j'arrive les crises, je peux pas faire tout seul le bain, maintenant j'arrive encore à faire le bain tout seul, quoi. Seul, mais, ma femme qui veut pas, je fais toujours quand elle est là. Pour le moment, j'arrive, mais quand j'ai les crises qui arrivent, quand il me prend, j'arrive pas. Quand ça bloque, ben quand ça bloque, après, pour le moment ça va...

**C : Autrement vous avez votre femme qui est là pour vous aider pour les soins ?**

**M :** *(Grommellement)* un petit peu… Pour le moment, mais après. Bon y a l'infirmière qui vient me préparer tous les médicaments… *(il se lève et cherche le carnet de santé où c'est marqué).* C'est marqué là, xxx par semaine.

**C : C'est noté dans le carnet de santé ?**

**M :** Ouais. xxx par semaine xxx…

**C : Voilà, juste par rapport à cette gestion des, des symptômes physiques, donc vous avez ce carnet de santé, avec les médicaments à prendre ?**

**M :** Parce que ouais, l'autre fois quand xxx deux, trois ans en arrière, il m'avait donné même quelqu'un de venir faire la douche, deux fois, trois fois par semaine. Pis après, bon, ça allait mieux, parce que je xxx les crises, ça va. Tout d'un coup. Maintenant, bon, j'essaie de pas xxx quand j'ai fini, mais ça va, ça va.

**C : Voilà. Donc vous avez les, les infirmières qui viennent vous aider de temps en temps ?**

**M :** Non, non, non personne qui vient, il vient juste pour préparer les médicaments, pis regarder la tension, ça. La douche, je sais faire tout seul, tout seul, avec ma femme.

**C : Donc vous avez, en terme de soutien de vos proches, vous avez votre femme qui est là, vous avez.**

**M :** Bon, elle est pas toujours là, parce qu'elle va aussi chez mon fils. Mais, qu'est-ce que tu veux, elle est aussi âgée, hein… On fait qu'est-ce qu'on peut.

**C : Vous avez votre fils aussi.**

**M :** Mon fils il a trois gamins, il est tout seul aussi… Il a trois gosses, 12, 13 ans, il va au cycle, ils ont commencé le cycle cette année, cette année, la fille, L'autre, ça fait la deuxième année qu'il va au cycle...

**C : Et, comment ça se passait justement ces visites une fois par mois des infirmières pour vos proches, quand elles sont venues ?**

**M :** Quand elles sont venues, elles ont posé les questions, elles ont marqué, et tout, je sais pas. Elles ont marqué, et pis c'est bon. On a discuté, elles m'ont posé les demandes, que moi je répondais à ses demandes qu'ils faisaient !

**C : Et vos proches étaient présents aussi pendant ces visites ?**

**M :** Ouais des fois ma femme, oui elle était là aussi.

**C : Comment ça se passait ?**

**M :** Elle avait tout marqué, vous avez le dossier, alors.

**Femme de M :** xxx, une fois quand elle est venue la Doctoresse, y avait aussi notre autre fille.

**M :** Aussi ma fille, oui.

**C : Aussi une autre fille qui venait.**

**M :** Non, ma fille aussi, elle est venue quand.

**C : Ah, votre fille venait aussi de temps en temps ?**

**M :** Elle est venue quand y avait la Doctoresse, il est venu.

**C : Comment ça se passait justement avec ?**

**M :** Comment il faut faire, quand j'allais mourir, tout ça, ils ont posé des questions. Ils ont marqué là-dessus comment il faut faire, c'est marqué, alors. On peut pas revenir en arrière maintenant, c'est écrit comme ça, on laisse comme ça.

**C : Et votre fille était présente donc, pendant ces moments-là, comment ça s'est passé avec votre fille ?**

**M :** Ben, rien, elle a posé comment *(rires)* que je voulais, si je voulais le curé, si je voulais pas, je sais pas ! Si c'est quelqu'un qui fait le discours !... Quand ça arrive, ça arrive.

**Femme de M :** *(Elle parle depuis la pièce d'à côté)* xxx pour l'assistance xxx à la fin *M.*, c'était pas pour après. Donc c'est pour avoir une assistance spirituelle, c'était ça qu'il fallait dé', tu devais décider, c'est ça qu'on a, qu'on a mis xxx.

**M :** Assistance ? Eh ben quoi ?

**C : Vous avez discuté d'une assistance spirituelle ?**

**M :** Ouais, mais, ou bien si y a un curé, ou bien si y a quelqu'un comme ça qui fait.

**Femme de M :** Tu as même pas encore décidé ce que tu veux.

**M :** Un discours, ou je sais pas. Comment il veut la famille, il fait.

**C : Comment la famille voulait ?**

**Femme de M :** *(Elle parle depuis la pièce d'à côté)* La famille fait selon ta volonté, c'est toi qui dois te décider ! C'est pas à la famille de décider.

**C : Donc ça vous avez pu un petit peu décider ce que vous vouliez ?**

**M :** Bon, on a décidé mais…

**Femme de M :** Il y pense pas, il y pense pas…

**C : Et, je sais aussi que pendant ces visites des infirmières, vous avez peut-être reçu des informations sur la BPCO, sur le, le traitement ?**

**M :** Ouais, le traitement, et.

**C : L'évolution.**

**M :** L'évolution, c'est, bon, y a des jours ça va, bon. Je peux dire pour le moment, c'est stable, c'est pas stable, stable, stable, c'est un moment ça va, un moment ça va pas.

**Femme de M :** *(Elle parle depuis la pièce d'à côté)* Ça fait à peu près deux ans qu'il fait pas à l'hôpital, hein.

**M :** Ça fait deux ans que je vais pas à l'hôpital, bon ben, mais je, je prends les médicaments, l'antibiotique parce que ça fait deux mois, voire trois mois, c'est depuis le mois d'août maintenant, mois août maintenant je le prends de nouveau. Pis j'ai toujours la bouche sèche, comme maintenant j'ai, sèche, sèche. *(Il se lève pour aller prendre de l'eau)*. Je sais pas pourquoi, si c'est à cause de ça, là… J'ai toujours une petite bouteille à boire.

**C : Prenez seulement.**

**M :** Pour mouiller la bouche *(il cherche de l'eau dans la cuisine)*. Comme ça. Je sais pas, faire un discours, je sais pas qui c'est qui va faire le discours.

**C : Et, et, quelles informations supplémentaires sur la, la BPCO auriez-vous aimé avoir ?**

**M :** Supplémentaires ?! Y a pas supplémentaire, ça se guérit pas, ils ont dit que ça guérit pas ! Ça peut stabiliser un petit peu, mais ça guérit pas…

**C : [Pas de, pas d'informations supplémentaires ?**

**Femme de M :** *(Elle revient dans la pièce)* Est-ce que t'as compris la question qu'elle t'a fait ?] Est-ce que t'as compris la question qu'elle t'a fait ? T'as pas compris maintenant la question qu'elle t'a fait. Vous voulez répéter s'il vous plaît ?

**C : Alors, c'était quelles informations supplémentaires sur la BPCO auriez-vous aimé avoir ?**

**Femme de M :** *(Elle traduit dans une autre langue pour M.)*. (*Puis elle s'adresse à C.)* Lui il vit comme ça, il vient quand il vient, ça a été toujours comme ça, la vie vient quand, je vis aujourd'hui, demain, je sais pas, il se passe ce qu'il se passe, ça me, je m'en fiche ! C'était toujours comme ça, lui. Alors ça lui est égal ! S'il va bien, j'espère qu'il aille bien tout le temps, s'il va bien, ça va ! Quand, quand ça va

mal il est affolé, il faut appeler l'ambulance, *(elle imite le bruit de difficultés respiratoires)* il peut pas : « appelle, appelle, au secours, au secours, au secours ». Mais autrement, il s'en… Il fait mauvais temps, il fait froid, il sort avec la jaquette toute légère, je dois aller le chercher dans l'ascenseur : « habille-toi, habille-toi mieux que ça ! ». Ou bien : « fais attention ! ». Je dois toujours lui courir derrière. Alors, il, il, ça, ça lui est égal ce qu'il se passe, dans un moment, demain, plus tard, ce soir. Donc, c'est, c'est un homme qui s'en fiche *(rires)*, c'est ça que ça m'énerve, parce qu'il ne prend pas soin de lui, c'est ça *(rires)* ! *(En s'adressant à M.)* C'est vrai, ou c'est pas vrai ?... C'est vrai ou c'est pas vrai ? Tu penses pas à ce que, à ce qu'il faut après, voilà. Alors, comme informations supplémentaires, qu'est-ce qu'il pourrait y avoir de plus ?

**M :** Qu'est-ce qu'il pourrait y avoir de plus ? Rien.

**Femme de M :** Rien.

**C : Rien.**

**Femme de M :** Voilà. Mais il faut, il faut, si tu veux savoir quelque chose sur ta maladie, qu'est-ce que tu peux faire plus de ce que tu fais, par exemple, c'est ça par exemple, n'est-ce pas, que vous aurez voulu avoir une opinion ?

**M :** Faut marcher, le Docteur il dit : « faut marcher, faut marcher. Vous sortez tous les jours. Faut faire promenades ».

**C : Et quand c'était les infirmières qui sont venues à la maison ?**

**M :** Aussi, la même chose.

**C : Elles ont pu vous donner des informations ?**

**M :** Informations, informations, des questions qu'ils me posaient, je répondais, c'est tout.

**Femme de M :** T'as eu un bénéfice de, de, de, de, des questions, des informations. T'as, t'as, t'as, *(elle traduit dans une autre langue pour M.)*.

**M :** *(Il parle dans une autre langue avec sa femme)*.

**Femme de M :** Ça lui fait plaisir d'avoir sa compagnie, mais c'est pas parce qu'elle lui apporte quelque chose *(rires)*.

**C : Ça vous, vous avez apprécié quand même ?**

**M :** Oh ben oui.

**Femme de M :** *(rires)*. Parce que lui, il est quelqu'un qui aime trop la compagnie. Il sort tous les jours, durant trois heures et demi, quatre heures. Et il rentre à 20 heures, 21 heures, 22 heures, minuit, ça dépend. Il aime bien la compagnie de dehors ! Voilà. Et c'est ça *(rires)* !

**C : Donc là, quand c'était les infirmières, vous avez apprécié la compagnie ?**

**M :** Mais, mais oui, pourquoi pas.

**C : Ça c'était, c'était agréable ?**

**M :** Mais la compagnie, c'est la compagnie parce que, maintenant, c'est, c'est tout moderne, moi je comprends rien, alors j'ai l'habitude de jouer un petit peu aux cartes. La compagnie, quand on est quatre, cinq, on joue, le temps ça passe, comme ça, quand on joue, le temps ça passe… Et pis on pense pas à la maladie, on pense à rien du tout, on pense aux cartes…

**C : Vous pensez à autre chose… Et, et quand elles venaient aussi les, les infirmières, ça, ça faisait de la compagnie ?**

**M :** Mais oui… *(toux)*.

**C : Et, voilà donc c'est quelque chose qui vous a quand même plu ?**

**M :** Mais oui…
**C : Mais pas, pas besoin d'informations supplémentaires ?**

**M :** Qu'est-ce que tu veux avoir comme informations supplémentaires ?!

**Femme de M :** Faites-lui un exemple.

**M :** Un exemple.

**Femme de M :** Faites-lui un exemple. Par exemple.

**C : Je pense quand elles sont venues, elles ont pu donner des informations sur comment ça se passe pour les médicaments, comment ça évolue, etc.**

**M :** Les médicaments, les médicaments, c'est toujours pareil.

**C : Et pour la, pour la gestion des symptômes physiques, comme de la difficulté à respirer ou de la fatigue, comment ces visites elles ont pu vous aider à gérer, ou non, ces symptômes ?**

**M :** Mais la fatigue, les gérer, xxx comme que je fais toujours.

**Femme de M :** Oui, mais on t'a expliqué quelque chose qui t'as fait du bien, par exemple, on t'a expliqué quelque chose ça te fait du bien. Oui ou non ?

**M :** Mais oui, quelque chose, elle m'a, elle m'a fait bien expliqué, mais je me rappelle pas tout, j'ai pas tout dans la tête ce qu'est-ce qu'ils me disaient. Ils me me disaient les phrases comme ça, et ça, et ça.

**Femme de M :** Des questions.

**M :** Oui, non, comme c'était.

**C : Y avait, y avait une phase voilà, de questionnaire, et pis.**

**M :** Elles posaient les questions, moi je disais : « oui », ou : « non », « combien, à combien de souffle, combien de douleurs, combien ça ». Maintenant je me rappelle pas tout ça, chaque trois mois. Moi je xxx journée xxx comment passe.

**C : Mais c'est ça y avait cette, cette partie questionnaire, pis l'autre partie aussi où elles vous donnaient des informations, ou.**

**Femme de M :** Des conseils, des explications.

**C : Voilà, des conseils, des explications, voilà sur la prise en charge des symptômes physiques aussi psychologiques, c'est que elles ont peut-être pu.**

**Femme de M :** Comment réagir quand t'as une crise, par exemple.

**M :** Ben quand j'ai la crise.

**Femme de M :** Tu te perds, [tu te perds.

**M :** Quand j'arrive pas à récupérer], je suis perdu, quoi.

**Femme de M :** Quand il a la crise, il panique.

**M :** Je panique un petit peu... xxx. Bon, quand je vois comme ça, je prends tout de suite un antibiotique, comme ils ont dit il faut prendre tout de suite un antibiotique, alors, petit à petit ça commence à xxx. Souffle dans la bouteille, souffle pour dégager, je fais xxx, je fais le masque. Le masque, je dors très mal la nuit, c'est mieux avant d'aller dormir que le matin quand je me lève. Après, xxx je fais tout le machin, prendre les médicaments, pis ça commence petit à petit à revenir à normal. Et oui… Voilà.

**C : Et, voilà donc… De, peut-être que, de quoi auriez-vous besoin ?**

**M :** Moi, moi j'ai rien besoin, pour le moment. J'ai besoin, quand je suis mal foutu, je monte à l'hosto. Ou un médecin, je commence à avoir l'antibiotique, s'il me vient la crise, je monte tout de suite en haut. Parce que, le *Docteur A. (pneumologie)*, il m'avait dit, quand il me prend les crises comme ça, il faut prendre 15 jours, 15 jours l'antibiotique plus le goutte à goutte. La dernière fois que j'étais, deux ans en arrière, il m'a dit : « il faut prendre 15 jours, 15 jours, vous restez 15 jours là, pis après c'est bon, 15 jours goutte à goutte et antibiotique ». Je suis ressorti, il m'a pas renvoyé ni à gauche ni à droite, je suis revenu à la maison, il m'a dit : « non, vous restez là jusqu'au xxx ». Tu vas à X *(hôpital)*, tu vois personne. J'étais à X *(hôpital)*, je suis revenu deux jours après, je xxx remonté à l'hôpital. A X , *(hôpital)* avec la machine xxx, faire la gym et tout, mais personne qui s'en occupe. Oui, ils passent pour manger, tout ça. Mais la douche, y a personne si t'arrives pas, y a personne qui te surveille. Tu vas à la physio, bon, y a quelqu'un, mais xxx, bon. Tu te sens mal, tu sonnes. J'ai dit : « mais, ça va pas, j'arrive pas xxx ». ; « oh, mais vous restez tranquille là, pis je vais revenir ». Et pis après il revenait plus. Alors, xxx ils voulaient m'envoyer là-bas, après j'ai dit : « non, non, j'y vais pas ». xxx je suis resté à X *(hôpital)* une semaine de plus, et pis après je suis revenu là. Même à X *(hôpital)*, je xxx les crises, prendre la douche, xxx, ouvrir la porte, ils regardaient à la place de me donner un coup de main pour m'essuyer *(rires)*. Ils ouvraient la porte et ils regardaient... Tu veux faire quoi ?! C'est pour ça, c'est pour ça quand j'ai les crises comme ça, je veux pas aller sur place, rester à l'hôpital ou bien. Bon, à l'hôpital, si j'ai la crise, j'attends, il vient quelqu'un quand même, même si je vais tout seul, il y a quelqu'un avec moi à la douche…

**C : Et, et, et si on revient vraiment à ces, quand elles sont venues une fois par mois les infirmières, de quoi d'autre auriez-vous eu besoin, quand elles venaient, une fois par mois ?**

**M :** Mais, j'ai besoin, j'ai rien besoin ! Bon, ils me posaient les questions, c'est tout, besoin… xxx.

**C : Quelle a été, pour vous l'utilité, de ces visites des infirmières une fois par mois ?**

**M :** Mais l'utilité ?! Il m'a posé tous les questions qu'il a marqué là.

**Femme de M :** *(Elle parle depuis la pièce d'à côté)* Elle t'a servi, l'utilité, ça veut dire elle t'a servi à quelque chose, ou à rien, quand elle venait ?

**M :** Ben, elle me posait les questions, je répondais, elle servait à quelque chose, c'est sûr, elle servait à quelque chose, mais !

**Femme de M :** Dans le sens, que ça t'a fait du bien, ou pas ?

**M :** Mais oui ça faisait du bien !

**C : Les, les visites vous [ont fait du bien ?**

**M :** Mais oui !] Ça m'a fait du bien, ça discute un petit peu, ça parlait du beau temps. Moi, c'est le temps qui me xxx, je suis toujours devant la télévision *(rires)*. Maintenant, l'hiver, je vais aller où ?...

**C : Donc ça, ça vous faisait passer du temps ces visites ?**

**M :** Elle passait une demi-heure de temps, une heure, passait le temps, discutait.

**Femme de M :** xxx *(rires)* Ça a changé le son des cloches *(rires)*.

**M :** Ouais.

**C : Ouais donc, donc c'est quelque chose qui vous a plu ces visites, parce que ça, vous me disiez ça vous faisait de la compagnie, et ça, ça vous faisait passer le temps ?**

**M :** Ouais… Bon, y a tellement des choses qu'on peut y aller, je sais pas, mais bon. Je suis pas pour y aller, comme ça… Moi avec la maladie que j'ai, tu veux aller où ?

**Femme de M :** *(Elle parle depuis la pièce d'à côté)* Parce que tu sais y a des maisons de personnes âgées où dans la journée où, t'as des cercles de récréation, pour les, pour les personnes âgées. Mais toi tu aimes pas y aller, tu t'estimes pas mieux, tu veux pas y aller. A part le bistrot, pour toi y a pas autre chose.

**M :** Oui, je vais aller.

**Femme de M :** Sinon y aurait où te passer le temps, hein, où y a les personnes âgées, comme toi, où ils font des jeux, des trucs comme ça.

**C : Parce que, là vous me disiez vous êtes pas mal à la maison, du coup, c'est quelque chose que.**

**M :** Non, je sors tous, tous les jours, je sors de la maison, je sors tous les jours. J'ai pas, bon, aujourd'hui il pleut, quand je suis pris, je sors pas. Mais autrement, si je suis pas pris, je sors tous les jours. Si je suis bien, je sors tous les jours, si j'arrive à marcher, si j'ai pas *(il imite des difficultés respiratoires)*, si j'ai pas les crises, je sors tout le temps. Faire un petit tour, je vais boire un café. Je vais faire une partie aux cartes. Mais maintenant, je sais pas si je vais plus jouer, si j'arrive y aller là, parce que… L'âge, ça, ça compte aussi, maintenant.

**C : L'âge ?**

**M :** Eh oui !... xxx il est très bien, il est en forme. Maintenant, les, les jambes, ça commence à, les genoux à lâcher un petit peu, les jambes ça commence à faire mal ! L'asthme, ça monte tout le temps…

**C : Donc, si, si je résume un peu, par rapport à ces visites que vous avez eues une fois par mois. Donc, donc voilà y avait toutes ces questions. Mais c'est quelque chose, [que ça vous a plu, vous disiez, parce que ça faisait, ça faisait de la compagnie.**

**M :** Oui, oui, ouais ! Bon, maintenant, ouais, ça m'a plu, pourquoi pas.] Tous les questions qu'il m'a posées, maintenant, c'est les questions, discours, parce que, bon, discours, moi je suis venu ici j'avais 17 ans, j'étais à X *(canton)*, j'ai voyagé, j'ai voyagé quatre, cinq ans, de, de 55, 56 jusqu'à 60, tourné tout la Suisse. Je travaillais chez X *(entreprise)*, bon, maintenant ils existent plus. Voitures tamponneuses, on avait les, les montagnes russes, l'himalaya, y avait beaucoup de manèges là, y avait trois, quatre xxx. Alors après, je me suis arrêté ici, à X *(canton)* à X *(quartier)*, j'avais trouvé une chambre aux X *(quartier)*. Après X *(quartier)*, j'étais X *(quartier)*. Après, j'étais aux X *(quartier)*, après je suis atterri ici. Donc, toujours par là autour, quoi. Mais, la première fois que je suis venu à X *(canton)*, sur la plaine là, en 56, à nouvel an, sur la plaine, c'était pas comme maintenant, tu étais pas encore née à cette époque-là *(rires)*. T'as pas vu comment c'était ! Une grande fontaine, y avait là, c'était tout vert, y avait des xxx, y avait deux, trois chalets, y avait des bistrots dans les chalets. Après, bon, après ils ont tout enlevé ça.

**C : Mhh.**

**M :** Eh oui ! On a vu, on a vu beaucoup de monde. Après j'avais travaillé dans la peinture, j'avais travaillé dans le bâtiment, xxx ponçage du parquet. Alors ça, ça sentait mauvais, tout la poussière, c'est pour ça mes poumons ils sont foutus. xxx a choppé.

**C : A cause de la poussière ?**

**M :** Ça fait 27, 28 ans, à X *(lieu de la profession)*. Et voilà, juste à la retraite... J'ai travaillé, ça allait ! 65 ans, ça allait, mais après, ça a commencé, ça fait cinq ans maintenant, jusqu'à, à 70 ans, ça allait.

**C : Ça fait cinq ans que ?**

**M :** Euh 6, 7 ans, commencé à *(il imite des difficultés respiratoires)*.

**C : Que vous avez la BPCO ?**

**M :** Ouais.

**C : Ouais, ouais. Donc voilà, si, si je résume un peu par rapport, voilà, à ces visites, donc voilà, c'est quelque chose qui, qui vous a, qui vous a plu. Y avait pas mal de, de questions. Vous avez pu discuter un peu de, des soins en fin de vie, du soutien spirituel aussi que, vous désiriez.**

**M :** Ouais, elle a, elle a tout marqué là-bas, c'est tout, tout, tout marqué, quoi.

**C : Tout noté.**

**M :** C'était tout noté. Alors.

**C : Ouais. Et, est-ce que vous aimeriez encore rajouter quelque chose par rapport à ces visites ?**

**M :** Bon, le discours, quand je suis venu, ou ce que j'ai travaillé, tout ça, bon, je sais pas quel discours il va faire quand je meurs, le curé.

**C : Par rapport au, au discours du curé, oui.**

**M :** Je suis venu, xxx revenu, j'ai voyagé 25 ans xxx. Plus après, j'ai travaillé dans le bâtiment. Pis après à X *(lieu de la profession)*, comme laveur de vitre. Je pense y a de tout, tout.

**C : Donc vous avez, vous avez pu noter ce que vous vouliez que ?**

**M :** Non, il a pas noté tout ça, il a pas noté. Non, ça on n'a pas dit, on n'a pas. Si vous voulez rajouter tout ça à mon discours quand je meurs, autrement, tant pis. xxx. Tu veux quoi. Il t'arrive un accident, poum tu meurs, et c'est tout. Il fait qu'est-ce qu'il peut, il fait qu'est-ce qu'il peut la ville, maximum, il fait le maximum, quoi, pas trop de frais.

**C : Qui fait le maximum ?**

**M :** Ben, il paraît c'est la ville qui s'en occupe quand, quand quelqu'un qui meurt, qu'il habite en ville. Mais, moi c'est comme ça que j'avais entendu hein, c'est comme ça qu'on avait mis là.

**C : Donc ça, ça vous avez discuté avec les infirmières de ça ?**

**M :** Paraît, bon, moi je sais pas, bon moi j'avais des copains par là, je connais des fils qui sont morts, c'est la ville qui s'en occupait, il a payé maximum, parce que comme il habite en ville, en ville, c'est la ville qui s'en occupe, mais je sais pas. Maintenant je sais pas si, s'ils lui ont fait brûlé, s'ils lui ont mis sous terre, s'il faut payer, tout ça, je sais pas. Moi j'avais entendu qu'il fait le nécessaire, les fleurs, le machin. Mais maintenant, je sais pas. Je suis jamais trop au courant, xxx comment il faut faire. Parce que je sais que ça coûte cher, l'enterrement. En X *(pays)*, ça coûte très cher, une fortune.

**C : Mais là, c'est la, la ville qui s'en occupe.**

**M :** Hein ?

**C : Là c'est la ville qui s'en occupe.**

**M :** Mais, moi comme j'ai entendu, les personnes qu'ils habitent en ville, c'est la ville qui s'en occupe, alors je sais pas.

**C : Ouais, ouais. Est-ce qu'y a autre chose que vous aimeriez rajouter par rapport à.**

**M :** Non.

**C : A ces visites des infirmières, par rapport à votre vécu ?**

**M :** Non.

**C : Par rapport à ce que vous avez aimé, pas aimé ? Ce que vous auriez voulu avoir en plus ?**

**M :** Qu'est-ce que je peux avoir plus, je peux rien avoir plus !... Rien avoir plus ! On attend, on attend la fin des jours !... J'espère que ça dure encore un petit peu, mais, quand ça arrive les crises comme ça, c'est très dur.

**C : C'est dur.**

**M :** Tu paniques. Tout le monde, il panique. C'est pas seulement moi. Aussi les autres.

**C :  Ça, ça fait paniquer d'avoir ces crises.**

**M :** Aussi les autres, hein, xxx à l'hôpital…

**C : Merci beaucoup en tout cas, de.**

**M :** Je vous en prie.

**C : D'avoir pris le temps de, de répondre à toutes ces questions encore *(rires)*.**

**M :** Quand on peut. Quand on peut plus.

**C : Voilà, je crois qu'on a.**

# Participant n°6

| |
|---|
| Nom d'emprunt : Marie |
| Âge : 74 ans |
| Sexe : F |
| Groupe : Contrôle |

**C : Si je reprends, vous avez participé donc à une étude qui avait pour objectif de comparer le traitement habituel de votre maladie pulmonaire, la BPCO, avec une prise en charge, précoce, soutenue et intégrée. Vous avez été dans le groupe bénéficiant des soins habituels.**

**M :** Absolument.

**C : Pouvez-vous me dire comment ça s'est passé ?**

**M :** Du point de vue du médecin, ou du point de vue étude ?

**C : De, de l'étude.**

**M :** De l'étude. Alors, aucun problème. J'ai eu la visite de vos, dames infirmières, absolument charmantes. Bon, on a fait connaissance, pis, au fil des, des rendez-vous, on se connaissait, c'est, heureusement les mêmes qui viennent. Et ça, je pense c'est très, très bien qu'on ait les mêmes personnes. Parce que ça c'est difficile chaque fois, de recommencer ça, son histoire, qui est pas toujours simple. Et là, j'avoue que *Claire (infirmière de recherche)* était vraiment, très, très sympa, et ensemble on a rempli des questionnaires, on a, j'ai pu répondre à leurs questions, elle précisait certaines choses, et, elles sont. Non, j'ai eu, j'ai participé volontiers et, les questions étaient pertinentes, répondu aux questionnaires, c'est plus simple que de parler sans autre.

**C : Donc ça, vous avez apprécié que ce soit toujours les [mêmes personnes ?**

**M :** Voilà, alors ça] c'est une chose qui est tout à fait appréciable. Et c'est un petit peu le, le défaut de certaines autres structures, qui vous mettent en contact régulièrement avec des personnes différentes. Alors, c'est bien d'avoir un peu de changement, mais suivant quoi, pour nous, qui sommes dépendants pis qui avons quand même des traitements, le traitement en lui-même, c'est des comprimés, de l'oxygène, lourds, oui, mais, fatiguants. Donc c'est très agréable d'avoir les mêmes personnes pour répondre. Et elles finissent par vous connaître, par voir le petit détail qui est connu, qui, qui rend service.

**C : Pas devoir ré expliquer.**

**M :** Voilà. Vous repartez pas à zéro à chaque fois, et ça c'est, c'est très agréable.

**C : Des questions aussi vous avez trouvé pertinentes ?**

**M :** Oui, ben quand on se connaît, c'est un questionnaire qui est, qui va pour tout le monde, avec un degré de, de réponse. Bon, parfois ça prête à sourire, mais, par rapport à soi. Mais si c'est concernant une autre personne qui a pas, tout à fait, puisque chaque BPCO est différente quand même, pas tout à fait les, les, les mêmes options, les mêmes difficultés, elles se comprennent très bien les questions, non, non. Et là, justement, les infirmières comme *Claire* ou *Julie (infirmière de recherche)*, ont été là pour préciser les, les informations qui étaient nécessaires.

**C : Donc c'était clair.**

**M :** Tout à fait, tout à fait ! Non, pis on peut leur poser la question, ou elles c', elles voient tout de suite si on comprend pas. Parce que c'est pas évident de leur répondre avec les, les, les plus cinq c'est, c'est très fort, c'est pas fort, c'est souvent inversé pour nous obliger à réfléchir. Mais, au début, c'est, c'est, c'est un peu compliqué, j'avoue. Mais bon, avec de l'aide, on s'en sort facilement, quoi.

**C : Ouais. [Donc elles ont pu vous aider et préciser ?**

**M :** Non, non, c'est, oui, tout à fait.] Pis suivant la réponse, ça surprend, donc elles reposent la question, la reformulent, peut-être différemment, ce qui permet de, de préciser la, la réponse aussi. Donc ça c'était une très, très bonne chose.

**C : Et, qu'est-ce que vous attendiez donc en participant à, à cette étude ?**

**M :** Personnellement, pas grand-chose. Je connais ma maladie, je sais que ça n'ira peut-être pas vers les beaux jours, et effectivement depuis quelques années, ça a quand-même décliné, principalement ces deux dernières années. Pour moi, personnellement, je pense pas que l'étude apportera beaucoup. Par contre, l'étude pour les, les futurs, rendront certainement de, de grands services. Y a, y a beaucoup de choses qu'on pourrait dire en tant que malades, utilisateurs, par exemple, d'oxygène, que ce soit liquide, que ce soit concentrateur, c'est, y a pleins de choses pratiques à dire. Mais en tant qu'étude, sur ce qui peut être, ce qu'on pourrait améliorer, ce qu'on aimerait que ce soit, qu'on puisse avoir des améliorations, mais, oui, vite, je veux pas dire vite fait, mais bien fait. Mais par exemple, une greffe du poumon, personnellement j'ai travaillé à l'hôpital, donc je sais tout ce que ça implique, avant, pendant et après. Les médicaments, les, les contrôles, et tout ça. Et ça, si on pouvait trouver des solutions meilleures, plus faciles, plus adaptées, plus adaptables, moins lourdes, ce serait bien. Mais ça on y est peut-être pas encore tout à fait. Eh oui ! Les médicaments, là aussi, j'aimerais bien, mais, qu'est-ce qu'on peut faire.

**C : Ou alors d'avoir des, des médicaments, des traitements moins lourds et [plus efficaces ?**

**M :** Oui, parce que là,] bon, je parle de mon cas, j'ai heureusement pas besoin de faire des, des aérosols. Ma, ma soeur qui souffrait également de, de la BPCO, devait elle, faire des, des, des, des aérosols, à tire-larigot, je sais pas cinq, six fois par jour. Donc elle passait ses journées là-dessus. Et là, je pense je dois quand même avoir aussi une machine pour les problèmes de, de gaz de, je sais plus comment ça s'appelle cette machine. Euh… Je dirais pas le nom, parce que je le sais plus, je le sais même pas. Donc, ça m'angoisse et c'est angoissant d'être toujours dépendant d'oxygène, de fils, de. Bon, pour moi qui suis sur un, comment… j'y arriverai, j'y arriverai, de concentrateur, si y a une rupture de, une coupure d'électricité, ça coupe, y a plus rien ! Donc, tout est angoissant. Et c'est une maladie qui est très, très angoissante, ça je dois le dire. Et là c'est de trouver les trucs, de prévoir, d'organiser, et on devient angoissé de service, c'est terrible ! Et ça, c'est le plus pesant, je crois, c'est cette angoisse permanente.

**C : Une maladie angoissante, et des traitements [xxx.**

**M :** Ben voilà c'est,] si, si y a une coupure d'électricité, j'ai plus de, d'oxygène. Si, pour d'autres personnes, si la bonbonne est vide, et qu'ils viennent pas tous les 36 du mois, faire telle chose, telle chose, ça fonctionne pas ! Donc vous avez, vous êtes toujours à la merci d'un, d'un pépin technique. Et ça, une personne qui est, qui peut se déplacer, elle ouvre la fenêtre, elle pourra respirer, elle a soif, elle pourra aller chercher de l'eau. Nous, on est, on est très handicapé par ce, ce problème-là. Et pour sortir, là je vous dirai franchement, depuis, bientôt une année, je suis pour ainsi dire pas sortie, à part un ou deux traitements médical, et même maintenant, là j'ai la chance d'avoir mes médecins qui viennent à domicile. Et d'un côté, ça me pèse, d'un autre côté, je suis très bien chez moi, j'ai un balcon, mais c'est un gros handicap malgré tout. Ça je pense que c'est très, très handicapant comme maladie. Et l'évolution elle est angoissante, c'est l'angoisse, c'est le handicap.

**C : Qui sont pesants.**

**M :** Oui, ça c'est très pesant.

**C : Le fait d'être dépendant.**

**M :** Oui, ces dépendances sont très difficilement gérables et, et. On peut pas, on peut pas ne pas y penser ! Y a la moindre des, le moindre des petits problèmes, ben voilà, c'est, c'est un problème ! Je fais quoi ?!... C'est le, cette maladie, bon c'est, c'est une maladie, y en a d'autres qui sont pires ! Mais c'est une des évolutions, c'est l'évolution, si on arrivait à revenir en arrière, à récupérer l'avantage de par un, je sais pas, la, la, la, le renouveau des médicaments ou des traitements, c'est quelque chose qui gagnerait énormément.

**C : Oui. En terme de trouver des nouveaux [traitements, des nouveaux médicaments, et].**

**M :** Voilà, des nouveaux traitements, des,] voilà, des, des choses nouvelles. Mais bon, c'est, là le nou', le renouveau, c'est, c'est la greffe. Et malheureusement, je crois qu'y a pas beaucoup, beaucoup d'autres choses à, à faire valoir. Bon, pis à un certain âge, je, je m'engagerais pas dans une greffe à, à mon âge.

**C : Vous disiez aussi que c'est quelque chose d'assez coûteux, aussi [cette greffe.**

**M :** C'est coûteux,] et c'est très lourd et, et que ce soit avant, pendant, et après ! Après, y a le, l'entretien, y a les, les problèmes médicamenteux. Et quand vous êtes, vous êtes seule, ben, ben vous allez vivre en institution, hein. Le gros problème il est là encore. Les, les soins à domicile, et tout ça, c'est bien gentil mais, à un moment donné, vous pouvez plus, vous pouvez pas.

**C : Rester à domicile.**

**M :** Non. Là c'est, ça pose un problè', rester seul, pardon, seul à domicile, j'entends bien. Là c'est un problème de dépendance, qui est important. Et je crois c'est, c'est à tenir, il faut en tenir compte dans les études, dans les traitements et dans les évaluations. A long terme.

**C : Le fait de.**

**M :** Ouais, ce problème de dépendance. Si une personne, au début, ben, ben non, j'ai commencé, oui, mon mari était, vivait encore. Pis après ben, il est décédé, vous êtes seul, la famille s'exporte, quoi *(rires)*. Ce qui est normal, c'est une évolution normale de la, de la vie. Et là, vous vous retrouvez seul, alors ça c'est, avec la maladie qui dégénère, c'est difficile à gérer. A un moment donné, c'est assez difficile à gérer, j'avoue.

**C : Parce que là vous aviez donc votre mari ?**

**M :** Ah ben au début, pis j'avais l'oxygène que la nuit, donc, si vous voulez c'était, bon, c'était une maladie qui me, m'empêchait pas de faire beaucoup de choses ! C'est vrai que quand vous arrivez à un certain, un âge certain, je suis pas une vieillerie tout à fait, mais enfin, vous avez passé la, la grande partie de votre vie, ben, c'est vrai que vous aspirez, bon y a des gens qui veulent encore faire 36 voyages, moi non, j'avais tout fait ça avant. J'aimais bien ma vie tranquille, tranquille. Et j'avais plus besoin d'aller ma balader ou comme ça, moi, non. Et là, après, ben, plus de mari, la, la maladie qui empire, les, les escaliers qui descendent plus que ceux qui remontent. Donc c'est difficile à gérer, oui, c'est difficile, oui.

**C : Voilà, pis la, en terme de soutien, quel type de soutien vous avez de la part de vos proches ?**

**M :** Alors, j'a' j'avais, oh soutien, oui je m'entends bien avec la, avec la famille. J'ai un fils, on s'entend bien, 'fin il habite pas chez moi, il vient, je le vois régulièrement. Mais, c'est pas, c'est pas lui qui viendra, excusez-moi me, me faire mes toilettes, hein ! J'avais ma soeur, on se téléphonait tous les jours, depuis dix ans. Et elle a fait six mois d'hôpital, on se téléphonait deux fois par jours. Elle est décédée au bout de six moi d'hôpital. Donc, c'est vraiment une chose qui me manque énormément ! Et pis sans ça le, les amis, bon j'ai, j'ai beaucoup de soutien par des amis. Mais, autrement, les voisins, ben, tout le monde travaille. Donc c'est, y a un isolement qui pourrait se faire rapidement. J'aurais pas pensé avant, mais, si, je le vis, je me sens pas isolée, pas, c'est pas ça, hein. Mais il pourrait se faire suivant l'évolution de la, de la maladie, facilement, parce que vous êtes plus en capacité de faire les choses. Et là, si encore, j'avais pas internet, je sais pas comment je vivrais. Bon c'est, c'est, vous êtes dépendants de nouveau des gens qui veulent bien faire vos courses. Heureusement j'ai internet, je travaille comme ça. Mais autrement non, c'est encore une autre difficulté. Si vous deviez allez faire pour trois patates, deux carottes, faire vos courses, c'est pas simple. Bon, je veux dire y a X *(aide et soins à domicile)*, y a ces choses-là, mais, c'est quand même des services qui sont, c'est une dépendance extérieure qui est difficile à gérer.

**C : Oui. Parce que là vous avez internet, des solutions à la [maison.**

**M :** Voilà.] Oui, oui, alors là j'ai, j'ai internet et livraisons à domicile, commander chez un, gran' grande surface et pis, pis voilà, ça va ! Bon y a des limites inférieures, supérieures, c'est évident. Mais, c'est une chose, une fois que vous êtes un peu organisé, y a pas de problèmes. Non, ça c'est une chose qui fonctionne. Pour autant que vos outils personnels fonctionnent. Le jour où vous avez plus d'électricité, ben vous êtes mort hein, là.

**C : Oui. Donc voilà, vous êtes pas isolée comme vous disiez, mais c'est [quelque chose.**

**M :** Ah oui, ah non,] avec internet non, je suis pas isolée, j'ai, les, les e-mails, ça fonctionne. Les amis, les, les anciens collègues, tout, ça, ça fonctionne très, très bien ! Mais je pense aux personnes plus âgées qui ne connaissent pas ce système. Ma soeur ne connaissait pas et, elle, ça a été très difficile. Sa fin de vie, ses dernières années a été diffi', 'fin cette dernière année surtout a été difficile, dépendante des gens qui voulaient bien aller faire les courses. Ben, vous êtes dépendant de votre entourage ou des, des amis. Ou justement d'une, des oeuvres, telles que X *(aide et soins à domicile)* à X *(canton)* ou des choses comme ça. Et ça c'est assez, bon, y a des gens que ça gêne pas, moi je trouve ça assez, pesant, c'est, c'est assez difficile à, à accepter.

**C : Cette dépendance ?**

**M :** Oui, oui. Ça j'ai, moi j'ai, j'ai toujours eu mon indépendance. Et cette dépendance me, me pèse beaucoup, alors ça c'est une chose qui me pèse. Pour le reste, on s'habitue, mais c'est vrai que c'est, on y pense quand même.

**C : Vous y pensez, c'est, voilà, quelque chose un peu qui vous pèse cette dépendance [aux autres.**

**M :** Oui, oui. A moi, moi] personnellement oui. Et je pense qu'à d'autres personnes aussi, c'est, c'est très pesant. Mais bon c'est l'évolution de la vie, et il faut, faut faire avec. Combien de temps ça va durer, combien de temps vous risquez de tirer à la corde, est-ce que ça va déc' décliner ? Rien que de devoir penser à tout ça, c'est juste pas simple.

**C : Vous pensez [à l'évolution de la maladie.**

**M :** Oui,] absolument. C'est pas une évolution qui ira du côté des escaliers qui montent, hein. Vous remontez pas au Salève à pied, là. Non, non, là c'est, faut pas rêver. D'abord vous prenez de l'âge, vous avez plus de problèmes, et puis ensuite l'activité physique ! Vous avez quand même moins de possibilités avec un, être relié en permanence à une machine, c'est difficile d'avoir de l'activité.

**C : Oui. Donc ça, tout ce qui est activité physique, mouvement.**

**M :** Voilà, tout à fait. Mais il faut, faut vraiment le vouloir. Certains jours je me pousse. Je fais du vélo, je fais des exercices avec des élastiques, j'ai une physio qui vient à domicile, ça va, là je, mais je me force à le faire tous les matins, tous les jours.

**C : A faire cet exercice physique, et.**

**M :** Oui, oui. Parce que sans ça, vous êtes paumé ! Les muscles ne résistent plus à un moment donné, si vous restez assis en permanence, c'est foutu. Pis vous avez plus envie ! C'est ça le problème !

**C : Faut un peu se pousser.**

**M :** Oui, oui. Alors ça, c'est une chose où il faut vraiment encadrer les gens. Et, les encadrer, mais pas les, pas, pas, pas peser sur les gens pour ça. On m'a jamais forcé, je me force toute seule. Mais je sais pas, une personne qui pourrait plus, qui a plus envie, est-ce que vous arrivez à la forcer, je crois pas. Donc ça, c'est un côté de la maladie qui est, qui à mon avis est à étudier à fond et de, essayer de trouver des, des formules ou des, des formulations, des propositions, ça je pense qu'y a un côté de cette maladie qui, qui échappe encore. On en parlait pas avant, on racontait pas, ça se disait pas ! Ça je pense qu'y a des progrès à faire de ce côté-là.

**C : Du côté  de ce qu'on peut faire en terme [d'activités physiques, de soins ?**

**M :** Ouais, voilà, et pis de,] de prise en charge du patient. Parce que la maladie elle évolue, on le sait. Mais le jour où vous avez plus envie de faire, d'être quand même entouré, d'être, qu'on vous pousse un petit peu, des fois il suffit peu de choses pour que on se stimule et pis ça, ça repart. Donc là, je pense que c'est très important ce côté-là.

**C : Trouver des, des choses pour [stimuler le patient, pour pousser.**

**M :** Ouais, ouais. Là y a des gens] qui ont pleins d'idées là-dessus, moi j'en n'ai pas, c'est pas mon rayon. Mais, mais y a des, des spécialistes là, et je pense que de ce côté-là, y a quelque chose à, à creuser, pour aider les patients… dans l'exercice, l'activité de cette ma', concernant cette maladie. Là je pense que y a quelque chose à faire.

**C : Oui. Des, [des spécialistes pourraient se pencher là-dessus.**

**M :** Voilà, peut-être, voilà !] Ouais, je pense. Parce que, à la limite actuellement on, on propose pas grand-chose. Si vous demandez, bon moi j'ai une physio, y a quatre ans qu'elle vient, depuis que j'ai l'oxygène à 100, 24 heures sur 24. En sortant de l'hôpital, j'ai, elle est venue, pis maintenant on a continué, on continue. Et elle vient, elle contrôle mes exercices, elle en fait d'autres, suivant dans quel était je suis, elle évolue avec moi. Mais si vous avez pas une physio, ou si vous avez pas quelqu'un qui vous suit, là je pense c'est, c'est une, un côté de la maladie qu'il faut, qu'il faut développer, ou des soins qu'il faut, qu'il faut cadrer et aider, parce que c'est pas simple d'avoir cette maladie.

**C : Oui. Pouvoir un peu mieux cadrer ça, [trouver.**

**M :** Ah, mieux] cadrer, proposer au moins ! Bon, jusqu'à maintenant, j'en n'ai jamais eu besoin. Donc j'ai rien demandé ! Mais je m'en rends compte au fur et à mesure, pis j'ai la chance d'avoir le, le caractère que j'ai, et, et d'avoir les contacts que j'ai, qu'il me reste, que je maintiens, que je, je conserve, je, je les cultive, comme l'amitié, ça se cultive. Et ça se cultive, pas en, en six mois ! Ça se fait de longue date ! Et ça, j'ai, j'avais un côté, j'ai eu la comparaison avec ma soeur qui avait pas ce, ces contacts, et elle était très, très isolée. Tandis que moi j'ai tout ces contacts et c'est, c'est très important.

**C : Le fait d'avoir [ces contacts que vous avez cultivé permet d'être moins isolée, et.**

**M :** Ouais, voilà. Absolument.] Et de pouvoir appeler, téléphoner, avoir un contact, que les gens passent vous voir, ou. Mais c'est, c'est difficile à un moment donné, parce que vous avez, vous avez pas envie, hein, tout d'un coup, il faut un petit peu qu'on vous bouscule. Enfin bon, c'est une histoire de, de caractère hein, ça c'est pas.

**C : Oui. Mais ça, vous avez l'impression qu'y a, y a pas grand-chose qu'on peut proposer, ou qu'on propose maintenant ?**

**M :** Ben, jusqu'à maintenant on m'a jamais rien proposé de ce côté-là ! J'ai jamais rien demandé vous me direz *(rires)*. C'est vrai que, soyons honnêtes, jusqu'à maintenant j'en, j'en éprouvais pas le besoin ! Mais, maintenant, non, je vais pas demander qu'on me propose quelque chose, mais je me dis mais, c'est une chose à laquelle j'ai pensé, qui peut peut-être aider quelqu'un d'autre, je sais pas. Peut-être un, quelque chose qui existe déjà, je sais pas ! J'ai jamais eu l'occasion d'en parler, pis en fait avec qui vous voulez en parler ?! Votre médecin, il est pas là pour ça. Les infirmières de l'étude, ben on les a vues dans le cadre de la, des infirmières. Mais peut-être que ça servira à la formation des futurs pneumologues, ou, 'fin à la société, je sais pas, c'est une idée, comme ça. Je vous l'offre, gratuitement *(rires)*. Y a pas de problèmes.

**C : *(rires)*. Toute idée est bonne à prendre.**

**M :** Tout à fait.

**C : Mais vous savez dans, vous sauriez dire dans quel domaine ce serait utile qu'on vous propose quelque chose ? Ou, ou pour d'autres patients comme vous ?**

**M :** C'est, c'est surtout sur la longueur, les journées sont longues, 24 heures ! Si vous avez pas un traitement spécifique, que vous lisez pas, que vous avez pas de télévision, ou la radio, ou, ben les journées sont longues ! Lorsque j'étais hospitalisée, je voyais de ces, de ces mamies qui étaient pas beaucoup plus, plus jeunes, qui étaient quand même un petit peu plus âgées que moi, mais, elles pouvaient rester, mais sans rien faire pendant des heures ! Une, par exemple, elle lui donnait ses chaussettes, pis l'infirmière lui donnait, pour lui enfiler ses chaussettes, ou ses socquettes. Deux heures après, elle avait encore ses socquettes à la main, elle avait pas bougée ! Je lui ai dit : « mais là il faut stimuler ces gens-là ! » Et, mais si vous faites rien, donc, ben voilà, quoi ! Et c'est à ça que je pense, je me dis mais c'est vrai que si on fait rien, les gens demandent rien, ben ils attendent que ça passe ! Mais vous pouvez attendre longtemps, hein ! Le coeur, il s'arrête pas comme ça parce que vous faites rien. Mais bon y a tout qui, tout s'enchaîne. xxx ces espèces de souvenirs, de, de, de flashs qui me reviennent de temps en temps. Quand je me souviens des périodes d'hospitalisation que j'avais, ou d'autres. Non, moi j'ai envie d'aller de l'avant, j'ai envie, j'aimerais aller mieux, j'aimerais m'en sortir. J'ai pas besoin d'aller au, au pré, aller manger de l'herbe, c'est pas ça ! Mais j'entends, revivre, bon je veux pas dire revivre, non, c'est pas le mot, mais, pouvoir retrouver certaines activités que j'ai, j'ai malheureusement dû abandonner depuis une année. Aller manger avec des amis, ou, sortir… Oui, y a pas besoin d'aller loin, moi j'ai, j'ai pas besoin, je vais au petit bistrot du coin, manger, j'allais régulièrement. Et, depuis décembre, j'y suis pas retournée parce que, ben, y a eu la maladie, y a eu l'effondrement, les, les. Bon, c'est vrai que j'ai eu encore de la radiothérapie, donc ça, ça a pas aidé la, la maladie. Et puis, la, la, ben vous êtes touché, là. Là, ça, ça touche terriblement. J'ai perdu mes cheveux, ils ont pas repoussé, c'est, c'est, c'est une mutilation ! Et là, je pense que ça, ça, ça plombe et avec l'oxygène en moins, vous, vous arrivez pas à remonter. Et là, peut-être que une, une stimulation un peu plus, oui peut-être, je sais pas, je sais pas.

**C : Ouais, comme vous disiez, y a des, des périodes aussi, et.**

**M :** Alors j'ai une psy', je fais aussi une psychothérapie, avec une médecin psychiatre, que j'ai fait avec le xxx. Et on travaille là-dessus, on, mais, mais c'est vrai que vous grattez, vous grattez, pis vous grattez un truc, pis ça sort un autre. Et pis, est-ce que vous, on s'effondre davantage. J'ai eu pleins de choses ces dernières années qui font que c'est, c'est difficile à remonter. C'est, c'est très, très difficile.

**C : Mais vous avez, voilà, ce soutien psychothérapeutique.**

**M :** J'ai un soutien, mais y a des moments où vous dites : « mais ça sert à quoi bordel !» *(rires)*. Pardon, pardon pour le gros mot, je l'ai, je l'ai dit doucement. Non, mais c'est vrai qu'y a des moments où vous mettez tout en doute. Je crois que quand on est bien aussi, ça arrive aux uns et aux autres de, de tout remettre en doute, de remettre en question la, tous les éléments de ce qu'il se passe, je crois que c'est naturel.

**C : Parfois vous vous demandez avec la psychothérapie si c'est utile, ou.**

**M :** Ah ouais, ouais, je, je me dis : « mais ». Pis après, elle vient, on, on parle, on, on regratte un sujet, on revient sur autre chose. Je travaille entre-deux. Pis, pis après, ben, voilà, on redescend un escalier, on essaie d'en remonter. Mais bon, vous descendez deux marches, vous en remontez qu'une ! C'est un peu délicat... Que faire ! Ahlàlàlàlà ! Ah, ça c'est la, la philosophie de la vie, je sais pas…

**C : Voilà, pis cette phase, où c'est un petit peu plus difficile à remonter comme [vous disiez.**

**M :** Ah oui, oui, oui.] Que dès que vous descendez c'est, y a, y a le temps aussi, tous ces changements. Vous avez les, les périodes cette année de canicule, pis tout à coup il fait froid, plus froid que, c'est, c'est en dents de scie, hier y avait un foehn pas possible ! Aujourd'hui mais vous vous avez vu ce temps ce matin, mais je respirais à peine ! Et, et, y a, tout est un, vous, vous êtes tellement plus sensible avec cette maladie ! Pis bon, est-ce qu'on met un peu la, la faute là-dessus, ça aussi. Hein, on veut pas voir non plus tout ce qui est peut-être.

**C : Mais vous sentez que vous êtes plus sensible aux changements de temps ?**

**M :** Oui, alors là je pense que c'est une sensibilité qui, qui s'exacerbe au fil des années, des, des mois et des années. Je pense qu'on est plus sensible. On s'écoute peut-être davantage. Vous savez quand vous pouvez aller, vous faites ci, vous faites ça, bon, vous êtes un peu essoufflé, on s'arrête, on reprend son souffle. Tandis qu'avec l'oxygène, vous, vous plus souff', ben vous arrêtez, pis vous attendez que ça passe ! Mais vous pouvez attendre un moment certaines fois, je, j'ai un problème de, de saturation, et ça c'est de, d'être, de vivre avec ça, c'est très difficile. Surtout si on prend, bon je prends ma saturation parce que je veux, je veux savoir le pourquoi du comment, j'ai besoin de comprendre, mais là j'ai de la peine à comprendre.
**C : Par rapport à la saturation ?**

**M :** Par rapport à la maladie, par rapport à ce qu'il faudrait faire pour que ça aille mieux. C'est, c'est très difficile, je sais pas y a des jours je, je, *(soupir)* vous vous mettriez des coups pour, vous avez envie de pleurer de rage parce que vous y arrivez pas.

**C : Vous aimeriez avoir des, quelque chose.**

**M :** Ça veut pas dire le coup de baguette magique, mais pas loin… Un médicament, vous vous dites avec tous les médicaments qui se font et tout, quelque chose qui vous aiderait à respirer un peu mieux, ben on peut rêver, quoi. C'est pas comme ça que le corps humain fonctionne et on a trouvé beaucoup de choses, on trouve beaucoup de choses quand vous entendez tout ce qu'ils trouvent à la télévision, toutes les do' les, les avancées technologiques, médicales et tout. Vous dites : « mais pourquoi ils ont encore pas trouvé quelque chose qui puisse faire que ça aille mieux ».

**C : Pour la BPCO.**

**M :** Voilà. Et que ce soit plus simple qu'une, une greffe et tout son contenu, contenant et, et tout ce qui va avec. Ben à part ça, je vois difficilement ce qu'on peut faire. De l'oxygène, et puis ça descend, donc vous devez plus d'oxygène. Plus d'oxygène, ça, ça signifie du, trop de gaz en général, trop de gaz carbonique, voilà. Vous devez encore avoir un appareil pour, un masque pour enlever le gaz, ce qui, ce qui me pend au nez et ce qui me panique complètement. Je suis complètement paniquée à cette idée-là !

**C : D'avoir un masque ?**

**M :** Ouais. La nuit, d'avoir un masque, je suis paniquée. Vous avez vu j'ai demandé qu'ils m'envoient de la documentation. J'ai, j'ai besoin de savoir, j'ai besoin de m'organiser. Me dire qu'il faut un masque, y aura quand même l'oxygène, y aura ci, y aura ça. Mais faut que je puisse organiser, parce que la nuit, je dois me lever ! Et vous faites comment, donc c'est, c'est paniquant, c'est paniquant !

**C : De pas savoir.**

**M :** Ouais, de pas savoir de, je sais pas. Pis de vous dire : « peut-être que ça ira mieux, pis peut-être pas vraiment ». C'est angoissant... Je peux, je peux rien changer, c'est, les choses se feront d'elle-même. C'est vrai que ça… ça use les organes. Et vous dites le coeur il en prend un coup, pis à un moment donné ben, ben voilà quoi…

**C : Pis en terme d'informations justement sur la, sur la BPCO, sur ben les traitements, de quoi d'autre [auriez-vous ?**

**M :** Ecoutez quand on a], si vous voulez j'ai, l'étude je l'ai pris un peu en route. Ou en fait l'étude, c'est pas elle qui m'a détectée, c'est pas grâce à elle que j'ai détecté que j'avais la BPCO. Donc, à la limite j'étais déjà au courant des traitements possibles avant, j'avais déjà été hospitalisée, j'avais déjà, c'est en 2014 que j'ai rencontré ces dames, que j'étais hospitalisée. Mais j'avais depuis 2011, l'oxygène en permanence. Depuis 2006, j'avais la nuit. Et depuis 2011, je l'avais en permanence. Donc, je savais déjà à peu près à quoi m'en tenir. Même si on refuse de croire ou de penser à, à la suite. Non, non, on veut vivre le moment mais on veut pas savoir ce qu'il se passe après. Pis quand vous êtes dans le moment après, c'est difficile à accepter. Ouais. Ça alors j'avoue honnêtement c'est, à l'heure actuelle, c'est très difficile à accepter, pis vous dites ben, si ça doit être, que ça soit vite fait,

que ça traîne pas. Voilà. C'est là que vous vous dites : « bon ben on remplit ses directives anticipées ou ses dernières volontés, et tout ce qui va avec ». Et bon, chacun le sait, quoi, faut le faire.

**C : Ça c'est, c'est quelque chose que vous avez déjà fait ?**

**M :** Ah oui, oui c'est fait, oui, oui ! Et ça, l'hôpital déjà la dernière fois, ils m'ont demandé. Et même ils m'avaient demandé. Je veux pas de soins intensifs, pas d'intubation. Et ils m'avaient, un, un médecin m'avait, ouais ça doit être dans mon dossier à l'hôpital, si le coeur s'arrêtait, est-ce qu'on le relançait, j'ai dit : « non ». S'il s'arrête, c'est que ça, y a une puissance supérieure qui dit que stop, eh ben, ou la machine elle dit stop, ben c'est stop, quoi. Non, non, pour vivre, non, hein, ça non, c'est pas pour moi, ça. Alors c'est vrai que vous vous dites, bon ben le coeur il va, il va s'arrêter un jour, c'est plus, ce côté là qui est… angoissant, oui, c'est angoissant, c'est sûr. Mais il faut pouvoir le, le, le verbaliser. Ça je l'ai fait, je le fais, c'est pas une, pour moi c'est pas une question, mais c'est une chose qui va un peu avec votre étude, c'est un peu la, la suite où une chose en parler en cours de, de, d'évolution de la maladie.

**C : C'est important de pouvoir [parler de.**

**M :** Ah ouais !] De, de pouvoir le formaliser, je pense. Personnellement je, je le conç', je le trouve comme ça. Beaucoup de gens n'en voudront pas, hein.

**C : De ces directives anticipées ?**

**M :** Voilà, les directives anticipées, quoi faire. Et pis le jour où vous êtes devant, voilà. Du jour où mon père est décédé, j'ai fait les faire-parts de décès de, de ma mère, de. Non, c'est quand ma soeur est décédée en 87, une de mes soeurs, on avait oublié d'avertir untel, on avait oublié ci, sur le faire-part, et tout, enfin la famille avait oublié, le, le mari. J'ai dit : « non. Ça c'est une chose qu'il faut pas ». Et, dans le moment, vous êtes, avec l'émotion, vous faites les choses à l'envers. C'est pour ça qu'il est utile et important en parler avant, à mon avis. Et là, au décès de mon mari, tout était clairement mis, on savait ce qu'on voulait, l'incinération, le jardin du souvenir. Et c'est des choses qui, si vous le dites pas avant, personne le sait. Et, et, si vous me l'aviez dit y a, y a, y a une vingtaine d'années, j'aurais dit : « tu rigoles, eh, j'y suis pas, c'est pas l'heure ! » Pis bon, s'il vous arrive un accident, et vous êtes seul, c'est l'heure. Donc c'est des choses qui doivent intervenir dans les, dans, dans cette, dans l'évolution de cette maladie. On sait pas ce qui va arriver. Ça, je pense que les médecins, même eux, à l'heure actuelle ne savent pas, donc c'est important d'en parler. Et de, de, oui, je trouve important d'en parler.

**C : Que les médecins en parlent ?**

**M :** Voilà. Médecins ou les infirmières ou les, les psychos, ou qui vous voudrez, mais, que dans l'évolution de la maladie, on en parle au cours de, au cours de l'évolution de la maladie. En tout cas moi j'ai, j'en ai parlé moi, mais, personne n'est, n'a été branché là-dessus.

**C : C'est vous qui [avez parlé de ça en premier.**

**M :** Ah oui, oui, c'est moi qui ai, oui tout à fait alors,] j'ai toujours été très claire là-dessus. Mais ça a été suite à des, des, des constats d'erreurs dans, dans la famille ou chez des amis. Je me dis mais quand ça arrive, c'est dramatique. Pis que si vous voulez pas qu'on vous le fasse, il faut le dire, faut le dire avant.

**C : Mais, mais on vous l'a pas proposé ?**

**M :** Ben non, mais tant que vous êtes bien, je vais pas vous proposer une intubation ! Mais le jour où vous commencez d'être éclopé, je suis arrivée un jour aux urgences, mais je crois que c'était en 2014, ils voulaient mettre en, ouais ben c'est là, en 2014, quand j'ai rencontré les filles, ils voulaient me mettre aux soins intensifs, j'ai dit : « non ! » Je connaissais ma, le chef des soins intensifs qui est venu bavarder vers moi, parce qu'on se connaissait de longue date, pis j'ai dit : « non ». Et là j'ai été au xxx, quelques jours, et pis après j'étais en médecine, et là j'ai toujours dit : « non. Les soins intensifs, intubation, je ne veux pas ». Le réveil aux soins intensifs, c'est exclu…

**C : Comme vous disiez, de, de faire avant plutôt que quand l'émotion est là.**

**M :** Voilà, quand l'émotion vous, vous savez pas ce que vous voulez, hein. Pis quand vous êtes comme ça, on vous demande pas, on vous y envoie. Alors que si vous avez dit les choses avant, on vous y envoie pas. Et là, les directives anticipées, je les ai faites selon, ma soeur les a faites avec *Julie (infirmière de recherche)* et avec ces dames, et, et une chose merveilleuse, qui a atténué la fin de vie de ma soeur, c'est *Julie* qui, je crois a quitté la derma', la pneumologie et, et à X *(hôpital)*, et c'est elle qui m'a répondu, qui a eu, qui était avec ma soeur les deux derniers jours de sa, de sa vie. Ma soeur a essayé de me le dire le, le, la veille de son décès, et le, le jour de son décès, j'ai téléphoné, c'est l'infirmière qui m'a répondu, l'infirmière c'était elle, qui était avec elle. Et une demi-heure après on me téléphonait comme quoi elle était décédée. Donc, elle l'a accompagnée, et ça, je pense que, bon c'était clair, elles avaient rempli les directives anticipées. Et pour ma soeur, j'avais toujours dit : « mais il te faut le faire, tu dois le faire. Tu veux pas que ça t'arrive n'importe comment, il faut en parler, il faut le faire ». Et on l'avait fait. Et elle l'a fait formaliser, correctement avec ces dames de la pneumologie. Et là je crois que c'est une bonne chose. Et pis bon, y a beaucoup de gens qui voudront peut-être pas en parler mais, le jour où ça arrive, c'est trop tard, vous pouvez pas en parler. Si vous êtes là sur une, comment, pas là qu'on peut vous demander votre avis, non, non.

**C : Pour vous c'est important qu'on puisse le faire [avant xxx ?**

**M :** Ben oui, si on veut finir,] on sait quand même que on n'ira pas en remontant la pente. Donc ça ira plutôt en déclinant. Donc un jour ou l'autre, le déclin c'est quoi ? C'est le départ. Donc il faut qu'il soit fait correctement. Et non pas tout d'un coup, on est mis devant le fait, ben voilà. Non moi c'est pas comme ça que je le vois.

**C : Ouais. Pouvoir le faire avant.**

**M :** Ouais. Y a pas besoin de s'appesantir là-dessus, non, c'est clair ! Mais l'évoquer et, et reprendre le sujet, je pense que ça vaut la peine pour apaiser les patients, pour apaiser les gens, oui.

**C : Certaines personnes voudront peut-être [pas le faire, mais c'est bien de pouvoir le proposer ou.**

**M :** Voilà exactement, de voilà,] de leur proposer, des fois on, on le propose, pis après on réfléchit et puis ça c'est des choses qui peuvent très bien aller dans, dans ces, ces, ces, ces moments de discussion, et tout. C'est des, vraiment des sujets à, à reprendre, à prendre et à reprendre.

**C : Oui. Des décisions des, des soins en fin de vie.**

**M :** Ben ouais, si, si, s'il arrive, qu'est-ce que vous voulez faire, qu'est-ce qu'on fait. Et là, y a beaucoup de choses on, on, on dit quelque chose, mais on le, on l'enregistre pas. Ça non, ça c'est, à un moment donné vous imprimez plus, hein. Ça fonctionne plus de la même manière. Tout est, ben c'est là qu'on voit que tout est <u>influencé</u> par cette maladie... Vous êtes tellement accroché à, à tout faire juste, que vous faites des bévues. Et ça c'est, c'est la grosse angoisse, c'est ne pas faire de, pas commettre d'impairs pour pas, pour que ça continue de fonctionner ou à peu près.

**C : Ouais. Pas commettre d'impairs [par rapport à la maladie.**

**M :** Voilà, exactement, ouais]. Ouais, ouais parce que sans ça, ça fonctionne plus, pis y a des ratés, et pis les ratés, vous faites quoi. Il faut se lever pour aller mettre plus d'oxygène, c'est pas le pied non plus, y a pas de, y a encore pas de télécommandes. Pis bon même s'il y avait une télécommande, vous l'oubliez sur la table, pis vous êtes dans le fauteuil, ça, ça le fait pas non plus. Et avec la télévision, ça devient pareil *(rires)*. On peut tourner ça longtemps comme on veut, ça peut être drôle.

**C : La maladie prend un peu le dessus.**

**M :** Ouais, mais dans tout elle prendra le dessus. Vous préparez quelque chose, *(elle simule des bruits de difficultés respiratoires)* il faut aller augmenter l'oxygène, bon ben ok j'oublie. Pis après il faut pas oublier de le redescendre, parce que, ben voilà, c'est, c'est en permanence ce, ce yoyo et c'est, c'est, c'est <u>pesant</u> ! Mais bon, c'est ce qui vous fait, ce qui vous permet de, de, de continuer, quoi.

**C : L'oxygène… Et… je sais qu'on peut aussi recevoir du soutien au niveau spirituel. Quel type de soutien avez-vous reçu au niveau spirituel ?**

**M :** *(rires)* Rien, zéro, niet. Pourtant je suis, je suis très croyante, pas pratiquante du tout. Ça fait 40, quasi 50 ans que je suis à X *(ville)*. J'ai vu un pasteur le jour de mon mariage, j'ai vu un pasteur le jour du, du mariage de mon fils, pis le jour de son baptême. Ben vous, vous pouvez vous arrêter à peu près là, ou des baptêmes ou j'ai été, ou de la, des, des sépultures où j'ai dû aller. Mais bon, là c'est, c'est clair que si quelqu'un a besoin, y a pas beaucoup, hein, on vous en donne pas dans les quartiers. A l'hôpital, ben ils ont, à l'époque ils, ils avaient, ils avaient de quoi faire, et y avait une équipe qui était assez extraordinaire. Je me souviens de mes débuts à l'hôpital, où y avait une équipe formidable, mais tant catholique que protestant, d'ailleurs. Et, c'était, c'était vraiment formidable. Et ça, malheureusement y a plus. Bon, une fois de temps en temps, vous avez un pasteur qui vient, et encore ! Faut déjà demander, pis, pis rester assez longtemps. Maintenant, vous restez plus assez longtemps, ils arrivent plus à tourner, et je pense qu'y en n'a plus beaucoup des pasteurs, ou des, des curés à l'hôpital. Ils ont juste, ils vont donner l'extrême-onction, ou des choses comme ça. Mais faire la tournée pour aller papoter, j'y crois pas tellement. Mais ça, je pense que c'est aussi une chose qui, bon, qui est un peu, ça dépend du, du patient. Celui qui veut oui, il faut lui en proposer, mais celui qui en veut pas, et en proposer, soit celui du quartier, s'il en a dans le quartier, c'est super, s'il en n'a pas, qu'est-ce que vous pouvez proposer ? C'est clair que c'est aussi un sujet dans ces études qui peut être évoqué. Et c'est vrai que moi, par exemple, on m'aurait proposé euh je sais pas si j'aurais dit oui, peut-être oui, pouvoir discuter de ce que, oui, c'est vrai que, si j'avais eu, peut-être. Là quand je pense quand j'étais à X *(lieu)*, j'avais demandé à voir le, le pasteur, il était venu une fois ou deux, voir, on avait parlé. Mais, mais aux X *(nom d'hôpital)*, ils ont pas le temps. Et même en X *(service d'hôpital)*, je me souviens pas de l'avoir vu, pourtant j'y suis restée deux mois, non. C'est vrai que, quand on pense au nombre de patients. Et, et maintenant où, où vous restez vraiment peu de temps à l'hôpital, ils ont pas le temps, y a pas l'équipe pour.

**C : Donc ça, c'est quelque chose que vous avez apprécié à X *(lieu)*, [mais là aux X *(nom d'hôpital)*, y avait pas le temps ?**

**M :** Oui, alors tout à fait, non, mais y a,] y avait pas la structure, y avait plus la structure. A l'époque, y avait une, une structure qui permet, les patients restaient pour un, je sais pas moi, des trucs, ici, maintenant ça se fait ambulatoire, ils restaient 15 jours ! Donc le, le pasteur il avait le temps de passer une fois ou deux ! Et si vous le demandiez à l'infirmière, elle vous l'envoyait, dans les 20, 48 heures, mais avant même ! Donc, c'est une chose qui se faisait, qui se fait plus. Et ça, c'est peut-être dommage, c'est peut-être une chose à creuser, aussi. Et c'est vrai que le patient qui retourne à domicile, si on pouvait le lui proposer, pour ceux qui veulent, bien entendu, c'est peut-être une chose que j'aurais accepté. Et bon, là il faut que vous, vous, que ceux qui proposent les soins, que ce soit le patient, l'hôpital, le, le, qui vous voulez mais, aient aussi les, les bonnes connexions, pour envoyer au bon endroit. Je sais pas, c'est, c'est un sujet effectivement, qui peut être étudié, aussi dans ce cadre-là. C'est vrai que c'est peut-être une chose que j'aurais accepté.

**C : D'avoir un pasteur ou un soutien au niveau spirituel.**

**M :** Ouais, ouais, probablement. Parce que ça c'est important.

**C : Donc voilà, quelque chose peut-être à creuser et à voir, [selon les patients, et ?**

**M :** Absolument, exactement !] C'est une question d'abord à poser, pis ensuite, mais d'abord mettre en, avant de proposer aux patients, c'est dans la structure actuelle, le, par votre étude, de, de, c'est un sujet à creuser actuellement. Voir ce qui serait possible de, de, proposer ou de mettre en place. Mais personnellement, on me l'aurait proposé, je pense que j'aurais accepté.

**C : En tout cas pour vous, ça aurait été quelque chose [que.**

**M :** Ouais,] c'est une chose que j'aurais accepté avec plaisir.

**C : Ouais, [quelque chose à, à creuser.**

**M :** Ouais, ça c'est,] absolument ! Ça c'est un très bon sujet de, de thèse ou de, d'examen pour passer votre bac.

**C : Ouais *(rires)*. Le soutien spirituel.**

**M :** Voilà, exactement.

**C : Si on revient, ben vous m'avez dit au, au début, par rapport à pourquoi vous aviez participé à cette étude, c'était plutôt pour faire avancer les choses, [plus pour xxx.**

**M :** Tout à fait !] Je connaissais l'hôpital, j'y ai travaillé pendant 31 ans. Ça fait maintenant 17 ans que je suis à la retraite. Donc j'ai fait partie de beaucoup de groupes, que ce soit, j'étais dans, j'étais membre fondateur du groupe X *(nom de groupe)*, j'ai été dans le groupe X *(nom de groupe)*, j'ai été dans le groupe, je crois que j'ai fait un peu tous les trucs possibles et même le, un des premiers journal de l'hôpital qui s'appelait le X *(nom du journal)* à l'époque. Donc j'ai été dans, dans pas mal de trucs, et là, quand on me parle d'une étude et que… physiquement et psychologiquement je suis concernée, ça reste une maladie qui me concerne, je me dis si je peux donner un, <u>aider</u>, je, je participe volontiers. Et c'est dans ce but-là que je l'ai fait.

**C : Pour participer, [pouvoir aider.**

**M :** Oui,] pis je me dis ben ça peut m'apporter à moi aussi quelque chose, et c'est vrai d'en parler, de parler, de, de voir les choses, de, de <u>verbaliser</u> certaines choses, ça peut être utile, ça rend service. Absolument. Et c'est dans, dans ce but-là, dans ce cadre-là que je l'ai fait, en fait.

**C : Parce que y avait voilà, la, la moitié des participants qui avaient les traitements habituels comme vous. Et l'autre [moitié qui avait cette prise en charge globale.**

**M :** Absolument, ouais on tire au sort, j'avais, voilà.] J'avais, moi j'avais un traitement, comme vous avez dit là, le premier là.

**C : Habituel.**

**M :** Voilà, habituel. Et là, je pense c'est une chose aussi, là je vous ai dit au début l'histoire, je pense c'était avant que vous enregistriez, le, suivant le groupe dans lequel vous, vous êtes, à mon avis, non, même que vous soyez les deux groupes, <u>bien</u> en parler avec le médecin traitant. Parce que le patient, quand le médecin traitant voit le document, est-ce qu'il comprend comme le patient souhaiterait qu'il puisse le comprendre, ça peut créer des malentendus. Et je pense que chez moi y a eu un léger malentendu et, là j'ai, j'ai eu vraiment une, une appréhension, une angoisse, en me disant : « mais mon médecin croit que je vais aller me faire soigner chez vous. Et je vais être suivi là-bas, et il me dit au revoir ». Et comme c'est vrai que c'était déjà une relation de très longue date, vous êtes encore plus angoissé. Donc y avait pas besoin de ça pour m'angoisser plus, n'est-ce pas. Donc, c'est vrai que là j'ai, j'ai eu un petit, un petit problème de, [de connexion.

**C : Malentendu.]**

**M :** De malentendu, oui. Et, bon heureusement ça s'est rapidement mis d'aplomb, mais je pense que là je, au moment où, où le où le patient est hospitalisé et fait partie de ce, de ces, de ces individus-là, ben il faut que l'hôpital puisse avoir un, un meilleur contact avec le médecin traitant. Je pense que.

**C : Pour éviter les malentendus, et.**

**M :** Voilà. Je pense que là y a un petit problème. Et le, l'hôpital ne, là j'avais fait une, comment un examen des, des bron', comment ça s'appelle, un…

**C : Une spirométrie ?**

**M :** Ouais, non mais j'ai, j'avais été ambulatoirement à un contrôle euh pulmonaire, voilà, j'y arriverais, contrôle pulmonaire, pis ils ont envoyé le, le résultat à mon médecin traitant. Donc lui m'avait dit : « ben j'ai reçu ». Il a pu avoir des points de comparaison avec ce qu'il avait. Mais

autrement, c'est vrai que j'étais pas suivie, j'étais suivie par mon médecin traitant, qui était pneumologue, mais qui me suivait depuis 35 ans comme médecin de famille, si vous voulez, comme généraliste. Donc c'est vrai que ça c'est jamais posé avant. Et là je pense effectivement que le contact, il, il, il faut peut-être avoir un meilleur contact. C'est quand même un, un service qui doit garder un contact plus, plus rapproché avec le, le médecin du patient.

**C : Donc faire [attention de.**

**M :** Ouais,] là je pense qu'y a un petit quelque chose de mieux à faire.

**C : D'accord. Faire attention à ça. Et, quand y a eu justement ce, ce tirage au sort, quelle a été votre réaction en apprenant que vous étiez dans le groupe qui avait ce traitement habituel et pas la prise en charge globale, spécialisée ?**

**M :** Ben, j'étais pris en charge par mon médecin trai', mon pneumologue qui était mon médecin traitant, ça me posait pas de problèmes. C'est quand même pas une chose que je <u>découvrais</u>, j'en, j'en souffrais déjà depuis quelques années. Donc, ça m'a pas dérangé plus qu'autre chose. Je savais que c'était des entretiens, si je pouvais y participer, pis donner un coup de main et aider, je le faisais très volontiers, je faisais ça, ça peut aider les, les suivants. Moi j'étais en plein dedans. Ce que ça pouvait m'apporter c'est un soutien moral, mais, mais ça s'arrêtait là ! C'est pas la science à l'heure actuelle qui, qui évoluait grâce à mon témoignage. C'était pas ça le but, en fait, c'était de participer à, à l'ensemble de l'évolution.

**C : Plus, ça vous a fait comme un soutien moral, et ?**

**M :** Oui, non ben, bon oui, ah oui, oui, le fait de parler, de pouvoir, ça permet de se poser des questions, pis de verbaliser les choses, mais. Au moment de, de, du tirage au sort, que ce soit l'un ou que ce soit l'autre, personnellement je voyais pas grande différence. En fait, je sais pas ce qu'ils auraient fait de <u>plus</u>, si j'avais été dans l'autre groupe.

**C : Pour vous c'était plus de participer.**

**M :** Voilà, c'était le, le fait de participer, pouvoir, mon témoignage, si mon témoignage pouvait faire évoluer les choses, ça me faisait plaisir, c'est tout. Et ça s'arrêtait là j'entends, j'étais pas, je gagnais rien à, à être d'un côté ou de l'autre, en définitive. L'important c'était d'être là, quoi.

**C : L'autre groupe avait donc une infirmière qui venait une fois par mois pendant un an et qui évaluait les besoins au niveau physique, psychologique.**

**M :** Oui, voilà, ma soeur était dans ce cadre-là. Mais en définitive, elle évaluait, mais elle faisait rien ! Elle faisait rien de plus, puisque si y a un changement de traitement, c'est le médecin traitant, 'fin voilà ça s'arrête là ! Et en définitive, si j'avais été pendant une année avec une personne qui venait tous les mois, et là je crois bien que c'était cette *Julie (infirmière de recherche)* qui faisait ça chez ma soeur. Eh bien, *(soupir)* ça lui apportait le plaisir de la voir et pis, mais de l'évolution de sa maladie, ben voilà quoi... Non. Je vois pas ce que ça m'aurait apporté de plus d'un côté ou de l'autre.

**C : Ouais, ouais. Donc pour vous, vous voyez pas vraiment l'utilité de cette prise en charge ?**

**M :** Ben, écoutez, si c'est fait par un médecin, ok. Mais si c'est fait par une infirmière, elle peut tout au plus rapporter au médecin ce qu'elle a vu, mais, mais elle peut pas faire mieux, quoi ! C'est pas elle qui va vous, vous faire changer votre traitement. Et pis une fois par mois, c'est pas là que vous faites faire certains exercices que vous faites pas autrement, donc. Je vais pas dire que je mets un doute, mais, mais, oui, bon, ben voilà, quoi.

**C : Ouais. Pis, pour, pour votre soeur, c'était quelque chose de, d'agréable d'avoir [ces visites.**

**M :** Voilà, elle avait quelqu'un] qui venait, elle était seule, c'était une compagnie, elle passait un certain temps. Bon, suivant quoi elle devait faire ses aérosols, donc, suivant quand elle passait, c'était pas évident. Et pis, pis, pis, ça lui apportait, mais, non pas, bon, simplement c'était une personne sympa, et puis, passait un moment avec elle ! Et je vois, personnellement pas que ça lui a apporté

plus, plus que ça… Bon, elle est plus là pour témoigner, mais non elle m'en parlait, bon : « *Julie (infirmière de recherche)* vient ». Et pis, pis voilà : « J'ai vu *Julie (infirmière de recherche)* cette après-midi ». Pis ça s'arrête là, quoi… Elle lui faisait pas faire un exercice particulier, une fois par mois c'est, vous iriez tous les jours pour faire faire quelque chose, bon ce serait peut-être différent, ou une fois par semaine. Mais là une fois par mois, oui, bon, d'accord : « merci, sympa de venir, je vous fais le café, ou je vous le fais pas ». Pis voilà… C'est un peu ça.

**C : Le fait que ce soit une fois par mois, [c'est peut-être.**

**M :** Ben je vois pas] l'utilité vraiment, puisque c'est pas <u>elle</u> qui vous suit, qui peut <u>changer</u> votre traitement, qui peut. A la limite, est-ce qu'elle transmet une information au médecin traitant ? J'y crois pas vraiment, puisqu'elle dépend de l'hôpital, et, voilà. Je sais pas, là c'est le, l'infirmière peut dire si elle a l'impression d'avoir joué un rôle. Moi personnellement, du point de vue patient, j'ai pas l'impression qu'elle puisse faire grand-chose, qu'elle apporte énormément, mis à part une présence, où on peut parler et reparler de sa maladie. Si on veut pas, ben c'est pesant, ben si on veut ben on en parler pis on dilaye, pis voilà, quoi…

**C : Donc vous voyez pas vraiment la, pour vous y aurait pas eu vraiment de [différence entre les deux groupes ?**

**M :** Non, non, non, non, non] je vois pas grand-chose, mis à part que la mienne je la voyais tous les trois mois ou six mois, que c'était *Claire (infirmière de recherche)* et pis que, quand elle venait elle me téléphonait, pis on passait un moment ensemble et on remplissait le questionnaire, pis on bavardait. Mais non, je voyais pas une grande différence en définitive.

**C : Vous avez pas, [voyez pas vraiment l'utilité de ces visites une fois par mois ?**

**M :** Non, non] mis à part pour pas, excusez-moi, pour papoter, du point de vue médical, traitement ou… avancée, je vois, je vois pas, honnêtement je, je vois pas.

**C : Comme les, les infirmières peuvent pas changer, voilà, le traitement xxx.**

**M :** Et non, et pis elles vous, si elles vont, elles viennent une fois par mois, comme je dis, pour faire une, une respiration, je sais pas moi, votre petite boule, ou votre petit truc, ou votre petit machin, bon ben : « c'est bien gentil, merci, sympa, à dans un mois ! » Entre-temps, vous faites quoi ?! Donc, je vois pas, bon y a peut-être d'autres facettes, hein, qui m'échappent. Mais de mon point de vue, là, pour moi, je vois vraiment pas... Mais y a, là c'est, la personne, l'infirmière selon les renseignements qu'elle prend, qui peut dire, si oui ou non c'est utile. Moi personnellement, de mon point de vue, vu comme ça, je dirais non.

**C : Plutôt, plutôt pas utile.**

**M :** Ben non, je vois pas l'utilité.

**C : Ouais… Et… De quoi auriez-vous besoin aujourd'hui ?**

**M :** De respirer *(rires)*. De respirer mieux ! *(rires)* D'enlever mes angoisses. Non, d'aller mieux ! Mais bon là, j'ai des oedèmes, j'ai, j'ai tout ce qu'il faut pour bien faire, vous avez vu ce qu'il m'a écrit dans mon petit message *(elle fait référence à un email envoyé par son médecin sur son état de santé)*. Donc, c'est vrai que j'aimerais un médicament qui, qui me fasse récupérer comme j'étais l'année passée, pis que je puisse aller mieux. Mais là, avec tous les événements qui se sont passés c'est, moralement plus pesant et j'ai de la peine à, à passer le cap. Et assisse tranquille, là je, je récupère, je suis bien, y a pas de problèmes ! Mais dès que je me déplace, que j'utilise les bras, alors je suis, je peux plus respirer... C'est ça mon gros problème, actuellement.

**C : C'est un peu, les angoisses et le, le fait de respirer, de pouvoir mieux respirer.**

**M :** Ben ouais de, que la saturation soit, je sois mieux saturée, que je puisse mieux respirer. Bon, augmenter l'oxygène, c'est pas la panacée, donc c'est clair que y a quand même. Bon là, comme mon médecin disait, qu'y avait peu de différence de, des, des… vous avez lu, vous pouvez peut-être

le dire pour moi. Peu d'évolution dans, dans la, la maladie, mais quand même, bon je suis pas idiote, hein, je sais quand même que ça, ça décline quand même un petit peu par rapport à avant. Et j'ai, et j'ai beaucoup moins de, d'autonomie, j'ai, une moins bonne saturation, pour pas dire une saturation péjorée par rapport à avant. J'ai moins de résistance, ça je le, je m'en rends compte. Je dois faire, je dois m'arrêter plus souvent, je dois faire plus doucement, et je sais pas faire doucement. Moi je suis de la génération où on a toujours fait vite, on fait vite ci, pis on fait vite ça. Et encore maintenant je me lève, je pars, et je pars trop vite. Et après je me calme, pis je me dis : « on se calme, doucement ». Pis après ça, ça re va, quoi. Mais bon, c'est vrai que pour moi c'est, c'est un peu difficile… Mais bon ça c'est, est-ce que c'est, ben j'ai eu hier matin, non, lundi matin, une prise de sang, donc j'attends de voir si y a une, un résultat, le médecin me donnera quelque chose. Bon là il m'a donné des… des trucs pour faire pipi, diurétiques, pardon, j'y arriverai. Et, bon ben voilà, quoi, les jambes en l'air…

**C : Vous attendez ce résultat.**

**M :** Ben oui, ben, ben si y a un résultat, il me le fera savoir, quoi, puisque il m'a encore envoyé un message hier. Donc, c'est clair que suivant le résultat, il me, il me donnera quelque chose en plus. Sinon, ben bon, est-ce que c'est peut-être une infection, je sais pas, j'ai pas de fièvre, j'ai pas de. Non, y a, y a pas de raisons, j'ai pas des, des, des… j'expectore pas sale. J'entends je, là je fais très attention de prendre tout de suite des antibiotiques, j'en ai dans mon tiroir, je sais que si, si j'ai quelque chose, faut que je réagisse très vite, sinon ça, ça prend tout de suite de l'ampleur. Mais bon là, ça fait bientôt une année ou plus d'une année que j'ai pas pris d'antibiotiques, moins j'en prends, mieux je me porte. Mais, mais bon, s'il faut, je le ferai. Et là, une infection, ben comme il me dit hein, ça peut arriver, on sait pas pourquoi. Mais bon, lundi prochain j'ai mon généraliste qui vient, pis qui me fera le vaccin. Donc, d'ici là je, je veux aller bien, quoi. Ben on va essayer de remonter la pente, je vais essayer de, de, je vais essayer de faire mes exercices, d'en faire, d'augmenter un petit peu, les, les, peut-être pas les cadences mais la, la durée, essayer de, de refaire repartir la machine.

**C : Oui, oui. Refaire partir la machine, oui. Du coup si, si je résume, donc par rapport à, à cette étude, donc vous avez participé pour faire avancer les choses, [pour.**

**M :** Voilà, pour donner mon.]

**C : Voilà, pour, pour voir si y avait, on pouvait faire évoluer les traitements, [par rapport à, aux médicaments.**

**M :** Voilà.] De par un témoignage, si ça pouvait aider à faire évolution, évoluer la, la prise en charge de cette maladie, ce serait, ce serait bien.

**C : De pouvoir trouver voilà, quelque chose de, [de moins lourd qu'une greffe ou quelque chose.**

**M :** Voilà, pis si, si y avait tout d'un coup], un de vos médecins trouvait quelque chose, qu'il, qu'il pense à moi, pis qu'il m'en fasse, qu'il m'en fasse bénéficier, je serais ravie. Mais bon, je vais pas jouer les cobayes, non plus, hein, je vous dit tout de suite, *(rires)* soyons honnêtes.

**C :** ***(rires)*. Mais en tout cas voir, en tout cas avec votre témoignage, qu'est-ce qu'on [pourrait aussi faire pour.**

**M :** Si ça peut aider] à faire évoluer les choses, je le fais [avec plaisir.

**C : Pour améliorer] cette.**

**M :** Non, non tout à fait.

**C : [Cette prise en charge.**

**M :** Non, non je sais que dans le cadre] de l'hôpital, il faut faire des études, faut, on entend assez tous, tous vos médecins, que ce soit un, un en oncologie, que ce soit en pneumologie, ben voilà. C'est pas un problème, hein, Docteur F., je l'ai connu, il faisait son, son, il apprenait son métier.

Professeur A. *(pneumologie)* je l'ai connu, il apprenait son métier. Donc c'est tous des gens que j'ai connu, et ça me fait plaisir de faire, si, si je peux aider d'un côté ou de l'autre, je le fais avec plaisir !...

**C : Donc, ce qu'on peut faire, pour.**

**M :** [Ben oui, si on pouvait faire.

**C : Pour améliorer, ce serait.]**

**M :** Si on pouvait faire quelque chose, je serais, je dirais merci…
**C : Ouais. Voilà, quelque chose pour pouvoir améliorer cette maladie qui est aussi assez angoissante, et.**

**M :** Ah oui, elle est très, très angoissante ! Ouais, ouais, c'est très pesant, et très angoissant.

**C : Donc voilà, et, en tout cas pour vous, vous m'avez dit aussi que c'était utile d'avoir ces, ces directives anticipées, [c'est quelque chose qui pourrait être utile aussi.**

**M :** Oui, ça c'est une chose qui peut être utile.] Tout à fait pour.

**C : Pour d'autres.**

**M :** Absolument. C'est une chose, à mon avis, il faut en parler. Mais, beaucoup de gens ne veulent pas, hein. Ça c'est, c'est clair. J'avoue, ça c'est ma belle-fille qui me l'a dit, que mon fils a été très choqué, quand ma soeur a été hospitalisée, ben ma soeur qui est sa tante et sa marraine, et que avec le médecin ils ont parlé de ces directives anticipées. Alors lui il était.

**C : Ça a choqué votre fils ?**

**M :** Que ma, ma, ma soeur avait fait ça, ben il devait se douter que je les avais faites aussi. Et il était très surpris des dire' de ces directives-là. Donc, voyez c'est pas, c'est pas une option, c'est pas une directive qu'on peut obligatoirement donner à chacun. Mais que certains voudront, d'autres voudront pas en entendre parler, mais en entendre parler et en parler est une, à mon avis, une chose utile. Mais bon, pas enfiler en bloc l'histoire dans la tête des gens, non, parce qu'y en a qui prennent ça très mal. Mais mon fils a, a été très secoué avec cette histoire-là, paraît-il. Ça me serait jamais venu à l'esprit de lui en parler, quoi. Ecoutez, moi c'est, quoi qu'il savait toujours que, ben voilà, hein.. c'est chez moi... Non, non, on relance pas, on fait pas traîner, je, je veux pas de, non, non.

**C : Donc ça c'est, voilà, ça peut être [quelque chose de bien d'en parler.**

**M :** Ça c'est quelque chose, c'est un des sujets] qui est, donc il faut parler, à mon avis. Parce que le jour où ça arrive, les gens savent pas quoi faire. Et si vous avez… devez être in' intubé, je sais pas si tout le monde, sait ce que ça veut dire. Mais par contre, si vous êtes là, que vous arrivez plus, pis qu'ils doivent le faire, ils le feront. C'est pas là que vous allez dire non, parce que vous saurez pas. Par contre, les choses, il faut les dire avant.

**C : Prendre temps de, [de parler de ça.**

**M :** Ouais. Il faut en, en parler,] à mon avis. Je trouve qu'on doit en parler, mais bon, si certains en veulent pas, en veulent pas, hein. Comme les épinards, si vous les aimez pas, ben vous en mangerez pas.

**C : Donc voilà, et aussi au niveau de, de ce soutien spirituel, ça pourrait être [quelque chose qu'on pourrait investiguer, et pis.**

**M :** Ça aussi ouais, je pense c'est une chose] qu'on pourrait en' il faudrait envisager. Il faut, pourquoi pas, il faut aussi en parler, certains en voudront, d'autres pas, mais, dans l'ensemble, personnellement, je l'aurais, je pense que je l'aurais accepté.

**C : Ouais. Et aussi, comme vous me disiez, on peut vite être isolé aussi, [avec cette maladie. Donc.**

**M :** Oui, oui.] Faire très attention.

**C : Faire attention.**

**M :** Ouais, de faire très attention à, à régulièrement revenir sur le sujet, mais sans être pesant. Pas, parce que sans ça, on vous dit : « mais, allez vous faire voir, je vous ai pas deman', je vous ai rien demandé ». Non mais, discrètement et de, de voir si vraiment les gens se, sont seuls ou, ou s'ils sont entourés, s'ils sont. Vous pouvez être entouré aussi par des disques, par les films, et tout, par n'importe quoi, mais, mais, où vous êtes tout d'un coup dans votre coin, vous laissez écrouler. Ça, moi je trouve c'est un des sujets aussi auquel je ferais attention. Ouais, à soulever.

**C : Oui. Et pis de, de voir aussi pour, pour certains patients qui ont peut-être besoin d'être un peu plus poussés, de proposer [aussi des choses.**

**M :** Ouais, de stimuler les gens, ouais.]

**C : De stimuler.**

**M :** Peut-être un stimulus, mais sans, de nouveau, forcer : « ah c'est à cause de ça que t'iras mieux ». Non, non, c'est pas ça qu'il faut faire. Parce que : « vous me cassez les pieds, j'ai plus envie de vous voir ». Par contre, en parlant, en ayant un, une bonne relation, vous arrivez à faire, faire les, beaucoup de choses aux gens.

**C : [Donc voilà de, de proposer sans.**

**M :** Mais y a un côté]… entretien, y a un côté échange qui va avec certains, pis qui va pas avec d'autres, ça c'est clair. Ça c'est, c'est up-to-date, c'est, ça dépend beaucoup des personnes.

**C : Donc, donc voir selon les personnes.**

**M :** Oui.

**C : Mais de proposer des choses sans que ce soit [pesant pour le patient.**

**M :** Oui, exactement, sans que ce soit une obligation] ou pesant ou contraignant. Ça, vous donnerez jamais rien avec la contrainte... La contrainte, on l'a déjà au bout du fil, donc, le, le reste, les contraintes vous les gardez.

**C : Proposer.**

**M :** Ouais…

**C : Est-ce qu'y aurait d'autres choses que vous aimerez encore rajouter ?**

**M :** Non, non, moi je suis à disposition si, si je peux rendre service, si je peux, faire des ouvertures, c'est avec plaisir. Mais, non, ben trouvez moi un médicament, trouvez moi une, une formule qui me fait revenir à, aux saturnalités. Bon, sans oxygène, je pense pas que ce sera possible. Mais bon, si je pouvais respirer un peu plus, mieux, ce serait génial.

**C : Merci en tout cas, [beaucoup pour ce témoignage.**

**M :** Mais merci à vous d'être venue.] Merci, c'est gentil.

**C : De nous avoir partagé tout ça *(rires)*. [Merci.**

**M :** Merci *(rires)*.]

## Participant n°7

Nom d'emprunt : Paul
Âge : 71 ans
Sexe : M
Groupe : Intervention

**C : Donc vous avez participé à une étude qui avait pour objectif de comparer le traitement habituel de votre maladie pulmonaire, la BPCO, avec une prise en charge précoce, soutenue et intégrée. Vous étiez dans le groupe bénéficiant de cette prise en charge, globale, spécialisée. Et vous avez reçu la visite d'une infirmière une fois par mois pendant un an. Pouvez-vous me raconter comment ça s'est passé ?**

**P :** Ben déjà, personne très, très charmante, cette *Laure Chappuis (infirmière de recherche)*, et qui m'a apporté toute une documentation sur, sur, sur, sur, *(il cherche des documents)* voilà ben tiens y a des photocopies de tout un dossier sur la réhabilitation pulmonaire. Voilà, « mieux vivre avec une insuffisance respiratoire », des graphiques, des... Ils traitent de tout, là-dedans, comment respirer, faire des exercices etc. Ça c'est la première chose. Comme je faisais déjà des exercices à l'hôpital, donc ça m'a pas servi énormément, mais, 'fin c'est toujours intéressant. Ce qu'on a travaillé beaucoup avec *Laure Chappuis (infirmière de recherche)*, c'était sur mes angoisses. Car quand je désature, la désaturation elle est violente. Et là, je panique, tout simplement. Donc on a commencé à travailler un peu sur ce, cette panique. Bon, on peut faire n'importe quoi, on peut chanter, on peut faire tout ce que l'on veut, mais enfin l'essentiel c'est de sortir cette angoisse, de, de, de penser à autre chose, que le souffle où on est en train d'étouffer vraiment. Et moi mon, mon truc, qui a marché très bien d'ailleurs, c'est de me pincer entre le pouce et l'index, me, pas me pincer mais enfin appuyer très fort avec l'autre pouce, d'avoir tellement mal que le mal surplantait au fond cette défaillance pulmonaire. Et maintenant j'en suis arrivé que je, j'appuie là *(il appuie entre le pouce et l'index)*, et je ne sens même plus le, la douleur. Donc ça, ça a été efficace. Et gentiment, ben cette angoisse a un peu passé, j'ai moins paniqué. Parce qu'il m'est arrivé dans la rue aussi, tout d'un coup de ne plus avoir d'oxygène par un problème technique ou, ou que tout d'un coup y avait plus de trams, plus de bus, plus rien, et j'étais en panique avec le bus, avec l'oxygène, et j'arrive à me maîtriser un peu mieux, on va dire. Alors ça, ça m'a vraiment aidé. Et puis je suis toujours en contact, même si le, l'évaluation, 'fin le, le, le test est terminé, je suis toujours un petit peu en contact avec elle, déjà pour entretenir les bonnes relations, donc ça c'est déjà pas mal, c'est humain, on va dire. Ça c'est la première chose. Mais, que vous sachiez que, après cette, ces entretiens, après une année, j'avais de plus en plus de difficulté à respirer. Donc, au début c'était tous les 50 mètres, après, il fallait s'arrêter tous les 20 mètres, après tous les 10 mètres. Donc là c'était plus possible. Et j'avais toujours une douleur au, c'est quoi le *(il se touche vers le haut de l'estomac)*.

**C : Sternum, ou ?**

**P :** L'oesophage ? Oesophage, es' estomac, toujours des douleurs là, des douleurs très, très importantes. Je regardais avec mon médecin traitant, 'fin le *Docteur D.*, que vous connaissez probablement très bien. Et, pour lui c'est plutôt musculaire, mais on n'était pas sûr. Résultat des courses, si vous regardez mon, mon dossier médical, j'ai fait avec le Professeur, le *Docteur E.*, un scanner complet des poumons et de la partie, comment on appelle ça la *(il se touche vers le haut de l'estomac)*?

**C : L'estomac ?**

**P :** L'estomac, ouais, intestins, et tout ça. Tout ça, négatif. Donc, y a quelque chose qui ne joue pas. Qu'est-ce qu'on va faire ? J'ai demandé au *Docteur D.* finalement, on a pris la résolution, de faire, si vous regardez tout le dossier médical, une réduction des, des plèvres, des, des trois, trois bulles qu'il y avait dans le poumon droit. Et là, tout a très mal été, puisque je suis resté trois mois à l'hôpital, juste pour cette bricole. Mais en réalité, je suis resté trois mois, puisque j'ai tout fait, infections, tout ce que vous voulez. Et finalement on a re collé le poumon. Ça s'est mal passé, mais je m'en suis bien sorti. Résultat des courses, trois mois après, ça allait pas trop mal, j'ai pas mal récupéré, mais trois mois après, de nouveau des douleurs, des douleurs terribles ! On refait un scanner, tout était normal, on repart sur l'idée musculaire, mais qu'est-ce qu'on va faire avec ces douleurs ?! Et là, tilt, j'ai repensé à

mademoi' à mademoiselle *Chappuis (infirmière de recherche)*, j'ai essayé de la recontacter, pour voir si elle avait, elle pouvait me donner un, au fond une adresse, ou un contact, pour voir si on pouvait faire quelque chose pour ces douleurs. Et effectivement, elle m'a mis en contact avec, *(il cherche dans ses papiers)* avec, avec… ces braves gens de, de, voilà, ces braves gens de la X *(service)*, *(lieu)*, avec le *Docteur F.* (*soins palliatifs)*, le *Docteur* C. (*soins palliatifs)* et le *Professeur B. (soins palliatifs).* Et enfin ils sont venus ici à la maison, pendant trois ou quatre fois. Et on a fait un petit traitement à la morphine. On a fait la morphine, deux milligrammes, donc rien du tout, aucun effet. On a dit : « on va passer à trois milligrammes ». Et avant de commencer à trois milligrammes, j'ai eu, je sais pas pourquoi, l'idée d'aller consulter mon cardiologue. Et mon cardiologue, après un, comment dire, un électrocardiogramme, il me dit ben : « vous pouvez rentrer immédiatement à l'hôpital d'urgence, car vous êtes à 160 de, de pulsation ». Donc, un petit peu un échec là. Donc je suis rentré 15 jours à l'hôpital, c'était cette année, 2016, au mois de juillet. On a régularisé tout ça, mais comme par hasard, toutes les douleurs ont disparues. Chose incroyable, personne ne, ne l'explique. Résultat des courses, je n'utilise touj' pas ce, cette morphine, et je veux l'utiliser le moins possible, parce que j'ai deux versions, j'ai une version qui me dit : « ce n'est pas addictif ». Et une autre version que c'était addictif. Alors, pour le doute, je préfère, et, le peu de douleurs que j'ai, c'est tout à fait acceptable. Maintenant, à l'heure actuelle que je vous parle, la respiration elle devient de plus en plus difficile, le *Docteur D.* est tout à fait au courant, et je crois que c'est une suite logique des choses. Tous les jours je m'efforce de faire une heure de marche à peu près, le plus possible. Et pis je crois que c'est le seul moyen. Point de vue nourriture, pas de problèmes. Le poids s'est stabilisé. N'oubliez pas que, en 1900', je crois en, en, non, en 2012, j'étais à 45 kilos, et maintenant je suis à 70 kilos. Donc, j'ai bien repris, et ça me fait des réserves au cas où il se re passait quelque chose. Donc, ben j'ai une confiance totale à *Docteur E.*, au chirurgien *Docteur G.*, qui m'a fait, m'a opéré deux fois. Et on est toujours en contact, ils regardent toujours mon dossier médical, ils ont toujours un nez dedans, parce que ils estimaient que j'avais vraiment des, des, un cas un peu, un petit peu particulier, ils ont même pas eux-mêmes compris ce qu'il se passait.

**C : Pour la douleur, et ?**

**P :** Non, même pas, mais pour le, la respiration. J'avais une fuite dans les poumons, et on ne savait pas d'où venait cette fuite. On m'a mis des, des valves, deux valves, on m'en a retiré, on m'en a remis. Mais ça a rien fait. On pensait que les douleurs venaient de ces valves, y avait une inflammation des valves, finalement pas du tout. On cherche quoi. Mais je crois que c'est l'évolution à peu près normale de la chose. Pour votre information, je vais toujours, à toutes les réunions de la ligue pulmonaire, avec le *Docteur D.*, tous les mois là au, à une petite réunion où c'est toujours, moi j'ai toujours été preneur des choses qui pouvaient améliorer ma santé. Donc, chaque fois qu'y a quelque chose je, je suis preneur, évidemment. Dans l'immédiat, j'ai pas trop d'autres choses, à moins des choses, plus précises, je ne sais pas.

**C : Ouais. Donc ouais, y a beaucoup de choses qui se sont passées depuis [ces visites.**

**P :** Oui beaucoup, beaucoup, beaucoup, beaucoup.] Deux fois, je suis retourné deux fois, trois mois, trois mois et demi d'hôpital, en deux fois.

**C : Ouais, ouais. Donc, beaucoup, beaucoup de choses. Et là, vous avez donc [recontacté, cette infirmière.**

**P :** Ouais, et pis, oui. ] D'ailleurs, elle m'avait vu cette infirmière, elle m'avait vu à X *(hôpital)*. Elle m'a dit : « qu'est-ce que vous foutez là monsieur X *(nom)* ? » Bon, il faut dire que j'ai tout l'air, qui passait, je sais pas, l'emphysème, on appelle ça je crois, qui passe sous la peau. Donc, pendant, xxx, j'étais un rat, je voyais plus rien, je pouvais plus manger, plus rien. Tout l'air passait de, sous la peau, et ça fait *(son)* comme ça.

**C : Ça, ça gonfle.**

**P :** Ouais, ça gonfle. Pis après fallait retirer ça. Ouais, et on avait toujours pas réglé le problème. Donc, ça c'est angoissant aussi. Beaucoup de choses comme ça angoissantes. Mais, j'ai surmonté. Et c'est ça, c'est peut-être grâce à mademoiselle *Chappuis (infirmière de recherche)* finalement.

**C : Ça, ça a aidé justement à.**

**P :** Oui, oui, tout à fait.

**C : Vous avez dit, qu'elle vous a aidé avec les, les angoisses notamment, [et.**

**P :** Ouais les angoisses,] surtout ouais. Et relativiser, se décontracter, patati. Et maintenant je reprends encore un peu de, je fais un peu de physio, mais, pour les, comment on appelle ça, les tra', le trapèze, enfin, pour me soulager un peu, parce que c'est, tout est étendu, c'est *Docteur F.* qui m'a dit qu'il fallait le faire, cette xxx. Alors je le fais.

**C : Ouais. Donc, en tout cas quand elles sont venues, [une fois par mois, ça.**

**P :** Ouais, trois.]

**C : [Une fois par mois pendant un an.**

**P :** Ah, une fois, ouais, ouais.]

**C : Là, vous aviez aussi reçu des informations, donc, [sur la BPCO.**

**P :** Tout, tout ce que je voulais] comme informations, je les avais. Mais bon *(soupir),* sauf le principal, l'oxygène, ou rayer le, la maladie, mais bon, ça on ne peut pas.

**C : En tout cas en termes d'informations, vous auriez pas, [voulu quelque chose d'autre ?**

**P :** Non, non, non, non.] Et j'avais toujours aussi un contact, comme j'étais très souvent à l'hôpital, avec *Claire (infirmière de recherche)* aussi… Donc, oui, oui, je savais à qui m'adresser là. Bon, ce qui m'intéresse, c'est de ne pas passer à la morphine, maintenant. Maintenant, où, où je vous parle, j'ai, j'ai xxx mal. Et c'est pas que j'ai mal juste là, j'ai mal sur le flanc ici *(il montre le côté latéral de son corps)*, c'est ça qui est bizarre. Et comme m'a fait, m'a, m'a fait remarquer le, le cardiologue, il m'a dit, mais moi j'ai pensé aussi que c'était le coeur qui pouvait emmerder. Il m'a dit : « pas du tout, le coeur en tout cas pas. Mais comme par hasard, c'est depuis que vous avez été, fait ces opérations ». Y en avait quand même quatre de ces opérations, c'est depuis ce moment-là que j'ai eu des douleurs très, très prononcées. Et tout d'un coup elles ont disparues, inutile de dire que… ça soulage un peu. Et ça favorise aussi pour marcher, hein. Ouais.

**C : Ouais. Mais là, les douleurs sont un peu revenues ?**

**P :** Très légèrement. Et, maintenant, je m'appe', avant, quand y avait *Laure Chappuis (infirmière de recherche),* j'étais à un litre, la journée au repos. Et deux litres, ou trois litres quand je marchais. Maintenant, je suis minimum à deux litres à la maison, au repos. Et je passe à trois litres, voire quatre litres, à l'extérieur en marchant. Donc, ça se dégrade. Ouais. Mais je gère, je gère et, mon, comment on appelle ça le *(il touche le bon d'un de ses doigts)*.

**C : [La saturation**

P : ma saturation], ouais, elle est toujours entre 92, 93, 92, 93 ouais, à peu près, comme ça au pif. Mais, quand on me la prend, je dis : « maintenant je suis à 92, 93 ». Parce que on commence à connaitre son corps, après cinq ans hein. Ça fait cinq ans que j'ai la *(il tire sur le tuyau à oxygène)*.

**C : L'oxygène.**

**P :** L'oxygène en continu.

**C : Ouais, ouais, ouais. Et là, justement, en terme de gestion des, des symptômes physiques de la BPCO, de la difficulté à respirer, comment les visites de, de l'infirmière une fois par mois, ou les informations que vous avez reçues, ont pu aider ou non, à gérer ces symptômes physiques ?**

**P :** Gérer les symptômes physiques *(rires)*. Bon, déjà il faut déjà apprendre à respirer. Jamais personne, en réalité vous apprend à respirer. Vous êtes à l'hôpital avec une pneumo', vous sortez,

ben vous allez faire quelques exercices, mais personne vous apprend à respirer en réalité. Personne vous *(toux)*, vous dit de, en faisant un effort qu'il fallait expirer, inspirer avec la bouche, fermer la bouche, faire avec le nez etc. Personne vous le, le dit. Vous le découvrez avec les autres, ou en discutant avec les autres. D'où, par exemple à la ligue pulmonaire, on va discuter un peu de ça. Ça c'est important. Et, alors avec *Laure Chappuis (infirmière de recherche)*, elle disait : « faut faire les choses beaucoup plus calmement ». Parce que j'ai toujours été très nerveux, aussi toujours un stress permanent, pendant les années, toute ma vie en gros. Donc là il fallait vraiment tirer un trait, et reprendre les choses calmement. Et ça aide, ça c'est sûr.

**C : Ça, elle a pu vous aider un peu, avec [ça ?**

**P :** Ça.] Ouais, et on revient toujours sur le, l'angoisse, sur l'anxiété, le, ouais, ça elle m'a aidé.

**C : Oui. Et de travailler là-dessus, d'où l'envie de la rappeler, aussi après ?**

**P :** Oui, j'avais son numéro de téléphone. Et puis si je l'avais oublié, je regarde avec *Claire (infirmière de recherche)*), pis *Claire (infirmière de recherche)*, y avait tout le tam-tam qui se, tout le monde se connaît quoi, aux X *(hôpital)*, voilà.

**C : Ouais, pis vous avez aussi ces, ces réunions donc, aussi avec la, la ligue pulmonaire, [où vous pouvez échanger.**

**P :** Voilà, donc y a,] y a l'infirmière, le *Docteur A*. *(pneumologie)* les connaît bien. Et pis, y a aussi un psychiatre, le *Docteur H. Docteur H.*, y a toujours *Docteur H.*, le psychiatre, le pneumologue *Docteur D.*, et une infirmière, X *(nom)*, par exemple. Ouais, alors donc, avec eux tac on sait tout de suite, comment, qui fait quoi, comment, où, à qui se renseigner. Oui, oui.

**C : Vous pouvez échanger des informations [avec les autres.**

**P :** Voilà, par exemple,] sur la vaccination des pneumocoques, et des choses comme ça. Et c'est important ! Y a beaucoup de gens qui ne savent pas, qui ont jamais entendu parler. Nous, dans le groupe bien sûr, tout le monde l'a, mais en règle général, y a beaucoup de gens qui ne, qui ont aucune idée que ça existe, même !

**C : La vaccination.**

**P :** Ouais, ouais, pour la grippe, bien sûr. D'ailleurs la grippe, moi je vais la faire le, le mois prochain.

**C : [La vaccination pour la grippe ?**

**P :** Mais directement à la réunion, tac.] Ils piquent, et pis c'est fait. Parce qu'il faut pas le faire trop tôt. Vous saviez ça ? C'est une, c'est un mois de, un mois de, d'incubation. Et en général, c'est, c'est valable pour trois mois. Donc, comme les pics de, de grippe, c'est plutôt janvier, février, y a pas intérêt à la faire maintenant, maintenant c'est novembre, décembre, janvier, pis février vous êtes plus couvert, donc faut vraiment mettre les, les choses de son côté. Ce que j'ai appris aussi surtout alors se désinfecter les mains avec les fameux petites fioles-là. Ça c'est vachement important, quand même. Je, j'essaie de mettre les choses de mon côté, quoi !

**C : Ouais, ouais. Ça vous disiez que, vous [cherchez l'aide, dont vous aviez besoin.**

**P :** Je peux rien faire d'autre. Oui.] Et je, j'ai, j'ai toujours ses téléphones, je peux l'appeler, elle me dit : « ouais ». Même si elle fait plus, elle est plus dans le service, etc. Mais, elle sait à qui me, me connecter. [xxx.

**C : Oui. Et, qu'est-ce qui vous a] plu ou déplu en particulier dans, dans ces visites une fois par mois ?**

**P :** Non, rien de spécial. Non, ça me dérangeait pas ! A partir du moment où j'ai accepté disons de, de faire le machin, je me soumets, y a aucun problème. Pis bon, je suis pas obligé d'aller bosser, rien du tout, j'ai, j'ai que ça à faire, non.

**C : Mais, là y avait pas quelque chose en particulier [que vous avez apprécié ou quelque chose que vous auriez voulu en plus ?**

**P :** Non. Elle-même], *Laure Chappuis (infirmière de recherche)* a travaillé aux, ou était rattachée aux, aux soins palliatifs à X *(hôpital)* à euh.

**C : A X *(lieu)* ?**

**P :** Non, non, à X *(hôpital)*.

**C : Ouais.**

**P :** Le X *(hôpital)*, donc ça voulait dire mort. X *(hôpital)* aussi, c'est la mort, en général. Et là, elle a, m'a quand même expliqué qu'y avait pas <u>que</u> la mort, y avait beaucoup d'autres choses qui se faisaient. Y a des gens qui venaient se reposer, à la limite. Donc ça rassure aussi un petit peu, bon. xxx et pis en cas de, ben voilà, comment ça se passe un peu, quoi, j'ai une idée.

**C : Vous avez pu discuter un peu de ça.**

**P :** Ça vous prépare gentiment, vous tombez pas des nues. Ouais !

**C : Vous avez pu, je sais que parfois on discute des, des soins qu'on aimerait aussi en fin de vie, est-ce que c'est quelque chose ?**

**P :** Ah ben, non, ça, ça, ça a toujours été pas d'acharnement, quoi. D'ailleurs j'ai fait le choix 10 jours aux soins intensifs, et là je voulais claquer, quoi, je, j'avais ras-le-bol… Ça allait mal, on va dire. Et je dois remercier encore, puisque le *Professeur A*. *(pneumologie) (il regarde l'enregistreur et s'adresse à l'enregistreur)*, je, si vous m'écoutez bien, je dois, je, j'ai vraiment une, presque une <u>dette</u> envers le *Docteur E.*, *Docteur G.*, et *Docteur D.*, qui ont trouvé le <u>temps</u>, alors vraiment, qui ont trouvé le <u>temps</u>, et comme ça au milieu de la semaine, de se réunir à 14 heures aux soins intensifs, et puis voir ce qu'il se passait, parce que moi je voulais plus vivre, je voulais arrêter de vivre, j'arrivais plus à respirer, c'était l'enfer ! Et, y avait des fuites, j'avais des fuites dans le poumon, qu'est-ce que vous voulez faire ! Et c'est *Docteur G.*, il m'a dit : « on n'a jamais vu quelqu'un qui est sorti de l'hôpital de X *(lieu)*, avec des fuites dans un poumon ». Il m'a remonté un peu le bourrichon, les trois m'ont remonté le bourrichon, ils ont fait l'effort déjà de venir me voir, et m'expliquer tout ça. Et là, je dois les remercier, vraiment. [Ouais.

**C : Ça a été,] un bon soutien de ces médecins.**

**P :** Ah oui, ouais, ouais, ouais mais c'est exceptionnel, quand même ! C'est pas un petit généraliste ou comme ça, qui vient. C'est quand même des gens de très haute, très haute qualité.

**C : Qui se sont mobilisés.**

**P :** Ouais, ouais. Mais maintenant, quand je les vois à l'hôpital, ils me disent : « on est toujours vivant ? On a passé tout près ». Ben oui, mais je m'en rendais pas compte. Et après je déconnais complètement, parce que, les médicaments étaient <u>hyper</u> violents. Voilà.

**C : Quand, quand vous étiez en soins intensifs.**

**P :** Oui, et après, malheureusement, je crois que j'ai, j'ai, je les ai <u>cumulés</u>, j'en ai mis des <u>couches</u> ! Après j'étais aux soins intermédiaires, mais c'est les soins intermédiaire au sixième. Et là j'ai deux personnes, qui sont décédées devant moi, là à 10 mètres, moi je suis pas habitué. Ça m'a <u>complètement</u> perturbé. Et j'ai eu peur, j'ai pris peur, un peu. xxx la nuit, y a des ombres, alors vraiment je <u>pataugeais</u>. Et, on m'a transféré, on m'a mis dans les, dans les étages classiques, et là j'ai tout de suite récupéré. Parce que j'ai, c'était négatif ces soins intermédiaires. Moi je suis pas habitué, enfin je crois que personne est habitué à ce genre de situations. Mais là j'ai eu *(sifflement)*, y a même ma femme aussi, qui est venu me voir, elle me dit : « il est en train de mourir lui ». Vous savez ça fait drôle. Ouais. [Alors j'en ai, j'en ai mis des.

**C : Ça fait peur.]**

**P :** Oui, oui ça fait peur. J'en ai mis des couches dans tous les sens ! Ouais. Pis je m'en suis sorti. C'est, c'est pas mal. Maintenant on va voir jusqu'à quand.

**C : Pis là, comme vous disiez, vous avez pu parler un petit peu de, justement la mort, ou de, des soins en fin de vie, que ça [préparait un petit peu.**

**P :** Oui, bon,] moi y a longtemps que je sais hein, que c'est une maladie évolutive, une saloperie, quoi. Ça va pas aller en s'arrangeant. Maintenant, on va essayer de le, la freiner, pis voilà, y a que ça à faire. Et aussi une, une chose que je suis arrivé à faire, c'est de pas penser sans arrêt à cette maladie. Ça c'est fondamental. C'est le psychiatre, *Docteur H.*, qui me demandait si j'arrivais bien à dormir, non j'arrive pas à bien dormir, toutes les heures, les heures et demies, je suis réveillé. J'ai des, mal sur les flancs etc. Je dois me tourner. Et il me dit : « quand vous arrivez pas à dormir, vous pensez à quoi ? » Evidemment. Eh bien, tout ça, j'ai pu l'évacuer, de plus penser à la maladie, mais plutôt à des conneries, à des witz, à des, à des boutades, à des. Et c'est une forme, pour moi de, d'évacuer, au fond. Et c'est ce qui me sauve un petit peu. Et tout d'un coup ça me rappelle à l'ordre, quand j'ai quelque chose, je me dis : « mais, attends ». Ouais. Mais.

**C : Essayer de ne pas trop y penser tout le temps.**

**P :** Ouais. Ah, si on y pense, on fait que parler de ça, c'est, ça devient un peu pesant. Oui. [Trop pesant.

**C : Donc là vous avez pu avoir un peu des ast'.]**

**P :** Imaginez vous avez quelqu'un en face de vous qui vous parle sans arrêt, sans arrêt, sans arrêt de ses malheurs, de sa mort, de *(grommellement)*, non mais c'est, on devient fou, l'autre devient fou ! *(rires)* On devient tous fous ! *(rires)*. Alors voilà.

**C :** ***(rires)*** **Essayer de penser à d'autres choses.**

**P :** Ouais. De plus gai !... Et pis bon, toute, bien sûr l'organisation de la vie, y a tout qui a explosé, tout a, tout a changé, quoi, évidemment. Je voyageais énormément, maintenant plus de voyages. Alors au lieu d'aller, je fais venir les gens, c'est déjà, c'est pas mal ! Mais bon.

**C : Des, des changements.**

**P :** Ah oui, radi' radicaux, hein, radicaux !... Mais heureusement, comme je disais toujours ou souvent à des personnes, heureusement qu'on a profité avant les 65 ans. Parce que si on dit à 65 ans, parce que c'est à 65 ans que ça allait mal, que j'arrivais plus à remonter les étages, j'ai dit : « maintenant je vais me soigner ». Et c'est là que c'était foutu, quoi, parce que, c'était trop tard. Donc.

**C : Vous avez profité avant.**

**P :** Oui. Et sur le champ, j'ai arrêté de fumer. C'est, c'est dû à la fumée, évidemment, sur le champ, et maintenant je suis complètement dégoûté, pas question, ça fait cinq ans que j'ai arrêté. Eh ben sur le champ là, tac !

**C : Vous avez pu arrêter de fumer.**

**P :** Ouais. J'ai tenté avec le psychiatre de, de la ligue pulmonaire, *Docteur H.,* il m'avait suggéré de faire une, des séances d'hypnose, pour essayer de décontracter, et ça a pas marché. Je, bon, faut dire, entre guillemets, le, je sais pas, y avait une relation qui était pas au top, disons, et ça, ça favorise pas non plus. J'ai utilisé l'hypnose, deux fois pour arrêter de fumer, ça a marché très, très, très bien, je me souviens plus le docteur. Là, ça a très, très, très bien marché. Je me suis dit : « pourquoi pas, j'essaie ! Si c'est un, ça peut apporter un bien, je, je le fais ». Mais là, ça, ça a pas marché, ça a pas marché, pis c'est tout, et c'est pas grave.

**C : Ouais. Et, comme vous disiez, là aussi par rapport à vos proches. Vous faites venir des gens ici.**

**P :** Oui, oui.

**C : Vous avez, quel type de soutien vous avez de la, de la part de vos proches ?**
**P :** Ah ben, eux ils me remontent le bourrichon ! Il me dit : « on va quand même, tu vas pas quand même pas mourir cette année, ou bien quoi ! » Bon, c'est des X *(origine)*, hein, c'est, ils viennent tous de X *(pays)*. Et, justement hier je les ai eus au téléphone, ou avant-hier. Evidemment, ils me, ils me remontent le bourrichon, quoi, hein. Et bon ben, je les aurais au moins amené par-là *(rires)*. En 2018, ils vont mettre par-là, donc c'est encore un motif, voyez pour, essayer de crocher. Arriver à ce 2018, pour les revoir encore un petit coup. Parce que je me fais pas d'illusions non plus. Alors, c'est, c'est, c'est un peu des choses, oui très souvent oui, une personne très, très âgée, et la petite-fille qui va accoucher, il veut pas mourir avant avoir <u>vu</u> cette petite fille, ou ce, ce, ce bébé. C'est un peu ça, au fond.

**C : Des choses qui font s'accrocher.**

**P :** Ouais, ouais, il sont marioles là-bas *(rires)*.

**C : *(rires)*. Mais c'est de la famille d'X *(pays)*, ou ?**

**P :** Pas du tout, des amis. C'est tout. Et, ça fait 40 ans, enfin non, ah ben oui de, 75, 1975, alors ça fait une quarantaine d'années, quoi, ouais. Et des gens que j'ai connu, qui faisaient du stop *(rires)*. C'est fou hein *(rires)* ! Non, c'était bien, c'est bien.

**C : *(rires)*. Et de, quel type de, d'autre soutien de la part des proches, auriez-vous eu besoin ?**

**P :** Non, j'ai très peu de proches. J'ai plus de famille. Ma femme non plus, elle a juste une, une, une soeur et un frère, et pis c'est tout, quoi. Bon ben, avec le temps, tout diminue. Et on n'a pas d'enfants non plus, donc. Mais bon, c'est comme ça, on se fait, on s'y fait !

**C : Et ces, ces visites de, de l'infirmière, comment ça s'est passé pour, pour votre femme aussi, qui était là ?**

**P :** Ben, elle pouvait assister, elle a eu assisté, d'ailleurs. Au début, pis après bon, elle l'entend toute la journée, alors. Mais, ou des fois, si je vous dis une connerie, maintenant je suis sûr qu'elle l'entend, elle réagit : « non, c'est pas vrai ! » Ou : « c'est pas autant que ça ! » ou voyez. Parce que des fois on a tendance à exagérer aussi. Mais, non, non, elle, elle, elle est dedans, elle me surveille toujours, parce qu'elle sait ce qu'il se passe. Alors, entre parenthèses, ce que le *Docteur A. (pneumologie)* va pouvoir voir, sur mon dossier médical, c'est qu'entre-temps j'ai de nouveau une bulle, qui s'est reformée dans le poumon droit, et qui probablement va bouffer les petites bulles. Et, maintenant y a plus rien à faire, donc ça, ça pue un peu le pneumothorax, tout ça. C'est-à-dire, avec un coeur qui allait 150, 160, bon maintenant qui est stabilisé, mais avec cette bulle, qui risque d'exploser. Donc, moi les exercices <u>trop</u> violents, <u>trop</u> physiques, j'ose pas trop. Et je prendrai plutôt peur, pour sortir.

**C : De, sortir de ?**

**P :** Pas de, de la maison, parce qu'à X *(ville)*, c'est un gros village, y a aucun problème. Mais, vous me dites d'aller dans le X *(région)*, même accompagné etc. *(Soupir)* voilà, même, même avec des bonbonnes, même avec tout ce que vous voulez, mais, y a toujours le doute. Alors ça, j'ai des réticences, maintenant. Je suis invité un peu partout, mais j'y vais pas. Ouais.

**C : Des, des réticences à, à aller plus [loin.**

**P :** Oui,] ben si le, si la bulle elle pète, donc le poumon il est *(sifflement)*, et y a quoi, en gros une heure pour aller à l'hosto. Si je suis coincé sur l'autoroute, en X *(pays)*, parce que y a je sais pas quoi, il se passe quoi ? Voyez, c'est, c'est des, des sources d'angoisse. Ouais, parce que l'air c'est fondamental, hein !

**C : Ouais, ouais. Et pis l'exercice physique aussi ça.**

**P :** Ah ben ça tue ouais, ça aide, ça, ça. De toute façon, vous vous levez le matin fatigué, parce que le fait de respirer, sans arrêt, sans, de chercher l'air, vous, vous épuise. Toute l'énergie passe là-dedans…

**C : Vous me disiez aussi au début, maintenant qu'y a trois exercices physiques, [c'est ça ?**

**P :** Trois, trois] fois par semaine. Moi j'ai renoncé, j'ai été, j'ai fait deux fois par semaine, pendant trois ans, c'était déjà pas mal, c'était déjà pas mal. Parce que *(il regarde l'enregistreur)*, si vous faites déjà deux fois par semaine le, les exercices physiques, vous avez le pneumologue, vous avez le, le médecin traitant, vous avez le cardiologue, vous avez le dentiste, il manque plus que le gynécologue *(rires)* ! Non, mais, j'en ai ras-le-bol des, comme on dit des blouses blanches. Bon, y a certains, on se fatigue, c'est vrai.

**C : Ça fait un peu beaucoup.**

**P :** C'est énorme, c'est énorme. Ouais, vous êtes sans arrêt là-bas... Alors maintenant, ce que je fais, donc je fais <u>ma</u> physio moi-même. J'ai émis la possibilité, et je crois savoir, d'après l'entretien que vous avez eu avec monsieur, avec le *Docteur D.*, il semblerait que, <u>peut-être</u> que l'année prochaine, y aurait un deuxième groupe pour venir <u>une</u> fois par semaine. Alors là, une fois par semaine, je crois que je, je serais présent. Ça fait quand même du bien. Une fois, pis ça permet aussi de sortir, de se changer la tête hein. Mais trois fois, vous oubliez, vous oubliez. D'ailleurs, une de ces braves jeunes filles, m'avait un peu <u>forcé</u> sur la, la vitesse du tapis etc. etc. Je lui ai dit : « vous m'avez dégoûté là. J'ai qu'une chose, j'ai envie de m'asseoir et plus jamais revenir ». Vous étouffez, vous étouffez, vous, ça va pas, si c'est bénéfique, alors aucun problème, je prends. Mais dès que ça devient, c'est ça qui est… Et puis, une chose aussi que, où je me bats maintenant, c'est que, j'essaie le plus possible de décourager les gens à fumer, ça aussi. Et, y en a pas mal qui écoutent, parce que ils commencent à avoir peur en voyant l'oxygène, le tuyau qui. J'ai dit : « oui, ça fait cinq ans que je me traîne avec ce tuyau ». Alors ça leur fout un peu les jetons, et y en a, là dans la maison, y en a déjà deux qui ont arrêté *(sourire)*. Alors c'est déjà pas mal hein.

**C : Ouais. Vous les, vous les motivez *(rires)*.**

**P :** Ben, voilà. Ils voient que c'est, c'est lourd, c'est lourd.

**C : Ouais. Avec l'oxygène, et. Et, donc… Donc oui vous m'aviez dit aussi que, vous aviez pu parler un peu avec l'infirmière des, des soins en fin de vie, ou ça c'est quelque chose que.**

**P :** Des ?

**C : Des soins en fin de vie, est-ce que c'est [quelque chose ?**

**P :** Des soins palliatifs.]

**C : Voilà, des.**

**P :** Oui, on a discuté un peu de, de, X *(hôpital)*. Parce que, bon, pour moi c'est du chinois, je connais rien du tout là-bas. Et, comme elle-même était là-bas, elle travaillait là-bas. Elle faisait les nuits, les week-ends etc. Elle m'a expliqué un petit peu comment c'était, quoi. Et bon, comme ça j'ai une idée.

**C : Ouais. Parce que, je sais que parfois on parle aussi des directives anticipées, je sais pas si vous avez ?**

**P :** Je sais pas.

**C : Voilà, c'est, on, on peut écrire ce qu'on [aimerait comme soins en fin de vie.**

**P :** Ah ouais, ouais, ah oui, oui, j'ai entendu.]

**C : Voilà.**

**P :** Non, je veux pas d'acharnement, point, fini, terminé. Le problème c'est pas quand on va mourir, c'est comment hein. Personne, personne ne veut souffrir hein. On reste raide sur une table d'opération, on s'en fout, on sent rien. Mais, souffrir, c'est ça qui fait peur à tout le monde, je crois. Alors elle me disait, y a des moyens pour, etc. etc. Bon.
**C : Bon au moins, vous avez pu avoir un peu [une idée, en discutant.**

**P :** Voilà, une idée, une idée, une idée,] oui.

**C : Voilà. Mais vous avez pas forcément [formalisé par écrit ?**

**P :** Non, non, non,] rien du tout, rien, strictement rien… Mon idée a toujours été, depuis tout petit, depuis gamin, être suicidaire. Je suis suicidaire, et j'ai toujours rêvé d'une mort violente. Et évidemment avec ça, ça fout tous mes projets en l'air *(rires)*. Et je dois dire, avec ces stages à l'hôpital, j'ai une autre vision de la mort, un peu... Il arrive un moment je crois qu'on choisit plus, on est sous médicaments, on est complètement à côté de la plaque. On sait même plus comment on s'appelle, alors je crois que on laisse aller comme ça, pis fini. Je, je veux même plus me poser la question. Je préfère penser à d'autres, à des conneries, que penser à ce genre d'histoires, quoi.

**C : Ouais, ouais. Donc ça, ça a changé votre vision de la mort.**

**P :** Un petit peu, ouais, un petit peu, ouais. Mais bon, c'est comme ça.

**C : Et je sais qu'aussi on, on peut proposer du soutien spirituel, est-ce que c'est quelque chose ?**

**P :** Je l'ai eu à l'hôpital avec les aumôniers. Je suis athée, donc, en toute franchise. Mais par contre, j'ai eu une aumônière, qui était, mais charmante, une X *(origine)*, vraiment adorable, cette femme. Et, d'ailleurs quand je vois sa copine, je lui dis : « ouais, vous la saluez bien ». Parce que, vraiment, elles ont des, il faut le faire ce qu'elles font quand même, ouais. On parle de tout et de rien mais, y a un soutien aussi. Ouais.

**C : Oui. Donc ça y a eu à, à l'aumônerie.**

**P :** J'étais *(rires)*. Y en a une que je chicane toujours, c'est X *(nom)* de la ligue, elle est aussi X *(origine)*. Et, j'étais aux soins intensifs, j'étais *(sifflement)*, on savait pas, j'étais sur le fil. Et qui c'est qui se pointe ? Ma X *(nom)*, avec l'aumônière. J'étais, elle vient me présenter sa copine *(rires)*, l'aumônière ! J'ai dit : « ouais pourquoi ? Pour l'acte de contrition ? », non mais j'étais même plus capable de, de discuter, ou. Et après je l'ai revu cette femme, et vraiment charmante. Tous des gens qui ont eu des gros problèmes dans les années 70, avec, X *(homme politique)*, tout le tralala. Et on a un peu discuté de ça, parce que je suis un peu passionné de ça. Et, ouais, ouais, super bien... Mais.

**C : Ouais. Donc ça, vous avez pas forcément eu pendant les visites de [l'infirmière à domicile ? Vous avez discuté un peu de ça.**

**P :** Non, non, non, non.] Ça, ouais, ouais à l'hôpital. Non, non, avec elle, non. Elle, c'était plutôt technique, plutôt, ouais technique, quoi au fond, comment gérer.

**C : Donc plus des informations comme dans [les documents, et, que vous avez.**

**P :** Ouais ! Par exemple elle], je fais quoi xxx. attendez, je vais sortir, c'est, c'est des machins, c'est des. *(Il cherche des documents)* Vous avez déjà vu ça ? Jamais vu ?

**C : Pas ceux-là, non.**

**P :** xxx… *(il lit des documents)* « suggestions pour réduire les soufflements ». Voyez ? Et là on en reparle. Tac, tac, voilà, comment, « positions pour réduire l'essoufflement ». Mais ça, jamais personne me l'avait dit. Elle, me l'a sorti. J'ai dit : « ouais, ouais, je ferai des photocopies ». C'est quoi, un machin canadien. Enfin disons, on a une idée, quoi.

**C : Donc, ça c'est quelque chose qui a, qui a pu être utile pour vous ?**

**P :** Apparemment. Peut-être pas, mais sûrement xxx hein. Comment faire les exercices, etc. Y a des exercices à faire *(il cherche dans les documents)*. « Anxiété », voyez, « essoufflement ; anxiété ; diminution des activités ; dépression ; isolement social ». Enfin bon, ils traitent un peu de tout hein. « La marche, comment faire, adapter les vêtements ». C'est des côtés, des choses pratiques. Mais c'est bien !

**C : Ça c'est, c'est quelque chose que vous avez apprécié.**

**P :** Ben oui, je les ai toujours là hein *(rires)*. *(Il parcourt les documents)* Y a des dessins xxx, suggestions… Voilà, comment utiliser les, les humidificateurs, pour avoir le, l'humidité. Elle m'a donné, voyez ? *(il montre des brochures).*

**C : Brochures.**

**P :** Elle me donnait les brochures. Maintenant, dernièrement, c'était la morphine. Oui, donc, y a, y a une, y a une information, il se fait quelque chose, ça c'est clair, on vous explique tout là.

**C : Donc ça, comme vous m'avez dit, en terme d'informations, vous auriez.**

**P :** Y a aucun problème.

**C : xxx faire quelque chose d'autre ? [Vous auriez pas ?**

**P :** Non.] Comment, qu'est-ce que vous voulez sortir de plus ? Voilà vos directives anticipées, voilà *(il sort la brochure sur les directives anticipées)*. J'avais même pas, xxx « vos valeurs, vos convictions ». Mais bon, ouais, on en a parlé, aussi. Bon après, xxx *(il finit de ranger sa pile de documentation)*.

**C : Quelle a été pour vous l'utilité de ces, de ces visites de l'infirmière ? De cette prise en charge globale spécialisée sur un an ?**

**P :** Bon j'ai appris quelque chose, ça c'est sûr, on apprend toujours. Mais bon, est-ce que ça, c'était hyper bénéfique ? Enfin moi, pour moi, c'est l'angoisse, oui. Moi c'est l'angoisse, qui était dét' qui était un peu déterminant de cette histoire-là. Ouais. Baisser cette anxiété, ne pas paniquer… Ouais pas paniquer quand y avait cette saturation. Parce qu'à un temps, quand je désaturais, à l'hôpital, donc quand je dis désaturer, c'est passer à 70, hein, pas à 92, 93, c'est 70, c'est bas. Je me voyais, par exemple, monter les murs, monter les murs, j'avais les ongles pleins, enfin les bouts des doigts pleins de sang. Et j'essayais de monter ce mur pour arriver en haut, pour aller chercher de l'air. Voyez c'est, c'est, c'est presque des hallucinations ça, c'est presque. Et ça, tout ça, ça a disparu. Voilà ce que ça a apporté. Et ça c'est important. Parce que c'est angoissant ! Ouf !

**C : Mieux gérer cette angoisse, ces anxiétés.**

**P :** Ah ben vous voyez, *(il inspire bruyamment)* xxx respirer.

**C : Ouais. Aller chercher de l'air.**

**P :** Chercher de l'air, tout simplement. Oui, parce que vous, vous vous levez le matin, vous pensez pas comment vous allez respirer. Moi je suis au lit, je sais déjà que ça va aller un petit plus difficile aujourd'hui, ou demain, enfin que le jour précédent etc. Je le sens tout de suite, ouais… Et pis, mais je m'efforce d'aller toujours devant. [Et on me force mon.

**C : D'aller marcher une heure par jour.]**

**P :** L'entourage et les amis qui me forcent, ils me, y a des jours bien sûr, je suis mieux sur le canapé, hein. Quand il pleut, tout ça, mais ils me forcent, ils me forcent, ils me forcent, ils me forcent. Et puis ils me disent : « viens on va changer la tête ! », aussi c'est important.

**C : Changer.**

**P :** L'ambiance, ouais, de voir d'autres tronches.

**C : Ouais. Se changer les idées.**

**P :** Eh ouais.

**C : [Mais ces amis qui vous.**

**P :** Mais dès que] j'ai un problème quelconque, je, je saute sur le téléphone chez *Docteur D.*, quoi.

**C : C'est le Docteur, ouais.**

**P :** Docteur, Pneumologue. Et qui me dirige soit aux urgences, soit chez lui. Et lui-même, se déplace, en plus. Mais, ouais, ouais, pis les relations sont excellentes.

**C : [Avec le Docteur.**

**P :** Et je crois] que c'est très important, ça. Parce que si vous avez pas confiance en celui qui vous soigne alors. Et il prend le temps d'expliquer. L'autre jour, par exemple, il nous a expliqué tout le mécanisme des vaccins, c'est, c'était super ! Et pis d'une manière simple, et tout le monde comprend. Et, dès qu'on dit : « mais on dit ci, on dit ça ». Il dit : « bon, faut en prendre, à l'essai ». Patati, patata, ouais. Et dans la ligue pulmonaire, faut quand même pas oublier que c'est tous des gens qui sont appelés à disparaître, y en a déjà trois, qui ont disparu, [dans le groupe.

**C : Dans votre groupe.]**

**P :** Donc, quand on, tout d'un coup on voit les petites bougies sur la table, c'est pas bon, ça. Ça, ça vous ting, ça fait tilt, hein, ouais, ouais. Ça vous rappelle à l'ordre.

**C : Ce groupe.**

**P :** Oui, mais bon, ça m'int' euh, autrement alors si vous restez cloué à la baraque, vous savez pas ce qu'il se passe. Non, faut essayer de lutter, quoi, dans la mesure du possible, parce que, y a pas de miracles, on le sait. Pis comme le disait le *Docteur D.*, : « vous êtes, vous êtes tous au bouquet final ». *(rires)*. Et c'est vrai, et c'est vrai. Ça, c'est une façon comme une autre de nous le rappeler. Que tout n'est pas super dans les… Et, dans ces réunions, moi je me trouve encore bien. Parce que y a des gens qui ont, beaucoup qui ont des cancers en plus, ils rajoutent *(soupir)*. Mon dieu, mon dieu, ça remet l'église au milieu du village.

**C : De voir les autres.**

**P :** Ouhlà, ouais. Et tout d'un coup, dans la rue, on entend : « ouais, mais j'ai une crève ». ; « je suis malade, j'ai la grippe ». ; « on se meurt ». Oui, oui, oui, allez, allez, allez à l'hôpital. Ouais. Mais bon. Alors chaque fois que, d'ailleurs chaque fois que je passe à l'hosto, je fais un peu tous les étages pour aller dire bonjour, parce que je suis passé à tous les étages. Et je vais leur dire bonjour, parce que j'ai toujours eu d'excellentes relations, très sympas, dans les moments difficiles, ou faciles. Alors je vais leur dire bonjour. Et si, *Claire (infirmière de recherche)* je sais où elle est, un jour il faudrait que j'aille, retourner la voir, voir si elle est là, quoi, au moins juste lui dire un petit bonjour.

**C : Vous avez maintenu des bonnes relations avec.**

**P :** Ouais ! Ben, et c'est, c'est important ça, je trouve ! La preuve c'est que, je peux toujours téléphoner pour, avoir un, un renseignement, savoir à qui je vais me.

**C : Qui appeler.**

**P :** Ouais. Et tout d'un coup, là, *Docteur F.*, je lui ai téléphoné, je lui ai dit : « je suis désolé ». C'était prévu qu'on se voit, je sais pas, le 23 août, ouais le 23 du 8, elle devait venir. J'ai dit : « ben je crois bien qu'on va y annuler, j'ai plus mal ». Elle m'a dit : « c'est pas possible ». Elle, et comment, comment, on en sait rien…

**C : De, de quoi auriez-vous besoin aujourd'hui, ou eu besoin [en terme de, de prise en charge ?**

**P :** Rien,] rien de particulier. Non, je crois qu'on a tout dit, tout, je sais où, je sais où je navigue. Si j'ai besoin de quelque chose, je sais qui, chez qui taper. Aussi alors automatiquement j'ai, une tour de contrôle qui est *Docteur D.*, le *Docteur D*. Je vais chez le cardio, c'est copie à *Docteur D*. Je vais, le *Docteur F.*, copie à *Docteur D*. C'est tout, il est informé de tout. Et une partie, c'est *Docteur E.*, pour l'évolution etc. Parce qu'elle, elle m'avait suggéré d'enlever la, la, la valve si on pensait vraiment que c'était elle qui emmerdait. Pis finalement, elle m'a dit : « non… on, on laisse. On laisse. On laisse et, on verra si, s'il faut remettre les doigts dedans ». *(rires)*.

**C : *(rires)*. Donc, donc si je résume par rapport à ces visites de, de l'infirmière. C'est quelque chose qui vous a été [plutôt utile.**

**P :** Ouais, c'est po', c'est, c'est positif,] c'est positif. C'est peut-être, une année, c'est peut-être un peu long, peut-être. Six moi je pense, ça aurait été aussi. Mais bon.

**C : Une fois par mois pendant six mois.**

**P :** Ouais, voilà. Ouais, au, au début c'était même deux fois par mois. Après, c'était tous les deux mois, une combine comme ça, quoi.

**C : Donc là c'était un petit long une année ?**

**P :** Ouais c'est, bon ça me faisait toujours plaisir de la voir, mais *(rires)*. Mais mis à part ça, ouais, y avait toujours des évolutions, y a toujours des évolutions. Elle suivait aussi : « ah ben vous avez été faire ci. Vous avez fait ça. On parlait de ci, vous l'avez fait ». Bon, elle me connaît pas, au départ. Si je le dis, pis je le fais pas, alors que, si je dis : « ouais je, je vais aller à l'hosto justement». Je le fais. Mais, bon sur six mois c'était, amplement suffisant, [en réalité.

**C : Ça aurait été suffisant,] ouais.**

**P :** Ouais, ouais.

**C : Donc c'est des, c'est des moments où vous avez aussi reçu des informations, même par écrit sur la, voilà la BPCO, et.**

**P :** Alors ça je le savais depuis 2011 déjà, la première fois que j'ai fait une, une pneumo... Là on m'a dit : « ouais, faudra passer à l'oxygène», j'ai dit : « euh oui pardon ? ». Eh oui. Mais je me rendais pas bien compte, en réalité. Pis gentiment, ben on se rend compte *(rires)*.

**C : De ce que c'est.**

**P :** Ouais. Ouais on se rend compte… Et puis, y a des gens aussi, qui se sont également fait opérés. Ça a bien tourné chez eux, ça a moins bien tourné chez un autre, etc. Pis on regarde un peu le, les évolutions. C'est assez curieux. Et je revois, le *Docteur G.*, il me dit : « ah votre copain là, que j'ai re opéré, il est fou ! » Et maintenant, un gars qui marche plus sans oxygène. C'est incroyable. Deux fois le, ouais.

**C : Des évolutions différentes.**

**P :** Ouais, pis ça dépend tout de chaque personne. Ouais. Bon pis, faut dire j'ai, j'ai le poumon, surtout celui-ci, il est dans un état tellement déplorable. Mais vraiment, critique, critique, donc ils peuvent pas me faire de miracles hein. Il dit : « vous avez les machins détruits, c'est détruit, quoi. Avec du, on peut pas faire du neuf avec du vieux ». J'ai dit : « ben ouais. C'est comme ça. Vous avez déjà pas mal fait ». *(rires)*. Vous vous rendez compte, xxx *Docteur E.*, y avait des fuites, elle dit : « je vais vous les trouver les fuites ». Elle est descendue dans les bronches, elle a été mettre des, des petits ballonets à chaque bronche. On gonfle, on regarde ce qui gonfle et pis ce qui est plat. Ce qui est plat, c'est qu'y a la fuite. Et on fait ça à toutes les bronches. *(soupir)* Pis ça a rien servi. Elle y est pas arrivée, la pauvre. Ouais. Elle a dit : « je comprends rien »…

**C : Ouais. Sacré travail.**

**P :** Une fois je me souviens, *Docteur G.* aussi, il m'a dit : « on comprend toujours pas ». J'étais avec des sondes là, deux sondes, et il m'a mis un petit sachet en plastique, qui était tout plat. Et il bougeait pas, ce sachet. Et y a des jours, tout d'un coup je, désaturais, mais j'étouffe ! Je claquais là. Et il fallait prendre ce sachet plat, fallait le, faire comme ça *(il se frotte les mains ensemble)*, je pouvais respirer sur le champ. Mais en, une fraction de seconde, je pouvais, comme maintenant. Ça sortait d'où ? J'ai dit : « expliquez moi ça ». Mais il a jamais répondu. Il m'a dit : « on y comprend rien, moi j'y comprends rien ».

**C : Donc des choses encore qu'on, [qu'on ne comprend pas.**

**P :** Il m'a dit : « je ne comprends plus ! »]… Et c'est terrible, parce que c'est ouh, oui xxx faut aller chercher, c'est leur boulot.

**C : Des choses qu'on, qu'on comprend pas encore.**

**P :** Non.

**C : Par rapport à ça. Et, donc aussi, pour reprendre par rapport à ces visites aussi, vous m'avez dit que ça avait, voilà, pu vous aider à gérer aussi les angoisses, c'est quelque chose aussi.**

**P :** Surtout ouais.

**C : [C'était surtout ça, en fait, qui vous a ?**

**P :** C'est surtout ça.] Sur la maladie en elle-même, on peut rien faire. Le peu que je peux faire, c'est via la car', pas le cardiologue là, le pneumologue. Mais, non, non.

**C : Ouais. Donc ça, vous avez [pu trouver des, des astuces.**

**P :** Apprendre à respirer.] Elle me soutenait pour faire les exercices, parce que c'est sans arrêt. Mais c'est surtout les angoisses.

**C : Oui. Ça c'est quelque chose qui, qui vous a été utile.**

**P :** Oui, oui.

**C : Vous l'avez même rappelé pour, continuer ça.**

**P :** Ouais, [ouais.

**C : C'était,] c'était surtout ça [qui était, qui était important.**

**P :** Qui était important pour moi,] en tout cas pour moi.

**C : Pour vous.**

**P :** Ouais, ouais. Je connaissais aussi d'autres personnes qui ont fait aussi le test, et on se mettait, on se contacte, et je les vois toujours d'ailleurs, à la ligue pulmonaire.

**C : Des gens qui ont participé à l'étude ?**

**P :** Aussi participé, donc.

**C : Ça a aussi permis de faire des contacts.**

**P :** Ouais, pis, voir eux ce qu'ils ressentaient etc. Ouais, tout, tout est, y a une ramification maintenant. Ouais.

**C : C'est ça, vous avez ces, ces contacts. Et aussi dans le groupe de la, la ligue pulmonaire.**

**P :** Ouais. Par contre, je sais pas l'organisation mais, c'est un détail. Mais, quand j'ai fait les trois mois à l'hôpital, je sortais de ces soins, de ces soins intermédiaires, pour aller en salle. J'arrive en salle, la première chose, j'ai une infirmière, une fille qui vient vers moi, pis elle me dit : « je vous propose de faire un, une étude, un test ». J'ai dit : « quoi ?! Je viens de le finir ». *(rires)*. ; « ah pardon ! » *(bruit avec la bouche)* elle s'est tirée. Donc là ça avait pas *(raclement de gorge)*, ça avait pas bien passé le, mais bon.

**C : *(rires)*. L'information avait pas bien passé.**

**P :** Je sais pas ce qu'elle a foutu la *Claire (infirmière de recherche)* là *(rires)*.

**C : *(rires)*.**

**P :** La X *(origine)*, elle est X *(origine)*.

**C : Et, est-ce qu'y aurait autre chose que vous aimeriez rajouter par rapport à.**

**P :** Non.

**C : A ces visites, à ce qui vous a plu, déplu ?**

**P :** Non, non. Moi ce qui est important c'est d'avoir les contacts. J'ai le contact, je peux téléphoner si y a quoi que ce soit, on, on me renseignera, ou elles reviennent. Elles reviennent, pis voir.

**C : Ouais. Donc ça, c'est important pour vous d'avoir ces contacts, de savoir qui vous pouvez appeler ?**

**P :** Ouais. Tout à fait, tout à fait ! Et pis, elles vont pas dire n'importe quoi hein. Voilà.

**C : Très bien. Je crois que si, s'il y a pas autre chose que vous aimeriez, voilà [rajouter. Merci beauc'.**

**P :** Non, non, non.] Mais je pense c'était, finalement c'était pas mal. Je crois c'est la première fois que je fais un genre de, d'exercice de ce genre. Mais pourquoi pas si. Moi je vous dis, si ça peut rendre service à quelqu'un d'autre, je suis preneur. Si ça peut m'aider, pour évoluer, ou du moins maintenir, on va dire maintenir, je suis preneur. Tout ce qui est pour, bon pour la santé, je prends, j'essaie.

**C : Ouais. Pour vous, ou pour les autres.**

**P :** Ou pour les autres, voilà.

**C : Ouais. Qu'est-ce que vous attendiez en participant à, à cette étude ?**

**P :** Rien de spécial, rien de spécial. Bon, toute façon y a quelque chose. Moi, ouais, c'était quand même l'angoisse. L'histoire, fallait quand même régler, un, l'angoisse, et deux, les douleurs. C'était ça, hein. Mais d'abord l'angoisse, parce que, évidemment, si je réfléchis pas, qu'est-ce qu'il se passe, je me lève d'un coup, *(soupir)*, je, je, vous me ramassez à la petite cuillère. Et ça, une fois, deux fois, dix fois, vous vous fatiguez hein, vous vous fatiguez ! Et maintenant, tranquille, on prend le temps, tout est, tout est fait avec le, le temps, quoi. Je suis obligé.

**C : Donc là vous, vous attendiez quelque chose pour régler [ces angoisses et les douleurs.**

**P :** Moi ce qui était,] xxx trouver une combine pour penser à autre chose que. Vous avez mal à l'estomac, vous avez mal à l'estomac, penser à autre chose de, d'agréable, surtout d'agréable, pour essayer d'évacuer ce, ce mal. Et c'est vrai, c'est efficace ! Mais moi, c'était ça.

**C : Penser.**

**P :** Ouais. Et, voyez comme les choses sont bizarres. J'en ai parlé au pneumologue, à *Docteur D*. Et il m'a dit : « mais vous vous souvenez ». Je m'en souviens. Une fois lors de la réunion, ils avaient fait venir une fille de, alors je sais pas si c'est un peu dans votre métier, j'en sais rien quoi, mais de, du X *(région)* probablement, où c'était des massages sous les pieds etc. Vous savez, vous touchez là, ça touche le, le poumon, vous touchez là, ça touche je sais pas quoi, la vésicule, tout ça. Et finalement, y a des terminaisons nerveuses aussi ici *(il se pince entre le pouce et l'index)*, qui font que. Il m'a dit : « on pourrait aussi travailler là-dessus ». Mais je l'ai jamais fait puisque je me suis arrêté à ça, mais, au fond c'était ça, hein. Et tout ça, ça se, voyez comme ça se, ça s'imbrique. Ouais.

**C : Tout est lié.**

**P :** Et pis je vois, y a des gens, à la ligue pulmonaire, qui viennent une fois, deux fois, pis je les ai plus revu pendant cinq ans. C'est-à-dire ils ont baissé les bras, ils font aucun effort, jamais vu dans les exercices. Bon, question de, mental. Ils veulent qu'on fasse tout pour eux. Parce que ça aussi on a tendance à, à baisser les bras et, prendre la facilité ! Oui, parce que se baisser c'est. Je me suis rendu compte ce matin, je commence à avoir des difficultés pour mettre des chaussettes, tout simplement. Ça paraît idiot ! Mais ça me coupe le souffle. Parce que, le problème c'est le diaphragme. Vous êtes plié en deux, diaphragme, ça vous coupe tout, et là j'étouffe ! Donc, il faut que je trouve une combine. Et ça on va la, on va la trouver. Et si je la trouve pas, y a des gens à qui je peux demander ! Voyez ?

**C : Vous savez.**

**P :** Et voilà.

**C : A qui faire appel.**

**P :** Parce que eux, ils ont déjà vu le problème. Alors que moi, non *(raclement de gorge)*. Et on trouve toujours une solution. Mais je vois que, ouais, je peux pas faire du vélo, par exemple, à la physio, ça me coupe trop le, le diaphragme. Et le diaphragme, pour respirer, je vous explique pas. *(raclement de gorge)* J'ai les chats. C'est, ouais.

**C : Ouais. Vous savez à qui faire appel pour trouver des solutions.**

**P :** Tout à fait. Mais, mis à part ça, vu que c'est enregistré, *(il s'adresse à l'enregistreur)* ça me ferait un grand plaisir de, vous rencontrer, *Professeur A. (pneumologie)*. Parce qu'on m'a parlé, passablement de vous. Voilà.

**C :** ***(rires)*** **Ouais. Alors je transmettrai.**

**P :** Ouais.

**C : Mais, je sais pas si vous avez encore quelque chose que vous aimeriez [rajouter ?**

**P :** Non, rien de particulier,] rien de particulier.

**C : [Merci.**

**P :** Et le] *Professeur A. (pneumologie)*, il travaille pas aussi à X *(hôpital)*, pour le machin du sommeil ?

**C : Alors, c'est possible. Je sais pas exactement.**

**P :** Parce que je connais des gens qui ont, qui ont été faire les tests, enfin, pour.

**C : C'est possible. Je saurais pas vous dire [s'il est rattaché à d'autres.**

**P :** Ouais, mais je crois qu'il,] ouais, il étudie ça. Les apnées.

**C : Oui. Du sommeil.**

**P :** Ouais.

**C : Ouais, c'est possible. Je lui transmettrai en tout cas.**

**P :** Ouais, ouais, ouais.

**C : Merci, merci beaucoup d'avoir pris [le temps de répondre.**

**P :** Mais je suis à peu, à peu près sûr] que je, je l'ai vu 50 fois à l'hôpital, et au X *(étage de l'hôpital)* probablement, sans le connaître, hein.

**C : Ouais, c'est possible.**

**P :** J'ai fait aussi des stages, pas maintenant, mais j'avais fait aussi des stages à X *(lieu)*, j'avais été à X *(lieu)*, y a un centre de réhabilitation. Bon j'ai tout essayé, quoi… Ça marche, ça marche pas, quoi. Bon, y a pas de miracles non plus.

**C : Vous avez essayé beaucoup de choses.**

**P :** Ben ouais ! Et la seule chose, c'est de, ouais, faire des exercices. C'est ça le, c'est fondamental ! Que ce soit pour le coeur, comme le coeur et le poumon c'est…

**C : Merci, merci beaucoup !**

**P :** Ben voilà, je vous en prie.

**C : D'avoir pris le temps de [me dire tout ça *(rires)*.**

**P :** C'est gentil, mais je vous dis, comme] j'ai été vendredi, c'est pour ça que je vous suggérais éventuellement qu'on se voit à l'hôpital.

**C : Ouais, c'est gentil. [Oui, oui.**

**P :** Juste après le.] C'est, c'est avec *Claire (infirmière de recherche)* c'est ce qu'on faisait d'ailleurs. J'ai dit : « ben je passe vers vous comme ça, vous avez pas besoin de vous déplacer ». Et voilà.

**C : On va pouvoir éteindre la petite machine.**

# Participant n°8

| |
|---|
| Nom d'emprunt : Yves |
| Âge : 67 ans |
| Sexe : M |
| Groupe : Intervention |

**C : Si je reprends par rapport à cette étude. Vous avez participé à une étude qui avait pour objectif de comparer le traitement habituel de votre maladie pulmonaire, la BPCO, avec une prise en charge précoce, soutenue et intégrée. Vous étiez dans le groupe bénéficiant de cette prise en charge, globale, spécialisée. Et vous avez reçu la visite d'une infirmière une fois par mois, pendant un an. Pouvez-vous me raconter comment ça s'est passé ?**

**Y :** Avec les infirmières ?

**C : Mmh.**

**Y :** Gentilles ! *Madame Chappuis (infirmière de recherche)* je l'aimais bien ! J'écoutais ce qu'on disait. Elles étaient, pour moi, elles étaient sympathiques, quoi ! Ça me réconfortait un petit peu ! Pas beaucoup, mais ça va, quoi. Pis après j'étais de nouveau hospitalisé pour ça. Elles étaient venues me voir à l'hôpital. Peu avant, j'étais à X *(hôpital)* aussi. Mais pour moi, les poumons ça va, si j'avais pas mal au ventre, hein !

**C : Ouais donc ça, ça vous réconfortait ces visites des infirmières, [une fois par mois ?**

**Y :** Ouais.] Bon, là j'en ai une qui vient une fois par semaine, hein, X *(service)* là. Je les vois tous les jours, ils sont juste en face. Mais, c'est une maladie, c'est de la merde, hein. A X *(clinique)*, ils m'ont dit que c'était même pas tellement les poumons hein, c'était plutôt le ventre…

**C : Donc voilà, là c'est plutôt le ventre, oui.**

**Y :** Bon, les poumons ils sont abîmés hein, ça c'est sûr, hein. J'ai fumé pendant 50 ans, hein. Puis j'ai arrêté quand j'ai eu la grippe, j'ai été aux soins intensifs pour la grippe.

**C : Là vous avez arrêté.**

**Y :** Ouais, j'ai arrêté. Du jour au lendemain… Voilà.

**C : Et, et peut-être, qu'est-ce que vous attendiez en participant à cette étude ?**

**Y :** Ben j'ai cru qu'ils allaient trouver un médicament ! *(rires)* Mais y en n'a pas, hein. Bon, y a X *(nom de médicament)*, ça fait rien du tout. Et puis l'autre, ça me fait, la X *(nom de médicament)*, ça me fait rien non plus... Alors je souffle dans le machin-là, mais, j'arrive à monter à six, sept.

**C : Plutôt des attentes par rapport aux médicaments ?**

**Y :** Ben les poumons ils sont abîmés depuis qu'ils m'ont opéré en 2011, hein ! J'étais en manque d'oxygène pendant l'opération. Alors ça m'a, il paraît que ça m'a abîmé un peu les poumons. Parce qu'avant ça allait extra, hein ! Ça allait bien jusqu'en, jusqu'à qu'ils m'opèrent en 2013 de ce trou-là, j'arrivais à marcher et tout ! J'étais pas essoufflé. Puis là, depuis qu'ils m'ont opéré là de, de, ils ont recousu l'intestin. Je crois que c'était l'opération de trop. Mais ils voulaient pas laisser le trou ouvert, parce que ça risquait de, de s'infecter hein, pour finir. [Alors voilà.

**C : Donc là ça,] ça allait moins bien depuis l'opération, oui. Et, qu'est-ce qui vous a plu ou déplu dans les, les visites de l'infirmière ?**

**Y :** Y a rien qui m'a déplu. Non, ça je peux dire qu'y a rien qui m'a déplu. Je vous dis, elles étaient tout le temps sympas. Bon, c'était toujours les mêmes questionnaires, hein *(rires)*.

**C : Et y avait, y avait des questionnaires à remplir.**

**Y :** Ouais, ouais, mais c'était tout le temps les mêmes. Non, pour moi c'était, ça allait, ça allait bien, hein ! Elles étaient pas agressives, rien du tout, hein ! J'ai même vu ici les médecins des soins palliatifs, super gentils, hein ! Moi j'ai rien à dire à l'hôpital, j'ai toujours été bien soigné… Alors voilà.

**C : Donc des, des infirmières et des docteurs gentils.**

**Y :** Oui.

**C : Et, qu'est-ce que vous auriez peut-être, souhaité recevoir en plus ou en moins pendant ces visites de l'infirmière ?**

**Y :** Ben pas grand-chose, hein ! Pour moi, ça me suffisait ce qu'elles me disaient, hein ! Moi, pour moi, je vous dis ça allait très bien, hein !

**C : Qu'est-ce qu'elles vous disaient justement ?**

**Y :** Qu'il faut être courageux ! Pis que ça allait bien, hein ! Elles me faisaient leurs examens-là, elles disaient que ça allait pas mal ! Elles m'ont jamais vu souffrir des poumons hein, quand elles sont venues. Alors. J'avais une préférence pour *Madame Chappuis (infirmière de recherche)*, puis après elle est partie aux, elle est au X *(service)* maintenant… Elle est à, là-haut là, à X *(hôpital),* non ?

**C : Oui, c'est possible. Mais vous avez appréciez donc la visite *de Madame Chappuis (infirmière de recherche)* ?**

**Y :** Les deux autres aussi, hein !... Les trois elles étaient très gentilles !

**C : Donc rien de, [rien de xxx.**

**Y :** Pis vous vous] êtes la quatrième ! *(rires)*

**C : *(rires)*. Exactement. Sauf que je suis pas infirmière, hein *(rires)*, mais.**

**Y :** Vous êtes psychiatre, non ?

**C : Psychologue, psychologue, oui, voilà.**

**Y :** Oui, c'est pareil, quoi.

**C : Je suis pas, je suis pas médecin, voilà, c'est ça la, la différence. Et, peut-être quelle, quelle est votre appréciation des informations que vous avez reçues sur la BPCO, sur le traitement, lors des visites de, de l'infirmière ?**

**Y :** Du, du positif, hein ! Du positif, moi je peux pas vous dire plus hein, parce que, pour moi ça, elles étaient bien !

**C : C'était positif.**

**Y :** Oui !

**C : Voilà, pas de.**

**Y :** Non, non, y a pas de soucis là-dessus, hein !

**C : Voilà, quelque chose de plutôt positif, donc, ces visites.**

**Y :** Oui.

**C : Et, comment les, les visites de l'infirmière et les informations que vous avez reçues sur, sur la maladie vous ont-elles aidé ou non à gérer ces symptômes de, de la BPCO comme peut-être des difficultés respiratoires, [des douleurs ?**

**Y :** Oui, elles m'ont aidé, quoi.] Elles m'ont aidé à, à me, quand, quand j'étais, j'avais pas le moral, elles m'ont remonté un peu le moral, mais. Oui, ça va, elles m'ont bien aidé, quoi !... Mais je peux pas vous dire plus, parce que moi j'étais très content, de leurs visites, et pis les discussions qu'on a eues ! Et pis voilà, hein ! Elles, elles peuvent pas faire grand-chose de mieux hein ! A part remplir leurs questionnaires, discuter avec les gens, pis voilà hein...

**C : Donc elles vous ont remonté le moral, [des fois.**

**Y :** Voilà.] Oui !...

**C : Amené du réconfort.**

**Y :** Ouais… Parce que le moral il est en, en bas, hein. C'est mon ventre qui m'embête, et pis que ça me fait souci. Puis j'arrive pas à souffler. Le moindre effort que je fais, ça me tire dans le ventre, puis ça me coupe le souffle hein, j'arrive pas à reprendre le souffle, hein ! Mais ça dure pas longtemps, hein, ça dure, cinq minutes, c'est tout.

**C : [Le ventre qui vous empêche de souffler.**

**Y :** Ouais, ouais, c'est.] L'impression qu'il est tout collé, mon ventre.

**C : Et c'est, c'est ça qui vous fait du souci ?**

**Y :** Ouais... J'ai une grosse boule qui a poussé. Le médecin il a dit faut la surveiller.

**C : Dans le, sur le poumon ?**

**Y :** Sur les seins-là, sur le. J'en avais déjà eu une, ils ont enlevé ! Alors voilà.

**C : Oui. Et, si on regarde peut-être au niveau des, des proches, quel soutien avez-vous reçu de la part de vos proches ?**

**Y :** Ah non ils m'aident hein ! Ma soeur, elle me fait mes courses, quand ça va pas. Non, non, pour ça alors, ça va. Non, j'ai une soeur qui est très gentille. Bon, mon frère il s'en fout. L'autre, il habite en X *(pays)*. J'ai encore ma maman, hein. Bon, elle, maintenant elle est dans une maison, depuis pas longtemps là. Elle a 93 ans. Elle va mieux que moi ! *(rires)*

**C : Votre maman.**

**Y :** Oui.

**C : Donc voilà, vous avez votre soeur qui vous fait les courses.**

**Y :** Oui, oui, oui.

**C : Qui est là.**

**Y :** Oui, oui, elle est là. Elle habite tout près, alors.

**C : Ouais. Elle était pas présente pendant les visites de, de l'infirmière, [voilà ?**

**Y :** Non, non, non, non.] Non, ça je gère tout seul.

**C : Oui. Et au niveau des, du soutien des proches, de quoi d'autre auriez-vous eu besoin, auriez besoin aujourd'hui ?**

**Y :** De rien, j'ai besoin de rien, aujourd'hui. Le laveur de vitres, mais il va venir *(rires)*.

**C : Le laveur de vitres *(rires)*.**

**Y :** *(rires)*. Autrement je me débrouille, hein, je fais mon ménage, ma lessive, je fais tout tout seul, à manger… Je me débrouille, hein !

**C : Vous vous débrouillez.**

**Y :** Des jours c'est dur, des jours ça va mieux, des jours c'est. C'est pas évident, quoi, tous les jours ! Comme dirait *Docteur D.*, le *Docteur D.*, c'est, je sais pas si vous connaissez, le chirurgien.

**C : Non, je connais pas.**

**Y :** C'est un grand spécialiste de l'estomac, il m'a opéré trois fois. Il m'appelle le « rescapé », parce que j'ai été plusieurs fois aux soins intensifs. Il se demande comment encore je tiens debout. C'est le moral, hein !

**C : Qui vous, qui vous tient ?**

**Y :** Oui, oui ! C'est beaucoup la tête hein, la maladie. Disons que je me laisse pas aller ! Quand j'ai mal comme ça là, que j'arrive pas à reprendre mon souffle, ben je, j'essaie de le reprendre tout seul, hein. Et puis c'est, puis ça revient, quoi. Je me laisse pas… tomber dans le, dans le canapé puis, me laisser aller, hein, non, non !

**C : Vous vous laissez pas aller, [et vous.**

**Y :** Ouais.] J'ai dit tant que ma mère est vivante, il faut que je tienne le coup. Ce qui est logique, hein ! Alors voilà.

**C : Le moral qui vous fait tenir.**

**Y :** Eh oui ! Bon, c'est pas évident, mais ça va !...

**C : Et, et quelle a été pour vous, justement, l'utilité de ces visites de, de l'infirmière, une fois par mois ?**

**Y :** Je sais pas moi, c'est toujours du bien ! L'utilité, bon ça m'a pas guéri, mais, c'est comme je vous dis, ça m'a remonté un peu le moral, et puis j'étais content de les voir, quoi !... Voilà.

**C : C'était surtout ça le, [remonter le moral, et.**

**Y :** Oui,] oui !

**C : Avoir du plaisir à les voir [xxx.**

**Y :** Voilà !]…

**C : Et, je sais que parfois, y a du, du soutien au niveau spirituel qui peut être apporté. C'est quelque chose que vous avez reçu, ou ?**

**Y :** Non, je veux pas. On m'a déjà proposé d'aller voir un psychiatre, un machin. Je veux rien du tout. Je me débrouille tout seul. Vous voyez un psychiatre, vous êtes encore plus fou qu'eux *(rires)*. Je dirais pas les psychologues, parce que je les connais pas !

**C : *(rires)*.**

**Y :** Je réfléchirai après, quand vous serez partie *(rires)*.

**C : D'accord *(rires)*. Vous verrez ce que vous penserez. Donc voilà, vous avez, on vous a proposé de voir un psychiatre, mais vous aviez pas, [envie.**

**Y :** Non,] non. Non, parce que c'est au sujet de mon ventre, hein. Parce qu'il paraît qu'ils ont des médicaments, pour calmer, pour, pour les angoisses. C'est même pas les angoisses, c'est, c'est une,

une peine que j'arrive pas ! Je marche un moment, tout d'un coup c'est, mon ventre qui, qui, comme s'il se bloquait, pis ça, ça me coupe la respiration, alors faut que je m'arrête ! Ben, voilà.

**C : C'est, c'est pas des angoisses, mais c'est le, [le ventre.**

**Y :** Oui, oui,] oui, oui, c'est vraiment le ventre qui... Alors voilà.

**C : Oui. Et… Je sais que parfois aussi on peut, c'est peut-être quelque chose que, qui a été fait avec les, les infirmières lorsqu'elles sont venues, c'est de, discuter des décisions autour des soins en fin de vie, où on peut décider si, dans l'éventualité où, [voilà.**

**Y :** Ça c'est déjà] fait le papier. C'est déjà fait le papier. Je veux pas de, d'enterrements, j'ai tout donné mon corps à la science... Il est à l'hôpital, le papier.

**C : Vous avez, [fait un papier déjà.**

**Y :** Oui.] Oui, oui !

**C : C'est, c'est quelque chose que vous aviez déjà fait avant les visites des [infirmières ?**

**Y :** Oui, oui, oui,] j'avais fait déjà ! Une fois que j'étais à X *(clinique)*, j'avais fait. Quand j'avais eu des, comment on appelle ça, ah des céphalées en grappe. Bon, ça c'est douloureux, hein !

**C : Des céphalées.**

**Y :** Faut de l'oxygène pour, pour que ça passe. Pendant dix minutes, faut mettre de l'oxygène, à dix. Et ça passe, hein !

**C : Après, les céphalées passent.**

**Y :** Ouais mais ça fait deux, trois crises dans la nuit. Ça venait pas la journée, c'est la nuit. Mais ça vous paralyse tout le côté, hein ! Puis c'est pas revenu !

**C : C'est, c'est à ce moment-là que vous avez aussi écrit [ça.**

**Y :** Ouais,] ouais c'est la, c'est la Doctoresse à X *(clinique)*, qui m'avait dit. C'est les directives anticipées. C'est déjà fait ça !

**C : Ça vous aviez, voilà, déjà. Donc vous aviez pas rediscuté de ça avec les infirmières ?**

**Y :** Oui, on a discuté de ça, oui ! Parce qu'ils trouvaient pas le papier, alors ils l'ont cherché. Même avec les Docteurs, j'ai discuté de ça. Non, non, ça, ça me fait pas peur hein, non, non. On doit tous y passer, hein. Moi je suis malade depuis l'âge de 36 ans, que j'ai des problèmes d'estomac. Alors voilà.

**C : Vous avez pas peur.**

**Y :** Non, non… Ce que je veux, c'est pas souffrir, c'est tout ! Mais tous les tuyaux, j'ai refusé. Si jamais ils me font mettre des tuyaux, je veux pas.

**C : Tout ce que vous souhaitez c'est pas avoir mal, [souffir.**

**Y :** Voilà !]…

**C : Et, est-ce que, vous avez d'autres choses, par rapport à ces visites, des infirmières une fois par mois, que vous aimeriez [me dire ?**

**Y :** Non, non], je vous dis elles étaient sympas ! Elles m'ont tout expliqué comme il faut !... Alors…

**C : Pas de, de besoins particuliers ?**

**Y :** Non, moi j'ai besoin de rien !

**C : C'est quelque chose que.**

**Y :** J'ai mon appareil pour la nuit là. Bon, y a pas d'oxygène hein, c'est juste, pour éclaircir les, les bronches. Je le tiens longtemps aussi, hein. En plus l'infirmière qui vient pour le contrôler, elle m'a dit que j'étais un exemple, hein ! C'est cinq, six heures toutes les nuits, hein, que je le laisse !

**C : L'appareil.**

**Y :** Ouais ! Ça me gêne pas, hein !...

**C : Donc, voilà, c'est quelque chose que, que vous avez apprécié, qui vous a apporté du, du réconfort.**

**Y :** Ouais, ouais, ça m'a… ça m'aide sans m'aider, quoi, ça, je le mets quand même. Mais quand j'ai mal au ventre, j'y sens hein, dans l'appareil. Maintenant mon ventre il me réveille la nuit ! Et le matin, c'est comme si c'était vraiment tout collé. J'arrive plus bouger le dos maintenant, le matin. Je suis content d'avoir rendez-vous le 11.

**C : Pour votre ventre, oui… Donc, donc voilà si, si je résume un peu par rapport à ces, ces visites des, des infirmières. C'est quelque chose que, qui vous a apporté du réconfort.**

**Y :** Ben oui, oui, oui !

**C : Vous appréciiez leurs visites.**

**Y :** Oui !

**C : Vous les trouviez gentilles.**

**Y :** Voilà.

**C : Et, et en termes, aussi de, ce que vous attendiez avant l'étude, c'était plus quelque chose par rapport aux médicaments, [voilà.**

**Y :** Oui.]

**C : Vous auriez souhaité qu'on trouve un médicament.**

**Y :** Voilà, pour, pour essayer d'améliorer cette maladie ! Parce qu'y en a beaucoup de gens qui ont cette maladie. Puis pour l'instant, y a pas de, de médicaments qui soignent vraiment, hein ! Alors.

**C : C'est ce que vous pensiez qu'on aurait peut-être pu trouver en participant [à cette étude ?**

**Y :** Ouais.] Bon, moi ça va, j'ai pas besoin d'oxygène hein. Y a un monsieur là, que je vois tous les jours là, qui se promène avec son oxygène, ça, ça doit pas être marrant aussi, hein, de vivre avec l'oxygène ! Je connais aussi un monsieur là, qui habite à côté là, un copain depuis longtemps. Alors lui, il peut pas sortir, lui. Il est trop abîmé ses poumons.

**C : Donc vous avez pas besoin de cette oxygène ?**

**Y :** Non, non. C'est même <u>mauvais</u> pour moi, on m'a dit.

**C : D'avoir l'oxygène ?**

**Y :** Oui.

**C : D'accord… Donc voilà c'est différent entre, entre personnes.**

**Y :** Ouais…

**C : Est-ce qu'y avait, autre chose que vous aimeriez encore rajouter, par rapport à.**

**Y :** [Ces visites.

**C : A ces visites] des infirmières une fois par mois ? Ce que, ce que vous a apporté, ce que ? Est-ce qu'y aurait d'autres choses que vous, vous imaginiez que vous auriez aimé avoir ?**

**Y :** Non.

**C : Ou, quelque chose de différent ?**

**Y :** Non, non. Je vous dis, moi elles m'ont tout bien expliqué, et elles étaient toutes sympas. Elles pouvaient pas m'amener plus, hein de toute façon !

**C : Elles vous ont bien expliqué par rapport à la maladie ?**

**Y :** Oui, oui, oui !... Je peux pas vous dire plus que je vous dis hein, parce que moi, pour moi elles étaient <u>super</u> ! Si y a des gens qui sont pas contents, parce que je pense y a des, des gens pas contents, ils vont en avoir, hein !

**C : Mais, mais pour vous c'était, c'était super.**

**Y :** Oui !

**C : Ouais, ces visites.**

**Y :** Oui.

**C : Très bien. Donc si, vous avez pas autre chose à, à rajouter, je vous.**

**Y :** [Non.

**C : Je vous] remercie de, d'avoir pris le temps pour répondre à ces questions.**

**Y :** Parce que moi je peux, je peux pas vous en dire plus, hein ! Pour moi, elles étaient sympas, et tout ! Elles m'ont expliqué, comme il faut, que ça pouvait pas tellement se soigner. Qu'il fallait faire attention à moi, pis voilà, quoi ! Moi j'ai beaucoup d'antécédents, c'est mon estomac, hein ! C'est pas, pour moi c'est presque une, c'est pas une goutte d'eau dans la mer, mais ! Ce qui me, ce qui m'embête, c'est que ça m'handicape !

**C : L'estomac.**

**Y :** Mais moi, mais moi je suis sûr que ça vient du ventre ! C'est ce qu'ils m'ont dit à la clinique X *(lieu)* là, spécialisée dans les poumons. Ils m'ont même demandé ce que je faisais là ! Alors voyez ! *(soupir)*.

**C : Ce qui vous embête, c'est que ça vous handicape la, la BPCO ?**

**Y :** Oui.

**C : C'est ça ?... C'est plutôt donc le, le ventre, [qui vous fait souci ?**

**Y :** Oui, oui,] parce que la BPCO, ça va, hein !

**C : C'est une goutte d'eau.**

**Y :** Oui !... Ça va tant que j'ai pas besoin d'oxygène. On m'a dit que pour moi c'était très mauvais, <u>l'oxygène</u> !

**C : Donc voilà, actuellement c'est plus le, le ventre qui vous fait du souci.**

**Y :** Oui, oui.

**C : D'accord. Mais, en tout cas ça, c'est quelque chose que vous appréciez d'avoir ces, [ces visites des infirmières.**

**Y :** Oui, oui, les infirmières,] j'ai apprécié ! Mais après y en a une qui est venue, parce qu'elle m'a dit qu'y avait encore une autre étude, qui durait une année...

**C : Alors, je sais pas si c'est.**

**Y :** C'était une prolongation, qu'elle venait deux fois par année. Elle est venue une fois, puis elle doit revenir, je sais pas quand.

**C : D'accord. Ça je, je saurais pas vous dire pour cette partie-là. En tout cas je sais que, là où vous aviez participé pour une fois par mois, pendant un an, ça on va, là c'est terminé avec les questionnaires. Et pis que maintenant, y a cette autre partie, c'est pour ça que je suis là, aussi pour, [qu'on puisse un petit mieux cerner les besoins des personnes comme vous qui ont participé à, à cette étude.**

**Y :** Oui, oui.] Non, non, moi j'étais très content de cette étude, hein ! Si y en a d'autres, je les fais... Non, non.

**C : Pourquoi vous avez aussi accepté de, de participer à cette étude ?**

**Y :** Parce que c'est intéressant, hein ! Ça, ça aide la médecine, ça. Moi je sais que ça m'a pas arrangé beaucoup, mais, si ça aide la médecine. On m'a bien expliqué, c'est tout… Voilà.

**C : Plutôt pour aider la médecine...**

**Y :** Eh oui, si on peut, hein !...

**C : Très bien ! Ben je vous, je vous remercie d'avoir pris le temps de partager tout ça *(rires)*.**

**Y :** Je peux pas vous dire plus que je vous dis, parce que, je vais pas critiquer les infirmières quand même, si elles étaient gentilles, et tout !

**C : *(rires)* Non.**

**Y :** Qu'elles m'ont bien aidé.

**C : Très bien.**

**Y :** Alors voilà.

**C : Oui. Merci d'avoir partagé tout ça. On peut, à part si y avait autre chose encore que vous vouliez rajouter ?**

**Y :** Non, non.

**C : Voilà, je pense qu'on a, vous avez dit ce que vous aviez à me dire *(rires)*.**

**Y :** Faut qu'ils trouvent une solution pour mon ventre.

**C : C'est ça qui vous préoccupe.**

**Y :** Et là je, normalement, je dois descendre voir le, le pneumologue, la semaine prochaine, mais, je vais téléphoner, je vais pas, j'arriverai pas à descendre. Faut que je descende chercher l'appareil là, qu'on met au bout du doigt. Après, faut que je retourne le lendemain, non j'arrive pas. C'est trop

fatiguant pour moi, pour l'instant... Alors je vais téléphoner, qu'il me donne un autre rendez-vous. De toute façon, je vois l'infirmière là, qui va venir, pour contrôler l'appareil, alors, c'est pas la peine que je descende, hein !

**C : Voilà c'est, plutôt le ventre maintenant qui.**

**Y :** Au mois d'août, j'ai été aux urgences, ils m'ont fait des radios des poumons, ils m'ont dit que ça allait bien. Alors, c'est pas la peine que j'aille là-bas.

**C : Plutôt, plutôt le ventre.**

**Y :** Ouais.

**C : Merci en tout cas. On peut arrêter là le, *(rires)* l'enregistreur.**

# Participant n°9

| |
|---|
| Nom d'emprunt : Gérard |
| Âge : 70 ans |
| Sexe : M |
| Groupe : Intervention |

**C : Vous avez participé à une étude qui avait pour objectif de comparer le traitement habituel de votre maladie pulmonaire, la BPCO, avec une prise en charge précoce, soutenue et intégrée. Vous étiez dans le groupe bénéficiant de cette prise en charge globale spécialisée. Et vous avez reçu la visite d'une infirmière une fois par mois pendant un an. Pouvez-vous me raconter comment ça s'est passé ?**

**G :** Ecoutez, sur l'ensemble ça s'est bien passé. J'ai aucun souvenir que chaque fois qu'elle venait chez moi c'était un calvaire, non, c'était agréable, quoi. On avait des, des discussions plutôt sympas, ou bien ces questionnaires à remplir, ça durait quoi, le temps, ça durait quoi, une demi-heure, approximativement. Autrement, bon, j'ai pas de souvenirs désagréables. Du sujet quoi.

**C : C'était quelque chose d'agréable ?**

**G :** Ouais, c'était du fait que j'avais accepté de participer à cette étude. Autant y prendre comme un côté agréable, c'était pas une… C'était pas un truc qui m'astreignait à faire quelque chose que j'ai pas du tout envie. Parce que j'étais libre de toute façon de rompre le contrat quand je voulais, quoi. Donc pour moi c'était intéressant, quoi.

**C : C'était quelque chose d'intéressant, et.**

**G :** Oui.

**C : De participer.**

**G :** Oui, pis de faire évoluer le, ma maladie, aussi, parce que bon, je sais pas dans quel but ça va aller, mais enfin, si ça peut améliorer quelque chose pour les autres, autant le faire.

**C : Oui. Voilà, c'était plus pour.**

**G :** C'est plus pour les autres que pour moi. Parce que bon, maintenant, moi je sais que, j'ai encore de la chance d'être encore là, pis c'est tout.

**C : Ouais, c'est plus, plus pour les autres.**

**G :** Ouais, parce que bon, moi avec ce qu'il me reste, je suis tout content de, chaque jour que je considère que, je diminue pas trop, quoi…

**C : Oui. Et, et qu'est-ce que vous attendiez en participant à cette étude ?**

**G :** Rien. J'attendais rien du tout. Je savais que ça allait pas du tout améliorer mon, ma santé. C'était surtout un truc pour, bon, comme je vous l'ai dit, faire profiter les autres. Mais, un apport pour moi, personnel, rien du tout.

**C : Ouais. Pas de, pas d'apport pour vous ?**

**G :** Non. Qu'est-ce que ça aurait pu m'apporter, quoi ? Plus grande force morale ? Et autre ?

**C : Peut-être.**

**G :** Oui. Y a des moments, mais bon, y a des moments où c'est, on prend conscience de sa maladie pis c'est dur, quoi.

**C : Ouais, ouais. Et, qu'est-ce qui vous a plu ou, ou déplu dans les visites de, de l'infirmière ?**

**G :** Mais y a rien qui m'a déplu. Elle venait là, elle faisait son job, moi je participais à cette, à cette étude, et pis, y avait rien de désagréable, j'entends, qui m'a, y a rien qui m'a déplu. Ça a jamais été un calvaire quand elle venait ici. J'avais une relation assez sympa avec *Madame Chappuis (infirmière de recherche)*, et pis c'est tout, quoi… C'était simple, y avait pas de, on discutait de la pluie, du beau temps, des fois, y avait, même ma fille qui venait là de temps en temps discuter avec nous. Ça allait, quoi !

**C : Votre fille était présente donc, [parfois ?**

**G :** Pas tout le temps,] non, de temps en temps, quand elle était là quoi, elle était… Ben elle participait à la discussion, quoi.

**C : Et, comment ça s'est passé pour elle, ces visites, pour votre fille, de l'infirmière ?**

**G :** Ben rien, elle était là. D'ailleurs elle posait la question si ça dérangeait pas qu'elle soit là ou pas. Non, l'infirmière répondait que ça dérangeait pas, quoi. Mais elle a pas assisté à toutes les, toutes les séances hein.

**C : Elle était là de temps en temps ?**

**G :** Oui, oui ! Oui, quand elle était, elle venait me faire des courses, ou, quand elle était là, quoi, quand elle était vers moi.

**C : Donc vous, vous avez votre fille qui vous fait les courses de, [de temps en temps ?**

**G :** Ouais,] ouais parce que je peux plus me déplacer.

**C : Oui. Et, et, quel soutien vous avez de, de vos proches ?**

**G :** Ben, le soutien de mes proches, ben, du fait que je peux plus me déplacer du tout *(raclement de gorge)*, ma fille qui me fait mes courses. Mon ex-femme qui vient, qui donne un coup de main aussi, qui fait des courses. On se voit, on mange ensemble. On essaie de vivre un peu normalement, quoi, par rapport à ma maladie.

**C : Ouais, donc vous avez votre ex-femme et votre fille qui vient [donner des coups de main.**

**G :** Ouais,] oui, oui. Et pis bon, autrement, j'ai une aide ménagère, quoi, toutes les semaines.

**C : Qui vient aussi.**

**G :** Ouais, ouais.

**C : Ouais, ouais, parce que là vous dites vous pouvez plus vous déplacer, donc elle vient.**

**G :** Non, j'ai beaucoup de peine à me déplacer, je dois dire. Du fait de mon état physique déjà, et pis bon j'ai été opéré d'une hanche, ça a pas été une réussite, non plus. Et pis bon, j'ai un déambulateur, quoi. Et je dois dire que j'ai vraiment de la peine, maintenant.

**C : Donc voilà c'est des coups de main.**

**G :** Pardon ?

**C : C'est des coups de main, [du coup de.**

**G :** Ouais.] Ça aide quand même !

**C : Ouais. Et, peut-être, par rapport aux informations que vous avez reçues, lors des visites des infirmières, sur la BPCO, son traitement ou son évolution. Quelle est votre appréciation de ces informations que vous avez reçues ?**

**G :** *(soupir)*. Pour dire la vérité, je m'en rappelle pas, parce que, les informations ouais, mais bon, le, le

traitement, qu'est-ce qu'y a comme, j'ai rien eu comme traitement de, à part prendre mes médicaments que mon médecin traitant me donne, y a rien eu d'autre comme traitement, hein…

**C : Pas.**

**G :** Pardon ?

**C : Pas de souvenirs [de ?**

**G :** Non.]

**C : Des informations que vous avez [pu recevoir ?**

**G :** Non, des informations si,] y avait des, j'ai l'exemple d'une information que je, que *Madame Chappuis (infirmière de recherche)* m'a donnée. C'est que j'avais des moments donnés où je <u>paniquais</u> à mon rendez-vous. Ça veut dire paniquer, je paniquais pour faire ma toilette, ça me stressait complètement, et c'est là que j'ai commencé à prendre du X *(médicament anxiolytique)*. Avant je prenais du X *(médicament anxiolytique)*, mais ça faisait pas effet assez vite. En prenant un demi X *(médicament anxiolytique)*, je pouvais, je vaquais plus facilement à mon, à mon rendez-vous sans me stresser, pis m'angoisser. Ça me permettait un certain relâchement au niveau pulmonaire, au niveau du souffle. Ça doit être le seul truc, le souvenir que j'ai, qu'elle me donnait. Autrement, au niveau de mes médicaments, ça a rien changé, hein.

**C : Ouais. Juste, juste le X *(médicament anxiolytique)*.**

**G :** Ouais, ce X *(médicament anxiolytique)*, ça me fait, j'en prends maintenant pour aller, exemple, j'en ai pris un tout à l'heure, pour pas me stresser, pour me doucher, me raser, parce qu'autrement, je dois mettre mon oxygène, c'est pratiquement deux litres et demi, pour…

**C : Ouais, ça permet de.**

**G :** Ça me permet de rester dans des normes <u>normales</u>, sans me bourrer de, d'oxygène, et pis de...

**C : De faire les choses [sans se stresser ?**

**G :** Ouais.]

**C : Oui, oui. Et, au niveau de, de la gestion des symptômes physiques de la BPCO, de la difficulté respiratoire, ou des douleurs, comment, les visites de l'infirmière et les informations que vous avez reçues, ont pu vous aider ou non à gérer les symptômes ?**

**G :** Mais, je dois dire que j'ai jamais eu de, de, de grosses, de grosses alertes, j'entends. Ouais, j'ai manqué d'y rester y a deux ans, en, parce que j'ai eu, mais c'était autre chose, j'ai eu une, j'ai eu une tumeur au, à l'intestin grêle. Pis j'ai perdu du sang, je m'en étais pas du tout rendu compte, dans mes selles, et pis, ben quand j'ai fait une prise de sang, il me restait, je crois, un tiers du sang qu'il me fallait quoi, je suis arrivé aux urgences, c'était limite. Je suis resté 15 jours là-bas, pour me retaper, ils m'ont mis, je sais pas, je crois deux litres et demi de sang, pour, pour me retaper. Mais autrement, mon BPCO, ben je sais que, depuis 2007, que le *Docteur D.* m'a dit qu'ils me transplantaient pas, que c'était comme un escalier, chaque fois que je prenais une grippe ou un truc, ça allait descendre les marches. J'ai essayé dans la mesure du possible de rester le plus sage possible, d'éviter de prendre des crèves pas possibles, mais enfin bon, ça a l'air d'aller, quoi. On est en 2016, ça fait neuf ans maintenant, je m'estime encore, gâté d'être là quoi.

**C : Donc, donc pas de greffes, c'est ça, vous disiez ?**

**G :** Non, non, ben, j'ai fait en 2007, tous les, tous les tests pour être greffé, et pis ça a duré le, l'étude a duré jusqu'à, ben ils m'ont donné, en février 2008, la réponse définitive. Les médecins, l'équipe de X *(ville)* voulait pas me greffer, y avait trop de risques. Y avait 70% de chance que j'y reste. Et pis l'équipe de X *(autre ville)* disait, moitié-moitié. Alors bon. J'étais assez déçu sur le moment, mais enfin bon, comme j'allais à la physio à X *(hôpital)*, les, les infirmières de X *(hôpital)*, qui donnaient les cours de physio, m'ont dit : « non, non, mais t'as de la chance, c'est mieux, reste comme ça. Tu verras, la greffe, c'est pas ».

Mais je me réjouissais d'être, je me suis dit : « tiens, ça va donner le tour ». Pis j'ai connu une personne qui a été greffée, j'ai vu ce que c'était, c'était vraiment dur pour lui. Bon il est décédé deux ans après. Mais bon, c'est vrai que, quand je me dis ça fait sept ans maintenant, depuis 2007, ça fait neuf ans, je suis toujours là, c'est bien ! Je connais xxx, je connais, j'ai connu une seule personne qui a été greffée, c'est tout. Mais bon…

**C : Mais voilà, pas, pas pour vous, ouais.**

**G :** Non, ben pour moi, je suis pas greffé. Pis j'ai fait sa connaissance par sa femme qui était avec nous aux cours, qui était aussi un BPCO…

**C : Parce que vous faites des cours ?**

**G :** J'avais, ouais, à l'époque où je me déplaçais encore. Maintenant, de la physio, j'en fais plus du tout. C'est peut-être pour ça aussi que je peux plus me bouger, je fais plus d'exercices physiques, plus rien du tout. Faudrait que je recommence de la physio, mais comme j'ai autant, j'ai pas beaucoup de volonté.

**C : Et, et justement pendant ces, ces visites des, des infirmières, qu'est-ce qui a pu aider ou non, pour ces symptômes physiques, de la BPCO ?**

**G :** Ben, rien… Rien, parce que j'allais, non j'allais plus, ça, ça date, y a trois ans, non j'allais plus déjà à la physio à X *(hôpital)*. Mais j'allais encore à X *(lieu)*, mais pas longtemps. Mais je faisais plus de physio, bon j'avais plus de facilité à bouger, je sortais plus facilement. Maintenant, c'est un peu, c'est un peu le, le, ouais, la flemme. Et pis bon, surtout, c'est que j'ai, j'ai vraiment de la peine à souffler dehors. Il faut vraiment, comme maintenant, un temps qui soit idéal, pour que j'aie pas besoin de me mettre sur deux litres et demi dehors pour respirer, quoi. Mais dès que je marche ou je fais un moindre effort, j'ai, je suis obligé d'augmenter mon taux d'oxygène, quoi.

**C : Donc c'est, c'est difficile dehors ?**

**G :** Oui.

**C : Oui. Pour marcher et pour les exercices physiques [aussi, la physio.**

**G :** Ouais.]

**C : Et… Vous m'avez dit aussi que ces visites, ça pouvait être bon pour le moral, ou, un poids ?**

**G :** Qui ? [Quand elles venaient ?

**C : Les visites] de l'infirmière.**

**G :** Ouais, c'était agréable. Mais bon pour le moral, je suis un solitaire, alors bon c'était, je prenais ça comme elle venait, quoi. J'entends, je me réjouissais pas spécialement d'être, qu'elle, qu'elle vienne. C'était agréable, quoi, quand elle venait, ben, chacun faisait son job, moi je me confiais à ce qu'elle, à ce qu'elle me demandait, pis je remplissais les formulaires. On passait une demi-heure, trois quarts d'heure ensemble, pis ça s'arrêtait là, quoi. J'entends c'était pas une *(bruits de travaux) (rires)*.

**C : *(rires)*.**

**G :** C'était pas une, un truc qui me réjouissait à l'avance. Je serais un menteur de dire ça, c'est pas vrai.

**C : Mais c'était quelque chose d'agréable.**

**G :** Exactement ! Ouais, ouais, c'était une visite agréable. J'entends, jamais un jour je me suis dit : « oh merde, ça me dit rien d'y faire. J'enverrai bien, bien tout promener ». Non, pas du tout. J'entends, quand elle sortait d'ici, ben je passais à autre chose, quoi.

**C : Vous pensiez à autre chose ?**

**G :** Ouais, je passais à autre chose, c'était ou penser à quelque chose d'autre, ou xxx *(bruit de travaux)* à autre chose. Mais j'entends, je restais pas braqué sur mon truc, de ma maladie... J'ai jamais été braqué sur ma maladie, à rester brancher dessus.

**C : Vous aimez pas être braqué dessus ?**

**G :** Non, je me dis : « bon ben je suis comme ça, je suis comme ça, c'est tout ! » C'était avant qu'il fallait que j'y pense hein, c'était pas maintenant, que j'arrive au bout qu'il faut que je me lamente sur mon sort… *(bruits de travaux)*.

**C : Et, je sais que parfois, on peut obtenir aussi du, du soutien spirituel. Je sais pas s'il y a quelque chose, qui vous a été proposé pendant ces visites ?**

**G :** Non, pas du tout. Non, pis ici actuellement, je suis pas tellement branché sur la religion alors…

**C : Ouais, c'est pas, pas quelque chose dont vous avez parlé ?**

**G :** Non, pas du tout, non.

**C : Et, aussi parfois pendant, pendant ces visites des infirmières, elles ont pu parler des, des types de soins qu'on souhaite en fin de vie, [voilà, ça.**

**G :** Ouais, ben ça] elle m'avait dit ouais, probablement. Bon, moi j'ai demandé, y a, y a tout un dossier sur moi qui est pas d'acharnement thérapeutique, il faut pas qu'ils essaient de, je veux être entubé, mais pas qu'ils essaient de me rester en vie pendant 30 sem' euh 30 jours, alors que…

**C : Donc, ça vous en avez parlé ?**

**G :** Ouais, ouais, c'est fait, j'ai rempli des papiers, tout est fait, c'est, c'est dans un classeur là-bas.

**C : Donc vous avez rempli ça avec [les infirmières.**

**G :** Oui, oui.]

**C : Ce que [vous souhaitiez.**

**G :** Sur l'acharnement] thérapi' thérapeutique et pis... *(bruits de travaux)*.

**C : Ouais. Vous avez, vous avez fait ça.**

**G :** Oui.

**C : Et, comment ça s'est passé ces échanges autour de, de ça ?**

**G :** Autour de ?

**C : Autour des soins en fin de vie ?**

**G :** Ben c'est des choses courantes à parler normalement. Il faut pas, faut pas se leurrer, c'est, c'est des trucs qu'il faut penser. Je me rappelle, c'est que y avait ma fille qui était là aussi. Parce qu'ils m'avaient posé la question, de savoir si je voulais me faire autopsier. Moi j'étais pour. Ma fille m'a dit qu'elle était contre, alors j'ai rayé le « pour », pour. Elle m'a dit : « je veux garder quand même une image de toi, à peu près entière, pas tout couturé ». J'ai dit : « bon ok ». Toute façon je me fais brûler, alors… *(bruits de travaux).* Mais bon.

**C : Ça, ça y avait, ça y avait votre fille qui était là aussi.**

**G :** Ouais.

**C : Et qui a, qui a pu donner son avis.**

**G :** Ouais, quand on en a parlé quoi, c'est tout, hein. Je sais pas si c'était le jour où on l'a rempli, mais c'est quand on, elle est revenue pis qu'on en a reparlé, qu'elle a… qu'elle a donné son opinion quoi *(bruits de travaux)*.

**C : Ouais, ouais, ouais. Donc ça, voilà c'est, c'est quelque chose que vous avez, que vous avez fait. Et, de manière générale, quelle a été pour vous l'utilité des, de ces visites des infirmières, [une fois par mois ?**

**G :** Mais c'est-à-dire l'utilité,] je comprends pas ? Comme je vous ai dit tout à l'heure, c'était un truc, un, un deal que j'ai passé avec eux, entre elles et moi, qu'ils venaient ici, on, je remplissais des papiers, je répondais à ses questions. C'était, bon, un truc que j'ai fait, pendant une année. On m'a posé la question si j'acceptais ou j'acceptais pas. *(Bruits de travaux)* xxx dans la mesure où ça me gênait pas ! Autrement, ça m'a rien apporté d'autre.

**C : Voilà, ça, ça vous gênait pas.**

**G :** Non.

**C : Mais ça vous a pas apporté quelque chose de.**

**G :** Non, pas du tout. Non plus, non.

**C : De spécial.**

**G :** Non.

**C : Ouais, ouais.**

**G :** Je suis pas tombé amoureux d'une infirmière, ça a rien changé à ma vie, non *(rires)*.

**C : *(rires)*. Et, donc qu'est-ce que vous auriez souhaité, peut-être recevoir en plus ou en moins, pendant ces visites ?**

**G :** Mais, recevoir en plus ou en moins… Non, rien ! J'ai besoin de rien. Comme j'ai dit, c'était un job que j'ai accepté de faire, je l'ai fait dans la mesure du possible. De <u>plus</u>, je sais pas ce que j'aurais pu demander de plus, hein !...

**C : Donc, c'est quelque chose que vous avez fait, voilà.**

**G :** J'ai fait.

**C : Ouais. Mais pas de.**

**G :** Non, j'ai fait ça sans autre, quoi. J'attendais rien de plus. Je sais que ça allait pas toute façon améliorer ma santé. Ça je me suis dit clairement, et ça gêne en rien. Ça aidera peut-être améliorer celle des autres, pour autant que vous arriviez au bout de ces études, parce que si ça fait déjà trois ans que ça dure. Dans 20 ans, vous arriverez au bout peut-être *(sourire)*.

**C : *(rires)*. Donc voilà, plus pour, pour les autres.**

**G :** Ouais, justement.

**C : Oui, oui. Plus que, que pour vous.**

**G :** Ouais, ça c'est sûr.

**C : Donc vous, vous saviez que ça allait pas améliorer votre santé, [d'avoir.**

**G :** Non,] ça d'entrée, je savais. Il faut pas se leurrer hein, c'est une étude, où on prend des gens. Ils savent assez quand ils nous demandent de remplir ces trucs, qu'on n'est pas sur la pente guérissable, on

est plutôt sur la pente descendante... Je fais peut-être pas preuve d'un optimisme *(rires)*, mais enfin, c'est la vérité hein, parce que s'ils commençaient à faire des études sur les gens bien portants.

**C : Donc plus, plus pour les autres, [que.**

**G :** Ouais.

**C : Que pour vous. Et… je sais qu'aussi on, on peut recevoir du soutien au niveau psychologique, est-ce que c'est quelque chose, pendant ces visites des infirmières que ?**

**G :** Non.

**C : Non…**

**G :** Non, soutien psychologique ? Non… Ouais c'est un peu comme, quand elles venaient, c'est un peu comme les, le rapport que j'ai avec mon aide ménagère, qui vient ici tous les lundis pendant deux heures de temps, quoi. On discute de la pluie, du beau temps, à bâtons rompus, mais quand elle a sorti d'ici à 13h20, à 15h20, elle est partie quoi, c'est fini, j'ai tiré la porte. Et puis je suis seul, et puis, puis je passe à autre chose.

**C : C'est, pour vous, c'est un peu le même type de rapport ?**

**G :** Exactement. Même, même style de relation. C'est-à-dire, ben comme vous sortirez d'ici, j'aurais accepté avec vous de discuter, mais, ça va pas me remettre en question, hein.

**C : Ouais. Vous penserez à autre chose après.**

**G :** Ouais. Ouais, non, ça va pas me remettre en question sur, sur ma maladie, sur tout ce qu'il y a, quoi… Ça doit pas être évident pour vous de faire ce genre de boulots. [Si vous avez 50 personnes qui vous répondent comme moi *(rires)*.

**C : Non,] *(rires)* non, c'est, c'est intéressant d'avoir les avis de tout le monde, qui sont différents, par rapport à ça, [c'est ça qui.**

**G :** Ah bon quand même,] y a quand même des gens différents.

**C : Oui, oui, y a des, des avis, c'est intéressant de voir, comment chacun a vécu ça justement, ces visites des infirmières, et, voilà. Donc, peut-être voilà, de quoi auriez-vous eu besoin ou de quoi auriez-vous besoin aujourd'hui ?**

**G :** Ben ce que j'aurais besoin aujourd'hui ? Ben c'est d'aller mieux, quoi. Mais ça, c'est trop tard, alors d'aller mieux. Peut-être être faire plus d'efforts physiquement. Ça *(bruit de travaux)* améliorerait peut-être mon état physique et un peu mon moral, mais autrement.

**C : De faire de l'activité physique, [ça améliorerait ?**

**G :** Ouais,] ouais mon moral. Et puis ça me permettrait de faire autre chose. De voir les choses peut-être un peu différemment.

**C : Donc ça peut-être, ça pourrait être quelque chose.**

**G :** Ouais, ça dans l'immédiat, oui. Mais ça a rien avoir avec l'étude xxx de l'infirmière. C'est, c'est un, un, un problème que j'ai actuellement. Ça a rien avoir avec *Madame Chappuis (infirmière de recherche)* et l'équipe d'infirmières qui sont passés y a deux ans en arrière, hein.

**C : Oui, oui. Mais ça c'est quelque chose dont vous auriez besoin <u>maintenant</u>, [aujourd'hui ?**

**G :** Oui.]…

**C : Donc voilà, si, si je résume un peu. C'est quelque chose, qui a, qui a été agréable, ces visites**

**des infirmières, vous aviez pas d'attentes pour vous, c'était voilà, vous avez participé plus pour les autres, pour peut-être [améliorer.**

**G :** Ouais c'était.] Améliorer le, comme je vous ai dit, améliorer le, la maladie dans le cadre de, si on arrive à, à progresser ! Mais j'attendais rien du tout pour moi.

**C : Ouais. C'était plus pour, pour faire progresser la, la maladie. Et, et donc, voilà, au niveau aussi des, des symptômes, vous avez pu recevoir ce X *(médicament anxiolytique)*. Ça c'est quelque chose au niveau médicalement, [que vous avez.**

**G :** Ben c'est-à-dire ça m'a,] vu, changé, de prendre, parce que avant je prenais un, un X *(autre médicament anxiolytique)*. Il m'a expliqué que ça mettait pratiquement 12 heures pour commencer à m'en aller, que le X *(médicament anxiolytique)*, en une demi-heure ou une heure de temps après, ça commençait un peu à voir les. Et c'est vrai. C'est le seul truc qui m'a changé, hein.

**C : Donc ça a juste.**

**G :** Un côté bénéfique, voilà ! *(sourire).*

**C : Ouais, *(rires)*, [un côté bénéfique.**

**G :** Un côté bénéfique.]

**C : Ouais. De, voilà de, vous disiez pour, pour être moins [stressé quand vous devez vous préparer.**

**G :** Voilà, c'est ça, ouais.] Ouais.

**C : Donc, donc ça y a eu. Et, autrement, vous avez aussi pu parler des soins en fin de vie, c'est quelque chose que vous avez pu faire pendant, pendant ces, ces visites notamment avec votre fille. Mais autrement, de manière générale, pas vraiment d'utilité pour vous, si je comprends bien, de ces visites, à part pour ce médicament-là. Mais.**

**G :** Comment, pour moi ?

**C : Voilà, par rapport à, vous disiez, par rapport à l'utilité de ces visites.**

**G :** Ça m'a pas apporté grand-chose, non.

**C : Voilà. Ça vous a pas apporté grand-chose. C'était quelque chose d'agréable, mais ça vous a pas apporté.**

**G :** C'était agréable d'avoir la visite de cette dame quoi, de parler de la pluie et du beau temps. Mais j'entends, ça m'a rien apporté, en me disant, en optimisant, en me disant, en optimisant, en me disant : « je vais encore vivre pendant 10 ans ». *(bruit de travaux)* xxx.

**C : On va attendre *(rires)*.**

**G :** Ouais, c'est atroce *(en parlant des bruits de travaux)*.

**C : Est-ce qu'y a, voilà, y a, y a quelque chose d'autre que vous aimeriez rajouter par rapport à, à cette étude, ou, par rapport à ce que ça vous a apporté, pas apporté ? Est-ce qu'y avait autre chose, qui vous vient en tête ?**

**G :** Non, non, parce que je saurais pas quoi vous dire, entre parenthèses...

**C : Pour maintenant, voilà, vous m'avez dit l'activité physique *(bruit de travaux)*.**

**G :** xxx aujourd'hui, hein. C'est pas, ça a rien à voir avec ce qu'y avait y a deux ans en arrière. Mon activité physique, maintenant, ça serait effectivement quelque chose que je devrais faire avec un peu plus

d'envie. Mais bon, y a, c'est aussi un peu le moral qui joue et pis c'est tout, pour se sentir, avoir envie de le faire.

**C : Ouais, c'est ça. Est-ce qu'y a autre chose que vous aimeriez encore, encore dire ?**

**G :** Non.

**C : Non.**

**G :** Non euh. *(sonnerie de téléphone)*. C'est juste ma fille.

**C : Prenez. Répondez seulement.**

**G :** *(conversation téléphonique)*.

**C : Donc voilà rien de, d'autre que vous aimeriez encore rajouter [par rapport à ça ?**

**G :** Non, non.] Je pense que ça, j'espère que ça va apporter quelque chose pour la médecine, pour le, dans le cadre du BPCO, et pis c'est tout, hein. Parce qu'on était combien à participer à cette ?

**C : Une cinquantaine.**

**G :** Ouais, ça va encore prendre du temps, parce qu'y aura, régler tous ces trucs, mettre après tout ça sur papier, les 50 réponses que vous avez. Pis les analyser, alors ça c'est encore autre chose.

**C : Ouais, on va faire ça prochainement.**

**G :** xxx, parce que vous allez partager ça comment ? Selon quels critères des réponses ?

**C : Ouais, voilà on va, voir selon ce qui ressort, qu'est-ce qu'on peut en ressortir de, ce que chacun nous a dit. Après, ça c'est une partie de l'étude, pis y a l'autre partie, qui est déjà en train d'être analysée, avec les questionnaires, auxquels vous avez répondu, donc ça c'est en train d'être finalisé. Et là c'est cette partie plus sur le, le vécu et les attentes.**

**G :** Mais c'est qui qui s'occupe de ça ? C'est ?

**C : Alors c'est le.**

**G :** C'est *Docteur C. (soins palliatifs)* ou c'est *Professeur A. (pneumologie)* ?

**C : Alors aussi, voilà *Docteur C. (soins palliatifs).***

**G :** *Professeur A. (pneumologie)* il participe pas du tout, lui là-dessus.

**C : Oui aussi, voilà.**

**G :** Ouais.

**C : Y a aussi le *Professeur A. (pneumologie)*, *Professeur B. (soins palliatifs)*, *Docteur C. (soins palliatifs)* qui sont impliqués dans cette partie de l'étude.**

**G :** D'accord.

**C : Mais, merci en tout cas d'avoir pris le temps de répondre.**

**G :** C'est rien, [c'est rien !

**C : Nous donner] votre avis sur, sur cette étude. Merci ! Je crois qu'on peut, éteindre la.**

# Participant n°10

Nom d'emprunt : Luc
Âge : 73 ans
Sexe : M
Groupe : Intervention

**C : Donc, vous avez participé à une étude qui avait pour objectif de comparer le traitement habituel de votre maladie pulmonaire, la BPCO, avec une prise en charge précoce, soutenue et intégrée. Vous étiez dans le groupe bénéficiant de cette prise en charge globale spécialisée. Et vous avez reçu la visite d'une infirmière, une fois par mois pendant un an.**

**L :** Moi elle m'a rien, j'ai pas, j'ai rien eu ! Elle m'a apporté de médicaments, ou de me dire. J'ai rien, elle m'a rien fait cette dame ! Ils m'ont fait quoi ?!

**C : Donc, vous aviez voilà, une infirmière qui venait une fois par mois, [pendant un an.**

**L :** Mais, mais qu'est-ce qu'elle est venue me faire ?] Elle m'a rien fait ! J'ai continué comme j'ai toujours fait ! Donc elle venait là pour me dire bonjour, c'est tout, quoi ! Je sais pas ! Parce que je me rappelle pas avoir changé de traitement, ou, ou quoi que ce soit, je m'en rappelle pas ! J'ai le même traitement depuis le début moi, alors, je sais pas pourquoi, je sais pas, non. Mais je peux pas dire que ça va mieux à cause d'elle ou pas, j'en sais rien.

**C : Voilà, vous avez pas changé votre traitement ?**

**L :** Non ! Rien du tout ! Mais ils m'ont apporté quelques chose, ces, ces, ces, ces infirmières, ou quoi ?

**C : Donc voilà, c'était, elles venaient.**

**L :** Ils ont pas apporté un truc pour essayer ou, non rien ?!

**C : Donc elles, elles venaient une fois par mois, [pis elles évaluaient.**

**L :** Oui, mais pour faire quoi ?]

**C : Elles évaluaient vos besoins, en termes de gestion des, des symptômes physiques, ou, ou psychologiques, ou, voir de quoi vous aviez besoin.**

**L :** Je m'en rappelle plus de ça… C'est, c'est que ça m'a pas trop marqué. Bon.

**C : Et, et, qu'est-ce que vous attendiez en participant à cette étude ?**

**L :** Ah, moi y a une chose, moi j'attendais rien du tout. Moi ce que je voulais c'est, c'était, je pensais qu'. Dans ma tête, moi d'abord, j'ai dit : « bon, j'ai eu ça. Je vais aider pour qu'ils puissent avoir des, des données, et tout ». C'est tout hein ! Ça, ça, ça s'arrête là. Et puis, je me suis dit, peut-être si y a des trucs, on sait jamais ça peut, nous rendre service à nous. Mais, j'ai pas eu, j'ai rien eu de plus. Ni en plus, ni en moins, en fait. J'ai perdu beaucoup de temps, mais bon, si ça peut faire avancer le, le schmilblick c'est le principal. Moi je l'ai fait pour faire avancer, pour qu'ils puissent avoir des données. Voilà, c'est tout ! Mais, si y a eu, moi je leur ai donné à ces dames les résultats. Vous arrivez aujourd'hui, vous me dem', moi j'ai, je peux, je peux plus rien vous donner ! Je sais pas ! Je ne me souviens pas ! Mais ça devait pas aller ni plus mal, ni, ni mieux, parce que, ça fait, jeudi, ça fait deux ans que j'ai pas été à l'hôpital. Je vais chez le pneumologue tous les mois, bon des fois j'ai fait un début de pneumonie, un petit peu, mais léger. Je m'y suis pris, bon c'est bon mais autrement, j'ai rien eu ! J'ai pas eu de problèmes. Le seul problème que j'ai c'est que je peux toujours pas bien marcher, je peux toujours pas bien souffler, et pis c'est toujours pareil. Donc, pis ça, ça, ça, ça, ça, ça va rester donc, on peut pas, elles peuvent venir tous les mois, ça changera pas hein… malheureusement !

**C : Donc, pas de différence ?**

**L :** Non. Non, je dirais que c'est même, je vais un peu mieux depuis, depuis une année je vais mieux.

Parce que j'ai perdu du poids, c'est tout. Mais autrement, c'est clair qu'en perdant du poids, ça va un peu mieux mais, c'est pas facile hein. Alors voilà, c'est, c'est tout ce que je peux dire, en tout cas. La seule solution pour moi, c'est de perdre du poids, si je veux aller mieux. Respirer mieux, perdre du poids, c'est la seule chose.

**C : [C'est perdre du poids.**

**L :** Ah ouais, c'est, c'est] tout, voilà.

**C : Vous vous souvenez plus très bien de ces visites ?**

**L :** Alors là, alors pas du tout ! Pas plus très bien, pas du tout !

**C : D'accord.**

**L :** Ça m'a pas marqué ! Je me rappelle, je me rappelle vaguement qu'elles étaient venues, mais je pourrais pas vous dire on a discuté de ci ou de ça, j'en sais rien, je me rappelle plus. Alors voyez, je vous dis, c'est trop espacé, pour moi ! Moi j'avais complètement oublié tout ça, je pensais que c'était fini, du reste. Déjà c'était assez long, hein ! Parce qu'ils disaient : « oui, faut, vous avez commencé, faut finir ». Bon d'accord, c'était pas toujours facile. Je l'ai fait, mais après maintenant ça, ça, ils vont continuer encore pendant maintenant dix ans à nous, à nous casser les pieds, ça c'est sûr ! Parce que c'est pas fini, vous dites c'est fini, c'est pas fini ! Après vous, y en aura une autre, ça c'est sûr ! Mais c'est, pour moi c'est fini, j'arrête parce que, ça sert à rien ! C'est de l'argent perdu de l'Etat de, de, du contribuable, ça. C'est vrai !

**C : Pour vous, ça sert [à rien ?**

**L :** Mais non,] ça sert à plus, pour moi ça sert à quoi que vous, que vous veniez me dire que, que y a deux dames, trois dames qui sont venues, si je m'en souviens pas ! Moi ce qui serait valable, c'est si on me dit : « écoutez on a, on a, on a ça éventuellement qui est, qu'on sert à l'hôpital, à l'heure actuelle, qui est très bien ! Vous devriez essayer ! » Mais, on me dit jamais ça ! Voyez, donc alors. Moi ce que je voudrais c'est avoir un produit qui me dise : « on a ça, ça fait du bien ! C'est mieux ! On a trouvé ! », « Bon, vous avez trouvé quelque chose de mieux, on le prend », mais autrement, quoi ! Pour moi, c'est ça le progrès hein. Bon, y en a déjà eu beaucoup là-dedans hein, faut, je reconnais. Mais, moi je sais que je peux pas faire grand-chose, donc hein, on se comprend, hein, voilà. Mais je me souviens plus, je me souviens plus, vous pouvez pas m'enlever, je veux pas raconter des, des, des, des bêtises pour dire, parce que je sais pas, alors, franchement. Je peux même pas vous dire comment elles étaient ces dames. Sûrement gentilles, mais…

**C : Mais ça, vous aimeriez plutôt qu'on vous propose un produit ?**

**L :** Non, j'aimerais pas plutôt, je dis, ça serait bien ! Mais bon, je sais que c'est pas possible. Si y en avait, mon, mon pneumologue me l'aurait déjà dit, hein ! Mais, moi je me soigne, et pis c'est tout ce que je peux faire. Le mieux possible, j'espère. Et voilà.

**C : Oui. Et pis, voilà, vous disiez que vous avez participé pour faire avancer.**

**L :** Oui, moi j'avais participé pour essayer de, qu'ils trouvent des solutions. Parce que c'est comme ça qu'ils vont trouver, c'est, c'est, faut participer, hein. Parce qu'autrement, on stagne. Mais, voilà, ma mémoire s'est, ça s'est effacé de ma mémoire ce truc-là. C'est trop long, je suis désolé. Ça, vous pouvez leur dire, que y a trop de temps qui passe, après la fin pour, voyez ? Moi j'ai tellement de médecins que je vais voir, je vois tellement d'infirmières, de docteurs, voyez ?

**C : Oui c'est, c'est trop long.**

**L :** Mais l'autre j'avais un rendez-vous au X *(lieu)* avec un, un gars, je me suis trompé de gars, j'ai pris celui de l'année d'avant. Voyez, donc, vous vous rendez compte hein ! Je croyais que c'était toujours le même, c'était. Alors, les infirmières, quand y en a trois ou quatre différentes. Après, j'ai mon frère à l'hôpital, je vais, j'irai voir l'infirmière, son médecin, le mien, bon. Ça, ça, vous savez c'est, pour finir, *(rires)*. Voilà. Non, mais autrement ça va, je peux pas, je peux pas dire, je peux pas dire que c'est, c'est, c'est ça qui m'a fait du bien ! Je peux pas dire ça, moi. Moi, ce qui m'a fait du bien, c'est, c'est moi, parce

que je me suis soigné, mais autrement. Moi je sais pas ce qu'elles m'ont fait, elles m'ont rien fait ces dames. Qu'est-ce qu'elles, elles, elles devaient me faire quoi, en fait ?

**C : Elles venaient évaluer vos besoins en termes de gestion des symptômes physiques de, de la maladie.**

**L :** C'est-à-dire ?

**C : Voilà, pour voir comment vous gérez les symptômes, comme peut-être la fatigue, ou les douleurs, voir peut-être à [adapter les traitements si nécessaire.**

**L :** Ouais, mais, elles ont jamais rien fait,] même quand j'avais très mal.

**C : Et y avait aussi, parfois du soutien au niveau psychologique, ou ?**

**L :** Parce que moi je veux dire une chose, y a deux ans en arrière, je pouvais plus marcher, hein. Personne a rien fait pour moi. Ni les infirmières de l'hôpital, personne !... J'ai dû me débrouiller, j'ai été je sais pas combien de fois à l'hôpital, pour discuter avec des médecins et tout, et tout, et tout, et tout ! Mais ils m'ont jamais dit qu'ils allaient m'envoyer quelqu'un qui allait me, non ! Alors à quoi ça sert ? Mais enfin ! Je vais pas rentrer dans les détails moi.

**C : Mais, je sais aussi que parfois elles ont pu peut-être parler des types de soins qu'on souhaite en fin de vie, quand.**

**L :** Ouais, ouais, alors moi, ça presse pas. C'est un truc que j'évite ça, pour le moment. Je suis pas en fin de vie, j'espère, en tout cas pas hein !

**C : Non mais de, elles ont pu peut-être discuter de.**

**L :** Ouais, ouais, moi c'est le X *(pont)*, vous pouvez marquer *(rires)*. Non, je charrie, mais, non, non. J'en sais rien, j'en sais rien, en fin de vie ! En tout cas une chose, je sais pas, je peux pas en parler maintenant, j'en sais rien ! Si je souffre trop, je demanderais EXIT, et pis si ça va, ben, on sait pas. Ça on sait jamais, hein. Mais j'espère vivre encore au moins quelques années bien ! Enfin le mieux possible, parce que, bien avec ça, c'est, c'est un peu difficile. Mais je veux dire, au moins, le mieux possible, quoi.

**C : Ça, voilà, vous vous souvenez plus d'avoir parlé de ça ou pas avec les infirmières ?**

**L :** Non. Il me semble que j'en ai parlé une fois, mais je sais même pas avec qui. Oui ben, ma mère est, est morte comme ça, voyez. Mon frère est, en ce moment, est très, très malade. Il a aussi, il a aussi l'oxygène, mais il a pas la BPCO lui, il lui manque un poumon hein. Alors, voyez, on parle de ça aussi. Y a tout un peu qui, qui se chevauche, qui, qui se mélange, ohlàlà. Mais moi je suis pas pour, alors, bon. Pas très, pas très chaud pour, quoique, chacun fait comme il veut, hein ! Moi…

**C : Vous êtes pas trop pour ?**

**L :** Non, je suis pas trop pour EXIT moi. Mais je dis chacun fait ce qu'il veut, je, je ! Ma mère elle a pris EXIT. Si ça avait été que moi, j'aurais dit non. Mais elle est libre, c'est pas moi qui vais aller à la police pour dire : « non, non, non ». Je suis pas comme ça alors. Elle avait envie, elle avait envie. C'est qu'elle en avait vraiment marre ! Bon, ben voilà. Moi je laisse faire les gens, leurs volontés. Mais, moi… Je suis pas chaud, chaud. Bon enfin, bref, ça c'est une autre.

**C : C'est pas, pas quelque chose dont, vous vouliez parler.**

**L :** Non.

**C : D'accord. Et, donc voilà, par rapport à l'utilité de ces visites des infirmières une fois par mois.**

**L :** Encore une fois, l'utilité, je me rappelle pas ce qu'elles m'ont dit ! Alors l'utilité, oui ou non, j'en sais

rien ! Je m'en souviens plus ! Ça m'a pas marqué ! Ou alors si ça m'a marqué, ben maintenant j'ai oublié, voilà, c'est tout.

**C : Voilà c'est, c'est quelque chose, c'est difficile à s'en souvenir, parce que ça fait longtemps que.**

**L :** C'est trop, ben oui, maintenant c'est, ça, ça fait un an et demi, et tout ça ! Moi j'ai, j'ai passé à autre chose, voyez ? C'est tout. Je me souviens plus ce que j'ai parlé avec. Là, le suivi est trop, trop long, à mon avis. Bon, je sais bien qu'ils font pas comme ils veulent non plus, mais je veux dire par là, c'est un peu long. Alors moi j'ai, j'ai, j'ai oublié, voilà.

**C : Donc, donc voilà, si, si je résume un peu, vous aviez participé à cette étude pour faire [avancer.**

**L :** Oui !] Oui, oui. J'avais dit : « je veux bien le faire ». Parce que je voulais pas le faire au début, mais après j'ai dit : « je vais le faire quand même, pour si ça permet de », voyez. Voilà, c'est tout. Mais maintenant je regrette presque de l'avoir fait, parce que, d'abord ça m'a avancé à rien du tout. Et, j'ai perdu encore du temps. Mais j'espère que eux, ne perdent pas, les médecins au moins, eux en gagnent, c'est déjà bien. C'était le but, pour moi, voilà. Mais moi j'ai rien gagné là, j'ai perdu beaucoup de temps, pour rien, quoi. Alors j'en ai plus beaucoup à perdre déjà, il faut, *(rires)*. Non, je plaisante un peu là, c'est pas très juste, mais. Je veux dire, je n'ai absolument aucune, aucun résultat à donner, ou qui, n'importe, voilà, vous avez compris, j'en n'ai pas, voilà ! Je peux pas dire : « ça, ça m'a, ah oui là, ah pis alors là oui! » Non, non je me souviens pas ! Donc.

**C : Et ça vous a fait plutôt perdre du temps ?**

**L :** Non, j'ai pas perdu temps, puisque j'ai dit que je le faisais, mais je veux dire par là que je n'ai pas eu d'avantages, dans ma santé, à cause de ça, voilà. Mais si la médecine a eu un avantage, ben tant mieux ! C'est le but ! C'est pour ça que je l'ai fait, voilà. Moi je sais que, je peux pas faire des miracles.

**C : Vous avez pas eu d'avantages pour vous, mais si ça a pu faire avancer les choses.**

**L :** Ben c'est ça que je dis, oui, tout à fait !

**C : D'accord, oui. Et, est-ce que y aurait quelque chose que vous aimeriez encore rajouter par rapport à ça ?**

**L :** Mais qu'est-ce que vous voulez que j'ajoute, quoi ?! Rien. Oui, si vous trouvez un autre, un bon médicament, faites-moi signe ! Je prends moi, *(rires)*. Quelque chose que j'ai plus besoin de prendre ça *(il regarde le réservoir d'oxygène)*. Ils trouveront un jour, hein ! Ils trouveront un truc qu'on met sous la peau, qui fera de l'oxygène, pis qu'on n'aura plus besoin de remplir, et tout. C'est sûr, ils trouveront. Mais pour le moment. Je sais pas si, vous étiez pas née vous, mais moi oui. Y a… 60 ans en arrière, c'était, ça c'était des poumons d'aciers à l'époque, hein. Les gens ils rentraient dedans, on voyait que la tête, ils rentraient carrément dedans. Je m'en rappelle, j'ai vu ça moi ! J'ai vu ça à l'hôpital. Y avait un hôpital, à la rue de X *(nom de rue)*. Ça s'appelait comment l'hôpital, comment est-ce qu'il s'appelait cet hôpital. Bon c'était, une annexe d'hôpital cantonal, mais. Je me souviens toujours, ça me, l'image m'est restée. Ce, ce gros truc, on aurait dit une citerne pour la benzine. On voyait la tête du gars en haut comme ça, ouais, je m'en rappelle. Donc, y a beaucoup de progrès, parce que maintenant on a la petite bonbonne, on peut sortir, voyez, donc. Mais faudrait qu'ils, qu'ils trouvent encore plus petit ! Mais ça.

**C : Donc y a eu des progrès.**

**L :** Ah oui, ben heureusement ! Ah ben oui, heureusement, hein. Ah oui.

**C : Et de, de quoi auriez-vous besoin aujourd'hui ? Vous disiez perdre du poids.**

**L :** J'ai rien besoin moi. J'ai, oui, j'ai besoin de perdre du poids. Mais c'est, c'est, c'est pas eux qui peuvent le faire pour moi, donc. J'ai été dix fois à l'hôpital pour leur demander s'ils pouvaient m'envoyer quelque part, pour que je perde du poids et tout, parce que ça n'allait vraiment pas. Mais ils

ont jamais rien fait pour moi, donc. Ça existe pas, ils ont pas, soi-disant ! Alors voilà bon.

**C : Ils ont rien pu faire pour vous, à l'hôpital.**

**L :** Oui, moi j'aurais voulu perdre du poids ! Même qu'ils me gardent à l'hôpital, 15 jours, pour perdre quatre, cinq kilos, que je sois mieux ! Ben non, ils ont jamais fait ça ! Ils, ils font pas pour des, je veux dire pour, ils vont pas prendre quelqu'un à l'hôpital pour le faire maigrir, voyez ? C'est pas le but de l'hôpital ça ! Ce qui est presque logique, en fait *(rires)*. Voilà, je maigris tout seul, le mieux que je peux.

**C : Donc voilà vous, vous suivez [votre traitement, et.**

**L :** Ouais, ouais bien sûr,] bien sûr.

**C : Et voilà, vous avez pas de besoins.**

**L :** J'ai un traitement de base, depuis, depuis que, depuis dix ans là. Pis je le suis tous les jours, pis voilà.

**C : Est-ce que y a autre chose que vous aimeriez encore rajouter par rapport à, à [votre participation à cette étude ? Les visites ?**

**L :** Qu'est-ce que vous voulez que je vous dise ?!] Non ! J'ai, j'ai, j'ai rien de spécial à dire, moi, rien, c'est bon.

**C : Donc voilà c'est, ça faisait un peu trop longtemps depuis que ça s'est terminé, et.**

**L :** Ouais ! Voilà, ils ont laissé passer beaucoup trop de temps maintenant ! Je vous dis, moi j'ai oublié, alors. Ça m'embête, franchement ! J'aimerais bien dire : « ah oui, on avait discuté avec madame, et tout ». Mais je m'en, je peux pas vous dire, je m'en souviens pas. Je veux pas être malhonnête pour vous dire des, des, des conneries, c'est pas mon genre, donc voilà, je peux pas. Vous me demandez ce que je veux, bien sûr que je veux des tas de choses, moi ! Par rapport à, j'aimerais bien pouvoir marcher comme il faut, être plus essoufflé, et pis voilà, bien sûr, mais bon. Tout le monde veut ça, hein alors.

**C : Par rapport à ça, à ces visites, vous, voilà, vous vous souvenez plus. Voilà, à part si y a autre chose que vous aimeriez encore rajouter, je vous remercie d'avoir pris le temps de.**

**L :** Ouais. C'est moi qui vous, je suis désolé de pas pouvoir faire plus, parce que, ça m'embête de, de, de, de, de, de pas pouvoir vous dire : « oui, oui, oui, oui, oui, oui, oui ». Mais… qu'est-ce que vous voulez que je fasse quoi ?! Je peux, je me, je me sens, je me sens presque, presque bête là, parce que j'ai, j'ai pas de, j'ai aucune réponse à donner, et ça, ça m'embête, parce que il me semble que j'ai oublié tout ça. Et je m'en rappelle même pas ce qu'elles ont fait pour moi, je me rappelle même pas qu'elles sont venues. Alors vous voyez hein ? Oui, c'est vague, je, je me rappelle qu'elles sont venues, mais. Est-ce qu'elles m'ont pas fait remplir des, des feuilles, aussi ?

**C : Des questionnaires, oui.**

**L :** Ouais. Ah ouais ça alors, ça, ça m'est resté, ça, ouais. Ouais, ouais, d'accord oui. Mais je les ai remplis les questionnaires, ouais, ouais, ouais, ouais, ouais, ouais.

**C : Non mais je vous, je vous remercie de nous avoir partagé comment ça s'est passé pour vous, et voilà, de, le.**

**L :** Ben ça a dû bien se passer, puisque, puisque je vais mieux depuis que je suis encore là, donc *(rires)*. J'espère que ça s'est bien passé.

**C : Merci en tout cas *(rires)*.**

**L :** Voilà. Je suis désolé de pas faire plus pour vous hein, mais. Je crois pas qu'on arriverait à plus loin, parce que.

**C : Y a pas de problèmes.**

**L :** Autant, mais autant être honnête que hein, vous êtes d'accord ?

**C : Y a pas, y a pas de soucis ! Y a pas de soucis !**

**L :** [Voilà.

**C : Ça faisait un petit moment.]**

**L :** Vous travaillez pour le Professeur ?

**C : Alors.**

**L :** Comment il s'appelle, le Professeur ?

**C : *Professeur B. (soins palliatifs)* et *Professeur A. (pneumologie)*.**

**L :** Ouais, *Professeur A. (pneumologie)*, oui, oui.

**C : Voilà, ouais. [Sur cette étude.**

**L :** Il est bien hein,] ce *Professeur A. (pneumologie).*

**C : Oui !**

**L :** A l'heure actuelle les, les, les BPCO, c'est, vous pouvez pas me répondre, non. Parce que point de vue médical, vous pouvez pas me répondre.

**C : Non, alors malheureusement, étant psychologue et pas [du domaine infirmier, ça, malheureusement.**

**L :** Ouais, ouais, tout à fait, tout à fait, tout à fait.]

**C : Si vous avez des questions, vous pouvez toujours contacter, par rapport à cette étude, aussi le *Docteur C. (soins palliatifs)*. Y avait le contact dans la lettre, que vous avez reçue, aussi.**

**L :** Ouais. Moi j'aurais voulu une fois, avoir un rendez-vous avec le *Professeur A. (pneumologie)*. Mais c'est très difficile d'avoir un rendez-vous avec lui. Ce que je conçois, parce que il doit être très demandé.

**C : Pour, pour discuter un peu de ?**

**L :** Ouais, j'aimerais bien, au sujet de ma maladie. Parce que y a des trucs que je comprends pas, alors bon, faudrait que je me, que je me renseigne. Parce que mon, mon pneumologue, c'est pareil. Ils ont jamais le temps. Ils vous soignent et tout, mais ils ont jamais le temps. Ils, c'est vrai qu'ils sont surchargés, parce qu'il faut qu'ils partent quand même 15 jours par mois en vacances, hein. Le mien est tout le temps en vacances. Il bosse 20 jours, il part 10 jours. Mais il a raison, hein, s'il peut *(rires)*.

**C : Donc voilà, ils ont pas le temps forcément.**

**L :** Non, non, mais je, je, je vais peut-être un peu fort là. Mais c'est lui qui s'occupe, il est bien, moi j'ai un bon, bon pneumologue. Peut-être vous le connaissez, c'est *Docteur D. (pneumologue)*.

**C : Non, je connais pas.**

**L :** C'est celui qui s'occupe du… du, comment est-ce qu'on appelle ça, du… truc hyperbare là, à l'hôpital, vous savez ?

**C : Je saurais pas vous dire.**

**L :** Vous savez ceux qui, par exemple, quand vous êtes dans l'eau, pis que vous remontez trop vite, faut aller dans le caisson. Ils vous mettent dans un caisson hyperbare. C'est, y en a un à l'hôpital, maintenant. Vous avez jamais vu ?

**C : Non, j'ai pas été dans cette partie.**

**L :** Ils vous montrent même pas ça ?! Eh ben dites donc ! Alors voilà, c'est pour ça que je dis, non, il est, il est bien, c'est, c'est un, c'est un bon bosseur, mais il part souvent, *(rires)* il est jamais là.

**C : Il part souvent en vacances.**

**L :** Non, pas en vacances, mais il est, il a des stages ici, un stage là, et pis, vous savez ce que c'est les médecins hein.

**C : Mais là, vous aimeriez pouvoir avoir un rendez-vous avec le *Professeur A. (pneumologie)* pour poser des questions ?**

**L :** Ouais, c'était surtout pour discuter de certaines choses, mais… C'est pas, je téléphonerais si.

**C : En, en termes d'informations, vous souhaiteriez plus d'informations sur [la maladie ?**

**L :** Non,] non j'ai ce qu'il faut, les informations. Je les ai <u>toutes</u> les informations, hein. Avec mon, mon pneumologue, il a tout. Mais, je veux dire par là, l'avis d'un Professeur des fois, c'est bien. Mais bon, y a pas de soucis.

**C : D'accord *(rires)*. Mais merci de, d'avoir pris le temps *(rires)*.**

**L :** C'est moi qui vous remercie beaucoup. Je suis désolé de vous avoir fait venir, déplacer, tout ça pour. Est-ce que je peux vous offrir un café ?

## Participant n°11

| |
|---|
| Nom d'emprunt : Thierry |
| Âge : 56 ans |
| Sexe : M |
| Groupe : Intervention |

**C : Vous avez participé donc à une étude, qui avait pour objectif, de comparer le traitement habituel de votre maladie pulmonaire, la BPCO, avec une prise en charge précoce, soutenue et intégrée. Vous étiez dans le groupe bénéficiant de cette prise en charge, globale spécialisée. Et vous avez reçu la visite d'une infirmière une fois par mois pendant un an. Pouvez-vous me raconter comment ça s'est passé ?**

**T :** Ben disons, en bref elles venaient me trouver pour savoir un petit peu l'évolution de ce que je ressentais, mon état de fatigue, mon état de, donc moral aussi. Des fois psy', effectivement, psychologiquement, des fois, on a des hauts, des bas. Y a des jours c'est la pleine forme, y a des jours on est fatigué, on a, des jours c'est pas évident, et des fois, bon ben, y a le moral bon de temps en temps il est au beau fixe, de temps en temps, souvent il est quand même au beau fixe, heureusement. Mais y a des f', mais y a des fois il tombe *(raclement de gorge)*. Moi j'ai quand même de la chance d'avoir mes trois enfants, ici à la maison, mon épouse qui me suit. Ça aide beaucoup, ça évite la solitude *(raclement de gorge)*. Vu mon, vu mon état de santé, je peux difficilement sortir, aller *(raclement de gorge)*, aller faire des soirées, tout, y a longtemps que c'est terminé. Parce que le soir c'est marqué dommage, j' mon masque à oxygène il me prend, quand même à peu près, pas loin de, 15, 18, 15 à 18 heures, je porte le masque à oxygène. Donc ça m'évite, ça me prive de toute sortie de, tout week-end, ou vacances, tout ça, y a longtemps qu'on en parle plus. Mais bon, on s'y habitue à la longue, je dirais, disons qu'y a pas le choix. Maintenant, ça fait quand même bientôt, j'ai attaqué ma septième année *(rires)*. J'ai la chance de, heureusement d'aimer beaucoup la télévision. Heureusement que j'ai ça, parce qu'en fait, autrement ben je serais là sur mon canapé, vu que je suis énormément étendu. Mes moments de libre, moments où je peux, où je suis efficace, je profite de faire de, bon ben, m'occuper à la cuisine, faire la, la vaisselle, et tout ce qui est des menus travaux, qui me changent aussi les idées. Bon j'ai la, une petite chambre, où c'est un peu en, en salle de sport, où je fais du vélo, je peux, on est obligé hein, pas se laisser complètement aller. Mais bon, ben des fois, c'est bien, mais c'est, il faut une attente. Que dire, maintenant, bon ben, on va voir l'évolution, je suis en attente sur une greffe de, de poumon, mais, faudrait encore que je perde une trentaine de kilos avant. Et pis, bon, c'est, ils sont vraiment dans un triste état. Je me pose actuellement la question si je veux vraiment le faire ou pas.

**C : Pour la greffe des poumons ?**

**T :** Ouais. Une, dernière rendez-vous que j'ai eu à l'hôpital, y a, y a trois mois en arrière. Ils m'ont dit quand même j'avais une chance sur quatre d'y rester, donc. Je veux dire, mais en fait je vis quoi, mais je me prive de, de, de certaines choses, mais bon ben je suis quand même très bien suivi. Je sais pas, c'est difficile à expliquer ce qu'on ressent. Avec ma femme, on s'est, elle était là à ce rendez-vous, on s'est regardé, y avait ces deux docteurs qui, qui m'ont expliqué en gros ce que, ce qui allait, ben *(raclement de gorge)* ça refroidit hein, je vais dire *(toux)*. Parce que même, même mes déplacements jusqu'à l'hôpital, c'est toute une aventure, toute une organisation. xxx tout de suite à l'entrée, en chaise roulante, parce que évidemment, tout est difficile hein pour moi. Maintenant je sais pas que dire de plus. Je vis mais, c'est pas le grand bonheur, mais c'est pas le grand malheur non plus. Voilà, en quelques mots, en fait.

**C : Oui. Vous disiez des *(elle lève et baisse la main)*.**

**T :** J'ai des amis, ouais y a toujours des hauts et des bas.

**C : Des hauts et des bas.**

**T :** Et pis ça dépend aussi la, la, la pression atmosphérique. Ça a l'air de rien, mais dès que le temps, changement de temps, quand ça devient *(il imite une difficulté respiratoire)*. Y a des jours je peux

même pas, je peux rien faire, je m'installe sur le canapé, je suis « out » hein. Tout d'un coup y a le soleil, alors là je revis, j'arrête pas « vroum vroum vroum » *(rires)*…

**C : Donc voilà des, des hauts et des bas, vous disiez parfois on a le moral, parfois pas. Et là, avec les infirmières, vous parliez de ça aussi ?**

**T :** Oui. Ben disons le, j'ai jamais eu le, le moral on va dire suicidaire, donc. Chaque fois j'ai, j'ai quand même une, une raison qui me, qui me rattache, ben *(rire)* on va dire à la vie quoi. C'est pas, je vais pas dire : « aller je veux en finir ». Heureusement j'ai jamais eu cet esprit-là. J'ai quand même toujours, j'aime bien que, que mes amis viennent me trouver, qu'on discute xxx. Les visites me font toujours plaisir. Tout d'un coup, ben si on a un repas au restaurant, pour un anniversaire tout, c'est toute une organisation. Pis je sais qu'au bout de, de une heure et demie, deux heures de temps, il faut, il faut me rentrer parce que je dois prendre mes inhalations, c'est aussi tous les deux, trois heures, surtout quand je suis en mouvement, il faut, il faut que je prenne une inhalation. Parce qu'autrement je, je fatigue, je fatigue, xxx dans le, et remettre le masque et « hop » me re coucher.

**C : C'est toute une organisation.**

**T :** Ouais. Même point de vue sommeil, donc là y a des heures, j'ai jamais dormi on va dire huit heures de temps, hein, c'est toujours par tranches de trois, deux, trois heures. Après je me réveille, je, je tournique. Après je remets, je me rendors *(rires)*. J'ai un, un somnifère léger le soir, qui me permet quand même de, de, pendant les heures, je veux dire, je regarde le début du film, pis je me rendors, je me réveille, y a longtemps que le film il est fini, pis c'est tout d'un coup minuit, pis je me, c'est presque automatique, je me réveille à minuit, de nouveau pour prendre une inhalation, après je fais minuit et demi, donc jusqu'à une heure, bon ben « hop », je me mets simplement sur l'oxygène comme ça. Et à partir d'une heure, ben je me recaille, *(rires)* enfin je me rendors. Et à quatre heures du matin, c'est rebelote *(rires)*, de nouveau réveillé, une inhalation. Pis après le petit-déjeuner, à six heures, toujours, ça c'est, ah les heures pour moi, c'est impératif, les heures de repas, c'est « tac, tac ». Je deviens fou autrement *(rires)*.

**C : D'avoir des horaires.**

**T :** Oui oui. C'est important, c'est mon, mon estomac qui le réclame. Pour suivre, disons que ce soit léger, pas que je mange entre les repas. Pis je mange jamais entre les repas, en fait, mais des fois c'est long quand c'est, tout d'un coup je sais que c'est une heure de l'apr', une heure, pour le dîner, c'est une heure, mais après, ça craint, donc je dois attendre une heure et demi, ou.

**C : Vous avez besoin de cette régularité.**

**T :** Et vu que mon épouse elle a des, beaucoup d'horaires de travail, donc elle travaille beaucoup de, de soirs dans les X *(type de travail) (raclement de gorge)*. Souvent je, le soir ben, j'ai à sept heures et demi c'est, j'appelle les enfants, c'est à table. Bon ben j'ai toujours ma fille, souvent qui met la table, ou. Quand ma femme *(raclement de gorge)*, quand ma femme part, autrement c'est elle qui fait. Ou souvent je le fais aussi, je mets la table, comme hier soir ben c'est moi qui leur ai fait à manger. Bon, ma femme a tout préparé, j'ai juste à réchauffer *(rires)*. Comme ça, heureusement d'ailleurs, là elle popote déjà vu que ce soir elle travaille de nouveau *(toux)*. Mais ça va, disons que ça, ça maintient bien le moral, d'être une famille très soudée. Ça, ça faut reconnaître, sans ça si je devais me retrouver tout seul dans une maison, j'aime bien les infirmiers *(rires)*, y a pas de problèmes ça fait de la compagnie, mais quand ils sont pas là, après c'est vite, on sent, on sent vite la solitude... Bon un de mes fils il a exercé, il exerce la même profession que moi, que j'ai exercé, alors encore maintenant, malgré qu'il a 30 ans, il me demande toujours conseil : « papa, tu ferais comment ? ». Ça me remet un petit peu dans la vie active hein... Ma foi quand on est atteint, on est atteint hein. Je pensais jamais un jour me retrouver à l'AI là *(rires)*.

**C : Donc le fait d'avoir une famille soudée, [ça aide pour la solitude.**

**T :** Oui, psychologiquement c'est énorme,] oui. Et même, aussi ma belle-mère, ça marche très bien. Non, c'est vrai, de l'autre côté, j'ai ma soeur qui habite dans l'immeuble, un peu plus loin, mais bon ben, je la vois peut-être une fois par année. Mais jamais on s'est engueulé, quand on se voit c'est le bonheur, mais c'est vrai on va pas se déranger pour un oui, pour un non.

**C : Vous avez votre femme, vos trois enfants qui sont là, et.**

**T :** Voilà, oui. Ma fille, elle fréquente donc déjà un peu moins maintenant, elle dort souvent chez son ami là. Ben elle a 26 ans, c'est le moment ou jamais hein *(rires)*. On attend des petits-enfants pour pouvoir égayer le tout *(rires)*. Mais ils sont pas pressés maintenant les jeunes hein, c'est dingue hein *(rires)*.

**C : *(rires)*. Donc ça, ça aide un peu pour le moral,** [**d'avoir.**
**T :** Ouais, ouais].

**C : Et, comment ça se passait lors des visites des infirmières avec vos proches, quand elles venaient une fois par mois ?**

**T :** Ben, rien de. Bon, ma femme, à force, elle connaissait X *(surnom donné à une des infirmières de recherche) (rires)*, Claire *(nom d'emprunt)*. Elle connaissait, elle était toujours un peu là, et pis bon ben, on répondait, c'était surtout le questionnaire, donc. Comment je me sentais, l'état de fatigue, l'état de moral.

**C : Pis ça, vous disiez, ces questionnaires, plus un peu regarder le moral comment ça allait, et.**

**T :** Ouais, où ça en était d'une fois à l'autre, d'un mois à l'autre. Mais bon ben ça bougeait pas, y avait pas grand-chose à redire d'un mois à l'autre, parce qu'en fait y a rien qui, y avait pas de modifications sur mon état de santé. *(toux)* Excusez-moi.

**C : Y avait pas de modifications sur votre état de santé.**

**T :** Encore maintenant si je devais répondre le questionnaire, je répondrais toujours la même chose *(rires)*.

**C : Et, peut-être qu'est-ce qui vous a plu ou déplu en particulier, dans ces visites, une fois par mois, des infirmières ?**

**T :** … Avoir une visite, on va dire *(rires) (toux)*. Voilà, pis donc, ben quand même, je vois pas ce qui aurait pu me déplaire, parce que bon ben, c'était surtout un questionnaire, et c'était pour une enquête, pour leur, pour leur business, en fait hein.

**C : Donc le fait d'avoir de la visite.**

**T :** Ça m'a jamais contrarié donc.

**C : C'est pas, pas quelque chose qui vous a contrarié.**

**T :** Si, quand elles ont arrêté *(rires)* !

**C : Vous auriez voulu que ça continue ?**

**T :** Bon, ben ma foi non, l'enquête était comme ça, on m'avait demandé si ça me, si y avait pas de problèmes, s'ils pouvaient le faire, j'ai dit : « moi y a aucun problème. Je suis pas, je suis pas un sauvage ». *(rires) (toux).*

**C : Mais ça, ça vous aurait pas dérangé que ça continue, par exemple ?**

**T :** Ben non, mais bon ben, peut-être il aurait fallu justement, après une année, maintenant qu'y ait de nouveau, qu'on puisse peut-être éventuellement voir si y a du changement, parce que c'est, d'un mois à l'autre, on voit pas de changements, mais, à moins qu'y ait vraiment le coup dur, on va dire, hein.

**C : Le fait de voir ces changements-là, voir l'évolution.**

**T :** Tout à fait.

**C : Et, qu'est-ce que vous attendiez en participant à cette étude ?**

**T :** Ben, moi, rien spécialement en fait *(rires)*. *(toux)*. C'était surtout pour ces, pour eux, pour leur enquête. Mais bon, mais moi, c'est comme un sondage, en fait. Ils demandent mon avis, ben je donne mon avis, mais bon ben *(rires)*.

**C : Oui, donc c'était plus pour l'étude, pour l'enquête, pour faire un sondage que vous avez participé.**

**T :** Voilà, tout à fait. C'est comme si vous me demandez Hillary Clinton ou l'autre pingouin, là *(rires)* je donnerais mon avis. Ça fait peur que l'autre il passe, mais enfin *(toux)*.

**C : C'est plus pour donner votre avis.**

**T :** Voilà, oui. L'exemple il est un peu bête, mais enfin *(rires)*.

**C :** ***(rires)*** **Non. Et, peut-être vous avez reçu des informations sur la BPCO, son traitement ou son évolution pendant ces visites des infirmières. Et, quelle est votre appréciation des informations que vous avez reçues ?**

**T :** … Ben… Ben, ça a permis de savoir l'évolution de, de ce que c'était en fait le, le BCPO, quoi le. Je savais quand même plus au moins *(rires)*, un peu depuis le premier jour, donc. Je sais pas, je peux pas, pas de réponse, quoi.

**C : Ouais. Vous, vous saviez déjà.**

**T :** Ouais, d'ailleurs j'ai juste parcouru comme ça, mais je me suis pas à fond dedans, hein.

**C : Oui, oui. Et, je sais que, voilà, parfois elles ont pu aussi aborder tout ce qui est soutien au niveau spirituel, pendant ces visites, par exemple. Est-ce que c'est, quelque chose que vous avez abordé, ou comment ça s'est passé ?**

**T :** Je comprends pas bien le.

**C : Parfois, je sais que, pendant ces visites, elles ont par exemple pu parler de pleins de choses différentes, de, voir quels étaient vos besoins. Et peut-être de, y a des besoins spirituels parfois chez certaines personnes, je sais pas si c'est quelque chose que vous avez discuté ?**

**T :** Ben on discutait un petit peu de, ben de, de, de ma vie, donc ce que je. J'ai dit bon ben moi, je m'occupais de, de X *(métier)*, j'étais X *(fonction)*, jusqu'à ma foi, où je puisse plus marcher donc je pouvais plus assumer ma X *(fonction)*. Et pis avec les X *(métier)*, ben du coup, chaque fois elles repartaient d'ici avec X *(produit de son métier)*. Y avait un contact <u>humain</u>, en fait, puisque j'aimais bien faire… Voilà quoi, je sais pas trop *(rires)*.

**C : Vous aviez un contact humain [avec les personnes qui venaient faire ces visites.**

**T :** Voilà]. Voilà. Beaucoup de, des activités que j'avais avant, parce que bon ben, j'ai pas, j'ai pas que fait de la X *(métier)*, j'ai fait 20 ans de X *(autre métier)*. Y avait tellement de, je m'occupais de X *(métier)*. J'ai fait partie de X *(autre activité)*. C'est vrai que j'ai, j'en ai eu des activités que, j'ai pas arrêté jusqu'à que je tombe malade, et pis bon ben, ça a été un peu le, le « out », hein. Beaucoup de gens me regrettent d'ailleurs dans X *(métier)*, parce que bon ben, j'ai quand même été 13 ans X *(fonction)*, donc ça, ça se compte, hein. xxx, ben elles sont venues, ils sont maintenant, ils étaient tout petits, huit ans, neuf ans, maintenant elles sont mères de famille, et tout, à chaque fois *(rires)*. Quand je passe dans X *(lieu)*, c'est toujours : « oh, le X *(fonction)* *(rires)*, l'ancien X *(fonction)* ». *(rires)* Evidemment que quand ça change, et pis qu'on a quelqu'un qui est pas apprécié après, la suite, ils aimeraient bien que je revienne, mais je dis : « mes pauvres, je peux pas » *(rires)*. Mais disons c'est vrai que ça me manque quand même, toutes ces activités que j'avais, toujours à gauche, à droite. Comme ma femme, elle était toujours aussi très active. Mais bon à l'époque c'était les X *(activité)*,

c'est comme ça qu'on s'est connu, moi la X *(activité)*, elle dans les X *(activité) (rires)*. C'est vieux, les années 77, 78 *(rires)*, 1900 hein *(rires)*.

**C : Quand même *(rires)*. Donc toutes ces activités vous manquent un peu.**

**T :** Au, au début, mais après bon, petit à petit, c'est vrai que le temps il passe hein. On se fait une raison en fait hein, on peut plus, on peut plus hein. Ça a pas été facile au début, c'est vrai que, quand on est très actif, parce que, tout d'un coup c'est cette rupture, on se retrouve dans les quatre murs, en fait hein. C'est pas tout le temps évid', voilà, c'était pas tout le temps évident, mais à la longue on s'y habitue. C'est vrai que la première année j'aurais pas eu la même discussion avec vous, hein. Mais bon ben six ans après, si on se fait pas une raison, on se la fait jamais, hein *(rires)*.

**C : Oui. Donc y a une part d'habituation à la situation.**

**T :** Mais les journées elles sont longues hein, des fois. Parce que c'est vrai que, bon ben, quand ma femme, elle, je vous dis, elle travaille, qu'elle est pas là, parce que des fois elle attaque X *(métier)* dès le matin. Comme hier matin, ben à huit heures, elle était déjà loin, et pis elle est rentrée qu'à minuit, hein. Donc ça fait des grosses journées. Pis mes enfants, bon ben ils sont toujours, ils sont à l'étage, je les vois, ouais, au moment du souper *(rires)*. Quand je dis : « à table ». *(forte toux)* Ouh là. Quand je dis : « à table ». Ouais, effectivement, ou autrement *(rires)*, on les voit pas beaucoup *(rires)*.

**C : Ouais *(rires)*. [Parfois des journées un peu longues, mais. Voilà, vous disiez aussi que vous organisez pour le souper avec votre femme qui vous prépare à l'avance, et parfois des coups de main aussi de votre fille, ou, des enfants.**

**T :** Ouais. Ouais.] Ben moi je remplis la machine, mais comme pour la vider, je demande à, quand même à ma fille de, de vider la machine.

**C : Ouais. Donc, et, de, de quoi d'autre auriez-vous besoin peut-être en terme de, de soutien de la part des proches ?**

**T :** Oh rien. Des fois un peu le, le, le ménage, parce que ma femme, avec toutes ses activités, y a des fois c'est la course, alors, le repassage, ça s'empile, ça s'empile, ça s'empile *(rires)*. Mais je crois qu'elle a une copine qui a proposé de venir faire quelques heures de repassage, ça lui fera quelques petits sous, bon ben. C'est pas évident, parce que sur cinq, on a chacun nos, nos, nos habits, c'est pas *(rires)*, c'est pas quand on repasse juste pour une personne, quoi *(rires)*.

**C : Ouais *(rires)*. Vous avez aussi des amis qui viennent vous rendre visite.**

**T :** Pas souvent hein, je dois dire, c'est ça qui est quand même étonnant hein, quand on est malade, les gens ils viennent pas beaucoup, hein. Mais bon ben… Quand ils viennent évidemment, moi je fatigue très vite, je remets mon masque, donc moi ça va bien une heure de temps, mais après c'est loin, ça c'est. Pis je deviens assez grinche, au bout d'un moment quand je sens que je fatigue *(rires)*.

**C : Ouais, c'est un peu la fatigue au bout d'un moment.**

**T :** Ouais, parce qu'il faut que je mette mon masque, après. Ouais, quand je commence un petit peu à me sentir moite là, que ça, c'est un manque. Encore juste avant que vous arriviez, moi à neuf heures et demi, j'avais déjà un premier rendez-vous avec l'hôpital, qui est déjà venu chez moi là. C'est le deuxième rendez-vous ce matin, neuf heures et demi, pis dix heures et demi. Pis quand elle me téléphone, je dis : « non mais, ça joue pas dix heures et demi ». Elle dit : « non mais on avait rendez-vous à neuf heures et demi ». Je dis : « non, j'ai marqué dix heures et demi ». J'avais confondu avec vous *(rires)*.

**C : *(rires)* Vous avez déjà plusieurs rendez-vous aujourd'hui.**

**T :** C'est pour la machine ce matin. Une fois tous les trois mois elle vient pour la machine.

**C : Pour l'oxygène ?**

**T :** Ouais, pis là j'ai demandé un nouveau masque, enfin bref, tout ce que j'ai besoin qui sont, qu'elle relève, tout joue bien. Donc question respiratoire, elle me prend la tension, le, ben elle fait toute la, la petite médecine, quoi, en fait. La tension, le souffle, le, le, le souffle, enfin la, le peu qu'y a dans les poumons *(rires)*.

**C : Et, aussi pendant, pendant ces visites de, de l'infirmière, comment ça s'est passé aussi pour la gestion des symptômes physiques justement, le souffle ou ?**

**T :** Ben toujours, toujours fixer les, les heures, c'est toujours les matins que j'aime bien, parce que l'après-midi, moi je suis « out », hein, donc faut pas me parler de rendez-vous. Même chez mon pneumologue hein, je prends toujours rendez-vous, c'est le matin, faut pas me parler l'après-midi, parce que.

**C : Et pendant ces visites des infirmières que vous avez eues une fois par an, comment ça s'est passé pour la gestion des symptômes physiques, que ce soit le souffle ou ?**

**T :** Ben là, maintenant, ça fait quand même plus d'une année, elle me dit : « ben c'est comme la dernière fois, comme la dernière fois ». Donc, ça se maintient, je suis à niveau, quoi… Elle dit y a pas d'amélioration, mais y a pas de dégradation.

**C : Oui. Et, voilà, donc pendant toutes ces visites des infirmières que vous avez eues une fois par an, ça s'est maintenu, c'était stable, [c'est ça ?**

**T :** Oui…]

**C : Et, peut-être qu'est-ce que, pendant ces visites une fois par an, qu'est-ce que vous auriez peut-être, souhaité recevoir en plus ou en moins ?**

**T :** Ben, rien, ce qu'il fallait. Ben, c'est mon, c'est mon pneumologue qui s'occupe hein, le reste. Je vois pas ce que je peux demander de plus, donc, je sais que j'aurais le droit s'il fallait des infirmières sur place, s'il fallait de, des trucs en plus. Mais tant que mon épouse s'occupe de moi pour ces petits trucs-là, y a pas de. Elle m'a renseigné, parce que je dois aller me faire piquer contre la grippe, mais paraît que les pharmacies, certaines pharmacies le font, ça m'évite d'aller jusqu'à l'hôpital pour une piqûre. Alors que j'ai une pharmacie juste à côté, où j'ai ma soeur qui est X *(métier)*, en plus là-bas alors *(rires)*. xxx : « tu prends la piqûre, pis tu viens me la faire à la maison ». *(rires)*.

**C : *(rires)*. Donc vous avez votre femme aussi qui s'occupe de ça. Et vous voyez pas d'autres besoins ou d'autres choses que vous aimeriez avoir ?**

**T :** Non, j'ai eu besoin à un moment d'un, d'un pédologue, d'un podologue, pas pédologue *(rires)*. Podologue, je l'ai eu quand je suis allé à X *(lieu)*, à X *(hôpital)*, l'année passée, j'ai eu un stage de trois semaines là. Là aussi ça m'avait bien, bien requinqué, question. Ils m'ont modifié le taux de la pression de la machine, y a eu pleins de choses. Et pis je me suis un peu, ben fait du sport. Donc là ça allait, parce que j'étais sur place. Y a pas ce déplacement, dire : « faudrait que je fasse de la physio à X *(hôpital)* ». Quand je vois que j'arrive devant l'hopital, faut encore que j'aille <u>tout au bout</u> du couloir pour la physio. Pis quand j'ai déjà fait ça, ma gym elle est faite hein ! Faut pas me demander de faire une heure de gym après, surtout qu'après j'ai le retour !

**C : Donc c'est les déplacements qui [sont.**

**T :** Ouais, qui sont] très, très durs pour moi, c'est lourd. Surtout quand je vais à l'hôpital X, alors là c'est encore pire, hein. Je suis toujours en souci avec le, le, possibilité d'oxygène dans mes bonbonnes, c'est qu'au bout de temps d'heures, quand j'ai plus l'oxygène, xxx. Hein, quand on arrive à l'hôpital, pis que les rendez-vous sont pas à l'heure, ouh j'adore ça, là ça me… Dès que je suis contrarié, alors je commencer à <u>suer</u>, c'est <u>horrible</u> ! J'ai de la difficulté avec la contrariété, en fait… J'ai toujours été une pers', j'ai toujours été à l'heure, une personne qui était à l'heure, je ne supporte pas les gens en retard *(rires)*. Ou alors, excusé, comme ben là vous vous êtes excusée, fallait pas juste pour 10 minutes, hein, mais. Mais quand elle commence à avoir une heure de retard. Mais là je vous attendais, j'ai, j'ai pas mis le masque, parce que le temps que je débranche tout, et tatati. Pis après il faut un laps de temps entre la, le masque et pis que je re passe à l'oxygène ou je me lève je

tombe hein, il faut je me réhabitue, pendant quelques minutes. Alors des fois tout d'un coup quand y a une heure de retard, alors effectivement, alors là, ça, je suis foutu, moi *(rires)*.

**C : Ça vous contrarie quand y a du retard.**

**T :** Ouais, surtout qu'après je suis en manque pour tout. Alors après j'essaie de, d'aller au plus vite… Comme la semaine dernière, on a été deux, deux, deux fois au restaurant dans la semaine. Vu que j'avais mes amis qui sont venus passer une semaine, les amis de X *(pays)*. Alors là aussi, c'est toute une semaine qui était chargée. Pour une fois que ma femme elle avait une semaine de congé, eux ils veulent aller partout, ils veulent visiter, comme les X *(origine) (rires)*. Alors, bon ben elle sortait avec eux, ils rentraient, ils faisaient à manger, et patati. Pis après, ben des fois au restaurant, l'anniversaire de, justement d'une des personnes. L'anniversaire de ma belle-mère la même semaine, et retourner au restaurant ! Ouh, bon dieu, j'étais content à la fin de la semaine moi *(rires)*.

**C : Ça faisait une semaine chargée.**

**T :** Oui, oui.

**C : Le, le fait de, d'avoir ces trajets aussi c'est difficile.**

**T :** Voilà, justement, c'est ça le plus dur pour moi. Pis ça me désorganise tout, après pour mes médicaments, parce que j'essaie de prendre avant, avant que ça arrive, avant. Des fois j'avance le médicament, pis après ben, du coup ça me manque, et ça me désynchronise pour le, pour la suite. Autant, après je suis tout content, parce que du coup ben ça m'a fait une sortie. Quand je vois le temps qu'il fait maintenant, ben je suis bien à la maison *(rires)*. Ça doit être aussi plus dur pour vous là *(rires)*.

**C : Ouais *(rires)*. Il fait froid, c'est vrai.**

**T :** Ça allait bien là quand il, ces derniers temps qu'il faisait beau, il faisait bon, c'était agréable. Pour finir, cette courbe xxx, elle est sorti de cette grosse chaleur. Pour moi c'est horrible quand c'est la chaleur, je supporte pas du tout. Alors, à des moments où y a tous les ventilos là-dedans *(soupir)*, j'aurais été incapable de sortir de la maison, hein, donc. Là maintenant, ça va quand même mieux.

**C : Ouais. Donc les, les grosses chaleurs et [ces changements atmosphériques, vous disiez c'est difficile.**

**T :** Ah, je support pas.] Bon, j'ai toujours été très sensible à la chaleur, j'ai jamais supporté la chaleur. En hiver, je suis toujours en t-shirt, je vois des gros pulls, des grosses vestes. Les gens ils doivent me traiter de dingue, mais moi c'est le contraire *(rires)*.

**C : *(rires)*. Et, donc si on regarde, si on revient un peu à ces visites que vous aviez eues une fois par mois. Donc en dehors des questionnaires, voilà, vous m'avez dit que vous aviez rempli, vous aviez aussi eu, vous avez pu discuter un petit peu du moral, de. De quoi d'autre avez-vous un peu pu discuter [lors des visites ?**

**T :** Ben on discutait] pas énormément, on faisait ce questionnaire, pis après ils repartaient, hein, donc. Non, y avait pas de discussions comme j'ai eu, comme j'ai avec vous aujourd'hui, donc y a pas de. Les questions, c'était le questionnaire en fait, voilà. « xxx ça en est où ; l'angoisse, paf. Votre état de santé en un mois, y a-t-il du changement ? ». Voilà, c'était vraiment des questionnaires, y en avait quatre pages, quand on avait fini, bon ben : « à dans un mois, quoi ». *(rires)*. Y avait pas de discussions autres de, du questionnaire, en fait.

**C : Vous discutiez un petit peu de votre vie, vous disiez.**

**T :** Ben voilà, comme je, comme je vous ai raconté en quelques mots. Jamais autant que j'ai discuté avec vous aujourd'hui, mais *(rires)*. Evidemment, quand elles voyaient cette grande X *(produit de son métier)* là, sur la table, ben tac on reparlait de X *(activité en lien avec la profession)*. Alors du coup, c'était presque une discussion avec ma femme, plus qu'avec moi *(rires)*.

**C : Vous dites, elles discutaient plus avec votre femme qu'avec vous ?**

**T :** Quand on parlait de X *(activité en lien avec la profession)*, oui, oui *(rires)*.

**C : Ouais *(rires)*. Vous disiez qu'elles repartaient aussi avec des X *(produits de son métier)*, et.**

**T :** Ah ouais, chaque fois ! Et la dernière fois elle dit : « mais je vais comment là ?! La X *(produit de son métier)* elle est, la X *(produit de son métier)* elles font la hauteur du salon là ». *(rires)*. Vous les connaissez ou bien ?

**C : *(rires)* Les X *(produits de son métier)* ?**

**T :** Oui, c'est des grandes X *(produits de son métier)* qui peuvent aller jusqu'à deux mètres d'hauteur.

**C : Je crois que j'en n'ai jamais vues des comme ça *(rires)*.**

**T :** *(rires)* Elles non plus, mais... Elles sont très bonnes entre parenthèses.

**C : Ouais. Donc voilà, parce que je sais qu'elles peuvent discuter de, de pleins de choses différentes parfois pendant ces visites. Aussi des soins par exemple, qu'on souhaiterait en fin de vie, y a aussi ce qu'on appelle [des directives.**

**T :** Ah ouais, j'avais dû] signer un papier là. Ouais, je sais pas, pour donner mon corps à la science, ou pour le. J'ai dit : « bon, prenez ce qui vous, s'il reste quelque chose de bon ». *(rires)*.

**C : Donc ça vous aviez ces, on appelle des directives anticipées, où on peut dire à l'avance si on se retrouve une fois dans une situation où on peut pas dire les soins qu'on aimerait, on peut écrire à l'avance ce qu'on souhaiterait comme soins. Donc ça c'est quelque chose que vous avez vu passer, [ou fait avec les infirmières ?**

**T :** Oui, oui, tout à fait].

**C : Comment ça s'est passé de faire ça ?**

**T :** Ben, rien. J'ai dit, j'ai dit : « bon ben moi toute façon ». Comme je vous dis : « prenez ce qu'il reste de, de bon chez moi ». *(rires)*… xxx débrancher s'il y a plus rien à faire hein, toute façon une fois que c'est foutu, c'est foutu hein.

**C : Vous avez pu discuter de ça avec elles et écrire ça. Et…**

**T :** Oui, moi si arrive un niveau où tout se dégrade, j'aimerais bien qu'on me débranche hein, pas me faire, d'être un cobaye quoi, en fait, ça m'intéresse pas, ça. Essayer de faire des essais à gauche et à droite. Etant donné que je sais exactement ce que j'ai, ça m'intéresse pas de... Les animaux on les fait pas souffrir, une piqûre pis on en parle plus, quoi ! Je préfère m'endormir. D'ailleurs y a quelques années, j'ai failli y passer, avec une piqûre de frelon. J'ai atteri, ben j'étais déjà, donc y a, justement au X *(lieu)*. Comme ça un frelon, paf, piqué là. Moi j'ai eu un arrêt respiratoire, quand même l'hélicoptère qui est venu me chercher, et tout le cheni... Pis là ben, voyez j'aurais pas eu de secours sur place, eh ben j'y restais. Mais souvent j'y ai pensé, j'ai dit : « ben tiens, ça aurait été une belle fin, pas le temps de ». *(rires)*.

**C : Pas de, de [pas souffrir.**

**T :** Pas le temps de souffrir,] voilà... Donc, ben c'est le copain qui vient souvent me trouver là. Il me dit quand même, s'il avait pas été là. Ben voilà, ben je serais pas en train de tirer la langue maintenant *(rires)*.

**C : Ouais. Mais ça vous, vous souhaiteriez pas souffrir.**

**T :** C'est la mort que tout le monde souhaite, hein. Donc, s'endormir, pas se réveiller, je crois que, qui n'aimerait pas une mort comme ça, franchement hein ?! Plutôt que de mourir dans beaucoup de souffrance, tout, moi j'aime autant m'endormir pis pas me réveiller.

**C : Ça vous avez pu dire justement que vous vouliez pas souffrir, et.**

**T :** Voilà. Surtout que j'ai ma, mon père qui est décédé, jeune, j'avais 15 ans, mort d'un cancer, je l'ai vu souffrir. Ma petite soeur, ben, elle à 36 ans, elle est décédée d'un cancer de la plèvre. Elle faisait plus que 40 kilos avant de partir. Vous avez vu tout ça, vous avez pas envie de, que ça se prolonge, hein... Enfin c'est les *(raclement de gorge)*, comme on dit, c'est les aléas de la vie, hein...

**C : Oui. De, de voir ses proches souffrir comme ça.**

**T :** Oui, oui. On a connu ça. J'ai toujours eu beaucoup de difficultés d'aller trouver quelqu'un à l'hôpital. Comme je dis, j'ai eu ma part, c'est… On va dire psychologiquement, c'était pas *(rires)*, j'ai, j'ai eu toujours de la peine après à aller voir, trouver quelqu'un à l'hôpital. Donc, s'ils viennent pas me trouver, je m'en fous, mais moi. *(raclement de gorge).*

**C : Ouais, vous avez pas envie [de retourner.**

**T :** J'ai donné, j'ai donné,] c'est.

**C : Psychologiquement, c'était.**

**T :** On a vu plusieurs personnes. Et mon beau-frère qui est décédé du cancer du colon, alors vous voyez, la famille va bien *(raclement de gorge)*. Et pis lui aussi il l'a pilé, parce que c'est un cancer qui est très, très douloureux. Je l'ai vu se dégrader en passant devant chez moi, parce que je vous dis ma soeur habite à, deux immeubles à côté. Un gars, un brave gars, qui fumait pas, il buvait pas. Oui, un verre comme ça, de temps en temps, mais il avait toutes les qualités, et pis voyez…

**C : Vous disiez les aléas de la vie, mais c'est, c'est difficile de voir ses proches souffrir.**

**T :** Mon beau-père qui est aussi, le père à mon épouse qui est aussi décédé d'un cancer, le cerveau, enfin la… Quand on *(raclement de gorge)*, quand on a vu tout ça, on n'a pas envie de, de, nous se faire prolonger la vie, hein, donc. Moi je dis quand c'est fini, c'est fini, on en parle plus, ça sert à rien de, de remettre en route la machine hein... Peut-être qu'on s'endurcit à la longue, aussi de...

**C : De voir les, les proches souffrir, [autour de soi.**

**T :** Tout à fait, ouais.] On se donne un avis différent, peut-être, <u>peut-être</u> hein, je sais pas.

**C : Ouais c'est psychologiquement pas facile de voir ses proches autour de soi souffrir, et.**

**T :** Oui pis comme je vous ai dit, surtout très jeune. Mon père, il est décédé j'avais que 15 ans, bon ben. Il est mort à côté de moi, donc effectivement ça, c'est un truc qui marque. Déjà ben, ben je devenais l'homme de la maison, il a fallu ben, j'avais une villa à entretenir à l'époque. Pis bon ben, ma mère elle avait pas tellement les moyens de, je voulais faire des études, ben c'était foutu, j'ai dû apprendre un métier parce que ma mère elle a pas pu m'entretenir. *(raclement de gorge).* Tout ça, on s'endurcit, donc automatiquement, mais. On s'endurcit, pis d'un autre, ben ma foi on mûrit plus vite, donc faut, on voit les choses différemment, quoi.

**C : On s'endurcit, c'est des expériences qui marquent.**

**T :** Tout à fait *(raclement de gorge)*, tout à fait ouais.

**C : Voir les choses différemment. Et, voilà peut-être, de manière générale, quelle a été pour vous l'utilité de, de ces visites de l'infirmière une fois par mois ?**

**T :** Comme je vous l'ai déjà dit tout à l'heure en fait *(raclement de gorge)*, bon ben c'était pour elles, pour un sondage, pour un truc, c'était une enquête pour elles hein, donc moi. *(rires)*. Moi, ça m'a rien rapporté de plus, hein, ni, ni en négatif ni en positif, hein donc. *(rires)*… Je sais pas quoi vous dire de plus de ce côté-là. C'était sympathique, on pouvait discuter un moment, bon ben… Ben voilà, rien de plus.

**C : Oui, plus pour l'étude.**

**T :** Voilà *(rires)*.

**C : Oui, donc si, si je résume un peu, vous avez participé comme vous participiez à un sondage, pour, [pour faire une étude.**

**T :** Tout à fait.]

**C : Et, donc vous avez reçu ces visites une fois par mois, c'était plutôt agréable, voilà, vous pouviez parler aussi un peu d'autre chose pendant, pendant ces moments-là. Mais c'était surtout ben remplir les questionnaires, c'était tout le temps les mêmes. Voilà, vous m'aviez dit, donc voilà ce serait peut-être utile pour vous de continuer maintenant pour voir la différence, c'était ça, de comparer.**

**T :** Ou *(raclement de gorge)* peut-être dans une année, xxx pas.

**C : Oui. Mais c'est, voilà, pas quelque chose qui vous a fait du plus ou du moins, ces visites de, de l'infirmière. Et, et autrement, au niveau aussi du, du, du soutien des proches, vous avez dit que voilà, vous avez, ce soutien de, de, de la famille, que vous étiez bien soudés et que ça, ça aidait aussi [au niveau de la solitude, et pour, pour le moral.**

**T :** Tout à fait.]

**C : Donc voilà, c'était, c'était un, un bon soutien et que.**

**T :** Tout à fait.

**C : Et que voilà, qu'y avait eu parfois des hauts et des bas, mais.**

**T :** Vous résumez un peu très bien notre discussion.

**C : *(rires)*. Voilà, donc.**

**T :** Vous auriez dû être psychologue d'ailleurs.

**C : Mais je suis psychologue *(rires)*.**

**T :** *(rires)*.

**C : Ça tombe bien *(rires)*. Mais voilà, en termes de besoins, vous voyez pas vraiment autre chose, que vous auriez besoin aujourd'hui, ou que vous auriez voulu en plus, par exemple, lors de ces visites des infirmières…**

**T :** Non, non *(rires)*.

**C : *(rires)*. Voilà, je sais pas si, s'il y avait quelque chose que vous aimeriez encore rajouter par rapport à, à ces visites des infirmières, ce que ça vous a apporté, pas apporté, ce que vous auriez voulu de différent, ce que vous retenez peut-être.**

**T :** Rien, en fait, parce que bon ben, je vous dis *(rires)* c'était, xxx c'était leur enquête… Plaisir de les voir, plaisir voilà, en résumé de, discuter un peu, mais c'est vrai, beaucoup c'était, quand on avait

rempli le format des, tous les, toute la discussion, ça faisait une bonne demi-heure, pis après ben je me remettais sous oxygène, quoi...

**C : Oui. Voilà, je sais pas si voilà vous aviez autre chose à, à rajouter ?**

**T :** Non.

**C : Donc voilà, ben merci en tout cas d'avoir pris le temps de partager votre expérience avec nous.**

**T :** [Mais je vous en prie !

**C : Merci d'avoir pris le temps.]**

# Participant n°12

Nom d'emprunt : François
Âge : 60 ans
Sexe : M
Groupe : Intervention

**C : Vous avez participé à une étude qui avait pour objectif de comparer le traitement habituel de votre maladie pulmonaire, la BPCO, avec une prise en charge précoce, soutenue et intégrée. Vous étiez dans le groupe bénéficiant de cette prise en charge globale spécialisée, et vous avez reçu la visite d'une infirmière, une fois par mois, pendant un an. Pouvez-vous me raconter comment cela s'est passé ?**

**F :** Oui, ça s'est passé très bien ! Les infirmières déjà, elles étaient très sympas, très sympathiques. Non, ça s'est bien passé, on a bien, bien parlé. Moi j'ai bien compris. Euh mais, dans le sens d'aide, oui, elles m'ont aidé dans le sens qu'elles ont discuté avec moi. Bon, mais par rapport à la maladie, y a pas d'aide, au niveau infirmier, parce que bon, c'est le médicament de base qu'on a, et c'est le seul qu'y a pour le moment. Et voilà, y a un certain moment, on commence à connaître mieux notre maladie. Et voilà, et c'est ça dans le principe, mais les infirmières elles, vraiment, elles ont fait, fait un bon travail, parce qu'elles ont été déjà très sympathiques, et elles connaissent bien leur métier. Voilà.

**C : Voilà, donc c'était des, des infirmières très sympathiques, [elles ont pu.**

**F :** Non c'était très sympathiques,] oui tout à fait. C'était très sympathique, et voilà, et on avait bien discuté, oui.

**C : Vous avez pu bien discuter de la BPCO ?**

**F :** Tout à fait, de la maladie, de la maladie. Voilà, et après, tout le reste que ça, l'intégration aussi, parce que pour ce type de maladie, l'intégration c'est très difficile. Parce que déjà, c'est une maladie que on est bien, mais tout d'un coup on est mal. Ça veut dire, même au niveau de, de, de jour en jour, c'est pas facile, c'est pas facile à vivre. Et c'est très angoissant, et c'est énervant. Voilà.

**C : Une maladie angoissante.**

**F :** Tout à fait, énormément.

**C : Ouais, ouais. Ça dépend un peu, ça varie.**

**F :** Voilà *(toux)*. Ça dépend comme on dort, la nuit qu'on a passée, et après le lendemain, voilà, comment on commence la, le matin, et après pour le reste de la journée. Et après si on a une maladie comme je vous ai dit, à soucier ou pas. Parce que voilà, quand on a aussi un autre type de maladie, c'est encore pire.

**C : Ouais. D'avoir d'autres troubles associés.**

**F :** Tout à fait, tout à fait.

**C : Et là, donc vous avez pu avoir des informations également sur la BPCO par les infirmières ?**

**F :** Tout à fait, oui *(toux)*. Ils m'ont, ils m'ont tout, tout appris. Dans un sens bon, tout appris non, c'est pas bien le terme. Mais elles m'ont bien expliqué qu'est-ce que je devais faire, qu'est-ce que je devais faire attention avec quoi. Voilà non, le médicament, on a discuté après au niveau du médicament, la façon comme on devait bien le prendre, et que je savais déjà mais elle a bien expliqué. Et je crois que c'est des gens que connaissent, ils savent ce qu'ils sont en train de faire.

**C : Les infirmières, elles savent ce qu'elles font ?**

**F :** Tout à fait, oui, oui, oui. Elles posent les bonnes questions. Et en plus elles ont le temps de discuter, voilà, pas que de la maladie, discuter de la vie. Voilà, et elles ont, elles ont bien pris son temps, oui.

**C : Vous avez pu discuter d'autres choses que de la maladie, [discuter.**

**F :** Voilà, tout à fait,] parce que la, la vie c'est relationné avec ce qu'on a comme santé, dans un principe. Après, y a tout le reste. Mais bon, mais là, oui on a bien, bien discuté, oui.

**C : Oui, oui. Et comme vous disiez, y a, pas d'aide au terme, en terme de médicaments, mais.**

**F :** Non. Bon, y a le médicament de base pour la BPCO, comme c'est le X *(médicament inhalateur à poudre)*, X *(médicament bronchodilatateur)*, X *(médicament inhalateur)*. C'est, le principe il est, d'aspirer, parce que y a la machine pour les aérosols, mais, voilà, tout le monde sait, et moi je le sais que le poumon c'est un, un organe que ça, y a pas de, ça régénère pas. Voilà, on pourrait se faire opérer du coeur, pour faire changer le coeur, et, et voilà, mais on sait que la maladie pulmonaire, c'est, et en plus avec un emphysème pulmonaire comme j'ai, avancé, ça veut dire, je sais très bien que, ma longueur de vie c'est, donnée par le médecin de X *(caisse d'assurance accident)*, l'année passée, c'est 65% de quatre ans. Alors, voilà, alors tout ça c'est angoissant. Bon, je dis bien 65% des cas comme moi. xxx ça veut pas dire c'est le mien. Le mien, j'ai arrêté de fumer pendant quatre ans et demi. Malheureusement, j'ai repris ça fait pas longtemps. Mais je fume pas beaucoup. Mais voilà, mais, parce que c'est l'anxiété, l'angoisse, et les années qu'on a passées avec la cigarette. Et voilà, y a le vice, certains, ce vice comme la cigarette, que ça revient, quand on est angoissé, quand on est énervé, voilà. Moi je crois que c'est une maladie que, actuellement, pour, peut-être xxx, si on arrête surtout la cigarette, mais on n'arrête pas la, cette maladie, on la stabilise peut-être dans certains années, de vie, d'espérance de vie, mais, on peut pas la soigner, pour le moment.

**C : Donc, c'est une maladie qui est, qui est angoissante, [et voilà, qu'on, on peut pas, soigner.**

**F :** Voilà. Tout à fait, non, non.] Non, on peut l'interrompre, pas interrompre, on peut le, comment dire, la rétrograder un peu, dans l'avancement de, type de maladie pulmonaire, voilà mais, l'arrêter on peut pas.

**C : Ouais. Et, par rapport justement à, au fait que ce soit une maladie angoissante, est-ce que vous avez pu parler de ça avec les infirmières aussi ?**

**F :** Oui, oui, on a parlé même, parce que comme j'ai mon psychiatre aussi. J'ai discuté pas mal. Et en plus je suis une personne que, j'ai besoin de parler déjà. Parce que, quand on, je, je me sers pas je, je me ferme pas sur moi, c'est impossible. Parce que rester à la maison, *(soupir)* ça angoisse encore plus, voilà. On doit discuter avec des, des gens. On doit prendre certaines activités, voilà, comme aller-retour, n'importe, voilà. Mais, à certains moments, parfois on n'arrive pas. Et ça veut dire, on se xxx même vers les autres, parce que, quand on prend nos Diskus, on va dans la rue, on se cache un peu pour, voilà, parce que, voilà. C'est ça, parfois la mentalité, mais bon, c'est comme ça que ça se passe, en tout cas pour moi.

**C : Oui, oui. Donc ça vous avez apprécié de pouvoir parler [de ces angoisses ?**

**F :** Ah oui, non, non.] C'était <u>très bien</u>, comme je vous ai dit c'était <u>très bien</u>, on a bien discuté, on a parlé, elles m'ont bien expliqué. Moi je vous ai parlé, parce que, de tout ce que je sentais. Mes sentiments, au niveau de la vie, au niveau de, de mon, de mon quotidien, avec, voilà. Non, on a bien discuté. Et elles m'ont bien entendu. Voilà, elles m'ont aussi bien compris, comme je les ai bien compris aussi.

**C : Oui ça, elles ont pu bien vous comprendre, et.**

**F :** Oui, tout à fait, oui.

**C : Et, et vous donner des informations.**

**F :** Oui.

**C : Est-ce que, quelles informations supplémentaires sur la maladie ou l'évolution, auriez-vous aimé avoir ?**

**F :** Oui. Mais *(raclement de gorge)*, le complémentaire sur ce type de maladie, c'est que elle s'empire,

voilà, c'est simple. Ça veut dire on doit avoir un, un niveau familière, avoir une bonne entourage, qui nous accompagne, ça c'est aussi très important. Mais, le problème d'aujourd'hui, c'est qu'il faut avoir de bons conditions de vivre, aussi, pour accompagner ce type de maladie, parce que le manque d'argent, c'est angoissant, comme c'était mon cas à l'époque, parce que j'avais personne pour me payer, sauf le X *(institution d'aide sociale)*. Et ça c'était très angoissant pour moi, parce que quand on va à X *(institution d'aide sociale)*, et quand, c'est pas la faute aussi à X *(institution d'aide sociale)*, c'est, c'est, c'est, c'est le méthode, c'est, c'est, c'est, voilà, c'est ça, qu'on nous demande tout, à certains moments, ça angoisse, parce qu'on s'expose, on expose notre vie, à quelqu'un, oui, que peut-être, on critique pas, mais elle, elle peut pas m'aider non plus. Alors voilà *(rire)*, quand on se trouve voilà, heureusement j'ai, un appartement que ça va, mais je connais des gens qui ont des appartements insalubres, et qui ont ce type de maladie, mais ils ont pas de moyens pour trouver un autre. Et c'est ça, moi c'est ça, très important, même au niveau infirmier, comme au niveau hospitalier, comme tout ça, que, qu'y a une association, et je sais qu'y a une, mais qui aide ce type de gens, aussi. Parce que y a des gens qui sont vraiment démunis, et qui se trouvent de, avec la BPCO, avec des courants d'air, parce que, bon, les appartements sont petits, sont des studios, ils sont insalubres parce que y a l'humidité, et ça c'est pas bon du tout, pour ce type de maladie, voilà. Mais y a pas mal de monde, qui, qui souffrent de ça, oui.

**C : Oui, c'est. [Donc les conditions de vie.**

**F :** Les conditions de vie,] c'est, c'est, c'est le plus, c'est très important les conditions de vie. Et voilà, et comme ça, ça nous aide aussi à se sentir un peu mieux, voilà.

**C : Oui, pis vous disiez tout ce qui est.**

**F :** Ouais, tout à fait.

**C : Voilà, aussi argent, où c'est angoissant.**

**F :** Oui, mais, mais, c'est, mais parce que, on dit que *(rires)* l'argent ça fait pas le bonheur, oui c'est vrai, mais ça aide beaucoup. Voilà, c'est vrai que la maladie et la, la santé, c'est très important, c'est, pour moi c'est le plus important. Mais l'argent, c'est important aussi.

**C : Oui, c'est important, oui, oui.**

**F :** Ben oui.

**C : Et vous disiez aussi d'avoir du soutien de l'entourage, [pour la maladie c'est important ?**

**F :** Oui, oui.] Moi ça va, non, non, j'ai un très bon soutien de mon entourage. Voilà, non, non, là y a pas de problèmes, je sais que voilà, j'ai aucun problème là-dedans. Mais bon, après c'est la maladie elle-même, voilà. Parce que, il faut voir une chose que, au niveau de l'entourage, parfois, on nous angoisse aussi, parce qu'ils nous voient angoissés, ils nous voient mal, et ça les angoisse. Moi je vous dis un exemple, pour moi, si je suis à l'hôpital, que j'ai été plusieurs fois, je veux pas, je veux que personne va me voir, je, je supporte pas. J'ai pas, j'ai pas besoin de ça, parce que comme j'ai dit, non, non, ça à, à l'hôpital. Non, je veux pas qu'ils me voient dans cet état-là. Ça, c'est mon problème personnel, j'aime pas. Et c'est pour ça, voilà que, mais à la maison, y a pas de problèmes.

**C : Ouais, vous n'aimez pas à l'hôpital, que les gens viennent vous voir.**

**F :** Non, j'aime pas, non. J'aime pas, parce que ça m'angoisse aussi, voir dans cet état-là. Non, c'est pas, non, je, je veux pas.

**C : Comme vous disiez ça vous angoisse de voir les autres [s'angoisser pour vous.**

**F :** Voilà, voilà] c'est ça, c'est ça. Ça c'est, voilà. Mais bon, c'est mon cas, alors voilà, pas pour, vis-à-vis des autres personnes, je ne sais pas. Chacun a sa façon de vivre, voilà, mais pour moi, c'est ça.

**C : Ouais, vous avez ce, ce [soutien de l'entourage.**

**F :** Non et j'ai discuté] avec ça avec les infirmières qui sont venues. On a, on a, comme j'ai dit, j'ai parlé de

la maladie, comme de la vie, parce que c'est, pour moi la vie c'est un complément de la maladie. C'est pas l'inverse. Parce que, on a une vie, après y a la maladie. Mais après la maladie elle s'installe, et voilà, et, il faut avoir la vie pour qu'on arrive à supporter cette maladie, parce qu'elle est très pénible et très difficile. Ouais.

**C : Oui. Donc c'est important de pouvoir parler de la vie aussi, pas que de la maladie.**

**F :** Oui, oui, non, il faut, déjà il faut pas, il faut essayer de s'abstraire la maladie. Il faut pas parler de la maladie, parce que les autres, ils savent pas. Parce que pour moi, personnellement, je crois que même les médecins ils savent pas les angoisses qu'on passe, et ce qu'on souffre avec cette maladie-là. Oui, ils sont médecins, et là je suis d'accord que ils savent à peu près, voilà. Mais ce qu'on sent en réalité, c'est nous, qu'on est dans ce type de maladie, qui savons. Mais aussi y a, y a des individus qui ont une façon de voir, y a d'autres que non. Ça dépend de la personne, voilà, c'est ça. Et c'est pour ça que je disais que l'entourage elle compte beaucoup, beaucoup, beaucoup. Voilà. Et la vie, et la vie elle-même, le quotidien, voilà, tout le reste.

**C : Tout le reste qui est aussi [très important.**

**F :** Oui,] tout à fait.

**C : Et les autres, que ce soit les, les médecins ou autre, comme ils ont pas la maladie, [ils peuvent pas savoir.**

**F :** Non, je, je dis pas] que, ce que je dis, c'est pas qu'ils connaissent pas la maladie, ils connaissent bien la maladie. Simplement, c'est ce qu'on sent avec ce type de maladie. Après, ce serait bien qu'ils trouvent d'autres choses, qu'ils arrivent quand même, pas à supprimer, mais, voilà, à trouver d'autres choses pour, pour ce type de maladie. Parce que ce qu'y en a, ça nous aide beaucoup, mais je crois que xxx, c'est pas suffisant. Parce qu'il faut voir que actuellement, dans le monde, et en Suisse, y a beaucoup de monde qui souffre de cette maladie. Et c'est une maladie mortelle, parce que, *(soupir)* on peut, oui on peut vivre cinq ans, dix ans avec, il faut, il faut être bien entouré, il faut bien soigner, et il faut pas faire des abus, ni au niveau d'alcool, ni au niveau de, de cigarettes. Parce que j'ai connu des gens qui avaient la bouteille d'oxygène, ils fumaient. Voilà *(soupir)*, ça je me demandais ça sert à quoi. Voilà, ça sert à rien. Alors, mais ça, ça dépend de la personne, ça dépend comment elle est, comme je dis, bon ça dépend sa façon de vivre, ouais.

**C : Ouais, ouais, ouais. Pis y a des, des médicaments qui aident, mais ce serait bien de pouvoir trouver autre chose.**

**F :** Oui. Moi, pour la cigarette, malheureusement après je suis retombé dedans. Ça fait, maintenant huit mois. Et, ce que ça m'a aidé à l'époque à arrêter, c'était plus au moins pour être hospitalisé. J'ai passé 15 jours oxygéné, et c'est là que j'ai arrêté la cigarette. Et quand je suis sorti j'ai eu tellement peur que j'ai plus fumé, j'ai plus fumé. Et je fume quand même j'ai 60 ans, et j'ai commencé à fumer à l'âge de 16 ans, au lycée. Ça veut dire c'est, j'ai jamais arrêté. Alors, jusque voilà, 2011. Euh mais bon, après je suis sorti, j'ai plus pris la cigarette jusqu'à un certain moment avec, voilà, la vie. Tout ce qui s'est passé avec l'AI *(assurance-invalidité)*, tout ça, mes angoisses, tout le reste, voilà ça. J'ai recommencé à reprendre parce que, le plus important pour la cigarette, pour moi, parce que j'ai essayé tout, le patch, tout ça, j'ai pas encore essayé l'hypnose, parce que je vais le faire, mais c'est aussi nous, parce que c'est, c'est notre volonté. Mais à un certain moment il faut voir que, qu'on se trouve avec beaucoup de problèmes, pas au niveau de santé, déjà au niveau du reste, de la vie quotidienne, parce que le grand problème, comme je dis, c'est l'argent, et l'argent il est très important. Euh voilà, après, c'est tout, c'est tout ce qui vient après, tout, ouais.

**C : Ouais, c'est ça y a, il peut y avoir des médicaments, comme pour l'arrêt du tabac, mais ça vient de [soi aussi.**

**F :** Non c'est, c'est nous-mêmes.] Pour moi, c'est nous-mêmes, on peut essayer, on peut tout essayer, j'ai essayé le patch, ça fait des années, j'ai passé à l'hôpital, y a le, pour l'alcool, pas pour l'alcool, pour la cigarette, à l'hôpital cantonal, pour l'alcool non, parce que je bois aux repas, un verre, je bois pas beaucoup. Mais la cigarette, je fumais beaucoup ! Ça veut dire que je fumais deux paquets par jour. Bon, c'était, mon service aussi à l'époque, c'était très stressant. Et j'étais obligé, voilà, j'utilisais la cigarette,

pour décompresser un peu. Alors, et après qu'est-ce que ça m'est arrivé, voilà, la polyarthrite et la BPCO. Mais faut voir aussi que, j'ai, j'ai eu un, un virus, en 2006, au X *(pays)*. C'était le, la pneumonie, une pneumocoque, voilà, j'étais pas vacciné pour. Et après, à l'hôpital encore *(rires)*, j'ai eu le Pseudomonas, ça aide pas non plus. Alors voilà c'est, c'est tout l'ensemble, c'est comme je dis, c'est tout l'ensemble, c'est tout l'ensemble, oui. Parce que par exemple, à l'hôpital, j'ai trouvé une très bonne équipe, même au niveau infirmier. Non, c'était sympa. Là, heureusement, où je suis passé, j'ai jamais eu de problèmes, je me suis bien intégré, malgré que *(rires)* hospitalisation, et même ici à la maison, quand l'infirmière est là, c'était très bien, voilà.

**C : Oui, ça allait, oui.**

**F :** Oui.

**C : Et, et peut-être, qu'est-ce que vous attentiez en participant à, à cette étude ?**

**F :** Peut-être aider les autres, qui ont pas encore, ou qui viennent de commencer à l'avoir. Parce que y a tout un début partout, pour tout. Alors là, parce que pour moi, comme j'ai dit, je connais à peu près, je pense, bien, maintenant hein, voilà, parce qu'au début je connaissais rien du tout, et j'ai, j'ai angoissé beaucoup plus. Maintenant je connais, le principe de ce type de maladie. Voilà, et, et peut-être cette étude-là, ça va aider, je sais pas, les médecins, bon, ça peut fonder pour trouver d'autres choses, je sais pas quoi, mais moi je suis un peu sceptique là-dedans personnellement. Mais peut-être ça va aider quand-même les autres, la façon xxx. Parce que, comme je le dis c'est très important, quand on a ce type de maladie, le contact, avec le service hospitalier, c'est très, très important. Parce que comme je dis, on est déjà angoissé, et si on trouve des mauvais relations, des gens qui nous entourent pas bien, excusez pour mon français un peu, mais c'est, ça vient pire. Parce que c'est très important, dans les EMS, par exemple, avoir un accompagnement. Pas qu'au, au niveau infirmier, mais aussi au niveau psychiatre, psychologue, c'est très important, parce que à certains moments, il faut que les gens parlent, et qu'on arrive à libérer les gens de la maladie. Et ça, mais ça c'est très difficile, parce que ça dépend aussi de l'âge, ça dépend ce qu'elles ont vécu, ça dépend beaucoup de choses, voilà. Mais moi, le but de cette étude, c'est ça, quand on m'a proposé l'étude, j'ai dit : « bon, oui pourquoi pas ». Alors comme ça, bon, voilà, c'est ça, ouais.

**C : Voilà, c'était pour, pour les autres, [pour les gens qui.**

**F :** Et pour moi-même aussi], parce que j'avais besoin de parler de la maladie, aussi. Voilà, mais aussi, peut-être, parce que je sais pas combien de monde était dans l'étude, combien de gens, mais ça aide. Après, analyser et tout, ça aide, ça aide ! Parce que y a des gens qui ont vécu la maladie différemment de moi, parce qu'ils bougent pas, qu'ils souffrent beaucoup. Moi je souffre aussi, c'est sûr, mais j'arrive quand même à bouger, voilà.

**C : Donc voilà, à la fois pour vous et pour les autres.**

**F :** Oui voilà, voilà, et pour la famille aussi.

**C : Ouais, c'est ça. Et vous disiez aussi que c'est important ces contacts.**

**F :** Tout à fait, parce que je, je connais des gens qui ont ce type de maladie, ils se laissent aller. Qui ont la peine de prendre un bain, par exemple, s'habiller comme il faut, ils se laissent venir, ils se laissent aller. Et après, bon après, y a tous les contacts qui sont, et même les amis, parce que les amis, c'est aussi important. Et parfois, les amis ils nous abandonnent. Et voilà, et ça c'est, des gens comme ça qui se laissent aller un peu, parce que, comme je dis, psychologiquement c'est très difficile, alors voilà, c'est pas bon. Alors, ça veut dire que c'est là que rentre l'infirmière, le psychiatre, le psychologue, pour aider ces gens-là, quand, voilà, comme ça, quand ils se laissent aller, parce que la vie, la journée, de jour en jour c'est très important, oui.

**C : Ouais, pour aider les gens à pas se laisser aller.**

**F :** Tout à fait, tout à fait. Bon, parce que, après ils s'enferment à la maison, alors ils parlent pas, ils *(soupir)* râlent tout le temps, avec tout le monde, voilà, ils s'habillent même pas, ils mangent pas. Et voilà, xxx y a quelques-uns que je connais, comme moi, ils ont commencé à fumer, fumer, à boire, à fumer, à boire, à boire, c'est encore pire ! Voilà. Et ça, faut, faut que, dans cette étude-là, ils voient avec tout ce

qu'ils m'ont, m'ont interrogé, quel est le type de gens, quels sont les gens que ça, ça risque de, d'avoir ce type de vie, et qui sont déjà dedans, voilà, ça peut aider.

**C : Pouvoir voir, quelles sont les personnes à risque pour la maladie, c'est ça ?**

**F :** Tout à fait, tout à fait, oui.

**C : Qui sont à risque de développer une BPCO, [pouvoir prévenir.**

**F :** Voilà.] Pour prévenir, parce qu'ils doivent avoir une profession, je pense, avant que, parce que c'est la maladie, c'est une maladie lente, <u>cachée</u>, parce qu'on la sent pas venir, on la sent pas venir ce type de maladie. C'est vrai oui, on commence le matin, peut-être à cracher plus, à être un petit peu plus essoufflé, mais on pense pas, cette maladie, jusqu'au jour, qu'on est à la maison, comme ça m'est arrivé à moi, à dix heures du soir, y avait plus d'oxygène, parce que la saturation elle est tombée tout d'un coup, je crois que 92, 90, c'est comme ça je suis allée à l'hôpital, bon j'avais aussi une pneumonie, et voilà. Et, mais je savais pas que j'avais pas que la BPCO, à l'époque, je ne savais pas ! La polyarthrite je savais, parce que j'avais fait aussi des examens à l'hôpital cantonal, avant la BPCO. xxx savait que j'avais déjà la polyarthrite. Et à l'époque, c'était <u>très</u> sévère, maintenant ça va, parce que, y avait pas, ils savaient pas quel traitement ils pouvaient me donner. Bon, et voilà.

**C : Oui. Donc voilà, essayer de pouvoir mieux prévoir cette maladie.**

**F :** Oui, tout à fait, il me semble que si on est fumeur, et bon, mais c'est pas facile à prévoir ce type de maladie non plus, même pour le médecin, pour voilà. Mais, quand on est fumeur, et xxx, notre médecin, ou on va à l'hôpital, pour une raison quelqu'un d'autre, il faut que les gens posent la bonne question. Voilà, pour, parce que voilà, après c'est normal, c'est une personne que, il a une infection, et qu'il fume beaucoup, qu'il boit beaucoup, alors cette personne, il faut qu'elle soit, ben parfois c'est très difficile aussi, parce qu'il faut avoir la volonté du malade déjà, parce que c'est pas la faute au médecin ni à l'infirmier, non, non pas du tout, ni aux aides-soignantes, rien du tout de ça, c'est la faute à lui-même, voilà.

**C : Avoir, avoir cette volonté.**

**F :** Tout à fait ! C'est la faute, avoir cette maladie déjà, parce que, on a fait des erreurs, on n'a pas fait une vie assez saine, et voilà, et bon, et c'est ça.

**C : Cette volonté-là.**

**F :** Oui, pas, bon, y a du monde qui ont, qui ont, qui ont eu déjà, qui ont la BPCO, et qu'ils ont jamais fumé. Mais, 80% des gens qui ont cette maladie, c'est la, c'est le tabac. Bon, mais ça peut venir, comme par exemple, même courants d'air de, voilà d'une infection pulmonaire, une pneumonie, déjà qui a par exemple, pas traité, les gens ils se laissent aller voilà, les gens ils se laissent aller, c'est un rhume ça va passer, mais parfois non, c'est une grippe, bon ça va, mais non. Et, et voilà, voyez, c'est tout l'ensemble de tous les choses. Mais c'est vrai que la cigarette, c'est 80% qui fait la cause de ce type de maladie, la polyarthrite, comme la BPCO.

**C : La cigarette joue un, joue un rôle.**

**F :** Oui, parce que je lis beaucoup même de, de l'Université du Canada, qui ont, ils ont plein de choses sur la BPCO et sur la polyarthrite, et comme je lis, et je vais sur internet, et je sais pas mal de choses. Et voilà, et là je, je, je fais, voilà, je regarde ce qu'il se passe ici en Suisse, et ce qu'il se passe à l'étranger, c'est juste ça.

**C : Vous lisez pas mal de choses sur internet.**

**F :** Non, je lis <u>beaucoup</u>, beaucoup, oui, beaucoup.

**C : Par rapport à la maladie.**

**F :** Oui, oui, parce que ça m'intéresse, parce que c'est ma, <u>ma</u> maladie *(rires)*. Je dois la supporter, je dois vivre avec, alors je veux quand même que ça passe encore un petit plus de temps, ouais.

**C : Ouais. Et, donc avec les, les infirmières, vous avez pu parler de, de pas mal de choses différentes. Je sais que parfois aussi, elles parlent des soins qu'on souhaiterait en fin de vie, par rapport [à décider.**

**F :** Oui, oui.] Là, d'ailleurs il faut que je discute un peu encore, parce que j'ai fait, j'ai, j'ai signé des choses, et que je peux changer. Je vais passer d'abord, parce que, oui, les soins palliatifs, tout ça, oui. On a, bon, à certains moments, comme je dis, on veut être là plus le temps sur terre, et on demande des choses, et, à un certain moment j'ai demandé une certaine chose, après on a changé. Non, on a discuté de tout ça, mais maintenant, à ce moment-là, j'étais dans une phase, au début, pas terrible. Maintenant, ça va bien, ça va mieux, euh ça va mieux, bon au niveau de mon quotidien. Et, y a des choses que je veux changer. xxx, par exemple, je suis X *(nationalité)*, j'avais demandé d'aller au X *(pays d'origine)*, si je meurs, aller au X *(pays d'origine)*, mais non, ça vaut pas la peine, parce que j'ai ma famille ici, malgré que j'ai la famille aussi là-bas. Non, non je, je vais pas, il faut des choses que je veux changer, voilà. Après on verra, il faut que je, on va encore discuter avec, je me rappelle jamais son nom, je l'ai vu l'autre jour lundi à l'hôpital, il faut que je passe, xxx, pour faire un petit changement de mes, voilà.

**C : Voilà vous avez pu discuter avec ces infirmières, donc, [de ça.**

**F :** Oui, oui, oui, on a discuté,] on a, j'ai signé même, ils m'ont expliqué, est-ce que je voulais faire ça, ou : « si vous voulez faire ». J'ai, y a des choses que je disais : « non ». D'autres, si je voulais être alimenté par une sonde, tout ça, on a discuté de tout ça. Mais ça dépend la situation qu'on se trouve, ça dépend, quel jour on est aussi, ça dépend comment on se sent, voilà. Et après bon, après on peut changer, aussi, voilà. Et c'est ça, oui. Non, non, mais elles m'ont bien, bien appris, bon. Déjà, je connaissais ça, parce que j'ai travaillé à X *(hôpital)* à un certain moment. Alors *(rires)*, voilà, j'ai travaillé un peu, je connais un peu le système, la X *(service de l'hôpital)* tout ça. Alors je sais comment ça se passe, bon elles savent beaucoup mieux que moi ! Mais voilà, non, non ça va, ça va, on a bien discuté.

**C : Oui.**

**F :** Oui, oui, oui.

**C : Oui. Ça vous, vous connaissez un peu, [pis vous avez pu discuter.**

**F :** Ouais, je connais, oui, oui.] Alors oui, on a bien discuté oui. Bon, à l'époque, j'avais des volontés, que peut-être maintenant je les ai pas. C'est pour ça que je veux encore passer les voir, parce qu'elle m'a dit de passer, et je l'ai appelé même ce matin. Mais on n'a pas, parce que ça passait au X (boîte vocale), bon. Mais je vais passer, je vais passer, parce que je passe souvent à l'hôpital, malheureusement pour moi, et je suis sûre que je vais la voir *(rires)*.

**C : Donc c'est quelque chose que vous allez peut-être changer ?**

**F :** Changer, oui, oui, oui, oui, changer, oui, oui, surtout ce, bon. Mais non, mais c'est, elles m'ont bien expliqué, non, on a bien discuté là-dedans ! Oui, oui, elles m'ont bien. Mais vous savez quand on parle de ça c'est toujours angoissant. *(rires)* Parce que là, dans cette maladie, l'angoisse elle est terrible. Alors, quand on parle de fin de vie, quand on parle tout ça *(soupir),* et qu'on n'a pas envie de parler, parce que on a peur déjà, voilà, c'est pas évident de parler de ça. Et bon, c'est pour ça que je veux changer, bon, parce qu'après j'ai parlé avec mon entourage aussi, j'ai discuté, ils sont venus, ils ont signé aussi. Voilà, maintenant mais, il, il faut, il faut que je change xxx.

**C : Ouais, ouais c'est angoissant de parler de ces choses-là.**

**F :** Ah c'est très angoissant, oui, parce que, regardez j'ai 60 ans maintenant, euh *(soupir)* c'était en 2010 que ça a, voilà. Alors, ça fait six ans, j'avais 54 ans. Alors *(soupir)*, c'était pas évident, très difficile, même aujourd'hui c'est pas évident. Parce que 60 ans, c'est pas quelqu'un de vieux, on est déjà au troisième âge. Mais bon, mais voilà, mais ça dépend la mentalité qu'on a, et voilà, et la vie qu'on a, voilà.

**C : Ouais du, du mental.**

**F :** Parce que là c'est sûr je me laisse pas aller, ça c'est sûr, pour le moment attention, pour le moment. Bon, ça peut venir un jour, mais non, pour le moment, non.

**C : Vous vous laissez pas aller.**

**F :** Non, non, non, non.

**C : Et, voilà, à coté de ces différentes discussions, je sais qu'elles peuvent parler aussi du, du soutien au niveau spirituel.**

**F :** Oui. Bon, euh à ce niveau-là, euh ça m'évoque, je veux pas parler, la vérité, je suis X *(religion)*, mais par contre je, depuis, on va dire 30 ans, j'ai jamais, jamais entré dans une église. Et pourtant, mes parents à l'époque, ben j'ai fait tout ce qu'il se devait faire dans la X *(religion)*, le X *(rite religieux)*, après tout le reste. Non, non, *(soupir)* moi vous savez si on doit prier quelqu'un, on peut entrer dans une église, il y a personne, c'est nous. On peut prier à la maison, j'ai pas besoin qu'un prêtre vient me voir, non, non, je veux pas. Ça c'est une chose que je veux pas. Et en plus, mon père il a étudié pour X *(métier religieux)* à l'époque, il manquait une année quand il s'est marié avec ma maman. Alors, voilà, je savais comme, et je connais, non je veux pas. Vraiment, je rentre pas dans ce type de, parce que xxx vraiment pas, non, non. Moi, c'est moi-même, si j'ai envie de prier, moi tout seul, avec quelqu'un, parce que dans l'église, bon, j'ai déjà passé tellement de phases, que non, que non, [non, non, non, non.

**C : Oui, ça c'est, quelque chose c'est vous.]**

**F :** Pour moi non, non, j'ai, j'ai pas envie, j'ai pas, non, non, non. Et j'habite à côté d'une église *(rires)*. Non, non, mais c'est, non, c'est pas ça, c'est que non. Non, non.

**C : Vous pouvez prier ici, si vous avez envie.**

**F :** Oui, oui, bien sûr, comme j'ai dit, je peux entrer dans une église, j'ai déjà entré plusieurs, quand y a personne. Non, voilà, non. Non, je veux pas.

**C : Pas xxx.**

**F :** Non.

**C : Et, et, de manière un peu générale, quelle a été pour vous l'utilité des, des visites des infirmières ?**

**F :** Comme je vous ai dit tout à l'heure, non elle était intéressante, parce que là, parce qu'on a discuté de la maladie comme j'ai dit, parce que ça, la, si elle discutait de la maladie et de la vie. Parce que ça se complète les deux. Alors voilà, c'est ce qu'on a fait. Et elles m'ont expliqué les soins qui allaient avec, que je savais déjà. Mais bon, elle m'a expliqué d'une autre façon. On a parlé de, aussi de, de fin de vie, on a parlé de tout ça. Non, non, on a parlé de tout ça, parce que à un certain moment, c'était deux différentes que elles sont venues, voilà. Non, non, on a discuté de, y avait toujours, la même façon de parler, la même façon de discuter, parce que, voilà, comme j'ai dit, mais, c'était <u>ça</u>, c'était <u>ça</u> ! Parce que vous savez, quand on parle avec quelqu'un, comme les infirmières qui vient là pour un but, on peut pas parler de tout. On parle de <u>là</u>. Alors voilà, et c'est ce qu'on a fait, voilà c'est ce qu'on a fait, on a discuté, voilà, qu'est-ce que vous voulez de plus, voilà. Non, non, voilà, on a discuté de la maladie, des soins, et, et tout le reste qui va avec, oui.

**C : Oui, discuté des soins et, et de la vie, oui.**

**F :** Oui, de la vie, oui.

**C : Et, qu'auriez-vous, peut-être souhaité recevoir en plus ou en moins pendant ces visites ?**

**F :** Non, rien *(rires)*. Pour dire la vérité, rien. Non, non, rien, parce que, ça m'a pris, ils me disent : « écoutez, est-ce qu'on peut passer, par exemple, le 5 ? » Si je me sentais pas bien, je disais : « non, passez après ». Vous voyez, pas de soucis comme ça. Alors, c'est comme j'ai dit tout à l'heure, quand je suis à l'hôpital, j'aime pas, que ma famille va me voir, j'aime pas. Alors, avec eux, c'était pareil. Je me sentais mal, j'avais pas envie de parler, de voir personne. Je disais : « non, on va changer le rendez-vous s'il vous plaît, parce que là, c'est mieux pas ». C'était ça, c'était ça ! Non, non, mais elle m'ont, on a très bien parlé, non, non, non, c'est vrai, c'est vrai, non ! Oui, oui, oui.

**C : Quand, quand on se sent moins bien on a, [vous aviez moins envie.**

**F :** Voilà, c'est ça on se ferme un peu.] On se ferme un peu. Bon, les angoisses parfois, ce type de maladie, que j'ai pas dit, c'est que, par exemple, parfois on va aux urgences, et on n'a pas besoin d'aller. Mais quand on marche, on est essoufflé, tout d'un coup, on s'essou' xxx, on a tellement peur, je vais à l'hôpital voir ma situation, comment ça se passe, ça, ça se met en nous, en tout cas à moi : « ah il faut que j'aille pour contrôler ma, si elle est toujours à 96, à 97 parce que là ». Et parfois, c'est les nerfs, c'est le système nerveux que ça fait ça. Alors voilà, et parfois on n'a vraiment rien, on n'a même pas besoin, y a d'autres, peut-être d'autres gens qui ont plus besoin que nous à ce moment-là, d'être aux urgences. Mais bon, mais c'est ça ! Vous voyez c'est, c'est compliqué, c'est très compliqué, parce que y a tout le, tout le système central de notre cerveau, et tout le reste, que ça nous fait, voilà, ça nous fait ça.

**C : Oui. C'est angoissant.**

**F :** Oui, oui.

**C : Oui, oui, comme maladie.**

**F :** Oui.

**C : Oui. Donc, peut-être, de, de quoi auriez-vous eu besoin, ou auriez-vous besoin aujourd'hui ?**

**F :** Pour le moment, rien. Parce que, j'ai fait tout seul, c'est vrai que bon, pour le poids, oui, comme j'ai la polyarthrite, y a xxx, prends mon bain, je le prends seul, pour faire à manger, je sais le faire, je le fais, personne me le fait, je peux le faire seul. Non, non, j'ai pas besoin, au moment, j'ai pas besoin, non, non. Peut-être, j'aurais besoin, le jour où j'en aurai besoin, c'est peut-être quelqu'un qui est à la maison, aider la famille, pour nous aider à nous. Parce que, quand, par exemple dans mon cas, si à un certain moment, j'avais pu prendre, à faire mes besoins, aller, prendre mon bain ou ma douche, aller préparer à manger, à me raser, à m'habiller, alors là oui, là oui, là, avoir, être oxygéné, là oui, là, on aura besoin, mais plutôt la famille, que nous. Parce que nous c'est la famille qui me doit faire tout ça, mais la famille, elle doit être aussi bien entourée. Alors, voilà, parce que, parfois la famille, elle a besoin aussi de travailler, alors elle peut pas s'occuper que de moi, y a les autres, comme j'ai deux enfants, ils doivent s'occuper de ses enfants. Alors des enfants, alors ça veut dire, que là on peut avoir besoin d'aide, mais comme je vous ai parlé, mais cette aide-là, après c'est le système, après c'est tout ce qui va avec. Et voilà, et c'est une organisation qui peut être, pour faire un complément des infirmiers, des médecins. Il doit y avoir certains services qui doivent s'en occuper, comme par exemple, X *(institution d'aide sociale)*, l'aide à domicile, ça c'est une autre chose ! Ça, mais pour le moment, je parle pas de ça, parce que pour le moment, j'ai pas besoin de ça, non ça va. Mais là oui, si on arrive à un certain moment que, ah non, non, que, qu'on est au lit, qu'on n'arrive pas à se lever tout ça, non ça, ce sera, ça sera une autre chose à voir. Mais là, c'est la question que je me pose parfois, est-ce que là, à ce moment-là, on va être bien aidé ? Parce que le système, tout le monde le connaît. Alors, et après, je veux pas dire, y a des organisations, oui, je le sais bien, mais après, ça dépend le temps que ça prend, combien, qu'est-ce qu'ils vont faire, si y a des disponibilités, voilà c'est ça, parce que c'est pour ça que je vous dis, la maladie, c'est la maladie. Après tout ce qui va, après cette maladie, et avec la maladie, c'est complètement différent. Le quotidien, tout le reste, tout le reste, tout le reste, voilà. Et c'est ça qu'il faut voir aussi, et c'est là qu'il faut que quelqu'un travaille là-dessus, aussi. Parce que, je crois que, même ici en Suisse, on est très bien, très bien au niveau social, on est très bien, vraiment. Mais, y a certaines lacunes, parce qu'il peut pas être partout, non plus. Mais là, après, bon.

**C : Donc c'est plus par rapport aux, aux autres besoins.**

**F :** Voilà, voilà, tout à fait.

**C : De la vie en général.**

**F :** Oui, oui, voilà tout à fait, oui.

**C : De soutenir [les proches, l'entourage, qui nous soutient.**

**F :** Oui, oui, oui.] Mais oui bien sûr ! Parce que, comme je dis, l'entourage elle a besoin de, elle a sa vie

aussi, notre entourage. Je me demande, je me pose une question, par exemple, des gens qui ont une certaine maladie grave, combien sont pas divorcés après ? Combien ont été pas abandonnés par la famille ? La famille, ça veut dire que les femmes, ont, ont leur famille, les enfants, après. Je parle pas des parents et des cousins. Quand je parle la famille, c'est, notre entourage à la maison. Mais combien ? Y a pas mal, hein ! Je peux vous assurer qu'y en a beaucoup ! Parce qu'on devient une charge pour la famille. Et c'est là que on va en EMS. On va à la morgue, ça c'est une autre chose. Mais voilà, mais c'est ça ! Oui.
**C : Des, des conséquences sur la famille, et.**

**F :** Mais oui. Mais y a toujours des conséquences sur la famille, toujours, toujours. Je me rappelle mes filles, quand elles me voient me faire le X *(médicament inhalateur)* ou les aérosols, elles me demandent tout le temps : « papa tu fais quoi ? Pourquoi t'as besoin de ça ? ». Voilà. Et je vous explique comment ? « J'ai besoin. C'est un médicament, voilà. ». Mais c'est tout ces questions-là. Par exemple, ma fille qui a un enfant, il va, il me dit : « papa », parce que c'est sur internet aussi. Elle me dit : « quand je vais me marier, tu vas m'accompagner à l'église, hein, tu oublies pas ! ». Je, je réfléchis pour moi : « tu as 9 ans, tu vas te marier *(rires),* jamais ». Mais voyez, c'est tout ça. A 28 ans, à 25 ans, je serais plus là, parce que j'ai fait mes comptes *(rires)*, et je sens ma santé, je dis : « non, c'est impossible ! ». Je pense être là le plus, plus de temps possible pour vous, mais voilà.

**C : Y a aussi ces choses-là, par rapport [aux enfants.**

**F :** Ah y a tout,] y a tout ! Y a tout ! Vous voyez, y a tout ! Et ça c'est important, j'ai une autre fille de 24 ans, bon elle travaille avec X *(métier)*, elle fait les cours de xxx, à X *(hôpital)*, ça dure une année, maintenant elle est à X *(ville)*. Voyez, elle me parle déjà différemment, parce qu'elle est dans le milieu. Alors elle sait bien ! Ma compagne, la mère de mes filles, elle est X *(métier du domaine des soins).* Elle me parle, elle me connaît, elle sait comme je suis. A certains moments, il faut pas parler de la maladie avec quelqu'un qui est malade, il faut l'éviter, vraiment l'éviter. Parce que on vient, on s'énerve plus, on stress beaucoup plus, voilà. Même c'est des gens qui connaissent la maladie : « ah mais tu te sens comment ? Tout est comme ça, tout ça ». Parce que vous savez une chose, euh parce qu'avec, par exemple, tous ces médicaments qu'on prend, le système cardiaque, le niveau cardiaque, ça demande beaucoup. Et là c'est *(rires)*, quand on est couché, on entend le coeur dans les oreilles, comme on dit : « tape, tape, tape » toujours à taper plus vite, parce qu'à chaque fois que je vais à l'hôpital, ils me font un électrocardiogramme, parce que mon coeur il tape tellement fort, à cause du X *(médicament inhalateur)*. Parce que le X *(médicament inhalateur)*, quand je me sens comme ça, je mets par exemple, cinq-six X *(médicament inhalateur)* en cinq minutes. Alors ça veut dire tout le système cardiaque, ouais. Mais c'est tout, tout, je veux dire que les maladies c'est un ensemble de beaucoup de choses. Et voilà, et je vais me répéter, et dans la vie aussi, ça change énormément et dans la famille ça change beaucoup. Après, c'est pas, c'est tout le reste qui vient après, au niveau d'aide, pour nous aider, quand on, voilà quand on peut plus. Et la famille, elle peut plus non plus. Voilà, parce que elle a sa vie, aussi.

**C : Toutes les, toutes les autres aides en plus, [à la fois pour soi, et pour les autres.**

**F :** Oui. Voilà, tout à fait, tout à fait.] Ça aide beaucoup aussi, si on a, oui.

**C : Voir, voir ce qu'il y a, à ce niveau-là.**

**F :** Tout à fait, oui, oui.

**C : Donc si, voilà si je résume un peu, par rapport à, à ces visites des infirmières. Donc vous les aviez trouvé sympathiques, [c'était un moment de.**

**F :** Ah oui, oui, tout à fait, non, non, oui, oui, oui !]

**C : Voilà, vous avez pu parler [à la fois de la maladie, à la fois de la vie en général.**

**F :** Tout à fait, tout à fait, oui, oui !]

**C : Vous avez aussi pris des moments pour discuter aussi des, des soins en fin de vie, [d'écrire ça avec les infirmières.**

**F :** Oui.]

**C : Donc, donc voilà par rapport à ces visites-là. Et au niveau aussi des, des proches, vous avez le soutien de vos proches, et.**

**F :** Oui, oui.

**C : Et voilà un petit peu, [par rapport à, à ça.**

**F :** Tout à fait, oui, heureusement, oui.]

**C : Oui. Donc, donc voilà, de, de manière générale, aussi, vous m’avez dit que c’était une maladie qui est, qui est assez angoissante, [donc c’est bien de pouvoir en parler, c’est des moments importants.**

**F :** Oui. Tout à fait.] Oui parce que, voilà, comme je dis, elle est angoissante, parce qu’il me manque l’air, on n’arrive pas à respirer. Et voilà, et après, on a peur, parce que quelqu’un qui connaît la maladie, qui réfléchit, on se dit, voilà, et après ça dépend l’âge de la personne, l’état qu’elle est la personne. Parce que je crois que j’ai plus peur, à 60 ans, avec quand même une BPCO 96, de que, quelqu’un de 80 ans qui a la même maladie que moi, qui est alité, qui est au lit, qui a sa, sa, sa saturation, à 94 ou 93, qui bouge pas, c’est complètement différent, y a rien à voir ! Alors, cette personne-là, c’est différent de moi dans un sens, même si on a la même maladie. Mais voilà, ça veut pas dire que une personne à 80, elle est pas active, c’est pas ça ! Mais, avec la maladie, elle peut pas. Ça dépend aussi le, les années qu’elle a ce type de maladie, mais elle peut pas, elle peut pas, je crois pas ! Pour moi, je crois que c’est impossible. Par contre, une personne 50 ans, 60 ans, s’il gagne bien sa vie, elle peut, malgré qu’elle sait que, bon, ça va arriver et que ça doit arriver, mais bon, voilà.

**C : Donc ça, ça dépend beaucoup entre les personnes.**

**F :** Tout à fait. L’âge, c’est aussi important, dans un sens, oui. Voilà, la qualité de vie, c’est très important.

**C : Qualité de vie qui change selon l’âge.**

**F :** Oui, tout à fait, selon l’âge oui, selon l’âge, parce qu’on est malade. Peut-être ça changerait pas si on serait pas malade, mais ça, ça change, oui.

**C : Ça change à plusieurs niveaux.**

**F :** Voilà.

**C : Je sais pas si y a, y a autre chose que vous aimeriez encore rajouter par rapport à, à ces visites des infirmières, ou des besoins ?**

**F :** Non, non, parce que, l’important c’est dit. Elles, elles m’ont expliqué, elles étaient très sympathiques, elles connaissaient bien, on discutait pas mal de la maladie et de la vie, parce que c’est un complément, non, non ça va, elles étaient, elles étaient bien. Oui, j’ai bien aimé, elles étaient vraiment bien, non, non. Pour moi c’est, simplement c’est le jour que je me sentais vraiment mal que je voulais pas, je voulais voir personne pour un certain moment. Ça veut dire, bon après, ça a changé, parce que par exemple si on avait un rendez-vous le matin, j’étais pas bien le matin, je proposais de venir l’après-midi mais elles avaient, elles pouvaient pas, parce qu’elles avaient d’autres, voilà, mais on faisait un autre jour, après, parce que ça, ça dure pas toute la journée, cette angoisse ça dure pas toute la journée. Ça dure un bon moment, des heures même, mais pas toute la journée, non, non.

**C : Ça change, comme vous disiez, vous pouviez changer les rendez-vous.**

**F :** Tout à fait, oui. Oui, non, elles étaient disponibles. Non, y avait, elles étaient disponibles. Bon, c’est vrai qu’elles pouvaient pas être si elles avaient d’autres choses, elles avaient d’autres malades à voir. Mais non, mais dans l’ensemble non, ça s’est bien passé, oui.

**C : Vous disiez que ça, ça change de jour en jour, y a des fois il faut pas parler de la maladie, parler d’autre chose aussi.**

**F :** Mais non, parce qu'on veut pas ! Voilà. Moi je parle pour moi, les autres gens je sais pas. Mais moi oui, je voulais pas parler parce que, *(soupir)* non. Mais bon, mais d'autres jours on peut discuter comme je suis en train de discuter avec vous, non, non y a pas de problèmes, non, non, y a pas de problèmes. Mais, ce type de maladie, faut pas trop la rappeler *(rires)*. Parce que ça va bien, faut laisser aller tranquille, voilà, jusqu'au moment que ça va mal. Voilà, mais c'est ça. Mais.

**C : Pas trop, pas trop la rappeler, oui.**

**F :** Non, non, non, non, pas trop, parce qu'on passe déjà assez, alors ça suffit *(rires)*.

**C : Oui *(rires)*.**

**F :** Non, non mais c'est pas la faute à vous ni, ni à l'autre infirmière. Non, non, non, mais c'est pour ça que je dis, le jour que je me sentais mal je voulais pas, alors, parce que voilà *(rires)*. Ça m'angoissait plus.

**C : Merci en tout cas, voilà de, ça vous angoissait plus de, d'en parler ?**

**F :** Tout à fait, tout à fait, parce que voilà. Quand je suis bien, je peux discuter, parce que c'est une maladie que je connais malheureusement, du coup je l'ai bien, à un moment je l'ai pas accepté, mais maintenant je l'ai accepté, elle est là, voilà, je dois vivre avec, le temps que je peux vivre. Voilà, mais à un moment c'était très dur, très dur, parce que on met la faute sur tout le monde. On dit : « Dieu, mais pour quelle raison, y a, les autres ils ont rien, moi ça m'a, ça a tombé sur moi, mais pour quelle raison, c'est pas, c'est pas gentil, c'est ». Voyez ? Ce qu'on pense, c'est ce type de choses qu'on pense, au début, quand on connaît pas. Mais à longueur de temps, quand on la connaît bien, qu'on a vécu déjà pas mal de choses, et l'hospitalisation aussi, alors voilà, on l'accepte. Le plus important c'est de l'accepter, voilà.

**C : Au début, vous, vous l'acceptiez pas ?**

**F :** Ah au début, c'est terrible au début, très difficile. Au début, c'est très, très difficile quand on se trouve à l'hôpital avec l'oxygène, et pour faire 200 mètres, on prend presque une heure à pousser, et qu'on a 54 ans et xxx, et j'arrive pas à marcher et qu'on a vécu une autre vie, toujours travailler, tout ça, c'est pas évident, c'est très, très, très difficile. Et après, c'est tout le reste, y a le travail qui s'en va, on perd le travail, après y a tout le niveau économique, ça change énormément. Et voilà, et quand on fait un type de vie, qu'on se dit *(soupir)*, on a tout, la santé aussi heureusement, et tout d'un coup, on n'a pas la santé, et après on n'a rien, on a beaucoup moins, c'est pas facile.

**C : C'est difficile, [mais c'est important d'accepter.**

**F :** Très, très difficile.] Mais oui, parce que c'est important d'accepter, et après, bon après, après ça vient, ça vient ce que ça vient. Voilà.

**C : Je vous remercie beaucoup d'avoir pris le temps de.**

**F :** Je vous en prie, ça m'a fait plaisir.

**C : Partager tout ça. A part si y a encore quelque chose d'autre que vous aimeriez.**

**F :** Non, non, pas du tout, non, non c'est bon *(rires)*.

**C : Rajouter. Je pense qu'on a déjà bien discuté *(rires)*.**

**F :** Non, non, non. Oui, oui, non, y a rien d'autre à discuter. J'espère que les médecins, les spécialistes savent bien le travail qu'ils sont en train de faire, et que ça donne des, des résultats. Voilà.

**C : Oui, oui. Mais merci en tout cas d'avoir accepté de participer *(rires)*.**

**F :** Je vous en prie.

**C : Merci.**

**F :** Ça m’a fait plaisir aussi.

# Participant n°13

Nom d'emprunt : Anne
Âge : 82 ans
Sexe : F
Groupe : Contrôle

**C : Donc vous avez participé à une étude qui avait pour objectif de comparer le traitement habituel de votre maladie pulmonaire, la BPCO, avec une prise en charge précoce, soutenue et intégrée. Vous avez été dans le groupe bénéficiant des soins habituels. Et pouvez-vous me dire comment ça s'est passé ?**

**A :** Bon ben j'ai eu beaucoup de péripéties avec cet appartement. Alors que je me rends compte maintenant, que je suis dans l'appartement où y a pas de problèmes, j'ai beaucoup moins de problèmes respiratoires, quoi. Avant, j'étais tout le temps sous le, avec le, le, le « puff », à faire sans arrêt des trucs, sans arrêt. Je faisais six à huit X *(bronchodilatateur)* par jour. Et ça c'est pas bon pour le coeur, parce qu'on m'a déjà fait un choc. Donc, maintenant, je pense que c'est assez linéaire. Bon, je pense pas que ça va s'arranger mais.

**C : Mais ça va mieux depuis que vous avez déménagé.**

**A :** Oui. Oui, ah oui alors ça. Pis je dors, vous comprenez. Avant je, je dormais pas ! Toutes les deux heures ou comme ça, à chaque fois qu'il faisait son truc j'étais réveillée. Fallait que je me lève, des fois à quatre heures du matin, deux heures du matin, pis que j'aille m'asseoir sur les escaliers ! Et ce qui est terrible c'est que, au lieu de penser que j'avais bien fait, on, on m'a accusé d'être cinglée ! Voyez ? De pas être normale ! Mais personne s'est jamais occupé d'écouter ce que je dis, et pis de contrôler la chose ! Parce qu'il fallait que ce soit contrôlé officiellement. Et j'ai jamais trouvé une assistante sociale qui, qui, vient quelqu'un, enfin de, de, comment dire, qui puisse avoir une parole. Parce que bon, moi je suis seule et tout, mais jusqu'à présent, je m'ennuie jamais, hein. A six heures du soir je suis crevée, mais je, j'ai l'impression que c'est le matin *(rires)*. Bon, c'est un fait que quand je suis chez moi, j'arrête pas. Et ça c'est peut-être aussi ça qui me fait cette fatigue.

**C : [Vous êtes fatiguée.**

**A :** Parce que,] j'avais été à Montana 15 jours, trois semaines, ça allait beaucoup mieux. Voyez, tous les jours j'allais marcher un peu plus, un peu plus. Et pis, pis quand je redescends, j'étais de nouveau à la fumée, c'était effroyable ! En plus la route de X *(lieu)*, par rapport aux pollutions, c'est toujours xxx les voitures, hein. Bon là ça circule jusqu'à neuf, dix heures. Mais vers onze heures comme ça, je peux ouvrir un peu les fenêtres, jusqu'à quatre, cinq heures du matin. Donc ça va. Si j'avais, j'étais moins fatiguée, que je puisse plus être mobile, quoi. Parce que je suis allée à l'inauguration du musée X *(nom de musée)*, pis j'aimerais y retourner, j'arrive pas, c'est tout près ! Et ça, c'est difficile, parce que, moi j'ai jamais vécu en communauté, mais j'ai vu qu'à l'hôpital, c'est infernal, quoi. Parce que maintenant, ils séparent pas les gens. Les gens qui sont valides de la tête, c'est avec des gens qui sont complètement malades d'Alzheimer, moi une dame elle tapait dessus à minuit pour me réveiller. Et je trouve que là c'est très mal géré. Il semble qu'ils pourraient garder une ou deux chambres pour que les personnes qui sont là, vraiment pour autre chose que la tête. Et, et l'on peut parler à personne, parce que c'est pas la faute des infirmières parce qu'elles, elles ont beaucoup de travail. Et pis, c'est tout, alors moi j'ai toujours bon contact dehors, je retrouve toujours les gens qui me sautent au cou, qui me font la bise : « qu'est-ce que tu fais ? Y a longtemps que je t'ai pas vue ». Et pis aussi donc, ça devient officiel, je suis la pire des bonnes femmes *(rires)*. Alors je comprends pas. Ouais je, j'ai rencontré l'autre jour une fille, elle me dit : « je suis X (prénom) », connais pas, « mais on a travaillé ensemble y a 25 ans ! » ; « ah bon ? » *(rires)*. Moi je me rappelais plus, mais elle se rappelait elle. Pis quand on fait des expositions, ça c'est vrai qu'y a des gens qui me connaissent, mais bon on les voit pas forcément, y a 80 personnes. Donc je suis plus connue, que moi je connais de personnes. Mais comme ça, assez, comment dire, extérieur quoi, y a pas de, j'ai perdu 17 personnes hein, en une année. Des, des, des personnes, avec qui j'habitais à X *(lieu)*, ma meilleure amie, hein. Je devais, j'allais, j'allais la retrouver tous les été deux mois en X *(lieu)*. C'était une vraie X *(nationalité)*, qui s'était installée. Et, c'était une X *(métier)* avant. Une femme extraordinaire ! Je prépare ma valise, tout là, fin juin. Je reçois un téléphone, elle était décédée le X *(date)*. Ça c'est dur, parce que c'était vraiment quelqu'un qui était bien dans la tête, qui était de bons conseils, qui était une femme de cœur *(pleurs)*. Et puis une autre là, dernièrement aussi. Mais, alors c'est difficile d'avoir

personne *(pleurs)*. Pis j'ai demandé à voir une assistante sociale pour au moins de temps en temps avoir un conseil si j'ai besoin. Mais non ! Ça fait deux ans que j'en cherche une, mais personne *(pleurs)*! Je comprends pas ! Y a des bureaux partout, et y a personne ! *(pleurs)* Notez que, où j'ai été là, où je voulais aller moi, c'était près de X *(lieu)*. Parce que c'était près de chez moi, c'était plus facile pour déménager. Et puis, bon y avait pas de place, ils m'ont envoyé ici. Mais, là j'ai rencontré, est-ce que c'est là où j'ai rencontré, une assistante sociale pas mal, et tout, mais, juste comme ça, elle m'a dit : « moi j'ai 600 dossiers, je sais plus où je nage ! » Je comprends très bien qu'elles ont du boulot. Là, y a une bibliothèque, mais bon c'est pas terrible. Quand même, parce que je lis beaucoup, j'ai toujours beaucoup lu. Parce que si je peux pas bouger, ben je lis, hein. Autrement, on s'ennuie. 'Fin voilà. Alors… toute façon, cette maladie c'est de pire en pire ou bien ?

**C : Là vous vous, vous sentez un peu seule, ces, ces derniers temps ?**

**A :** Oui, oui, parce que quand je peux pas. Oh, ils sont gentils, ici hein, ils sont très gentils. Mais bon, des fois ça, je sais pas, aller boire un café avec quelqu'un quoi, ce serait *(elle renifle)*. Parce que là, y a, y a beaucoup de personnes, bon y en a déjà deux qui sont décédées depuis que je suis là, qui sortent pas, quoi ! Et pis qui n'ont pas, moi je me rends compte que les mamans qui ont, ont les enfants pendant dix ans sont déconnectés avec la vie. Et au bout de dix ans, ben ils ont de la peine à rattraper le, le truc. Et pis, c'est toujours beaucoup de travail, donc, intellectuellement c'est assez difficile hein, à trouver quelqu'un avec qui on puisse avoir des, des contacts. Parce que j'ai voyagé, mais surt' toujours pour les civilisations anciennes. Et puis c'est rare qu'on rencontre quelqu'un qui a, surtout dans ce milieu. Evidemment qu'y a peut-être des milieux qui sont plus fortunés, donc, ils ont peut-être contact avec des gens qui ont, qu'ont pu faire des choses aussi. Alors ça aussi c'est difficile. Mais bon moi, tout ça *(montre de la main des objets dans la pièce)*, c'est tout les expositions que j'ai vu, tout ce qu'y a là-dedans. Mais j'ai encore une bibliothèque énorme, elle est à la cave, je sais pas quoi en faire. J'ai l'impression que maintenant il faut que, que je liquide tout ça. Parce que j'aimerais pas que si je meurs, on retrouve un, un bazar chez moi, c'est ça. J'aime bien être un peu clean, quoi. Ouais ça fait une drôle de, d'impression quand on a une vie assez organisée, pis qu'on peut plus organiser physiquement. Ça c'est dur. Bon, des fois je suis même obligée de rentrer en taxi hein, parce que je. Comme c'est très mal, vous avez vu comme c'est mal organisé point de vue transports ici. Ce qu'il y avait à X *(lieu)*, je sortais, le tram était devant la porte. Et là, vu les jambes que j'ai, faut que je marche jusque là-bas. J'avais trouvé une combine, je descends à X *(lieu)*, pis après je reprends l'autre qui, qui sort, qui monte sur X *(lieu)*. Parce que mon atelier est à X *(lieu)*. Donc, bon je travaille plus beaucoup, les derniers trucs c'était Noël passé. Pour des Anglais j'avais fait, ils avaient une commande. Ils sont venus chercher, et pis voilà. Là cette année, moi je pense que c'est très grave de, ça a toujours été très grave de déménager quand on est âgé. Moi j'ai, j'avais de bons amis, avec cet X *(métier)*, X *(nom de famille)*, qui avait un atelier, enfin un truc, un grand bureau. Et, avec sa femme, X *(prénom)*, c'était vraiment des gens formidables. Et pis ils ont voulu déménager dans un truc plus petit quoi, plus, parce qu'ils avaient une grande maison à X *(lieu)*. Mais ça les a tué, hein. Après, ils étaient, ils montaient le X *(mont)* à pied, tout d'un coup ils étaient tellement fatigués qu'ils pouvaient plus. Et moi je me rends compte que ça, ça m'a zigouillé hein, ça.

**C : Le, le déménagement ?**

**A :** Oui. Ce qu'il y a, c'est qu'il faudrait que je puisse aller me reposer quelque part, parce que j'ai pas eu un jour de vacances cette année, quand même. Mais je sais pas où, comment, et pis voilà. Comme y a personne pour vous… Pis le médecin je sais pas, ces médecins *(elle renifle)*. Moi je comprends pas. Alors j'ai, on m'a dit d'aller chez un angiologue. Je vais chez l'angiologue, il m'envoie chez un dermatologue, j'ai été hier. Et pis ils me font, j'ai dit : « mais enfin y a 15 jours que j'ai été chez l'angiologue, je vais pas retourner chez l'angiologue ! Il m'a dit que ça fonctionnait bien les artères ». Comme, ils ont, comment, disons que moi je mange beaucoup, je sais pas, je sais même pas comment, maintenant ils disent vegan, je sais pas ce que ça veut dire en fait, hein. Mais disons plutôt végétalienne. Donc j'ai un très bon sang, et pas du tout les artères, tout ça, ça coule bien. Donc ça vient pas de là, mais il a pas cherché la cause hein, il m'a renvoyé à la maison : « revenez dans, dans un mois ». Point, rien !

**C : Un peu, un peu difficile [avec.**

**A :** Alors c'est frustrant] ! On se dit : « mais nom d'une pipe, c'est des médecins qui ont fait des études ! On va sur la lune, incapables de faire, trouver un truc pour enlever l'eau dans les jambes ! ». Je comprends pas ! Alors il me donne un rendez-vous, l'autre fois, pour dire : « bonjour, bonsoir ». C'est pas la peine d'y aller ! Moi avant j'avais un médecin, il était tout le temps sur son ordinateur *(elle imite le bruit*

*des doigts qui tapent sur les touches de clavier)*, il parlait pas ! Pour finir, j'ai acheté mon journal, pis j'ai commencé à lire le journal, et c'est là qu'il a, qu'il a eu le choc, pis qu'il s'est rendu compte ou que j'étais là, ou qu'y avait des choses à dire. Ça a beaucoup changé l'histoire des ordinateurs. Y a plus de contacts avec les médecins. Ou bien… oui moi je trouve qu'ils gagnent facilement leur vie, hein. Je sors de l'hôpital, ils me disent d'aller voir le généraliste. Je prends un rendez-vous, j'arrive chez lui, il me dit : « j'ai, je regarde, mais j'ai pas votre dossier. Au revoir madame ! » 150 balles ! Et je, je sais pas comment ça marche, hein ! On devrait pourtant avoir confiance comme avant. Bien sûr ils sont tous morts les médecins que j'avais, hein. J'avais des bons médecins qui, qui conversaient, qui disaient les choses, qui vous expliquaient. Là rien, hein !

**C : C'est difficile avec ces différents médecins, et.**

**A :** Je me rends compte c'est tout comme ça ! Pis c'est la même chose, le pneumologue à l'hôpital. Là-bas, il me donnait des rendez-vous tous les trois mois. J'arrivais tous les trois mois. Pis je suis arrivée aussi une fois, il m'a dit : « j'ai pas votre dossier ! » Mais j'ai eu un rendez-vous depuis trois mois quand même. Bon ! Il m'ausculte « tchouk tchouk », « au revoir, bonjour madame, à la prochaine ! ». Moi je sais pas ! Est-ce que c'est, je rêve ?!

**C : Et [par rapport.**

A : Parce que] j'ai, pis alors le seul truc, vous avez mal aux pieds, vous avez mal aux coudes, vous avez mal au nez, antidépresseurs. Mais nom d'une pipe, trois mois d'étude, ça suffit pour donner des antidépresseurs ! Pas besoin de faire sept ans d'études, hein. Vous avez une crise d'asthme, antidépresseurs. Et moi je veux pas prendre ces saloperies, j'en ai jamais pris, j'ai toujours eu, tout le monde a des pépins dans la vie, et moi ça a toujours été, ce qui sauve, je trouve c'est le travail. Et chaque fois que j'ai eu des gros pépins, ben j'ai travaillé, ou j'ai pris des cours. Le soir vous êtes fatigué, vous dormez. Le lendemain, vous allez au boulot. Et ça, ça, ça vaut tous les antidépresseurs du monde, hein. Plutôt que de se pleurer sur la barbe. Déjà les, parce que j'étais très énergique. On m'appelait le TGV *(rires)*. Donc, c'est pas mon truc de rester assise sur une chaise à pleurer ! Il faut que je fasse quelque chose ! Alors voilà. C'est comme ça, je sais pas.

**C : Donc là vous avez oui, pas envie de prendre ces antidépresseurs, mais plutôt envie de pouvoir travailler, et.**

**A :** Moi je trouve c'est la meilleure chose hein ! Parce que moi je vois de amies, mais elles sont cuites ! Complètement cuites ! Y en a une elle a perdu, moi je l'ai pas reconnue en une année, tellement elle est shootée ! Et pis je crois qu'y a le 80% de la population qui est comme ça. Et plus les, les autres qui font la cocaïne, c'est aussi payé par l'état. Y a quand même quelque chose qui joue plus là ! Et, et bien sûr, c'est, ça c'était encore des amis qui étaient plus jeunes, ils sont partis dans leur maison, en, dans le X *(lieu)*, dans le sud. Donc j'ai plus de contacts. Parce qu'ils prennent aussi de l'âge, et pis je sais pas si elle conduit toujours. Elle me téléphone de temps en temps, mais, elle me dit : « amène mes, amène des sculptures, y a tous mes patients ». Parce qu'elle fait sur le dos : « tous mes clients qui voudraient te voir ». Mais j'ai dit : « mais j'ai pas de voiture. Comment veux-tu que je t'amène des choses ? ». C'est un peu incohérent… Et pis voilà, et pis bon, il faut que, il faut que je fasse le test', bon j'ai déjà fait le testament, mais, pour régler mon atelier. J'aimerais, euh comment dire, donner à la ville de X *(lieu)*, au centre de loisirs. Parce que j'ai quand même un X *(outil de travail)*, qui est comme neuf parce que je suis très soigneuse, donc c'est, c'est quand même un X *(outil de travail)* de 17'000 francs, ça pourrait rendre service au centre de loisirs. Donc je veux faire un, un papier dans ce sens que, les outils, enfin tout ce qui pourrait les intéresser, soit légué à la ville de X *(lieu)*. Pis bon je sais pas, le reste des X *(produit de son travail)*, vous les jetez à la poubelle. Je sais pas.

**C : Ça vous allez, vous allez faire prochainement ce papier.**

**A :** Oui. J'ai déjà fait les papiers, j'ai, j'ai un notaire à X *(lieu)*, pour le, l'intérieur, si jamais il reste de l'argent, comme ça. On s'est foutu de moi, parce que j'ai légué ça à la ville de X *(lieu) (rires)*. J'ai dit : « c'est normal, ils m'ont aidé ! ». Je sais pas, pour moi, s'ils m'ont aidé, et pis qu'il reste quelque chose, je rembourse un peu ! Voilà.

**C : Et, je sais aussi qu'on peut, discuter aussi des, des soins qu'on désirerait en fin de vie. On peut aussi faire des papiers qui s'appellent des, [des directives anticipées.**

**A :** Ah oui j'ai déjà, mais moi j'avais déjà dit ça,] que je voulais moi, je voulais qu'on me, qu'on me brûle et qu'on me foute les cendres n'importe où, ça a pas d'importance. Si ça fait pousser une petite marguerite, c'est déjà quelque chose. Je suis pas croyante, pour moi on est des molécules. La même chose que, que la nature. D'ailleurs ils ont trouvé qu'on descendait du poisson dernièrement. J'ai vu une émission, c'est incroyable ! Bon, la grenouille, je me dis d'accord, y a des. Mais les poissons, les ailerons ! Et c'était extraordinaire, hein ! Je sais plus ce que je regardais à la télévision, parce que, ouais toutes ces émissions comme ça, parce qu'alors entre la, la pub et pis les, les jeux à la con, ça m'énerve. Et, mais y a des choses intéressantes quand même.

**C : Donc ça, ça vous avez déjà écrit ce que vous désiriez ?**

**A :** Oui, oui, oui.

**C : Et aussi en terme des, des soins qu'on aimerait en fin de vie, [qu'est-ce qu'on aimerait.**

**A :** Quels soins ?]

**C : Si.**

**A :** Moi j'aimerais mourir d'une crise cardiaque, c'est plus vite fait, hein. Voyez, pis y a pas des tuyaux partout, parce que, moi j'ai vu déjà j'ai fait, j'ai été quelques jours à, à X *(nom d'Hôpital de réhabilitation)*, *(elle chuchote)* mon dieu, mon dieu. Moi je trouve que c'est inhumain de, de, de garder des gens qu'on sait qu'ils vont mourir dans quand même assez peu de temps, leur, leur, leur bourrer la bouche à la cuillère pour les obliger à manger. Ils se bavent dessus, et tout. Moi je, je sais pas, oui ça c'est de l'acharnement. Et j'ai appris l'autre jour une chose formidable, c'est que les gitans, ils mettent jamais leur, leur mère à l'hôpital, jamais. Ils meurent à la maison. Ça n'existe pas. Et je crois qu'y a quand même des civilisations comme ça. Mais nous, on est une civilisation, l'hôpital poubelle, quoi. Et surtout qu'ils mélangent tout le monde. Je dis pas le, le grand hôpital, on est très bien soigné hein quand on a des choses graves. Là on est très bien. Mais alors quand on passe dans les, les, je sais pas, les X *(nom d'hôpital gériatrique, de réhabilitation)*, les machins comme ça, mais c'est, on devrait mourir de, je sais pas quoi, de choc quand même, tellement c'est, c'est déplorable, quoi.

**C : Ça vous, vous désireriez pas de, d'acharnement [thérapeutique ?**

**A :** Non, non.] Alors aussitôt qu'on essaie de discuter avec ces gens, ils vous flanquent un psychiatre dans les mains. Mais moi je, j'ai remarqué que les gens qui font la psychiatrie, faut quand même qu'ils aient aussi un problème à régler, hein. Parce qu'ils font pas ce métier comme ça, moi je crois pas. D'ailleurs moi j'ai, ma belle-soeur, qui me l'avait dit. Elle m'a dit : « oh moi tu vois je me lance là-dedans, parce que j'ai un problème à régler ». Ben elle a 70 ans, elle a toujours pas réglé, hein *(rires)*. C'est une catastrophe pour tout le monde. Et, et c'est pas, pis en plus, je sais pas, dans le temps je crois qu'il fallait avoir une analyse avant d'avoir, donc elle a pas voulu faire ça. Et alors elle est psychologue pour enfants. Mais je trouve que c'est encore pire ! Psychologue pour enfants, quand on, moi je sais pas, je. Enfin dans notre vie, nous on avait pas ça. Plutôt une baffe, et pis tu, tu reviens sur terre, quoi *(rires)*. Pour ça, c'est difficile, je. D'ailleurs y en a un des psychiatres, il m'a dit : « c'est pas faux ».

**C : C'est pas, pardon ?**

**A :** C'est pas faux. Quand je lui ai dit ça. Pas tous, mais enfin je veux dire quand même, parce que, j'en ai vu là, tellement bizarres que j'avais les yeux comme ça *(elle ouvre grand les yeux)*. Donc c'est, c'est vraiment un drôle de métier ça.

**C : Psychiatre.**

**A :** Alors ils emmerdent les grand-mères tous les jours, ils font sortir les assassins. C'est, c'est vraiment incroyable, quoi ! Ça prouve qu'y a quelque chose qui va pas dans cette psychiatre. Y a des choses qui vont pas dans ce métier-là. Je, j'avais lu des bouquins sur.

**C : Et, et si on revient un petit peu [à la, à cette étude.**

**A :** A notre problème *(rires)*.]

**C : A la participation à, à cette étude. Qu'est-ce que vous attendiez en participant à cette étude ?**

**A :** Ah moi je pensais que ça vous rendait service, c'est tout ! Pour avoir une idée de comment ça se passe ! C'est dans ce sens-là, parce qu'autrement moi, qu'est-ce que vous pouvez m'apporter avec ça, ces questions ? Rien ! Parce que c'est pas médical. Enfin vous n'êtes pas médecin !
**C : Donc c'était pour, pour l'étude, pour… voilà pour, pour l'étude que vous avez participé, pour.**

**A :** Ben disons que c'était, madame *Claire (infirmière de recherche)*, je me rappelle plus son nom de famille. Elle était très, très sympathique hein. Je l'ai rencontrée quand j'étais une fois, en attente, en hospitalisation. Très, très gentille. Elle est venue vers moi un moment discuter. Et, et voilà, je vois pas, je sais pas ce qu'on peut faire de plus. Parce que ça, bon, ça va pas revenir en arrière. Et pis y a l'âge aussi, mais moi j'ai pas envie de mourir, hein *(rires)*. Je trouve que la vie elle est tellement intéressante, hein ! Tout, des fois je vais au CERN, enfin j'allais au CERN, parce que j'ai des amis qui sont physiciens là-bas, pour faire un peu, expliquer leur. Vous avez vu aujourd'hui le journal ? Ils disent qu'y a des tremblements de terre à cause de la, de, de, des trucs de particule là, au X *(lieu)*. Vous avez pas lu le journal ?

**C : Ah, non, non.**

**A :** Y a toujours eu des choses assez farfelues, hein. Mais c'est quand même, quand ils ont trouvé le boson de Higgs, ils se sont quand même rendu compte que ça confirmait la relativité d'Einstein, quoi, hein. Donc, pis ces types-là, ils ont trouvé ça on sait pas comment y a, y a des années en arrière, c'est quand même incroyable ! Et pis tout d'un coup, scientifiquement ça sort, mais eux ils ont trouvé ça où ? C'est absolument génial, je trouve !

**C : Pleins de choses intéressantes à découvrir.**

**A :** Ouais. Pis, pis Einstein il était génial. Il a vu ma bobine Einstein. Moi j'ai pas vu mais. J'ai fait du stop avec son voisin. Et pis, il habite la villa à côté. Pis il nous a filmé, pis il a dit : « quand je reviens ». Il veut voir mes films. Alors il a dit, bon la première chose, il joue du violon le matin, après il soigne ses roses. Comme quoi, la vie c'est aussi fait de simplicité… De petits bonheurs, comme ça.

**C : De petits bonheurs.**

**A :** Ouais. Ouais, je sais pas ce que, moi j'aimerais revenir architecte, ou un truc comme ça. Ça me plairait.

**C : Architecte, ouais.**

**A :** Ouais, tout ce que. Ou anthropologue, voyez ce genre de métiers où on cherche, on trouve des trucs assez intéressants, quoi. Ça, ça meuble la vie, hein. C'est pas les « feux de l'amour », les mamies *(rires)*.

**C : *(rires)*.**

**A :** Elles préféreraient pas manger que louper les « feux de l'amour », à 80 ans *(rires)*.

**C : *(rires)*.**

**A :** Elles espèrent toujours. Trouver le prince charmant. Ça existe pas. Parce qu'on est différent, l'homme et la femme. Vous savez comment j'ai compris ça ? Parce que moi je, j'aime beaucoup les courses de voiture. Et, ça me plaît, parce que je dis le bonhomme-là, il doit avoir, pour faire ça il faut vraiment être « pfiou ». Et pis, bon, j'avais les amis, et lui il était, mécanicien sur voiture. Alors moi je dis : « ah il est formidable celui-là ». Et tout. Et pis lui, lui il voyait le moteur de la voiture, et moi je voyais le bonhomme. Voyez un peu la différence ? C'est incroyable ! C'est pour ça qu'effectivement, on n'est pas toujours sur la même longueur d'ondes, hein.

**C : On porte des regards différents.**

**A :** Alors que ça fait des pétards quand, quand y a la brosse à dents qui est pas au bon endroit, des trucs assez ridicules quoi, des disputes, c'est vrai. Ça part de rien. Enfin comme ça, je me suis pas

mariée, c'est bien.

**C : Vous êtes pas mariée.**

**A :** Non, j'ai refusé, enfin, disons que j'ai eu des, deux enfants prématurés. Ils sont décédés, pis après je me suis dit, il voulait absolument se marier. J'ai dit : « si j'ai pas d'enfants, ça sert à rien, quoi ». Moi je suis pour la vie, en libre, quoi. Comme ça, on se dispute pas. D'ailleurs on s'est pas disputé, on s'est expliqué les choses. Et puis bon, ça peut arriver un commun accord, quoi. Mais, il y tenait hein, à son mariage. Moi ça m'a toujours fait peur. J'ai l'impression qu'on allait être coincé, quoi.

**C : Pas libre.**

**A :** Ouais. Ça vous bouffe la liberté. Parce qu'on peut très bien vivre ensemble sans. Cette histoire de mariage c'est un papier, ça devient un contrat, une obligation, mais ça c'est pas normal.

**C : Donc là vous êtes séparée ?**

**A :** Oui, je suis pas, je suis pas mariée. Il avait mis les bancs là-bas aux X *(lieu)*, ou je sais pas où. Et puis, j'ai pas signé le papier. J'ai dit : « écoute ». Bon si j'avais eu des enfants, pour les enfants. Mais, je pense pas que j'aurais eu une vie très marrante, hein. C'était aussi quelqu'un hein. Personne n'est parfait. Moi non plus. Voilà.

**C : Et, par rapport à, à votre BPCO, quel soutien vous avez de la part de vos proches ?**

**A :** J'ai pas de proches, j'ai plus de proches, moi. Ma famille est en X *(pays)*, mes restes de famille, du côté de ma mère. Du côté X *(pays)*, j'ai personne. Mais bon, ils sont à X *(ville)*, c'est pour ça quand j'allais en X *(région)*, je passais toujours chez ma cousine. J'en ai une qui est très proche, enfin, sa mère aussi. La soeur de ma mère là, elle nous a toujours, elle s'est toujours occupée de nous, parce qu'on, nous on a perdu ma mère à 15 ans. Donc, bon on a été dans une maison à X *(ville)*, mais, y a toujours le soutien de ma tante, avec des lettres, avec, on allait en vacances chez elle. Donc j'ai gardé beaucoup de contact avec sa fille, X *(prénom)*. On s'écrit. Mais bon c'est pas proche, quoi. Puis j'aime pas tant téléphoner, moi j'ai horreur de parler au téléphone. J'aime bien donner un rendez-vous, puis voir la personne, voyez ? Moi j'ai vraiment remarqué que les gens qui téléphonent tout le temps, c'est très répétitif. Voyez ? Voilà.

**C : Vous préférez voir les gens.**

**A :** Ben moi je, j'espère que si j'arrive au printemps, que je serai en meilleure forme, pour pouvoir un peu, bon je crois pas que je peux voyager, la dernière fois que j'ai été, heureusement au mois xxx, j'ai été dans les X *(lieu)*. Je suis allé voir le frère de ma cousine est au X *(lieu)*. Et puis après j'ai été en X *(pays)*. C'est les trois derniers voyages que j'ai fait. Mais j'ai vu que j'arrivais pas tellement à suivre les autres. A ce moment-là, j'avais beaucoup de problèmes respiratoires. Et y avait pas, j'avais pas de X *(bronchodilatateur)*, j'avais. Alors, je pouvais pas trop suivre partout les autres. Je devais m'asseoir un moment. Je me suis dit : « je suis en train de freiner le groupe c'est ». Et puis, seule, c'est difficile, il faut, faudrait plus de temps pour réunir le programme qu'on veut voir. Tandis quand c'est organisé, ben on va voir tous les sites. C'est pour ça que je regarde toujours, maintenant à la télévision y a deux, deux films policiers. Et, ça se passe en Italie. Alors on voit Rome, on voit Florence, y a des, des très belles prises de vue. C'est pour ça que je regarde *(rires)*. Et, l'autre fois c'était aussi à Malte. Malte, c'est intéressant aussi, hein. Et, et, les peintures du Caravage beaucoup, moi j'adore le Caravage. Puis voilà, mais maintenant, avec les jambes que j'ai, j'ai de la peine, hein. Et puis je sors quand même faire les courses, parce que je veux pas rester enfermée. Parce que si je peux marcher bien, je sors toujours deux fois par jour.

**C : Vous sortez deux fois par jour.**

**A :** Oui. Je faisais les courses le matin, puis après, je vais voir un truc. Ou je vais, je vais prendre un dessert l'après-midi pour m'obliger, à marcher. Mais là, je suis coincée, hein.

**C : C'est difficile pour se déplacer ?**

**A :** Oui, parce que, je dois tout le temps mettre des, des X *(paracétamol)*, autrement je marche pas tellement c'est douloureux. Quand vous avez le pied comme ça, que vous posez le pied par terre, ça écrase. Des fois ça, ça saute ici, ça me fait des blessures. Et puis les médecins font rien. C'est pas normal !

**C : C'est frustrant.**

**A :** Mais moi je suis sûre qu'ils peuvent faire quelque chose. Parce qu'il me semble qu'on avait fait, une fois à l'hôpital, comme une ponction, ou je sais pas quelque chose comme ça, pour enlever l'eau. Parce que ça aussi finalement, ça va aux poumons, cette eau, hein. Et, alors je sais pas si, je me suis dit : « peut-être qu'y a rien à faire, ils pensent que je vais mourir bientôt, comme on me dit rien ». Je sais pas ! Si ce truc va monter plus haut que les jambes, si, comme on me dit rien, je sais pas ! Parce qu'apparemment, je trouve quand même c'est un peu bizarre, hein.

**C : Et puis, et par rapport à la BPCO, comment ça se passe, par rapport à la gestion des symptômes ? La difficulté à respirer, ou la fatigue, comme vous disiez avant ?**

**A :** Déjà un, pour, pour, moi j'ai remarqué que pour cette maladie, enfin problèmes pulmonaires, il faut marcher ! Parce que si vous avez un peu d'essoufflement le matin, que vous marchez sur 500 mètres, ça, ça vous fait respirer beaucoup mieux, voyez ? Mais autrement, bon ben je suis médicalisée à peu près toujours la même chose. Là, je suis à deux X *(bronchodilatateur)* par jour, je prends un truc pour l'eau aussi, pour le. Et puis, truc pour le, le coeur, pour. Je sais même pas s'il faudrait que je prenne encore ça, j'ai été op', on m'a fait un truc y a deux ans, puis ça à l'air d'aller, voyez ? Puis c'est, c'est des médicaments qu'il faut pas prendre avec d'autres en même temps. Et puis finalement le médecin il vous, il vous, il vous remet jamais à plat les médicaments, hein : « vous prenez quoi ? » Tac, tac, tac, tac, tac. Mais il semble que de temps en temps il devrait mettre à plat, pour voir si on continue ça, si on, voyez ? Il se passe rien. Et puis j'ai la cortisone, moi j'ai réussi à baisser à 15 milligrammes. Parce que j'essaie de, baisser ça. Et puis, 15 milligrammes, c'est juste hein, si je descends plus, ça va pas. Parce que j'ai de l'asthme aussi, je sais pas si c'est en plus ou en moins. Donc, autrement je fais deux X *(bronchodilatateur)*, en ce moment ça va bien. Pas de problèmes, j'ai plus d'ennuis de voisinage. Là ça risque pas, parce que, toutes ces dames là, elles sont quand même, moi je me, je me rends compte qu'y a des dames qui ont, qui sont plus jeunes que moi je les trouve plus abîmées, hein. Surtout mentalement, quoi, y a pas, pas grand-chose à dire. Je sais pas. Comment elles le vivent, ça ? Cette espèce de vie d'intellectuelle, en fait. Mais bon, on peut pas discuter. Elles ont des enfants, des petits-enfants, puis c'est xxx qui tourne en rond. Bon, c'est aussi une bonne chose, quoi, pour elles, hein.

**C : Vous pouvez pas discuter avec elles.**

**A :** Non, non. Y a juste la dame de la bibliothèque, mais je l'ai vue très peu de temps. C'était une bibliothécaire, elle est très bien. Et puis un vieux monsieur qui était au X *(pays)*, mais il est pas toujours bien. Je discutais avec le jardinier qui est assez marrant, enfin un monsieur qui était jardinier. Et puis il s'occupe des plantations là, dans les arbres, et tout. Il est très drôle, alors on discute des graines, parce que, moi ça m'intéresse, dans le sens que, quand on me dit on peut pas, alors je, je, j'y vais, quoi. Quand j'étais aux X *(lieu)*, j'avais pris des graines de dragonnier, ça, ça, ils viennent de X *(pays)*. Mais, et puis ils m'ont dit : « jamais vous ferez pousser ça à X *(ville)*, hein ». Mais il est comme ça *(elle montre la taille avec ses mains)*. Je l'ai donné, parce que je peux pas le mettre ici. Il a poussé ! Je ramasse des trucs. C'est tellement intéressant aussi ces développements, enfin c'est comme nous, hein, le foetus hein, ça, ça se développe, ça, ça multiplie, ça. Non, la nature c'est génial, hein. On est sur une très jolie planète. C'est une merveille, cette boule bleue. Bon, je veux pas vous enquiquiner plus longtemps.

**C : Et peut-être par rapport, voilà, parce que, y avait eu, cette séparation en deux où vous aviez eu les soins habituels. Et, quelle a été votre réaction en apprenant que vous faisiez partie du groupe qui avait les soins habituels, et pas cette prise en charge globale, spécialisée ?**

**A :** Qu'est-ce que vous appelez prise en charge ?

**C : Parce qu'en fait y avait, quand vous avez participé à l'étude, y avait la moitié des gens, comme vous, qui avaient le traitement habituel, vous avez continué avec votre traitement**

**habituel. Et l'autre moitié, qui avait une prise en charge spécialisée, avec une infirmière, qui venait une fois par mois, pendant un an.**

**A :** Ah oui ça je veux pas alors, j'ai jamais voulu ça.

**C : Ouais. Ça, vous aviez pas voulu ?**

**A :** Non, parce que j'ai fait, je suis bien dans ma tête, je fais mes trucs très régulièrement, je prendrais pas un truc qui est, qu'il ne faut pas, quoi. Si jamais je demande au médecin. Et je demande toujours au médecin ce que c'est, à quoi ça sert. Et je, je prépare tous les soirs, enfin y a pas grand-chose, hein. Y a un X *(médicament antiarythmique)*, un machin pour l'eau, et pis un peu de cortisone. Alors là c'est des X *(bronchodilatateur)*, et des trucs. Maintenant, c'est assez linéaire, c'est pas. Ben disons si un jour j'ai besoin d'aide, je demanderai de l'aide, hein. Mais, pour le moment non, quoi. Parce que… *(soupir)* moi j'estime que, je suis encore bien pour prendre des décisions, je veux pas qu'on les prenne pour moi. Au moins en discuter. Y a longtemps qu'ils me cassent les pieds avec l'infirmière. Et moi je me dis, finalement, on a l'impression qu'ils font de plus en plus pour faire de l'argent, pour, pour, parce qu'ils sont, très insistants. Pour ça je veux plus X *(institution d'aide et soins à domicile)*, parce qu'X *(institution d'aide et soins à domicile)* ils m'ont fait deux, trois coups tordus *(toux)*. Et puis, voyez, parce qu'elle m'a compté des, trois jours l'infirmière, sont jamais venus. Puis elle m'a dit : « je vous le facture ». Elle m'a envoyé une lettre en me disant qu'elle me facturait quand même à l'assurance. Donc elle a facturé ça, j'ai jamais vu les infirmières. Et puis aussi, téléphoné comme quoi je m'absente, ils ont jamais enregistré. Alors ils ont commencé, là j'avais donné les clefs, que j'ai reprises. Ils ont commencé à venir tourner dans mon, dans mon appartement, ils m'ont fait des billets partout. Et, quand je suis revenue l'année d'après, j'ai envoyé une lettre recommandée, en leur disant que je m'en vais : « je vous téléphone quand je rentre ». La même chose ! Alors j'ai dit : « maintenant je veux plus rien avoir affaire à vous, parce que c'est malhonnête, ça ». C'est vraiment l'impression qu'ils poussent, ils poussent, ils poussent, à la consultation. Quand on en a besoin oui, mais. Et là, moi si jamais je prends des infirmières, je vais prendre des infirmières à X *(lieu)*, elles sont très bien, elles vous cassent pas les pieds. Voyez ? Parce que là, y a eu une dame africaine, je dis pas au point de vue soins, parce que j'ai souvent des plaies aux jambes à cause de cette fragilité de peau. Et, mais elle a commencé à me faire un cinéma, il fallait que je, je, je tire des lumières blanches sur mon voisin, des trucs un peu là de, comme ils font chez eux là, « gling gling gling ». Et je, ça, ça m'a pas plu du tout, parce que j'ai dit je, ça n'a rien à voir avec moi, puis moi je crois pas aux imbécilités pareilles. Alors y a, c'est quand même tangent, y a de plus en plus de gens qui sont un peu comme ça, farfelus, hein. Donc.

**C : Donc ça, vous souhaitiez pas avoir des visites des infirmières à domicile, c'est quelque chose que.**

**A :** Si j'en ai besoin, oui ! Pour ça là, y'a X *(société d'hospitalisation à domicile)* à X *(lieu)* et, si jamais ben je, je demanderai pour avoir une infirmière là. Parce que j'aurai quand même, ce que j'aurais voulu c'est qu'il y ait quand même quelqu'un qui vienne une fois par semaine, un peu voir si je suis toujours là. Mais comme maintenant je suis ici, on est, on est entouré. Mais quand j'étais chez moi là-bas, je me disais : « si un jour on me retrouve trois semaines après ». Je voulais pas des bracelets, machin. Tandis que là c'est bien, parce qu'on, on, elle me voit tous les matins, c'est très, c'est très surveillé. Si elle me voit pas de la journée, elle vient.

**C : Une infirmière, ou ?**

**A :** La, la directrice. C'est elle qui a dû vous ouvrir la porte, je sais pas comment ça s'est passé ?

**C : C'est possible que ce soit elle.**

**A :** Comment vous avez fait pour sonner chez moi ?

**C : *(rires)* J'avais votre nom sur la, sur la sonnette en bas. On peut vous appeler, voilà.**

**A :** Ah je savais pas ça. Y a une sonnette en bas ?

**C : Y a, oui, y a la, la liste des personnes qui habitent ici.**

**A :** Un tableau ?

**C : Oui. Et c'est un petit, un petit appareil, puis on peut trouver votre nom. Donc je me suis permise de sonner là. Et quelqu'un est arrivé en même temps, donc qui a pu m'ouvrir, qui rentrait dedans. Donc j'ai profité.**

**A :** Ah d'accord !

**C : Voilà.**

**A :** Parce que je me disais, parce que cet apr', je l'ai vu à dîner. Mais en principe le bureau, elle est souvent dans les étages. Puis là il manque une personne. Cette semaine, ils sont très pris, je vais pas les enquiquiner.

**C : Donc vous êtes bien entourée, ici ?**

**A :** Oui ! Oui, oui. Puis ils sont venus me mettre tous les tableaux, c'est eux qui m'ont mis, enfin pas tous, parce que j'en ai tellement que *(rires)*. C'est fou ce qu'on accumule dans une vie…

**C : Et si on regarde.**

**A :** Mais c'est tout des choses avec les gens, avec les, les, contacts.

**C : Et, peut-être de, de, de quoi auriez-vous besoin aujourd'hui ?**

**A :** *(soupir)*… Oui, y a l'histoire de, de mes jambes, il faut que je trouve une solution, parce que la solution de pas sortir, ça va pas ! Voyez comment ? Y a des jours, par exemple aujourd'hui j'aurais pas pu sortir, je me suis fait mal au dos, c'est imbécile, en déménageant, donc j'ai très mal au dos. Et puis, c'est comme si je marche sur des, des bouées de caoutchouc, voyez c'est difficile. Et puis moi j'ai un peu honte, parce que normalement je suis speed, j'ai rien aux jambes, rien physiquement, voyez ? Donc, et puis, des fois, je devais faire mes courses, j'essayais toujours de combiner les trucs, et. Mais quand j'arrive à X *(lieu)*, j'ai plus le courage de prendre le 15, puis de refaire le truc ici. Donc finalement je prends un taxi, et puis je vais quand même pas prendre un taxi tous les jours, hein. Alors je sais pas.

**C : Ce serait, ce serait plutôt pour, pour vos jambes, mais pas par rapport à votre BPCO ?**

**A :** Oui, c'est à cause des jambes, c'est ça ! Enfin les, d'avoir les, les pieds enflés. Parce que c'est comme si j'avais des chaussettes jusque-là *(elle montre ses jambes)*. C'est incroyable, hein, on a l'impression d'avoir des chaussettes qui serrent jusque-là ! Et puis alors bon ben, quand vous, vous savez de la peau, ça tire, hein. Je sais pas, je vais voir, mais si ça continue comme ça qu'ils trouvent, qu'ils font rien, faudra que je, enfin que je trouve quelqu'un qui fasse mes courses ! Mais alors si je sors plus, c'est quoi ? C'est un enfermement hein, c'est pas, et puis voilà. Moi j'aime, moi j'aime, une première chose le matin c'est de, d'aller boire un café, prétexte pour lire tous les journaux, parce que j'aime bien être au courant de ce qu'il se passe dans la vie. Et puis, voilà, après je vais faire les courses. Mais c'est, en tout cas profitez de votre jeunesse. C'est horrible d'être vieux. Moi je trouve que, *(soupir)*, c'est mieux de travailler hein, que d'être vieux. Même si des fois c'est dur.

**C : Vous disiez que l'activité professionnelle, c'est important.**

**A :** Voilà, l'activité professionnelle y a le contact, y a, on a l'impression de servir à quelque chose. Et puis, c'est chouette quoi, je sais pas, la vie c'est chouette, quoi ! Y a, y a toute la nature, y a… Mais j'espère, moi je sais pas, là je trouve que si je peux pas sortir. Il va falloir que je me secoue. Mais trouver où un médecin qui s'occupe de ça ? Personne s'occupe de ça. Puis on me dit pas ce que c'est, ils doivent quand même savoir, je suis pas la seule ! Alors c'est aussi quelque part, pourquoi on me dit pas ? Voyez ? Je me dis ça, parce que c'est, pourquoi on me dit pas ? Moi je préfère qu'on me dise les choses en pleine figure, au moins je suis au courant. Mais là, rien, je me la coule complet, quoi. Enfin bon, je vais pas vous retenir beaucoup plus longtemps quand même. Je vous ai même pas offert une tasse de thé.

**C : Pas de soucis, y a vraiment pas de soucis.**

**A :** Ça va ?

**C : Oui, oui, vous inquiétez pas. J'ai bu un café avant de venir, [donc, tout va bien.**

**A :** Ah bon.]

**C : Mais voilà, si on, si on reprend un peu par rapport à, à cette étude à laquelle vous avez participé. Vous m'avez dit que vous aviez participé ben, pour l'étude, [en gros.**

**A :** Oui.]

**C : Et que, vous auriez pas voulu être dans le groupe qui avait eu les infirmières qui venaient une fois par mois, [pendant un an.**

**A :** Ah non, non, non.]

**C : Voilà. Vous avez eu les soins habituels, [pour.**

**A :** Ah parce que l'autre groupe,] c'est des gens qui sont assistés ?

**C : L'autre groupe, pendant un an, ils ont eu une infirmière [qui est venue une fois par mois.**

**A :** Ah d'accord, ah d'accord.]

**C : Pour voir les besoins en terme de gestion des symptômes physiques, [psychologiques peut-être.**

**A :** Si on se débrouille.]

**C : Voir comment on peut soulager [des douleurs.**

**A :** Oui.]

**C : Ou donner des informations, [aussi sur la BPCO.**

**A :** Ah d'accord.]

**C : Donc, voilà. Et, ça c'est quelque chose, ce type d'intervention-là, vous me disiez que c'est quelque chose que vous aviez pas besoin ?**

**A :** Oui, c'est ce qu'a décidé, enfin je sais pas, c'est sorti des enveloppes, hein. Non, point de vue là, je me débrouille.

**C : Et, quelle serait peut-être, pour vous l'utilité d'une prise en charge comme ça, une fois par mois, avec des infirmières qui sont là pour donner des informations sur la BPCO, voir la prise en charge des symptômes physiques ?**

**A :** Elles viennent d'où ces infirmières ?

**C : Là, c'était des infirmières que vous avez aussi vues pour les questionnaires que vous avez dû répondre.**

**A :** Ah oui d'accord.

**C : Voilà.**

**A :** Elles vont chez les gens ces dames ?

**C : Alors y a eu, voilà, maintenant ça s'est terminé cette partie de l'étude.**

**A :** Ah d'accord.

**C : Voilà. Mais elles ont été pendant un an, et, l'idée c'était de voir si ça ce serait utile pour les personnes qui ont une maladie pulmonaire comme vous, voir si, cette prise en charge une fois par mois, [est-ce que c'est quelque chose qui serait bénéfique.**

**A :** D'avoir de l'aide, oui.]

**C : Donc voilà si, qu'est-ce que vous en pensez, par rapport à ?**

**A :** Moi je pense que c'est pas si mal. Parce qu'y a au moins un intervenant. Mais, ça dépend ce que ça représente. Parce qu'elles viennent une fois par mois pour quoi faire ? Si c'est pour avoir un peu un entretien, d'accord, un peu une mise au point. Mais pour, pour me prendre en charge, et me faire les médicaments, ça je veux pas… Alors je sais pas.

**C : Mais si c'était pour un entretien ?**

**A :** Oui, si c'est pour un entretien, disons, pour voir, effectivement comment on se porte une fois par mois, ça je trouve que c'est bien… C'est ce que j'avais voulu, ce que j'aurais voulu avoir là-bas, j'en avais discuté, oui, comme je vous dis, parce que j'aurais pas voulu qu'on me retrouve en marmelade. Mais, apparemment ça marchait pas. C'était pas une fois par mois, c'était par semaine, c'était, c'était trop, *(elle fait un geste de rotation avec la main et un bruitage)* la main mise, voyez dessus ? Et, non mais je pense que, autrement c'est bien, d'avoir une personne qui contrôle un peu comment vous allez plus au moins, une fois par mois, je trouve que c'est même assez sympa, quoi. Oui ! Ça, ça.

**C : C'est quelque chose que vous auriez trouvé sympa ?**

**A :** Oui, oui, tout à fait. C'est ce que j'ai cherché un temps, mais j'ai pas trouvé. Enfin, en passant, peut-être aussi une assistante sociale, ou. Quand je dois faire des démarches, moi je vaux rien du tout, pour les trucs de bureau. Je vous fais une X *(création de son métier)* quand vous voulez, mais au point de vue bureau, c'est pas que j'y comprends rien, mais ça m'embête. Et c'est marrant quand quelque chose vous embête, on n'a pas envie, hein. Voyez, là il a fallu faire quand même beaucoup de papiers, mais je pense maintenant je suis assez à l'abri de ces papiers. Faut que je fasse de l'ordre et que je liquide tous mes trucs là.

**C : Donc ce serait utile pour vous d'avoir une, une assistante sociale pour les papiers ?**

**A :** Oui, voilà ! Ou, oui, et pis demander un conseil quand je suis un petit peu, quand je dois faire des démarches. Mais je crois que là maintenant, c'est assez pris en charge, enfin je sais pas. Mais, si je dois faire des démarches, là il faut encore que je regarde si, les démarches, à X *(office cantonal des assurances sociales)*. Parce que X *(office cantonal des assurances sociales)*, devait me donner une subvention pour l'appareil *(auditif)*. Cette année, ça a été vraiment la catastrophe, la, la dame. Parce que là, elle m'a mis un appareil de, provisoire. Et puis tout d'un coup j'ai été à l'hôpital, elle m'a harcelé comme une dingue pour que je lui paie les 2000 balles de l'appareil. Et puis moi je comprenais pas, enfin j'ai compris après c'est que, elle a fermé son magasin, elle est partie. Je me suis retrouvée avec un appareil pas réglé, rien du tout. Enfin, heureusement c'est X *(entreprise d'appareils auditifs)*, un magasin là en ville, qui a repris, parce que je suis pas la seule qu'elle a planté. Et puis après, j'ai eu une combine, c'est que tout d'un coup j'avais plus d'assurance. On s'est demandé, j'ai recherché, enfin en cherchant il s'est trouvé que y avait une dame qui s'appelait comme moi, qui est partie en EMS. Et puis au lieu de couper son assurance, a coupé la mienne, je sais pas ce qu'il s'est passé. Enfin il a fallu me bagarrer deux mois pour que ça reparte. Et puis, ça a pas arrêté depuis le mois de janvier. Donc, maintenant ça a l'air d'être calmé les trucs, mais *(rires)*.

**C : Une année difficile.**

**A :** Oui, oui. Oui, parce que, oui, il vous tombe dessus des trucs, vous avez pas chercher quand même. Parce que moi je, je paie tous mes trucs *(raclement de gorge)*. Ça c'est en ordre, je vais faire mes paiements, je vais faire mes trucs, tous les mois. Et puis, j'ai jamais eu de dettes de ma vie. Alors

donc, y a, je crois que là aussi, c'est des fois géré par des personnes. Moi je veux pas qu'on gère ma vie. Jusqu' jusqu'à ce que je me rende compte que si ça va pas, bien sûr.

**C : De ça vous avez, vous avez pas besoin maintenant.**

**A :** Non.

**C : Mais si vous avez besoin une fois.**

**A :** Parce que j'ai fait un examen neurologique, pour voir, xxx, puis j'ai entendu le truc sur les cognitives aujourd'hui. Eh ben ils m'ont mis que j'ai les cognitives en ordre, et que j'avais une mémoire intacte. Ça ça embêtait beaucoup ma mère. Parce que j'ai une telle mémoire qu'elle me disait : « si je te dis un truc faux, un de ces quatre matin tu vas me voler dans les plumes». *(rires)*.

**C : *(rires)*. La mémoire est intacte.**

**A :** Ouais. Mais bon ça peut changer, hein. Ma grand-mère elle était, je sais pas quel âge elle avait, 94, 95 ans. Mais vous pouvez demander le nom des étoiles hein, incroyable la culture qu'elle avait ! Elle, c'était surtout les étoiles. Elle avait 42 bouquins d'astrologie comme ça, mais *(soupir)*.

**C : Une bonne, une bonne mémoire.**

**A :** Ah ouais ! Fallait pas lui casser les pieds, hein. Parce que j'arrivais : « ah ben tu vas chez tes tantes, moi je vais ». Elle avait eu neuf enfants : « moi je veux plus voir une couche devant moi, je veux plus voir une couche devant moi, tu vas chez tes tantes ». Très indépendante, aussi. Oui, je ressemble à ma grand-mère, quelque part.

**C : Très indépendante. Et.**

**A :** Oui.

**C : Et si on revient un petit peu par rapport à la BPCO et les informations que vous avez peut-être reçues sur la BPCO, son traitement, son évolution.**

**A :** Ben l'évolution, ça j'ai pas reçu, je sais pas comment ça marche. Ça veut dire que, on meurt de quoi là ? Ça bouche les poumons ? C'est les poumons qui meurent, c'est quoi, [exactement ?

**C : Alors ça, ouais.] Ça je peux pas vous répondre exactement vu que je suis pas du, du domaine médical, et j'ai pas envie de vous dire des, des choses fausses. Mais, mais ça par rapport à l'évolution, vous avez pas reçu comme informations ?**

**A :** Non. Et ça, ça m'intéresse, parce que je sais pas quoi, bon, j'ai vu qu'y a des gens qui arrivent à l'oxygène, donc, je sais pas si c'est les, les, les, les trous-là dans les bronches qui se ferment, qui, qui meurent quoi, je pense que c'est ça. Puisque j'arrive à 40, je suis à 40%, presque la moitié de la respiration normale. Donc y a quand même des trucs, mais enfin le dernier truc que j'ai passé là, chez le pneumologue, il m'a dit que c'était pas mal du tout, ça s'était pas aggravé.

**C : Donc ça vous, vous souhaiteriez avoir plus d'informations, [par exemple sur.**

**A :** Ouais, j'aimerais bien savoir de,] bon, on va mourir je sais à un moment donné, on peut plus respirer, je pense c'est ça, parce que. Mais, mais, je sais pas vraiment ce que c'est.

**C : Donc, ça, par exemple, votre pneumologue, ou quelqu'un, vous souhaiteriez, [pourrait vous dire.**

**A :** Il faudrait que je demande à.]

**C : Mieux comment ça se passe, [par rapport à l'évolution.**

**A :** Moi j'ai un rendez-vous] au mois de décembre, je vais vraiment lui mettre le truc sous le nez,

parce que, c'est bien joli 40% machin, chose, chouette, mais on sait rien de plus, hein. Moi j'aime bien creuser un peu, savoir le pourquoi, le comment, et les finitions.

**C : Donc ça vous, vous verrez en décembre, vous pourrez discuter de ça [avec votre pneumologue.**

**A :** Oui je vais, je vais] vraiment le secouer le médecin, parce que là maintenant moi les médecins, je vous dis, comme je vous dis c'est ordinateur, boum, boum : « bonjour madame, au revoir madame ». Ils contrôlent, mais ils nous disent rien ! On n'a aucune information ! Je sais pas, c'est des ordinateurs, et puis, ou ils pensent qu'on est les dernières des imbéciles, pis ils nous disent rien ! Je trouve que c'est assez méprisant vis-à-vis du patient de ne pas lui expliquer les choses. Ouais, ils sont assis sur leur grande chaise, et nous on est en bas. Il me semble que c'est, il me semble pas que c'était comme ça avant. J'ai eu des très bons médecins, hein. Ma foi, ils sont comme les autres, hein. Comme ils avaient 10, 12 ans de moins, plus que moi, ben ils sont décédés. Parce que je restais toujours 20 ans avec les mêmes médecins, 20, 30 ans. Mon physio, ça fait 30 ans que je vais chez lui. Donc là, mais alors là depuis, depuis cinq, six ans, je m'en, je sais pas, c'est des ordinateurs, ils disent rien, on est comme des, des espèces de paquets, qu'on a classé, voilà : « bonjour madame, au revoir madame ». Celui d'hier alors, le, le, le dermato, alors merci beaucoup, hein. Rien. Ni explications.

**C : Donc [ça vous souhaiteriez.**

**A :** Ils devraient quand même] avoir des explications puisqu'ils sont médecins ! Non ?

**C : Donc ça, ça vous souhaiteriez avoir plus d'explications, autant de la part du dermatologue, ou du pneumologue ?**

**A :** Oui ! Qu'ils expliquent qu'est-ce que c'est que ça, est-ce qu'on peut faire quelque chose, ou on peut rien faire. Mais, mais vous mettre au courant ! Parce que là ben, si on savait ce que c'est, on n'est, on n'est pas angoissé, enfin angoissé, c'est beaucoup dire, parce que, moi vous me réveillez le matin, je suis de bonne humeur, hein. Les, les dames à l'hôpital, *(rires)*, elles me demandaient : « mais comment vous faites ? Vous ouvrez l'oeil le matin, vous êtes de bonne humeur ». *(rires)* C'est un caractère !

**C : Donc ça vous angoisse pas de pas savoir ?**

**A :** Par contre, c'est quand même embêtant qu'on vous mette pas au courant de ce que vous avez. Et pourquoi, et quelle évolution, voilà. Comme ça, c'est déjà plus rassurant, on sait pourquoi on fait le traitement. Moi je, j'aurais bien aimé arrêté la cortisone, mais j'ai vu que c'est un gros problème. Parce qu'on m'a fait, sans me le dire à l'hôpital, et puis, moi j'ai pas regardé ce qu'ils me donnaient, j'ai confiance en l'hôpital, hein. Et puis tout d'un coup je me suis retrouvée, j'étais deux, deux nuits assise sur une chaise, je pouvais plus me ravoir, je sais pas pourquoi. Et ils m'avaient diminué à quatre milligrammes. Voyez, je, c'est pas normal ! Finalement les médecins, quels contacts ils ont avec les patients ? Je sais pas.

**C : Ça ce serait rassurant de, d'avoir plus d'informations, sur ce qu'on a, sur l'évolution et ce qu'on peut faire ?**

**A :** Oui, sur l'évolution. Bon, y a des gens que ça n'intéressent pas. Mais moi je pense que pour, si on a envie de faire des choses dans la vie, on veut savoir jusqu'où on peut aller. Mais à quel moment on se dit : « ben là, tu peux plus avancer, faut rester là, quoi ». Moi je vous dis ceux qui meurent d'une crise cardiaque, j'ai deux, trois amis qui sont morts, quel, quel bol, hein ! Ils s'endorment, je sais pas s'ils ressentent un choc mais, c'est rapide. On devrait tous mourir d'une crise cardiaque.

**C : C'est rapide.**

**A :** Voyez ces gens qui souffrent pendant des xxx. Moi j'ai vu ma voisine, c'était effroyable, c'était effroyable ! Et puis une voisine à l'hôpital, elle a lutté pendant 24 heures. Bon elle avait un cancer aux poumons mais, et c'était terrible, parce que, c'est comme si elle se noyait. Elle se débattait comme ça *(elle imite sa voisine qui se débat)*, comme si vous tombez dans une piscine, puis que, voyez, vous

allez, vous voulez sortir de l'eau. C'était effrayant. Et ça a duré ça, 24 heures, hein. Puis le médecin il arrive, et puis il lui dit, je sais pas quoi : « je vais vous mettre en bas l'oxygène », « Non je veux ». Au lieu de lui dire : « mais madame vous serez mieux, je vais vous descendre, y a une chambre ». Et tout ça. Il lui dit : « ben si vous voulez, si vous voulez pas, vous serez morte demain matin ». Mais je, mais c'est quoi ça, y a plus d'empathie, hein, envers les patients ! Il l'aurait pris gentiment, en lui disant : « écoutez, on va vous aider. On vous, là y a un truc exprès avec de l'oxygène ». Non ! C'était, c'était terrible, hein. Puis mon autre amie, elle avait le cancer aux intestins.

**C : C'est difficile de voir ses amis souffrir comme ça.**

**A :** Ça, justement, j'ai encore des souffrances terribles. Je crois d'ailleurs que, mon amie en X *(lieu)*, elle est morte de ça. Sa fille m'a dit que c'était effroyable parce qu'elle a, je sais pas quoi, ils l'avaient mis sous des trucs, perfusions, mais, des fois elle hurlait. Et puis comme ils sont shootés, on peut pas savoir, ils peuvent pas s'exprimer. Enfin heureusement, ça a duré deux jours.

**C : Oui c'est pas, pas facile.**

**A :** Non.

**C : Je sais pas si.**

**A :** Et c'est aussi dur pour ceux qui sont à côté. Parce qu'on peut rien faire.

**C : Les proches.**

**A :** [Ben voilà, je veux pas vous ennuyer plus longtemps.

**C : Je sais pas si y a.] Vous m'ennuyez pas du tout *(rires)*.**

**A :** *(rires)*.

**C : Je sais pas si y a autre chose que vous aimeriez rajouter, par rapport à, au traitement de la BPCO, votre participation à cette étude ? S'il y avait d'autres choses que vous aimeriez encore dire ?**

**A :** Ben non, moi je pensais que c'était, c'était bien de participer pour que vous ayez aussi plus de personnes, pour avoir une opinion quoi, pour voir comment ça marche, ça descend, ça monte, où ça s'arrête, où ça repart. Je pense que c'était intéressant à faire. Ils font aussi ça pour d'autres choses, je crois. Le protocole, en fait. Dans ce sens-là, moi je trouve que c'est bien. Autrement je, si je pensais que ça servait à rien j'aurais pas fait, hein. J'espère que ça sert à quelque chose.

**C : C'est bien d'avoir l'opinion de plusieurs personnes.**

**A :** Oui. Voilà.

**C : Bien. Ben je vous remercie [en tout cas d'avoir pris le temps de, de partager votre expérience, donner votre opinion et, d'avoir accepté de, de discuter aujourd'hui. Je vous remercie**

**A :** C'est moi, c'est moi.] Ben moi aussi ça m'a fait plaisir quelqu'un qui écoute.

**C : Oui. Je vous remercie *(rires)*, merci beaucoup.**

**A :** *(rires).*

## Participant n°14

Nom d'emprunt : Lise
Âge : 70 ans
Sexe : F
Groupe : Intervention

**C : Donc si je reprends, vous avez participé à une étude qui avait pour objectif de comparer le traitement habituel de votre maladie pulmonaire, la BPCO, avec une prise en charge précoce, soutenue et intégrée. Vous étiez dans le groupe bénéficiant de cette prise en charge globale spécialisée. Et vous avez reçu la visite d'une infirmière une fois par mois pendant un an. Pouvez-vous me raconter comment ça s'est passé ?**

**L :** Ben y a pleins, beaucoup de choses à raconter. Bon, d'une manière générale j'ai, j'appréciais bien ces rendez-vous. Moi qui aime pas tellement avoir des horaires et des contraintes, finalement, bon, faut dire que je m'entendais très bien avec *Julie (infirmière de recherche)*. Je crois que j'ai principalement eu *Julie (infirmière de recherche)* il me semble depuis le, pratiquement depuis le début. Et, c'était très décontracté. J'ai trouvé, y a toujours un questionnaire hein, à chaque fois, sauf erreur. Et je trouve que cette, ça paraît contraignant, puis finalement j'ai trouvé que c'était bien pratique, parce que ça permet de, de mettre en train, de, de cibler les, les, mieux les, les sujets qui peuvent être intéressants. Parce que c'est pas facile de trouver des choses à dire, cerner ce qui relève de, de la maladie ou de ce qui relève simplement de l'humeur du jour ou, 'fin c'est, parce que ça fait un tout quand même. Alors, donc d'une manière générale je, je m'étais vraiment très bien habituée. Et puis, bon, surtout ce que j'ai apprécié, c'est que, moi je pensais pas qu'y aurait autant de questions autour de cette maladie. Pour moi, on sort de l'hôpital, et puis c'est réglé. Peut-être qu'on a un traitement à suivre. Mais là y avait, en fait ça changeait complètement ma vie ! Puis parfois elle me, chaque fois donc y avait les mêmes questions : « est-ce que vous avez de l'aide pour ceci, pour cela ? S'habiller, se baigner, les courses ». Puis chaque fois je devais répéter la même chose, puis en même temps je me disais : « oui bon au moins, ça veut dire qu'y a des gens qui ont besoin de ça, moi j'en n'ai pas besoin ». Et puis, en plus j'en n'ai <u>toujours</u> pas besoin au bout de, au total ça fera, ça aura fait plus que six mois, je sais plus. Ça fait combien au total, depuis le début ?

**C : Alors là c'était 2015 vous m'avez dit.**

**L :** Donc 2015.

**C : Donc là ça ferait déjà un an et demi, si c'est depuis début 2015.**

**L :** Là je vois mai, 18 mai. Alors, ah ben oui alors ça fait, en juillet, août, oui, bon… Avril, mai. Oui, ça fait six moi je pense hein, je crois pas que ça ait duré plus longtemps. Alors elle m'a aussi, surtout apporté des, des, des éléments pratiques que personne d'autre pouvait me donner, en fait. Les médecins ils nous traitent pas comme ça. Donc elle était, en fait elle faisait la transition entre, le côté simplement humain, les questions banales et puis le médecin qui répond jamais à ces questions : « comment est-ce qu'il faut manger » ou « qu'est-ce que je pourrais faire pour respirer mieux ». Souvent, bon, le pneumologue il, même le pneumologue, dans mon cas en tout cas, il entrait peu, la physio, apparemment c'était, c'est pas encore acquis, en tout cas chez ce pneumologue-là. Entre-temps j'ai changé, donc, et je m'en félicite, et visiblement il connaît mieux ces choses-là. Mais, donc, <u>elle</u>, elle, elle avait réponse à toutes mes questions. Et donc tout au début, bon vous me re ciblez hein, si, si je réponds pas à vos questions.

**C : Oui, oui, oui. Non, non, non, non c'est *(rires)*.**

**L :** Par exemple, y avait des choses toutes bêtes, comme la physio respiratoire, ici à X *(ville)*. J'avais un numéro, donc je suis allée à X *(hôpital)*, faire trois semaines de physio, au mois de mars, mars-avril, 2015. Et, on nous a donc recommandé de continuer la physio ici. Et, j'avais un numéro de téléphone et, et j'arrivais nulle part. Après je suis encore allée à X *(hôpital de réhabilitation)*, prospecter, parce que je me disais que X *(hôpital de réhabilitation)*, donc c'est, c'est le, la médecine de, réhabilitation on pourrait dire. Et ils ont justement des salles de sport, parce que y a, beaucoup de gens accidentés à X *(hôpital de réhabilitation)*. Et, impossible d'avoir une information ! Jusqu'à ce que, justement *Julie (infirmière de recherche)* me dise : « ah mais essayez ce numéro-là ». Donc, j'ai passé un temps fou, et elle, elle m'a donné la solution comme ça. Et la même chose pour les, les protéines. Donc à l'hôpital on nous donnait du X *(produits pour la nutrition clinique)* tous les jours. Puis j'ai dit : « mais on trouve ça où ? » Puis

personne avait vraiment pu me répondre. Et, ben, c'est, encore une fois c'est *Julie (infirmière de recherche)*, non seulement elle m'a donné le numéro, mais finalement, elle a fait tout le nécessaire, j'ai pas eu à lever le petit doigt. Mais ça c'est un bonheur ! Quand on se sent comme ça, épaulée. Moi j'ai beaucoup, je pensais pas que j'aurais, j'en n'avais pas besoin, ou je pensais pas en avoir besoin, mais en réalité, ça, je crois que pour moi ça fait toute la différence, vraiment.

**C : Donc elle a pu vous, vous épauler, [répondre à vos questions ?**

**L :** Oui.] Ça, ça m'a permis d'être beaucoup plus détendue, je pense. Parce que c'est une maladie qui est angoissante *(elle boit un verre d'eau)*. Parce que pour moi le, je pense que c'est pour beaucoup de gens comme ça, ce qui est angoissant c'est de pas comprendre, c'est de pas maîtriser. Tout d'un coup on est essoufflé, après on l'est pas. Je fais le même parcours, les mêmes gestes, puis je suis essoufflée, ou je le suis pas. Je me lève, c'est normal, enfin à mon niveau. Et puis d'autres fois je me lève, j'arrive à la cuisine, je fais donc trois pas, et puis je peux plus souffler. Puis j'ai pas la réponse, je sais pas pourquoi ! Alors elle, je pouvais lui en parler, et puis, bon ben, y a toujours des explications. Et puis alors elle me stimulait aussi pour faire des exercices. Parce que je dois dire que la physio moi j'y allais à reculons. D'ailleurs j'ai pas continué, ce qui est, une grave erreur. Elle était plus là non plus pour me donner des coups de pied au cul *(rires)*. Parce que je râlais, parce que c'était. Bon déjà pour aller dans cette salle de physio à l'hôpital cantonal, c'est des kilomètres ! Donc on arrive on est déjà sur les genoux. Et puis c'est tellement intense. Bon donc moi on me l'a assez répété, donc je sais que si c'est pas intense, ça sert à rien, ben disons que c'est de la rigolade. Donc il faut souffrir. Et moi ça me, j'avais pas le courage ! On avait beau me dire : « mais tu verras tu, en sortant t'es contente ». Moi non, c'est pas. Donc là j'ai arrêté, puis j'ai plus, plus rien fait. Et puis ça s'est dégradé, j'avais donc des gros problèmes. Et je suis retournée à X *(hôpital)*, maintenant y a, c'était quel mois, octobre.

**C : Donc *Julie (infirmière de recherche)*, l'infirmière a pu vous pousser un petit peu pour ces exercices, la physio.**

**L :** Alors là, bon évidemment on nous re, re redit tout ce que, tout ce que je savais déjà, mais c'est vrai que quand on, on est en face de la personne qui nous le dit, c'est pas la même chose. Mais, là j'ai toujours pas commencé la physio. Bon c'est, faut dire qu'elle a, elle était pas disponible, donc pas à l'hôpital. Et j'ai cru comprendre que ça existait plus à l'hôpital, mais vous vous êtes pas sur X *(ville)*, je crois ?

**C : Non.**

**L :** Donc vous savez pas. Mais, donc la physio, y a un, y a une… Une, une salle de physio avec deux physios, donc j'ai cru comprendre que c'était privé. J'avais donc le numéro de téléphone, que mon pneumologue m'a donné. Et puis elle était, si j'ai bien compris elle était toute seule ou peut-être deux, et puis je lui ai dit : « puis y a aussi l'hôpital ». Elle m'a dit : « mais ça, ils ont arrêté ». Alors je comprends pas très bien parce que dans un des derniers numéros de, dans « *Pulsation* », donc la brochure de, qu'on distribue à l'hôpital tout public, ils, ils parlaient justement de cette physio et, et que c'était formidable, et que, et que. Alors bon, donc j'ai pas commencé, et ça fait maintenant, presque un mois que, que je suis rentrée à la maison. Enfin c'est en route *(rires)*. Qu'est-ce qu'on a fait encore ? Ben des choses, oui des choses à laquelle, c'était pas seulement en rapport avec la BPCO, c'était aussi global, santé globale, comme par exemple les directives anticipées. C'était, voilà, de ces choses qui sont, pas faciles à, à aborder, qui prennent du temps, puis on sait pas à qui s'adresser, même des fois on va sur internet, c'est pas, c'est pas clair. Donc là par exemple les directives anticipées, elle m'a, elle m'a mâché le travail. Et elle me les a même, rédigées. Donc j'en ai deux en fait, j'ai celle de l'hôpital, une formule officielle, mais je crois que c'est l'hôpital qui m'ont fourni, ou bien non c'est Exit. Et puis l'autre, qui est sur mesure. Donc les deux sont déposées maintenant dans mon dossier à l'hôpital. Et puis la même chose pour Exit, ça c'est moi qui l'ai, qui l'ai amené sur le tapis. Donc ça, c'est, c'est vraiment que du bonheur !

**C : Donc ça vous avez pu rédiger ensemble, [ces directives anticipées ?**

**L :** Oui.] Moi j'estime qu'elle a, elle a vraiment fait un sacré boulot !

**C : Comment ça s'est passé pour la rédaction de ces, ces directives avec ?**

**L :** Ben oui, moi j'aurais jamais su quoi mettre ! Bon y a des modèles, mais ça je savais pas non plus, qu'y avait un modèle. Je savais pas qu'Exit, en avait, en avait un, des, en avait des toutes faites ! Mais qui se

font, c'est les mêmes pour tout le monde. Alors la xxx, quand je suis retournée à l'hôpital, j'ai fait la maligne, j'ai dit, oui c'était pour, mais alors c'était après… L'opération de la hanche, non c'était pas après. Je sais plus quelle intervention j'ai dû. Ah oui parce qu'entre-temps, on, on m'a posé des « coils », vous savez des spirales, y a un autre nom d'ailleurs pour ça plus français, c'est des petites agrafes qu'on met pour resserrer les parties poreuses du poumon, j'explique avec mes mots hein. Puis c'est censé libérer de la place pour le poumon, ça. Donc on a voulu essayer ça, y a, on, on ne sait pas du tout le résultat, on peut, on peut absolument pas garantir, surtout que c'est assez récent. Apparemment, ça m'a pas fait d'effets. Mais bon y a deux interventions. Et, justement à l'occasion de cette intervention, le médecin arrive et puis : « oui, alors ». Puis il est très gêné : « si jamais, y a des complications ». Alors là moi, relaxe : « c'est dans mon dossier ». Et c'est, c'est précieux ! Parce que c'est, c'est pas très confortable ce genre de questions, quand on n'est pas préparé. Donc là vraiment. Et, c'est pas la même chose non plus quand c'est des copains, des copines, qui ont été malades ou qui ont l'expérience. Moi, en tout cas ça me, je, je le… Je fais pas confiance comme ça. Tandis que là, je pouvais y aller les yeux fermés. Donc y avait ça aussi, les directives anticipées.

**C : Donc là, c'était confortable pour vous de, d'avoir pu rédiger ces directives [anticipées ?**

**L :** Ben oui !] Oui alors pendant la semaine, moi je notais tout, tout ce que je trouvais bizarre. Par exemple tout, je vois là j'ai marqué : « cheville enflée » ; « reflux ». Oui, parce que j'avais des, je sais pas, j'avais un problème de digestion, et puis je comprenais pas ce que c'était. Ben je lui demandais à elle ! C'était.

**C : Vous pouviez poser toutes ces questions et.**

**L :** Et puis aussi. Donc ça c'est le côté plutôt physiologique. Mais y avait aussi, le, le côté moral, oui mon, mes états d'âme. J'avais pas de scrupules, non plus. Puis, elle savait aussi très bien m'amener à dire certaines choses. Donc quand j'avais pas le moral, on pouvait en parler. Elle, elle me donnait toujours quelq', elle repart', elle me laissait toujours quelque chose de, de positif, à faire ou… *(elle boit un verre d'eau)*. Et moi je pense que ça ça. Donc, c'est pas des médicaments, mais je suis convaincue que moi je me porte mieux que des gens qui sont sans ces informations. Ne serait-ce déjà que le, le, la relation à la maladie. Parce que c'est une maladie vraiment perverse, je trouve qu'elle est, elle est, elle est pas quantifiable, elle est pas, elle, on peut pas la maîtriser. Bon, moi je suis, surtout que je suis pas sportive, moi je, j'aime bien être à la maison, donc faut vraiment que je me force pour faire de la, des exercices. Et, ben si j'ai pas des encouragements, eh ben je serai prétéritée par rapport, si j'avais que, autrement, je serais plus prétéritée que si j'avais pas eu ces, ouais bon.

**C : [Ces informations.**

**L :** C'est clair *(rires)*.]

**C : Oui *(rires)*. Donc là vous avez pu avoir des informations sur la maladie, aussi sur d'autres choses, [vous disiez, pu poser des questions, et.**

**L :** Oui, oui.] Et puis elle a, bon faut dire aussi c'est la personne elle-même ! Ça aurait été quelqu'un d'autre, j'aurais peut-être pas du tout eu cette, ce, cette bonne relation, ou ce, la façon de dire les choses, par exemple. Moi j'ai horreur qu'on me fasse, qu'on me, les gens qui positivent beaucoup : « oh vous verrez, ça vous fera du bien ! Mais oui, faites ça ! », moi, moi alors y a rien de tel pour me, me braquer. Je dis : « ouais cause toujours ». C'est des gens qui, qui croient qui savent ce qui est bon pour nous. Mais y a une façon, ils le savent. Bon, d'accord, c'est normal qu'ils le savent, c'est leur métier. Mais y a une façon de le transmettre. Et *Julie (infirmière de recherche)* elle a, elle, bon d'abord c'était, c'est une personnalité très gaie, très positive, donc déjà rien que sa façon d'être, c'est convaincant *(rires)*. C'est un bon exemple. Et puis, oui sans, je dis pas que je pétais le feu chaque, après quand elle partait mais, c'est un soutien quand même !

**C : Donc y avait ce soutien et un bon contact [avec *Julie (infirmière de recherche)*.**

**L :** Ouais.] Vraiment, c'est le mot soutien. Un soutien qui, qui, qui durait, puis jusqu'à la fois d'après, puis qui, qui se renouvelait. Elle serait contente, si elle m'entendait. Bon mais, je crois qu'elle a, elle sait que je l'ai appréciée.

**C : *(rires)*. Plutôt quelque chose que vous avez apprécié ces visites, [de l'infirmière.**

**L :** Ben oui !] Alors j'ai pas le souvenir, peut-être que ça m'ennuyait des fois. Mais, ça devait pas être vraiment… Et, et j'étais aussi frappée, y a des choses qu'on m'a dites à X *(hôpital)*, que les médecins disent, que je retrouve que *Julie (infirmière de recherche)* disait ! Là je vois, chaque signe est à surveiller. Par exemple une petite toux. Mais y a encore quelques mois de ça, je toussais, ben je suis arrivée à X *(hôpital)*, je tousse tous les jours ! Puis je dis : « mais écoutez, moi je suis inquiète, c'est pas, c'est pas normal cette toux qui traîne ! ». Mais c'était pas une petite toux, c'était une toux tous les jours ! Et ils ont confirmé que les petites choses sont importantes, puisque comme on a des capacités réduites, le plus petit effet fait un grand effet. La plus petite cause a un grand effet. Mais ça, *Julie (infirmière de recherche)* elle me l'avait déjà dit. Bon moi il faut qu'on me redise des fois. Alors, moi je crois que je.

**C : Et en termes d'informations, est-ce qu'y aurait d'autres choses dont vous auriez eu besoin ou ?**

**L :** Qu'elle m'a pas donné, vous voulez dire ?

**C : Oui, oui pendant ces visites… Des informations supplémentaires que vous auriez peut-être aimé avoir ?**

**L :** Je crois pas... Comme je vous dis, j'ai un petit peu oublié, hein. Peut-être l'histoire du pneumologue. Parce que là, peut-être qu'elle aurait pu se permettre d'être un peu plus intrusive, parce que je lui ai, je lui disais : « mais ce pneumologue, il est quand même bizarre », « je sais pas, je le sens pas ». Pourtant ça fait des années que je l'ai mais, c'est pas le médecin que je vais voir le plus souvent, heureusement ! Avant ça, j'allais pas le voir très souvent. Et puis, je sais pas, je le sentais pas, puis il voulait tout le temps faire des radios. Parce qu'il a, il a l'équipement sur place. Alors c'est bien agréable, mais en même temps, ou bien j'arrivais, puis il me disait : « alors qu'est-ce qu'on fait ? » ; « ben euh, c'est vous le médecin je crois ». Et justement la dernière fois qu'il m'a fait le coup, j'avais déjà été ben, j'avais eu la pose des « coils », donc j'étais, j'étais suivie. Et puis, il avait pas vu le rapport. Alors là ça m'a, pour moi ça a été la, ça a été le signal que vraiment fallait que je change de médecin. Parce que il a passé plus de temps à chercher mon, le rapport dans, dans, dans son ordinateur qu'à me donner une vraie consultation. Et puis il a voulu faire une radio, puis j'ai dit : « mais je vais justement faire un contrôle dans trois jours ». Donc, parce que j'avais un contrôle de suivi à l'hôpital, aussi, tous les trois mois, suite à cette pose de « coils ». Et il faisait des radios et un test de marche. Donc évidemment le pneumologue, ben il avait plus grand-chose à faire ! Et là je pense que j'étais aller le trouver parce que j'avais, ben visite de, de contrôle, parce que je l'avais pas revu depuis une année puis que il avait justement pleins de nouveaux trucs dans ses dossiers, qu'il n'avait pas trouvé. Alors j'ai piqué une rage et puis j'ai changé, puis je suis très contente, il est très, très bien celui que j'ai maintenant. Donc, là, elle a peut-être été trop respectueuse. Elle aurait, je sais pas, elle aurait peut-être, ou elle aurait pu me dire directement d'aller voir un autre médecin. Ça aurait peut-être un peu, été un peu plus vite.

**C : Faire avancer les choses [un peu plus rapidement.**

**L :** Oui.] Et puis donner, m'encourager à changer parce que changer de médecin, c'est pas évident ! Surtout si y a longtemps qu'on va chez le même ! Moi d'ailleurs je lui ai rien dit, je lui ai pas encore dit. Je me suis dit, après tout il est tellement désordre *(rires)*. Je l'ai dit à sa secrétaire, j'ai, j'ai dit : « qu'est-ce que, comment ça se passe si on change de médecin, si j'arrête de venir chez vous ? » ; « ah ben faut récupérer le dossier ». Puis patati, puis patata. J'ai dit : « ouhlàlà ! Pour l'instant, je bouge pas ». Donc là peut-être, elle aurait, mais justement ça, elle était très discrète ! Autrement, je vous dis je sais pas, je me souviens pas.

**C : Est-ce que y a des, des choses, qui vous a particulièrement plu ou déplu dans, dans ces visites ?**

**L :** Je pense ce qui m'aurait le plus ennuyé, peut-être, c'était la, la redite, parce que autant, y a le côté positif, et évidemment je respecte, c'est nécessaire à, à l'étude. Ils peuvent pas se baser comme ça sur des discours, faut bien faire des questionnaires. Mais ça, ça me cassait un peu les pieds pour finir, c'est toujours les mêmes questions. Et puis moi c'était toujours les mêmes réponses *(rires)*. Et puis des fois je me disais : « ouais mais, je suis pas concernée par ce questionnaire ! ». Justement dans, y a toute une, une catégorie de questions où, sur les aides. Et là depuis, à partir du moment où on dit non à la première, on dit non jusqu'au bout. Et ça, ça changeait jamais. Alors ça m'énervait mais, c'était pas vraiment justifié, mais ça m'énervait quand même. J'avais envie de dire : « mais voyez, vous savez bien ! » *(rires)*. « Est-ce que j'ai l'air d'avoir besoin qu'on me fasse prendre ma douche ? ». Puis en même temps l'autre jour,

j'avais, justement quand j'avais comme ça plus de souffle, je me suis dit : « ouais, ben peut-être bien que j'aurai besoin d'aide » *(elle boit un verre d'eau)*.

**C : Plus ces questionnaires qui étaient parfois un peu de la redite, [voilà, plus par rapport aux questions.**

**L :** Ouais.] Bon alors, au début évidemment je, comme je suis très scrupuleuse, je réfléchissais xxx. Y a cette ambiguïté, faut répondre vite, faut pas trop réfléchir, mais en même temps faut être, faut être, faut que ça soit juste. Et puis alors après, au bout du je sais pas, au bout du troisième ou du quatrième, c'était « vroum, vroum, vroum ». Je m'en faisais plus tellement. Et puis c'était plus, plus facile. Mais c'est vrai que ça, ça m'agaçait un petit peu.

**C : Vous disiez que ça a pu vous faire aussi un peu réfléchir par rapport à toutes les dimensions autour de, de la maladie ?**

**L :** Ça aurait pu mais je l'ai pas utilisé comme ça, non. Pas, non je me rendais pas compte. Je me comparais pas à moi-même, en me disant : « ça pourrait m'arriver ». Je pensais plutôt : « y a d'autres gens qui sont concernés, moi je le suis pas ». Mais je dois dire qu'à un moment donné, c'est comme l'oxygène, quand j'étais dans, vraiment si mal y a, y a deux, avant d'aller à X *(hôpital)*, donc y a trois mois, deux mois ou trois mois, eh ben j'avais, je me disais ben : « si ça se trouve l'oxygène il est pas loin ». Alors que, avant je, j'étais convaincue que j'aurais jamais besoin d'oxygène!

**C : Là vous vous êtes dit que peut-être un jour.**

**L :** Alors là, oui, surtout à un moment donné le, le pneumologue il est, tout en écrivant, puis il avait l'air de penser comme ça. A voix haute, il dit : « ouais alors y a pas, on n'a pas besoin d'oxygène ». Puis je me suis dit. Moi dès que j'entends le mot, c'est *(elle imite un bruit de panique)* !

**C : Par rapport à l'oxygène ?**

**L :** Oui ! Et à X *(hôpital)*, ils m'ont dit : « non, je pense que vous en aurez pas besoin ». Mais je vois bien qu'ils, ils, ils, ça fait partie de : « est-ce qu'elle en a besoin quand on a des normes, des valeurs qui sont limites ». Et puis, j'ai dit : « toute façon il en est pas question, je me flingue » ; « ah bon, pourquoi ? » ; j'ai dit : « je veux pas de cette machine chez moi ! » ; « ouais mais ». Bon, je sais pas ce que je ferais en réalité.

**C : C'est pas quelque chose que vous aimeriez avoir ?**

**L :** Vous croyez que ce serait beau à une bonbonne à, à oxygène là au milieu *(rires)* ? Ça va juste dans les couloirs d'un hôpital.

**C : *(rires)*. Oui. Vous disiez c'est une maladie qui est assez angoissante, de manière générale.**

**L :** Si on, si on, on gratte un petit peu, ça peut être très angoissant. Et puis bon, c'est surtout le, la, la non maîtrise, ouais, j'ai aucune, l'impression d'avoir aucune emprise dessus. Et c'est vrai qu'y a que la physio qui, qui, qui, qui change la donne ! Entre le jour où je suis arrivée à X *(hôpital)*, et le jour où j'en suis sortie, mais y a un monde ! Et là je suis en train de jouer avec le feu, parce que bon je monte les escaliers, je. Mais le premier jour, je suis rentrée de X *(hôpital)*, le lendemain, il pleuvait des cordes, il faisait froid, enfin c'était la totale ! Et puis, je me suis dit : « tant pis ! » Je me suis équipée, moi qui sors jamais quand il pleut *(rires)*. J'ai tout pris, plus un petit, un, un, un bout de plastique, et pour poser sur un banc pour m'asseoir. Et j'ai bien fait, mais heureusement qu'y a des bancs là le long, mais j'ai dû m'asseoir je ne sais combien de fois ! J'étais là sous mon parapluie, en train de reprendre mon souffle. Et, c'était le lendemain de X *(hôpital)* ! Et à X *(hôpital)*, la, la même distance je la faisais sans problèmes ! Alors X *(ville)*, c'est pas un cadeau.

**C : La ville ?**

**L :** Ah oui ! Tout le monde le dit, on le sait maintenant. Pour les asthmatiques, les, les, les bronchites, c'est vraiment de la saleté ! Alors bon, alors peut-être justement, par exemple si Julie *(infirmière de recherche)* venait maintenant dans cette période, peut-être que je serais mieux, plus stimulée à, à, à téléphoner pour

me reprendre un rendez-vous de, de physio.

**C : Pour la physio. Quelqu'un qui arrive à vous motiver à, à.**

**L :** Parce que c'est pas encore assez ce que je fais. C'est, c'est, oui, c'est homéopathique. Je monte un ou deux étages, je suis déjà contente de pouvoir en monter un ! Avant je pouvais même pas monter un étage. Là c'est un ou deux, et puis y a, bon on on a un ballon, à presser comme ça. Tout ce qu'on peut faire comme ça, tout seul, ça je le fais. Mais c'est pas très, pas très physique quoi, c'est pas très fort. Là je pense qu'elle me, elle, elle me stimulerait.

**C : Parce que ça vous fait du bien la, la physio, les trois semaines que vous avez fait à X *(hôpital)*, vous avez vu la différence.**

**L :** C'est, c'est magique, c'est magique ! J'ai envoyé un message à mon pneumologue pour lui dire que tout allait bien et que j'adorais X *(hôpital)*. Et puis il m'a répondu : « vous êtes bien la première patiente que j'ai qui rouspète pas ». Mais moi je suis comme ça, je suis là pour ça. J'ai que ça à faire toute la journée ! Donc je le fais ! Et avec plaisir ! Tandis qu'ici, dans, dans, j'ai mille autres choses que j'ai envie de faire ! Bon y a, y a pleins de choses que je fais plus, et que je faisais avant c'est que moi je, je me déplace en scooter. Alors là aussi *Julie (infirmière de recherche)* elle me donnait, me disait : « mais vous prenez le scooter jusque-là, puis après vous allez à pied ». Enfin, mais des petites choses très pratiques que je pouvais visualiser et, et ça marchait. Elle m'en mettait jamais plus hein, elle devait savoir qu'il fallait pas trop pousser non plus. Et là maintenant, je prends, je prends pas mon scooter, je, je vais au bistrot, c'est, c'est mon bout de marche, où y a les bancs, où donc j'ai aucune excuse à le faire. Mais c'est très, très, très, très, très pénible. Et c'est pas les vingt minutes qu'on est censé faire par jour, jamais !

**C : *Julie (infirmière de recherche)* elle a pu vous donner justement ces petites astuces pratiques, comme avec le scooter ou des choses. Est-ce qu'y aurait d'autres choses pratiques qu'elle a pu [transmettre ?**

**L :** Oui. Ben comme je vous dis,] *(rires)* c'est, je vois, ben je vois d'après mes notes *(elle regarde dans un cahier de notes)*, y a pas tant que ça finalement que j'ai noté. Oui, y avait des choses pour l'alimentation. Je sais qu'elle m'avait, carrément fait un programme, j'ai, je pense que j'ai pas noté. Mais… Parce que j'avais perdu, ouais j'étais vraiment trop maigre quoi. Je sais pas combien de kilos j'avais perdu. Et, donc l'alimentation, il fallait que je mange des trucs bien, bien précis dont les protéines. Alors là je lui disais : « oh mais j'aime pas ça, puis, oh, puis, puis moi j'aime, le matin j'ai pas faim ». Mais elle avait réussi à me trouver comme ça de, alors elle me : « puis ça, vous aimez ? Ah puis, puis vous pourriez cuisiner comme ça ce que vous aimez ». Parce que, je sais pas, si je disais : « j'aime bien le fromage ». ; « ben, ah mais vous pourriez faire une omelette au fromage ». ; « ah oui, ça j'aime bien, d'accord ! » Donc c'était, c'était vraiment une collaboration. C'était pas que du soutien. Elle avait un don pour ça. Et en même temps je pense qu'elle ça lui permettait de, de mieux, mieux voir le, le fonctionnement aussi. Parce que comme j'ai, là-dedans j'ai toutes les notes hein *(elle montre son carnet de notes)*, donc c'est aussi bien les médecins que, alors *Julie (infirmière de recherche)* elle est, elle est par dedans *(rires)*, c'est dans l'ordre chronologique.

**C : Puis elle arrivait à pas trop vous pousser non plus, à savoir la bonne dose.**

**L :** Oui. Ben là par exemple je vois, beaucoup de choses comme la *(toux)*, le, ada', changer de point de vue. Je vois là j'ai noté : « me dire, j'ai déjà tellement gagné ». Alors moi je disais : « oui mais j'ai perdu ça, puis j'ai perdu ça, puis j'ai perdu ça ». Donc faut pas partir de, quand j'avais vingt ans hein, j'exagère, mais faut partir de la situation de départ, puis de voir tout ce que j'ai gagné. C'est, c'est des bêtises, mais moi c'est des petites choses comme ça que je supporterais pas suivant qui me le dit. Dis : « ah ouais c'est de la, de la morale de, de, bon marché ». Mais là c'était juste bien ciblé. Ah oui puis alors y avait bon, évidemment, je sais aussi pourquoi y a ça, c'est la cigarette. Parce qu'alors moi j'ai, bon j'ai pas arrêté de fumer pendant toute cette période. J'ai, j'ai, j'ai joué avec, j'en enlève une, j'en enlève deux, aujourd'hui j'en achète pas. Mais au bout du compte, j'avais toujours ma dose, ce qui était environ un paquet par jour, il fallait que je maintienne cette dose. Mais, je pouvais ne pas fumer pendant un ou deux jours, ou bien après c'était cinq jours, xxx, je me disais : « c'est un exploit ». Maintenant j'en peux plus. Et, alors là elle a été aussi d'un, d'un bon soutien, et elle m'a jamais dit : « faut arrêter ». Elle m'a jamais peint le diable sur la muraille, parce qu'elle savait que je savais. C'est ça je pense aussi qui, elle, elle m'en donnait pas plus que ce dont j'avais besoin.

**C : Moralisait pas, et.**

**L :** Oui. Et ça, quand on, quand, je sais plus qui est venu à l'hôpital, ça devait être *Claire (infirmière de recherche)* je crois. Tout d'un coup y a : « y a une dame qui veut vous voir ». Et puis on m'a présenté l'étude. Et puis, moi j'ai tout de suite dit oui, d'abord parce que j'aime bien ça. J'adore les tests *(rires)*. Et puis que je suis convaincue de, de l'utilité de le faire ! Parce que j'ai appris que y avait beaucoup de gens qui refusaient, dans l'équipe où on était tous les malades qui étaient là, je sais pas mais je crois bien que je suis la seule, ou on était peut-être deux à avoir accepté. Donc, si on le fait pas, ben on peut pas faire avancer non plus la, la médecine ! Donc je l'ai fait avec conviction, mais, la personne qui, qui m'a présenté ça, ben je sais pas si j'aurais continué avec elle, voyez ? J'aurais peut-être pas été jusqu'au bout.

**C : Mais là vous aviez un bon contact avec *Julie (infirmière de recherche)* qui est venue ?**

**L :** Oui.

**C : Et donc vous avez, voilà accepté aussi de participer à l'étude parce que, vous pensiez que c'était utile et c'est quelque chose que [vous aimez faire aussi de participer ?**

**L :** Oui.] Oui, puis de, bon, j'en ai pas, je sais pas j'en ai peut-être, je fais des, j'aime bien faire des tests, mais, je sais pas si j'en ai fait d'autres, peut-être une ou deux de ces. Oui j'en ai fait. Y en a qui sont simplement, je veux dire, comment on dit, par la poste, hein on n'a pas de rencontres. Mais ça, en général je fais si c'est pas pour de la, pour de la pub, je le fais. Et… Et puis bon, je savais que je pouvais arrêter quand je voulais, qu'on m'en tiendrait pas rigueur, qu'on chercherait pas à me rattraper, donc c'était facile d'accepter.

**C : Et qu'est-ce que vous attendiez en participant à, à cette étude ?**

**L :** Ben je pensais que j'allais être quelqu'un de bien qui allait offrir mes, mon vécu. Je m'attentais pas du tout à ce que ça puisse être une aide ! Mais pas un instant ! Puis en plus je savais pas que j'avais besoin d'aide, j'étais, je savais pas ce qui m'attendait, dans l'avenir, puisque j'étais encore hospitalisée. Non, non je me disais : « ben je fais une bonne action, ça sera utile à la société ». Et puis, puis voilà. Et puis j'étais curieuse, parce que je trouve intéressant, en plus, je veux dire intellectuellement aussi ça m'intéressait.

**C : Voilà vous aviez, vous aviez pas d'attentes pour vous, mais c'était pour, [pour les autres ou pour la science.**

**L :** Mais non pas du tout ! Donc c'était une bonne surprise aussi.]

**C : Une bonne surprise que ça vous a apporté quelque chose ?**

**L :** Oui. D'abord je savais pas que j'aurais autant de questions pratiques, du quotidien. Et puis je savais pas que ça pourrait rentrer dans, dans le cadre de l'étude. Pour moi ça. Et puis le médecin, c'est des questions qu'on pose pas au médecin parce que, bon le temps est chronométré. Et puis le temps qu'on ait parlé de tout ce qui est, ce que eux veulent entendre, ben y a, c'est rare, moi je fais pourtant des listes maintenant, quand je vais chez le médecin, pour pas me retrouver dehors en disant : « j'ai oublié de lui dire ça, puis ça, puis ça ». Et, mais malgré tout, c'est pas à lui que je vais demander si, ou alors, en tout cas pas chaque fois, si je peux manger, je sais pas des carottes, si j'ai le droit de, hein, de faire des choses ou, ou si je marche dix minutes, est-ce que ça suffit. C'est pas à lui que je vais demander ça, sauf si, s'il l'inclut dans la consultation. Et là je vois, bon les « coils », c'est, c'est ce pneumologue dont je, je vous parlais avant qui me, qui me les a proposé. Ça c'était, c'était bien. Mais des trucs tout bêtes comme par exemple, des choses qu'on m'avait dites à l'hôpital, mais qu'elle m'a redit, quand on fait les, les sprays, faut se rincer la bouche, c'est tout bête. Et moi je le faisais pas, enfin j'y pensais pas. Donc c'est des piqûres de rappel, comme ça. Et puis elle parlait aussi des médicaments, de temps en temps on, elle me disait : « vous prenez toujours ci ? Ou qu'est-ce que vous avez ? ». C'était très, très, très global… Ou je pouvais aussi savoir, y a des trucs qui sont assez, assez pointus comme la lecture des courbes, des tests de, des fonctions respiratoires, ou, ou pourquoi j'ai tel médicament, à quoi il sert, je sais plus, j'ai oublié, est-ce qu'y a pas une interaction avec celui-là. Parce que la pharmacie bien sûr surveille ces choses-là. Mais bon on n'a pas, moi j'avais pas le détail. Et puis, qu'est-ce que je disais ?

**C : Par rapport aux traitements.**

**L :** Oui.

**C : Elle a pu vous donner aussi des informations par rapport aux, aux médicaments que vous deviez prendre, c'était assez large.**

**L :** Oui des trucs, des trucs tout bêtes, qu'on m'avait déjà dit, ou alors moi je mets tous les médicaments sur la table, je les prends tous en même temps. Et, heureusement c'est un par jour. Mais en fait y en a que je devrais prendre avant d'autres, et laisser passer deux heures. Et ben elle elle me l'a, elle me l'a rappelé. C'est bête, hein.

**C : Elle vous, vous rappelait des informations que vous aviez reçues ailleurs, comme à l'hôpital ou du médecin, les rappeler.**

**L :** Oui, oui je disais : « ah oui c'est vrai ». Je le savais, comme les gosses *(rires)*. J'étais un peu à l'école avec elle. Alors elle m'a aussi, ben c'est elle, sauf erreur c'est elle qui m'a parlé de X *(hôpital)*, mais là je suis pas sûre. Mais là je vois que le X *(date)*, j'ai marqué « hôpital de jour ». Donc elle m'a proposé quelque chose là, mais c'était, c'était comme les aides au ménage, les aides à domicile. Elle, elle m'a fait une ou deux fois, mais je suppose que c'était dans le cadre de son, ça faisait parti des consignes, à mon avis, pour elle. Mais des choses qui étaient vraiment au-dessus de mes besoins, genre cet hôpital de jour, moi, je sais pas on allait une journée, une à deux fois par semaine à X *(hôpital psychiatrique)*, X *(hôpital psychiatrique)*, c'est l'hôpital psychiatrique. Je veux dire, eh mais ça va ! Oui, parce que j'étais déprimée. Mais, je lui ai dit : « mais je serais encore plus déprimée si je fais ça ! ». Et là elle, elle avait, oui, elle m'en a parlé assez longtemps puisque j'ai un, un paquet de notes. Mais ça aurait pu m'être utile.

**C : Donc là, là ça vous était pas utile, mais vous avez pu discuter de.**

**L :** Oui. Ah mais je vois qu'elle est venue cette, elle a, on est allé plus loin que ça. La médecin, la femme médecin de cet hôpital de jour, on s'est rencontré, donc faut croire que j'avais pas été assez convaincante quand je lui ai dit que je voulais pas y aller. Alors à la fois d'après quand, quand j'ai revu *Julie (infirmière de recherche)* toute seule, je lui ai dit : « oh ben non alors ! ». Elle m'avait pas plu du tout la, la, cette médecin… Je trouve rien de, je sais pas ce que je pourrais [vous proposer de.

**C : Parce qu'en termes voilà,] en termes d'aides, ou de besoins, vous aviez pas forcément besoin de, de toutes ces aides ?**

**L :** Alors je sais pas, j'en aurais peut-être, ça m'aurait peut-être été utile, j'en sais rien. Mais j'en, je, moi je le sentais pas.

**C : En tout cas pas maintenant.**

**L :** Je suis sûre, voilà, ce que je peux dire c'est que je suis sûre qu'y a des méthodes, des, qui sont beaucoup plus efficaces, à portée de main, pour se redonner le moral ou pour avoir une meilleure santé, sans qu'on ait recours à des professionnels. Mais il faut le faire, simplement ! Moi j'ai, j'ai, j'ai un carnet d'adresses, je peux téléphoner à mes copines, ça sera tout aussi utile que d'aller en, faire une journée de, de traitement. Ah oui puis, surtout je crois, je me souviens maintenant aussi pourquoi je lui ai dit : « non, non ». C'est que, je sais, je connais quelqu'un qui fait ça. C'est qu'on est, les, les gens qui sont réunis là, à cet hôpital de jour, sont tous très différents ! Ils ont tous des problèmes complètement différents ! Et je pense, on leur, moi je le vois d'ici, on leur met des crayons de couleur, des feuilles, et puis ils font des *(rires)*, comment ça s'appelle là déjà ces dessins, vous connaissez ?

**C : Des mandalas *(rires)* ?**

**L :** Rho. Ah moi je pars en courant quand je vois ça *(rires)* ! Ou bien alors de la musique, ou bien je sais pas quoi. Y a peut-être des trucs, sur une journée, mais dans une journée, il doit y avoir 80, pour moi, 80% d'ennui ! C'est, c'est très critique, je sais pas, j'y suis pas allée, j'ai pas. Mais j'ai une amie qui me raconte quand elle y va, ben. En plus, mais c'est déprimant ! Mais quelle horreur *(rires)* ! Cette amie quand elle y va je peux vous dire que *(soupir)*, quand je la vois comme ça, je lui dis : « dis donc, ça va pas ? » ; « non, non ». C'est, *(soupir)*, ça, ça, pouf ça, ça fait mal aussi ! Quand c'est des amies en plus ! Donc je me, je crois que j'aurais été encore plus déprimée qu'elle *(rires)*.

**C : D'aller dans, dans ce genre de groupes ?**

**L :** Oui. Je crois qu'il vaut mieux aller boire des verres avec les copains dans ce cas-là *(rires)*.

**C : Faire des, d'autres astuces ou d'autres, d'autres choses à disposition que.**

**L :** Mais enfin, ça aurait pu, peut-être hein, elle a essayé !

**C : Et, de quoi d'autre, peut-être, auriez-vous eu besoin, lors de ces visites ou, ou que vous auriez besoin aujourd'hui ?**

**L :** Je crois que la, la seule chose vraiment, nécessaire et que je fais pas, c'est cette histoire de physio et d'exercices. Et là je, et puis je, je suis la seule, c'est, c'est, c'est sur moi que ça repose, je veux dire ! C'est pas comme un enfant où on lui dit : « maintenant tu t'habilles, tu viens avec moi puis on sort ! » Là, c'est à moi de me prendre en main ! Parce que les copines elles, elles, y en a, je crois qu'y en a une, je lui dis : « même tous les jours ? Même quand t'en n'as pas envie ? C'est vrai ? Tu sortirais avec moi ? » ; « Oui ! ». Bon, dans la pratique je sais qu'elle pourrait pas. Mais ça fait rien, c'est égal, c'est déjà beaucoup, même si elle faisait que la moitié de ça. Mais je, bon là je, je suis trop fière ou je me dis : « j'y arriverai toute seule ». Puis en fait je suis là, puis tout d'un coup y a des, j'allume l'ordinateur, y a pleins de trucs passionnants. Puis tout d'un coup c'est midi, puis je suis pas sortie. Ou, ou des choses de ce genre !

**C : Ce serait plus, ce que vous auriez besoin c'est ces exercices, [plus physiques.**

**L :** A mon avis c'est, c'est ça] le plus.

**C : Réussir à, [à se motiver à aller faire.**

**L :** Oui, oui.]

**C : Donc vous avez aussi ces amies, qui vous proposent de faire des xxx ?**

**L :** Oui alors j'ai une ou deux amies qui marchent vraiment, partent pour marcher, une qui habite juste à côté. Mais elle va pas marcher tous les jours à la même heure. D'ailleurs je, je sais pas, tout le monde me dit aussi : « y a que ça, le groupe et l'horaire ». Et moi c'est tout ce que je déteste. Donc c'est à moi de savoir ce que je veux aussi. Est-ce que je veux aller mieux ou bien, ou est-ce que je veux faire ce que je veux. C'est comme la cigarette : « oh mon dieu c'est tellement bon, bon je continue à fumer ». Puis je suis en train de me détruire, donc je choisis, c'est, c'est moi qui le fais, personne, c'est pas quelqu'un d'autre qui fume à ma place. Mais là ça a été, ça a été vraiment. Mais, on peut avoir quand même de l'aide ! Et là c'est mon pneumologue qui, parce que, bon à force de dire : « ouais j'ai quand même fumé. Oh c'était seulement trois ou quatre mais c'est encore trop ». Je sais pas tout d'un coup il s'est mis à parler, il arrêtait plus ! Je disais : « mon dieu, je suis sûre qu'y a au moins 50 personnes qui sont dans la salle d'attente » *(rires).* Et puis, je sais pas, je me suis sentie mieux, j'ai, j'ai eu l'impression, je sais pas ce qu'il m'a dit, il a, il a parlé autrement que les autres pour dire la même chose hein : « vous êtes en train de, vous allez vers la mort ». Enfin des, des choses comme ça. Mais, je sais pas, ça m'a plu. Bon, c'est pas acquis, ça fait maintenant peut-être trois mois, c'est un exploit, j'ai jamais arrêté aussi longtemps.

**C : [Là ça fait trois mois que vous arrêter de fumer.**

**L :** Oui. Puis y a eu X *(hôpital)* entre-deux]. Parce que quand j'ai été hospitalisée en 2015, j'ai arrêté pendant quatre mois, puis j'ai recommencé une fois que j'ai été ici, de nouveau dans mes petites habitudes. Alors là, la cigarette, non là *Julie (infirmière de recherche)* elle a pas, ou elle a peut-être pas voulu, c'est moi qui ai pas voulu. En tout cas là on n'a pas, je crois pas qu'on n'a vraiment… Je sais plus si on traitait le sujet… Mais le fait qu'elle vienne souvent, c'était, ça aussi c'est, c'est pas mal. Mais alors là ça, évidemment c'est, c'est le côté utilitaire pour moi, hein, c'est pas le côté de l'enquête. Mais si elle était venue moins souvent, ce qu'elle me disait ça pouvait très vite, être oublié. Tandis que là, j'avais pas le temps de, de ne plus faire ou d'oublier ce qu'elle m'avait proposé. Comme justement la physio, elle me disait : « mais, allez-y quand même parce que, vous, vous, quand vous le faites c'est un plaisir ». C'est un plaisir jusqu'à ce que ça devienne insupportable, c'est

vrai. Mais, après on se sent mieux. Et puis c'est pas, c'est pas le bout du monde, en scooter je, je mets trois minutes jusqu'à l'hôpital ! J'ai pas vraiment beaucoup d'excuses, hein. Et, donc elle, elle me donnait comme ça des petites, des petits tuyaux pour, pour me donner envie. Et le fait qu'elle revienne le mois d'après, ça prolongeait. Mais ça a pas suffit donc pour que je continue *(rires)*.

**C : Mais, mais c'était bien que ce soit assez fréquent ces visites.**

**L :** C'est pas mal comme rythme, oui.

**C : Vous aviez le, le temps de poser vos questions et. Ça, ça vous a plutôt plu ces, cette fréquence-là une fois par mois ?**

**L :** Ben oui, contrairement à ce que je pensais c'était pas, oui je, c'était bien.

**C : Et vous avez parlé un peu de, de vos amis. Quel type de soutien vous avez de la, de la part de vos proches ?**

**L :** Oui, sûrement… Mais toute façon là je, je fais que, je fais que me, on peut me dire ce qu'on veut, je fais quand même comme je veux, je veux dire. On a parlé aussi, elle m'a, on a parlé de ma fille aussi parce que ma fille ça peut être un soutien très important. Et elle a, elle a une sensibilité aussi très… ouais très, très pointue. Elle, elle, elle comprend bien le, les autres et puis elle s'est beaucoup renseignée aussi sur les mécanismes de la dépendance, et puis le. C'est pas, pas comme une fille, plutôt comme une amie, elle a, elle a fait ça parce qu'elle m'aime, et que ça lui est pas indifférent que, que je, que je sois, elle a, elle a envie que je sois en bonne santé, mais pas comme : « oh maman je t'aime tellement ! ». Pas du tout, au contraire. Des fois je lui dis : « eh mais, t'entends comme tu me parles ?! » *(rires)*. Alors là *Julie (infirmière de recherche)*, de temps en temps elle me disait : « vous pourriez demander à votre fille ? » Mais bon, X *(prénom)* ma fille c'est, c'est, non, on peut rien lui demander. C'est elle qui décide quand, comment et puis c'est, c'est, ça peut pas être un, enfin ça pourrait être un soutien parce que l'autre jour elle m'a dit que elle allait marcher avec son père, je crois tous les jours ! Je tombais des nues ! Ben elle a fait ça justement ! Elle lui donnait rendez-vous en ville, là où elle était, donc il devait faire la, il devait se démerder, tel jour à telle heure, et j'ai compris qu'elle l'avait fait tous les jours pendant quelques temps. Parce que lui il faisait de la physio, à X *(hôpital de réhabilitation)* justement. Il y allait presque tous les jours, pour le coeur. Et, il a arrêté. Alors elle a gueulé. Et puis elle lui a proposé ça, alors je sais pas pendant combien de temps, xxx. Ça c'est des choses qu'elle est capable de faire. Mais moi je voulais pas lui demander parce que je culpabiliserais trop en me disant : « oui mais elle a pas le temps » *(rires)*. Je vais rechercher de l'eau.

**C : *(rires)* Oui prenez prenez !**

*(L va chercher à boire)*.

**L :** Je sais pas si ça vous satisfait comme réponses ?

**C : Oui on a, on a déjà bien, pu aborder beaucoup de choses, déjà, oui !**

**L :** C'est vrai ? Je me rends pas compte !

**C : *(rires)* Non, vous avez déjà pu me donner beaucoup, beaucoup d'informations. Donc, et, si on regarde, ben vous me disiez par rapport à vos proches. Donc vous avez votre fille, mais vous voulez pas trop la faire culpabiliser, enfin vous culpabilisez de lui demander de l'aide.**

**L :** Ben oui ça me gêne, parce qu'elle est, elle est hyper occupée tout le temps. Alors moi, la seule chose que je fais, d'ailleurs je fais comme ça avec tout le monde. C'est : « si t'as envie de me voir tu te manifestes. Je te donne le, je te donne le mode d'emploi, puis après tu l'utilises ou tu l'utilises pas ». Et là je lui dis : « dis donc ça fait longtemps qu'on n'est pas allé manger ensemble ! Tu sais moi je viens où tu veux, même une heure si tu veux ! ». Parce qu'elle peut, elle s'imaginait un temps que quand on va au restaurant faut, faut compter au moins deux heures, enfin. Puis j'ai dit : « mais moi ça me fait plaisir, même une heure ! ». Alors des fois elle relève la proposition mais, je dois dire depuis une année, on s'est pas beaucoup vues. Mais là on se verra plus parce que, dans, dans quelques heures ou quelques jours je serai grand-maman. Alors là je sais qu'on se verra plus *(rires)*. Ben,

dimanche, pour la première fois on a passé toute la journée, tout l'après-midi, depuis midi jusqu'à 18 heures ensemble. C'était pas arrivé depuis des années *(rires)* !

**C : Ouais. Donc là vous allez être grand-maman, prochainement.**

**L :** Ouais, ouais, c'est dingue. Alors là je sais que, elle va apprécier ma présence. Mais là, ça justement ça me, ça me désole, peut-être là *Julie (infirmière de recherche)* elle aurait pu me donner des tuyaux, c'est que je dis à X *(prénom de sa fille)* : « mais ce bébé je vais même pas oser le porter ! Puis je pourrai pas aller le promener ! Bon je peux le porter pendant qu'il est tout léger, puis après ? Je pourrai ». J'ai dit : « ben ma foi je lui raconterai des histoires ! ». Ça, je dois dire que ça me, ça, ça me tombe sur le moral, puis en même temps ça me stimule pas assez pour. Voyez, parce que la physio j'aurais déjà, j'aurais dû l'appeler déjà lundi, je l'ai pas fait, puis on est mercredi.

**C : Ça vous tombe un peu sur le moral avec ce petit qui va arriver.**

**L :** Ah oui. Oui, puis donc j'ai envie d'être en forme pour ce bébé, puis en même temps voyez j'ai pas téléphoné pour la physio. Donc, les amis je peux les solliciter mais, je fais une fois, deux fois, et puis après, non.

**C : Comme vous disiez, ils ont le mode d'emploi et eux peuvent vous contacter aussi.**

**L :** Alors c'est vrai que y en a une qui va tous les jours en principe boire le café juste là à côté au bistrot avec son mari. Et puis, de temps en temps bon, elle y va tôt, c'est à 14 heures, moi j'aime pas sortir si tôt ça, enfin j'aime pas couper la journée. Mais enfin je sais que je peux la trouver là. Mais les rares fois où j'y suis allée, elle y était pas. Et puis justement là, je voulais lui proposer quand son mari rentre, que nous deux on aille marcher, mais elle est toujours occupée ! Alors, c'est ambigu !

**C : Oui. Les gens sont quand même assez occupés. C'est pas facile.**

**L :** Bon moi mes amis sont spécialement occupés *(rires)*. Mais c'est aussi moi qui pourrais, je sais pas je dois être trop difficile ou trop exigeante. Mais, si je, si je fais pas ça, ça veut dire je devrais le faire par moi-même.

**C : Les contacter ?**

**L :** Non, mais aller marcher toute seule ! Ça me dérange pas d'aller toute seule. Je fais beaucoup de choses toute seule...

**C : De temps en temps, ces amis qui sont là.**

**L :** Oui ! …

**C : Mais, mais merci vous m'avez déjà, dit pas mal de, de choses. Si, si je résume un peu. Donc vous aviez participé à l'étude, plus pour, pour la science, pour faire avancer les choses, moins, moins pour vous au départ.**

**L :** Au départ, oui.

**C : Ouais. Et, vous avez vu que ça vous a quand même apporté des choses d'avoir ces visites, avec *Julie (infirmière de recherche)*, où vous aviez eu un bon contact. Et vous avez pu poser pas mal de questions sur la maladie ou d'autres choses autour de, de la maladie. Et, c'était aussi un moment où vous avez pu avoir un peu des rappels sur certaines informations que vous aviez reçues, [à l'hôpital.**

**L :** Oui.]

**C : Aussi vous me disiez par rapport aux médecins aussi on n'a pas peut-être pas le temps d'aborder certaines choses. Là c'était des moments où vous aviez le temps de, de parler un peu plus de choses. Et en plus de tout ce qui était gestion de, des symptômes ou des médicaments, y avait aussi ce soutien moral, vous pouviez parler un peu de, de ça également.**

**Donc, de manière générale, c'est plutôt quelque chose que vous appréciez ces, ces visites des, des infirmières. C'était aussi un moment où vous avez pu parler des directives anticipées. Donc ça, ça vous avait un peu soulagé de pouvoir avoir ces choses qui sont écrites par rapport aux soins en fin de vie. Et, et voilà de manière générale plutôt quelque chose qui, qui vous a plu, ces visites, aussi la fréquence, que c'était assez fréquent pour pouvoir se souvenir des, des choses de séances en séances et de pouvoir aussi avoir quelqu'un qui vous motive pour participer soit à la, à la physio ou ces activités physiques.**

**L :** Ouais c'était un petit peu, d'abord, bon c'était un soutien. C'était un, un, un éclairage, un autre regard… Elle, un prolongement un petit peu de ce que je, ce qui s'ébauchait chez moi, elle, elle arrivait à le prolonger et éventuellement à le concrétiser. Et puis alors une chose qu'il faut que j'ajoute c'est que, moi je, bon j'avais pas de préjugés hein, mais enfin pour moi une enquêtrice, c'est pas un médecin. Mais j'étais épatée par ses connaissances, mais elle savait <u>tout</u> ! J'étais, à un moment donné j'ai dit : « mais vous en savez plus que mon médecin, ma parole ! ». Ah oui alors le, le niveau de compétences remarquable, oui.

**C : Aussi ces compétences-là que vous apprécié, [les connaissances.**

**L :** Oui, oui.]

**C : Sur la maladie.**

**L :** Ah oui parce qu'alors moi, je suis très, oui bon comme mon pneumologue. Mais maintenant avec l'âge, je suis devenue beaucoup plus critique, plus exigeante, et j'ose des choses, dire des choses ou faire des choses que j'aurais jamais osé avant. Et, et si, si un, si j'estime que mon médecin est, il répond pas à ma question, j'<u>ose</u> lui dire : « vous avez pas répondu à ma question ! ». Mais c'est pas évident !

**C : Oui. Faut oser.**

**L :** Et, mais là alors, alors là justement j'étais à l'aise, puisqu'elle était là pour moi ! C'est ça que, j'avais, voilà j'avais pas l'impression d'être au service de l'enquête, ça veut pas dire que, j'ai pas perdu de vue, que, qu'on était là pour ça ! Mais c'était pas ça qui était présent dans ces moments-là, c'était qu'elle était à, à mon service, entre guillemets service, hein. Et qu'elle était là pour moi et plus que moi pour, pour l'enquête. Voilà ce que je dirais pour résumer en positif.

**C : Ouais. D'avoir ces, ces moments seuls où si on est que deux.**

**L :** Oui !

**C : Pouvoir partager.**

**L :** C'est pas, c'est pas un psychologue, c'est pas un médecin, c'est pas, c'est pas une copine, c'est, c'est, c'est quelque chose d'à part.

**C : Quelqu'un un petit peu en dehors de, de tout ça.**

**L :** Oui.

**C : Je sais pas si vous aimeriez encore rajouter autre chose ?**

**L :** Ben non, là je crois que vraiment, là c'était le mot de la fin *(rires)*.

**C :** ***(rires)*** **Mais merci en tout cas.**

**L :** Sauf si vous avez des questions bien sûr.

**C : Non mais je crois qu'on a pu bien faire le, le tour. Je vous remercie d'avoir donné toutes ces informations sur, sur l'étude. On peut arrêter là.**

**L :** D’accord.

# Participant n°15

| |
|---|
| Nom d'emprunt : Rose |
| Âge : 70 ans |
| Sexe : F |
| Groupe : Intervention |

**C : Donc si je reprends, donc vous avez participé à une étude qui avait comme objectif de comparer le traitement habituel de votre maladie pulmonaire la BPCO, avec une prise en charge précoce, soutenue et intégrée. Vous étiez dans le groupe bénéficiant de cette prise en charge globale spécialisée. Et vous avez reçu la visite d'une infirmière une fois par mois pendant un an. Pouvez-vous me raconter comment ça s'est passé ?**

**R :** Très bien, très bien. On a bien parlé, on a, on a parlé de la santé, comment j'allais, le moral. Elle m'a bien soutenue moralement. Pourquoi normalement c'était un moment très, très difficile pour moi. Ça faisait pas longtemps que j'avais sorti de l'hôpital avec une thyroïde un petit peu, pas tellement jolie. Et puis, elle, elle m'a apporté pas mal de choses dans ma vie. Et puis avec elle j'étais vraiment bien, c'était une dame formidable. Elle était bien à l'écoute, en disant qu'est-ce que moi je disais, non, j'étais très, très contente. Et ça m'a bien apporté, ça m'a apporté si vous voulez un petit peu, aller en avant, aller en avant pour ma maladie, que normalement ça fait des années que je la, que je la, je la tiens. Seulement là, à ce moment-là, c'était un petit plus difficile pour moi parce que j'étais tombée malade très, très gravement. J'étais aux trucs intensifs. J'étais plus que de l'autre côté que d'ici si vous voulez. Mais ben à l'hôpital, elle était très, très, très, très efficace, très, très formidable les médecins, et tout. Et puis, mais cette infirmière vraiment elle m'a, elle m'a bien, elle m'a bien aidé pendant une année, oui. Cette année ça a été un peu plus que difficile.

**C : Cette année.**

**R :** Oui, c'était moralement, physiquement, mais physiquement aussi. C'est la thyroïde, elle m'aide pas beaucoup, elle m'aide pas beaucoup, je suis très fatiguée, le moral il va pas. Que normalement je suis une femme très, très joyeuse. C'est moi qui apporte aux autres le, le, si vous voulez le, la joie, le… On dirait que moi avec mon, mon énergie c'est moi qui prends le, les, les soucis des autres. Mais cette année non, cette année ça a été très, très, c'est très, très difficile. Et j'espère qu'il finit vite, cette année ! J'ai, j'ai été très malheureuse, c'est la thyroïde, elle m'a pas aidé, elle m'a pas aidé du tout cette thyroïde. Je suis vraiment très inquiète, bon là on est bientôt à la fin. On est en train de faire des examens justement pour, avec la doctoresse de l'hôpital, je me rappelle plus comment elle s'appelle. Et puis aussi une femme très, très gentille, elle m'explique bien les choses et tout. Mais, pour la fin de l'année on va voir de, ou de m'opérer, ça c'est une chose que je voudrais bien, quand je sais que c'est la thyroïde qui me détruit comme ça. Moralement, en tout cas moralement. Puis voilà, j'ai toujours eu mon oxygène normalement, pas de problèmes oxygène, oxygène. Maintenant avec cette thyroïde depuis l'année passée, le mois de mars l'année passée, j'ai, j'ai plus assez d'oxygène. Et ça aussi ça me travaille énormément. Je supporte pas de sortir avec cette bonbonne sur moi. Et je xxx, jusqu'à hier je me sentais pas malade. J'avais ma maladie, mais je me sentais pas malade. C'est pourquoi, on le voyait pas. En disant d'une manière, moi je le savais, mais peu de gens le savaient. Mais avec l'oxygène, que je dois, *(quelqu'un sonne à la porte)* je me, je me, je me sens vraiment, je me sens pas bien, je me sens, non, pas du tout.

**C : Ouais, ouais.**

*(R ouvre la porte et parle avec la personne à la porte)*

**R :** Bon l'oxygène je le mets en cas de besoin, ça c'est vrai. Mais j'avais remarqué je voulais pas le mettre dehors, le regard des gens ça me, ça me gênait un petit peu. Mais ça fait une semaine là, que j'ai plus le courage de le mettre. C'est vrai que ça me gêne, quand je marche avec les yeux bas, c'est une chose que, en tout cas dans ma vie j'ai toujours été une femme très battante, à tous mes problèmes, j'ai toujours essayé de, d'aller en avant. Et avec ma maladie je, j'ai fait la même chose que c'est de l'année 2000 que je suis très, très malade, mais plus gravement là disons. Ils ont découvert ce BPCO. Et puis, puis voilà. Je marche avec la tête baissée, ça me, ça, ça m'embête un petit peu que les gens ils savent que, que, que madame X *(nom)* voilà, elle est très malade et puis qu'elle a de l'oxygène, c'est un petit peu un orgueil, je sais pas. Et ça, ça m'aide pas du tout ! Je suis vraiment pas bien !

**C : C'est, c'est difficile le regard des autres.**

**R :** Le regard des autres et, oui, pourquoi j'étais toujours une femme, malgré avoir, d'avoir mes problèmes, j'ai toujours essayé de le, de les résoudre, avec ma façon de faire, avec ma façon de, d'aller en avant, voyez, c'est ça. Et puis voilà. Donc, *(soupir)* je dois vivr', je dois bien me, je dois bien vivre, en tout cas j'ai cinq petits-enfants. J'ai trois filles qui m'adorent. Je dois quand même aller en avant mais j'y vais pas vraiment bien en avant.

**C : Ouais, c'est difficile.**

**R :** C'était mieux l'année passée que, que cette année. L'année passée, j'avais encore l'oxygène. Cette année, non, ça a été vraiment, je suis entrée à l'hôpital, cette année, de nouveau que j'ai fait deux mois d'hôpital, toujours pour la thyroïde, hein. Ça a été plus difficile et puis l'oxygène il baisse peu à peu, c'est ça que c'est le problème, c'est, je comprends <u>pas</u> pourquoi ! Donc moi je, je pense que c'est la thyroïde qui m'embête. J'ai dit à la doctoresse que je voulais m'opér', faire des choses le plus vite possible, que moi je suis très, très fatiguée. Je dors tout le temps ! J'ai jamais dormi autant, moi je suis pas une grande dormeuse, hein. Moi cinq heures, six heures par jou', par nuit, ça me suffit. Demain je suis en pleine bombe ! Mais là vraiment je suis, là je me suis réveillée à 8h45, je me suis couchée à 23 heures. Mais je me mettrais sur le canapé, je dormirais de nouveau.

**C : Vous êtes fatiguée.**

**R :** Ouais, je suis très, très fatiguée ! Donc, voilà c'est ça. Mon problème, mon problème plus, important c'est, ce serait cet oxygène qui me, qui me fracasse. Je le mets, je vais à l'hôpital, je vais à la gym. Mais, je suis pas aussi heureuse, pas vraiment heureuse.

**C : C'est plutôt le moral.**

**R :** Non, non, là je suis pas bien. Là je, je, je, encore j'ai mon petit-fils qui va venir manger à la maison, je suis contente quand j'associe avec lui. Puis, il me dit : « nonna, ça va ? Qu'est-ce qu'il y a nonna ? » Puis je dis : « mais rien nonna, je suis fatiguée ». Puis, ça me, ça m'aide un petit peu à surmonter la journée.

**C : D'avoir votre petit-fils qui vient.**

**R :** Ouais. Il va venir aujourd'hui, il a 16 ans. Et puis il vient deux fois par semaine. Bon les autres, ils ont xxx plus loin. Et puis l'autre il est aussi à côté, mais il vient pas à midi à manger à la maison, donc je le vois que le mercredi. Mercredi, je vois tout le monde. Je vois toute la famille, le mercredi, je fais à manger pour tout le monde, après eux ils m'aident. Là je me suis acheté un lave-vaisselle comme ça, j'ai dit si je veux continuer à avoir mes petits-enfants et mes filles à la maison, que moi j'aime bien la table, j'aime bien discuter, et tout. Alors j'ai dit il faut que je m'achète un lave-vaisselle. Et là j'ai pris la décision, je me suis acheté un lave-vaisselle, comme ça le soir je mets tout dedans, et puis après je suis tranquille. Voilà, on essaie d'être bien sans l'être bien. Mais je vous répète, cette oxygène elle m'aide pas du tout, elle est en train de me détruire.

**C : D'avoir l'oxygène.**

**R :** A la maison ça me fait rien. A la maison, je le mets quand j'ai besoin. Mais dehors c'est, c'est vraiment catastrophue.

**C : Que, que les autres [voient que vous avez ?**

**R :** Ouais, ouais, surtout] le quartier où je suis, où j'ai été. Les autres quartiers ça me fait rien, hier j'ai été chez, en X *(quartier)* avec ma fille, ça m'a rien fait le regard des gens, parce que c'est des gens que je connais pas, voyez, ils me connaissent pas. Donc ça m'a rien fait, puis j'étais avec ma fille, voyez, c'était pas la même chose. Mais si je dois sortir à X *(quartier)* ou aux X *(quartier)* où j'ai habité pendant, pendant 28 ans, ça m'embête un petit peu, tous les regards de, des amis. Il dit : « oh la pauvre, la pauvre ! ». J'aime pas trop ça, mais bon.

**C : Les gens ne savent pas ?**

**R :** Non. Bon, c'est normal moi aussi je regarde si quelqu'un il est handicap ou, moi aussi je. Des fois j'essaie de, de me mettre à la place d'un handicapé, de dire : « voilà, celui-là il est comme moi, alors pourquoi l'autre ? ». Je sais pas, je dis : « il faut te donner du courage, y a pas seulement toi ! ». J'essaie de, de, de me mettre à la place des autres, pour être bien. C'est vrai que des fois ça me donne du courage, mais c'est la, c'est un petit orgueil, c'est un orgueil qui me détruit là. Par contre je suis toujours, mon orgueil je l'ai toujours utilisé pour des belles choses, jamais pour les mauvais. Jamais de dire, pour l'orgueil : « non, je m'en fous je me, je vais me détruire », non ! Mon orgueil il, il m'aide à m'en sortir. Mais là il veut pas, là il veut pas. Je me bats, vous savez que je me bats, hein ! Je me bats, j'essaie de le mettre, j'essaie de sortir, j'essaie de faire la forte, j'essaie, mais je marche avec le, on dirait je le hais ! Je le hais, xxx *(mot dans sa langue natale)* !

**C : Le, l'oxygène. Oui, c'est difficile.**

**R :** C'est ça *(soupir)* !

**C : Mais là vous avez vos petits-enfants donc, et vos filles aussi, vos enfants qui sont là, pour vous ?**

**R :** Oui, ils sont là, ils m'aident à : « mais c'est rien maman, mais c'est rien ! Mais t'es là, t'es avec nous et tout ça ! » Mais *(pleurs)*.

**C : Oui. C'est difficile.**

**R :** Excusez-moi.

**C : Non, non. Tenez un mouchoir. C'est, c'est difficile de devoir sortir avec, et, oui.**

**R :** En tout cas moi, ça me. Moi j'avais ma copine qu'elle était aussi, elle avait l'oxygène, elle avait aussi, *(toussotement)* elle a commencé à avoir l'oxygène presque à mon âge. Parce que moi je l'ai connue à 75 ans. Je l'ai connue, oui, à 75, je crois. Non, 72, elle avait 72 ans ! Elle avait déjà l'oxygène. Mais, mais elle le portait bien, elle était brave, elle était formidable, maintenant elle est plus là, elle est morte. Elle m'écoutera de l'autre côté. Et puis, elle était brave, elle était courageuse, elle le portait sans, sans problèmes. Des fois moi j'étais à côté, et je voyais les gens qui la regardaient, les enfants et tout. Je me gênais moi pour elle. Puis elle marchait avec une fierté. Bon elle était une femme assez, elle s'en foutait un petit des autres. Moi non, moi je m'en fous pas du tout, c'est ça, on n'a pas le même caractère. Et voilà, et des fois je pense à elle, j'essaie de penser à elle, comme elle était forte de porter ce truc sur l'épaule, après elle le portait. Moi j'ai trouvé un autre système plus élégante, vous savez je le mets dans mon sac. Je mets sur mon dos et puis quand ça, ils disent : « mais elle porte quoi cette femme ! » *(rires)*. Oui, je suis un petit plus coquine, ou coquette je veux dire, coquette *(rires)*. Oui.

**C : Coquette oui *(rires)*. Oui. Donc là vous le mettez dans le sac à dos.**

**R :** Oui, oui, j'ai un belle sac, ma fille elle m'avait fait cadeau, et il va tout juste ! Je le mets sur le dos là, y a juste le fil qui pend. Mais, voilà, c'est, c'est le seul problème que j'ai avec ma maladie. Autrement ma maladie ça va et, je fais mes sprays, je, ça va. Ma respiration elle a, elle a bien repris un petit peu. En tout cas moi j'ai, j'ai beaucoup d'appareils, à la maison. J'ai, pour prendre, combien d'air vous avez dans les poumons, pour souffler. J'étais à, à 75, j'étais arrivée. Et puis, bon faire des exercices, la gym et tout ça, le, le, le, le truc là, j'ai réussi à en arriver à 180. Alors je sens, je sens que je vais mieux quand même avec la respiration. Mais faut qu'encore je travaille beaucoup. Bon je fais quand même la gym trois fois par semaine à X *(hôpital)*. Ils sont tous très gentils, les inf', les, les dames là qui sont X *(prénom)* et puis madame, elles sont formidables. Ça fait des années que je les connais donc. Et puis, puis voilà. Je veux dire autour de moi, tout va bien mais, non, c'est l'oxygène qui m'embête, y a rien à faire. Alors j'ai toujours cet espoir, cet, qu'elle me fait aller en avant, c'est que moi en me, m'enlevant la, la, la thyroïde, je vais tout reprendre normalement à vivre normalement. Et j'ai tellement de la force de, de, de tellement que j'y crois à ça, moi xxx comme que je le ressens, tout ça. Je sens que c'est la thyroïde qui m'embête, qui m'embête pour beaucoup de choses ! Des choses que j'ai jamais eu dans ma vie, la fatigue, j'ai jamais pensé la fatigue, quand on est fatigué, quand on est fatigué, ah on est fatigué de, de travail, mais pas fatigué, vous avez pas de forces, vous avez pas, vous vous sentez pas bien. Et puis voilà, et puis après, vous vous couchez sur le canapé, vous vous endormez, quand vous êtes pas bien, voilà. Donc c'est pour ça je pense que c'est la thyroïde qu'elle m'embête un petit peu. Et j'ai encore la force d'aller en avant, en

espérant que quand je vais m'opérer, on va me la détruire, avec le diode. Moi je reprendrais l'oxygène comme avant, voilà. *(sonnerie de téléphone)*. C'est mon concierge. *(conversation téléphonique)*. Il me descend le meuble-là. Voyez mon petit-fils il est pas venu manger, et le meuble il est resté là. Et puis voilà, voilà c'est tout. J'ai pas grand-chose, je suis une grand-mère et maman heureuse. Je remercie le bon Dieu que il m'a donné quand même ça. Ils sont toujours là, ils sont toujours avec moi, on me téléphone tout le temps. Ils sont vraiment braves, non, je suis entourée d'amour, ça c'est, ça c'est exclu, je ne peux rien dire. Mais, bon ma maladie elle joue là-dedans, mais je la, je l'ai bien supportée, je la supporte, je sais que je suis malade. Mais avec cette maladie je peux marcher, et sans l'oxygène je ne peux pas vivre. Donc voilà, et cette oxygène elle me, il est en train de me détruire un petit peu. Alors je vais voir maintenant, qu'est-ce qu'on va faire l'année prochaine. Alors, si on m'opère, si, ils ont, ils ont peur de m'opérer à cause de mes, ma respiration, ils ont peur qu'après en m'enlevant le tuyau, j'arrive plus à respirer. Mais mon pneumologue il avait dit que y avait pas de risques. Autrement ils vont m'enfermer dans cette chambre mais, ça peut marcher bien, ça peut pas marcher, ils sont pas sûrs non plus. Que l'opération, ils sont sûrs, mais à risque.

**C : Pour la thyroïde.**

**R :** Donc, mais, moi d'un côté, vous savez je suis quelqu'un je ressens un petit les choses hein, moi sur moi-même, hein. Je voudrais quand même prendre la déc', j'ai dit : « j'ai fait ma vie. J'ai vu mes petits-enfants grandir. Y en a qu'il a 18 ans. La plus petite, elle a 12 ans. Mes enfants ils sont bien, ils sont casés, ils sont mariés, ils ont les enfants et tout ». J'ai dit : « il faut que je prends ce risque, pour moi-même ». Parce que moi j'ai dit : « en m'enlevant cette thyroïde, je ne peux, je peux vivre mieux. Et sûre, que je l'ai plus ! Que en allant dans cette chambre, on est pas sûr de qu'est-ce que ça peut arriver. Y en a que ça détruit, y en a que ça détruit pas ». Ma belle-soeur elle a fait, elle a dû faire la chambre, plus que l'opération. Donc, si je dois faire la chambre, plus attendre pour l'opération, moi ça me convient pas du tout, que moi j'en ai marre. Et qu'est-ce que je vais lui dire à doctoresse, à l'hôpital, quand je la vois ? J'ai dit : « moi j'ai pris la décision. Je veux signer, mai je veux m'opérer, c'est tout »: C'est la chose que, la dernière fois quand ils nous ont mis le, le, le, le, le tuyau, comment on dit le tuyau, pour m'endormir, ils ont un petit peu touché mes, mes cordes vocales et puis mes, mes muscles. Et puis quand je mangeais, ça allait tout à travers, ça allait dans les poumons. J'ai un petit peu peur ça, ça j'ai peur. Mais, ça c'est quand même pour moi, comme j'ai déjà vécu ça, pour moi c'est pas grave, pourquoi j'ai guéri ! On m'a mis le, le truc dans le nez, j'ai essayé de manger pendant un mois avec ce truc xxx, jusque ça, ça me fasse, ça fasse bien la gorge. Puis là aujourd'hui je suis bien, je mange tout, je suis en pleine forme, j'ai pas de problèmes à la gorge ! Donc voilà, c'est ça.

**C : Vous êtes prête à prendre ce risque [de l'opération.**

**R :** Ouais,] ouais. Pourquoi je sais qu'avec, sans la thyroïde, parce qu'elle est assez grande, hein. Ils nous on fait des analyses au dernier moment. Et puis, j'étais au truc nucléaire. Et puis, j'ai dû prendre, j'ai voulu avaler des pastilles nucléaires, pour l'avoir mieux, cette pastille. Et depuis là que je suis vraiment, vraiment pas tellement bien aussi hein, depuis quand j'ai avalé ces deux pastilles, je suis vraiment, vraiment là, là l'oxygène elle a vraiment, parce que moi je l'avais toujours 94, 95. Seulement avec les efforts, il part. Mais là, même en étant assis, elle est 91, 90, 92, il baisse toujours. Donc cette pastille, ces pas', je pense que ces pastilles, ils nous ont pas fait du bien, pour les, parce que normalement c'est des pastilles qui vont dans le sang. Ils vont dans le sang et puis, après ils vont vers la pastille, vers la, la gorge et puis pour voir. Et c'est là qu'ils nous on dit : « ouais, elle est assez grande, y a rien de grave », il avait dit le jeune homme, « mais elle est assez grande. Elle a bien, elle a beaucoup évolué ». Donc voilà. Là j'ai encore un examen le 20, et puis après je dois voir le chirurgien, juste si en cas d'opération. Et je pense que pour l'année prochain, ça va être fait. C'est toujours, j'ai toujours arrivé à être malade, mais le mois de mars, le mois d'avril, le mois de février, toujours, ouais.

**C : Donc là vous, vous attendez pour [ce rendez-vous là, oui. Et.**

**R :** *(toux)* Ouais.] J'attends ça et puis on va voir.

**C : Oui. Et vous disiez aussi, pour ces visites des infirmières, qu'elles ont pu vous remonter un peu le moral ?**

**R :** Oui *(raclement de la gorge)*. Justement *(raclement de la gorge)* pardon, justement cette dame, cette infirmière, on parlait de tout, hein. Et puis, c'est vrai que, que elle m'apportait, elle m'apportait beaucoup

de choses. Elle m'apportait un petit peu de sérénité. Bon j'étais pas, j'étais assez, moi j'étais bien aussi, hein, l'année passée, je vous parle de l'année passée. J'étais bien aussi l'année passée, j'étais bien, j'étais sortie de l'hôpital, j'avais, j'avais quand même passé un, un moment très, très grave. Et, parce que j'étais vraiment très, très, très, très grave ! Et puis, je me suis en sortie. Même les médecins à l'hôpital, ils disaient, le pneumologue il disait : « c'est une femme très, très forte, elle va s'en sortir ». Ils disaient tous comme ça : « bravo madame X *(nom)*, vous êtes très, très forte », ils disaient. C'était vraiment, j'ai eu un, j'étais en pneumologie, au 7ème étage, je suis restée un mois chez eux, mais des, des personnes formidables, braves, des infirmières. On rigolait, on disait des, des bêtises, je draguais avec les petits monsieurs, les petits jeunes hommes, que c'était mes petits enfants hein, si vous voulez. Y avait un qui s'appelait X *(prénom)*, il était vraiment chou comme tout. Ça a été vraiment super ! Ça a été vraiment, et c'est grâce à eux, et grâce à mes enfants, le soutien de mes enfants et de mes amis, que je me suis sortie de cette, l'année passée, parce que l'année passée c'était vraiment plus dur que cette année. Cette année, c'était souvent la thyroïde qu'elle m'a embêté. Mais c'était encore plus, ça a été encore plus grave encore, la thyroïde elle voulait pas se mettre en, en place, elle voulait pas se régler, avec les médicaments ! Quand on avait arrêté le mois de mars, pour justement, pour. Mais, le professeur à l'hôpital, il m'avait dit : « quand, quand, si vous vous sentez pas bien, vous venez vite à l'hôpital ». Mais c'est trop tard ! Qu'ils m'avaient fait arrêter les médicaments ! Justement pour aller dans cette chambre, pour le diode, pour le diode, je sais pas comment ça s'appelle. Et puis, mais malheureusement c'était trop tard. J'ai dû rentrer à l'hôpital, et puis y avait tout qui a bousculé, y a tout qui a, et puis voilà.

**C : Oui. Donc quand y a eu les visites des infirmières, c'était une période où vous étiez un peu mieux ?**

**R :** Oui, oui, oui, oui, non, non mais même, même quand j'ai, j'ai, j'ai rentré de, au truc dans, intensifs, ils étaient super, ils étaient tous autour de moi. Y avait une dame, elle s'appelait X *(prénom)*, une infirmière, une aide, une aide, une aide, mais elle était choue quand elle me lavait ! Elle était choue, mais, c'est, j'oublierai jamais ! Et puis, quand je suis sortie de l'hôpital, j'ai été les voir plusieurs fois. Alors le petit jeune homme, je lui ai apporté une bouteille de vin, et puis, pour lui dire merci pour sa gentillesse, parce que c'est lui qui me lavait hein, vous savez hein ! Moi j'ai, ça me gênait pas du tout alors ! Et puis, cette X *(prénom)* aussi elle venait, elle me lavait, elle me lavait, elle était choue comme tout ! Et puis après j'ai retourné, je lui ai apporté une pizza, je lui ai apporté des, j'ai toujours fait. Bon ça, maintenant ça fait un moment j'ai plus été, hein. Mais les premières temps j'allais souvent. J'ai été quatre, cinq fois j'ai été les voir, au septième étage, ouais.

**C : Vous avez.**

**R :** Ouais, ouais, non, j'ai, j'étais vraiment bien. Où j'étais bien aussi, c'était à X *(hôpital)*. J'étais à X *(hôpital)*, pour trois semaines. A X *(hôpital)*, on était bien, on était vraiment bien ! On avait un grand balcon, le lac juste en face, les infirmières ils étaient super, aussi ! On mangeait comme des rois ! Qu'à X *(hôpital cantonal)*, on mange pas bien du tout, je vous le dis très fort ! On mange pas bien ! Je regrette de le dire mais on mange pas bien ! Et puis, mais qu'à X *(hôpital)* non, à X *(hôpital)*, on mangeait bien ! On mangeait comme des rois ! Mais alors vraiment hein, on était dans un restaurant, hein ! Et là j'ai pris tout de suite deux kilos, j'ai pris *(rires)*. Et puis voilà. A X *(hôpital de réhabilitation)* aussi j'étais bien. J'ai resté un mois cette année au X *(hôpital de réhabilitation)*. Aussi ils étaient formidables, j'étais au troisième étage. Les infirmières, y avait des jeunes, des jeunes hommes là, des, c'était, ma' c'était la joie, c'était vraiment ! C'est, justement, j'ai resté là-bas pour régler cette, cette thyroïde qui voulait pas se régler ! Puis après à X *(hôpital),* j'ai été pour les poumons, pour ma respiration.

**C : Donc vous avez eu du bon soutien de la part des, [professionnels ?**

**R :** Ah oui, oui, non, non, oui, oui, oui, oui,] oui, oui, tout à fait !

**C : Et aussi de la part de la famille ?**

**R :** Oui, oui, oui, à X *(hôpital)* ils venait là-bas, des fois ils mangeaient avec moi ! En tout cas normalement on pouvait commander un repas, en plus, et puis on le payait bien sûr ! Sept euros, sept francs je crois, je me rappelle plus. Oui sept francs je crois que je payais. Et puis, puis mes, mes enfants ils mangeaient avec moi. Un jour on était tous là, on a pris une grande table ! On était tous ensemble ! Non, non, c'est, non, je vous dis que je suis, je suis une nonna et une maman très heureuse !

**C : Vous êtes bien entourée.**

**R :** Oui. Mais moi j'ai beaucoup donné aussi, hein. Vous savez, on a rien sans le, on a, on a, on nous donne rien sans le recevoir, on le donne rien, non on le, on reçoit rien sans le donner ! Bon, nos enfants c'est normal, c'est de l'amour, et vous le donnez. Mais des fois, vous savez, on, on n'arrive pas à le, on n'arrive pas à le montrer cet amour, avec nos propres enfants. Que moi oui, j'arrive. J'arrive, hier ma petite-fille, elle m'a descendu la poubelle, puis, les bouteilles. J'ai dit : « nonna te donne 20 francs », j'ai dit. « Mais c'était pas pour la poubelle, c'est parce que je t'aime ». Et je lui ai donné 20 francs. Elle a dit : « moi aussi nonna ! ». Elle m'a embrassé. Elle a 17 ans, cette fille. Elle s'appelle X *(prénom)*, elle est tellement belle, tellement choue. Non, ils sont tous beaux mes petits-enfants, c'est mes, mes, mes petits anges qui me tiennent dans la vie, c'est eux qui me, c'est eux qui me, qui me, qui me tiennent, qui me donnent cette force, d'aller en avant !

**C : [Les petits-enfants.**

**R :** Ouais, ouais,] ouais. Ils sont très attachés à moi. J'ai toujours, je m'en suis toujours occupée un petit peu. Que quand j'étais petite, quand eux ils étaient petits, moi je travaillais, j'étais comme X *(métier)* aux X *(quartier)*. Je travaillais 100%. Alors quand les, des fois ma fille elle doit aller chez le médecin, elle disait : « maman, tu me la gardes, tu me la, tu me la tiens ? » ; « oui, bien sûr ! » Ils venaient avec moi, ils travaillaient avec moi ! Ouais. Surtout celui-là de, du X *(lieu)* là, le petit garçon, celui-là qui a 18 ans. Aujourd'hui X *(prénom)*, il venait tout le temps dans mon travail ! Il m'aidait à, il prenait une pâte, il nettoyait, il faisait semblant de nettoyer : « nonna je veux essayer, nonna ! » *(rires)*. Voilà.

**C : *(rires)*. Ils sont, ils sont très présents.**

**R :** Oui, oui, oui, oui, oui, oui.

**C : Quel type de soutien vous avez, justement de la part des proches, de la famille ?**

**R :** [Quel soutien ?

**C : Quel soutien] ils vous amènent ?**

**R :** Le soutien qu'ils m'amènent c'est quand j'ai besoin déjà, ils sont là. Je les téléphone, ils viennent vite, ça c'est, c'est. Moi je suis quelqu'un, je dérange pas trop, j'aime pas trop déranger mes enfants. Mais si en cas de besoin je suis obligée, c'est normal qu'ils viennent. Si en cas de besoin, pourquoi, autrement le reste, le soutien moralement, si je parle avec mes enfants. Mais j'essaie de, de pleurer devant eux, j'essaie moins de pleurer, moins de me plaindre, de ma maladie, pour pas les faire souffrir ! Alors j'essaie toujours d'être une femme rigolote, que je, on dirait que c'est moi qui veut le, je soutiens à eux. Ouais. Voilà, mais autrement si, la chose qu'est-ce que j'ai, si en cas j'ai besoin, je peux les appeler, ils viennent, s'ils sont à la maison bien sûr quand y a, ils travaillent. Mais X *(prénom)*, elle est à X *(lieu)*, elle travaille pas, elle est maîtresse de gym, mais elle habite à X *(lieu)*. La petite qu'elle est ici, malheureusement elle est malade. Et ma fille qui habite à côté, elle travaille toute la journée ! Donc, si en cas, c'est le soir qu'elle est là. Mais elle souvent, j'appelle celle-là de X *(lieu)*. Mais j'appelle vraiment en cas de besoin, vraiment si j'ai besoin, mais c'est pas que je l'appelle pour rien comme ça, j'ai le moral qui va bas, je vais la faire venir ici, non, non, non, non, non. Je reste dans mon petit cocon et j'essaie de, de comprendre pourquoi, j'essaie la, cette, cette, cette, cette oxygène, elle me travaille énormément, j'essaie de m'en sortir ! Mais, c'est vraiment quelque chose, si je continue comme ça je vais avoir une dépression à cause d'elle, hein ! Mais j'essaie de me battre, j'essaie de me battre, contre elle ! Voyez, pour pas arriver à cette dépression ! Parce que vous avez vu tout à l'heure, j'avais envie de pleurer ! Et ça c'est quand on, on, on entre dans une dépression. Donc, j'essaie de me battre contre cette, cette, cette oxygène. Quand ils m'ont amené ça, que mon médecin et mon pneumologue, *Docteur D*, il m'avait dit d'avoir l'oxygène à la maison, ils nous ont apporté cette bonbonne. Je lui ai téléphoné, j'étais fâchée, d'avoir cette grande bonbonne à la maison. J'ai dit : « je suis foutue, je suis foutue. Là maintenant, je suis foutue. Jusqu'à maintenant, je me sentais encore bien. Je suis foutue ! » Mais malheureusement c'est comme ça, il faut l'accepter. Je l'ai accepté, regardez qu'est-ce que j'ai fait, je lui ai fait un habit, pour pas le voir.

**C : [A la bon'.**

**R :** A la machine,] au, le truc là. Je lui ai fait un habit pour pas le, pas le voir, pour me sentir mieux de pas

trop le voir devant les yeux, voyez. Je vous dis   l'oxygène il m'a tellement détruite ! Et si je, si je me bats pas, elle va m'enterrer hein, je sais qu'il va m'enterrer hein, si je moi je me bats pas, hein ! Mais j'essaie moins d'y penser la journée, j'essaie de, de sortir, même que je l'aime pas l'oxygène, mais je sors quand même. Quand j'étais dehors, ça me gêne, cette bonbonne, mais je suis dehors ! Je vois autre chose que, que rester à la maison et puis réfléchir, voyez. Et puis voilà.

**C : Vous vous battez.**

**R :** Oui je me bats, oui comme à la, voilà, comme je vous ai dit avant, je suis une battante, je me bats. Oui. Mais je me bats aussi contre la mort, hein. Vous savez, j'ai pas peur de mourir mais, un jour tout le monde on doit partir *(raclement de gorge)*. Quand j'ai, j'ai passé par-là, je sais qu'est-ce que c'est, ça veut dire que, quand, quand j'ai eu le truc intensif, quand je me suis réveillé, j'ai dit : « mais je suis où ? J'ai été où jusqu'à maintenant ? ». Mais j'étais tellement bien vous savez. Y avait plus rien, y avait le calme, je souffrais plus, je pouvais respirer, parce que je pouvais plus respirer ! L'oxygène, la thyroïde elle m'avait complètement détruite ! En tout cas normalement j'avais ça plus que j'avais un côlon, une infection au côlon j'avais plus ça encore ! Et puis je m'avais cassé une côte ! L'année passée, je vous parle. Alors j'avais cassé une côte parce que j'étais tombée dans la baignoire, j'ai eu un malaise. Et j'ai eu un malaise, je tombe, je vais à l'hôpital, on m'emmène, l'ambulance ils m'apportent à l'hôpital, et ils découvrent, bon cette côte cassée, plus côlon qui était une infection du côlon, plus j'avais… Et j'avais cette thyroïde, qu'on avait, on savait pas que, que c'était la. Et plus je faisais des, une petite attaque du coeur ! L'année passée, hein. Cette année ça va, le coeur, les poumons, tout va bien. C'est juste l'oxygène qui m'embête. C'est juste ça. Je dis y a pas grand-chose pour. Alors moi l'espoir de vivre, l'espoir de, d'aller en avant, *(raclement de gorge)*, je pense que en m'opérant cette thyroïde, peut-être que les choses ils vont se mettre quand même un petit peu. Et ça me donne l'espoir d'aller en avant, l'espoir de m'opérer, l'espoir de.

**C : D'aller mieux.**

**R :** D'aller mieux, et puis voilà. L'espoir que l'oxygène il revient comme avant !

**C : Oui, oui. Vous attendez ce, ce [rendez-vous, oui.**

**R :** C'est ça.]

**C : Et pendant, quand vous avez eu ces visites des infirmières, une fois par mois, qu'est-ce qui vous a plu ou déplu, pendant ces visites, particulièrement ?**

**R :** Qu'est-ce qu'ils nous ont apporté, [qu'est-ce que ?

**C : Oui, qu'est-ce que.]**

**R :** Moi je vous ai dit avant ils nous ont apporté justement ce soutien moralement. D'avoir une personne qu'elle est là pour moi, déjà aussi. De s'occuper de moi, de ma maladie. De parler avec quelqu'un, déjà aussi, parce que c'était une personne que je pouvais parler avec. Et guérir, ils pouvaient pas me guérir quoi, normalement ils me donnaient rien pour me faire guérir. Mais, elle m'apportait juste, beaucoup de moral, et moralement elle m'apportait beaucoup, moralement oui, ouais.

**C : De pouvoir parler avec quelqu'un.**

**R :** Oui, oui, oui, oui. Oui, oui, non, c'était une fille, vraiment ouais, on parlait de la, on parlait de ça comment j'allais, qu'est-ce que j'ai fait, qu'est-ce qu'on a fait pendant un mois, comment je me suis sentie, si j'étais fatiguée, si j'étais pas fatiguée. Y avait toutes ces questions un petit peu, pour ma maladie hein! Mais, la présence de cette personne à moi, personnellement à moi, elle m'a apporté beaucoup de consolation, présence humaine, le soutien moral, voilà.

**C : Oui. Vous pouviez aussi poser des questions sur la maladie ?**

**R :** Oui, oui, oui, oui, oui, oui, tout à fait, oui, oui.

**C : Est-ce qu'y a des informations supplémentaires que vous auriez voulu avoir pendant?**

**R :** Non, non, non, non. Y a rien qu'ils pouvaient me donner. Donner quoi ?! J'étais malade, j'étais malade, alors, donc elle était là, pour savoir comment je, comment ça allait passer, comment ça avait passé le mois comme si j'étais mieux. Mais c'est vrai que j'étais stable, j'étais bien stable moralement, j'étais bien, j'étais stable. L'année passée, je vous parle hein. Cette année non, ça a pas été comme ça, ça a été différemment, et puis voilà. Depuis le mois de juin, le mois de juillet, ça a pas, ça, ça a commencé cette oxygène. En tout cas normalement il avait bien repris, à l'hôpital cantonal, je n'avais pas, je devais marcher toujours avec l'oxygène. Puis après tout d'un coup, il s'est repris, voyez c'est ça que ça vous fait réfléchir. Après un moment il a bien repris à l'hôpital. Ils ont rien fait pour que il se reprend, c'est pas que, moi j'avais l'oxygène, et puis après on voulait me prendre la tension et tout, ils voyaient que l'oxygène il était bien stable. Et puis, ça allait bien ! J'ai dit à X *(hôpital)*, ça allait bien aussi. C'est vrai que quand je marchais, il descendait, mais il restait à 88. Il restait comme ça avec, avec la marche. Mais là, ça baisse peu à peu, ça baisse toujours plus. Moi j'ai constaté moi-même, que quand je, je, je marche, je fais des trucs <u>sans</u> oxygène, il arrive à 84 ! Parce que moi j'ai l'appareil pour, pour voir, alors je m'assis <u>vite</u>, pour voir combien il a descendu ! Il descend 84, mais même à 84, j'arrive bien à respirer ! Et une fois il est arrivé à 75 ! Et à 75, alors c'est catastrophe ! Catastrophe, là vous pouvez plus hein. Ça j'avais constaté moi. J'étais en train d'avoir même un malaise dans le balcon. J'ai rentré vite à la, dedans, je me suis mis l'oxygène. Un petit effort que j'avais fait. Donc, je vous explique ça, ça veut dire que y a quelque chose qui m'embête, xxx oxygène. Alors ma maladie c'est toujours la même, y a pas de, y a pas de, y a toujours la même ! C'est vrai que depuis deux ans en arrière, en 10 ans, deux en arrière, j'avais le, j'avais le, le souffle à 250, 280 ! Depuis l'année passée, il a <u>descendu, c'est vrai ça</u> ! Mais moi en travaillant, je l'essaie de le, de, de de nouveau de me mettre un, de travailler mes poumons, voyez. Mais, voilà, c'est ça, c'est.

**C : Et puis vous allez aussi faire le, l'activité physique trois fois par semaine, vous disiez ?**

**R :** Oui, oui, oui, tout à fait, oui, oui, là je vais aller à X *(hôpital de réhabilitation)*. Quand je peux pas aller, je téléphone et je vais pas. Mais c'est vrai que ça m'a, seulement il me prend, me prend toute la journée. Que je dois partir à 11 heures le matin, d'ici, pour arriver à 13 heures à l'hôpital. Alors imaginez-vous, hein. Je dois prendre le bus à X *(lieu)*, le tram à X *(lieu)* et le petit bus à X *(lieu)*. Donc le petit bus après il fait tout le détour, ça me fait perdre le temps. J'arrive à 13 heures, et puis après je me déshabille, et puis après on passe à la gym ! Ils ont toujours, normalement c'est à 13 heures 30, mais moi je commence toujours avant, et je pars avant. Et en revenant, je fais un petit peu moins, moins vite ! Non, plus vite ! C'est pourquoi le parcours de, de petit bus il est plus court. Alors je descends plus vite à X *(lieu)*, je prends le tram, je prends un autre bus et puis je rentre. Mais c'est la marche ici que ça me prend du temps. En descendant depuis le bus à la maison. Alors voilà, je suis pas à la maison avant 15 heures 30, hein !

**C : Ça, ça prend la journée.**

**R :** Ah moi ça me prend la, ça me bouffe la journée. Et ça, j'aime pas du tout. Déjà le lundi, mardi et jeudi j'ai mes petits-enfants. Donc le matin je peux rien faire ! Si je dois aller chez un médecin, je dois aller toujours l'après-midi. Maintenant c'est l'été, c'est l'hiver, ça me fait rien de pas sortir le matin ! Je préfère sortir l'après-midi, c'est plus doux disons. Mais quand il vient l'été, mais bon après, les infirmières qui viennent le matin ici. En tout cas moi le lundi et le, le lundi et le jeudi, non, le lundi, et le vendredi et le mercredi, je peux pas sortir le matin, c'est pourquoi ils viennent les infirmières. Y a X *(soins à domicile)* qui vient, à cause de ma jambe d'abord. Mais après l'année prochain, ils viennent qu'une fois par semaine. Donc après j'ai, j'ai le vendredi et le, et le lundi libre. Si au cas l'été je veux sortir, je peux sortir voyez, j'essaie de calculer un petit peu pour, pour pas rester tout le temps à la maison.

**C : Pouvoir sortir un peu.**

**R :** Ouais. Maintenant l'hiver, ça me fait rien de pas sortir le matin, je sors plutôt l'après-midi. On mange vite, je mange vite, je fais la vaisselle et je m'en vais, puis bon. Mais voilà, c'est, bon, ma, ma semaine elle est bien prise, elle est bien, ça c'est bien déjà. Pourquoi j'ai pas le temps de réfléchir à mon oxygène. Voyez, là j'ai pas d'oxygène et je suis assis, hein. Je vais voir combien j'ai *(elle prend sa saturation)*. Et puis, si je suis assise, je fais rien, ça va ! Là elle doit être 93, peut-être. Mais ça suffit que je bouge, et il arrive à 88 !

**C : Ça descend.**

**R :** Ouais. Voyez, 92 *(elle regarde son saturomètre)*. Maximum 93, elle fait. Qu'avant j'avais 94, 95, 96, sans rien faire. Non, y a vraiment un gros problème.

**C : Quand vous bougez ça, ça descend.**

**R :** Voyez, il ne bouge pas, hein *(en regardant le saturomètre)*. Mais il a descendu, regard', bon maintenant 91. Il monte entre 90 et 92. Mais bon, peut-être que je parle aussi, je sais pas. Mais c'est pas bien, je suis pas contente, je suis pas bien *(soupir)*. Eh oui !

**C : Et, peut-être qu'est-ce que, qu'est-ce que vous attendiez en participant à, à cette étude sur la BPCO ?**

**R :** De, d'avoir, de, que, on trouve… Eux, qu'ils trouveraient peut-être quelque chose pour *(rires)*, pour me guérir, pour que j'aille mieux, voilà ! J'ai fait ça justement pour que les médecins ils étudient cette chose, pour que, pour trouver encore quelque chose qu'on, nous on va mieux, que on fait des progrès sur cette maladie ! Pourquoi, moi je trouve que cette maladie, moi ça fait, ça fait déjà de l'année 2000 que je suis malade. Donc, y a pas eu de changements. Donc moi je prends toujours les mêmes médicaments. Donc y a pas eu de progrès sur la BPCO ! Et moi je, qu'est-ce que j'ai fait ça, je, je me suis mis avec eux, j'ai fait ça, c'est à cause que les médecins, ils peuvent trouver quelque chose de mieux, pour nous ! Pour qu'on est mieux ! Je sais que c'est pas la faute, c'est la faute à nous que si on est, la faute à qui, c'est la faute au destin je sais pas, qu'on est malade ! C'est la faute à la cigarette oui, pourquoi pas ! Je dis pas « non », la cigarette elle m'a pas aidé non plus. Mais je trouve que, que la BPCO, elle est stable, elle fait pas de progrès, pour moi. Ils essaient de trouver, la gym c'est vrai, la gym ça nous fait du bien, ça nous fait travailler, je dis pas « non ». Mais, comme médicaments, c'est toujours la même chose, y a pas de changements. Donc, je trouve qu'ils, ils restent trop stable sur la maladie.

**C : Donc essayer de trouver des, des progrès.**

**R :** Oui, j'aimerais bien qu'ils, qu'ils trouvent encore quelque chose que, que nous on va mieux que, pour aider, pourquoi normalement j'ai trouvé tous les gens autour de moi, tous ceux là qui sont malades BPCO, c'est des gens qui sont très coopératifs, voyez, ils essaient d'être bien, ils essayent de s'en sortir. Moi je vais à la réunion là avec mon pneumologue, on discute un petit peu, on est quatre, cinq là. On essaie de discuter. Mais personne elle dit : « je vais mieux ! ». Personne ! Toujours : « ça va, ça a été ». Mais y a pas un progrès de cette maladie ! On reste trop stable, c'est vrai, mais on vit, on vit quand même, c'est vrai ! Ça baisse pas non plus, bon c'est vrai qu'il faut faire attention de, puis se couvrir, faut pas avoir de courants d'air, de pas être avec des gens malades. Ça c'est logique, qu'on est très fragiles ! Mais y a pas de progrès, y a pas de, je trouve qu'ils sont, ils sont mis là, ils bougent pas les médecins. Ils disent : « on, on n'a plus rien à trouver ». Voilà, mais moi c'est pour ça que j'essaie de, de faire ça pour que les médecins, s'ils peuvent trouver, un petit peu quelque chose qu'on puisse aller un petit mieux que, que, que, que comme on est. Que si nous on va mieux, c'est pourquoi c'est nous qu'on veut, c'est nous qu'on se bat, moi en tout cas, moi je me bats contre ma maladie. Mais ma maladie elle est là. C'est vrai, disons, on peut rien faire. Mais bon voilà, moi j'ai, c'est pour ça que j'ai, j'essaie de collaborer, de, avec eux, pour qu'ils puissent. J'ai toujours dit la même chose : « je vais bien, mais je suis stable ». J'ai.

**C : C'est stable.**

**R :** Les médicaments que, que je prends, c'est pas eux qui, c'est eux ils me font, ils me donnent xxx, ils me font respirer, mais ça améliore pas ma. Si j'améliore, disons que je vais améliorer peut-être, c'est grâce à la gym, peut-être que c'est la gym ! Mais pas grâce aux médicaments, pas du tout.

**C : Les médicaments ça [améliore pas.**

**R :** Parce que normalement ils disent] : « qu'est-ce qui est abîmé, c'est abîmé ». Ça c'est vrai, ils ont raison, d'un coté je peux pas non plus. Mais pour dire j'ai collaboré là justement pour que, pour que les médecins ils, ils. On entend autour d'eux les gens comme ils parlent. Essayer de trouver quelque chose de mieux, je sais pas, moi.

**C : Trouver quelque chose pour la [maladie.**

**R :** Ouais, ouais.] Oui, oui, tout à fait.

**C : De mieux que les médicaments.**

**R :** Oui, oui.

**C : Oui. Et, je sais que vous avez pu discuter de, de différentes choses pendant ces visites des infirmières. Je sais pas si vous avez parlé aussi des types de soins qu'on souhaite en fin de vie quand, quand on peut écrire ce qu'on appelle des directives anticipées, où on dit si une fois on n'est pas bien, qu'est-ce qu'on aimerait comme soins. Je sais pas si c'est quelque chose que vous avez discuté avec les [infirmières ?**

**R :** Je crois pas, non je crois pas.] Non, non, je crois pas qu'elles m'ont parlé de ça, non. Avec l'infirmière qu'elle est venue, on a d'abord rempli un questionnaire. Et puis elle m'a demandé comment j'allais, comment ça s'était passé. Voilà, moi j'ai raconté un petit peu mon mois, de ma maladie, de ma vie, de mes choses, de, voilà, c'est tout. Mais le reste, ouais.

**C : Vous avez discuté d'autres choses.**

**R :** Oui, oui. Non, non, on n'a pas parlé, non, oui.

**C : Oui, de, de ça.**

**R :** Je me rappelle pas que.

**C : Que vous avez discuté.**

**R :** Non.

**C : C'était plus comment vous vous sentiez.**

**R :** Voilà, comment ça a été le mois, tous des trucs comme ça.

**C : Et est-ce que, de, de quoi d'autre auriez-vous besoin, ou auriez-vous eu besoin, par rapport à votre maladie ?**

**R :** Quelque chose qui me, qui me, quelque chose je sais pas, je vous ai parlé de cette oxygène ! Encore pour ma maladie, j'arrive à marcher, j'arrive à respirer, <u>ça va</u>, je, je pourrais continuer comme ça, si je reste stable, pourquoi pas. Mais, mais l'oxygène qui me, qui me détruit... C'est l'oxygène. Je me sens plus que malade maintenant. Avant j'étais malade, mais on le voyait pas. Ça veut dire moi je le savais, mais les gens ils le voyaient pas. Je vis pas pour les gens, ça c'est vrai. Mais ça, ça, c'est important pour moi. Que mon entourage il me voit bien, il me voit souriante, qu'il voit pas toujours, moi j'aime pas pleurer devant les gens, vous savez. Mais cette oxygène qui me, qui me détruit, oui. Mais personne il a su me donner une réponse, hein, personne, hein.

**C : Par rapport à l'oxygène ?**

**R :** Par rapport à l'oxygène, ouais.

**C : Pourquoi vous avez besoin de l'oxygène.**

**R :** Une réponse, je sais pas moi, *(soupir)*. C'est vrai qu'ils disent ce qu'il peut, il peut dire quoi : « c'est votre maladie, on peut rien y faire ! Grâce à ça vous pouvez vivre ». C'est ce qu'il a dit le, qu'est-ce qu'il a dit le médecin, il a dit : « ça il vous fait aller mieux, que d'habitude, donc ». Mais voilà, c'est, c'est pas le cas pour moi ! Je dis pas non ! Je dis mais c'est vrai que. Mais bon.

**C : Ça ne vous fais pas aller mieux l'oxygène, pour vous.**

**R :** Dans ma tête non, dans ma tête non. Dans le corps peut-être oui. Je vais, peut-être que voilà, je suis moins essoufflée quand je marche, c'est vrai. Mais dans ma tête, non, moralement non, il m'aide pas. Ça c'est moralement qu'il m'aide pas. J'ai jamais, c'est une chose que j'ai jamais voulu avoir. Je voyais ma copine, je disais toujours : « mon dieu, pas moi, s'il te plaît ». Mais c'est arrivé aussi à moi, depuis l'année passée, cette année c'est aggravé complètement. L'année passée encore j'allais bien, sans, j'étais bien vous savez l'année passée. Le professeur il m'a fait arrêter ces médicaments, depuis là c'est une

catastrophe ! Arrêter ces médicaments c'est une catastrophe. Et là j'ai plus avoir *(soupir)*. Et cette année ça a été très, très dur. Je me suis, je me suis battue, c'est grâce à, bon grâce aussi à X *(soins à domicile)*, hein, X *(soins à domicile)* aussi il m'a beaucoup aidé, pas tous, tous les infirmières. X *(soins à domicile)* elle est bien, mais, y a trop de, y a trop de, j'ai pas toujours la même, j'ai pas toujours la même infirmière. Moi, la femme de ménage, disons qu'elle vient de temps en temps une, de temps en temps l'autre, bon, je m'en fous. Mais l'infirmière pour moi c'est important d'avoir une infirmière. Quand même avec mon, mon infirmière moi je parle beaucoup. Je raconte comment ça a été la journée, je raconte comme j'ai dormi. Mais je ne peux pas, c'est ça qu'ils comprennent X *(soins à domicile)*, je ne peux pas raconter à tout le monde mes problèmes. Et on n'est pas, nous malades, qu'est-ce qu'ils comprennent pas non plus, nous malades on a besoin de parler, on a ce besoin. On peut pas garder tout dedans, on est des personnes malades. Donc, moi j'ai une, une infirmière, elle s'appelle X *(prénom)*, c'est à elle que j'ai tout raconté ma vie. Cette fille elle est super, c'est une infirmière qui vient, elle discute avec moi, on parle de ma vie, je raconte mes petites choses, mes petits bobos, mes petites choses. Après quand elle s'en va je me sens bien. Mais je ne peux pas raconter ça à tout le monde. C'est ça X *(soins à domicile)* ils veulent pas se mettre dans la tête que ça, c'est pour ça je vais arrêter. Je vais venir une fois par semaine, juste pour, avoir, que si en cas y a quelque chose qui va pas, j'ai mon infirmière qui peut faire des choses pour moi. Elle peut téléphoner au médecin, à l'hôpital, comme elle a fait la dernière fois, X *(prénom)*. X *(prénom)* c'est elle qui a tout fait ! Elle a téléphoné au pneumologue, téléphoné à l'hôpital, à l'ambulance, elle était là jusqu'à la dernière minute ! Elle m'a vu me mettre dans l'ambulance, et puis après elle m'a quitté ! Ça j'aime bien ! C'est qu'est-ce qu'il y a, y a trop de, X *(soins à domicile)* ils comprennent pas, comprennent pas. Et ça j'aime pas du tout.

**C : Vous aimeriez avoir la [même personne.**

**R :** Une ! Voilà.] Que de temps en temps ça arrive dans les vacances, y a une remplaçante, c'est pas la fin du monde. Que de temps en temps, X *(prénom)* elle est malade, c'est pas la fin du monde ! J'accepte ! Mais toutes, j'en ai jusqu'à maintenant je ne sais pas combien d'infirmières ! J'ai, X *(prénom)* je l'ai eue hier. Bon elle était malade, aussi. Aussi elle était malade. Mais, c'est pas qu'on me met toujours la même, pour un bout de temps. Non. Y a eu là, une dizaine de fois, y a eu une jeune fille qui est venue, mais ça m'intéressait pas de parler avec elle. Ça m'attirait pas de parler, non. Moi, il faut que je suis, pour parler avec quelqu'un, faut que je suis à l'aise, faut que je suis bien, faut que la personne elle m'est sympathique, faut qu'y ait une attirance, faut qu'y ait, y ait une sympathie qui passe entre moi et la personne. Si y a pas ça, je ne peux pas parler, je peux pas raconter ma vie, et je ne peux pas pleurer devant elle. Parce que je suis sûre qu'il va pas me comprendre.

**C : Donc là vous allez arrêter.**

**R :** Voilà.

**C : Vous avez pas envie de devoir raconter aussi à chaque fois.**

**R :** A chaque fois les gens. Moi cette année, j'ai pas été bien, hein ! Mais chaque fois qu'ils sont venus : « ça va madame ? » ; « oui ça va, oui ça va, ouais », « bye », non plus. Que hier avec X *(prénom)*, c'était la grande joie quand je l'ai vue ! Oh mon dieu on dirait qu'elle apportait le soleil chez moi ! C'est incroyable cette fille ! Quand elle m'a tapé la porte, et puis j'ai ouvert la porte, j'ai dit : « non mais c'est pas vrai ! ». Et puis après, on a discuté, on a parlé, on a, ouais, c'est, voyez.

**C : Des bons contacts avec elle.**

**R :** Ouais, ouais.

**C : Oui, oui.**

**R :** Y avait une autre aussi, j'aimais bien aussi, elle était brave. Ouais, elle était mariée, elle avait, je me rappelle plus comment elle s'appelle. Elle était bien aussi, c'est elle qui avait ouvert mon dossier, normalement. Puis après c'est X *(prénom)* que chaque trois mois, on va.

**C : [C'est important.**

**R :** L'autre je l'aimais bien aussi, l'autre.] Oui, y en avait deux que j'aimais bien. X *(prénom)* j'aimais, X

*(prénom)* c'était vraiment, c'est comme ma fille, si vous voulez. C'est une présence, ouais, de ma fille à moi, voyez, c'est quelque chose de particulier. L'autre, non, c'était comme une amie, si vous voulez. Mais c'est toujours les infirmières, ça reste toujours infirmière et madame X *(nom)*, non y a pas de.

**C : Mais c'était un soleil.**

**R :** Ouais, ouais, ouais, ouais. Mais les autres non, les autres ça va mais, c'est pas. Y avait une infirmière qui était xxx qui était aussi assez, assez gentille. Ils sont pas méchantes hein, ils sont très, très gentilles, très polies. Mais c'est moi, c'est moi qui, quand vous aimez pas quelque chose, voilà, vous aimez pas, y a rien à faire ! Vous pouvez pas faire des efforts, moi j'arrive pas à être souriante ou quand quelque chose il me plaît pas. Je, je, moi je dois montrer comme je suis. Je suis joyeux, je vous le montre, si je suis pas joyeux, je vous le montre pas. Y a rien à faire, j'arrive pas à être faux, faux, voilà, je trouve pas le mot.

**C : *(rires)* C'est, c'est important d'être bien avec la personne, [pour pouvoir parler.**

**R :** Voilà, tout à fait.] Ouais. Mais oui, c'est comme vous la même chose, je pense que vous devez être la même chose. Et puis voilà.

**C : Et, oui donc si, si je résume un petit peu par rapport à ces visites des infirmières. Vous les avez trouvées sympathique, la personne qui venait, [vous voir, la dame qui venait, voilà.**

**R :** La dame là qui venait. Oui, oui, celle-là, oui.] Oui, oui, ça je vous ai dit que oui.

**C : Une fois par mois. [Vous pouviez aussi, discuter, parler.**

**R :** Oui, oui, oui, oui, oui, oui, oui. Oui, oui, oui, oui.] Non elle était vraiment, elle était super, elle était, X *(prénom)* aussi, X *(prénom)* aussi c'était une brave fille, c'est une brave dame, une dame d'un certain âge. Aussi elle était souriante quand elle venait, elle était toute contente.

**C : Donc là vous pouviez discuter aussi de, de tout et de rien pendant ces moments.**

**R :** Oui avec, bon pour dire la vérité, avec, avec la dame-là, pas X *(prénom)*, bon X *(prénom)* non, elle sait rien de moi.

**C : *Julie (infirmière de recherche)* ?**

**R :** Voilà. Non, j'ai pas raconté ma vie, non. Mais on a parlé de, on parlait de tout. J'étais bien avec elle, on parlait de ma santé, elle me comprenait, voyez c'est ça. *(quelqu'un sonne à la porte)*.

*(conversation avec la personne à la porte)*.

**R :** Mon concierge. Aussi avec ma concierge. Il est tellement chou, il est là, il est serviable, il vient. C'est, c'est formidable. Puis de temps en temps je fais la pizza, je le fais une pour lui, je fais le pain, je le fais une part pour lui. J'essaie toujours récompenser la gentillesse, voyez.

**C : Oui.**

**R :** C'est ça !

**C : Le, le concierge il est là aussi.**

**R :** Oui, il est là pour moi, il est vraiment gentil, vous savez j'ai de la chance. Les gens ici ils travaillent tous, vous savez. Moyennement, y en n'a peu qui travaillent pas.

**C : Oui. Oui, et puis, par rapport à ces visites donc des infirmières, c'est, vous avez aussi pu avoir des informations sur.**

**R :** C'est quelle heure ?

**C : C'est, 10 heures 30.**

**R :** Mon petit-fils, oui.

**C : Il va arriver ? On va.**

**R :** Non, il va pas arriver maintenant, mais, [faut que je prépare à manger, c'est ça.

**C : Bientôt. On va, non on va, on va terminer.]**

**R :** Oui.

**C : Voilà, si je résume, donc avez pu parler de, de la maladie aussi pendant ces visites, et elle a pu vous, vous remonter un peu le moral.**

**R :** Oui, je vous ai dit, oui ! Oui, oui, oui, tout à fait !

**C : Donc, donc, voilà. Et maintenant ce qui, ce qui vous fait plus du souci, c'est plus par rapport à l'oxygène.**

**R :** Oui.

**C : Et, et si vous avez participé à cette étude c'était pour, pour qu'on puisse faire des [progrès médical, pour cette maladie, voilà où y a.**

**R :** Voilà, voilà, voilà, vous avez tout compris.] Vous avez tout compris, vous avez tout compris.

**C : Où y a, pas grand-chose qu'on peut faire pour, c'est une maladie qui est, qui est assez stable et on peut pas, [pas grand-chose à faire.**

**R :** Ouais.]

**C : Donc, donc voilà je sais pas si vous aviez autre chose à rajouter ?**

**R :** Non. Je crois le plus important je vous ai dit.

**C : Oui. Vous m'avez aussi dit que vous étiez bien entourée par votre [famille et par les professionnels de la santé aussi.**

**R :** Oui.] Non, pour ça, j'ai, j'ai pas de problèmes, j'ai. Non, je suis bien entourée, non, non, y a pas de. C'est ça qui me, qui me, peut-être qui m'aide, de, d'aller en avant, c'est pourquoi j'ai des personnes gentilles autour de moi. Pourquoi, si, je serais une femme solitaire, je souffrirais plus, de cette maladie, voyez. Mais, grâce à eux, grâce à mes petits-enfants, grâce à mes amis qui me téléphonent tout le temps aussi. Je vais en avant.

**C : [Vous vous battez pour la.**

**R :** Mais ça s'est arrivé] depuis toujours, je suis comme ça. Je vous répète, c'est toujours avec, toujours de l'oxygène. Quand j'avais l'oxygène, j'étais pas comme ça. Le matin moi à 8 heures 30, j'étais déjà dehors. Maintenant je sors plus le matin, à 8 heures 30, voyez. Mais à 8 heures 30, j'étais déjà dehors, j'allais aux X *(quartier)*, j'allais boire le café avec mes amis. Ça fait un mois qu'on me voit pas. C'est toujours que je suis pas bien, à cause de l'oxygène je suis pas bien. xxx j'ai pas envie, je, je sors, pourquoi, je dois sortir, je suis obligée, d'aller à la poste, d'aller à la X *(magasin)* pour faire mes courses, mais pas pour me promener, faire une promenade comme ça avec mon oxygène, non, je le ferais pas. Ça me dit rien.

**C : C'est un peu plus difficile avec.**

**R :** Ouais, c'est plus, non je, je, non. C'est, c'est, c'est, c'est justement <u>l'obligation</u> qui me fait souvent sortir, pas le plaisir, voilà. Avec l'oxygène.

**C : Vous vous battez [pour, pour aller mieux.**

**R :** Donc voilà, je, je, je me bats encore] jusqu'à l'année prochain, je vais voir qu'est-ce que ça donne. J'ai cet espoir de, de, de, de l'opération, de détruire cette thyroïde. J'ai un espoir que après ça va mieux. Alors j'ai la patience d'attendre, de, de, d'aller en avant et puis on va voir juste là. Parce que moi j'ai comme l'impression que la thyroïde elle y est pour quelque chose ! J'avais demandé à mon docteur, à mon oculiste pour les yeux, j'ai dit : « vous savez ». J'ai dit : « moi je suis, j'ai un problème thyroïenne, thyroïde ». Elle a dit : « ça peut jouer sur les yeux ». Ah elle a dit : « bien sûr madame X *(nom)* ». Elle a dit : « quand vous enlevez la thyroïde, vous venez on va faire un contrôle ». Alors vous voyez, touche même les yeux, alors, hein. C'est qu'elle y est pour quelque chose cette conne, cette connerie, cette conne de, de, de thyroïde, ouais. Ça, ça m'aide pas beaucoup, ça m'aide pas, non. Je lui ai dit à la doctoresse à l'hôpital, je lui ai dit : « je suis très fatiguée, je suis pas bien ». Mais voilà, hein, elle fait, elle essaie de faire le mieux possible, mais. Mais là elle a, bon elle a bien bougé ces derniers temps elle a bien fait la doctoresse. Elle s'est donné, devant moi elle avait tout fait ! Elle a pris le téléphone, elle a téléphoné à celui-là, celui-là, celui-là. C'est pas quelqu'un, elle dit : « oui, je vais faire le nécessaire, et puis après je vous téléphone ». Non, non, non, non, non. C'est devant moi qu'elle a tout fait. Elle a pris les rendez-vous, tout ! Elle avait tout fait ! Donc c'est là que j'ai vu la doctoresse elle avait quand même un, un intérêt pour moi, vous voyez. C'est ça qu'on voit les, les personnes qui ont du, vraiment on s'occupe de vous, font les choses vite. Et là elle m'avait pris déjà deux rendez-vous, là ! C'est pour ça que ça me, pour la doctoresse je suis, je sais qu'elle va faire quelque chose.

**C : [Oui, et vous espérez aussi que, ça ira mieux avec l'opération.**

**R :** Oui, oui, oui. Mais là maintenant je vais à cette rendez-vous] là, je vais faire cette truc-là. Et après les fêtes, je la téléphonerai pour voir où elle en était. Puis je vais lui dire : « vous vous dépêchez, hein, le plus vite possible, que moi je peux pas rester comme ça ». Je sais pas l'année prochaine qu'est-ce que ça va donner.

**C : [Je vous remercie d'avoir pris le temps de.**

**R :** De rien, de rien. Merci, de rien, de rien. Ça m'a fait du bien.]

**C : De partager votre expérience.**

**R :** Ça m'a fait du bien parler aussi, voyez ! Comme ça les, ils voient quand même une personne malade de BPCO, comme elle, comme elle, comme elle vit avec sa maladie ! Et je pense que tout le monde il est un petit peu comme moi. On a quand même le moral qui monte et qui descend, qui. L'espoir d'être mieux, l'espoir de vivre mieux, de respirer mieux. Ça nous aide à aller en avant, je pense.

**C : Oui, oui.**

**R :** Malheureusement c'est comme ça. Je suis arrivée à 70 ans, j'espère d'arriver à 80 ans. La seule chose que je me souhaite de, je voudrais voir un de mes petits-enfants marié, un, quand même un ! Et j'aimerais bien venir de nouveau grand-mère, une deuxième fois. C'est la plus belle chose que, si je peux l'avoir. Etre deux fois grand-mère, et puis après je m'en vais tranquille.

**C : *(rires)*. Une deuxième fois grand-maman, hein, ouais, une deuxième fois nonna.**

**R :** Vous savez c'est beau ça, vous savez ma copine elle est, elle est deux fois grand-mère. Mais c'est, c'est magnifique !

**C : Je crois qu'on peut éteindre.**

## Participant n°16

Nom d’emprunt : Arthur
Âge : 64 ans
Sexe : M
Groupe : Contrôle

**C : Vous avez participé à une étude qui avait pour objectif de comparer le traitement habituel de votre maladie pulmonaire, la BPCO, avec une prise en charge précoce, soutenue et intégrée. Vous avez été dans le groupe, bénéficiant des soins habituels. Pouvez-vous me dire comment ça s’est passé ?**

**A :** Ben j’ai été suivi par le *Docteur D.*, donc qui était précédemment le médecin qui m’a accueilli quand j’ai été aux urgences, puis après il s’est mis à son compte. Alors, quand je suis sorti des soins intensifs j’ai été chez lui. Donc, j’ai fait tout le traitement avec lui, c’est lui qui m’a appris comment utiliser ben les aérosols. Après, quand je suis sorti de X *(hôpital)*, c’est lui qui est venu ici pour me, m’expliquer ici à domicile, l’usage de l’oxygène, comme ça, enfin médical. Et, puis après ben j’ai eu les, comment s’appelle, entre le *Docteur E.* et lui, toute une série de, de, de, de contrôles en moyenne toutes les deux semaines, je voyais un médecin, soit l’un, soit l’autre, et toutes les deux semaines j’ai été suivi. Toujours avec, avec régulièrement, tous les trois mois, la soufflerie, c’est-à-dire le, la cabine de, de contrôle, capacité pulmonaire et compagnie. Bon ça j’ai fait à peu près en moyenne tous les trois mois. Bon c’est là qu’on a vu que ma, sitôt qu’y avait le moindre pollen, ou le moindre, le moindre coup de froid, chaque fois ma capacité pulmonaire, ben diminuait, que y a eu, ça s’est détérioré régulièrement. En plus cette année, donc au mois de juin, début juin, j’ai fait plusieurs baisses de pression ici, je me suis rendu moi-même à l’hôpital, enfin c’est un, un de mes amis m’a amené à l’hôpital, le X *(date)* juin, je suis ressorti le X *(date)* août. Il a fallu tous les traitements entre l’hôpital, X *(hôpital de réhabilitation)*, après X *(autre hôpital)*, pour me remonter un petit peu, parce que j’ai fait des baisses de pression, pendant trois jours. Je me suis fracassé contre le, le, le sol, enfin toute la tête, les hanches, tout qui était cassé, quoi. A l’hôpital en plus, ils m’ont cassé deux côtes, en me mettant l’électrocardiogramme dans le dos. Donc, après ben j’étais sous morphine, en injection dans le dos, tellement ben les, les, chaque respiration, les côtes frottaient, donc enfin j’ai, j’ai, j’ai xxx depuis, depuis trois ans quoi, maintenant. Puis en plus, c’est pratiquement impossible de contrôler les douleurs du dos, parce que je dois, je prends un minimum de, de X *(morphine)*, je peux prendre jusqu’à X *(morphine)* 60, quatre fois par jour, moi j’en suis à X *(morphine)* 30, trois fois par jour. Mais si on me, si je pouvais doubler la dose, je serais peut-être un peu plus confortable. J’imagine pas que les douleurs puissent disparaître complètement, mais je, ben là maintenant c’est, c’est ouais, heureusement que j’ai ma compagne moi, parce qu’autrement, xxx vite en bas quoi. Mais si j’avais pas ma compagne et ma fille, j’aurais fini, j’aurais arrêté déjà depuis deux, trois ans, quoi, tellement c’est, tellement c’est violent, quoi.

**C : Les douleurs, aussi.**

**A :** Les, les douleurs, et l’angoisse. Le moindre effort, si je me lève, je suis sûr de, si par hasard j’ai besoin, tout à coup un besoin pressant pour aller aux toilettes, je vais là-bas, j’ai oublié de mettre, j’ai pas augmenté l’oxygène, si j’ai, ma compagne est pas là, mais je me traîne mais à quatre pattes pour, en reprenant l’air, pour réussir à venir ici pour mettre un, un débit plus, plus important. Donc c’est, c’est très, très pénible comme maladie et c’est très angoissant. Et en plus bon, du, du, vu, vu ce protocole de greffe, ben j’ai plus le droit aux trucs contre les angoisses, j’ai, j’ai, de moins en moins de trucs contre les douleurs, très, très peu, ouais, alors je mets les patchs, ça c’est une chose, pour le moment X *(assurance)* n’a jamais rien dit, mais j’en prends à peu près une vingtaine par semaine, j’en ai là, j’en ai sur l’épaule. Parce qu’y a tellement de fortes tensions, sur le, la cage thoracique que, malgré les soins de, de mon ostéopathe, les épaules qui, comment s’appelle, qui tirent dedans, puis ça, ça déclenche tout des, des irritations, des, deux emboîtements d’épaules. Donc j’ai les patchs sur les deux épaules, normalement, plus aux, aux endroits où j’ai eu des côtes cassées, parce que j’ai eu deux fois les côtes cassées. Une première fois à l’hôpital, quand ils m’ont fait l’échographie cardiaque, et une deuxième fois quand ils ont oublié la prise de, l’électrocardiogramme dans le dos pendant trois heures. Puis elle a appuyé, elle a appuyé, puis là les côtes ont cassé, quoi. Donc, j’ai toute la cage thoracique qui est, depuis quatre ans qui est pas équilibrée. L’osthéo, il arrive pas à la remettre en place, parce que y a de telles tensions musculaires, que ben sitôt qu’il met en place d’un côté, ça relâche de l’autre. Donc y aurait, les péripéties

comme ça, je pourrais vous en parler jusqu'à demain quoi, donc *(rires)*. Si vous avez des questions plus précises, parce que *(rires)*.

**C : *(rires)* Ouais. Mais alors peut-être, qu'est-ce que vous attentiez en participant à, à cette étude ?**

**A :** Pour moi, rien. Je voulais être disponible pour qu'y ait une comparaison qui soit possible par rapport aux autres, parce que j'ai, dès le départ, j'ai dit que je pouvais pas me permettre de faire un traitement expérimental. J'étais dans un état trop désastreux, pour prendre le risque de faire un truc expérimental, dont on connaît pas si c'est bon ou pas. J'avais besoin de quelque chose de bon. Donc c'est pour ça que je suis pas, que j'ai demandé de, de, d'avoir le traitement connu. C'est-à-dire le, les chambres, les inhalateurs, les trucs de dilatation et tout, donc le truc habituel. Parce que si je prends un truc expérimental qui n'est pas valable, dans l'état où je suis, je peux en mourir, donc c'est pour, c'est ce qui a été discuté, c'était je crois avec le *Professeur A. (pneumologie)*, si je m'en rappelle bien, avec sa, sa collaboratrice qui était venue me voir à l'hôpital. Elle a expliqué à *Professeur A. (pneumologie)* et là on a décidé de faire le programme normal, pour pas prendre le risque d'avoir des complications. Donc dans le cadre de ce programme normal, moi ben je voulais juste, simplement que, que pouvoir continuer à vivre de façon pas trop pénible, quoi, tout en sachant, parce que j'ai, *Docteur D.* a été très clair, qu'à la moindre complication, le moindre pollen, ou le moindre petit, bronchite, ben j'allais de nouveau avoir une perte de la capacité pulmonaire, puis j'allais devoir monter petit à petit les doses d'oxygène que je prends, augmenter le, les doses de remède, et puis que l'état ben se détériorait petit à petit, puis qu'il fallait ben, arriver le plus vite possible à, à la greffe, quoi. Mais seulement le problème, c'est que, y a eu des complications avec les, quand on fait les radiographies, ouais les radiographies pulmonaires, ou les scans, j'ai eu le malheur, ils voyaient régulièrement des tumeurs, enfin des nodules. Alors première fois ils les voyaient comme ça, un exemple ils les voyaient comme ça. Et trois mois après, ils disparaissaient, puis ils les voyaient comme ça. Alors ils disaient : « c'est quoi ces histoires ! C'est des tumeurs, c'est ceci, c'est cela ». Alors ils ont mis ben depuis le X *(date)* 2013, jusqu'au X *(date)* de cette année, pour se rendre compte que j'avais un, une chose qui était rarissime, un champignon, comment ça s'appelle, ancestral, donc qui a disparu depuis deux siècles. Parce que je suis architecte, et j'ai participé à la démolition de la maison X *(nom)*, non X *(nom)*, pardon, à X *(lieu)*, qui est une maison du, du 17ème siècle, de 1685. Et dans le cadre de la transformation, j'ai été sur place, en haut dans la toiture, quand ils ont découpé la charpente. Et c'est un champignon des vieux bois. Et le champignon il a disparu depuis deux siècles, il n'existe plus depuis deux siècles, ce champignon. Alors y a pas de traitements, et le problème c'est que ce champignon, petit à petit ben, il grossit un moment, il est là, il est visible, mais quand il disparaît, il laisse des scories, donc ça vous bouche une partie des poumons ici. Puis après il vient ailleurs, il s'enflamme, donc je, je, j'ai de nouveau des problèmes respiratoires, puis ils laissent des scories, ils ont mis jusqu'au X *(mois)* de cette année, quand ils ont poussé les contrôles, encore plus, vu que c'est de la greffe devenait, vraiment nécessaire, urgente. Et puis c'est là qu'ils se sont rendus compte qu'ils auraient dû, se dépêcher beaucoup plus, quoi. Parce que le champignon il peut, si d'un coup il se décide à, à se multiplier encore plus vite, je peux perdre tous les poumons d'un coup, donc. Ouais, je regrette ça, c'est qu'ils aient mis si longtemps pour faire, tous les, les tests sérieux, quoi. Parce que là bon j'ai passé deux semaines à, enfin dix jours à l'hôpital, en plus ils ont eu, ils se sont loupés, mais je les adore à l'hôpital *(ton ironique)*, à X *(hôpital)*, parce que c'est vraiment les rois, hein pour dire comme ça. Ils ont demandé de faire le, comment ça s'appelle, l'étude de l'intestin. J'en ai fait une en X *(date)*, qui était nickel, j'avais rien du tout ! Et en X *(date)*, donc 13 mois, 13 mois après, ils demandent d'en refaire une. Alors bon, on en fait une, ok ! Ils me descendent à 9 heures, en bas dans la salle. Alors je reste dans le couloir, au froid, sans, avec les pieds, juste avec une petite, les petits habits dans le couloir, aux courants d'air, jusqu'à 11 heures. A 11 heures, ils viennent me dire que le gars il refuse de me faire le truc, l'analyse, parce qu'il faut faire une légère anesthésie pour, pour me passer la tuyauterie, le, la caméra dans, dans l'anus. J'ai pas pu lui causer, il était, il est reparti dans l'autre salle. J'aurais pu lui dire que 13 mois avant, on me l'avait fait sans problèmes, et puis que une légère anesthésie, ou, ou même le faire sans anesthésie, ça allait pas me gêner beaucoup, hein. Alors bon, non, il m'a alors après, ils ont, ils ont décidé de me ramener dans la chambre, d'accord c'était superbe. Alors là ils m'ont mis dans la salle, mais en face du desk de, et trois fois mon nom a disparu de l'écran pour le, pour le renvoi, le comité de transport. Trois fois il a disparu de l'écran ! Donc à 17 heures, j'étais toujours en bas dans le desk, au froid, avec juste ma couverture, et compagnie. Alors là à 17 heures j'ai poussé une gueulée, mais ils m'ont entendu dans tout le couloir, puis c'est le responsable des desks, qui a téléphoné directement au responsable des transports, puis qui lui a dit si dans cinq minutes, il venait pas lui-même, ça allait faire du foin quoi, ça faisait juste quatre heures et demi que j'attendais dans le couloir ! Alors j'ai attendu 9 heures en tout, pour rien ! Et le lendemain ils ont décidé de me faire une gastroscopie, mais virtuelle. Alors de virtuelle, ça en a que le nom, hein. Ils vous passent au scan d'accord, mais avant ils vous gonflent, par l'anus, ils vous gonflent quatre litres d'air.

Seulement le problème, c’est que j’ai toujours maintenant le, le, le bourillon *(il montre son ventre)*, et puis qui est ultra sensible, qui fait à peine je touche, ça fait que je suis sous morphine, et que je le sens mais comme il faut, la douleur quatre ou cinq, facile, hein en permanence ! Ils m’ont tellement bien gonflé que les quatre litres d’air ils sont jamais. J’avais un ventre plat, j’avais les abdos, avant. J’avais encore un corps de sportif. Je suis ressorti j’ai un ballon, puis qui fait mal. J’ai de l’air qui se ballade dans tous les sens, c’est douloureux ! Et puis ben ils ont vu que j’avais rien du tout, quoi, comme, comme 13 mois avant. Mais ils ont jamais été capables. Les quatre litres d’air, qu’ils m’ont enfilé, ils ont jamais été capables de les aspirer ! Ils ont pas de système pour les enlever : « ah ça partira tout seul ! » La preuve. J’ai un joli ballon, j’en avais pas du tout avant, puis j’ai mal ! Alors ça je les félicite, je les félicite vraiment quoi *(ton ironique)*! Entre les côtes cassées, les, hein, très efficaces !

**C : Des péripéties.**

**A :** Ah X *(hôpital)*, j’ai fait 17 semaines, mais y a pas une semaine qui. Deuxième fois par exemple, c’était X *(mois)* l’année d’avant, donc 2015. Toujours là au X *(unité de l’hôpital)*, ils ont réussi à me mettre près de la fenêtre, les jours où y avait la bise. Ma compagne et puis ma, sa fille ont tous les deux les cheveux longs, comme vous. Elles étaient en face de moi, j’étais le lit près de la fenêtre, elles étaient en face de moi, elles avaient les cheveux à l’horizontal. Mais les fenêtres étaient fermées ! Mais elles avaient les cheveux à l’horizontal, tellement y avait le courant d’air qui passait d’une fenêtre à l’autre. On appelle le médecin, il était là, il est venu voir, j’ai dit : « vous pouvez pas me déplacer, parce que je vais choper une bronchite ». ; « ah c’est pas possible on va pas ». Y avait le lit d’en face, mais à, une place de la fenêtre, donc à trois mètres, était libre ! « Ah on peut pas vous déplacer, ça fait du boulot pour les infirmières ». C’est très bien. Je suis ressorti trois jours après. J’avais une bronchopneumonie ! Et j’ai refait trois semaines d’antibiotiques ! Juste parce que monsieur voulait pas déranger les infirmières, pour qu’on me déplace d’un lit à l’autre. Y a, y a, puis des trucs ! Mais j’ai appris la patience, là-haut. J’ai, appris à aimer les infirmières et puis les, les aides, et les aides. Parce que c’est des personnes qui ont un dévouement et une gentillesse extraordinaire. Je suis tombé sur une femme, une femme qui faisait les nettoyages là. Puis je disais mais, j’avais mal, à cause de mes côtes, je disais : « nom de bleu, mais foutez-moi du X *(crème)* dans le dos ! » Je sais que c’était un bon, bon truc contre les douleurs musculaires, et contre l’arthrose. Puis, l’infirmière elle arrive, tac, tac, deux minutes, xxx. Puis y a la nana qui rigolait, je voyais qu’elle avait un grand sourire. Puis elle m’a dit : « vous êtes d’accord moi je peux vous, vous mettre, comme il faut ». Alors elle dit : « oui, mettez-vous à plat ventre ! » Elle me l’a mis, mais elle a mis une demi-heure pour l’étaler. Elle m’a fait un massage, mais les montés, les petits roulés, et tout ! J’ai dit : « vous faites quoi ? » ; « ah mais, on m’a, j’ai pu étalé plusieurs fois là, et des pommades, puis on m’a dit que j’avais des mains d’or. Alors maintenant le samedi, je prends des cours de massage ». Puis elle est restée là une heure après son, elle avait fini son boulot ! Une heure après, elle était toujours avec moi, en train de, de me masser, puis de m’expliquer son, sa démarche. Une petite portugaise, une cinquante d’années, qui paie pas de mines, et puis qui a un coeur gros comme ça ! Ça, ça m’a, ouais.

**C : Ça vous touche.**

**A :** Ouais j’ai appris, là-bas j’ai appris à, à, à pleurer de bonheur, tellement les gens ! Les, les docteurs, je leur trouve des excuses, parce qu’ils ont un tel stress, ils font de tels horaires, j’ai discuté avec deux, trois, deux, deux, deux, trois jeunes toubibs, les, les dames qui étaient par-là. C’était pas possible. Tu crèves de mal, tu sais que la dose de morphine elle est de l’autre côté dans le tiroir-là, puis, alors c’est l’aide qui va demander à l’infirmière, qui demande au responsable d’étage, qui doit demander au médecin, t’as ta dose de morphine, quatre heures après ! Alors tu sais que t’as droit, à, les doses toutes les six heures, 60 millilitres. Puis quatre heures après, ils viennent avec 20 millilitres : « ah j’ai pas le droit de donner plus ». J’ai dit : « mais vous regardez dans mon dossier, c’est marqué : « quatre fois 60 par jour ». Ça fait quatre heures que j’aurais dû avoir 60, puis vous venez avec 20 ! ». Alors là je m’énerve, et puis je pousse une gueulée. Et puis après ils arrivent, puis ils me foutent sous calmants, parce que je gueule trop ! Mais ils me donnent pas d’antidouleurs. Ça a été comme ça pendant des semaines ! Au niveau, au niveau des antidouleurs, je demande un médicament, mais il arrive, mais des heures après, des heures après ! Ça c’est, une gestion pour ça qui est, avec leur système pyramidal, où l’infirmière n’a pas le droit de prendre des décisions, alors que elle a le dossier, elle sait, y a un retard, parce que ça a été loupé par le, la série d’avant qui donne les médicaments, ils ont été trop stress, ils ont loupé un malade, parce que ça leur arrive : « ah celui-ci il est moins malade, on est pressé, on passe à la chambre suivante ». xxx, puis tu, puis tu vois la tête du monsieur qui est là, qui, qui retient pour pas pleurer tellement il a mal, puis on lui, on considère qu’il est pas trop malade, qu’il peut attendre un moment. J’ai appris la patience, mais j’ai appris la révolte, aussi. Et ça, ça m’a, ça m’a peiné, de voir qu’en Suisse on peut arriver d’avoir un état de, de je-

m'en-foutisme. Et ça, c'est de la part des dirigeants. Pas de la part des soignants ou des infirmiers, mais de la part des toubibs, de la part des toubibs. Comme y en a une qui a voulu me faire une, prise de sang artérielle, là. La première fois qu'elle me l'a faite, elle a piqué 16 fois, avant de trouver l'artère. Puis trois jours après, elle revient : « ah j'ai pas pris assez de sang, je dois recommencer ». J'ai dit : « c'est vous qui avez l'intention de me recommencer une prise de sang artérielle, quand vous savez pas les faire ?! » ; « oh, je sais les faire, j'ai réussi ». ; « oui, vous avez piqué 16 fois, vous avez mis une heure et quart, pour réussir à trouver l'artère. Y a tout le monde qui rigolait, je sais pas si vous. Regardez les autres patients ». Les autres patients : « oui, oui, oui, oui, on a bien vu ». Elle est partie, elle est venue avec un autre. J'ai dit : « non, désolé. Je suis à deux jours de sortie, je vais bien. Si y a quelqu'un qui fera un, un contrôle, une prise de sang artérielle, ce sera mon médecin traitant, mon pneumologue ! ». J'ai été trois jours après chez le *Docteur D.*, il m'a simplement fait mettre un bout de mousse comme ça, pour que le, le poignet tombe bien. Puis quand le poignet tombe bien puis qu'il est décontracté ben, l'artère ici dans le petit creux, elle sort, et puis en deux minutes, c'était fini ! Ils piquent une fois l'artère, je leur ai dit : « nom de bleu, mais mettez sur un, un », ; «ah non, faut que le poignet soit bien à plat ». J'ai dit : « madame, vous apprenez quelque chose. Quand le poignet est en suspension, ben l'artère elle sort tout de suite ». Alors après j'ai été lui montrer, trois jours après j'ai été à l'hôpital, j'ai dit : « madame, vous avez un petit moment ? ». J'ai dit : « regardez voir, mettez le poignet comme ça, puis regardez-voir là. Voyez ce qui vient, ce qui sort, là ce qu'on voit battre ? Ben le poignet comme ça là, l'artère elle est visible, elle sort d'elle-même ! » ; « ah, vous êtes sûr ? », j'ai dit : « mais maintenant quand vous la voyez là, vous pouvez me faire une prise de sang ». ; « ah je vais essayer ». J'ai dit : « non, non merci ». J'ai dit : « je viens d'en avoir une, voyez. Je viens d'en avoir une là y a cinq minutes, c'est bon ». Mais là vous, « essayez d'apprendre, essayez d'apprendre. Si vous savez pas, demandez. Mais, essayez, ayez le ». Je leur ai dit : « mais ayez la, la capacité d'apprendre, puis d'écouter, ça vous aidera beaucoup dans le travail ». Ça je leur ai dit, hein !

**C : Vous avez appris la, la patience et la, et la révolte aussi, comme vous disiez.**

**A :** Ouais. Oh puis j'ai bien rigolé, c'est avec le chef des inf', le chef de l'école d'infirmerie. C'est un monsieur qui est assez imposant, et tout. Puis bon, on a sympathisé dès le, dès le, dès mon premier séjour à l'hôpital, en X *(date)* 2014, on a sympathisé ! Et puis, il a vu une fois que il me faisait les prises de sang, sans problèmes, comme ça ! Alors il est venu quelques jours après, il m'a dit : « écoutez je suis, je suis le chef ». J'ai dit : « ouais, t'es X *(prénom)*, t'es, t'es, t'es, t'es, t'es, t'es le chef des, des, des, des, des aspirants ! ». Il m'a dit : « ouais, c'est, c'est bien ça ». Il a dit : « mais toi tu, tu, tu, tu xxx ». Alors on s'est tutoyé tout de suite ! J'ai dit : « toi tu fais faire les piqûres xxx. Toute façon je suis sous morphine, donc je sens, je sens un peu moins ». Et puis j'ai dit : « si c'est pour rendre service, par rapport à tout ce qu'on me, à tout ce qu'on m'apporte ». Parce que bon, j'ai, y a des conflits et des problèmes, mais à côté de ça y a tout le positif, tout ce qu'on m'a fait de bien ! Tout, tout l'évolution de la maladie, les, les bons contrôles, les bons médicaments qu'on a, ça a mis longtemps, mais c'est venu petit à petit ! Parce que maintenant le, le suivi médicamenteux que j'ai, où depuis le X *(mois)*, on a fait une grosse descente, j'avais 14 ou 15 médicaments avant. Maintenant j'en ai plus que six. Donc j'ai un très, très bon suivi. Alors il me dit : « bon écoute, comme je suis chef, j'aimerais bien, si je peux venir de temps en temps avec, avec, cinq, quelques infirmières, quelques stagiaires pis pour faire des petits contrôles ! ». J'ai dit : « oui bon ben d'accord ! ». Alors il vient trois jours après avec six, six dames. Alors j'ai été très étonné d'abord parce qu'y avait deux, trois femmes de 25 ans. Et toutes les autres dames avaient plus de 50 ans, certaines plus, plus de 60. J'ai discuté avec : « oh, j'étais 30 ans banquière, et tout ! J'ai repris ma retraite anticipée à 55 ans. J'ai bien travaillé, mais je sais pas quoi faire de mes dix doigts, alors je fais l'école d'infirmerie ». Et les trois quarts c'était des femmes comme ça. Trois quarts des infirmières, de plus de 50 ans c'était des femmes qui ont fait leur carrière et tout, mais qui ont envie de donner quelque chose. Et ça, ça m'a ému, mais ça m'a. Alors après ben le contrôle, le contrôle des oedèmes, contrôle des trucs, à l'époque j'avais pas d'oedèmes, bon maintenant je suis sous X *(médicament)*, parce que j'en ai beaucoup, à force de prendre le X *(morphine)*. Alors ils me contrôlaient les oedèmes et tout, puis après bon, écouté le, écouté les poumons, puis écouté le coeur. Alors elles se mettaient en face de moi, elles venaient toutes les six après une à écouter le coeur, elles appuyaient, elles disaient : « écoutez bien ». J'ai dit : « ouais, ah vous m'aurez jamais, vous me ferez jamais mal, vous me ferez jamais pleurer, j'ai pas de coeur, j'ai pas de coeur, j'ai pas d'émotions, j'ai pas de coeur, vous m'aurez jamais ! » Puis les nanas : « mais c'est vrai, il a pas de coeur ! On n'entend pas son coeur ! ». Et là, lui il rigole, il dit : « vous avez consulté son, son dossier ? » ; « ah non ». On regarde le dossier : « grosse masse graisseuse de deux centimètres autour du coeur ». On sait pas si c'est de la graisse ou, ou des tumeurs, des vieilles tumeurs et tout, enfin toujours est-il que mon coeur est entouré d'une grosse masse et que, faut, faut vraiment appuyer très, très fortement, puis qu'y ait pas de bruits à côté, pour entendre un petit peu. Ce coup-là, on l'a fait, ben il venait toutes les semaines, avec cinq, six personnes. Et puis à la fin ils, ça c'était le premier séjour, il m'a dit :

« mais puisque t'es là, est-ce qu'on peut, je peux leur montrer comment on fait une piqûre ? ». J'ai dit : « ouais ! ». Alors il dit : « bon ». Il leur montre, il fait la piqûre. J'ai dit : « bon ben si, puisqu'elles sont là, maintenant il faut, continue ! Ma veine est assez longue, je crois ! ». Alors je lui, je lui ai fait faire les six, une après, sur la veine là, puis après sur la veine là, puis après ici. Les, les six ont, puis il était là, il a dit : « mais c'est pas possible ! ». J'ai dit : « mais tu reviens demain avec un autre groupe, ça me gêne pas ! ». J'ai fait ça toutes les semaines, trois fois par semaine quoi, euh 8 ou 10, <u>chaque fois</u> !

**C : De belles rencontres aussi.**

**A :** Et mieux, c'est à chaque fois des, des, des femmes qui ont, qui ont des parcours de vie, et tout. Y avait quelques jeunettes de temps en temps, mais <u>très rare</u> ! J'étais vraiment très, très étonné, la, la, la, la majeure partie, c'est des, des, ouais on peut dire des femmes d'âge mûr, quoi ! Vraiment des, des femmes qui ont, qui ont fait leur vie, puis qui sont là, qui ont envie de donner quelque chose. Ou une, une, une femme de ménage espagnole ! Dit: « ah, j'ai, pour pouvoir faire l'école d'infirmière, j'ai passé la maturité du soir. J'ai mis cinq ans. Après mes heures de nettoyage, j'ai mis cinq ans pour passer la maturité du soir. Puis ben mon, mon, mon mari a assez d'argent pour que je puisse aller étudier à X *(hôpital de réhabilitation)*, sans avoir besoin de ramener la paie. Alors je fais l'école d'infirmière ». Une femme, une femme de chambre, qui avait pas été à l'école, qui a, donc qui a, qui avait pas fini la scolarité obligatoire au Portugal. Mais qui a, qui, qui a fait la matu par correspondance pour pouvoir aller étudier.

**C : Oui. Et reprendre plus tard.**

**A :** C'est, y a des parcours de vie, qui sont, qui sont vraiment aberrants, quoi !

**C : Donc voilà, malgré.**

**A :** Hein ?

**C : Malgré quelques aussi conflits, ou mésaventures comme vous disiez, [y a eu aussi des choses qu'on a pu vous apporter, par rapport à, à la maladie.**

**A :** Ouais ! Ouais ! Ah oui, oui, oui !] Ah non mais attention il faut bien être clair alors, je retire beaucoup plus de, de <u>bonheur</u> que de, on peut dire que de, que de, de râleries, quoi de. Ça, j'ai donné les exemples parce que, y a ces exemples, c'est dommage que pour certaines choses ils aient pas la qualité d'<u>écoute</u>, donc y a ces, ou des, des trucs d'inattention, parce que, la, la jeune femme aux urgences, qui m'a fait l'électrocardiogramme, puis qui m'a mis, parce que c'était en vitesse, puis qui a laissé, c'est la prise, tous les fils, xxx, qui m'a laissé dans le dos, par hasard parce qu'elle était pressée, puis que, le fait d'être resté trois heures comme ça. Elle était gentille cette jeune femme, c'était, j'ai discuté avec elle, de la moto, du bateau, et tout. On a, xxx je l'ai trouvé très sympathique mais, elle a oublié, c'est un truc d'inadvertance, moi ça m'a cassé deux côtes, j'ai eu mal, j'ai encore mal maintenant une année, une année et demi après ! Mais elle l'a pas fait exprès, puis ça a, ça a été une vitesse, ça va tellement. J'ai passé un bon moment avec elle, moi j'aurais, j'ai, ok, j'ai toujours mal maintenant, mais je me rappelle plus des bons moments que j'ai passé avec elle ! Les douleurs elles sont, bon des douleurs j'en ai, j'en ai partout. Donc j'ai fait xxx, j'étais comme architecte, mais j'ai travaillé sur des chantiers pendant 30 ans. Et avant de faire les chantiers, j'ai fait cinq ans comme manoeuvre sur les toits, parce que ben, j'ai eu mon diplôme en X *(année)*, quand y a eu les premières crises pétrolières. Donc c'est là qu'y a eu les premiers chômeurs en Suisse. Puis comme j'étais architecte, ben à l'époque les architectes, ils avaient plus de boulot du tout. Mais j'étais dans, dans la, alors j'ai fait d'abord, guide touristique, après j'ai fait taximan. Puis après je suis arrivé sur les chantiers. Alors j'ai tout fait, j'ai fait plâtrerie-peinture, maçonnerie, puis après j'ai fait la couverture, ferblanterie. Et ça j'ai trouvé, j'ai trouvé un boulot magnifique. Alors j'ai fait ça pendant quatre ans, dans, deux, deux entreprises différentes. Et après je, j'ai trouvé du, là, là j'étais en X *(canton)*. Après j'ai, j'en avais marre, et puis, ma, ma compagne aussi elle en avait marre de, de rester enfermée dans, un truc où y a pas beaucoup de possibilités d'évolution, on va dire, dire ça comme ça gentiment. Et puis on a, on a trouvé la, la, la chance de venir à X *(ville)*. Pour venir à X *(ville)*, j'ai accepté un travail, mais 300 francs plus qu'en X *(canton)* comme architecte. Parce que je connaissais pas les tarifs, puis quand je suis arrivé à X *(ville)*, le troisième mois je me suis rendu compte qu'il me manquait 1'000 francs, au minimum dans le budget pour réussir à vivre normalement à X *(ville)*. Et puis bon j'ai demandé de, j'ai discuté avec les patrons. Ils ont dit : « nous, on t'a, on t'a engagé comme ça, c'est parce que t'avais un petit salaire, quand on t'a engagé ! Autrement t'as pas les, les qualifications ». J'ai dit : « bon, c'est bien gentil ». Je leur ai cloué le bec deux mois après, je leur ai cloué le bec comme il faut, juste avant de partir. Mais entre-

temps, j'avais connu un, un gars qui était responsable dans une entreprise de couverture, qui avait une quinzaine d'ouvriers, il avait besoin d'un technicien. Je lui ai dit, expliqué un peu mon parcours. Il m'a dit : « ok, tu commences demain ». J'ai dit : « non. Soyons corrects, j'ai beau avoir que trois mois, j'ai trois mois de délai de congé quand même, je donne les trois mois de délai de congé ». J'ai donné les trois mois de délai de congé. Puis pendant ce temps mes patrons sont partis en vacances les deux en même temps. Et j'avais un grand immeuble, X *(rue)*. J'ai fait tous les dessins d'architecture et tout. Ils reviennent trois semaines après, ils disent : « oh ben maintenant il faut qu'on aille voir, ingénieur en chauffage, ingénieur en ventilation, ingénieur sanitaires, pour faire les plans de chauffage, sanitaires, et ventilation ». J'ai dit : « mais messieurs, vous êtes des petits cons ! » ; « comment tu nous causes ?! ». Ceci, cela. J'ai dit : « mais regardez là. Vous avez tous les plans de chauffage, tous les plans de ventilation, électricité, sanitaires, ils sont tous faits ! ». Nous quand on apprend un boulot, on va, on apprend tout. On n'apprend pas juste à dessiner les murs, on apprend à placer des plages électriques, et tout. Alors ils m'ont, ils m'ont gardé, ils m'ont prolongé le contrat, on a discuté avec l'autre patron, ils m'ont gardé pour que je finisse l'immeuble et tout. Mais tout l'immeuble, y a, 14 francs de frais de régie, donc pour, en plus, un truc en plus par rapport aux devis, sur l'ensemble de tous les corps de métier, pour déplacer une prise électrique qui était un petit peu trop près d'un radiateur. C'est la seule chose. Tout le reste, c'était parfait ! Ils ont jamais vu ça ! Et ils ont économisé tous les frais d'ingénieurs, tous les plans que j'avais fait, étaient corrects ! Alors là les, il était prof à, à l'école technique du soir, alors là il m'a pris, puis il m'a, il m'a présenté à ses étudiants, pour dire : « ben voilà comme un étudiant. Je regrette qu'il parte ». Là ils m'ont, ils voulaient me doubler le salaire. J'ai dit : « non. J'ai pas voulu avant, maintenant, j'ai trouvé autre chose ». Et puis bon, j'ai été bosser sur les toits avant. Mais comme technicien, ben tu dois montrer l'exemple, quoi. Donc tu dois montrer aux ouvriers ce que tu sais faire. Ce qui fait que j'ai bossé énormément, et que je me suis foutu énormément le corps, enfin sous stress, sous dangers, accidents, et compagnie, donc. J'ai mal aux chevilles, aux genoux, aux hanches, aux épaules, aux coudes, aux poignets.

**C : Lié à votre travail.**

**A :** Y a tout ! Y a tout qui fait mal, parce que j'ai tout. Ben je suis tombé une fois du septième étage. Bon, j'ai de la chance, y avait un tas de sable dessous. Alors je me suis juste fait un écrasement des talons, puis des hanches. Mais, à l'époque, on savait rien, hein. Bon maintenant j'ai mal aux hanches. Donc, mais je regrette pas, j'ai eu une vie passionnante ! Bon, comme tout, comme tout un chacun. Mon patron il a beau être un copain, le jour de mes 50 ans, il m'a pris à part, il m'a dit : « désolé, maintenant je dois payer 20% de, de salaire pour le deuxième pilier, alors qu'avant je payais que 7 ». Comme on avait fait un accord, et tout. Donc j'ai un deuxième pilier qui est pas, qui est pas énorme par rapport au salaire que j'avais : « toi, je te fous dehors. Salut ! Voilà ta paie, t'as les trois mois qui sont là, tu pars immédiatement ». Et en 25 ans sa boîte a passé de 7 ouvriers à 45. Et moi j'avais 45 ouvriers tout seul. Y avait pas de contremaître. C'est moi qui gérais tous les ouvriers ! Lui il faisait le côté administratif, et il cherchait, il cherchait des contrats. Moi je faisais tout le reste ! Les livraisons, les contrôles, bon apprendre aux apprentis, et tout. Quand je suis, quand je suis parti pour gérer les 45 bonhommes, il a dû prendre sept techniciens, sept ! Moi je faisais du boulot de sept personnes à la fois.

**C : Donc il a dû, dû vous remplacer par plusieurs, oui, oui.**

**A :** Bon, moi ça m'a été une baffe terrible, c'était du mobbing typique ! Dépression, j'ai fait une année et demi d'assurance, ben je déprimais complètement. Après c'était le chômage, puis après les, les petits jobs et tout jusqu'à ce que petit à petit ben je ré apprenne le dessin, mais sur ordinateur ! Dessin d'architecture sur ordinateur. Et puis après j'ai eu la chance de tomber ben dans un bureau d'architectes, de jeunes architectes d'une quarantaine d'années, qui avaient besoin d'un gars d'expérience, qui m'ont engagé pour mon expérience. Par contre, alors malheureusement c'est chez eux, au bout de cinq ans et demi que j'étais chez eux, ben que j'ai fait la transformation de cette villa X *(nom)*. Et puis, c'était au X *(mois)*, et puis je suis arrivé nom de bleu un jour ! J'avais au centimètre, j'avais une vingtaine de points rouges ! Mais tout le corps ! J'étais couvert de points rouges, une vingtaine au centimètre. Alors je vais le X *(date)*, je me rappelle toujours chez le, le spécialiste de la peau là, il était à X *(lieu)*. Il m'a dit : « oh ». Puis je, je m'habille, je me déshabille derrière le rideau, puis lui est à côté, puis il dit : « ça fait longtemps que vous soufflez comme ça quand vous vous déshabillez ? ». Je dis : « oh ça fait quelques mois que, que, que je respire mal ». Il m'a dit : « ouais, vous avez aucune maladie de la peau. Vous respirez plus par les poumons, vous respirez par les pores de la peau, seulement vous respirez tellement que vous avez tous les pores qui sont enflammés, vous êtes en train de vous étouffer. Alors vous allez tout de suite chez *Docteur F.*, qui est dans l'allée d'à côté, et tout. Il m'a vu, il m'a fait faire immédiatement un scan. Il a dit :

« non, y a pas de, de tumeur cancéreuse. Par contre, alors vous avez plus que 32% de poumon ». A cause des, bon on savait pas, à cause des, des, des, des tumeurs qu'y a dedans, on savait pas que c'était un champignon, puisqu'on a découvert ça seulement cette année ! Alors il m'a mis en arrêt de travail, immédiatement, et deux mois et demi après je touchais l'AI à 100%, donc ce qui était excessivement rapide, quoi. xxx. Puis après bon l'AI a essayé de me replacer. J'ai fait des cours de l'AI, des cours de replacement de l'AI, ils voulaient me replacer comme dessinateur, architecte. J'ai dit : « excusez-moi, je sors d'en prendre. J'ai été dessinateur, architecte. J'ai été licencié, parce que même ce boulot-là, je peux plus le faire ». Alors, ils m'ont dit : « vous pouvez arrêter de faire ». C'était des cours de replacement, on était une vingtaine de personnes. « Vous pouvez arrêter de faire les cours, c'est inutile, et tout ». Puis moi ça faisait trois, quatre jours, j'étais là avec cette équipe, et puis on discutait bien, y avait un bon, j'ai dit : « non. Y a encore trois semaines, je vais faire encore les trois semaines de cours ». Il me dit et puis le prof et puis le directeur du centre de réadaptation, ils, ils viennent me voir ils disent : « mais qu'est-ce qu'il vous prend ? Pourquoi ? Vous pouvez arrêter, et tout ». J'ai dit : « mais écoutez, vous avez parlé au départ la théorie des groupes, ceci, cela. Là on a un groupe qui est soudé, moi ben, j'ai l'habitude de causer, j'ai, j'ai l'habitude, je suis un meneur d'hommes, ça a toujours été comme ça ! ». J'ai dit : « si je pars, vous faites comment ? Vous trouvez qui pour me remplacer pour, pour orienter les discussions, pour créer un dynamisme de, de relationnel, puis des trucs ? ». J'ai dit : « est-ce que vous voyez quelqu'un ? », il a dit : « non, dans le groupe, y a personne » ; « ben je reste ! » Du moment que je sois enfermé à la maison, à étudier le, parce que bon c'est là que je, je m'étais payé un ordinateur privé, à étudier le, l'électronique et compagnie, puis les, à, à travailler, évoluer sur, sur l'ordinateur, ou que je sois ici en train d'évoluer avec, faire connaissance avec des gens, et tout. Quand je partirai d'ici dans trois semaines, j'aurai le plaisir d'avoir connu telle et telle personne, échangé les parcours de vie, d'avoir, de m'être enrichi ! Et puis d'avoir permis au groupe de, de continuer son chemin !

**C : Donc vous avez continué comme ça.**

**A :** J'ai été jusqu'à la fin du cours, et tout. Chaque fois qu'y avait un questionnaire : « qu'est-ce que vous voulez, désirez faire comme travail ? », j'ai marqué mais : « déjà replacé définitivement à l'AI ». Puis bon, ils ont jamais posé plus de questions ! Ça a été accepté dès le départ, que je sois impossible à replacer tellement j'étais malade ! Mais j'ai tenu jusqu'au bout ! Alors après j'ai dit : « bon ben voilà, maintenant on va ». J'ai organisé une sortie, pour aller tous ensemble manger, manger un truc, quoi ! Alors, alors on va, la vingtaine, c'était le restaurant X *(nom du restaurant)*, c'est juste en bas de, du X *(lieu)*, où on était. Et puis, y avait un gars frontalier qui était là avec nous, qui devait être replacé mais qui avait, qui avait l'intention d'ouvrir un, un restaurant, un bistrot, et tout. Puis, il m'avait l'air très sympathique, puis on avait discuté, il était venu manger à la maison, parce qu'à l'époque, j'habitais à X *(lieu)*, à 300 mètres de, de l'école, donc il était venu discuter deux, trois fois à la maison, et tout. D'un coup je le vois disparaître, et tout. Et puis, tout d'un coup y a un petit mot qui arrive, un joli petit gâteau qui arrive, avec un joli petit mot : « je vous ai beaucoup aimé, mais là je suis occupé, je dois partir ». Et tout. J'ai dit : « bon d'accord, salut ». Puis il me salue. Puis après je dis, j'appelle la, la, la dame : « ah vous nous amenez l'addition. On va se partager tout ça ». ; « ah non, non, non, monsieur, monsieur ». Il s'appelait X *(prénom)* là : « il a tout offert, parce que vous étiez une équipe géniale ». C'est un, un petit retour, moi j'ai fait un geste, là y a un autre geste en retour, c'est des, d'être tombé à l'AI c'est une richesse ! J'ai connu énormément de gens !

**C : En étant à l'AI.**

**A :** Oui, en étant à l'AI. Puis étant au chômage, aussi.

**C : Vous avez fait des, des rencontres, et.**

**A :** Ouais !

**C : Et là vous disiez que, par rapport à l'AI, vous êtes aussi en train de faire des démarches pour, pour votre femme, pour.**

**A :** Oui. Enfin, c'est pour moi, disons que, j'ai déjà le degré d'impotence léger, donc je touche déjà 400 francs, 480 francs par mois d'aide supplémentaire par rapport, parce que je vis, je n'ai que l'AI, l'AI pour vivre, parce que ma fille est encore aux études, et puis ben, bon elle termine l'année prochaine. Alors je donne l'AVS, la, la partie de rente AVS que je touche, 2'000, 2'200 je crois, je la verse directement sur le compte de ma fille. Puis moi il me reste que 1'700 francs par mois pour vivre. Mais, je, j'arrive, j'arrive juste, quoi. Les frais fixes, c'est 1'500 francs, j'ai 200 francs pour, pour manger, mais, je bois pas, je fume

pas, donc, évidemment ça, et puis avec l'aide de ma compagne, c'est plus qu'assez ! Mais, comme j'ai expliqué avant, ma compagne ne peut plus donner d'aide dans sa famille, et puis ben j'aimerais pouvoir, ben payer la, la, la femme, la compagne, la, ah, la nounou des, des gamins, je trouve que c'est à moi de la payer, puisque leur mère, elle, elle, la mère de mon beau-fils peut pas avoir, peut pas être chez, chez lui à cause de moi, ben je trouve normal que ça soit moi qui la paie, quoi. Alors, c'est un peu dans ce sens-là que j'aimerais bien que l'AI reconnaisse mon impotence. Surtout que c'est une impotence totale, quoi. Je suis plus capable de m'habiller, je peux pas me laver, c'est-à-dire, me laver je dois, je suis assis, puis elle me passe la lavette un peu partout, mais. Même en me mettant sur six ou huit le, le débit d'oxygène, si je fais juste ça comme ça trois fois, en appuyant un peu je suis, je m'essouffle et puis je tourne de l'oeil, et puis je suis, je suis pas bien, donc. Et je suis à, je peux plus sortir ! Si je sors, pour aller au physio, elle vient avec la voiture le plus près possible de, de la porte en bas, parce que derrière la barrière où y a les, les, les vélos et les motos, puis elle vient près du quai de chargement, comme ça j'ai, j'ai 12 mètres à faire depuis la porte de l'ascenseur jusqu'à la voiture. Puis après pour aller, au cabinet médical, ben j'ai, j'ai la, le trottoir, j'ai quatre mètres à faire et puis je suis, je suis au cabinet, je suis attendu sur la table de massage. C'est les seules choses que je peux faire ! On a voulu récemment aller à Ikea pour acheter des trucs, j'avais deux bonbonnes d'oxygène, j'en avais mis une devant, une derrière, puis je me suis fait un masque parce que, en cas de, de nécessité, ils expliquent qu'avec le masque ça prend plus l'oxygène que quand on est sur six ou sur huit, parce qu'y a plus. Alors comme on peut être que sur six avec, avec une bouteille là, ce petit Stroller qu'on met sur le dos, j'ai, j'en ai deux. Et puis j'ai fait un masque où j'ai fait un deuxième trou dans le masque, puis j'ai mis un, un tuyau supplémentaire, donc j'ai un appareil qui donne un débit de six, non, le débit de six il est dans le dos. Puis l'appareil que j'ai sur le ventre, je le règle trois, quatre, parce que c'est visible, enfin j'ai le bouton. Alors j'ai le masque, et puis j'ai un, le masque me donnait six, huit, dix, douze de débit. Chose que je peux pas faire avec, avec des, avec des lunettes, quoi. Alors pour me balader je mets ça. Bon, les gens sont tout étonnés que je, deux bouteilles, le masque, c'est clair que c'est, c'est spectaculaire ! Puis les gens, y a des gens qui viennent carrément, je dis : « non ». Simplement, une bouteille d'oxygène me suffit plus, je suis obligé de passer à deux bouteilles, le masque. Mais bon quand j'ai montré à mon médecin traitant, il est venu l'autre fois quand j'ai traficoté le masque, pour avoir un masque avec plus de, plus de débit. C'est juste génial, mais c'est juste catastrophique que j'en sois arrivé à ce stade-là ! Que j'aie pas encore été greffé, maintenant, quoi ! Et tout ça parce qu'ils ont pas réussi à identifier le, ce qui se baladait dans, dans les poumons, quoi.

**C : Donc là vous attendez toujours [pour la greffe ?**

**A :** Ah ben maintenant, ben maintenant] j'ai, demain j'ai le rendez-vous donc de coordination finale, aux X *(hôpital)*. Après je serai officiellement sur la liste des greffes. Bon après, le problème c'est que c'est un, comment ça s'appelle, je me rappelle jamais ce nom-là. Calcul mathématique qui est fait par l'ordinateur, ah, logarithmique, voilà, où ils mettent dedans ben la grandeur des poumons, le taux de, toxicité, l'âge. Et puis ça fait, il calcule, et puis en fonction du calcul, ben vous êtes le premier, le deuxième ou le cinquième de la liste, quoi. Et puis, je sais pas du tout quels éléments vont être pris en compte, à quel, quel truc, quel, et puis où ça va me placer dans la liste, quoi. Si je suis en premier ou en deuxième, je peux attendre peut-être que deux, trois mois. Si je suis en dernier de liste, je peux attendre une année ou deux ans. Mais dans une année ou deux ans, je serai plus là. Au, à la vitesse où ça se dégrade, y a beau un moment, soit je m'étoufferai, puis je manquerai d'air par, à cause d'une pollution, n'importe quoi, soit je me serai suicidé avant, parce que y a, y a un moment c'est plus <u>viable</u> ! C'est trop, c'est trop, c'est trop <u>angoissant</u> ! En plus j'ai pas le droit de prendre des trucs contre les angoisses, pour pouvoir être opéré, parce que c'est, c'est en contradiction avec les, les, les immunosuppresseurs. Donc, il suffit qu'il reste un petit peu, xxx dans le sang encore pendant deux semaines, les immunosuppresseurs fonctionnent pas au départ, et puis votre poumon, il est foutu tout de suite, donc. Oui, j'arrête, parce que il faut le faire, <u>mais</u> ça a des conséquences à beaucoup de niveaux, quoi, c'est ! Enfin c'est le, j'ai vraiment l'impression c'est le serpent qui se mord la queue quoi, y a. Puis y a personne qui arrive à dire : « ben voilà maintenant on décide, on le fait, on le met, en tête de liste, on fait quelque chose pour qu'il soit, ce soit rapide, quoi ». C'est pour ça que demain, j'espère que demain ils pourront écouter un peu ce que j'ai à dire, pas seulement eux le raconter, l'opération, j'ai déjà passé une fois au mois d'avril, à l'époque j'avais encore trop de tabac puis d'alcool dans le sang, ils ont refusé de, de me prendre à cause de ça. Parce que personne n'avait expliqué qu'il fallait pas boire d'alcool dans le cadre d'une greffe. Donc je ne bois plus de vin rouge et d'alcool fort déjà depuis cinq ans ! Je buvais que du, je buvais de la bière, je buvais deux bières par jour à l'époque ! Mais personne m'a jamais dit que même deux bières par jour, c'était <u>déjà trop</u> ! Puis là ils m'ont dit : « oh mais vous avez un degré d'alcool dans le sang qui est incompatible avec une greffe ». C'est bien gentil d'avertir que. Depuis X *(date)* 2014, jamais personne qui m'a rien dit ! Donc, ils disent : « ouais, les trucs de, de, d'information ». Et tout. Je dis : « mais vous regardez dans la liste

d'information si une fois y a le mot, y a le mot éthylique qui est marqué dans les trucs que vous m'avez donné ». Je dis : « c'est maintenant, dans votre rapport du X *(mois)* que ça apparaît, enfin du X *(mois)* plutôt ». J'ai trouvé ça un joli gag, quoi.

**C : Vous auriez aimé avoir plus [d'informations ?**

**A :** Mais ils m'aurait dit] ça avant ! Quand ils m'ont dit ça j'ai arrêté immédiatement de boire de la bière ! J'avais plus touché une bière depuis, depuis le X *(date)*, le jour où ils m'ont dit ça, j'ai plus jamais touché une goutte d'alcool ! Ça m'a jamais, déjà quand j'ai arrêté le vin et la, et les alcools forts à l'époque, alors j'étais un buveur régulier, ça m'a jamais manqué ! Puis mon médecin traitant il dit, il comprenait pas ! Il disait : « tu devrais avoir le, comme tout le monde ! Et ça fait 30 ans que tu bois, tu devrais, tu devrais avoir le ». J'ai arrêté d'un jour à l'autre, j'ai eu aucun, aucun manque, aucun malaise ! J'ai arrêté la bière, j'ai eu aucun malaise ! Bon, ils sont étonnés, ils veulent pas croire. Demain j'ai vu dans le truc, demain ils feront des analyses complètes, urine, sang et tout pour confirmer le fait que j'ai tout arrêté. Bon, si ma parole vous suffit pas, moi toute façon j'ai pas peur des piqûres, vous pouvez faire ce que vous voulez, quoi.

**C : Encore faire ces tests-là. Mais c'est, c'est une maladie angoissante, vous disiez.**

**A :** Oh oui !

**C : Surtout que vous pouvez pas prendre des, quelque chose contre l'angoisse.**

**A :** Faut apprendre à serrer les fesses. Mais, *(rires)* c'est un jeu de mot, mais *(il prend une boite de médicaments)*. Constipation chronique, à cause du X *(morphine)*, alors là je serrais les fesses. Je dois pas les serrer moi, je dois plutôt essayer de les déserrer. Enfin y a. En ayant ce type de maladie, vous apprenez énormément de choses. Déjà au niveau du fonctionnement de, de l'hôpital, des fonctionnements humains, des gars qui se croient plus malins que les autres parce qu'ils ont un diplôme un peu plus gros, mais qui sont encore plus cons que les autres, faut le dire franchement ! Le gars le, ah il était fier, il était fier le toubib : « moi je suis cardiologue voyons, je sais ce que je fais ! ». Je dis « ouais, mais vous m'écrasez trop les côtes, vous me faites mal ! » ; « mais j'ai déjà fait des échographies cardiaques, je sais ce que je fais, merci ! ». Trois semaines après je vais faire une, comment il s'appelle… Regarder, faire une, une radio du corps, pour regarder les zones de, comment ça s'appelle, si j'avais assez, les os étaient assez forts, donc. Puis là le gars il ressort, il, je sais pas, un scan et tout, puis le toubib il sort pratiquement en criant : « nom de dieu mais quand vous avez un accident frontal, avec une voiture et puis que vous avez les côtes cassées, faudrait peut-être nous aviser avant, comme ça on vous xxx ». Je dis : « mais j'ai jamais eu d'accident frontal, j'ai jamais eu de côtes cassées, rien du tout ! » ; « ah regardez vos poumons. Deux sur le, deux à droite du, deux à gauche du coeur, une à droite. Vous m'avez fait quoi ces derniers temps ? » ; « oh j'ai fait une échographie cardiaque y a cinq jours, puis j'ai dit au cardiologue qu'il appuyait un petit peu plus fort » ; « vous me donnez le nom du cardiologue, s'il vous plaît ? ». Puis je l'ai plus jamais revu dans les couloirs d'hôpital. J'ai dit, j'ai fait plusieurs échographies, après plusieurs fois chez les cardiologues, je l'ai plus jamais vu dans les couloirs d'hôpital. Je sais pas si, s'il a, il a changé de métier, s'il a fait autre chose ou s'il a été mis sur une voie de garage, mais je l'ai plus jamais vu à l'hôpital. Mais, ah il était fier d'être costaud puis de pouvoir appuyer fort : « on entend peu votre coeur, il faut appuyer fort ». J'ai dit : « oui, d'accord, d'accord ». Trois côtes cassées. Alors quand vous avez les côtes qui frottent une contre l'autre comme ça quand déjà vous devez respirer à fond, pour, pour prendre de l'air et tout, puis que vous avez des côtes qui flottent et tout. Puis que les gens ils mettent, ils ont mis quoi, un mois à peu près, pour admettre que j'avais vraiment un problème, puis que ce serait bien de me mettre quand même, au lieu de me donner des dafalgans, de, de me donner quelque chose de plus fort et tout. Puis à la fin j'ai appelé mon médecin traitant, puis c'est lui qui depuis, parce que j'étais à X *(hôpital)* à l'époque. C'est lui qui depuis X *(canton)* a donné l'ordre de, *Docteur F.*, je me rappellerai toujours ce, ce toubib à X *(lieu)*, pour qu'il me mette sous morphine. Il a dit : « ça suffit, il a des côtes cassées, maintenant vous le mettez sous morphine ». Intraveineuse, j'ai été au lit je pouvais pas bouger, je, intraveineuse.

**C : Oui. Pas mal de traitements. Vous avez beaucoup appris avec [la maladie.**

**A :** Ouais puis là-haut], là-haut à l'hôpital. Déjà ils sont bien à X *(hôpital)*, ils sont géniaux ! J'étais bien ! Je suis X *(origine)*, j'étais content d'aller à X *(lieu)*. Ouais, d'accord. Alors premier jour, comme y avait, à l'époque y avait eu, je sais plus quoi, le SRAS, ou, y avait eu une maladie infectieuse importante qui était, qui était un peu partout, puis ils contrôlaient, ils contrôlaient partout ça. Alors comme je venais de X

*(canton)*, puis qu'ils savaient pas si à X *(canton)* on était plus propre qu'en X *(canton)* ou pas. Alors ils m'ont mis dans une chambre seul. Alors je suis resté dix jours dans un lit sur une chambre seul avec la TV en face. Mais la chambre était toute petite, puis moi j'avais pas, j'avais l'oxygène mais juste direct au mur, donc je pouvais pas bouger. Puis les lits ils sont un peu trop petits. Alors vous avez les pieds, vous mettez les, les, les jambes en grenouille, comme ça. Puis vous bougez pas ! Quand j'ai essayé de me lever dix jours après pour marcher, pour aller à l'autre chambre. Inflammation des, des, ah, des nerfs, les, ah, merde je sais plus, chaque fois que je veux en parler, j'oublie le nom, ça me reviendra. Les, les attaches, sur l'haine des muscles, et tout. Et puis des, comment ça s'appelle, le talon d'Achille. Je marchais, je faisais des pas de 10 centimètres. J'avais une tendinite sur le talon d'Achille, des tendinites ici sur les adducteurs, voilà. Alors ben j'étais censé faire de la réhabilitation musculaire, pulmonaire, donc faire du vélo puis, puis de la marche. Alors j'ai voulu m'entraîner la salle de, de, en bas. Alors j'ai mis 75 minutes pour arriver à la salle, en marchant comme ça *(il mime des pas)*, en crevant de mal. J'arrive à la salle, ils veulent me faire faire du vélo, faire de la marche. Alors xxx, puis j'ai commencé à pleurer, j'ai dit : « non ». Au bout de deux minutes je pleurais. L'infirmière, elle vient voir qu'est-ce qu'il se passe, j'ai dit : « mais je crève de mal ». ; « mais vous êtes sous morphine, vous pouvez pas avoir mal comme ça ». J'ai dit : « regardez ». Les, les tendinites étaient tellement enflammées, des, des talons, c'était rouge. Normalement, une tendinite, ça, ça fait rien quoi, c'est invisible. Là c'était rouge, tellement c'était enflammé ! Alors ils ont mis trois semaines, parce que, normalement on reste trois semaines à, là, mais j'étais tellement mal, qu'ils ont dû me garder cinq semaines. Alors au bout de trois semaines, ils se sont décidés à me donner une crème X *(nom de la crème)*. Une crème, j'en ai ici d'ailleurs, j'utilise encore régulièrement là, pour soulager justement les inflammations, les tendinites, alors je mets sur les coudes, je mets là. Là bon j'ai trop d'arthrose, c'est pour ça que j'ai mis, avec des tensions ça, ça ressort, j'ai mis des, des patchs. Alors j'ai pu mettre ça, puis ça m'a soulagé, ça m'a permis un peu de, de marcher. J'ai pu re marcher pour revenir, revenir ici. Alors au bout de cinq semaines, ce cher toubib, il me dit : « ben je suis désolé, faut qu'on vous renvoie à la maison parce que on peut pas vous garder ici, vous prenez trop de maladies ». Cinq semaines j'ai été, j'ai été là-haut, j'ai chopé cinq maladies nosocomiales, cinq ! Bronchite, la grippe, inflammation du sexe, inflammation de l'anus, tendinite, les adducteurs ! Il m'a dit : « on peut plus vous soigner ! Faut que vous rentriez à la maison, on est incapable de vous garder ». Bon j'ai passé Noël, Nouvel an là-bas. Ils m'ont renvoyé, c'était le X *(date)*, ils m'ont renvoyé à la maison avec un mot pour, pour mon médecin traitant : « incapable de le soigner. Faites ce que vous en pouvez à X *(canton)*. »

**C : Beaucoup de traitements, beaucoup de.**

**A :** Alors, mais des traitements complètement fous, complètement à côté de la plaque ! Ils m'ont donné des trucs, ils m'ont donné pour traiter le, l'inflammation du sexe, je me serais trop lavé ! Avoir un sexe enflammé, mais qui est rouge vif comme ça, puis qui est, qui est deux fois plus gros d'habitude ! Donc l'érection en permanence, 24 heures sur 24, avec toutes les fatigues que ça suppose ! Ah c'était parce que j'étais trop propre ! Quand je suis arrivé à X *(canton)*, j'ai demandé à mon médecin traitant de me faire un petit frottis, et tout. Il a fait le petit frottis, il a dit à X *(entreprise)*, X *(entreprise)* ils sont venus. Deux jours après j'avais de la X *(médicament)*, antibiotiques et tout parce que j'avais un germe corsé ! Non, non, je me suis trop lavé, c'est pour ça. Faire une, faire une analyse pour voir si j'ai un germe, non, non, c'est dans le forfait faut pas, dans le forfait faut rajouter encore le prix de, le prix d'analyse parce qu'on va gagner moins sur le dos du malade. C'est vraiment l'impression que le *Docteur F.* m'a donné, quoi. Faut pas faire de soins externes, on a un forfait, faut pas dépasser le forfait. C'est vraiment le, le, le, le message que j'ai reçu là-haut. Alors ils m'ont, ils m'ont lâché, donc. Puis là-bas heureusement que j'avais un bon médecin traitant puis le pneumologue, ils m'ont soigné comme il faut. Puis au bout de, quelques semaines j'allais mieux ! Mais les, les inflammations de, comment ça s'appelle, des jambes, maintenant, bon ça c'était au mois de X *(mois)* 2014, non X *(mois)* 2013, ouais de X *(mois*) 2013. Je les ai toujours ! C'est terrible ! J'ai, j'ai beaucoup xxx, bon je suis sous morphine, et tout, pour me laver l'entrejambe, et tout. J'ai le vélo et tout, bon je fais du vélo et tout, mais quand, après ben tu crèves de mal, tu crèves de mal ! Alors j'ai, à force j'ai réclamé et tout, j'ai réussi à avoir seulement au X *(mois)* cette année, le compte rendu de, de ce qui a été fait à X *(hôpital)*. Et là, le *Docteur E.*, il marque bien noir sur blanc qu'y a eu erreur médicamenteuse, ils ont mélangé cinq antibiotiques différents en, en cinq semaines de traitement. Et que le mélange des cinq a fait une brûlure des terminaisons nerveuses, partout où y a, on a des liens importants. C'est-à-dire au coude, à l'épaule, aux mains, aux adducteurs, au genou. J'ai toutes les terminaisons nerveuses qui sont attaquées. Et celles de, des adducteurs qui sont détruites. Donc celles-ci, j'aurai mal à vie. Ça la douleur, elle restera à vie ! Parce qu'ils ont mélangé des antibiotiques alors qu'ils savaient pertinemment que le mélange créait une destruction des terminaisons nerveuses. Et ça c'est

marqué.

**C : [Des erreurs médicales.**

**A :** C'est, c'est, c'est] marqué noir sur blanc, qu'y a eu erreur médicale, et que cette erreur va me, va, va m'empoisonner l'existence, toute ma vie.

**C : Oui donc vous avez, en plus de la BPCO, ces différentes douleurs et problèmes de santé qui se sont ajoutés.**

**A :** La BPCO c'est angoissant, c'est pénible parce que je suis coincé là. Mais à part ça, j'ai mal partout. Et je peux plus, je dois diminuer les. Alors cette semaine, au lieu de prendre quatre fois 30 de, de X *(morphine)*, j'ai essayé de prendre quatre fois 20, et puis après de prendre que trois fois 20. J'ai essayé ça jusqu'à mercredi. Puis mercredi j'ai re craqué, j'ai repris tout de suite une dose d'abord de, il est ou, de morphine liquide, de X *(médicament)*, pour que, ça, ça agit en un quart d'heure, pour, pour, pour que ça disparaisse un peu. Puis après j'ai recommencé le X *(morphine)* 30. J'avais trop mal, j'avais trop mal. Faire un pas, j'étais en larmes, en larmes, tellement mal aux jambes, mal au, ici au fond du dos là, puis ça irradie toute la, la douleur qui vous prend, jusqu'au milieu du dos, puis jusque sous les, sous les genoux, la, la douleur qui irradie, puis, tellement mal que vous sentez plus où vous mettez les pieds ! J'aurais pas pu dire si je marchais sur un parquet ou si je marchais sur les graviers, sur, sur le tapis. Y a plus de sensibilité, tu te tiens pour pas tomber, et tu, parce que tu sais pas où tu marches. Tu sais pas si tu marches sur de la glace, sur du béton, ou, t'as, tu sens plus les jambes, tu sens plus la douleur.

**C : Donc des douleurs très, très importantes.**

**A :** Ouais, ouais. *(soupir)* Je dirais, la douleur dans le dos avec la morphine, je dirais six, sept, quoi. Donc, pour moi à huit, neuf, c'est des brûlures quoi, c'est des grosses brûlures, quand vous avez. Parce que bon, xxx, dans l'étanchéité vous avez des, vous utilisez les propanes, et les gros chalumeaux. Donc j'ai vu des ouvriers brûlés, j'ai vu, ce que c'est que des, des grosses douleurs. Et ça pour moi c'est des grosses douleurs comme ça, c'est huit, neuf. Moi je peux dire avec mon dos, j'en suis à six, sept, quoi. Mais, ouais puis pour, pour dire la chance qu'on a avec ma compagnie, c'est une femme qui est admirable. Mais, elle a, ses deux petits chenapans, un il a neuf ans, l'autre il a, il a sept ans. Ils sont tous les deux footballeurs, à X *(lieu)*. Donc le premier il fait deux entraînements plus un match toutes les semaines. Et elle ben, sitôt qu'elle est avec eux, que ce soit dehors ou à la maison, ils jouent au foot puis ils jouent au hockey ! Alors au mois de janvier, février elle était dans la cour là, puis elle a joué au foot avec eux ! Et puis à un moment, elle a perdu tout à coup, il lui est arrivé deux choses. Un jour elle a perdu l'oreille interne, crac elle s'est écroulée, crac. Elle est arrivée ici, ils m'appellent X *(surnom)* les petits : « X *(surnom)*, X *(surnom)* viens ! Mam elle est malade ! » Elle était, puis elle marchait à genou, les bras comme ça, puis à genou ! « Qu'est-ce que ? » ; « je peux plus me tenir debout, j'ai plus d'équilibre ». Ça lui a fait dix jours avant que, elle a eu un déséquilibre de l'oreille interne. Ils pensent c'est parce qu'elle avait, elle a pris une douche et puis qu'elle avait de l'eau dans l'oreille, et puis que elle est sortie avec le froid, ça a dû geler, enfin des explications, je vois pas comment ça peut geler à l'intérieur, parce que pour moi y a quand même tout le crâne qui protège, et puis ça peut pas geler. Enfin, l'explication qu'il avait donnée, le docteur X, j'étais pas, enfin toujours est-il qu'au bout de dix jours, elle a retrouvé l'oreille interne. Mais, au bout de quelques temps, elle se plaignait, elle dit : « nom de dieu j'ai mal au dos, j'ai mal aux jambes ». Je dis : « ouais, c'est un lumbago alors ! ». Elle commence à prendre X *(paracétamol)*, après je lui ai donné, j'ai d'autres, toute une série de, des X *(médicament AINS)*, xxx, puis après je lui ai donné un autre truc un peu plus fort. Puis là je dis : « non écoute si ça, ça te fais pas d'effets, tu vas voir *Docteur F* »., Donc son médecin traitant : « puis il t'envoie prendre, faire une radio ! » Elle va faire une radio à X *(hôpital)* : « ah, ça va pas, j'ai pas pu faire la radio, je tremblais tellement devant le, devant la plaque verticale qu'ils ont pas pu faire la radio, elle est floue, ils me mettent sous morphine ! » Alors ils l'ont mis sur morphine mais les mêmes doses que moi ! Vraiment, alors d'accord, trois, quatre jours après sous morphine, elle monte à, à X *(hôpital)* elle fait les radios, elle revient ici : « ah xxx j'ai mal. J'ai trois vertèbres cassées ! » Mais cassées nettes ! Ecartées, avec écrasement des disques ! Alors ben hôpital et tout. Alors moi je me retrouve ici tout seul. Elle a fait six semaines d'hôpital. Après elle est partie, elle a fait six, six, six ou huit semaines à, à X *(hôpital)*. Alors là c'était magnifique, parce qu'évidemment, donc elle est tombée malade, c'était le X *(date)* juin. Puis moi le X *(date)* juin, j'ai fait mes baisses de pression et compagnie ! Je me suis assommé. Alors le X *(date)* juin, j'étais à l'hôpital. J'ai passé le X *(date)*, pour mon anniversaire, pour mes 64 ans, j'ai été sous le scan, enfin dans la, la, j'appelle la soufflerie, enfin tous les trucs vraiment, puis bien agréable toute la journée. Donc j'étais, ils sont venus avec un gâteau le soir, j'ai même pas pu manger une tranche tellement j'étais mal. Alors les petits gamins quand ils voyaient que je pouvais même pas manger

ça ils étaient en larmes, les petits chenapans, parce qu'ils m'aiment bien, puis bon, quand ils voient quelqu'un qui a très mal, ben ils souffrent aussi, quoi. Et, deux, trois semaines après, ben elle était au deuxième étage à X *(hôpital)*, puis moi j'étais au troisième, quoi. On a fait six semaines ensemble à X *(hôpital)*. Puis après elle quand elle est ressortie elle est venue ici, puis moi j'ai fait encore cinq ou six semaines à X *(hôpital)*. En tout j'ai fait 17 semaines de suite.

**C : Oui. Oui assez long comme hospitalisation. Et votre compagne aussi qui a eu [des soucis de santé.**

**A :** Et puis] au bout de 17 semaines, eh ben j'avais perdu six kilos ! Parce que la nourriture… Une semaine ou deux ça va. Mais au bout de deux semaines, quand vous avez toujours les, les mêmes épinards, le même, la même saucisse, la même purée, avec toujours le même gout, puis toujours le même dessert et compagnie, à la fin vous mangez le tiers, le quart, juste un petit peu, puis vous vous bourrez de chocolat, de fruits et tout, pour remplir le ventre pour que vous ayez, que vous ayez pas trop l'impression de faim quoi, simplement. Puis alors je sors de, j'ai trouvé admirable, parce que quand je suis sorti de X *(hôpital)*, le X *(date)*, j'avais le, le rendez-vous le lendemain, avec le *Professeur G.*, responsable du centre de, enfin vous devez connaître, quoi. Et puis j'étais là avec le *Docteur H.*, xxx, et madame, madame la Professeure. Elle me voit arriver, j'étais en chaise roulante. C'est mon, c'est mon, mon beau-fils qui m'a amené, j'avais tellement mal aux adducteurs, comme j'étais resté des semaines au lit, j'avais tellement aux adducteurs, j'étais incapable de marcher ! Parce que c'était complètement enflammé, et j'étais dans le même état que quand je sortais de X *(hôpital)*. Elle, elle m'a vue : « oh mais on vous opérera jamais si vous êtes faible comme ça ! Si vous êtes faible comme ça, il vous reste une chose, c'est la caisse à sapin ! » qu'elle me dit, directement. Elle dit : « on va pas mettre un demi million à vous opérer si vous êtes mal comme ça, puis qu'après vous allez pas tenir le coup ! ». Puis elle me dit : « vous avez tellement maigri, que vous avez aucune réserve ». J'ai dit : « mais vous voyez ça où ? » ; « ah vous avez le creux du doigt ici qui est tout plat. Une personne en bonne santé, c'est tout rond ». Je dis : « mais madame, vous regardez dans la glace, ouais d'accord ! Mais vous vous êtes d'un caractère, vous êtes pas grosse ! Vous êtes d'un caractère rondelet, vous avez des rondeurs partout, sans être grosse, mais vous avez des rondeurs partout ! ». « Y a des personnes qui ont le caractère sec, qui n'auront jamais de rondeurs, même qu'ils sont en pleine santé ! » ; « ah c'est pas possible, il faut que vous repreniez des kilos avant qu'on vous opère ! ». J'ai dit : « mais madame, y a aucun souci ! Vous me revoyez dans trois semaines. J'ai pris trois kilos ! » ; « c'est pas possible ! ». J'ai dit : « si madame. C'est moi qui cuisine, ou c'est ma compagne ». Maintenant j'ai pris, le X *(date)*, j'ai pris sept kilos !

**C : Vous avez réussi à reprendre le poids.**

**A :** J'ai, j'ai, j'ai deux kilos de plus que mon poids de forme hab', normalement mon poids de forme c'est 71. Là je suis à 73, 73.5. Et, je, je prends, je prends à peu près 150 à 200 grammes par jour, avec le, le, le, le [régime alimentaire que j'ai.

**C : Vous avez repris.]**

**A :** Donc, je, ben, bon moi c'est, hier j'ai fait, hier j'étais un peu en forme, c'est moi qui fait le ragoût, autrement c'est elle qui cuisine. Mais à midi, elle m'a fait un truc que je connaissais pas, c'est la première fois qu'elle cuisine, c'était, c'était, c'était réussi ! Elle avait pensé qu'y en aurait pour ce soir, puis en définitive on a fini les casseroles, quoi. Ce soir elle fera autre chose.

**C : C'est votre compagne qui fait à manger, [souvent.**

**A :** Ouais.] Puis on s'entend très bien, que ce soit à ce niveau-là. Puis, enfin l'humour, aussi, donc on a, ouais, de la chance, quoi.

**C : Ouais vous disiez que, vous avez ce soutien de [la part de votre compagne et de votre fille qui vous faisait avancer.**

**A :** Ah ouais, ouais, ouais, ouais, ouais, ouais.] Ouais, c'est clair. Ouais puis ma fille, c'est aussi, c'est aussi une aventure extraordinaire, quoi, j'en reviens pas. Parce que bon, moi mon père était architecte, et lui il a arrêté de bosser à 45 ans parce qu'il a, il a tout lâché pour, pour aller vivre, parce qu'on avait une maison à X *(lieu)*, près de X *(lieu)*, et un chalet à X *(lieu)*, à 15 minutes de X *(lieu)*, aussi en montagne. Alors il a décidé de vivre avec sa maîtresse. Alors il a vendu la maison de X *(lieu)*, qui était un chef-

d'oeuvre. 12 pièces, piscine bassin olympique, enfin de, il avait les, les sous à l'époque. Il a revendu ça, il a payé deux appartements à ma mère en X *(lieu)*, il lui a donné une pension alimentaire, il est parti vivre au chalet à 45 ans. Puis là il a vécu, vécu de ses rentes. Bon, il est décédé justement le X *(date)*, juste, ben deux semaines avant que, deux, trois semaines avant que moi je rentre à l'hôpital, puis que, commence mes péripéties, quoi. Bêtement, un accident bête mais. Enfin toujours est-il que ma, ma fille quand elle a fini son, sa matu, bon elle était, une facilité d'étude. Elle parlait déjà couramment français, allemand, anglais, italien, quand elle a fini le, enfin l'italien un peu moins, mais français, allemand, anglais parfaitement *(toussotement)*. Puis elle a commencé les, les études de notariat. Elle est arrivée à Noël, elle m'a montré son carnet, enfin son, ses résultats. Evidemment, elle était dans les premières, comme toujours, quoi. Puis là : « papa, j'arrête. Je prends six mois sabbatique, puis après je commence l'architecture » ; « comment ça ? T'es folle ! ». Puis là elle me sort les photos. Parce que bon, elle avait reçu un appareil numérique de la part de son oncle qui est canadien. Puis moi je lui avais offert mon vieil appareil, X *(marque)* argentique, avec un téléobjectif et compagnie. Un, bon c'était un bijou, l'appareil c'était une fortune ce machin. Mais moi je, moi j'étais passé au numérique, plus simple, plus léger, alors je lui ai donné ça. Puis elle m'a rien dit, puis après elle m'a, elle m'a montré des photos d'architecture qu'elle a fait. Puis c'est là qu'elle me racontait, parce que je la voyais jamais le week-end. Puis elle m'a dit : « mais moi je suis une fana de easy-jet. Tous les week-ends je suis, une fois à Barcelone, une fois à Stockholm, une fois à Bucarest, une fois à xxx. Toutes les semaines je vais dans une autre ville ! Tous les week-ends je suis trois jours dans une autre ville ! » ; « mais comment ? » ; « mais j'ai des copines dans toute l'Europe ! X *(ville)*, c'est une ville internationale, j'ai étudié avec des copines de toute l'Europe, elles sont reparties chez eux, elles ont fini le truc, elles ont des adresses pour moi au Portugal, en Espagne, en, j'ai des adresses à travers toute l'Europe. Je suis tous, tous les week-ends ». J'ai dit : « c'est pour ça que ». ; « non les week-ends je suis toujours ! Easy-jet, X *(somme d'argent)* ». Elle dit : « mais je mets X *(somme d'argent)* par mois d'easy-jet et je suis ». ; « et tu fais quoi ? » ; « je regarde les maisons, je regarde l'architecture, je fais des photos ». Elle m'a montré, elle avait un book, mais rempli de photos d'architectures ! Des immeubles rébarbatifs, des usines, des immeubles magnifiques et tout, mais rempli !

**C : Oui, donc vous avez.**

**A :** J'ai dit : « bon ok, t'as chopé le virus comme moi, quoi ». Moi j'ai fait pareil quand j'avais, quand j'avais 18 ans. Si je suis devenu architecte c'est parce que, parce que j'adorais ça, quoi. J'avais aussi le virus, quoi. J'ai dit : « oui, t'es comme moi, t'as chopé le virus. Bon ok, alors ». J'ai dit : « bon ok d'accord ». Alors je lui ai payé six mois sabbatique, je lui ai donné le. Moi je vis simplement donc je suis, puis à l'époque elle touchait, pension de, j'ai de la chance, un deuxième pilier pour moi, il est, bon il est pas trop important, j'ai 2'000 francs par mois, mais j'ai une assurance complémentaire pour les études, pour les frais d'étude. Et ça me donne 1'200 francs pour les frais d'études de ma fille. Alors ça, je lui ai toujours donné ça ! Puis je lui donnais la pension normale, 1'000 francs de pension que j'avais décidé depuis des années, depuis qu'on s'est séparé quand elle avait 15 ans, j'ai décidé ça avec mon ex-compagne, de lui donner 1'000 francs par mois de pension. Même que mon salaire il a été divisé par trois par rapport à ce que je gagnais les derniers temps. C'est normal pour moi de, je veux pas diviser par trois la pension que je donne, quoi. Donc j'ai continué à payer ça. Alors elle a profité de, de faire. Alors là elle est partie, puis elle s'est créée, elle a construit un appareil de photos à l'ancienne, comme avait les, les gars du temps des cow-boys, et tout. xxx sur trépied, la caisse en bois, la plaque de verre avec le sel d'argent dessus, le jus de citron, l'urine, pour. Et, elle est partie avec sa caisse en bois et ses trucs. Elle a été faire des photos des maisons en chaume, en X *(pays)*, en X *(pays)*, en X *(pays)*. Mais elle a fait que des maisons en toit de chaume. Mais c'est des vieilles photos floues, avec juste, y a juste quelques parties nettes, quand par hasard ça bouge pas, qui est assez lumineux. Puis elle a fait un petit book comme ça, juste pour elle, pour son plaisir quoi, puis, puis elle a gardé ça comme ça. Puis là elle commence, alors première année d'architecture, ils sont 700 à X *(ville)*. Première année : « ah puis t'es combien avec ça ? » ; « 5.72. Ouais, avec ça je suis troisième sur 720 ». Deuxième année comme ça. Bon : « ouais, ah non cette année c'est moins bien, je suis quatrième ». Mais y a plus que 500 étudiants là, y en a déjà 250 qui ont raté, quoi. Puis là elle dit : « oh bon, je dois continuer l'autre année, mais j'ai pas envie de, de continuer à X *(ville)*, je vais me renseigner, et tout ». Puis là sans qu'elle demande rien, y a l'Université de X *(pays)* qui lui écrit : « on suit les étudiants un peu partout, à travers l'Europe. On a vu vos résultats à X *(ville)*. On vous invite à venir suivre les cours de l'Université de X *(ville)*, tous frais payés pendant une année ».

**C : Donc elle a fait ça ?**

**A :** Alors elle est partie au X *(mois)*, elle s'est fait six semaines de cours de X *(langue du pays en question)*, parce que X *(langue)* et X *(langue)* et X *(langue)* c'est pas la même chose. Y a autant de

différences entre X *(langue)* et X *(langue)* qu'entre X *(langue)* et X *(langue)*. Donc c'est, c'est, c'est, alors elle a fait des cours de X *(langue)*. Elle a fait une année à X *(ville)*, bon, easy-jet et compagnie, donc elle a continué à travers l'Europe, puis, puis ici en X *(pays)* quoi, pour, pour venir me voir moi puis sa mère. Elle finit évidemment avec les grands honneurs, là-bas à X *(ville)*, première de la promotion pour une étrangère, ça faisait des années que c'était pas arrivé, quoi. Et puis là-bas elle a connu alors, là-bas elle a connu des, elle a connu des X *(origine)*, des X *(origine)*, elle a connu beaucoup de personnes des pays de l'est. Avant elle connaissait des personnes de l'occident. Et là elle a connu beaucoup de personnes des pays de l'est. Alors elle ressort de là. Elle dit : « oh non maintenant que j'ai appris ça. J'arrête à X *(ville)* ». ; « comment ça ? » ; « non, non je veux perfectionner mon X *(langue)*, je vais étudier à X *(université)* à X *(ville)* ».

**C : Vous avez votre fille qui suit vos pas [xxx.**

**A :** Alors] en trois semaines, en trois semaines, j'ai un ami d'enfance, un de mes meilleurs amis, enfin un des seuls qui est resté, qui est un très bon ami, qui a, qui a, qui a une petite entreprise de conciergerie à X *(ville)*, il lui a trouvé un petit  appartement pour quelques temps. Puis trois semaines ou un mois après, elle a trouvé une colocation avec d'autres étudiants d'architecture. Donc elle a fait quelques mois de colocation avec eux. Elle a fini l'année d'avant et tout. Puis elle est venue bosser dans un bureau ici pendant, l'année passé au X *(mois)*. Et dans le berceau d'architecte qui est ici à côté à X *(lieu)*. Puis là elle a, elle a dû participer à un concours d'architecture, pour la X *(projet)* à X *(lieu)*. Alors ils lui ont dit : « écoute, puisque toi t'es nouvelle, tu sors de, tu fais toi tout le concours ». Alors elle a fait tout le concours. Ils ont déposé le concours au nom du, au nom du bureau d'architecte, donc le X *(bureau)*. Mais elle, elle a pas pu signer le, le, le book. Puis un book, c'est quand même 1'000 francs. Donc toutes les, tous les plans, les photos, des maquettes, et tout. C'est, c'est, c'est un truc cartonné, avec des photos magnifiques, ça vaut une valeur, c'est, c'est, ouais c'est assez monumental, comme, comme, comme, réalisation. Mais pas que, tout le projet qu'elle a fait, alors qu'elle est étudiante en fin de troisième année, c'est ça qui a été décidé, c'est ça qui va être construit chez nous. Y a pour 500 millions de francs de travaux. C'est elle qui a tout déssiné. C'est son premier projet, premier truc qu'elle fait, ils ont chopé le mandat.

**C : Elle a suivi vos pas et plutôt bien réussi.**

**A :** Beaucoup, beaucoup mieux réussi.

**C : En architecture.**

**A :** Beaucoup mieux réussi que moi. Alors pour, pour la remercier, le bureau d'architecte, a refait faire tout le book complet, mais en mettant chaque fois, au bas de chaque page : « créé par, dessiné par, imaginé par ». Y a chaque fois son nom sur toutes les feuilles. Et ils ont refait le book complet à son nom.

**C : [Une belle reconnaissance.**

**A :** Alors après elle revient ici] : « papa, j'ai un souci. Tu connais X *(nom de bureau)*, et tout ? » J'ai dit : « ouais, c'est les X *(nationalité)*, ça ». Elle m'a dit : « ouais c'est le meilleur bureau d'architecture en X *(pays)* ». J'ai dit : « qu'est-ce qu'y a ? » ; « ah ils veulent que j'arrête les études ». ; « comment ça ? » ; « ah ils me donnent 10'000 livres, donc 26'000 francs par mois, pour venir bosser chez eux ». Sans qu'elle ait le diplôme. 26'000 francs, salaire de départ, quand elle a travaillé chez eux. C'est un des meilleurs bureaux d'architecture, aller, peut-être quatrième ou cinquième bureau au monde.

**C : Ouais. Donc elle a des bonnes, des bonnes opportunités, des belles propositions de, [de travail.**

**A :** Ouais. Alors elle a dit] non, elle a refusé, et tout. Puis maintenant donc elle est en dernière à X *(ville)*. Elle a un prof qui s'appelle X *(nom)*. C'est un monsieur qui a fait les nouveaux X, au X *(pays)*, je sais pas si vous avez vu, une maison tout en pierre taillée, et compagnie. Alors, sur 400 élèves, du cursus final, à X *(université)*, il a une classe de sept élèves. Elle fait partie des sept élèves. Et ils vont tous les deux jours, ils prennent le train de X *(ville)*, pour aller au X *(lieu)*, qui est à une heure et quart de train, c'est assez proche, c'est. Et ils vont sur place, dans le village, ils doivent refaire un village complet tout en pierre de taille, la même chose, mais un village où y aura le bistrot, l'épicerie, le grand magasin, l'école, l'église, la

salle de sport, et les maisons d'habitation. Et tout en pierre naturelle, comme ça. Et ils doivent faire les plans d'urbanisme, les plans des maisons, les, tout, tout, tout ! Ils ont six mois pour faire ça, à sept. Alors ils vont tous les deux jours, en train, là-bas, et puis. Bon à part ça ils ont encore le boulot normal d'étude à faire. Et là, on aura fini, les maisons seront finies, puis ouais, on aura tous notre diplôme, quoi.

**C : Des belles, des belles perspectives.**

**A :** Et : « et puis après tu fais quoi ? » ; « oh je sais pas. On me propose d'aller bosser en X *(pays)*, en X *(pays)*, en X *(pays)*. Je crois que je ferai jamais d'offre d'emploi ». Moi je suis sidéré, quoi. Bon, sa mère est artiste aussi, elle fait de la peinture, de la sculpture, de la poterie.

**C : Elle a de bonnes possibilités pour, pour l'avenir.**

**A :** Oui ! Elle m'a vu dessiner, puis photographier pendant des années ! Ouais, elle a chopé un virus comme ça, puis, puis, mais alors elle est, ouais elle a, je crois 170 de, de, de, de QI, quoi, ce qui est. Moi j'étais content avec mes 130. Elle, je sais pas, je sais pas comment elle a fait quoi !

**C : Et, et si on revient plus vers, vers la BPCO.**

**A :** Ouais.

**C : Si, si on regarde par rapport à ce soutien, vous disiez de votre fille et de votre compagne, est-ce que vous avez, quel autre type de soutien avez-vous de la part de vos proches, par rapport à la maladie ?**

**A :** Ben j'ai des soutiens au niveau de, on peut dire ce qu'il est des bricolages, des travaux lourds. Bon maintenant on a, on a un petit jardin familial, donc c'est eux qui s'en occupent, c'est moi qui l'avais pris mais, c'est eux qui s'en occupent complètement, parce que moi depuis, j'y ai pas mis les pieds depuis deux ans. Donc ils m'aident pour tout ce qui est de, le côté pratique, et tout. Et puis bon, c'est surtout l'aide morale quoi, parce que, je dis c'est mon beau-fils, c'est pas mon beau-fils, c'est le fils de ma compagne mais, pour moi c'est mon beau-fils. Sa femme, c'est sa belle-fille, je suis avec elle partout, c'est mon beau-fils. « ah mais c'est pas ». J'ai dit : « si ». Et puis, sa fille, a eu un ami, bon ils sont plus ensemble maintenant, mais ils sont toujours, ils sont toujours amis, mais ils sont plus amants. Mais, mais, pour moi c'est, c'est comme mon petit frère, il a 45 ans, c'est comme mon petit frère, on est comme cul et chemise. J'ai un souci, bon lui, il est, il est, il travaille dans les ordinateurs et compagnie. J'ai un souci d'électronique, il vient ici il me l'installe. Il corrige mes programmes, il regarde les bugs et tout. Puis après on passe des, on fait des rires, il explique mes boulots, il m'explique les siens. Il a été militaire de carrière dans le, il a été xxx, dans les, les armées italiennes. Donc il a fait la, la guerre en X *(pays)*, en, comment ça s'appelle, au X *(pays)*, enfin il a, il a dû intervenir dans, dans le cadre des missions de l'ONU, dans les forces italiennes, les missions de l'ONU. Il a, il a fait 12 ans d'interventions sérieuses, quoi, il a vraiment dû se battre et puis défendre sa peau aussi, donc. Ouais, donc on a énormément de choses à partager, quoi ! Et puis, alors, sa fille aussi qui est, qui a aussi une vie passionnante, tout ce qu'elle a vécu ! Alors j'ai énormément de soutien moral, j'ai énormément de soutien moral ! Et je suis… j'ai énormément de distractions, de par, de par toutes les amitiés que. J'ai perdu mes amis, à part un de X *(ville)*. De X *(ville)* et du X *(canton)*, parce que ben, ouais, de temps en temps un coup de fil, mais y a plus de présence, je peux plus aller, je peux plus participer aux fêtes ou aux, ou aux réunions, ou aux sorties qu'ils font, donc tu t'éloignes, puis tu les perds petit à petit, quoi. Ouais, de temps en temps j'ai une carte de visite, ou un, un petit mot sur internet, un truc comme ça. Mais c'est plus la même, j'ai plus le même rapport amical que la, tout ce que j'ai avec ma belle-famille, quoi. Par contre du côté de ma famille, elle n'existe plus. Ils m'ont, ils m'ont exclu lors du décès de mon père, ils m'ont exclu du deuil, pour, pour, pour, pour des, des questions mais, des questions d'argent, sur lesquelles moi je suis pas coupable du tout. Mais, ils veulent que ce soit moi le coupable, alors ils m'ont, ils m'ont, ils m'ont tout mis sur le dos. Donc y a toute ma famille en X *(canton)* qui croit que c'est moi le mouton noir, alors qu'en définitive c'est ma grande soeur qui est une conne parfaite. Elle a juste divorcé cinq fois pour vous dire. Et puis qui, qui, qui est une langue de vipère, quoi. Et puis bon moi j'en ai, j'ai ma mère qui est en train de mourir de BPCO, dans un EMS à X *(ville)*, elle a 97 ans, maintenant, donc elle s'accroche à la vie pour voir ben ma fille, avoir son diplôme, parce que c'est la, de tous les petits-enfants, c'est la seule qui a été à l'Université, donc elle s'accroche, elle veut voir ma fille avec son diplôme d'architecte. Puis après elle se laissera partir, mais elle s'accroche à ça. Elle parle que de ça, c'est le seul, quand elle est lucide, c'est, c'est de savoir comment va X *(prénom de sa*

*fille)*, si elle réussit bien. Puis après elle replonge dans ses, dans ses délires parce qu'elle est, ouais elle shootée aussi à pas mal de trucs pour supporter, parce que bon, c'est des produits, des produits d'accompagnement, enfin de, comment ça s'appelle de mort tranquille, quoi. Mais bon, je préférerais qu'elle, qu'elle téléphone quand même à Exit, parce que là, c'est vraiment pas, catastrophique ce qu'elle vit maintenant, quoi. Mais bon, elle est trop catholique pour ça. Par contre, donc, donc ce côté-là, pour moi le X *(canton)* n'existe plus. Par contre, mais alors j'ai retrouvé.

**C : Votre belle-famille.**

**A :** J'ai trouvé des, des trucs magnifiques, quoi. Et puis dans le voisinage, par exemple ici, d'être ici au X *(lieu)*, le X *(lieu)*, c'est un petit village, les gens sont, les voisins sont super ! Ils sont, ils sont d'une attention, d'une gentillesse : « ah, si on peut vous rendre service. Ah mais avec votre truc, laissez-moi vous porter votre ». Ouais, le coeur sur la main, les gens ici. Quand on disait la réputation du X *(lieu)*, c'est ci, cela, mais alors ceux qui disent ça connaissent pas le X *(lieu)* ! Parce que les gens du X *(lieu)* entre eux, ils sont superbes, c'est vraiment superbe, quoi !

**C : Ouais, donc vous avez ce soutien de [la belle-famille, de.**

**A :** Ouais, ouais, ouais.] Bon, par contre la régie ici, complètement cons ! Alors on doit partir, ils nous ont expulsés, ils nous expulsent pour. Ils veulent simplement rendre l'appartement encore plus confortable et puis le louer encore plus cher ! Alors déjà qu'ils ont passé de 1'200 à 2'600 francs quand nous on a loué. Donc j'ai vu le tarif précédent c'était 1'200, nous on paie 2'600, plus charges, plus garage. Et, donc on est à 3'080. Ça reste un appartement HLM qui est pas isolé ! Quand la voisine est dessus, bon elle travaille la journée, donc elle, elle vit la nuit ici. C'est *(il imite le bruit)*, vous vous entendez, on a l'impression qu'y a un éléphant qui, qui court en-dessus, donc y a aucune isolation phonique contre les coups. On n'entend pas les bruits aériens. Mais tout ce qui est des bruits de chapes, y a pas ce qu'on appelle des, des, chapes flottantes, c'est-à-dire le sol transmet les, le bruit en-dessous. Chose que maintenant, ne se fait plus du tout, quoi. Et, les dalles, c'est du béton brut, y a même pas de plâtre au plafond. Y a des, y a des, des coulures, des infiltrations, ils réparent pas ! Ils laissent aller à vau-l'eau et tout, puis ils veulent essayer de louer ça encore plus cher ! Mais ils arriveront jamais ! Maintenant ce qu'il y a de bien, c'est qu'on connaît bien le concierge, et puis les autres, je connais les régisseurs des autres années, et on veut rester dans, on va déménager, mais ici, dans la grande tour. On veut finir notre vie ici ! Parce que c'est, c'est, c'est, venez-voir.

**C : Vous allez rester ici.**

**A :** Venez *(il se lève)*.

**C : Je peux vous faire. Ça va pour l'oxygène ?**

**A :** Mais je vais le monter *(en parlant de l'oxygène)*. On va aller cinq minutes sur le balcon.

**C : Faut que je prenne mes enregistreurs avec alors.**

**A :** Ouais.

*(A et C vont sur le balcon)*.

**C : Je veux pas vous retenir trop longtemps non plus, [hein. Vous me dites si vous fatiguez.**

**A :** Non mais, ça me gêne pas du, ça me gêne pas du tout.] Regardez, [regardez ce qu'on a.

**C : Ah oui c'est magnifique !]**

**A :** Là, la plupart du temps vous avez les canards, les, les renards qui se baladent le long de la berge. De temps en temps vous voyez une couleuvre qui traverse. On a les jumelles qui sont là. Moi je passe, quand il fait beau, qu'il fait pas trop froid, je passe deux, trois heures par jour ici assis sur la chaise, à regarder dehors et tout. Bon, faut juste qu'on se protège des pigeons, pour pas être *(rires)*. *(toussotements)*. Non c'est magnifique, c'est, c'est, c'est. Une vue pareille, et pis y a, bon on entend les, quand y a la, la bise qui rabat le bruit, on entend les avions, au, au décollage, ici. Autrement on les entend dans la chambre à

coucher mais, c'est un bruit ça passe, deux minutes après y a un autre bruit qui revient, ça repasse, et puis on s'habitue. Mais c'est tellement bon [d'être là.

**C : C'est très beau.]**

**A :** Dans la, dans la chambre de l'autre côté y a la lumière, y a le soleil qui arrive jusque, jusque la chambre de l'autre côté. C'est juste génial, donc, si on doit déménager ce sera dans cet immeuble. On va pas, on va pas aller ailleurs, quoi !

**C : Pas repartir.**

**A :** Et je pense plutôt acheter un appartement. Parce que ma mère ben malheureusement va, va disparaître prochainement, elle a quand même encore ses deux appartements qui valent dans les 7 à 800'000 francs, je sais pas exactement, j'ai pas, je me suis pas amusé à faire les calculs, j'ai pas envie de. On va partager ça entre les trois soeurs, ça fait 250, 300'000, ça suffit comme apport principal pour, pour acheter un appart ici, même si c'est 5, 600'000 si vous mettez le tiers au départ, après on met 2'000 francs par mois de loyer au lieu de 3'000, et puis en dix ans, l'appartement est à vous, quoi.

*(A et C retournent dans l'appartement)*.

**A :** Donc, on part plutôt dans cette optique, avec ma compagne, pour pouvoir rester, ou louer si on peut, si on n'a pas la, la chance d'acheter, ben on continue en location. Mais on veut vraiment rester ici, parce que c'est, malgré les inconvénients du bruit, y a tellement d'avantages. Déjà au niveau de la situation, vous sortez bon, maintenant je peux plus faire, mais avant ! On met huit, dix minutes à pied, à travers le parc pour être à la X *(magasin d'alimentation)* ou à la X *(magasin d'alimentation)*. Y a X *(magasins d'alimentation)* puis un petit restaurant entre-deux, une petite cafétéria. Y a le toubib, y a un centre médical complet. Y a, y a deux, trois bistrots sympathiques. Y a tout ce qu'on, c'est un petit village, mais y a tout, à cinq, sept minutes y a tout, puis on passe [à travers un.

**C : Vous aimeriez rester] ici.**

**A :** Ah oui, oui ! Oui, oui. J'ai envie de finir ma vie dans le quartier ! Ça c'est, et ils sont en train de construire un, la X *(commune)* a décidé dans le, dans la boucle de X *(fleuve)*, du X *(fleuve)* qu'est-ce que je dis. Juste, là vous avez la station d'épuration. Juste à côté, y a un terrain qui est libre, ils vont construire un grand EMS avec 600 chambres. Ce serait le plus grand EMS du canton. C'est la X *(commune)* qui va faire ça.

**C : Je vois que, je vois que l'heure avance, [je veux pas vous retenir encore trop, trop, trop longtemps.**

**A :** Ouais, ouais.]

**C : Vous me dites [si vous commencez à, à fatiguer. Donc voilà c'est.**

**A :** Non, non, pour moi, moi j'ai rien d'autre.] Donc si, si je vous, je vous intéresse, vous restez ! C'est vous qui décidez quand vous voulez partir !

**C :** ***(rires)*. Donc peut-être juste, par rapport aux, aux soins de, de la BPCO. Vous m'avez dit, vous avez reçu différents traitements. Je sais qu'on peut aussi discuter des soins qu'on désirerait en fin de vie. On peut aussi écrire, ce qu'on appelle des directives anticipées. [Vous avez peut-être entendu parler de ça ?**

**A :** Oui, alors ça justement.] C'est la seule chose que j'aimerais faire. J'aimerais, je sais pas à qui je dois m'adresser pour avoir ces formulaires de directives anticipées. J'en ai parlé déjà plusieurs fois avec ma fille, puis ma compagne. J'ai encore, j'ai encore vu dernièrement à la TV. Je l'ai cherché sur internet, je les ai pas trouvés, les formulaires. Ça, je serais intéressé de remplir ces formulaires, oui. Parce que je sais que je veux pas, si y a des risques que je sois légume, je préfère passer l'arme à gauche. D'ailleurs je veux, je veux aussi m'inscrire à Exit. Parce que je veux vraiment pouvoir choisir, xxx ma famille quoi, je veux pas finir comme ma mère que je vois, ma mère qui, qui, qui, se détériore petit à petit, puis qui, qui, qui, qui hallucine, qui sait plus où elle en est, parce qu'elle prend tellement de mélanges entre, comment

ça s'appelle, entre les antidépresseurs et la morphine que, elle voit des papillons, des, des, des, des, des, des éléphants roses carrément, quoi ! Elle me dit : « mais je vois des éléphants roses, quoi ! ». Donc, j'ai pas envie d'en arriver là, quoi. Je veux mourir avant, ça c'est clair. Je veux choisir, je veux choisir, ça c'est sûr, point.

**C : C'est quelque chose que vous avez pas encore fait [mais que vous seriez intéressé.**

**A :** Ouais. Que, que j'ai l'intention de faire.] Bon, de toute façon avec les, avec les doses de morphine que j'ai ici, ça c'est de la morphine à 0.2%, je bois la bouteille, dans une heure je suis plus là, hein. Bon, c'est pas une belle mort, la morphine. J'ai déjà vu des overdoses, quand vous étouffez, c'est pas xxx, pour ça que je partirai plus dans l'idée d'Exit, où y a un calmant, puis vous vous voyez pas partir. Parce qu'avec les produits que j'ai là, je pourrais facilement partir, mais, ce serait pas agréable. Et puis pour le moment il en n'est pas question ! Pour le moment je m'accroche, à ce désir de, de, de, de transplantation. Je suis tout heureux d'aller aux X *(hôpital)* demain, et puis j'espère que, les nouvelles qui vont me donner seront positives, quoi. Moi je m'accroche à ça, puis j'ai vraiment envie de. J'ai des travaux à faire au jardin, j'ai envie de, de, retaper le toit. Le toit, je connais parfait, par coeur toutes les techniques. Y a des choses à faire, j'ai envie que ce soit moi qui le fasse ! Et puis j'ai envie de planter mes légumes, regarder comment. xxx On a des tomates qu'on a plantées au mois de janvier. On n'a jamais pu les transplanter parce qu'elles étaient rabou'. C'est les tomates les plus vieilles que j'ai jamais vu au monde ! Elles ont 11 mois. Ce plant de tomate il a 11 mois, et il commence à faire, à faire les petites tomates. Donc je pense que pour Noël on a des petites tomates, c'est des minis ! Elles seront rouges pour Noël. Au bout de 12 mois, donc, c'est. Jamais vu ça ! Mais bon, c'est tous des petits plaisirs comme ça, mais. Ouais, moi j'ai envie de continuer ! Et puis pas seulement d'avoir des petits plaisirs, moi j'ai envie de pouvoir re, recommencer du ski, re, refaire, refaire du, du, du canoë, refaire, reprendre mes activités normales ! J'ai envie de reconstruire xxx, de, de refaire mes muscles, de reconstruire mon corps, quoi ! Bon, je sais qu'y a pas beaucoup, je vois en définitive comment, les deux, trois fois où je dois faire un effort pour, je sais pas pour un bricolage ici. Comme l'autre jour, on a dû mettre de l'ordre dans le réduit. *(soupir)* J'ai bossé xxx, on a mis l'oxygène sur six, en étant tout lentement, mais j'ai travaillé quand même pendant quatre heures ! Et le lendemain j'avais aucune courbature, j'avais rien du tout. Les seules douleurs que j'ai vraiment, et qui sont, qui sont violentes, c'est ces douleurs des adducteurs, les, les, les douleurs dans les jambes, quoi. Parce que maintenant c'est tellement fort, les adducteurs là, que ça vient sur les côtés et puis j'ai aussi les fessiers qui sont, qui sont irrités, par, par irritation chronique, quoi. Ah purée, j'ai une merde dans l'oeil depuis ce matin *(rires)*.

**C : (rires). Donc voilà, vous avez envie de, de poursuivre, et de.**

**A :** Ah oui moi je, oui, oui, j'ai vraiment envie de, de me reconstruire, et puis de, de profiter de la vie, profiter de, de mes enfants, de mes petits-enfants, et. Non, non, ça j'ai puis j'ai des choses à faire, quoi ! Je vais pas, je vais pas m'arrêter maintenant, quoi ! Non pour ça, les questions de moral, des fois j'aurais besoin d'aide, parce que y a des, des, des, tout à coup j'ai, j'ai un, un téléphone d'une de mes frangines pour une connerie, et puis ça me fait, ça me fait replonger pendant deux, trois mois, quoi, par rapport à, aux vacheries qu'ils ont fait. J'ai pas pu assister à l'ensevelissement de mon père. Ils m'ont même pas dit quant c'était, pour vous dire *(toussotements)*.

**C : [Donc en terme de soutien.**

**A :** Donc ça, ça, ça c'est une blessure] qui restera toute ma vie, ça. Et puis ben il suffit qu'un, qu'y ait un truc sur le X *(canton)*, ou un truc sur la, à la TV, un truc sur le X *(canton)*, ben ça me replonge dans toutes ces histoires, et puis. Je sais que si je prenais un antidépresseur, ça c'est des trucs qui passeraient très vite. Mais là comme j'en prends pas ben, ça traîne tout à coup des jours, voire des semaines quoi, donc. Mais c'est des, c'est des conditions pour que je puisse avoir la greffe, pour que je sois disons dans les meilleures disponibilités possibles pour la greffe. Alors je m'accroche à ça, et puis, et puis je veux passer ce cap, quoi ! Je, bon maintenant j'espère faudra pas attendre des mois, voire des années pour.

**C : Pour la greffe.**

**A :** Pour la greffe, quoi. Bon, c'est un peu ce qu'ils m'ont expliqué vu que je suis A positif j'ai plus de chances que les autres, parce que je peux recevoir les deux, les O et les A positif, donc ça fait 75% de chance, au lieu d'avoir que la moitié, quoi. Donc c'est, c'est une plus-value, si on veut. Mais bon !

**C : Ouais. Donc, dons si je résumé un petit peu tout, tout ce que, tout ce que vous m'avez dit. Donc vous avez participé à cette étude, pas pour vous, mais pour qu'on puisse aussi avoir [un point de comparaison.**

**A :** Ah oui, tout à fait.]

**C : Avec, avec l'autre groupe qui avait reçu cette, cette intervention, parce que vous aviez pas pu être dans ce groupe d'intervention, parce que vous aviez une santé plutôt fragile.**

**A :** Ouais, ouais c'était, c'était prendre un trop grand risque.

**C : Ouais, de, de, d'être dans, dans l'autre groupe.**

**A :** Ouais.

**C : Donc vous avez eu passablement de traitements et de mésaventures pour, pour la, pour la BPCO. Et ces douleurs aussi importantes et tout ce traitement à, à la morphine. Et maintenant vous êtes en train de, d'attendre, et vous avez ce rendez-vous demain pour voir, pour , pour la greffe.**

**A :** Ouais.

**C : Donc c'est aussi pour ça que vous avez pas pu prendre d'autres médicaments, comme vous disiez, pour gérer l'angoisse, par exemple.**

**A :** C'est depuis le mois de juillet que, ils ont fait, quand ils font les électrocardiogrammes, ils ont vu que le cul, donc les, les, l'espace entre les pointes était pas, pas correct, et pis que c'était, c'était à cause des mélanges médicamenteux. Puis c'est là qu'ils ont discuté avec moi, puis qu'ils ont dit : « est-ce qu'on essaie de, d'améliorer. Dire on peut y aller comme ça, mais c'est prendre un risque de rejet plus grand ». J'ai dit : « non on va pas faire tout ça, puis après que je passe l'arme à gauche pour une connerie ! ». Bon ben alors, ben alors, de 15, une quinzaine de médicaments le matin, maintenant j'en ai plus que quatre, quoi. Plus le, plus les inhalateurs, donc, l'électrocardiogramme est beaucoup plus positif, donc y a moins de, y a, y a, y a plus de chance. Puis, puis le but c'est ça quoi, c'est vraiment de, d'arriver à améliorer ! Alors bon ben, dans la mesure du possible, je diminue les doses de morphine ! Y a des jours, ben comme aujourd'hui, j'ai pris juste du 20, j'ai pas pris le 30. On verra ce soir si je peux continuer comme ça. Par contre demain, je sais que si je vais à X *(ville)*, je prends du 30 le matin et du 30 à midi avant de partir. Et puis je reprends une dose de 30 sur moi, si nécessaire, si j'ai besoin. Parce que, laisser venir la douleur qui soit tellement forte que vous arrivez plus à causer, ça je veux, mercredi j'ai été dans cet état-là, je ne pouvais plus causer ! Alors c'est ma, ma compagne elle est arrivée. Moi je voulais pas, je voulais insister encore. Elle est arrivée, elle m'a foutu la pilule devant moi, puis elle m'a dit : « maintenant tu la prends, quoi ». Puis une heure après la douleur a disparue, puis j'ai pu, j'ai pu donner. J'arrivais plus à articuler, tellement j'avais mal. Donc, elle m'a dit : « non, c'est ».

**C : Ouais, et vous avez [aussi.**

**A :** Qu'on aille] à la greffe d'accord, mais qu'il faut mourir de douleurs avant pour arriver à la greffe. Ou alors on appelle Exit tout de suite. Mais y a des moments ! C'est dur ! C'est trouver un équilibre là-dedans. C'est ce que m'a, ce qu'il m'a expliqué, puis ce que m'a dit mon, enfin les deux toubibs, ils m'ont dit qu'ils comprenaient pas comment la force de résilience que j'ai. Mais moi-même je sais pas pourquoi j'ai ça. Je sais que je veux, je me rends compte par rapport à toutes les douleurs, toutes les vacheries et tout ce que j'ai supporté, tout ce que j'ai subi, enfin tout ce que j'ai subi… j'arrive à garder l'espoir puis à être heureux malgré tout ! Et ça les.

**C : Vous avez cette force de résilience.**

**A :** Ma soeur était infirmière, qui a aussi, une expérience de vie qui est pas banale. Elle, elle est complètement, elle est, elle est, quand elle a vu, elle s'est fâchée avec moi à cause de ça ! Elle me cause plus ! Alors qu'elle est, pour dire, elle a été, elle a été choisie par la X *(association)*, la X *(association)* lui a offert, lui a trouvé la bourse, pour faire infirmière, alors qu'elle était simplement aide-ménagère pour la X *(association)*. Ils ont trouvé qu'elle avait des mains d'or, puis qu'elle s'occupait bien des petits vieux, ils lui

ont payé, ils lui ont trouvé la bourse, c'était la X *(fondation)* pour qu'elle fasse les études d'infirmière. Là elle a bossé pendant quelques, huit ou dix ans à X *(hôpital)*, en X *(service)* pour fin de vie, pour accompagner les fins de vie. Puis maintenant elle est dans un centre, en ville, toujours pour, comment ça s'appelle X *(service)*. Donc elle est censée bien connaître les, voir les, les, les résiliences, fin de vie et compagnie, tout ça. Donc, elle est censée avoir l'habitude de ça ! Puis quand elle voit comment moi je réagis avec les douleurs que j'ai, et tout, elle arrive pas à me regarder dans les yeux ! Elle comprend pas ! Elle sait pas, elle sais plus quoi dire ! Donc elle a coupé complètement, elle a coupé les ponts. Puis bon moi, après les vacheries qu'ils m'ont fait par rapport au décès de mon père, j'ai jamais essayé de la re contacter, vu que pour moi elle n'existe plus, donc c'est une partie de ma vie qui est, alors que c'était pas seulement ma soeur, c'était, ça a été ma meilleure amie pendant, pendant 40 ans ! Et puis maintenant, bon ben, ouais, elle est là, elle habite à X *(lieu)*, moi j'habite ici, puis c'est comme ça, quoi.

**C : Donc voilà, vous avez ces, ces contacts un peu coupés avec [votre famille.**

**A :** Complètement.]

**C : Vous disiez que vous avez toujours ce soutien de la belle-famille, et.**

**A :** J'ai, j'ai le soutien de la belle-famille, mais du côté de ma famille, y a plus rien, quoi. Si, j'ai, non c'est pas vrai, j'ai encore un oncle et une tante qui ont 80 ans, que, je téléphone toutes les deux semaines, on rigole pendant une heure au téléphone. C'est des personnes charmantes, et puis bon, eux ils connaissent tous les deux son histoire, ils s'en foutent des, des ouï-dire et des qu'en-dira-t-on et compagnie. Donc ils ont un bon contact avec moi et puis ça s'arrête là, quoi. Non j'ai de la chance, j'ai vraiment, ouais je vois pas la, je vois pas la vie en rose, non, je la vois, je la vois telle qu'elle est mais, j'ai envie d'avoir ce, ces, ces.

**C : Envie de, d'aller de l'avant, de poursuivre.**

**A :** Ouais !

**C : Est-ce qu'y a, est-ce qu'y a d'autres choses que vous aimeriez encore rajouter par rapport à la BPCO, ou ce dont vous auriez besoin en termes de, [d'informations ou traitements, prises en charge ?**

**A :** Non. Non, entre l'information] comme j'ai eu la chance d'être soigné par le *Docteur D*, donc qui était responsable des, des urgences, c'est lui qui m'a sauvé la vie, parce que, quand je suis arrivé, ils ont mis sept minutes, depuis que ma compagne a téléphoné à ambulance. Ils ont mis sept minutes, l'ambulance était juste à côté dans, dans le quartier. Sept minutes pour arriver et m'amener, à me mettre sous masque oxygène d'abord. Puis après, au bout de 12 minutes, j'avais le masque d'hélium. Et là ils m'ont sauvé la vie, parce que, ouais. Et en plus, pour dire, c'était vraiment une connerie, mais, je venais de faire le vaccin le X *(date)* août, pour, pour la grippe, comme toujours. Puis les deux chenapans, mes petits enfants sont arrivés fin octobre, ils avaient la grippe, mais je me suis pas méfié, j'ai fait le vaccin, et tout. Mais ils avaient la grippe juste, en dehors du vaccin, juste celle qui était juste pas couverte ! Trois jours après, je me suis levé, c'était dimanche matin, je me suis levé à six heures pour aller aux toilettes, je suis jamais arrivé aux toilettes. Je me suis, en me levant je me suis évanoui. Puis là ben j'ai plus rien compris, quoi. J'ai fait sept jours de soins intensifs, où ils ont réussi de nouveau à faire une, j'accumule la poisse, vraiment. Au bout de cinq jours j'étais là-bas, puis ma compagne n'a pas réalisé, n'a pas pensé non plus. Au bout de cinq jours j'étais là-bas je suis sorti des, parce que j'étais, ils m'ont fait des mélanges de morphine, de, comment ça s'appelle, de masques, de, de masques, j'hallucinais. Je me voyais mourir d'une part, je voyais ma vie qui défilait devant moi, et j'ai halluciné complètement. Et puis j'avais des angoisses, mais terribles ! Et puis je m'étouffais, je m'étouffais ! Et c'est au bout de cinq jours j'ai dit : « antibios, antibios, antibios ». Puis là ils ont demandé pourquoi à ma, ma compagne. Puis là elle leur a dit : « mais ça fait trois semaines, je vous ai dit, ça fait trois semaines qu'il est sous antibiotiques, qu'il a une bronchite qui lui remplit ». Alors là ils se sont décidés à me mettre sous antibiotiques en intraveineuse. Donc en deux, trois jours, ça m'a dégagé les poumons, j'ai pu de nouveau re cracher, et puis ça a fluidifié, puis j'ai pu de nouveau me vider les poumons. Puis je leur ai demandé mais les angoisses, hallucinations, angoisses ! Alors là ils se sont décidés à me donner du X *(anxiolytique)*, mais. Puis là j'ai, les hallucinations, les angoisses, sont parties. Mais je suis resté pendant cinq jours avec des hallucinations puis des angoisses, mais à chaque seconde, chaque seconde tu te vois mourir ! Puis, ma vie déroulait, c'était comme, j'avais les, voyez des, des, des, les dés à six faces, sur chaque face du dé y avait une, un

petit écran de cinéma, et puis une partie de ma vie qui déroulait. Puis je voyais les dés qui roulaient, chaque fois je revoyais le même dé, ben je voyais la suite de l'histoire et compagnie. Et j'ai eu ça pendant cinq jours ! Tous les dés qui s'écroulaient partout, puis, puis dans des gerbes de sang, des explosions, enfin vraiment le, comme, comme dirait un, un drogué, un bad trip, vraiment le, le mauvais voyage, quoi. Donc les angoisses, cinq jours comme ça, cinq jours. Et là j'ai perdu six kilos, cinq jours, j'ai été.

**C : Puis après on a pu traiter vos angoisses [avec le X *(anxiolytique)*.**

**A :** Puis après ben,] ben après ils m'ont donné, au bout de cinq jours ils m'ont donné des trucs contre les angoisses, et puis contre, contre la, contre la bronchite que j'avais. Puis trois jours après ils ont pu me sortir des soins intensifs, puis m'amener en haut au, au septième étage ! Mais je suis resté de, sept jours aux soins intensifs, quoi. Mais il a, il a fallu cinq jours pour qu'ils se rendent compte que j'étais en train de m'étouffer, parce que j'avais une bronchite ! Et ils avaient même pas, ils avaient vu que j'avais la BPCO, c'est une chose, mais que j'avais encore la bronchite, puis que j'avais les voies qui étaient surinfectées. Alors là ben ils m'ont mis tout de suite. Puis après bon le *Docteur D.* il est venu s'excuser il me dit mais ils ont complètement passé à côté de ça ! Ils ont pas vu du tout que j'étais encombré, en plus de, de la diminution de la capacité pulmonaire, que j'avais en plus une bronchite par-dessus. Ça ils avaient passé à côté, quoi. Alors après, bon j'ai fait quatre semaines là, puis après j'ai fait donc le, le, X *(hôpital)* avec les aventures que je vous ai décrites avant. Et puis après, ben une année après ça a recommencé quand y a eu de nouveau les pollens. Ouais, pendant toutes ces années, en plus j'avais des allergies, donc je me grattais, ça me démangeait, et tout, j'avais, j'étais, je prenais X *(médicament)*, et compagnie. Mais en définitive, heureusement, mon médecin traitant, *Docteur E.*, il est, *(toussotement)*, il a eu la curiosité de demander à un laboratoire de X *(ville)* de faire une analyse comparative entre les différents médicaments que je prenais. Trois jours après il avait une lettre, un express, et tout, comme quoi il fallait absolument que j'arrête ce médicament, parce que ça faisait une contre-indication par rapport à l'autre. C'est censé donner des démangeaisons très, très graves. Ça faisait juste cinq ans que j'avais ce mélange de médicaments, puis que je prenais des X *(médicament)*, et d'autres trucs contre les, soi-disant les pollens, ou les fleurs et compagnie. A l'époque je pouvais pas traverser un magasin ! Si y avait un magasin de fleurs à l'entrée de X *(magasin d'alimentation)* ou n'importe quoi, je pouvais pas y aller ! Si je traversais avec les, les fleurs et tout, je me grattais pendant une semaine, après ! Et puis je me grattais, mais au sang ! Et là, changé mes médicaments et tout, maintenant je peux de nouveau, ben je fais de nouveau le jardin, donc je peux travailler dans le pollen et tout, je peux aller dans les magasins de fleurs. Enfin quand je suis capable de sortir, si xxx, si on m'amène des fleurs ici, je peux avoir des fleurs ! Avant, pendant ces années, c'était interdit d'amener des fleurs à la maison !

**C : Vous disiez vous avez accumulé un peu de la poisse.**

**A :** J'ai accumulé, mais, y a tout, tout qui m'est arrivé dessus ! Puis bon, puis, puis à force d'accumuler, ben tu, tu développes cette capacité de résilience. T'es obligé de… Ouais de te dire, ben t'avances ! Tu te laisses pas aller, t'avances ! Parce qu'autrement ben tu, ici c'est facile, c'est, tu ouvres la fenêtre, tu sautes, quoi. Avec le $11^{ème}$ étage, y a, y a pas plus facile, ça fait, c'est deux secondes, et puis c'est fini, quoi. Donc, mais non ! Non ! J'ai envie, j'ai envie de plus ! J'ai envie encore de croquer la vie à pleines dents, et puis de.

**C : Envie de poursuivre.**

**A :** Ouais, j'ai envie d'aller plus loin, j'ai envie d'aller plus loin ! Malgré les douleurs ! Bon, maintenant le problème c'est qu'il faut, c'est ce que je verrai demain, dans quelle mesure il faut que j'essaie de diminuer les douleurs ! J'ai, bon j'ai déjà, ils ont le papier de, X *(hôpital)* avec la liste des, des scolioses, des arthroses et compagnie, mais ils m'ont pas vu. Quand ils me verront, quand ils verront ma capacité à, à résister à la douleur et pis les, les sommes de douleurs qu'y a, parce qu'y a pas que le dos, y a, les épaules, les poignets, les mains. Ben on pourra discuter de tout ça, puis, puis voir avec eux demain, ben demain à la même heure, je serai fixé par rapport au traitement que je peux avoir, savoir si j'ai le droit de prendre plus, si c'est, si c'est admissible ou, ou s'il veut, ou s'ils ont, alors d'autres produits, peut-être !

**C : Vous verrez [demain comment ça va se passer.**

**A :** Donc demain à la même,] demain à la même heure je serai fixé, je saurai, je saurai ce qu'il se passe, je saurai ce que je peux faire pour passer le cap, aller plus loin, tout en ayant quand même un certain confort ! Parce que là maintenant, si je dois supprimer les, baisser les doses, c'est, c'est, c'est encore plus

inconfortable que maintenant, quoi ! Donc.

**C : Vous verrez [demain ce qu'on peut faire pour.**

**A :** Demain le, le choix qu'on peut faire,] oui.

**C : Pour poursuivre.**

**A :** Ouais.

**C : Mais je vous, je vous remercie beaucoup de, [d'avoir pris le temps de partager votre expérience. Je sais pas si vous aviez autre chose.**

**A :** Mais je vous en prie ! Non, puis moi c'était dès le départ que,] que l'infirmière, je sais plus comment elle s'appelle, une dame un petit peu potelée, très, très sympa, très, très souriante.

**C : Dans les, dans les infirmières y avait Julie *(infirmière de recherche)*, y avait.**

**A :** Je connais pas les prénoms, je saurais pas vous dire, non.

**C : Claire (*infirmière de recherche*) ou Laure (*infirmière de recherche*).**

**A :** Claire, Claire *(infirmière de recherche)*. Très, très sympa, j'ai trouvé vraiment. Et puis bon, bon moi j'ai, j'ai une facilité à parler, à m'exprimer à, je dis pas à sortir mes émotions, ça c'est un peu plus dur, parce que maintenant, ouais je bloque pour pas me laisser déborder. Ça c'est ce qui, ouais, faut mettre un frein quoi, on peut pas, autrement après c'est *(rires)*, vous vous arrêtez plus, quoi. Mais, je trouve c'est normal, tous les efforts qu'y a de recherche, de, de, de, de. J'ai vu mourir des gens à l'hôpital. J'ai des gens qui sont morts à côté de moi aux soins intensifs, j'ai des gens qui sont morts dans la chambre où j'étais. J'en ai un qui m'a beaucoup touché, j'ai vu l'avis dans le journal, X *(prénom et nom)*, je suis resté sept semaines à côté de lui. C'est moi, c'est sur mes conseils qu'il a acheté l'ordinateur, c'est moi qui lui ai installé les programmes, qui lui ai appris à l'utiliser. Là j'ai vu il est décédé y a trois semaines. Ses parents habitent ici à côté. J'ai commencé dix fois la lettre pour sa maman, pour, pour lui dire, ouais, mais j'en ai marre, ouais. Je l'ai, je l'ai aimé pendant sept semaines.

**C : Difficile, ouais.**

**A :** Ouais, ça pourrait être moi. Il attendait un foie, mais il est pas venu. Et il buvait pas ! Il avait une maladie du foie, il buvait presque jamais. Et moi j'ai bu pendant des années, et puis des grosses doses, et j'ai le foie d'un bébé. Comme au niveau des oreilles, j'ai fait un contrôle des oreilles l'autre fois, deux fois de suite, une deuxième fois, puis la nana elle est ressortie, elle m'a dit : « c'est pas possible, vous avez quel âge ?! ». J'ai dit : « j'ai 64 ans ». Elle a dit : « vous avez les oreilles d'un enfant de cinq ans qui vient de finir sa croissance ! Vous entendez mieux qu'un enfant de cinq ans qui finit sa croissance ! ». Elle a dit elle a jamais vu ça. Alors maintenant elle m'a écrit un petit mot récemment, elle veut que je participe à une étude sur la, j'ai dit : « oui, mais après la greffe. Pour le moment, je peux pas faire deux choses à la fois ». Mais après la greffe je suis d'accord de participer à son, à l'étude, si, si ça peut, si ça peut aider.

**C : Ouais. Je crois qu'on peut arrêter l'enregistrement.**

**A :** Ouais.

# Participant n°17

| |
|---|
| Nom d'emprunt : Henri |
| Âge : 81 ans |
| Sexe : M |
| Groupe : Contrôle |

**C : Vous avez participé à une étude qui avait pour objectif de comparer le traitement habituel de votre maladie pulmonaire, la BPCO, avec une prise en charge précoce, soutenue et intégrée. Vous avez été dans le groupe bénéficiant des soins habituels. Pouvez-vous me dire comment ça s'est passé ?**

**H :** La participation ?

**C : Mmh.**

**H :** Ecoutez je l'ai fait par, comment dire ça, sympathie, pour, pour les personnes qui doivent faire ce type de travail, que je trouve ennuyeux, embêtant *(rires)*, et, mais qu'il faut faire quand même ! Mais voilà, par sympathie, simplement. Je ne m'attendais à rien du tout, personnellement, rien du tout !

**C : Vous aviez pas d'attentes personnelles, [en participant ?**

**H :** Non.] Je l'ai, enfin, au moins la partie qui m'a correspondu, était tout à fait, je crois que inintéressant, puisque rien était proposé à mon égard. Simplement, dire comment ça allait, ça ne me conduisait pas à grand-chose, ou à rien du tout, plutôt.

**C : Y avait, y avait pas de propositions, et, donc cette partie était plutôt inintéressante pour vous ?**

**H :** Voilà. Mon expérience avec les, les médecins, qui m'ont traité, avec les spécialistes d'abord, n'était pas très réussie, puisque j'avais l'impression, d'être utilisé ou être une source de, de financement, plutôt que un patient à traiter pour le guérir, mais pour l'entretenir, en me faisant faire des consultations, toujours la même chose et, sans résultats positifs ni négatifs non plus ! Enfin j'étais, laissé en, à la réaction de ma nature, sans, sans autre. Et, les médicaments que j'ai pris et que je prends, je crois qu'ils n'ont fait pas d'effets. Et, cependant je n'arrête pas la médication, voilà, mais, mais je le fais avec le sentiment, et d'après mon expérience bon, par la suite, j'ai appris que c'est une maladie incurable. Alors je me suis contenté de me dire : « bon voilà, tu prends les médicaments peut-être pour ne pas empirer, alors tu continues ! ». Voilà. Mais, de temps à autre j'ai eu, des états bien, bien meilleurs que j'ai respiré normalement sans encombrements, sans bruits. Et, bien que je devais m'arrêter parce que je n'avais pas assez d'oxygène pour continuer ma marche. Et, attendez que je ne me perds pas *(rires)*…

**C :** ***(rires)*****… Vous disiez par rapport aux médicaments que vous les [preniez.**

**H :** Voilà.] Donc j'ai continué à, à prendre ces médicaments sans, sans, sans rien changer, sauf, sauf un élément. Mon, mon, mon, mon médecin, je parle toujours de, de le spécialiste, m'avait prescrit le double des médicaments que je prenais. La première fois je l'ai mis au courant, mais il a été furieux ! Alors je lui ai menti par la suite. Il m'a dit : « bon, vous prenez tel et tel médicament comme ci, comme ça ? ». J'ai dit : « oui ». Mais c'était pas vrai. J'ai pensé que, dû à ma taille et mon poids, étaient très disproportionnés à la dose que il m'avait prescrit. Et, j'avais des effets secondaires, et, et je ne voulais pas vivre ces effets secondaires ! Lui, le médecin il prétendait. Mais le jour que j'ai, lors de la consultation je venais avec des bonnes nouvelles de, de respiration. Il m'a dit : « vous voyez il y a des cas où on peut guérir ! Bien que c'est incurable, vous pouvez guérir ! ». Alors ça m'a donné du courage, pour persévérer. Mais, ça m'a pas fait me détourner de prendre toujours la moitié des médicaments que il m'avait prescrit, de la dose qu'il m'avait prescrit. Et, ce spécialiste a pris sa retraite, et il m'a confié la personne qui allait lui succéder. Mais je ne suis pas allé, je ne suis pas retourné chez le spécialiste. Et j'ai pris un médecin de, de médecine générale, qui habite ici à, à X *(lieu)*. Alors j'évite tous ces déplacements que je devais faire. Et ce médecin il m'a dit, et ça le, le décrit très bien. Il m'a dit : « je suis un médecin de campagne ». Alors on s'entend très bien parce que on se sert du sens commun. Et, et voilà ça va beaucoup mieux avec lui, bien que on a rien touché du,

du plan de, de médication du, du spécialiste. On l'a pas changé. Cependant, j'étais d'accord avec le nouveau médecin de continuer à ne prendre que la moitié... *(il reprend sa respiration)*. Une fois j'ai eu un accident… c'est-à-dire que le, l'antibiotique que je devais prendre en cas de fièvre, était périmé. Et j'étais tellement malade, ça allait de, de minutes en minutes qui étaient pires ! Le médecin des urgences, est venu et tout de suite il a demandé de venir le cardiomobile. Et grâce à ça je suis encore vivant. Donc quand j'étais à l'hôpital, là-bas ils ont aussi voulu me changer le, la médication. Ce qu'il s'est passé c'est que je n'ai pas pris de médicaments quand j'étais à l'hôpital. On me donnait pas de médicaments. Et *(toussotement)*, enfin j'étais dans, dans une machine qui a fait je ne sais pas quoi. Mais, moi je n'ai pas pris de médicaments par la bouche. Les piqûres et autres, parce que j'avais un masque etc. Alors je ne sais pas si j'ai pris des médicaments ou pas. Le fait c'est que en sortant de l'hôpital, j'ai continué mon, mon rythme de, de médication. Et, les résultats sont les mêmes, dans ma maladie. J'ai, il y a des hauts et des bas, périodiques. Et, actuellement, depuis lundi dernier, pas ce, ce lundi-ci, aujourd'hui, mais lundi de la semaine passée, je respire bien, sans bruits, sans difficultés. Donc, je dois pas ouvrir la bouche pour respirer. Et, j'ai pas de fièvre, bien sûr, parce que je respire bien. Et, et je ne prends pas d'antibiotiques. Donc… comment dire, comment continuer ça, je suis, oui dans, depuis une semaine, j'ai mes, je, je suis beaucoup mieux. Et ça me fait penser que je pourrais, un jour, si j'ai le temps, de guérir. [Voilà ça c'est en, en résumé mon, mon vécu.

**C : Donc là vous avez des hauts.] Par rapport à la BPCO. Donc vous avez ces, ces hauts et ces bas. Et là c'est une période où vous êtes plutôt bien et vous avez cet espoir de pouvoir guérir peut-être un jour.**

**H :** Bien que je dois m'arrêter, je dois pas faire des longs trajets sans, je peux pas courir non plus. Je peux pas tout nettoyer chez moi, d'un seul coup, je dois m'arrêter, régulièrement. Mais, disons j'ai, j'ai beaucoup moins d'expectorations, presque rien du tout, par jour. Et ça me donne de l'espoir.

**C : Comme vous disiez c'est une maladie incurable, [mais on peut guérir.**

**H :** Oui.] Peut-être !

**C : Et, et vous avez ce médecin aussi de campagne qui vous suit maintenant.**

**H :** Oui, il est beaucoup plus, comment dire ça, moins spécialiste, donc plus terre-à-terre. Et, même ces factures sont, sont beaucoup plus normales *(rires)*. Les factures de l'autre médecin étaient très fréquentes et assez élevées. Et, voilà ! Donc je suis content du changement. Lui, il m'a dit : « mais monsieur *(nom)*, si vous voulez un, un spécialiste, être suivi par un spécialiste, je peux vous donner des, des bons adresses ». J'ai dit : « non ». Puisque voilà. Personne va me, me prescrire d'autres médicaments. Et pour être contrôlé, moi je sais très bien comment je me trouve et quelle est ma, ma, mon état ou ma situation !

**C : Vous évaluez vous comment vous vous sentez.**

**H :** Voilà, le spécialiste m'avait, m'avait éduqué pour ça. Alors maintenant je me sens bien formé, pour ne pas être toujours pris dans la main du médecin.

**C : Ça vous vous sentez formé par rapport à la BPCO et, et [en termes.**

**H :** Par rapport à la**]**, oui à comment attaquer, en cas de, en cas de crise, en cas de problème, oui.

**C : Et aussi en, en termes d'informations, par rapport à votre maladie pulmonaire ?**

**H :** Oui ! L'information c'est moi plutôt qui la fournissait au médecin ! C'est pas lui qui m'informait ! J'ai appris avec lui à ne pas m'affoler, par exemple, en cas de, de d'obstruction. Mais, voilà. Il y a des moments où, où ça a été très, très, très dur, très difficile d'expectorer. C'est des moments très, comment dire ça, qui font peur, hein ! On croit qu'on va s'étouffer. Mais bon, comme j'ai personne qui puisse me taper derrière, alors je me contente avec les moyens que, qu'on a, tout naturellement pour, pour expectorer.

**C : Mais, mais ça fait peur ces moments de, d'obstruction.**

**H :** Oui. Parfois ça fait, ça fait peur, oui *(rires)*.

**C : C'est effrayant.**

**H :** J'ai froid tout le temps, hein, excusez-moi.

**C : Ah non, c'est bon, si, si vous avez besoin de, d'un pull ou quelque chose, hésitez pas. Soyez confortable *(rires)*.**

**H :** Je ne sais pas pourquoi. Vous, vous sentez froid ici, chez moi ?

**C : Ça va, [ça va !**

**H :** Ça va.]

**C : Oui, il faisait, il fait assez froid dehors, du coup, [c'est peut-être ça.**

**H :** Moi j'ai froid !] Mais toujours, hein, toujours ! Je sais pas pourquoi je tremble.

**C : De temps en temps, oui. Et, donc en termes d'informations, est-ce qu'y aurait des informations supplémentaires, par rapport à votre maladie pulmonaire, dont vous auriez besoin ?**

**H :** Je ne crois pas. Je suis, je ne suis pas un spécialiste, hein ! Mais je ne crois pas parce que non. L'hôpital n'a pas corrigé le, la médication que, que m'avait fait, prescrit le, le spécialiste ! Donc, et le médecin actuel, il dit : « continuez comme ça ».

**C : Pas de, pas de besoins particuliers, par rapport à, à l'information.**

**H :** Et ma maladie évolue comme toujours ! Avec des hauts et des bas. Sans, sans améliorer, disons la source de la maladie, sans la changer.

**C : C'est toujours la même évolution avec des hauts et des bas.**

**H :** Voilà. Toujours, tout le temps ces, ces manques d'air que, que on a l'impression qu'on va étouffer *(rires)*.

**C : Et vous disiez que vous avez pas quelqu'un pour vous, pour vous taper dans, dans le dos *(rires)*. Quel soutien vous avez de la part de vos proches, par rapport à, à la maladie ?**

**H :** Soutien, je ne, je ne vois pas. Il y a mon fils qui me téléphone de temps en temps pour prendre des nouvelles. Et, on va, de temps à autre manger au restaurant. Mais, mais c'est tout ! J'y pense, parce qu'une fois un médecin de médecine générale, qui m'avait donné l'adresse de, du spécialiste, m'a, nous avait, à ma femme et moi, nous avait donné des instructions pour expectorer. Et je me rappelle qu'une, une des choses qu'il avait insisté beaucoup c'est de, de me mettre tête en bas, et frapper au dos, pour faire décoller les phlegmes. Ça m'a resté. Mais, jamais ma femme a eu l'occasion de pouvoir le faire. Et actuellement, y a personne qui puisse, qui puisse le faire. Et je me débrouille. Je me débrouille en ne m'énervant pas, quand la crise arrive. Je, j'essaie de faire tout, calmement.

**C : Vous arrivez à rester calme pendant la crise, à vous calmer.**

**H :** Oui. A ne pas, non le mot c'est, ne pas me, m'énerver, non, me, de ne pas m'angoisser non plus. Bien que quand c'est, ça insiste, ça, ça bloque, oui j'ai eu des moments difficiles.

**C : Pendant les crises.**

**H :** Oui. Et on pense tout le temps quand est-ce qu'on va mourir. Ça, ça est aussi évident ! Et on se pose la question du comment, pas du quand, mais du <u>comment</u>. Mais il n'y a pas de réponses, personne peut répondre à cette question. Donc, voilà.

**C : Vous vous posez cette question de, du comment vous allez mourir ?**

**H :** Oui *(rires)*. Une fois, j'ai parlé même à, au voisin dans le café parce que je sors tous les jours, pour prendre un café, le matin. Et, au bistrot, c'est pas le bistrot c'est le restaurant de, de X *(entreprise)*. Alors le, la personne qui est à, dans la table à côté, je lui ai fait des commentaires, concernant cette question, qu'on, qu'on se pose comment, comment est-ce qu'on va mourir. Et lui il m'a répondu : « heureusement ce sera toujours une surprise ». Alors j'ai, j'ai gardé ça pour moi comme une réponse à ma question *(rires)*. Ça sera toujours une surprise ! On verra, donc, à pas se faire du souci pour le moment, inutilement, parce que on va pas changer le résultat ou l'événement.

**C : C'est la surprise, oui. Est-ce que vous avez pu parler de ça avec des professionnels ou des, ou du médecin qui vous suit ?**

**H :** Non, non. Ils pourront pas m'apprendre grand-chose, puisque j'ai, mon métier c'est pasteur. Et j'étais aumônier à l'hôpital. J'ai vu mourir beaucoup de personnes. Je n'utilise pas le mot accompagnement, parce que c'est risqué de dire ça, mais j'ai vu mourir beaucoup de, de gens. Alors, je sais qu'il y a beaucoup de, de, chacun a sa façon et ses circonstances, à, à passer avant la mort, donc ça se répète <u>pas</u> ! C'est toujours très, très différent. Et, un médecin, même si c'est un médecin, ou une infirmière qui n'ont pas vécu la mort des autres, ne peut pas me dire à moi quoi que ce soit, à ce sujet. Parce que, à l'hôpital il m'est arrivé en général d'être tout seul auprès des mourants, sans la présence de, de quiconque. Alors, je, je n'attends pas grand-chose sur ce point de, de quelqu'un.

**C : Vous attendez pas un soutien.**

**H :** Et même je, quand j'entends des conférences, à ce sujet, c'est pour ça que j'ai, utilisé le mot accompagnement tout à l'heure. Il y a des conférences, des gens d'église ou, ou pas d'église, qui visitent les malades, qui parlent beaucoup d'accompagnement. Et moi, je crois que leur discours est une grand mensonge. Parce qu'ils n'ont pas de, <u>pas du tout</u> une expérience, à ce sujet ! Ça je l'ai vu et je l'ai vécu, à l'hôpital. J'ai vu la personne qui donnait la conférence qui n'a jamais été auprès d'un malade, et pourtant, parler avec autorité de ce sujet. Aide, une aide je ne peux pas attendre de, de quiconque, non.

**C : Faut pouvoir avoir expérimenté pour pouvoir accompagner quelqu'un.**

**H :** Parce que je sais ce qu'on <u>peut</u> savoir, et ce qu'on saurait <u>pas</u>, jamais... Même en voyant quelqu'un mourir, on sait jamais ce que c'est ! Parce qu'on n'est <u>pas</u> à sa place ! On est observateur, mais pas à sa place !

**C : Comme on peut pas être à la place de l'autre, vous aimeriez pas ou vous souhaiteriez pas avoir du soutien spirituel, par exemple, ou ?**

**H :** Non, non, j'ai pas besoin, non, non. Maintenant, y a un collègue qui m'écrit et, si, je dois répondre. Non, non, je n'ai pas besoin de, dans ce domaine, non. Vous savez même, j'ai, j'ai fait des, des expériences que <u>peu</u> de gens, au moins autour de moi, ont, ont fait. Ma femme a été une année à l'hôpital. Elle a été opérée d'une hémorragie cérébrale. Et une année après, sans quitter l'hôpital, elle est décédée. Et, je suis allé <u>tous</u> les jours, <u>tous</u> les jours, <u>toute</u> la journée, j'étais avec elle. Et, j'ai beaucoup lu, au sujet de la mort et de l'au-delà. Et j'ai dit à ma femme… Je crois que c'était trois jours avant, avant son décès. J'ai dit à ma femme, ce qui a laissé passer, lors de sa mort, ou plutôt après, après sa mort. Parce que elle avait toujours peur du <u>comment</u> mourir. Alors je lui disais que je ne pouvais pas lui parler de cela, parce que on sait <u>pas</u> comment on va mourir. Et d'après mes lectures, je lui dis : « je peux te dire l'expérience qu'ont fait d'autres personnes qui sont revenues de la mort ou qui ont fait une sorte de mort ». Et lors de, de cours de psychologie médicale qu'on donnait à X *(hôpital)*, et aux cours où j'allais, il y a eu des patients qui ont parlé de leur expérience de la mort, pas du fait de mourir, mais de ce qu'ils ont vécu, étant morts. Et j'ai lu d'autres, l'expérience d'autres qui ont été publié. Et tant protestant que catholique. Et, et j'ai annoncé à ma femme que un ange ou plusieurs viendraient la chercher. Je lui ai dit : « une fois morte, tu ne bouges pas, du lieu où tu es ». Je ne lui ai pas dit de, de rester à mon côté avec moi. Je lui ai dit comme ça : « tu ne bouges pas, parce que un ange ou plusieurs viendront te chercher »… La, la malade, ou les patients qui étaient dans le lit à côté de, de ma femme, me disaient que je pleurais comme une sorte de, de ruisseau,

j'avais des larmes énormes qui, qui, qui coulaient de. Comme je parlais à ma femme en, en X *(langue)*, elles, elles ne savaient pas ce que j'étais en train de lui dire. Alors ce qui s'est passé c'est que ma femme a été incinérée, et j'ai compté les jours après. Pendant 40 jours, elle a été là *(il tape du poing sur la table)*, avec moi. Mais je l'ai vue, hein ! J'ai vu une, une boule comme de tennis comme ça *(il montre la taille avec ses mains)*, à peu près ça, cette grandeur-là, blanc, brillant, mais d'un blanc qui ne m'aveuglait pas, quand je regardais. Et tantôt on se croisait dans les couloirs, ou elle était à la cuisine quand je venais de, de faire les commissions à la X *(magasin)*, elle était devant le frigo. Ou bien elle, elle était là, elle, elle, elle m'observait ou elle me regardait. Et quand on se croisait, on se, on se frottait, et j'ai senti la chaleur de cette lumière qui se promenait chez moi. Et un jour j'étais là, ici, j'étais là comme ça, *(il se lève et montre l'endroit où il était)* j'ai regardé un, un livre que je venais de recevoir de, de xxx, j'étais en train de le fouiller comme ça, et, un bruit et quelque chose qui me touchait dans l'épaule. Alors, il a fallu un bon moment hein, pour que je, je réalise que, que quelque chose est passé. Et je regarde et, et la boule était là, moi j'étais là, et la boule était là *(il montre l'endroit)*. Et au bout d'un moment j'ai vu entrer par la fenêtre, un nuage, tout blanc, de la même éclat, éclat, éclatement de lumière que avait elle. Elle s'est mis ici *(il montre l'endroit)*, tout la paroi ici était pleine de lumière, de la même nature que ma femme. Et, le dialogue, en pensées qu'on a eu c'est : « voilà, c'est vrai ce que tu m'avais dit. Une, un grand nombre d'anges sont venus me, me chercher ». Oui le temps de cette phrase-là a duré leur présence ici, parce que tout de suite après, tout ce monde est parti par, par le même endroit, par la même fenêtre. J'étais tout, pas effrayé, mais étonné de voir cela. Et tout d'un coup je suis, j'ai réfléchi, je me suis rendu compte que quelque chose d'extraordinaire venait de se passer. Et j'ai couru à la fenêtre, j'ai ouvert pour regarder. J'ai vu disparaître, ce grand nuage a disparu, voilà là, en face. Après, j'ai pu comparer que j'avais un grand poids en moins. Parce que tout ce temps, qu'elle a été ici, sous cette forme, j'avais toujours comme une sorte de poids de sa présence. Indépendamment de si je voyais ou pas la lumière. Et ce poids, a disparu complètement. Mais alors c'est seulement à ce moment-là que j'ai décidé de prendre ses cendres et aller au caveau, on dit caveau, que a sa famille en X *(pays)*, ses parents et ses grands-parents ont un grand machin là-bas au cimetière. Alors je suis allé pour déposer ses cendres à elle. Et je n'ai eu aucune conscience de sa présence à elle, à côté de moi, qui était, je me sentais comme une plume, tout léger.

**C : Vous aviez plus ce poids.**

**H :** Alors, vous voyez je pourrais même aller lors d'une, de ces séances de psychologie médicale, aller à l'hôpital et parler de, de mon expérience à ce sujet. Et ça, a été pour moi une, oui, une réponse mais, bien que par mes lectures, je savais, mais je n'avais pas vécu. Et cependant, ça ne donne pas réponse à ma question du comment je mourrais.

**C : Ça reste une surprise.**

**H :** Voilà.

**C : Mais vous avez eu cette expérience-là.**

**H :** Oui ! Avant c'était tout de la théorie et de la croyance. Maintenant c'est quelque chose de vécu.

**C : Mais vous saurez [toujours pas comment.**

**H :** Il y a,] il y a une infirmière venait pour me prendre la tension et je lui ai raconté ça. Et elle ma recommandé la lecture d'un, d'un livre qu'elle m'a prêté sur ce même sujet. Et je suis reconnaissant parce que elle m'a dit : « au moins votre femme a tenu à vous prouver que ce que vous lui avez dit était vrai ». Et voilà ça, comme vous voyez ça m'accompagne.

**C : Ça reste avec vous. Donc vous savez toujours pas le comment, mais vous avez cette expérience-là.**

**H :** J'ai la confirmation que oui de, de la réalité matérielle de ce que je crois. Et, et voilà, tous ces personnes qui parlent de la mort et de l'accompagnement, des mourants et tout ça, ne savent pas ce qu'ils disent, non. Parce qu'ils n'ont pas fait, ils n'ont pas accompagné d'abord, personne, non. Je l'ai vu, ça, à l'hôpital, je l'ai vu. Et, ils n'ont pas eu d'expérience, aucune.

**C : Et c'est pour ça que vous avez pas.**

**H :** Même, même, ils n'ont pas parlé avec des malades de l'hôpital qui témoignaient de leur vécu après la mort. Même, même ça, ils ne savaient pas. Alors je ne peux pas, disons, demander à qui que ce soit de, de répondre à mes questions, parce que je sais que ils sont ignorants. Et s'ils osent parler, ils savent pas de quoi ils parlent.

**C : Donc vous voulez pas leur demander. Et je sais que on peut parler de, de la mort. Je sais que les professionnels peuvent parler aussi des, des soins qu'on désire en fin de vie. Et on peut aussi écrire avec eux ce qu'on appelle des directives anticipées. Je sais pas si vous avez entendu parler de ça, vous ?**

**H :** Non.

**C : Si, s'il arrive à un moment qu'on puisse plus s'exprimer ou dire [ce qu'on aimerait comme, comme soins.**

**H :** Ah, ah oui, ça, ça oui, ça oui.] Ça oui oui. Quand j'étais à l'hôpital, et je suis sorti des soins intensifs, un médecin est venu me demander ça. Mais ça c'est différent parce que le médecin il, il a pu me, me dire comment je pourrais me trouver, dans quelle situation je pouvais me trouver. Alors j'ai dit : « bon merci beaucoup, mais ce que j'aimerais c'est que si on me voit, on me voit en difficulté de mort, qu'on m'aide plutôt à mourir qu'à ne pas mourir ». Ça c'est nouveau à l'hôpital. Avant, quand j'étais à l'hôpital, et j'étais 15 années à X *(hôpital)*. Et, on posait pas ces questions aux patients. Et même pour le médecin était gênant de nous voir nous pasteur à côté de, enfin en visite de ces, de ces personnes. Bien que tout le monde restait très prudent, mais je voyais que c'était pas, c'était pas agréable de me voir.

**C : Les médecins étaient pas confortables.**

**H :** Non.

**C : Mais vous avez [pu.**

**H :** Mais parce que] les années que j'étais à l'hôpital, j'étais connu, bien connu. Oui, par mes, ma présence auprès des malades, et aussi par mes prédications à l'hôpital. Même les professeurs venaient voir, entendre ce que je, je disais. Bon, je leur apprenais pas, enfin, je, je pense que ils étaient au courant de, de ce que je pourrais dire, parce que ils ont fait des lectures au sujet de, de tout ce qu'un pasteur peut, peut dire. Mais ils venaient me, m'écouter. Surtout à Noël, quand je, je faisais un peu le Noël dans les unités. Alors la cohorte des, des, des médecins était là, ils étaient témoins.

**C : Donc ils venaient, ils venaient vous écouter.**

**H :** Oui. Et je me, je me sentais avec une autorité que eux ils n'avaient pas. Donc je me sentais supérieur à, à tous ces personnes dans mon, dans, dans ce, dans ce sujet-là. Et mon expérience de, de la mort était aussi très différent de, des médecins et des infirmières, parce que eux ils aband', ils fuyaient les, les lieux où étaient les, les morts, ou, ou, ou la personne qui allait mourir. Et moi je, je restais là. Alors je pouvais parler comment tel et tel était mort, et pas eux. Et pourtant eux ils parlaient, et moi pas. Je ne le racontais pas. Mais eux ils, oui, eux ils savaient comment il avait mort, il, il était mort. Bon.

**C : Ils fuyaient, et vous vous restiez, vous aviez cette expérience de la mort.**

**H :** Non mais, oui. Mais moi j'ai jamais, comment dire ça, j'ai jamais osé en parler si un médecin parlait de, de tel ou tel. Moi je restais silencieux.

**C : Et par rapport à vous, vous avez pu dire aussi que si vous arriviez dans cette situation vous aimeriez qu'on vous aide plutôt à mourir plutôt ?**

**H :** Oui piqûre, piqûre pour, pour m'en aller au plus vite possible, oui. Ça.

**C : Donc ça vous avez pu en parler, vous l'avez écrit également ?**

**H :** Oui, j'ai signé un papier que j'ai, que j'ai signé, oui, à l'hôpital. J'espère qu'ils vont enregistrer ça pour toujours.

**C : Et comment ça s'est passé pour écrire ça ?**

**H :** Bien. Pas de problèmes ! … Non, non… Et il y a seulement une fois une question dans les questionnaires concernant ce sujet.

**C : Dans l'étude ?**

**H :** Oui. Une fois le questionnaire posait la question, oui. Moi je trouve ça tout à fait normal et courant ! La, la seule, seul problème que je vois c'est que, bien sûr on sait pas si on va souffrir ou non, physiquement. C'est ça qui, que je crains. Parce que depuis la mort de ma femme… Et, comment dire ça… La seule, les seules craintes que j'ai, c'est la souffrance physique. Et… J'ai, j'ai dit souvent à mon fils : « je suis incapable de souffrir un peu plus, parce que j'en ai souffert tellement auprès de ma femme ». J'ai dit : « je suis, je suis incapable, je deviendrais fou, si je dois supporter encore de la souffrance physique ». J'ai dit : « j'ai perdu ma, la capacité qu'on a de, de souffrir ». Bon, ça fait maintenant 10 ans qu'elle est décédée. Et, comment dire ça, ce sentiment a disparu peu à peu chez moi. Je, je souffre, comment dire ça, je supporte mieux l'idée de souffrance aujourd'hui que il y a dix ans.

**C : Ça vous fait un peu moins peur maintenant que [y a dix ans ?**

**H :** La souffrance physique.] Ou d'imaginer que je pourrais souffrir physiquement, oui, ça fait moins peur maintenant que ces années passées. Cependant, ça reste une, une question !

**C : Comment on va mourir et la souffrance physique reste une question.**

**H :** Pas l'après, mais l'avant *(rires)* !

**C : *(rires)* Avant de partir. Et, si on regarde aussi en termes du, du soutien que vous avez pu recevoir. Quel type de soutien avez-vous reçu au niveau psychologique, par rapport à la BPCO ? … Rien ?**

**H :** Non… La dernière fois que je suis allé consulter le spécialiste, un jour ou deux avant sa retraite, il m'a parlé lui, de son, comment dire ça, son, son vécu, en tant que médecin, et il m'a parlé tellement que je n'ai pas osé lui parler de moi. Et on a oublié, lui et moi, de renouveler la, l'ordonnance. C'est pour ça que j'étais, lors de une crise, avec des médicaments périmés, parce que j'avais oublié de, de, de demander au médecin de renouveler l'ordonnance. Et, c'est, l'oubli a été dû au fait qu'il m'a parlé de son, attendez…

**C : De son vécu vous disiez.**

**H :** Oui mais c'est, je cherche un autre mot, parce que au fond, il m'a parlé de sa souffrance, parce qu'il ne supportait plus voir des malades. De son dégoût de voir des malades. Il était comme obsédé, il parlait : « je ne supporte plus les malades ! ». Il m'a parlé d'une façon très, très négative. Et je voyais que c'était en lui une souffrance énorme, et un dégoût total ! Alors moi je ne me prêtais pas après son discours à, à, à, à jouer des patients ! Alors j'ai dit : « bon alors bonne chance ». Et je suis parti, comme ça ! Alors je, je ne pensais pas, ni au contrôle, ni, ni à l'ordonnance qu'il devait me, me renouveler. Et, pour vous dire que du soutien psychologique, je n'attendais rien de personne ! De personne ! Ce médecin il aurait pu, mais, voilà. C'est, c'est après son discours d'adieu que j'ai compris pourquoi, le pourquoi de, des éléments de, de sa consultation. J'étais une personne qui passait par des machines, c'est tout. Et mes questions, toutes, étaient des, des questions erronées. Et, il me, me répondait toujours en me disant que c'était pas ça : « non, c'est pas ça ». Mais il m'expliquait pas le pourquoi. Même quand je lui dis : « votre balance donne quatre kilos de plus que la mienne ». Il m'a dit : « la, la vôtre est en dérangement. La bonne est le poids que vous marquez ici, pas chez, chez vous ». Alors il y avait une telle, comment dire, autorité mal, mal appliquée, ou mal comprise, que ça nous distançait de plus en plus. Peut-être comme j'étais si docile à, à son écoute, il

a eu la chance de, de me faire ce discours d'adieu, en me parlant de ce dégoût des, des malades. Pas de la maladie, mais de la personne, des personnes !

**C : Donc c'était plutôt le médecin qui parlait de ses souffrances plutôt que vous qui [avez.**

**H :** Voilà.] J'ai dû lui dire et lui parler de cela. Et je lui ai dit : « d'après mon expérience personnelle, en tant que pasteur à X *(lieu)*, voilà, j'en avais aussi assez, des jours où j'en avais assez de voir des malades. Mais je prenais congé. … Et je revenais quand je croyais que je, que j'étais bien ». *(rires)*. Mais bon. Donc voilà le soutien psychologique… Les profs qui donnaient des cours de psychologie à l'hôpital à cette époque-là, une personne qui était pour moi très, très sympa, mais je connaissais pas, que son, sa façade. Il s'est suicidé. Lui, qui parlait de suicide ! Il nous disait combien de suicides y avait à X *(ville)* et en X *(pays)*, par jour… Et il s'est suicidé. Pourtant c'était, je croyais, en tant que professeur de psychologie, peut-être, enfin, nous, nous nous sommes jamais parlé mais, entre nous. Seulement c'était le, le, l'apparence, le, ce qui est permis socialement de, de, de se dire entre deux personnes… Et, alors ma conclusion c'était que même un spécialiste en psychologie, était soumis à, à des problèmes insolubles. Alors je me, après je me suis dit : « comment peut-il aider les autres ? ».

**C : S'il a des problèmes insolubles.**

**H :** Et surtout s'il n'a pas d'expérience, sur la. Il, il ne voit que les, les côtés matériels de la déchéance des autres ! Et si, surtout si c'est quelqu'un qui ne, ne pense que à faire des grosses factures ! Et comment multiplier ces factures ! Alors il ne peut pas parler d'autre chose, il est incapable ! Pour moi il n'est pas crédible !

**C : Oui, donc, pour vous, vous avez pas reçu ou eu besoin de ce soutien psychologique, [par rapport à la BPCO.**

**H :** Si j'avais eu] des conférenciers qui étaient des mensonges parce qu'ils n'avaient pas d'expérience et des médecins qui, qui ne voyaient que des factures, alors je ne pouvais pas attendre quoi que ce soit ! Pour moi, personnel !

**C : Et si.**

**H :** Moi je prenais de l'air et la distance quand j'en n'avais pas, je n'avais plus de, de force ou d'équilibre personnel pour continuer ! C'est le seul remède. Ou bien vous démissionnez, ou bien vous vous aérez. Ce n'est pas la médecine radicale, c'est temporel, c'est *(rires)*. Parce qu'il arrive un moment où de nouveau ça se répète, si vous n'avez pas autre horizon que les malades ou la maladie plutôt, et la mort.

**C : Donc vous, vous preniez congé quand il fallait. Et, et si, si on revenait aussi à, à l'étude, et si, donc vous aviez été dans, dans le groupe qui avait le traitement habituel. Si vous aviez été dans le groupe qui bénéficiait de cette prise en charge globale spécialisée, avec une infirmière qui venait une fois par mois pendant un an, quels types d'aides et informations par rapport à votre maladie pulmonaire, auriez-vous souhaité recevoir ?**

**H :** On a déjà parlé, ces coups au dos *(rires)*. Aider à, à cracher. Et, je crois que, bon le plus urgent dans mon, dans mon cas, c'était besoin d'un aspirateur. Le spécialiste il m'a mis un aspirateur par la bouche, et c'était, après, j'étais bon pour quelques jours. Donc ça c'était la, la première aide que j'aurais demandée. La, la répétition de cet acte médical que j'ai eu une fois, c'est l'aspirateur, oui je crois que il y a d'autres noms pour ça. Mais, même on, on pouvait le voir par la télévision parce qu'il y avait aussi une, une caméra qu'on, qu'on met, à part de l'aspirateur. C'était très intéressant. Mais enfin c'est le fait d'être dégagé, ça c'est la première aide. Pour le reste, vous comprenez que je me sens assez compétent, pour le reste. Puisque il n'y a pas de, de médicament miracle… Et que, du côté moral, psychologique, spirituel, je n'attends pas grand-chose des autres. Donc voilà, c'est tout à fait, mon besoin personnel, l'aspirateur *(rires)*.

**C : Ça aurait l'aspirateur et avoir quelqu'un qui puisse répéter ce geste technique. Mais pour vous, pas [d'autres besoins ?**

**H :** Ou, ou les coups] au dos, hein *(rires)*.

**C : Oui ! Oui, donc pas de, pas d'autres. Donc ce que vous auriez besoin aussi aujourd'hui, ce serait plus ça ? Ou de quoi d'autre auriez-vous besoin ?**

**H :** Aujourd'hui ?

**C : Mmh**.

**H :** Mon problème actuel, c'est que je reçois mensuellement 300 francs de plus que la limite fixée pour avoir une aide ménagère. J'aurais besoin de quelqu'un pour, pour nettoyer, ce que je ne peux pas, ou que je fais très rarement et très peu à peu. Quand je nettoie ici, c'est déjà nécessaire de nettoyer là-bas, parce que je n'ai pas nettoyé là-bas, seulement ici. Alors quand je nettoie là-bas, c'est déjà urgent de le faire ici. Voilà. Pendant une période, j'ai payé une aide ménagère, mais, cela m'a empêché d'acheter des bouquins, deux ou trois que j'achète par mois, parce que je n'ai pas d'aide sociale pour payer l'aide ménagère. Et, moi je ne peux pas, je ne peux pas vivre sans lire. J'ai mon, ma vie dépend maintenant de la lecture et de l'écriture. Donc, voilà. C'est parce que je, je gagne 300 francs en plus de la limite, que, que je n'ai pas l'aide sociale. Et en plus, j'ai fait plusieurs tentatives, et la dernière fois, j'ai eu comme réponse de renseigner les autorités sur mon héritage en X *(pays)…* Donc voilà, j'ai pas répondu à cette, à cette demande, de mon héritage en X *(pays)*, parce que ça c'est une, c'est une, c'est une boule énorme, puisque ma famille, la famille de ma femme avait des moyens. Et elle avait son héritage en X *(pays)* à la banque, mais la banque, avait cet argent en action je ne sais pas où. Ces actions ont chuté, et il n'y a rien. Mais, le bruit court que, que je suis riche, par ma femme. Et il n'y a rien de, de, de plus mensonger que ça. Parce que voilà, on a tout perdu, bien que en X *(pays)*, il reste un petit reste de 300 francs, pour que tel compte continue à être, et mon nom figure sur la liste des personnes qui ont des biens à l'étranger. Alors, bon il y a plus encore pour, pour expliquer de cela, mais.

**C : [Ce qui vous manque, c'est.**

**H :** Voilà, je ne peux pas, je ne peux pas,] avoir d'aide. Et mon besoin c'est une aide. Même si c'est seulement pour enlever la poussière, voilà. Parce que le reste je peux le faire, lentement, peu à peu, je peux le faire. Mais tout, je ne peux pas.

**C : Donc il vous manque cette aide [sociale, qui vous permettrait d'avoir quelqu'un pour.**

**H :** Voilà.] Une aide matérielle.

**C : Venir vous aider à faire le ménage ou juste passer la poussière, oui. Merci. Si, si je résume un petit peu tout, tout ce que vous m'avez dit. Donc, par rapport à l'étude, vous avez participé pour participer à l'étude. Vous attendiez rien de spécial [pour vous.**

**H :** Par sympathie.]

**C : Par sympathie, voilà. [Pour les personnes qui mènent.**

**H :** Parce que j'ai fait, j'ai fait] des enquêtes, quand j'étais X *(métier)* à X *(institut)*, j'ai fait beaucoup d'enquêtes. Alors par sympathie à, à la, au fait de, de devoir faire des enquêtes, je l'ai fait.

**C : Voilà.**

**H :** On dit aujourd'hui empathie.

**C : Par sympathie et empathie vous avez participé comme vous aviez déjà vous-même dû faire des études. Donc c'est pour ça que vous avez participé. Vous aviez pas d'attentes pour vous par rapport à, à l'étude. Et bon, vous m'avez dit aussi que c'était un petit peu ennuyeux les, les questionnaires, mais que voilà, vous aviez participé.**

**H :** Cependant, j'aimerais bien connaître les résultats, pour voir de trouver une réponse à ma question ! Le pourquoi de cette étude-là !

**C : Donc voilà vous avez ces, ces attentes par rapport aux, aux résultats qu'on n'a pas encore**

**par rapport à l'étude, et vous [souhaiteriez les avoir.**

**H :** Oui.]

**C : Et de manière générale, par rapport aux, aux soins que vous recevez pour la BPCO, donc vous aviez eu cette expérience avec le spécialiste. Et puis maintenant vous êtes suivi par ce médecin de campagne et vous prenez toujours [ces médicaments.**

**H :** Vous savez ce que je] crains. Et je le dis avec tous mes respects, c'est que cela soit seulement un sujet de thèse, doctoral pour quelqu'un, sans avoir comme but d'être efficace pour le malade.

**C : Par rapport à l'étude vous avez [ces craintes, ces craintes-là ?**

**H :** Oui.] Alors ça c'est.

**C : On va essayer d'utiliser, là, y a pas de, de doctorat, on va essayer d'utiliser au mieux ces données pour pouvoir essayer [d'améliorer la prise en charge.**

**H :** Parce que je me pose la question comment,] comment est-ce que vous, vous, ou la personne qui, qui fait, dirige cette étude, comment pense-t-il aider au patient ? Ça, ça je me pose la question !

**C : Ce sera à voir selon les résultats, qu'est-ce qu'on peut en faire.**

**H :** Mais oui. Parce que dire que je ne peux pas faire 100 mètres d'une seule fois, et que je ne peux, je me fatigue quand je monte les escaliers, ou que je suis plutôt mieux ou pire que il y a trois mois, ça rime à quoi ?

**C : Donc pour vous [de voir.**

**H :** Mais quelles sont] ces, qu'est-ce que va faire un médecin devant ces éléments-là ? Qu'est-ce qu'il va faire ? Parce que il n'y a pas de médicament miracle, même pas en laboratoire, pour venir en aide demain ! Alors, où est l'utilité ? L'utilité, pour le patient, pas pour, pour celui qui fait le, l'étude ou l'observation des malades. Parce que c'est ce côté-là qui m'intéresse pas.

**C : Donc vous vous questionnez sur l'utilité et qu'est-ce qu'on pourra faire de ces résultats pour le patient.**

**H :** Voilà. Quelle est l'application qu'y aura après, comment ça va se répercuter pour le malade.

**C : Ça c'est les, les questions que vous avez. Par rapport à, à vos propres besoins, donc l'utilité que ça aurait pour vous d'avoir quelqu'un une fois par mois, ça aurait plus été pour ces gestes techniques dont vous me parliez avec l'aspirateur ou quelqu'un qui puisse vous, vous taper dans le dos. Et autrement en termes de besoins actuellement, ce serait plus une, une aide ménagère qui vous serait utile. Parce que tout ce qui est, comme vous me l'avez dit, tout ce qui est soutien psychologique ou spirituel etc., vous avez votre propre expérience de la mort et vous avez pas besoin ou envie de, de ce soutien particulier. Donc, donc voilà, vous avez aussi.**

**H :** Je crois savoir un tout petit peu plus que les autres, avec modestie, mais. Je l'ai dit, je ne suis pas en âge de prétendre autre chose. Je suis quelqu'un à la retraite, donc voilà, les aspirations c'était fini.

**C : Est-ce qu'y aurait d'autres choses encore que vous aimeriez rajouter par rapport à, à l'étude ou votre vécu par rapport à la maladie ?**

**H :** Ajouter ?

**C : Oui, est-ce qu'y aurait encore quelque chose que vous aimeriez me dire par rapport à ça ?**

**H :** … Oui, chez moi il y a une sorte de, de, ce n'est pas le mot « regrette », c'est, je sais pas. Je crains tout ce qui peut être que du domaine de, intellectuel. Et quand je dis cela, je ne pense que aux

médecins. Que, je regrette, que voilà, qu'il n'y ait pas une, un résultat qui puisse, que puisse bénéficier tout de suite et matériellement un malade. Que cela ne soit que un, un jeu intellectuel de la médecine, qui ne va pas très loin, du côté médical ne va pas très loin, parce que vous partez sans mettre en question que la maladie soit guérissable. Alors c'est déjà assez décevant. Faire des recherches sans espoir. Donc il y a le côté intellectuel, et le côté humain qui manque, à mon avis. Et pourtant je, je n'ai pas mis beaucoup de temps à réfléchir sur ces questionnaires. Mais voilà mon, ma réticence de principe.

**C : Par rapport à ce type d'étude-là.**

**H :** Par rapport à, à, aux questionnaires.

**C : Qui manquerait ce côté plus humain et, et qu'on reste à un niveau [trop intellectuel.**

**H :** Ou d'application, d'application] pratique, immédiate.

**C : Ce serait plus ça qui serait utile, d'avoir des applications immédiates qu'on puisse tirer de ce type d'étude.**

**H :** Si non immédiate, au moins à, à, à court terme ! Voilà une solution, un espoir, sinon, je n'attends que la facture du médecin. Voilà, c'était ma, mon vécu avec le spécialiste, que je savais qu'à la fin, il y aurait une grosse facture à la fin du mois ! Alors ça c'est pas intéressant *(rires)*.

**C : Bon comme c'est une maladie, comme vous disiez, incurable, vous souhaiteriez qu'on puisse plutôt trouver des solutions pratiques, que.**

**H :** Mais, le spécialiste il sait que ce type de malade, a besoin de l'aspirateur. Je ne sais pas s'ils ont assez d'expérience pour pouvoir dire, une fois par mois ou une fois tous les deux mois. Enfin, mais le besoin est là. Il faut aspirer le malade. Quand on verra, selon besoins, peut-être. Mais, mais qu'est-ce que fait le malade ? D'abord, il doit le savoir, qu'il y a cette solution. Ce n'est pas une aide radicale, ou définitive de guérison. Mais c'est une façon de aider à vivre. Et, alors il n'y a aucune indication de, d'une solution ! Celui de l'aspirateur ou, ou une autre, hein. Peut-être il y a d'autres solutions ou d'autres types d'aides ! Mais moi je ne les connais pas ! D'après mon expérience, je n'ai que une seule, et c'est l'aspirateur.

**C : Et vous souhaiteriez pouvoir savoir, avoir l'information [sur, sur ces aides.**

**H :** Oui, ou le faire mécaniquement] une fois par mois, voilà. Ou bien quand l'alerte sonne, vous pouvez aller à tel endroit, et on vous aspire ! Mais devoir demander un rendez-vous, c'est déjà comme aller chez le dentiste, dans six mois, ça c'est pas solution.

**C : De pouvoir avoir accès à, à cet aspirateur [régulièrement et.**

**H :** A l'intervention médicale,] oui !

**C : Quand vous en avez besoin.**

**H :** Voilà.

**C : Je vous.**

**H :** Comme on va à l'hôpital, aux urgences, voilà, pouvoir aller, quelqu'un qui a, qui a la machine adéquate.

**C : Pour pouvoir faire cette intervention. Je vous, je vous remercie beaucoup de, de votre témoignage et d'avoir pris le temps de me raconter votre vécu. Si vous avez pas encore autre chose à ajouter, je pense qu'on peut arrêter, arrêter là ?**

**H :** Ah oui, bien sûr, si c'est.

**C : Si on a fait le tour. Mais merci.**

# Participant n°18

Nom d'emprunt : Edouard
Âge : 78 ans
Sexe : M
Groupe : Contrôle

**C : Vous avez participé à une étude qui avait pour objectif de comparer le traitement habituel de votre maladie pulmonaire, la BPCO, avec une prise en charge précoce, soutenue et intégrée. Vous avez été dans le groupe bénéficiant des soins habituels. Pouvez-vous me dire comment ça s'est passé ?**

**E :** Ecoutez, ça s'est bien passé. Ouais, c'était assez léger comme. Ouais, moi j'ai jamais cru au, à la BPCO hein. Bon j'avais l'appareil, et tout ça, et tout ça, mais. Je sais pas *(soupir)*. Quand j'avais les gens de X *(hôpital)* qui venaient, l'infirmier, l'infirmière ou comme ça, je leur disais : « mais je comprends pas ». Puis ils prenaient des mesures. Ils étaient pas d'accord avec, disons, le médecin qui était le *Docteur D*. Ils disaient : « oui effectivement ». Après j'allais chez le médecin, les infirmiers ils me disent que : « oui mais ils ont des appareils, ils sont pas étalonnés, ils sont, ils sont pas juste ». Voilà, bon, voilà. J'ai toujours eu un doute disons sur le, ouais, comme maintenant je fais absolument plus rien du tout. J'ai changé de pneumologue, je prends un qui est ici, donc *Docteur E*. Il m'a dit, bon la dernière fois que je l'ai vu, il m'a dit : « écoutez, on se revoit dans trois ans, hein ». J'ai dit : « ben oui si je suis encore là ». *(rires)*. Mais, non j'ai jamais… Oui, je toussais pas, je, oui j'étais essoufflé, tout simplement. C'est le seul truc. Suite à un infarctus, donc, le cardiologue pensait, il, il m'a fait faire un test d'efforts et tout ça. Puis, il a dit : « ça vient pas de ». Le test d'efforts était correct, enfin, j'étais peut-être pas champion du monde, mais enfin voilà quoi. Il m'a dit à ce moment-là : « faut aller voir un pneumologue ». Et puis c'est là que j'ai été chez le *Docteur D*, en disant, voilà. J'ai toujours pensé *(rires)* que le *Docteur D*, je sais pas si vous le connaissez ?

**C : Non.**

**E :** Je crois que c'est, c'était à l'époque le président de X *(ligue pulmonaire)*, puis je me dis toujours ils ont un certain nombre d'appareils à placer, faut faire tourner le business, hein, à monsieur *(nom)* on en refile un. C'était quand même 600 et quelques francs tous les, tous les trois mois, ou je me rappelle plus, disons. Bon, mais, voilà, j'ai jamais.

**C : Vous avez jamais vraiment cru à la BPCO ?**

**E :** Non, non, non, non. Et puis ça me prenait beaucoup de temps parce qu'il fallait, bon, j'ai négocié avec le toubib, il voulait que, il voulait que je fasse je crois 15 heures par jour, j'avais dit : « c'est pas possible. Hein, à 15 heures par jour, moi dans six mois je suis un légume, hein ! ». Assis dans, parce que vous allez nulle part avec votre appareil, hein. Parce que vous avez des trucs dans le nez, ben vous restez assis dans un fauteuil. Je vais pas rester 15 heures par jour assis, hein. Alors j'avais dit : « écoutez je suis d'accord éventuellement la nuit hein, quand je vais au lit je mets l'appareil, voilà je dors sept heures par jour, ça suffira, quoi comme ça ». Puis c'est comme ça que j'ai fait, hein. Mais au début, effectivement, je passais mon temps dans un fauteuil avec, alors c'est pas, c'est pas terrible, terrible. C'est vraiment, vous avez une, une vie qui est, qui est nulle, hein. A l'époque ma femme était encore vivante donc, moi comme, ouais j'étais assis dans, au salon dans le fauteuil à, voilà comme ça, à regarder la télévision, quand même faut pas, au bout d'un certain temps, j'ai dit : « c'est exclu ».

**C : Vous avez [arrêté.**

**E :** Puis j'ai été,] oui, j'ai été voir le médecin. Puis je lui ai dit : « écoutez si, moi je continue pas, 15 heures par jour, il faut, et j'y crois pas, hein c'est » *(rires)*. Surtout que je souffrais pas vraiment, quoi, j'avais pas, je toussais pas, je crachais pas, voilà. Tout simplement j'avais, j'ai, j'ai fait un infarctus, bon c'était le deuxième mais, ouais, j'avais pas de, puis bon, c'est le cardiologue qui a dit : « ah c'est bizarre y a ». Ils ont cherché, disons au niveau du coeur et tout ça. Ils ont rien trouvé. Ils ont dit : « faut aller voir un pneumologue, quoi ». Ça s'est passé comme ça. Mais j'ai eu plusieurs infirmiers, plusieurs infirmières qui sont venues. A toutes j'ai récité la même chose, ils prenaient des mesures et tout ça. Ils prenaient, ils notaient disons les.

**C : Celles de l'étude, qui venaient ?**

**E :** Oui, oui, oui, oui, oui. Oui, parce qu'ils, je sais plus, ils venaient une fois par mois, ou je sais pas exactement. J'ai eu je crois deux ou trois infirmières, un infirmier qui est venu. Voilà quoi. Moi personnellement, je me dis, y a eu, comment… Ouais, pour moi, je sais pas comment dire, ils ont amplifié la chose, disons je sais pas. C'était pour une question de, justement placer un appareil ou un truc comme ça, après tout pourquoi pas, lui. Certainement il devait y avoir un petit problème, disons. Mais peut-être pas au point d'avoir un appareil qui arrête pas de ronfler, faut tirer des câbles à travers la maison pour faire, faire passer les tuyaux. Du reste voyez *(il montre les trous dans les murs)*, je l'ai encore ici, quand j'ai, quand j'ai déménagé. J'habitais à X *(lieu)*, donc quand je suis venu ici, j'avais encore l'appareil. Alors bon ben, j'ai fait aller jusqu'au lit et tout ça, j'ai gardé les tuyaux, je veux dire on sait jamais si des fois il fallait le reprendre. Puis j'ai été chez un autre pneumologue ici, il m'a fait passer tous les tests, tous les examens, et j'ai expliqué la même chose qu'à vous, que j'y croyais pas. Pour moi, le *Docteur D* il, c'est un bon businessman disons, il a placé, il avait des appareils à placer, puis voilà il les a faits, quoi, voilà. Est-ce que je me trompe, c'est possible, peut-être, je sais pas *(rires)*.

**C : Mais pour vous les, les infirmières, elles ont un petit peu amplifié ?**

**E :** Ouais, voilà, voilà, ils en ont fait un petit peu trop disons, à mon avis, hein, à mon avis. Moi je suis pas médecin, mais c'est toujours le, disons l'impression que j'avais, quoi.

**C : Et y avait des discordes, c'est ça, entre les infirmières et le médecin, par rapport aux résultats, vous disiez des tests.**

**E :** Ben écoutez, je sais pas s'ils ont jamais contacté le *Docteur D* ou pas, mais, j'avais dit au *Docteur D*, je lui avais dit : « écoutez voilà ». Je me rappelle plus du nom de, des personnes qui venaient, mais enfin : « ils me disent que les, que les mesures qu'ils font eux, disons, nécessiteraient en somme, ouais, disons le, comment le concentrateur là est en quelque sorte inutile quoi ». Et j'arrivais très bien à tourner sans le, voilà. Puis lui il me disait toujours : « ouais, mais ils ont des appareils, et eux, c'est pas étalonné et ils les transportent avec eux, ils mesurent, ils mesurent pas juste, c'est mes instruments qui fonctionnent comme il faut, et tout ça ». Voilà, je sais pas, je sais pas, je sais pas. Bon, j'ai jamais eu beaucoup d'atomes crochus avec le *Docteur D*, disons, contrairement au pneumologue que j'ai maintenant, qui est vraiment un type, bon c'est, il est jeune déjà, puis vraiment il est, ouais, ouais il est, il est plus performant que mon, que ma, que ma, ma, ouais, j'ai une doctoresse qui est, généraliste, disons, qui elle sait strictement rien du tout. Elle tape sur l'écran comme ça, puis après elle me dit : « faut aller voir celui-ci, faut aller voir celui-là »*(rires).* Mais, alors que le pneumologue, lui non, il, il se donne quand même la peine d'écouter, disons il, ouais. Je me trouve plus chez un généraliste avec lui que *(rires)*, qu'avec la doctoresse que j'ai, quoi. D'autant plus que moi j'ai pas de problèmes disons, je vais, je circule, je, je bouge. Ouais, c'est, ouais j'arrête pas de bouger. On me dit que je suis hyperactif peut-être *(rires)*. Mais, ouais, ouais c'est.

**C : Mais vous avez pas de problèmes pour bouger, pour, pas de symptômes, vous disiez ?**

**E :** Rares sont les gens qui me dépassent dans la rue. Y en a quelques-uns, des très grands, des très jeunes qui marchent plus vite que moi *(rires)*. Mais sinon j'ai, xxx, ouais je suis actif, quoi, y a pas, je reste pas assis. Bon, je fais un peu d'informatique et tout ça, ça me fait aussi du bien ça, ça me change un peu les idées, surtout les jours de pluie, mais, s'il fait beau, je vais sortir, j'ai une amie qui a un jardin, je vais chez elle, je fais le jardin, je, ouais, ouais, non, c'est.

**C : Vous êtes actif.**

**E :** Ouais, ouais, je suis actif. Puis je suis bien, je mange, je dors, franchement ouais bon.

**C : Vous disiez que vous avez pas de toux, pas ?**

**E :** Non. Je tousse pas, je crache pas *(rires)*. C'est, [xxx.

**C : Et là vous aviez] l'oxygène que la nuit maintenant.**

**E :** Que la nuit oui, oui. Mais bon maintenant ça fait, ça fait deux ans que j'ai arrêté le, l'oxygène, j'ai plus de, j'ai rendu tous les appareils. Puis la dernière fois que j'ai été chez le, chez le pneumologue, il m'a dit : « bon, écoutez monsieur *(nom)*, ben ». Du reste j'ai marqué, je dois le rappeler en X *(mois)* 2018, hein. Donc je reporte ça d'une année sur l'autre *(rires)*, pour pas l'oublier parce que, non, non, franchement, franchement je vais bien.

**C : Ça fait deux ans que vous avez arrêté totalement l'oxygène, même la nuit ?**

**E :** Complètement. J'ai pas de médicaments pour le, pour les, j'ai des médicaments disons, mais pour le, comment, y a de l'aspirine cardio, puis bien entendu j'ai du cholestérol, j'ai de l'hypertension, je prends mes médicaments. Avec ces médicaments-là, y a aucun, dernièrement j'ai été faire le check-up, une fois par année, en même temps je fais le vaccin pour la grippe, disons. Toutes les mesures sont bonnes, voilà. C'est pas naturel puisque je prends des médicaments, disons. Mais je les prends régulièrement, puis voilà, puis ça va très bien, ouais. Je suis content, je suis heureux, je suis bien dans mes bottes *(rires)*.

**C : [Ça va bien, ouais *(rires)*.**

**E :** Ouais, ouais, ouais, ouais, ouais, ouais.]

**C : Puis vous avez pas de médicaments spécifiques pour la BPCO ?**

**E :** Non absolument rien, rien. Non c'est, je sais pas… C'est *(rires)*. Voilà. Alors bon, quand vous m'avez téléphoné, je me suis dit : « ben bon pourquoi pas, toute façon j'ai rien à faire hein ». Voyez c'est, disons que j'ai quand même du temps à moi en principe, je peux très bien me, puis ça me change un peu les idées, je rencontre d'autres personnes, voilà. Mais, c'est pas. Voilà, c'est un peu mon histoire *(rires)*.

**C : Oui. Et, qu'est-ce que vous attendiez en participant à cette étude ?**

**E :** Ah rien du tout, rien du tout ! Je l'ai fait parce que on m'a posé la question si je voulais. Ben j'ai dit : « oui ben, pourquoi pas, mon dieu ». Mais, ouais, quand les, quand les, les, les infirmières venaient, donc quand j'étais dans, ouais, j'étais d'abord suivi donc par des infirmiers, mais après y avait donc des dames qui venaient ou un monsieur, non c'est trois dames qui sont venues. Enfin bon je remplissais un questionnaire mais, avec des, ouais, je me suis jamais plaint de rien, jamais, franchement ce serait pas justifié disons que, je souffre de rien. Je vais, je viens, non, non, c'est pas, c'est pas… Donc moi quand j'ai fait le truc c'était pour faire plaisir, voilà, si on veut bien. Après tout pourquoi pas, mon dieu, si ça peut rendre service à la science, chic alors, quoi *(rires)*. Et puis voilà, mais moi personnellement, j'en ai jamais rien retiré. On m'a jamais rien apporté. Ouais, non, franchement c'est, on m'a pas dit : « faudrait faire ci, faudrait faire ça ». Non, non, non, non, c'est, voilà.

**C : C'était pour faire plaisir que vous avez participé.**

**E :** Ouais pour faire plaisir, oui tout à fait *(rires)*. C'est, c'était le, alors voilà.

**C : Et, et quelle a été votre réaction en apprenant que vous faisiez partie du groupe qui avait pas d'intervention ? Quand y a eu ce tirage au sort.**

**E :** Ça m'a fait ni chaud, ni froid *(rires)*. Ouais je m'en fichais. Je sais qu'on m'avait dit, ouais, que j'étais dans le groupe normal, en somme. Mais si j'avais été dans l'autre groupe, quelle aurait été la différence ?

**C : Y aurait eu une infirmière qui serait venue une fois par mois, pendant un an. Et à côté des questionnaires, l'autre groupe avait aussi les questionnaires, mais y avait des évaluations des besoins, en termes des symptômes physiques, des symptômes psychologiques, donc, plus un accompagnement global, [voilà.**

**E :** Ouais, d'accord.] Ouais c'est plus, mais, bon ben, non moi ça, je sais qu'ils m'ont dit : « ouais, il a

fait le tirage ». Ben bon ça m'était égal, que je sois dans un, je suis toujours le même hein, ça va rien changer à *(rires)*, à ma façon de, disons à ma, à ma santé, quoi. Tout simplement on me met dans un groupe, dans un autre, comme des fois on fait des, on donne des médicaments à certains, et puis à d'autres on leur refile des, des trucs qui, qui sont pas des vrais médicaments, qui sont des. Et puis voilà. Alors je me suis dit : « ben c'est un peu ce genre de trucs ». Mais pour moi non c'est *(rires)*.

**C : Ça vous a fait ni chaud ni froid [de, d'être dans un groupe, ou dans.**

**E :** Ah oui, ah oui, oui, oui, oui, oui, oui, oui,] j'ai… Ouais, non, j'avais, j'ai fait une autre étude, mais c'était autre chose. C'était pour, c'était avec ma femme là, parce qu'elle a fait des AVC donc, et puis y a eu, on a fait partie d'une, aussi d'une étude quoi, de nouveau, après tout pourquoi pas *(rires)*. On va, et puis ma fille, mon fils aussi ont dû aller, ont pris des, fait des prises de sang, pour voir si y avait, un, disons un, ouais c'était une étude pour voir quelle était, est-ce que c'est familial, est-ce que ça, enfin est-ce que c'est, je sais pas, est-ce que ça se transmet de père en fils, ou voilà, quoi. Mais bon, voilà, c'était une autre étude qui était un peu la même chose. On y a été, on l'a suivie, ça nous a rien apporté, je sais pas si la science a, a pu avancer pour ça. Si oui, tant mieux, sinon tant pis *(rires)*, c'est, ouais, ouais.

**C : Vous avez participé à d'autres études aussi.**

**E :** Oui, oui, ouais, ouais, c'est.

**C : Et si on, si on imaginait que vous aviez été dans le groupe qui bénéficiait de cette prise en charge globale spécialisée, quelle type d'aide ou informations par rapport à la BPCO, auriez-vous souhaité recevoir ?**

**E :** Ouais, disons que, non je vois pas, le problème c'est que… Je me sentais pas malade, si on veut bien. Je voyais pas bien qu'est-ce qu'on aurait pu m'apporter, enfin j'ai besoin de rien, je suis, hein. C'est, non, franchement, je m'attendais à rien suite à cette étude, disons. Parce que je me sentais pas la nécessité de, je trouvais le, disons le, le comment dire, le, ouais je trouvais qu'on en faisait trop ! Moi je me sentais bien et je savais pas pourquoi disons, je disais : « bon ben puisque ça leur fait plaisir, voilà ». Mais sinon non. Non, non, non c'est, ouais.

**C : Pas de besoins ?**

**E :** Non rien, rien, rien, rien. Je sais qu'au début le *Docteur D* m'avait donné des, des sprays, des trucs. J'ai dit : « mais vos machins-là, ça me brûle, ça, je suis plus mal fichu avec vos, vos médicaments, qu'avec rien du tout ! » Il a dit : « bon alors on va arrêter, puis on verra ». *(rires)*. Ouais, ouais c'est. J'avais de ces, ces trucs-là pour mettre dans la bouche, et puis il faut. Et après ça me brûlait, c'est oh, c'est, non, non, c'est. Pour moi… je retournerais d'abord pas chez lui *(rires)*. Et puis, j'ai jamais été d'accord, j'ai jamais compris pourquoi, pourquoi j'avais un inhalateur, parce que je dormais, je, je me suis jamais plaint de rien disons. Tout simplement, j'ai été, parce que le cardiologue m'a dit : « il faut aller là-bas ». Après l'autre m'a dit : « maintenant il faut prendre ça ». J'ai fait un petit peu, puis après j'ai dit : « oh ça va ! Je vais tomber malade avec votre truc, hein ! Si vous voulez la nuit quand je dors ça me gêne pas mais, pas dans le, la journée c'est exclu ! Je le mets pas hein, c'est, moi je veux pouvoir bouger ! » *(rires)*. Puis je regrette pas disons, peut-être que je me trompe hein, mais bon de toute façon, on y verra, quoi. C'est, je sais pas du tout si… Je crois ce qui m'a fait le plus de bien, c'est en somme d'arrêter de fumer, voilà. C'est peut-être, au deuxième infarctus, j'ai arrêté de fumer du jour au lendemain, j'ai plus retouché une cigarette, voilà. C'est peut-être ce qui m'a fait le plus de bien, ce qui me permet peut-être d'être, d'être bien maintenant. Est-ce que je referais un autre infarctus j'en sais rien, quelles en seront les causes, j'en sais rien du tout, c'est, alors bon. On est une famille assez solide disons, où les gens vont assez loin, et. Donc je me dis : « ben après tout peut-être je me xxx *(rires)* de ce truc-là ». Parce que quand je me compare aux autres personnes qui sont ici, j'ai pas non plus tellement ma place ici, disons, parce que je suis trop, ouais, je suis en somme trop bien pour être là quoi, mais enfin bon. Ma femme étant décédée, j'avais un grand appartement, qu'est-ce que je voulais en faire. Puis mon fils il habite là, et il a une copine qui est infirmière ici, puis il me dit : « tu sais, y a un appartement qui se libère, si elle te dit d'aller voir madame *(nom)* ». Donc c'est la gérante sociale ici. Et puis j'ai été la voir, et puis elle m'a dit : « écoutez, ouais effectivement on a ». Puis elle m'a donné les coordonnées pour contacter le, la

fondation et tout ça. J'ai, ça s'est fait, en quinze jours, j'avais l'appart, hein. Ils m'ont pas fait d'histoires, ils m'ont pas fait, bon. Puis je suis bien là, qu'est-ce que je veux de plus, hein *(rires)*. C'est vrai, c'est. J'aurais peut-être une pièce de plus, ce serait mieux ! Mais il faut être en couple, y en a de l'autre côté, mais il faut être en couple, alors bon ben, ça va pas jouer, quoi, c'est. Parce que ça c'est toujours embêtant, les gens, si quelqu'un vient, c'est la chambre, ça fait chambre à coucher et puis salon en même temps. Je préférerais avoir une chambre à coucher disons, même pas grande disons, mais à part, puis pouvoir recevoir dans, dans le salon normalement. C'est pour ça qu'on est à la cuisine, maintenant *(rires)*.

**C : Mais vous vous sentez bien ici.**

**E :** Oui, oui oui, oui, oui !

**C : Vous vous sentez peut-être pas trop à votre place dans, dans l'immeuble ?**

**E :** Ouais, ça me gêne pas après tout, je me dis bon ben, il vaut mieux rentrer ici en bonne santé, quitte à ce que la santé se dégrade, à ce moment-là je bénéficierais disons de, d'une aide pour être maintenu le plus longtemps possible à domicile. Parce que, si je viens ici au moment où je suis malade, ils vont pas, il vont me dire : « c'est pas tellement votre place ». Si vous tombez malade en cours de route, on peut éventuellement vous. Voilà c'est. Mais pour le moment ça va bien, voilà c'est, oui, oui.

**C : Vous disiez vous avez votre fils qui, qui habite pas loin d'ici ?**

**E :** Ouais, il habite là juste ici. Moi j'ai habité pendant 18 ans au X (numéro). Voilà, puis après je suis parti, on a été habité à X *(ville)*, X *(village)*, enfin on a. J'étais X *(métier)*, donc on rachetait des établissements, on déménageait chaque fois avec, quoi c'est *(rires)*. Voilà, quoi, c'est, non, non c'est. Et puis ma fille aussi, elle est pas très loin, mais elle est aussi sur la X *(commune)*, donc.

**C : Et quel type de soutien vous avez de la part de vos proches ?**

**E :** Oh très peu ! Très peu ! Très peu. Bon j'ai une famille qui est, qui est très restreinte, disons. Il me reste plus que deux cousines, voilà, c'est pas beaucoup *(rires)*. Et puis aussi elles sont nonagénaires, ou, voyez, quoi, c'est *(rires)*. Alors, et puis bon ben mon fils il est célibataire, il a X *(âge dans la cinquantaine)*, et, il est vraiment indépendant, il est. Mais bon, des fois je le vois ici, il regarde, puis je lui fais comme ça, pouf il monte. Puis il vient discuter un moment. Ou bien, j'ai un ordinateur, lui aussi il a. Mais il a pas d'imprimante, alors il vient des fois ici, pour imprimer des trucs, des machins comme ça. Puis ma fille, ben bon, elle, elle est à, elle est vers X *(parc)*, ici. Bon elle a la, elle a deux gamins, disons, bon y en a un gamin, y en a qui a X *(âge)*, disons l'autre qui est plus petit, il a X *(âge)*. Alors bon elle s'occupe de ses gamins, elle. Mais bon elle a sa vie à elle, hein, c'est. Surtout qu'elle est pas là, elle est là depuis, ah, environ trois mois. Parce qu'avant elle vivait en X *(pays)*. Elle, et puis après elle s'est séparée avec son mari, donc il fallait qu'elle suive les, il fallait qu'elle s'occupe des, des enfants. Eux, voulaient pas faire l'école en X *(pays)*, ils voulaient faire l'école en Suisse. Donc, elle, elle était obligée, parce qu'ils étaient avec le papa, les enfants. Et puis après le papa, bon il s'est trouvé une, une copine, il s'est marié, il a fait d'autres enfants. Alors ben elle a dit : « bon ben ma foi je reviens en Suisse ». Mais elle attend pour repartir que le petit dernier il termine, ait un métier, un apprenti'. Mais elle dit : « bon, il a X *(âge)*, ça va aller en tout cas jusqu'à 18 ans, avant que je puisse repartir, quoi ». Parce qu'elle c'est, c'est X *(pays)*, c'est, le soleil, la mer *(rires)*. Oui, du reste ben elle est, elle est là *(rires)*.

**C : *(rires)* Vous disiez pas, pas beaucoup de soutien de la part des proches.**

**E :** [Non, non, ah non.

**C : Mais] de quoi vous auriez besoin, en terme de, de soutien ?**

**E :** Ah moi rien, ah rien, j'ai vraiment, ouais, bon maintenant j'ai une compagne, on vit pas ensemble parce que elle est, bon elle est un peu plus jeune que moi, elle a X *(âge)*, mais c'est, mais bon c'est plus pareil, c'est plus comme quand on a 30 ans, hein, c'est pas. Chacun a besoin de son espace de

liberté, de, puis on peut pas, on a un vécu qui est quand même trop, trop important disons, alors des fois elle me dit : « mais écoute, j'en ai marre, moi on se voit pas pendant deux, trois jours, parce que *(rires)* je vais aller me promener ». Elle aime beaucoup la, elle aime marcher dans la nature, et tout ça, c'est. Non, non, non, non, de ce côté-là ouais, franchement, je sais pas, je demande, ouais je demande rien du tout, parce que, ouais je sens pas le, la nécessité de. Au début je m'étais dit : « tiens, je vais peut-être faire du bénévolat, ou des trucs comme ça, puis ». Puis j'ai vu qu'après tout je m'embêtais pas du tout même sans activité bien définie, disons. Et puis, voilà, et puis j'ai laissé tombé. Puis après bon ben j'ai rencontré cette amie, où le bénévolat ça, c'était plus tellement disons d'actualité quoi, c'était pour occuper mon temps. Alors bon, toute façon, elle, elle vit à la campagne, elle a un jardin, donc je passe ma vie à faire des trous, à *(rires)*. Comme ça c'est, non, non, non, c'est, non, non, je suis content, y a pas de, je demande rien à personne *(rires)*. Ouais, c'est, ouais.

**C : Pas de, pas de besoins particuliers.**

**E :** Tout au plus, plus d'argent oui *(rires)*. Si vous avez une option, y a pas de problèmes. Je vous donne mon numéro de compte. Voilà, je.

**C : *(rires)* Plus le soutien financier.**

**E :** Pardon ?

**C : Plus un soutien [financier.**

**E :** Financier,] oui, oui, oui, oui, oui, oui. Mais même, c'est, un soutien financier, mais même pas pour, pour vivre, disons, plutôt pour, pour les extras, ouais. Vivre, y a pas de problèmes. Je me débrouille avec ce que j'ai, et puis, puis voilà, quoi. Mais, je sais pas, si je veux partir un mois en vacances ou, bon, je pourrais partir un mois par année, peut-être mais, comme j'ai rien à faire, pourquoi est-ce que je partirais pas, je sais pas, tous les trois mois, partir un mois *(rires)*, ce serait pas plus mal, mais bon financièrement je peux pas, quoi, c'est, c'est ça le. Mais du point de vue physique, et tout ça, non rien. Point de vue psychologique non plus, ça, ça marche quoi, c'est. Non, non, c'est. Non je vous dis, je vous ai dit de venir, c'était pas une obligation, disons. Mais j'ai dit : « bon ben pourquoi pas, mon dieu, si ». Et puis après on a eu un peu de peine à se, à se trouver une date du fait que, parce qu'avant j'avais le chien qui était là, je le prenais chez moi, disons et tout ça. Et puis après, le prob' c'est que c'était un gros chien, il fait 40 kilos, je peux pas le prendre sous le bras, il arrivait plus à monter dans le bus, parce que j'ai bazardé la voiture, donc je circule avec les, les transports publics. Au début y avait pas de problèmes, il sautait dans le bus, tac, tac, il était gentil, c'était un golden, donc c'est des bonnes bêtes, il, voilà. Mais après ben bon, voilà, je pouvais pas moi le garder ici, c'était pas possible. Donc il a été chez ma compagne disons, mais c'était pas son chien *(rires)*. Et puis c'est elle qui l'a gardé chez elle, elle avait le jardin, elle avait tout ce qu'il fallait pour, pour le chien, ça allait tip top, quoi. Mais ça me prenait tout mon temps, parce que, bon elle a aussi, elle a aussi une petite-fille, elle a des, enfin bref elle a aussi des obligations, résultat on se relayait, hein. Je disais : « ah bon, tu viens, ouais, tu peux venir demain matin ? » ; « ouais, pas de problèmes ». Et le lendemain matin, voilà, quoi, on se, mais on le gérait disons, xxx, donc et à la campagne. Et puis, et puis voilà. Puis bon, pour finir, l'arthrose, la dysplasie, du, il arrivait plus en avant, il pouvait plus se tenir debout, puis dimanche, ça allait plus du tout, j'ai téléphoné au véto, et puis il est venu à domicile, il l'a endormi, quoi c'est, voilà. Alors bon, c'était pas mon chien disons, mais je l'aimais bien, c'est. Mais, pour mon amie, c'est beaucoup plus difficile, parce que, d'abord elle est plus sensible que moi, disons. Et puis elle l'a eu pendant 12 ans, donc, elle s'est toujours occupé de lui, quoi. Parce que son maître, il a un métier à la gomme, il est X *(métier)* à X *(lieu)*. Il a des horaires le jour, la nuit, le samedi, le dimanche. Alors.

**C : Donc c'était plus difficile pour votre amie.**

**E :** Ouais, ouais, oui, oui, ouais, ouais, c'est sûr, [c'est sûr. Alors voilà.

**C : Que, que pour vous. Et, et si on regarde,] Je sais que dans, dans le cours des soins, on peut aussi parler des directives anticipées, je sais pas si vous [avez déjà entendu parler de, de ça.**

**E :** Non, non, non, jamais.]

**C : C'est, le type de soins qu'on souhaiterait en fin de vie, au cas où on se trouve un jour dans une situation où on peut plus décider, [ce qu'on aimerait comme soins.**

**E :** Ouais, ouais, d'accord. Ah oui, d'accord. Oui, oui, oui, oui.

**C : Je sais pas si vous avez déjà pu discuter de ça avec un professionnel ?**

**E :** Non, non. Je l'ai vécu par deux fois disons comme intervenant disons, une fois pour mon père. Et puis une autre fois pour ma femme. Mon père, donc lui il était aux, il était aux X *(hôpital)*. Bon il avait la maladie de parkinson et puis pour finir il a fallu le mettre, disons, à l'hôpital, là-bas. Mais bon, il en avait marre, il a, alors il arrachait les tuyaux, les trucs comme ça, alors pour finir ils l'attachaient dans le, ouais il, il était attaché dans son lit, pour qu'il puisse pas arracher les tuyaux. Pour finir, j'ai été trouvé le médecin, puis je lui ai dit : « écoutez, hein, il va pas guérir, hein. Donc, en plus de ça il, il veut, ouais, il en a marre ». Il voulait partir, hein. Puis il m'a dit : « bon ben ». Ils peuvent rien faire disons. Mais il m'a dit : « bon ». Parce que des fois ils devaient être actif dans les, dans les interventions. Ils ont dit qu'ils allaient tout simplement lever le pied, quoi, disons, pas se montrer trop actif pour le, voilà. Puis trois jours après il me téléphonait pour me dire : « écoutez votre père est décédé, quoi ». Voilà. Si j'avais, si j'étais pas intervenu, ben je sais pas combien de temps il aurait pu encore vivre ou comme ça, quoi. Quant à femme eh ben bon, c'était un peu du pareil au même. Elle a fait deux AVC. Le deuxième s'est mal passé, disons. L'intervention qu'ils ont fait à l'hôpital, ça, ça a blessé encore les, les vaisseaux de, du cerveau. Mais bon, toute façon, elle est, elle a vécu trois mois en regardant le plafond, elle savait plus le français, le. Elle pouvait pas parler, elle reconnaissait pas les gens. Quand j'allais la voir, en tout cas les premières fois, quand j'arrivais pour lui faire une bise comme ça, elle se reculait, comme pour dire : « il est gonflé celui-là, non mais je le connais pas ». Les enfants non plus, elle avait, les petits-enfants non plus, elle avait pas l'air de les, elle était complètement, puis elle était 100% dépendante. Elle pouvait pas manger, elle, voilà. Puis ils ont essayé de, au bout de, de trois mois, ils ont essayé de, parce qu'elle avait une sonde, ils voulaient après faire, je sais pas ils font, ils font un trou là, et puis ils mettent une sonde directement pour la nourrir quoi, parce que c'était pas possible, toujours dans le nez, et tout ça, mais. Puis ils ont fait un test pour la narcose, disons, puis il a mal tourné le test, hein c'est. Ils ont bien cru qu'ils la perdaient. Puis après ben bon, ils ont dit : « on peut rien faire, il faut continuer comme ça, quoi ». Elle faisait, ouais, elle était dans son lit, elle regardait le plafond, elle disait rien, elle criait, ouais elle gênait les autres malades. Du reste tout son temps, elle a toujours été en chambre privée, alors qu'elle a pas du tout d'assurance pour être en privé, mais, ça créait un tel, un tel désordre dans le, qu'ils étaient obligés de l'isoler, puis elle a toujours eu une chambre, elle était aussi aux, elle était d'abord à l'hôpital, elle était aux X *(hôpital)*. Et puis ensuite, comme j'habitais à X *(lieu)*, je leur ai dit : « vous pourriez pas la transférer à, au X *(hôpital) »*. Et puis ils m'ont dit : « ouais ça peut jouer, parce que maintenant ». Avant c'était une fin de vie le X *(hôpital)*, alors que là, ils ont dit : « on a une unité de réadaptation ». Je sais pas trop quoi. Mais ils ont jamais rien pu faire. Puis au bout d'un certain temps ils m'ont dit : « faut qu'on la transfère, ouais faut qu'elle aille dans un, au département de fin de vie, quoi, disons ». Et puis là ils s'acharnaient un petit peu, j'ai pris les médecins, j'ai dit : « écoutez, on en est où ? On va où ? Vous savez aussi bien que moi où on va ». Et puis il m'a dit : « oui, bien sûr ». Alors il m'a dit : « ouais, en somme, ce que vous demandez c'est le, c'est le retrait thérapeutique ». Puis j'ai dit : « oui, puisque y a pas d'autre ». Puis il m'a dit : « bon ben voilà, on va le faire ». Elle a vécu dix jours, puis. Alors, les dix derniers jours c'était impeccable. Mais alors morphine, morphine, morphine, y avait plus de, y avait plus de, avant y avait une espèce de feuille de route. Puis les infirmières devaient s'y, tandis que là c'est, si y en avait besoin on lui en donnait hein, c'est, voilà. Et puis l'encadrement en fin de vie est différent de, de ce qui se passe, disons en réhabilitation, où c'est quand même des gens qui sont censés revenir. Tandis que là bon, les infirmières sont tops, elles, non, non, elles, bien, bien, ouais. Voilà. Donc, mais pour moi, personnellement disons que j'aimerais pas être un légume, j'aimerais pas être dans un lit à regarder le plafond. Disons, ça m'intéresse plus de, de vivre, si je deviens dépendant, voilà. Plus aucun intérêt, qu'est-ce que je vais faire, hein. C'est, voilà. Y a, par exemple, y a, y a *Exit* hein, qui, là pendant qu'on est, on est en bonne santé on peut, disons, éventuellement souscrire un truc, ils peuvent intervenir et tout ça. Là je suis un peu moins, je suis un peu moins chaud, disons *(rires)*. Ouais. Parce que j'ai vécu le, ouais la fin de vie d'une dame qui, qui était une de mes clientes quand j'étais à X *(lieu)*. Et puis, un jour y a la serveuse qui me dit : « ah écoutez, voilà ». Et je vais apporter ce dernier repas pour madame X, quoi. A 15 heures, y a *Exit* pour. J'ai dit : « bon, eh ben voilà, on perd une cliente, mais ». Il faut avoir un certain, ça doit pas être évident, quand vous le savez que à 15 heures, bon. Bien sûr c'est des gens, xxx, ils sont, ils veulent <u>partir</u>, hein ! Ils demandent que ça, disons, mais enfin quand même ça doit être, ça doit être très, très

spécial, comme, ouais, ouais. Ça m'avait, ça m'avait choqué un petit peu, disons, ouais *(rires)*.

**C : Donc ça, *Exit* [ça vous parle moins.**

**E :** Ouais, ouais, ouais, ouais, ouais, ouais.] Ouais, on lui a apporté tous les jours donc, tous les jours à midi on lui a apporté le plat du jour, disons, ou bien quelque chose qu'elle avait commandé, ou un truc comme ça, puis la serveuse m'a dit, elle m'a dit : « eh ben c'est le dernier aujourd'hui. A 15 heures, elle part ». Bon, ça fait drôle *(rires)*.

**C : Ça fait bizarre.**

**E :** Ouais, ouais, ouais, ouais, tout à fait, tout à fait.

**C : Donc là vous aimeriez pas être, si c'était pour vous, vous aimeriez pas être [dépendant, mais vous souhaiteriez pas faire appel à quelque chose comme *Exit*.**

**E :** Voilà, non.] J'aimerais que disons, les proches qui sont autour aient le bon sens *(rires)*, de dire : « ouais bon, il en marre ». Ou bien à la limite y en, y en a qui demandent, y en a qui, ouais. Je connais une personne elle pouvait encore écrire un petit peu, elle a marqué : « laissez-moi partir ». Elle avait pas *Exit*, elle avait rien du tout, mais tout simplement, elle, voilà, c'était fini, elle en avait marre. 80 ans, malade, avec un cancer et tout ça. Puis en phase vraiment terminale, quoi, disons. Elle a écrit sur un papier : « laissez-moi partir ». Hein, voilà. C'est ça la vieillesse hein, y a un moment où *(rires)*. Mais pour le moment ça me pose pas de problèmes. Ouais, ouais. Mais je peux pas dire, peut-être, avec les, bon j'ai 78 ans maintenant, mais je sais pas, peut-être quand j'aurai, peut-être qu'à 80, 82, 84, j'en sais rien moi, je peux tomber malade, puis devoir rentrer en EMS. Alors là, y en a qui sont bien en EMS hein, ceux qui sont encore en bonne santé. Mais y en a aussi qui sont moins, qui, ouais, qui sont. Bon je vois, vers, vers chez ma, vers chez mon amie là, y a un EMS, y en a qui sont, vraiment ils sont contents, ils sont, ils sont tout heureux d'être là-bas. Puis y en a d'autres, c'est, c'est un peu plus difficile hein, c'est. Ceux qui peuvent pas sortir, qui sont. A voir, à voir.

**C : Mais pour l'instant [ça va plutôt bien.**

**E :** Pour le moment on me pose pas la question.] Moi je vis un peu, je regarde jamais en arrière, je regarde toujours en avant, puis je me fais pas, on va y aller au jour le jour, on verra bien comment ça se passe, je crois que *(rires)*, ouais, c'est.

**C : Et si, si je résume un peu par rapport à, à cette étude. Donc vous, vous m'avez dit que vous avez [participé pour faire plaisir.**

**E :** Oui, oui, oui, ouais, ouais.]

**C : Vous aviez pas d'attentes pour vous.**

**E :** Non, rien.

**C : Et, ça vous a fait ni chaud ni froid que vous soyez dans un groupe ou dans un autre.**

**E :** Ouais, ouais, non, non. Pour moi, aucune importance.

**C : Ouais, ouais. Vous vous sentiez pas, vous vous sentez pas malade.**

**E :** Pas du tout, non, non. Non, je me sens des fois poussif disons, mais je me dis : « ben bon faut quand même pas exagérer ». Y a certains trucs que je pouvais faire avant que je peux plus faire maintenant, disons, mais. Ouais, si l'ascenseur tombe en panne ici, puis que je reviens avec 20 kilos de commissions sur les bras, je vais souffrir pour remonter les six, faire les, les six étages à pied, hein, c'est. Ou bien s'il faut courir, non je m'essouffle, mais bon, je fais pas de, à part la marche, je ne fais pas de sport, voilà. Alors, je sais pas si je faisais partie d'une équipe de foot senior ou un truc comme ça, j'aurais peut-être de la peine, disons. Puis du fait que, non, tout simplement de la marche, je marche puis voilà. Je fais pas de varappe, je fais pas de vélo, je fais pas de natation, je fais pas de

ski. C'est tous des trucs que j'ai fait en étant jeune, disons. J'ai fait du sport comme tous les, comme tous, tous les jeunes, quoi. Mais comme maintenant, ben je fais plus rien, tout simplement, mon, mon pneumo là, il me dit : « écoutez monsieur *(nom)*, tous les jours faites une demi-heure de marche régulièrement, qu'il vente, qu'il pleuve, qu'il fasse chaud, qu'il fasse froid, vous y allez, vous faites une demi-heure de marche ». Voilà, puis, puis voilà, je, puis, pour moi c'est, c'est bien. J'apprécie d'aller marcher. Si, si je peux pas y aller, je me sens un petit peu, ouais je suis un peu rogne, je dis : « oh zut, hein, pourquoi est-ce que je peux pas y aller ». Parce que des fois, si, par exemple, si, s'il fait vraiment pas beau ou comme ça, je dis : « bon, je xxx de. S'il tombe des seilles d'eau, je vais pas aller marcher une demi-heure, quoi ». Je laisse tomber, mais après je suis pas content à la fin de la journée. Je dis : « ouais d'accord, j'ai pas été » *(rires)*.

**C : Ça vous fait du bien [d'aller, d'aller marcher.**

**E :** Ah oui, ah oui, oui, ah oui, oui, oui,] ouais, ouais, ouais, ouais.

**C : Vous me disiez vous êtes très actif et vous avez pas de symptômes comme [la toux ou d'autres symptômes de, de la BPCO.**

**E :** Non, non, non, non, non.]

**C : Donc en termes de traitements, vous avez arrêté aussi tout ce qui était.**

**E :** J'ai rien de rien en termes de traitements. Ben, une fois, une fois par année, chez le pneumologue. Maintenant la dernière fois qu'il m'a vu, il m'a dit : « écoutez, revenez dans trois ans » *(rires)*. Et puis, puis voilà, quoi. Mais franchement, par contre, si, si j'avais un problème, ou un truc comme ça, j'hésiterais pas à le contacter. C'est bien ce qu'il m'a dit, il m'a dit : « écoutez, on y va comme ça pour trois ans, s'il y a un problème, vous me téléphonez, vous venez me voir, on regarde le, on regarde le problème, et puis voilà, quoi ». Mais, pour le moment, y a rien, rien, rien, rien, rien.

**C : Vous pouvez contacter si jamais ce pneumologue avec qui vous avez un [bon contact.**

**E :** Oui, oui,] oui. Oui, il est là, il est, c'est le *Docteur E*, il fait partie du, de la, de la permanence, mais il est pas ici, il est à l*a* X *(lieu)*, non pas à X *(lieu)* sur la X *(adresse)*, juste vers l'église. C'est la première permanence, puis après ils ont agrandi, ils se sont mis là, mais lui il est là-bas. Puis avec lui alors j'ai vraiment une entente tip top, c'est, ouais. Je regrette qu'il soit que pneumologue *(rires)*. Alors ouais, c'est.

**C : Donc là vous avez pas de, de besoins particuliers.**

**E :** Rien, rien.

**C : A part peut-être un peu plus [de sous pour pouvoir peut-être faire des voyages, mais.**

**E :** Oui, oui, bon, voilà, voilà, c'est ça.]

**C : Mais par rapport en tout cas à [votre maladie pulmonaire, vous avez pas de besoins spécifiques.**

**E :** Voilà, non, non, rien, rien du tout, rien du tout.] Non, non, c'est plus une question de, de moyens. Parce que c'est toujours le problème quand on est indépendant, on n'a pas de caisse de retraite, on n'a rien du tout. Puis les affaires sont quand même pas faciles, donc quand on a un peu d'argent, ben on l'investit, voilà, on se retrouve à la retraite, on a, on a l'AVS, éventuellement une aide, l'aide complémentaire, disons, mais ça va pas chercher bien loin, disons. Mais mis l'un dans l'autre avec ça y a pas de problèmes, je, je fais, je tourne, je vis, là, voilà. C'est juste une question de dire ouais, ouais. Des fois je dis à ma copine : « ouais j'aimerais bien si on pouvait partir plus souvent ou bien ». Tandis que là faut faire attention, quoi. Il faut gérer son argent, il faut éviter de tout claquer, et puis *(rires)*. C'est.

**C : Mais autrement pas d'autres, [d'autres besoins.**

**E :** Non,], ah non, non, non, non.

**C : Je sais pas si y avait autre chose que, que vous souhaiteriez encore ajouter par rapport à, à l'étude ou [par rapport à.**
**E :** Non, non franchement] pas, je vois pas disons, aussi bien aujourd'hui que, que précédemment… Au départ, quand on, quand le, quand le cardiologue m'a envoyé chez le pneumologue, j'ai dit : « bon, bon je suis peut-être malade, quoi ». Et puis pourquoi pas. Le pneumologue : « oui, oui, vous êtes, vous êtes malade, vous avez ». Je veux bien. Alors il m'a donné des médicaments, le concentrateur, tout le bidule, et tout ça. Puis avec le temps, j'ai dit : « mais ça va pas mieux avec tout ça, y a rien qui change » *(rires)*. J'étais pas. Et puis, puis voilà quoi, c'est.

**C : Ça changeait rien pour vous [d'avoir.**

**E :** Rien,] rien, rien, rien du tout. Je me suis, à aucun moment je me suis, je me suis senti, même quand j'ai fait mes, mes deux infarctus là, ils vous proposent éventuellement d'aller faire, à X *(hôpital de réhabilitation)*, on peut aller faire la piscine, je sais pas quoi, des trucs comme ça. J'ai jamais rien fait du tout. Bon j'étais encore actif à l'époque donc, moi je suis rentré, je sais pas, disons, vous êtes transporté à l'hôpital en ambulance, ils vous font le, ils m'ont posé des stents, donc. A la fin de la semaine vous sortez de l'hôpital, puis je retourne au boulot. J'ai jamais fait un jour de congé. Tant que j'étais à l'hôpital je pouvais pas travailler, disons. Mais dès que j'arrivais à la maison, je retournais à mon X *(lieu de travail)*, je retournais à ma X *(lieu de travail)*, puis voilà quoi. Jamais, jamais fait *(rires)*, je me suis jamais arrêté. Je crois c'est les deux seules fois dans ma vie où je suis tombé malade si on veut bien. Deux fois une semaine j'ai pas pu travailler, mais tout le reste du temps, d'abord à l'époque le chômage ça existait pas *(rires)*. Et puis bon, du fait que j'étais indépendant, c'est pas trop, mais j'ai jamais arrêté de bosser, voilà, c'est.

**C : Vous avez toujours [continué, à part ces deux fois.**

**E :** Ouais, ouais, ouais, ouais, ouais, ouais. Puis j'ai toujours] aimé ce que je faisais. Y en a quand ils vont au travail « ooohhh » *(rires)*. Non, non, moi au contraire, non, non j'aimais bien. Il m'arrivait de prendre des vacances, 15 jours comme ça, par année. Mais qu'est-ce que j'étais content quand je revenais *(rires)*. Parce qu'on peut s'embêter hein pendant les vacances. Surtout que ma femme, les vacances c'était plutôt les doigts de pieds écartés, à se dorer au soleil, et puis voilà quoi. C'était pas des vacances actives ou bien culturelles, on aurait pu, mais non, non, non, non, farniente *(rires)*.

**C : Vous vous aviez du [plaisir à retourner travailler.**

**E :** Oui, oui, oui, oui.] Bon elle bossait avec moi, on a toujours travaillé ensemble, donc elle était aussi fatiguée, parce que bon. Elle, elle s'occupait de X *(lieu de travail)*, moi j'étais X *(lieu de travail)*, mais, X *(lieu de travail)* d'un côté c'est plus pénible parce qu'il faut se taper les clients, hein. C'est pas toujours facile. Y en a qui sont pénibles. Tandis qu'en X *(lieu de travail)* ben bon, bien sûr c'est pas facile hein c'est, il faut chaud, il fait, y a le stress du X *(particularité du travail)*, et tout ça, mais, non, c'est pas la même chose. Moi j'aurais pas pu faire les clients, parce qu'au bout d'un moment ils, ils vous gonflent *(rires)*. Il faut avoir le caractère mieux fait que la figure, parce que sinon vous y arrivez pas, c'est. Non, non, c'est.

**C : A part si, à part si vous aviez autre chose à, à ajouter, je pense qu'on peut.**

**E :** Non, non, rien de rien.

**C : On peut terminer.**

**E :** Ouais, c'est, si vous voulez venir me revoir une fois y a pas de problèmes *(rires)*. Je vous dirais oui, mais, non, j'ai pas. Mais c'est possible que dans les années à venir, tout à coup, y a quelque chose, on sait pas, hein. On sait pas de, de quoi on va souffrir, mais pour le moment hein, je touche du bois, mais, ouais, tout marche bien.

**C : Pour le moment tout, tout va bien.**

**E :** Voilà. Dernièrement, y a, y a six ou sept mois, j'avais mal à la gorge, voilà. Alors j'ai été à la permanence. Ils m'ont dit : « ben écoutez y a rien à faire. Vous attendez, ça va partir comme c'est venu »*(rires).* Voilà. J'ai pas eu de médicaments, rien du tout *(rires)*. J'espérais qu'ils allaient me refiler un truc, non, non. Non, on m'a dit : « non, non, non ». Je toussais, ouais, je toussais. Puis ils ont dit : « oh, vous avez attrapé un virus, enfin un petit truc qui va partir comme il est venu ». J'ai dit : « merci. Je reviendrai » *(rires).* D'autant plus que j'ai été au, à la permanence mais en urgence quoi, enfin au truc en bas là, c'était un samedi alors j'ai attendu trois heures de temps *(rires)*. J'étais plus malade en sortant qu'en rentrant, quoi.

**C : Pour l'instant tout, tout va bien.**

**H :** Oui.

**C : Je vais arrêter. Merci de, d'avoir participé.**

**E :** Ça a duré combien de temps ?

**C : 50 minutes, voilà.**

**E :** Oh 50 minutes, oh ben ça va alors.

**C : On peut, on peut arrêter alors l'enregistreur *(rires)*.**

**E :** Voilà *(rires)*.

**C : Voilà.**
